# Author Correction: Lactylation-driven FTO targets CDK2 to aggravate microvascular anomalies in diabetic retinopathy

**DOI:** 10.1038/s44321-025-00238-y

**Published:** 2025-04-25

**Authors:** Xue Chen, Ying Wang, Jia-Nan Wang, Yi-Chen Zhang, Ye-Ran Zhang, Ru-Xu Sun, Bing Qin, Yuan-Xin Dai, Hong-Jing Zhu, Jin-Xiang Zhao, Wei-Wei Zhang, Jiang-Dong Ji, Song-Tao Yuan, Qun-Dong Shen, Qing-Huai Liu

**Affiliations:** 1 Department of Ophthalmology, The First Affiliated Hospital of Nanjing Medical University, Nanjing Medical University, Nanjing, China; 2 Department of Ophthalmology, The Affiliated Suqian First People’s Hospital of Nanjing Medical University, Suqian, China; 3 Department of Polymer Science and Engineering and Key Laboratory of High-Performance Polymer Materials and Technology of MOE, School of Chemistry and Chemical Engineering, Nanjing University, Nanjing, China

## Abstract

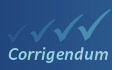

**Correction to:** *EMBO Molecular Medicine* (2024) 16:294–318. 10.1038/s44321-024-00025-1 | Published online 31 January 2024

The authors contacted the journal after detecting an error in their paper.

The authors provided the original source data for the affected figure. Upon review of the source data, the journal agrees to withdraw and replace Figure 3C.

**Figure 3C is withdrawn and replaced**.


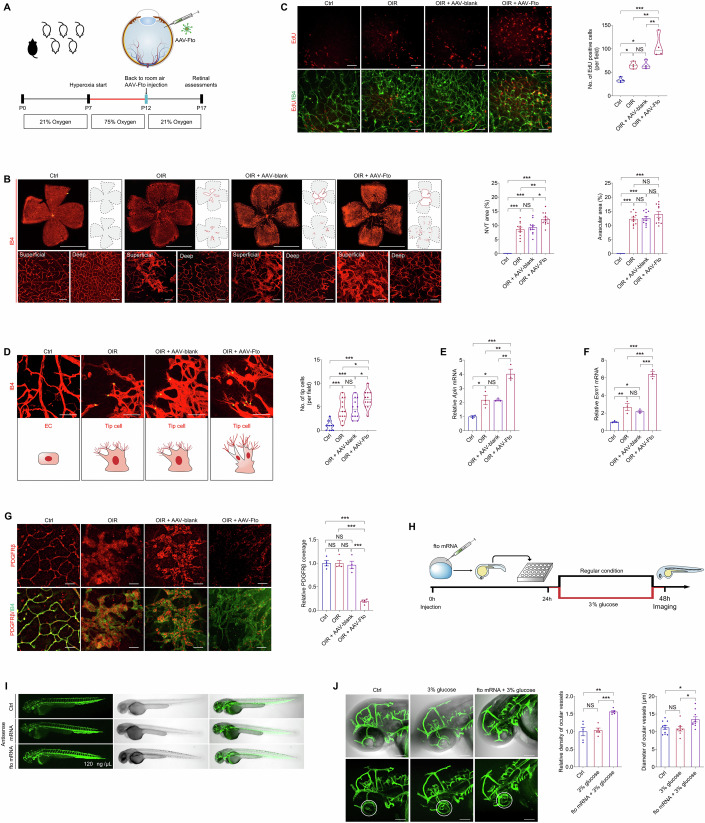


**Figure 3C. Original.**


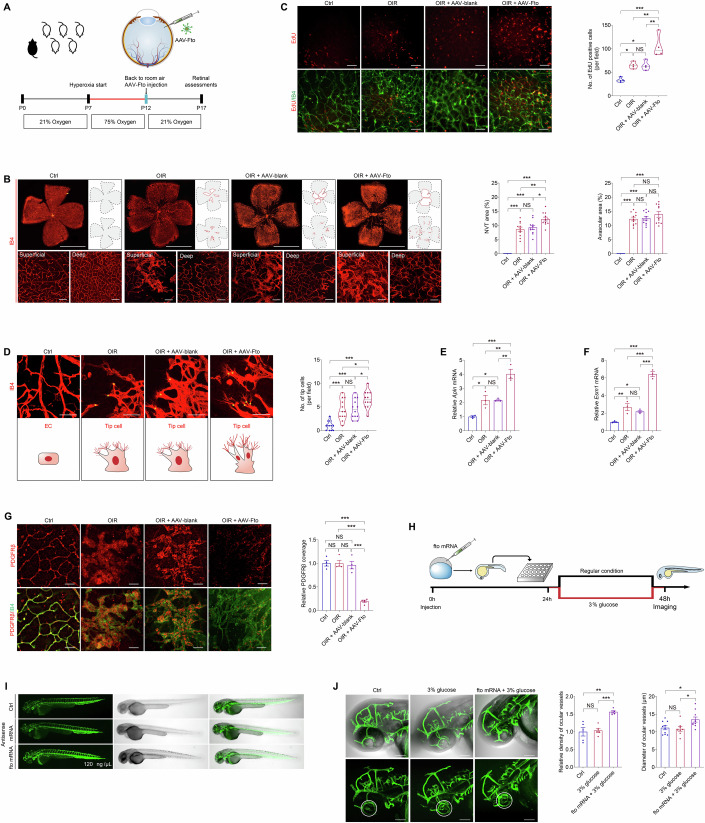


**Figure 3C. Corrected.**

Source data are available online for this figure.

**Corresponding source data for the revised Figure 3C is published with this author correction**.

Author statement:

Upon reviewing our published work, we identified an error in Figure 3C, where an unintended overlap occurred between images from the OIR group and the OIR+AAV-blank group. This error resulted from a misclassification of images during the figure assembly process.

To address this issue, we have carefully re-examined our original data and assembled a corrected version of Figure 3C. We have provided the revised figure along with the corresponding source data for verification.

All authors acknowledge this error and have collectively approved this correction.

This correction does not affect the conclusions drawn from Figure 3C or the overall findings of the study.

## Supplementary information

source data for Figure 3C affected images

