# Supplementary material for: Lactylation-driven FTO targets CDK2 to aggravate microvascular anomalies in diabetic retinopathy
Source: EMBO Mol Med. 2024 Jan 31;16(2):294–318. doi: 10.1038/s44321-024-00025-1 (PMC10897304; doi:10.1038/s44321-024-00025-1)
Supplement: Supplementary file 1 — Appendix [file 44321_2024_25_MOESM1_ESM.pdf]

Lactylation-driven FTO targets CDK2 to aggravate microvascular anomalies in diabetic retinopathy

APPENDIX

CONTENTS

Appendix Figures

Appendix Figure S1

Appendix Figure S2

Appendix Figure S3

Appendix Figure S4

Appendix Figure S5

Appendix Figure legends

Appendix Figure S1. Cell viability of HUVECs treated with high glucose.

Appendix Figure S2. Overexpressing efficiency of L-FTO and knocking down efficiency of siRNAs targeting FTO and YTHDF2 in HUVECs.

Appendix Figure S3. FTO is evolutionarily conserved among various species.

Appendix Figure S4. Overexpressing efficiency of AAV-Fto in mouse retinal vascular endothelial cells.

Appendix Figure S5. The concentration selection of C646 in HUVECs.

Appendix Tables

Appendix Table S1. Primers used in this study.

Appendix Table S2. Antibodies used in this study.

Appendix Table S3. Sequences of siRNAs.

Appendix Figure S1

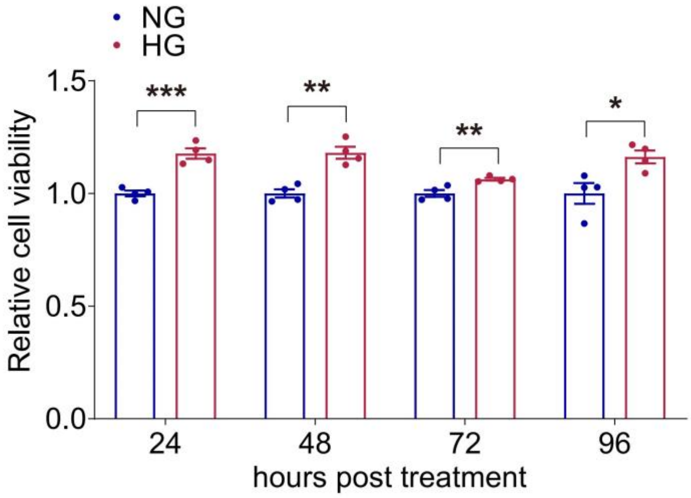

Appendix Figure S1. Cell viability of HUVECs treated with high glucose.

Cell viability of HUVECs at 24, 48, 72 and 96 hours post normal/high glucose treatment was detected by the CCK-8 assay. *n*=4 per group.

Data information: Data represent different numbers (*n*) of biological replicates. Data are shown as mean ± SEM. Two-tailed Student's t test is used. *$p<0.05$; **$p<0.01$; and ***$p<0.001$.

Source data are available online for this figure.

Appendix Figure S2

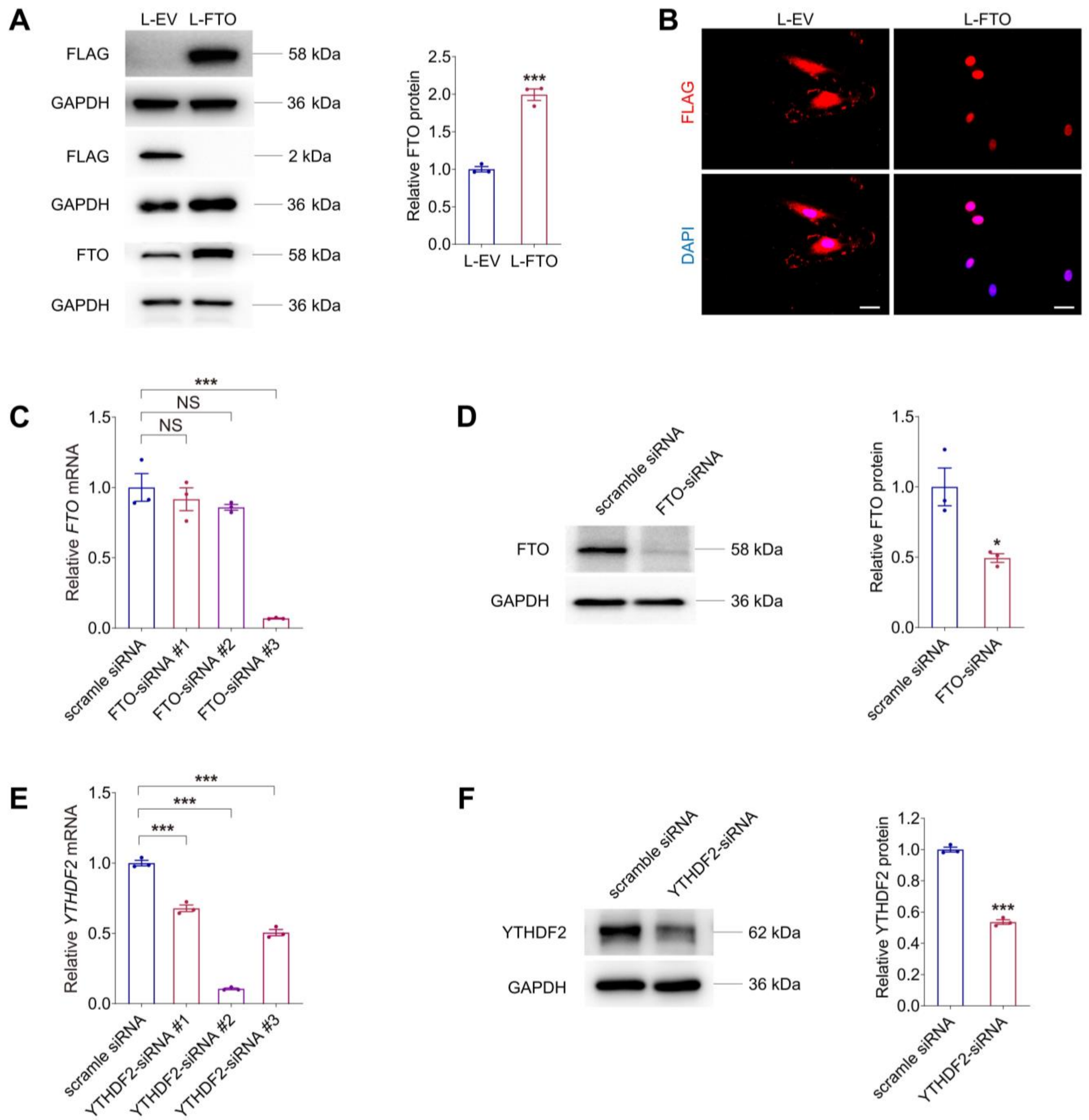

Appendix Figure S2. Overexpressing efficiency of L-FTO and knocking down efficiency of siRNAs targeting FTO and YTHDF2 in HUVECs.

A. Immunoblotting of FTO and FLAG in HUVECs transduced with L-EV or L-FTO. GAPDH is used as an internal control. *n*=3 per group.

B. Immunofluorescence staining of FLAG in HUVECs transduced with L-EV or L-FTO. Scale bar: 20 μm.

C. *FTO* mRNA level detected by qPCR in HUVECs transfected with distinct siRNAs. *n*=3 per group.

D. Immunoblotting of FTO in HUVECs transfected with scramble siRNA or FTO-siRNA-3. GAPDH is used as an internal control. *n*=3 per group.

E. *YTHDF2* mRNA level detected by qPCR in HUVECs transfected with distinct siRNAs. *n*=3 per group.

F. Immunoblotting of YTHDF2 in HUVECs transfected with scramble siRNA or YTHDF2-siRNA-2. GAPDH is used as an internal control. *n*=3 per group.

Data information: Data represent different numbers (*n*) of biological replicates. Data are shown as mean ± SEM. Two-tailed Student's t test for **A**, **D** and **F**, one-way ANOVA followed by Bonferroni's test for **C** and **E.** NS: not significant ($p>0.05$); *$p<0.05$; and ***$p<0.001$

Source data are available online for this figure.

Appendix Figure S3

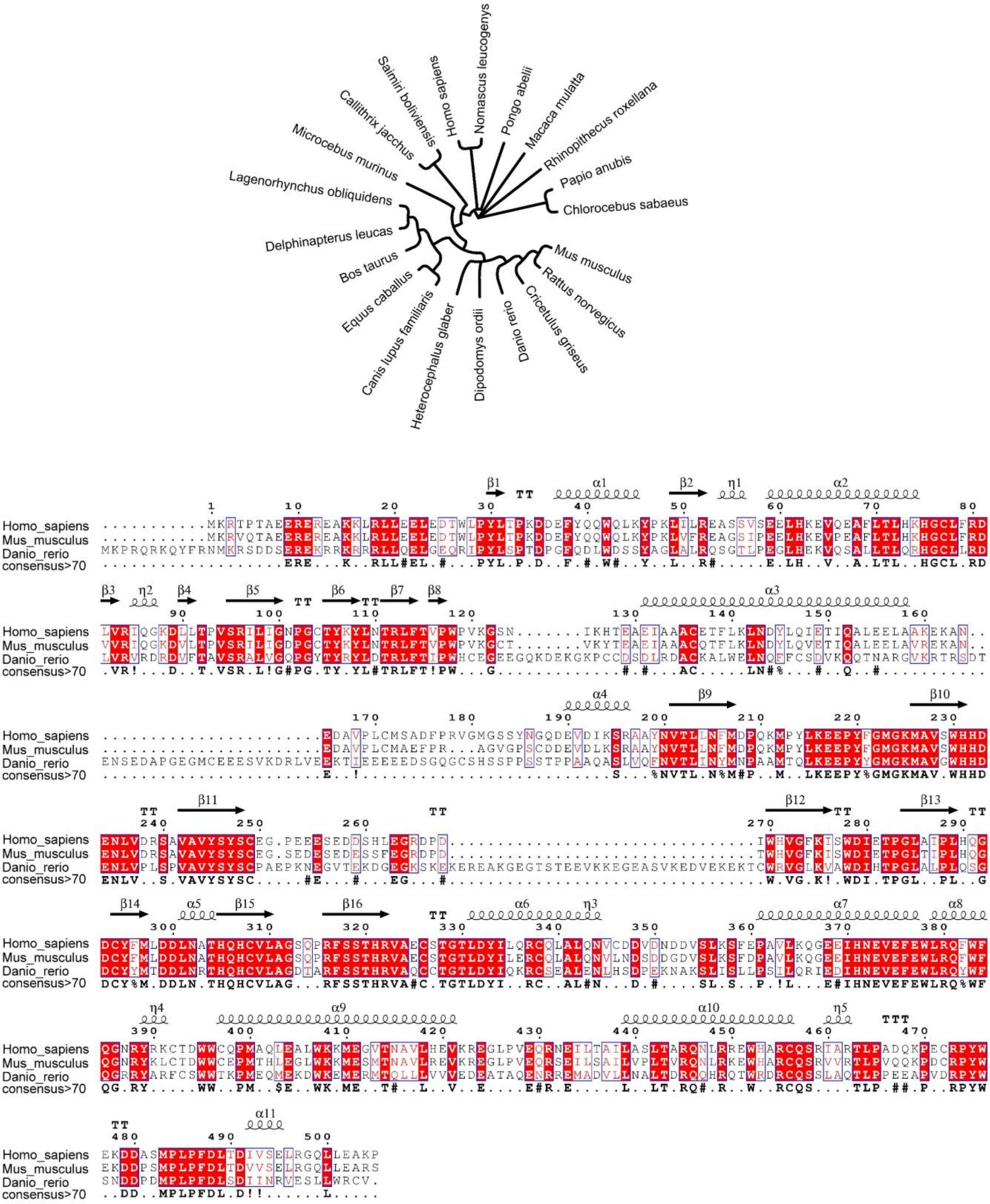

Appendix Figure S3. FTO is evolutionarily conserved among various species.

The phylogenetic tree is constructed with Evolview. The FTO from different species are clustered and divided into distinct subfamilies.

Appendix Figure S4

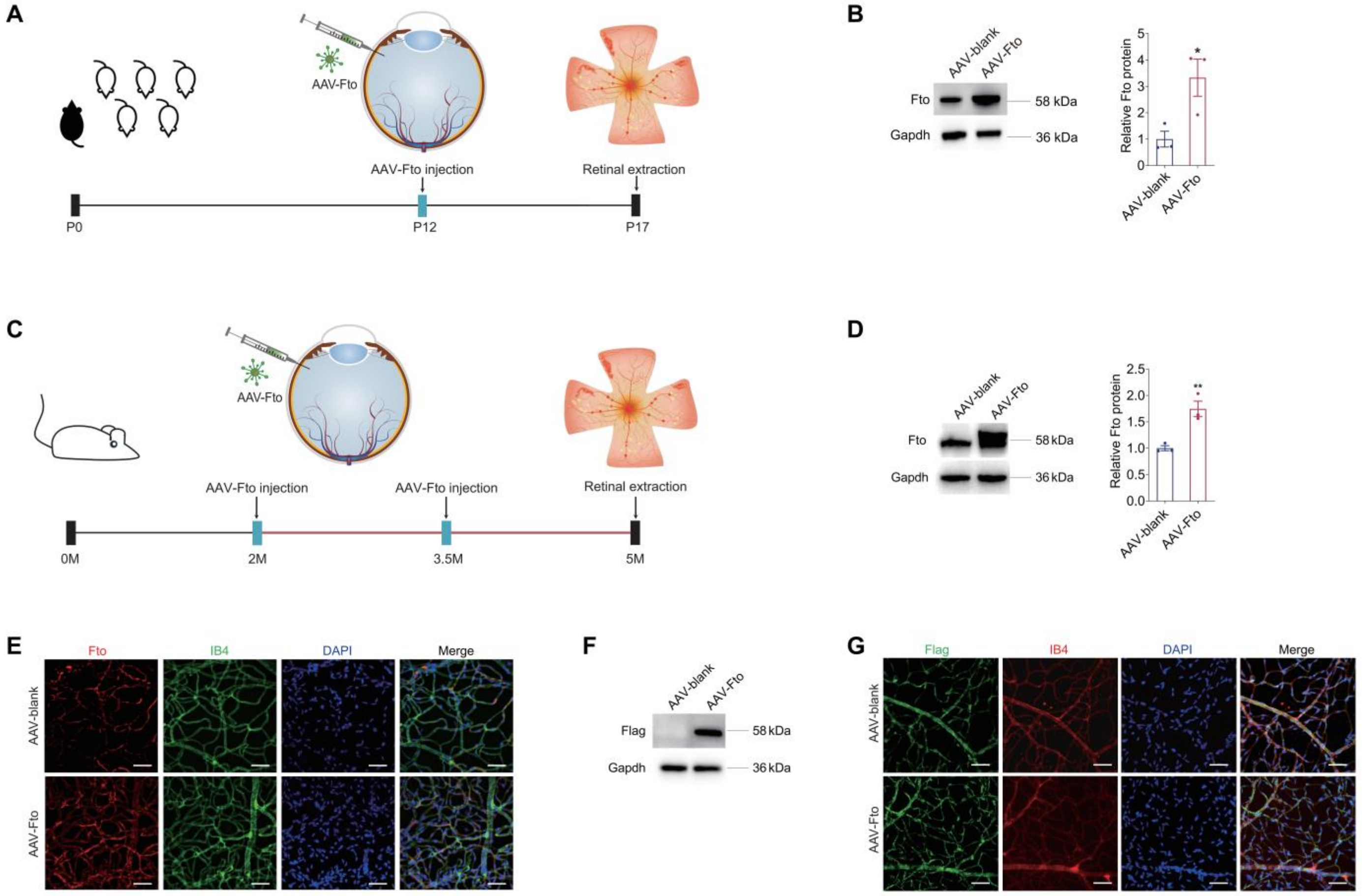

Appendix Figure S4. Overexpressing efficiency of AAV-Fto in mouse retinal vascular endothelial cells.

A. Experimental scheme for intra-vitreal AAV-Fto injection in infant mice.

B. Immunoblotting of Fto in neural retinas of P17 mice intra-vitreal injected with AAV-Fto or AAV-blank. Gapdh is used as an internal control. *n*=3 per group.

C. Experimental scheme for intra-vitreal AAV-Fto injection in adult mice.

D. Immunoblotting of Fto in neural retinas of 5-month-old mice intra-vitreal injected with AAV-Fto or AAV-blank. Gapdh is used as an internal control. *n*=3 per group.

E. Immunofluorescence staining of FTO and IB4 in trypsin digested retinal vasculatures originated from mice receiving intra-vitreal AAV-Fto or AAV-blank injection. Cell nuclei are counterstained with DAPI. Scale bar: 50 µm.

F. Immunoblotting of Flag in neural retinas of mice intra-vitreal injected with AAV-blank or AAV-Fto. Gapdh is used as an internal control.

G. Immunofluorescence staining of Flag and IB4 in trypsin digested retinal vasculatures originated from mice intra-vitreal injected with AAV-Fto or AAV-blank. Cell nuclei are counterstained with DAPI. Scale bar: 50 µm.

Data information: Data represent different numbers (*n*) of biological replicates. Data are shown as mean ± SEM. Two-tailed Student's t test is used. *p<0.05; **p<0.01.

Source data are available online for this figure.

Appendix Figure S5

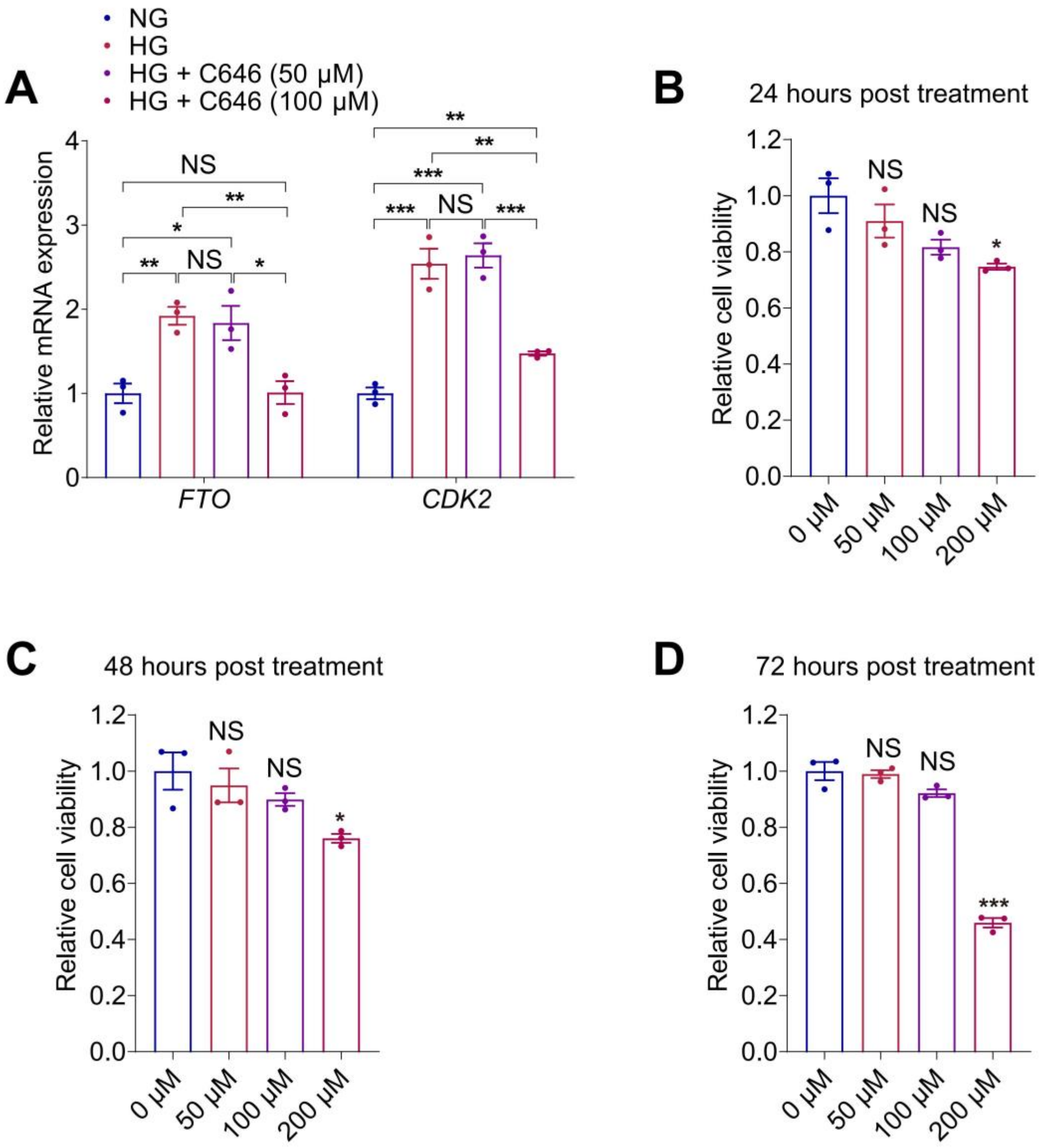

Appendix Figure S5. The concentration selection of C646 in HUVECs.

A. qPCR presents mRNA levels of *FTO* and *CDK2* in HUVECs receiving distinct treatments. *n*=3 per group.

B-D. CCK-8 assay demonstrates the cell viability of HUVECs added with C646 at distinct concentrations (0, 50, 100 and 200 μM) for 24 **(B)**, 48 **(C)** and 72 **(D)** hours. *n*=3 per group.

Data information: Data represent different numbers (*n*) of biological replicates. Data are shown as mean ± SEM. One-way ANOVA followed by Bonferroni's test is used. NS: not significant (p>0.05); *p<0.05; and ***p<0.001.

Source data are available online for this figure.

Table S1. Primers used in this study.

Gene/RNA	Forward primer (5'→3')	Reverse primer (5'→3')
In vitro		
METTL3	TTGTCTCCAACCTTCCGTAGT	CCAGATCAGAGAGGTGGTGTAG
METTL14	TGACATCAGAGAACTAACACCCA	GATCGAGGTGCTGCAATCTC
WTAP	TGAACTGAGTGCCTGGAAGT	CATCTCTTGTTCCTTGGTTGCT
ALKBH5	TCAGGAAGACAAGATTAGATGCAC	TCCTTGTCCATCTCCAGGAT
FTO	TCATAATGAGGTCGAGTTTGAGTG	TCATGAAGCACAGCATTTGTC
GAPDH	ACAACTTTGGTATCGTGGAAGG	GCCATCACGCCACAGTTTC
CXCR4	ACTACACCGAGGAAATGGGCT	CCACAATGCCAGTTAAGAAGA
FSCN1	CTGCTACTTTGACATCGAGTGG	GGGCGGTTGATGAGCTTCA
APLN	GTCTCCTCCATAGATTGGTCTGC	GGAATCATCCAAACTACAGCCAG
ESM1	ACAGCAGTGAGTGCAAAAGCA	GCGGTAGCAAGTTTCTCCCC
PLAUR	TGTAAGACCAACGGGGATTGC	AGCCAGTCCGATAGCTCAGG
ITGB1	CCTACTTCTGCACGATGTGATG	CCTTTGCTACGGTTGGTTACATT
CDK2	GGCATTCCTCTTCCCCTCAT	AGTCTGCTAGCTTGATGGCC
YTHDF2	CCTTACTTGAGTCCACAGGC	TGCATCTGGTAGGAAGTGGG
In vivo		
Mettl3	CTGGGCACTTGGATTTAAGGAA	TGAGAGGTGGTGTAGCAACTT
Mettl14	GACTGGCATCACTGCGAATGA	AGGTCCAATCCTTCCCCAGAA
Wtap	ATGGCACGGGATGAGTTAATTC	TTCCCTTAAACCAGTCACATCG
Alkbh5	TTGCCACCCAGCTATGCTTC	CAGACCGCCGGTTTTCTTCTT
Fto	TTCATGCTGGATGACCTCAATG	GCCAACTGACAGCGTTCTAAG
Gapdh	AGGTCGGTGTGAACGGATTTG	TGTAGACCATGTAGTTGAGGTCA
Apln	CCATGCCTTTCTAAAGCAGGATT	GCGAGACCACGCCATTAGA
Esm1	CTGGAGCGCCAAATATGCG	TGAGACTGTACGGTAGCAGGT
Il1b	GAAATGCCACCTTTTGACAGTG	TGGATGCTCTCATCAGGACAG
Ccl2	TTAAAAACCTGGATCGGAACCAA	GCATTAGCTTCAGATTTACGGGT
Tmem119	TCTTCCGGCAGTACGTGATG	CGGCGCAGACTATGAACATGA
Trem2	CTGGAACCGTCACCATCACTC	CGAAACTCGATGACTCCTCGG
Lgals3	GGAGAGGGAATGATGTTGCCT	TCCTGCTTCGTGTTACACACA
Cd11c	CTGGATAGCCTTTCTTCTGCTG	GCACACTGTGTCCGAACTCA
Tubb3	TGTTCAGCAGCTAAGGAAGAG	GCTCTTGGGTCATGCACTTC

**Table S2.** Antibodies used in this study.				
Anti-protein	Host	Dilution and Application	Supplier	Catalog
In vitro				
m6A	Rabbit	1:1000, dot blot;<br>1:100, MeRIP-qPCR	Merck	ABE572
m6A	Rabbit	1 μg antibody/100 μg RNA, MeRIP-Seq	Synaptic system	SS-202 003-50
METTL3	Rabbit	1:1000, Immunoblotting	Invitrogen	PA5-28178
GAPDH	Mouse	1:2000, Immunoblotting	Proteintech	60004-1-Ig
ALKBH5	Rabbit	1:1000, Immunoblotting	Abcam	ab195377
FTO	Rabbit	1:1000, Immunoblotting,<br>1:50, RIP	Proteintech	27226-1-AP
FLAG	Rabbit	1:1000, Immunoblotting<br>1:100, Immunofluorescence	Beyotime	AF0036
cleaved caspase-3	Rabbit	1:500, Immunoblotting	Abcam	ab2302
IBA-1	Rabbit	1:150, Immunofluorescence	Abcam	ab178846
TMEM119	Rabbit	1:100, Immunofluorescence	Abcam	ab209064
CDK2	Rabbit	1:1000, Immunoblotting	Abcam	ab32147
β-actin	Rabbit	1:1000, Immunoblotting	Beyotime	AF5003
YTHDF2	Rabbit	1:50, RIP	Proteintech	24744-1-AP
H3K18la	Rabbit	1:1000, Immunoblotting;<br>1:100, RIP	PTM Bio	PTM-1406RM
H3	Rabbit	1:1000, Immunoblotting	Proteintech	17168-1-AP
In vivo				
Mettl3	Rabbit	1:1000, Immunoblotting	Invitrogen	PA5-28178
Gapdh	Mouse	1:10000, Immunoblotting	Proteintech	60004-1-Ig
Alkbh5	Rabbit	1:1000, Immunoblotting	Abcam	ab195377
Fto	Rabbit	1:1000, Immunoblotting;<br>1:100, Immunofluorescence	Abcam	27226-1-AP
Flag	Rabbit	1:1000, Immunoblotting<br>1:100, Immunofluorescence	Beyotime	AF0036
Ib4	/	1:100, Immunofluorescence	Vector Laborato ries	FL-1201-.5&<br>DL-1207-.5
Pdgfrβ	Rabbit	1:100, Immunofluorescence	Cell Signaling Technology	3169

Ve-cadherin	Rat	1:100, Immunofluorescence	Abcam	ab282277
Zo-1	Rabbit	1:1000, Immunoblotting	Invitrogen	#61-7300
Ng2	Rabbit	1:100, Immunofluorescence	Abcam	ab275024
Iba-1	Rabbit	1:150, Immunofluorescence	Abcam	ab178846
Tubb3	Rabbit	1:100, Immunofluorescence	Abcam	ab18207
Cdk2	Rabbit	1:1000, Immunoblotting	Proteintech	10122-1-AP

Table S3. Sequences of siRNAs.

Targeted genes	Sequence (5'→3')
scramble siRNA	sense: UUCUCCGAACGUGUCACGUdTdT
	antisense: ACGUGACACGUUCGGAGAAdTdT
FTO-siRNA-1	sense: GGACCTGGTTAGGATCCAAdTdT
	antisense: UUGGAUCCUAACCAGGUCCdTdT
FTO -siRNA-2	sense: GAGCTGGCATCATGATGAAdTdT
	antisense: UUCAUCAUGAUGCCAGCUCdTdT
FTO -siRNA-3	sense: GCAGAATGTCTGTGACGATdTdT
	antisense: AUCGUCACAGACAUUCUGCdTdT
YTHDF2-siRNA-1	sense: GACCAAGAATGGCATTGCAdTdT
	antisense: UGCAAUGCCAUUCUUGGUCdTdT
YTHDF2-siRNA-2	sense: GCACAGAAGTTGCAAGCAAdTdT
	antisense: UUGCUUGCAACUUCUGUGCdTdT
YTHDF2-siRNA-3	sense: GGTAGCGGGTCCATTACTAdTdT
	antisense: UAGUAAUGGACCCGCUACCdTdT
CDK2-siRNA-1	sense: GCACCAAGAUCUCAAGAAAdTdT
	antisense: CGUGGUUCUAGAGUUCUUUdTdT
CDK2-siRNA-2	sense: GAGUCCCUGUUCGUACUUAdTdT
	antisense: CUCAGGGACAAGCAUGAAUdTdT
CDK2-siRNA-3	sense: GGAUGUGACCAAGCCAGUAdTdT
	antisense: CCUACACUGGUUCGGUCAUdTdT
