# Supplementary figures and images for: Lactylation-driven FTO targets CDK2 to aggravate microvascular anomalies in diabetic retinopathy (part 1 of 4)
Source: EMBO Mol Med. 2024 Jan 31;16(2):294–318. doi: 10.1038/s44321-024-00025-1 (PMC10897304; doi:10.1038/s44321-024-00025-1)

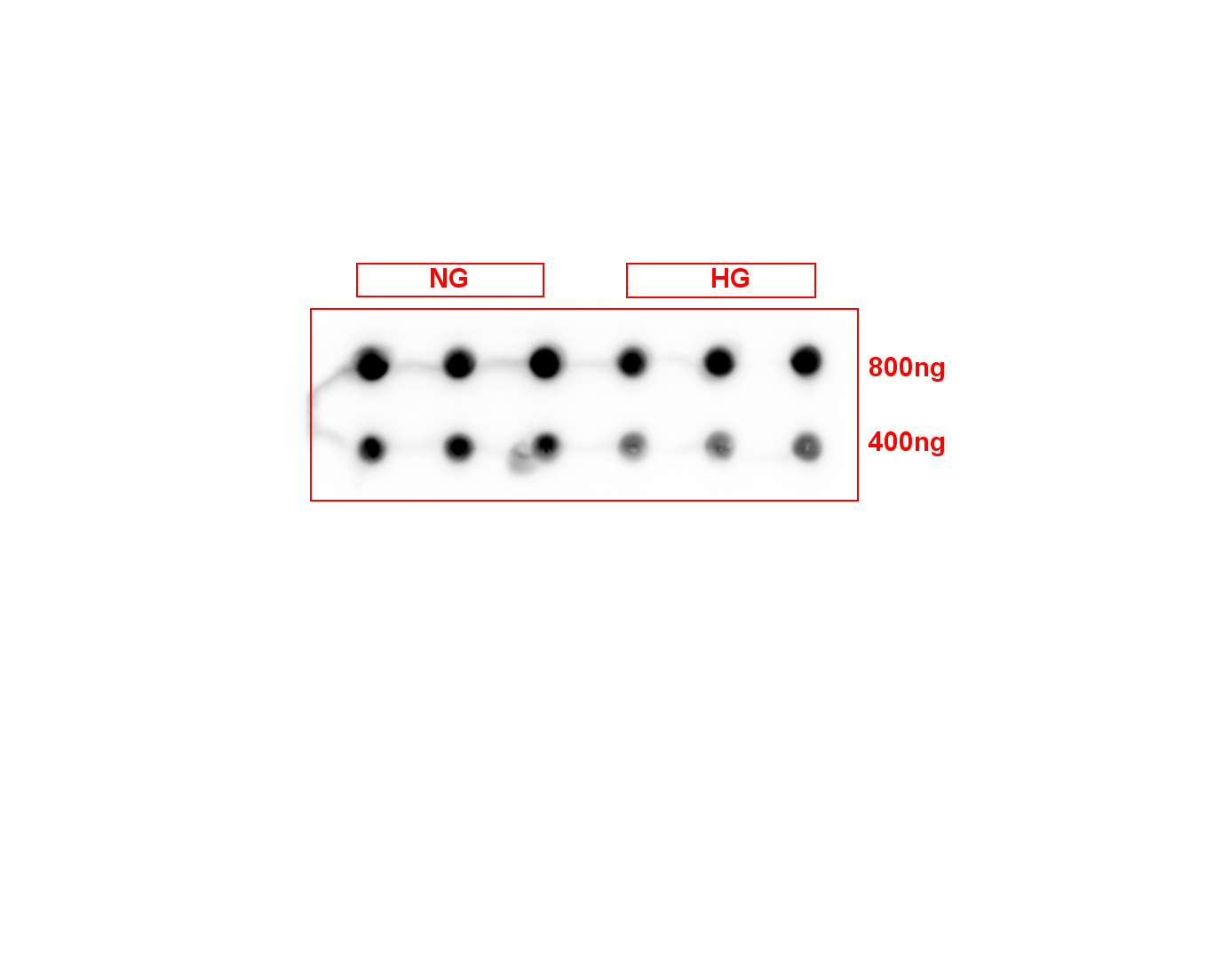

Supplement: Supplementary file 2 — Source Data Fig. 1 [file 44321_2024_25_MOESM2_ESM.zip › figure 1/1A/1A/1A m6A mark.Tif]

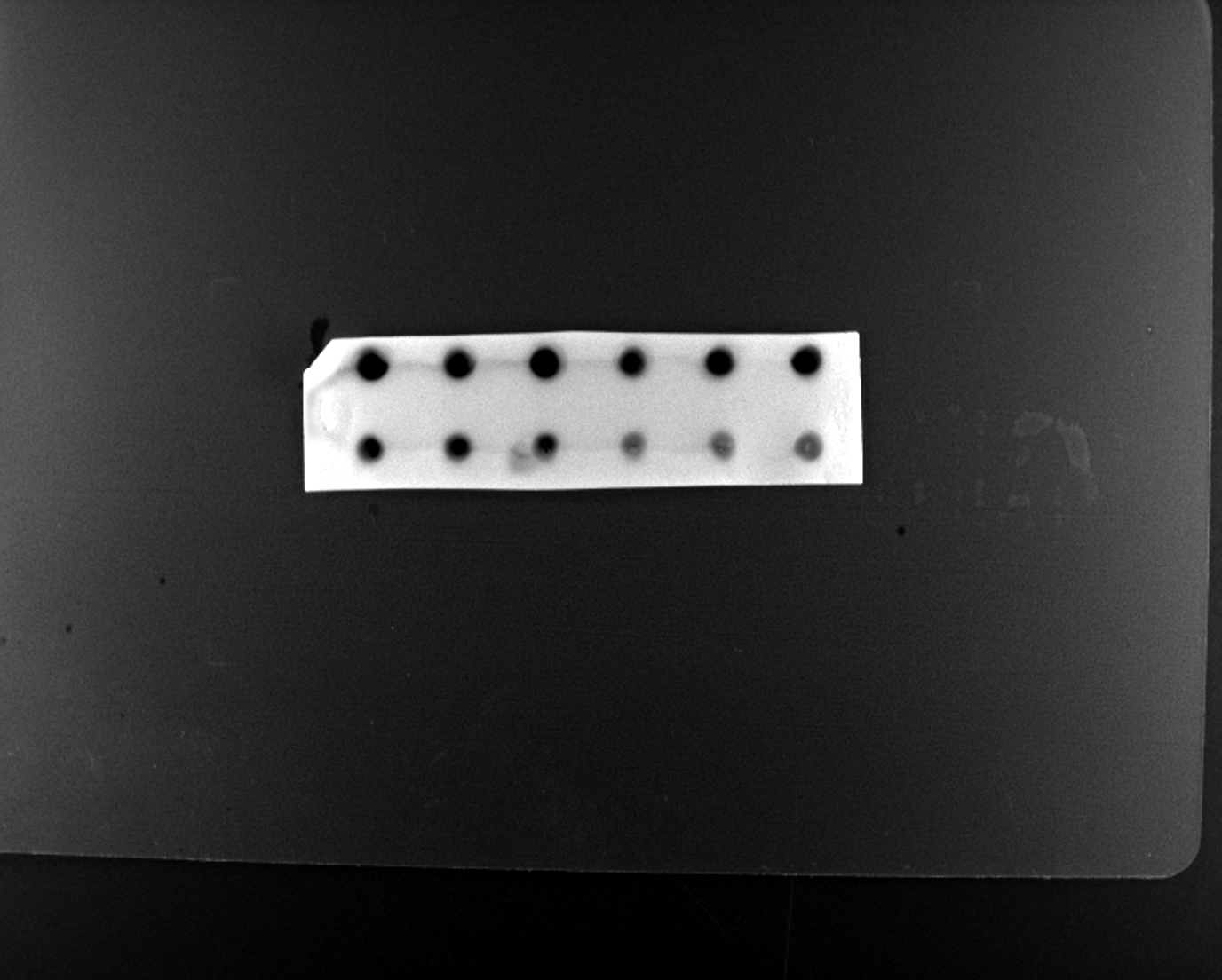

Supplement: Supplementary file 2 — Source Data Fig. 1 [file 44321_2024_25_MOESM2_ESM.zip › figure 1/1A/1A/1A m6A.Tif]

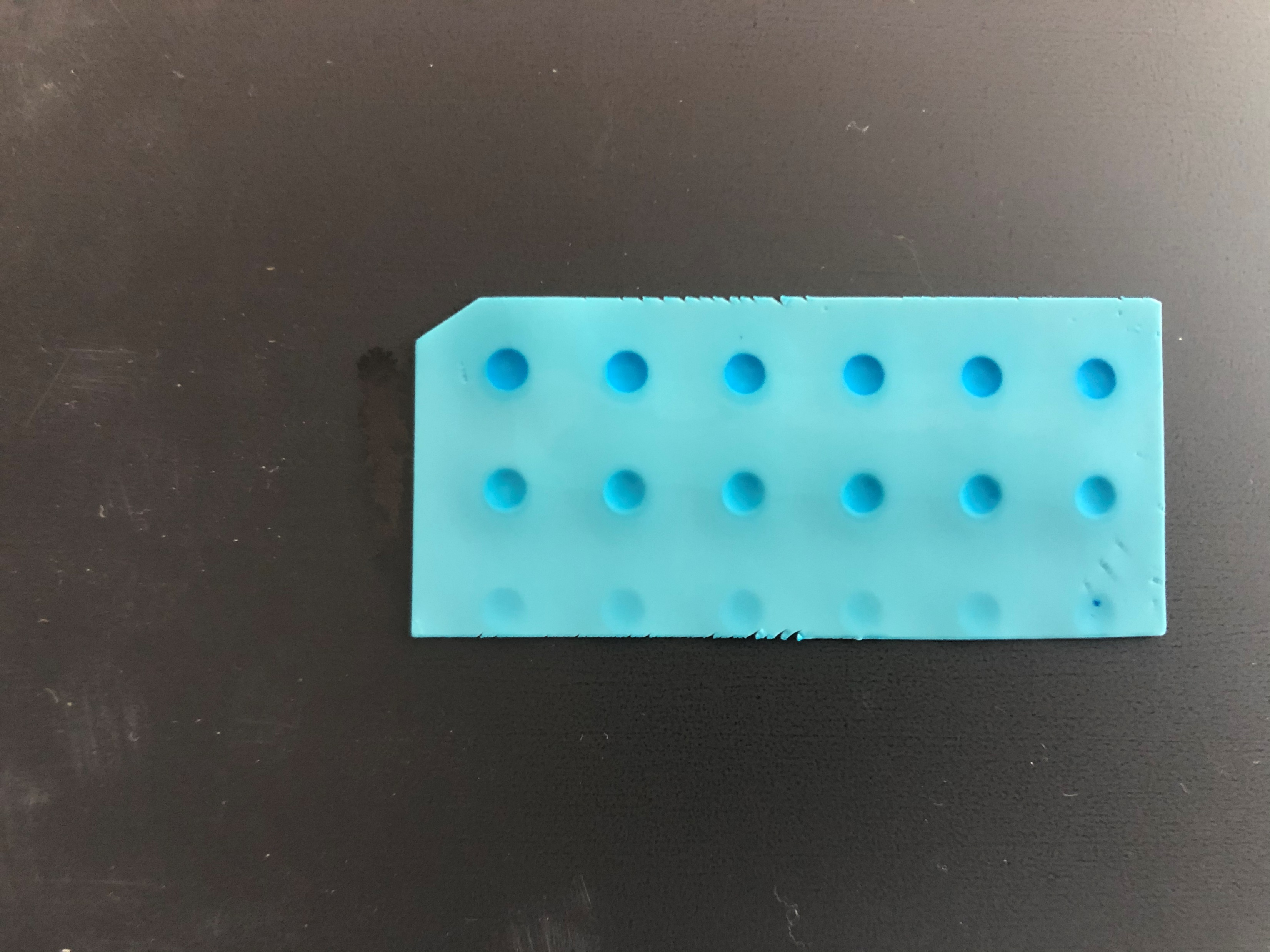

Supplement: Supplementary file 2 — Source Data Fig. 1 [file 44321_2024_25_MOESM2_ESM.zip › figure 1/1A/1A/1A MB mark.tif]

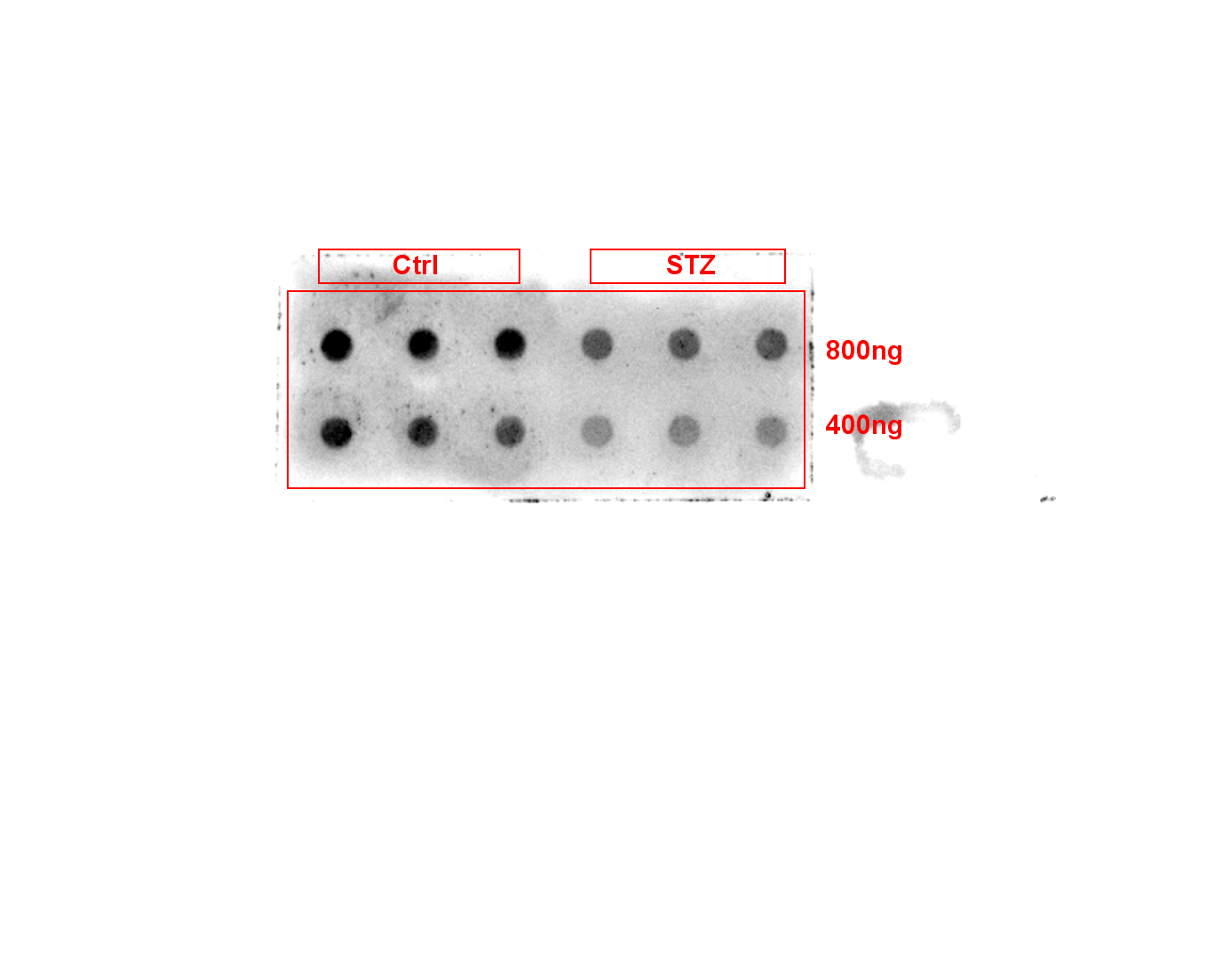

Supplement: Supplementary file 2 — Source Data Fig. 1 [file 44321_2024_25_MOESM2_ESM.zip › figure 1/1C/1C/1C m6A mark.Tif]

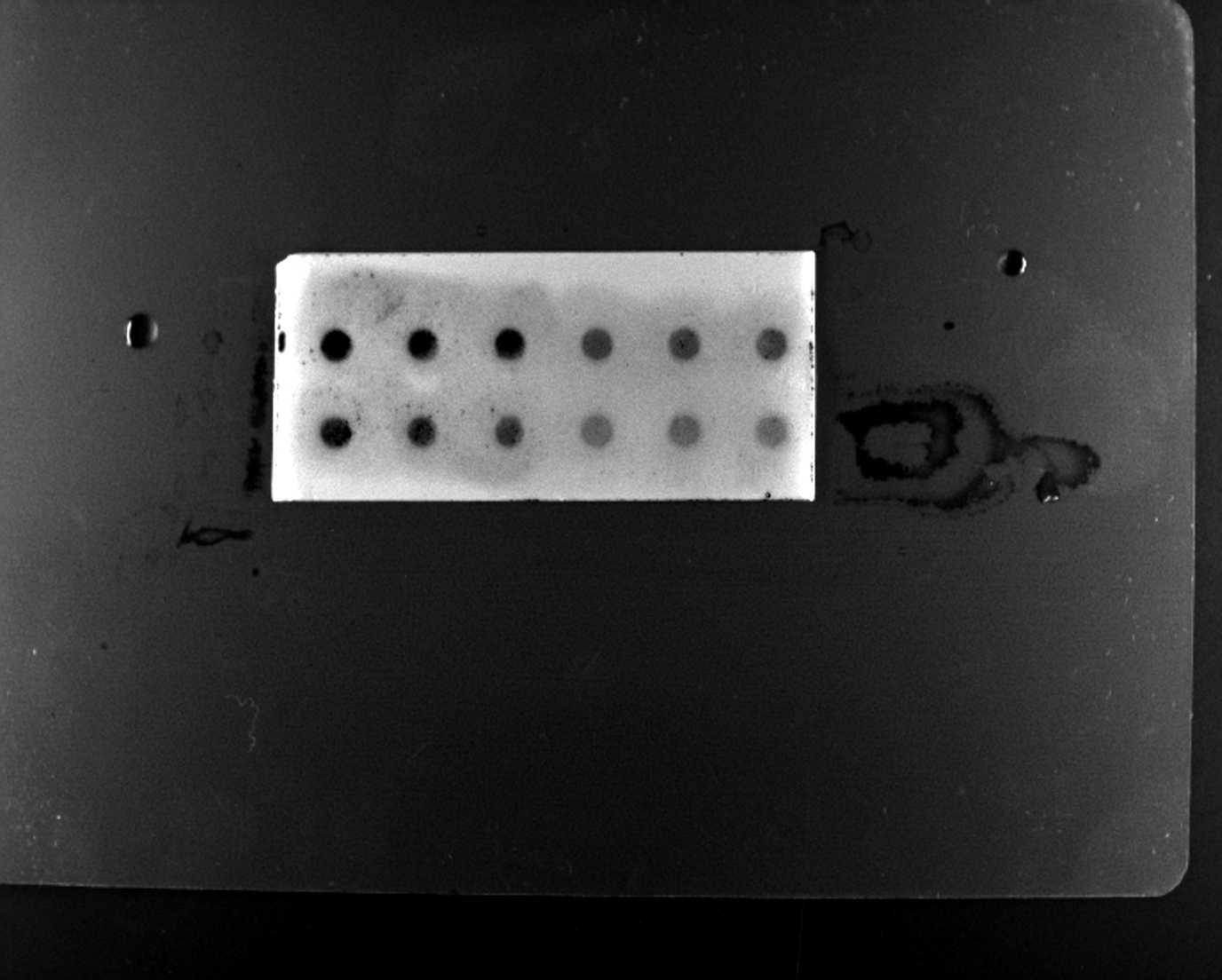

Supplement: Supplementary file 2 — Source Data Fig. 1 [file 44321_2024_25_MOESM2_ESM.zip › figure 1/1C/1C/1C m6A.Tif]

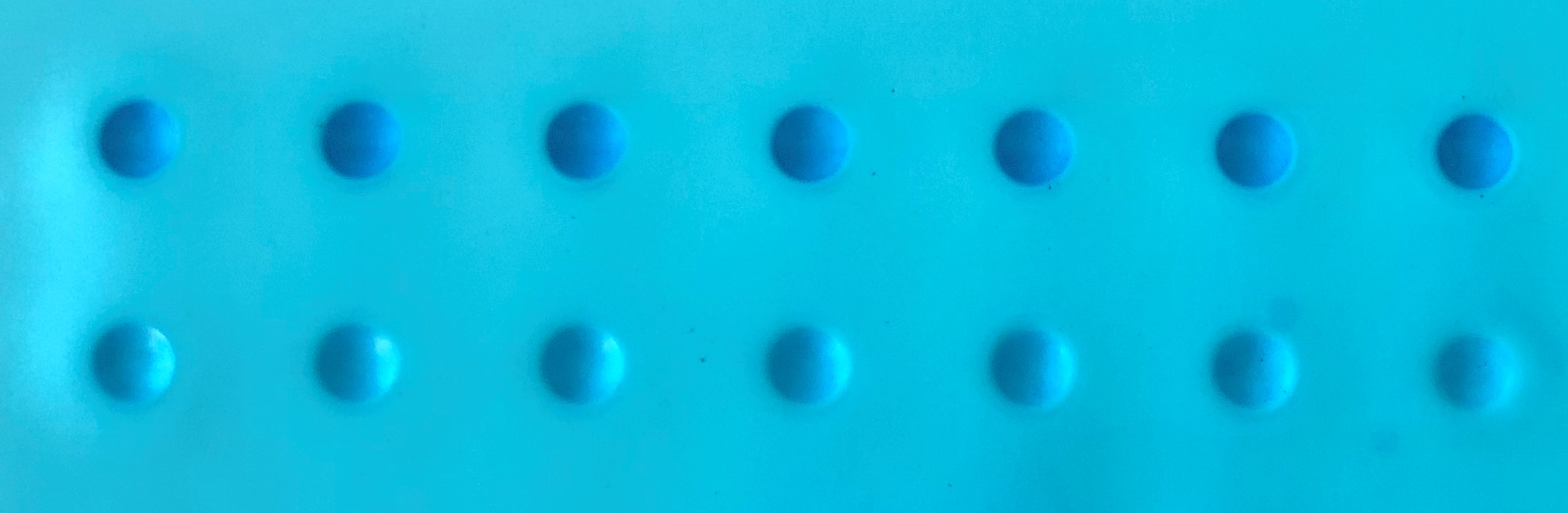

Supplement: Supplementary file 2 — Source Data Fig. 1 [file 44321_2024_25_MOESM2_ESM.zip › figure 1/1C/1C/1C MB.Tif]

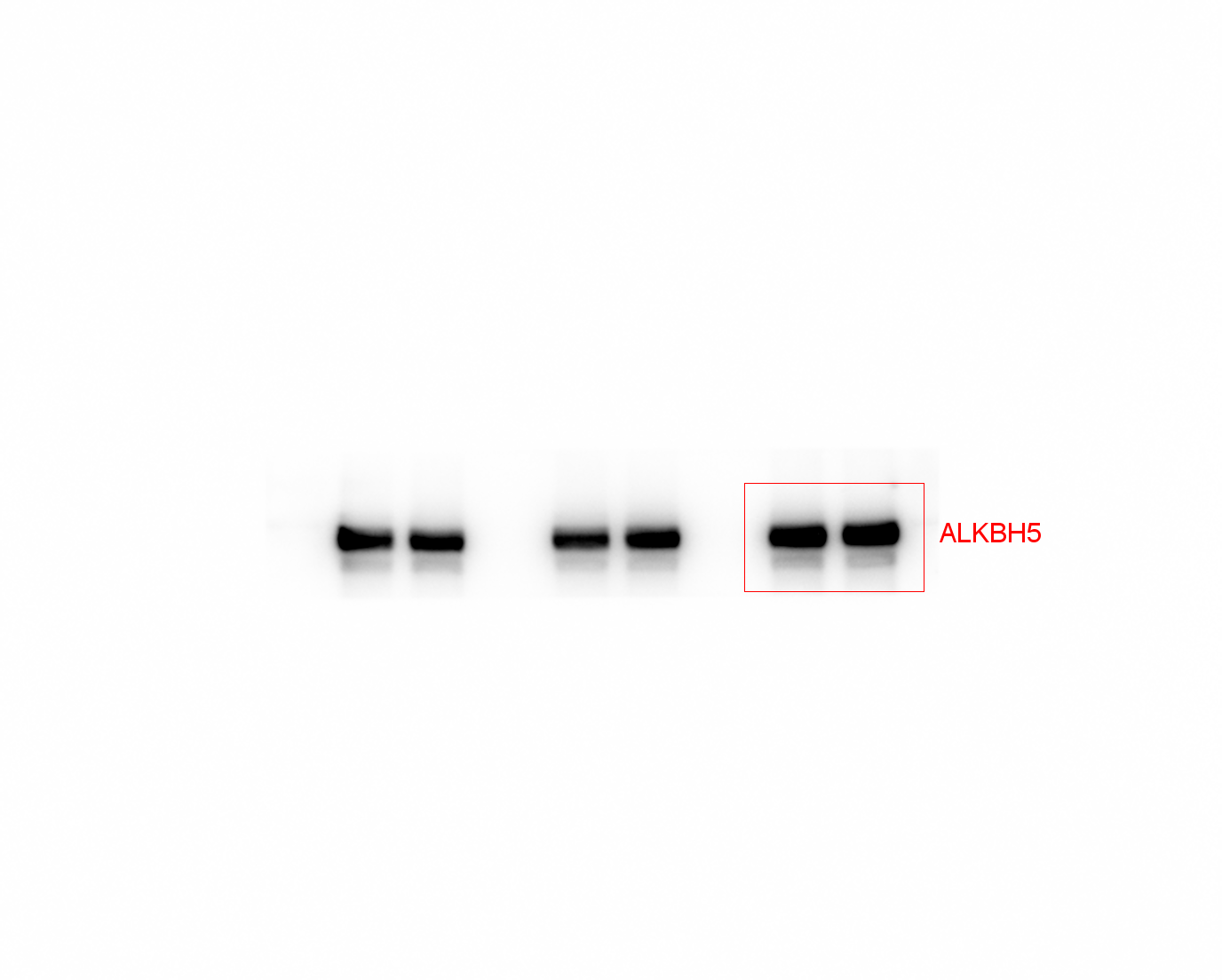

Supplement: Supplementary file 2 — Source Data Fig. 1 [file 44321_2024_25_MOESM2_ESM.zip › figure 1/1G/1G/1G ALKBH5 mark.Tif]

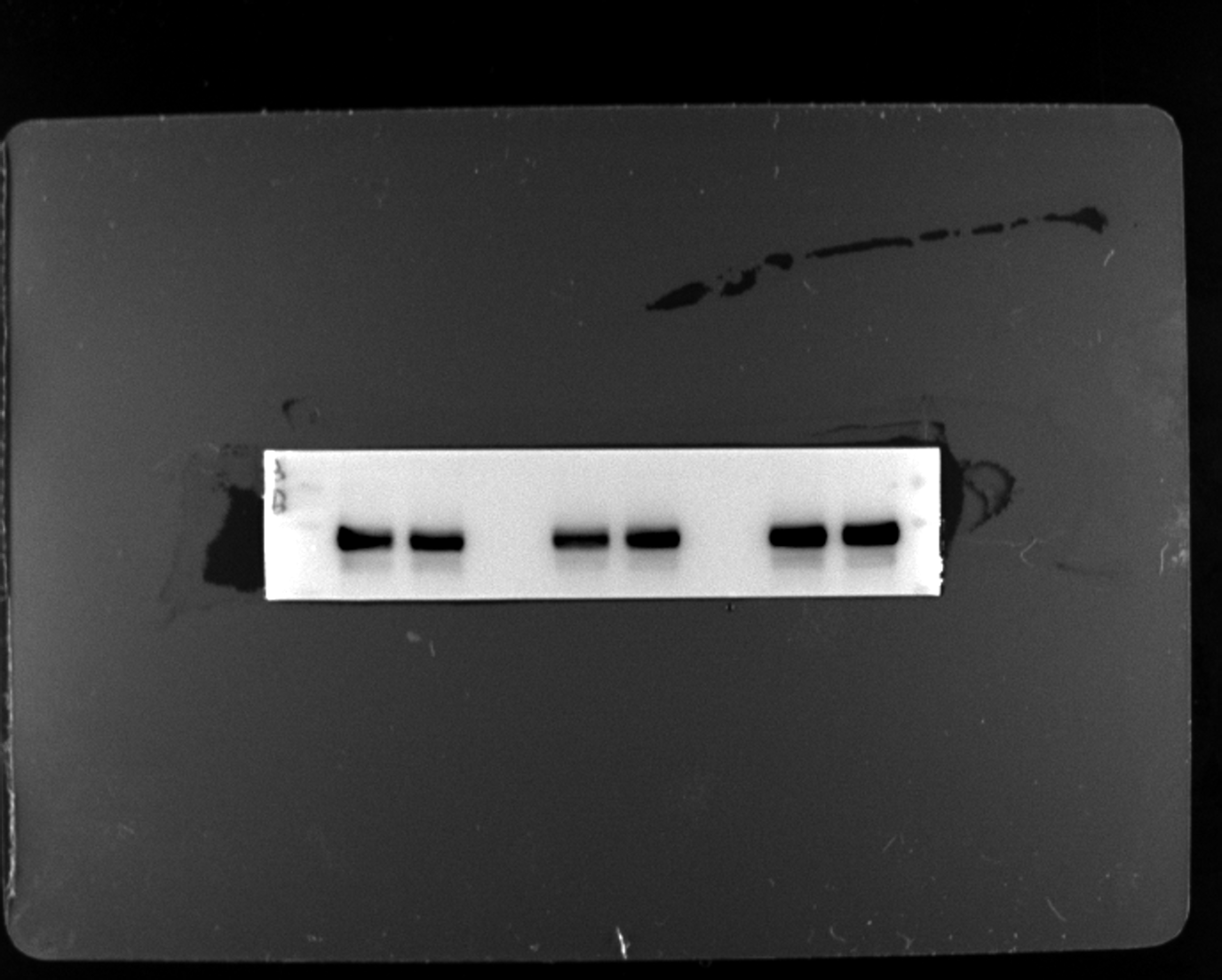

Supplement: Supplementary file 2 — Source Data Fig. 1 [file 44321_2024_25_MOESM2_ESM.zip › figure 1/1G/1G/1G ALKBH5.Tif]

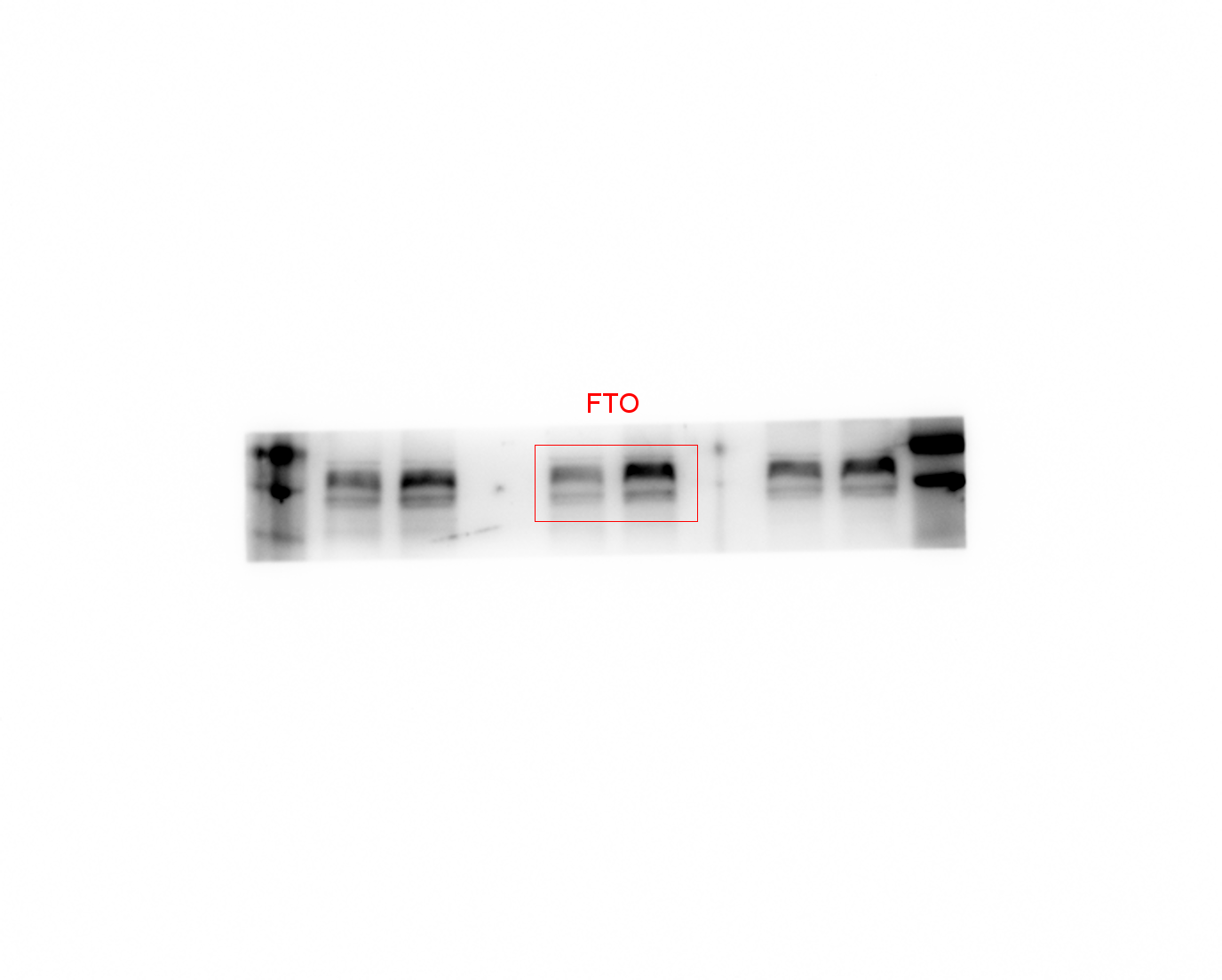

Supplement: Supplementary file 2 — Source Data Fig. 1 [file 44321_2024_25_MOESM2_ESM.zip › figure 1/1G/1G/1G FTO mark.Tif]

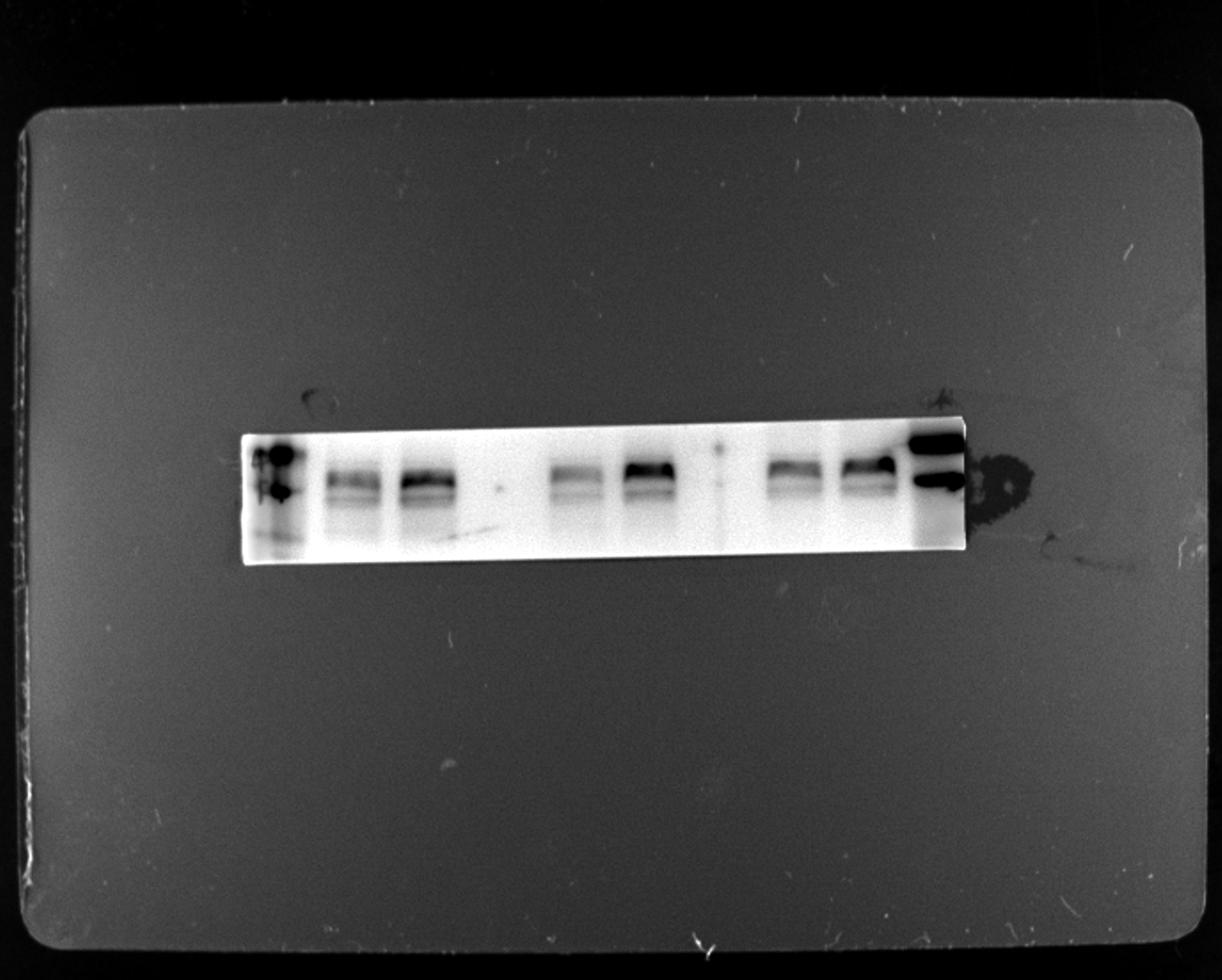

Supplement: Supplementary file 2 — Source Data Fig. 1 [file 44321_2024_25_MOESM2_ESM.zip › figure 1/1G/1G/1G FTO.Tif]

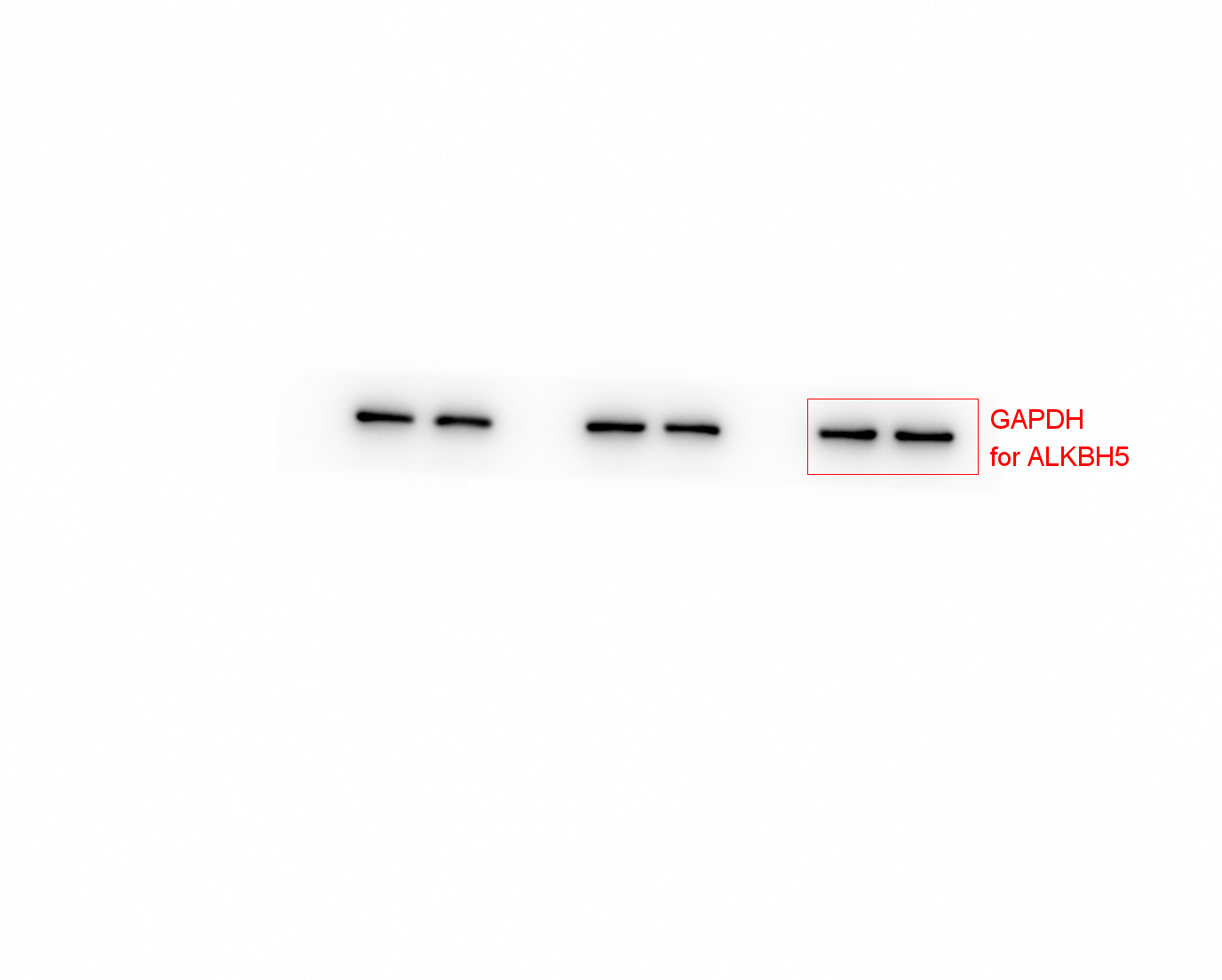

Supplement: Supplementary file 2 — Source Data Fig. 1 [file 44321_2024_25_MOESM2_ESM.zip › figure 1/1G/1G/1G GAPDH for ALKBH5 mark.Tif]

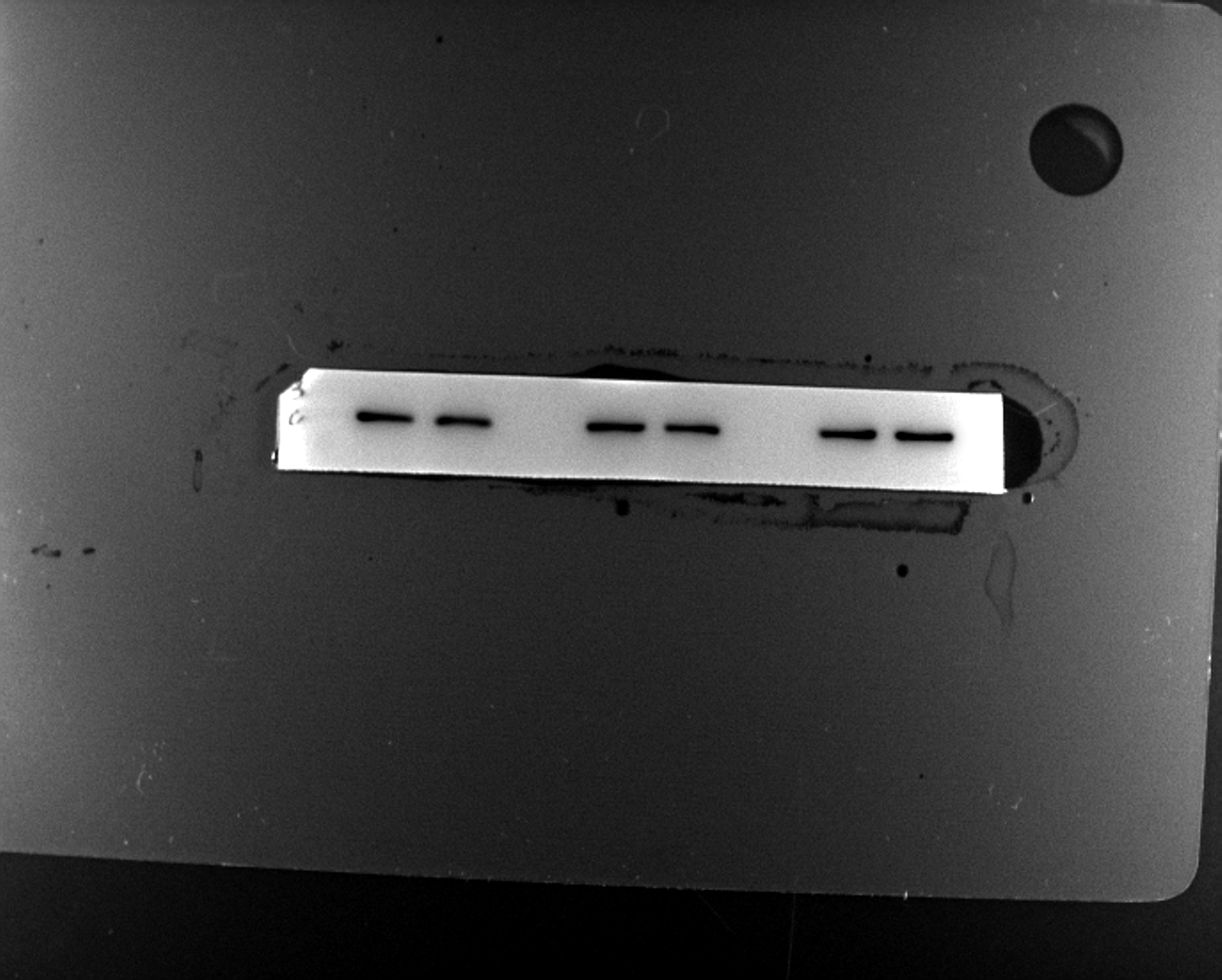

Supplement: Supplementary file 2 — Source Data Fig. 1 [file 44321_2024_25_MOESM2_ESM.zip › figure 1/1G/1G/1G GAPDH for ALKBH5.Tif]

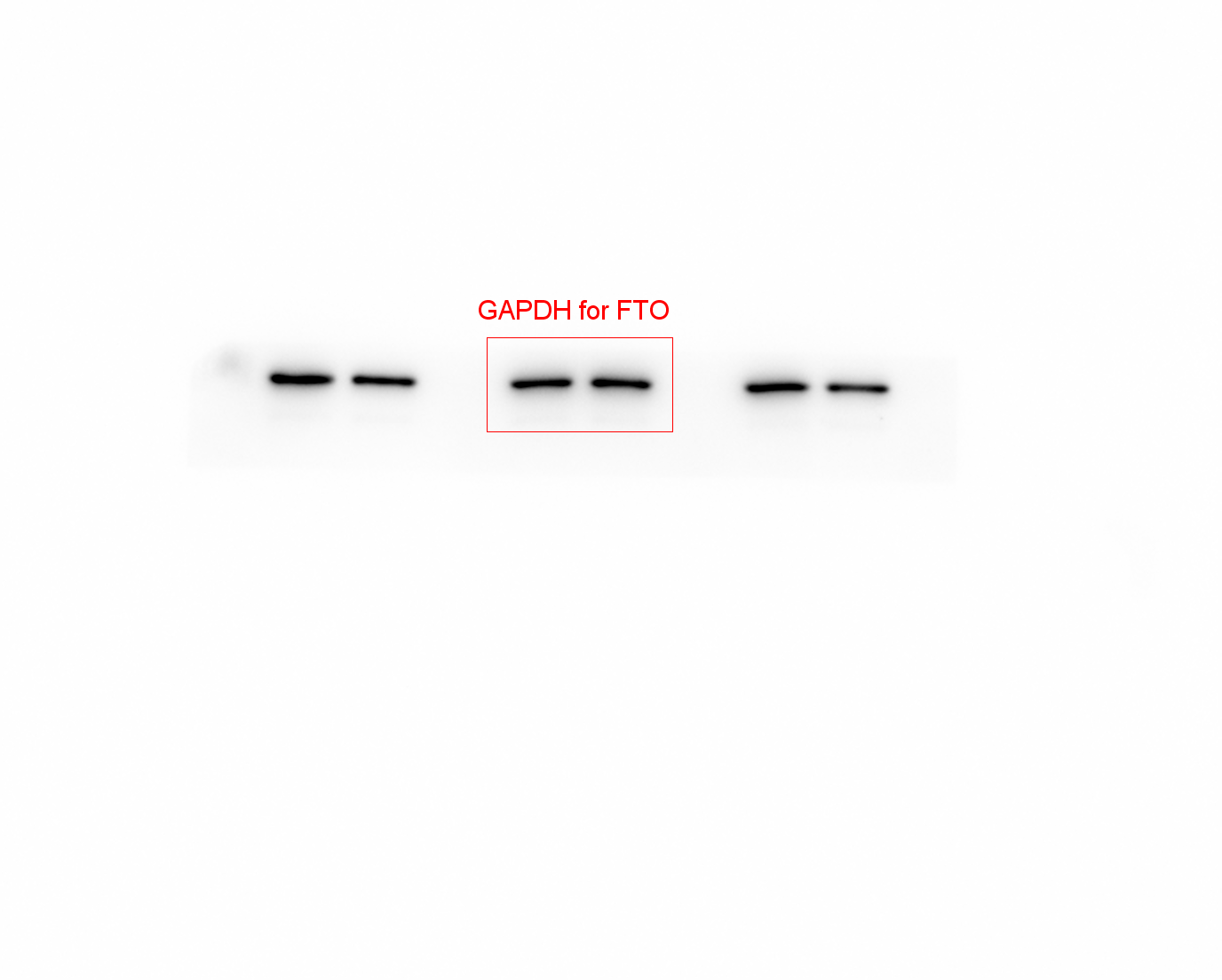

Supplement: Supplementary file 2 — Source Data Fig. 1 [file 44321_2024_25_MOESM2_ESM.zip › figure 1/1G/1G/1G GAPDH for FTO mark.Tif]

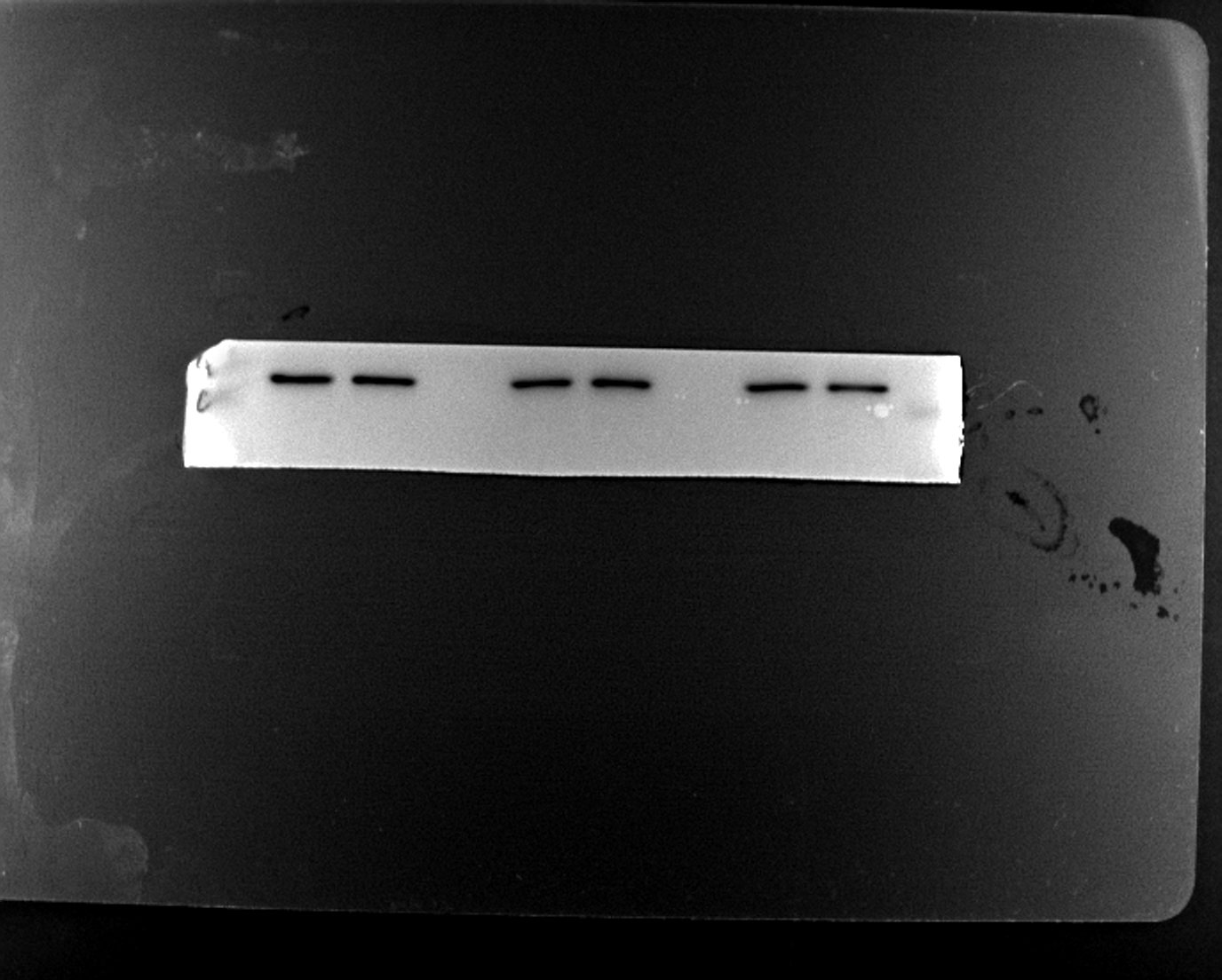

Supplement: Supplementary file 2 — Source Data Fig. 1 [file 44321_2024_25_MOESM2_ESM.zip › figure 1/1G/1G/1G GAPDH for FTO.Tif]

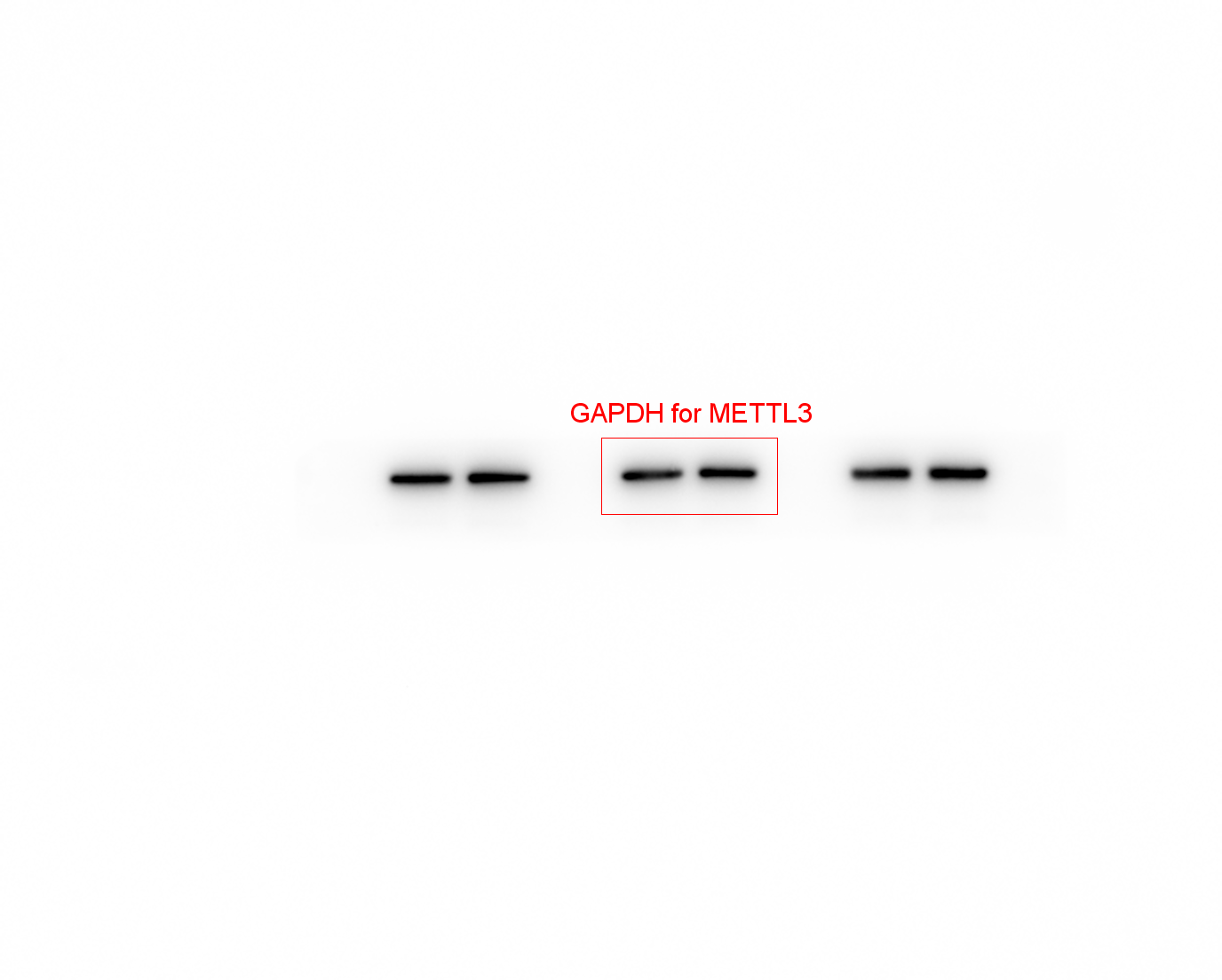

Supplement: Supplementary file 2 — Source Data Fig. 1 [file 44321_2024_25_MOESM2_ESM.zip › figure 1/1G/1G/1G GAPDH for METTL3 mark.Tif]

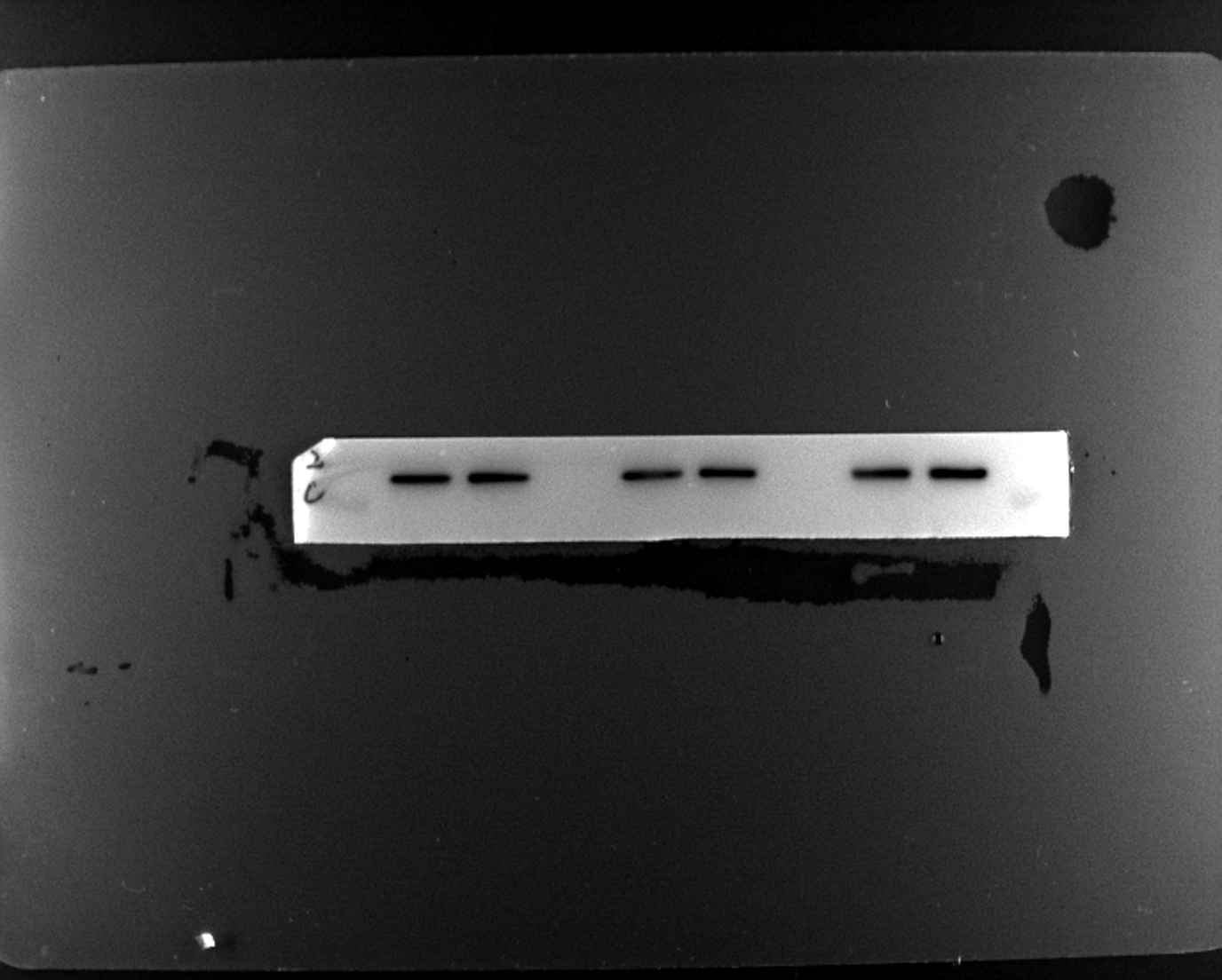

Supplement: Supplementary file 2 — Source Data Fig. 1 [file 44321_2024_25_MOESM2_ESM.zip › figure 1/1G/1G/1G GAPDH for METTL3.Tif]

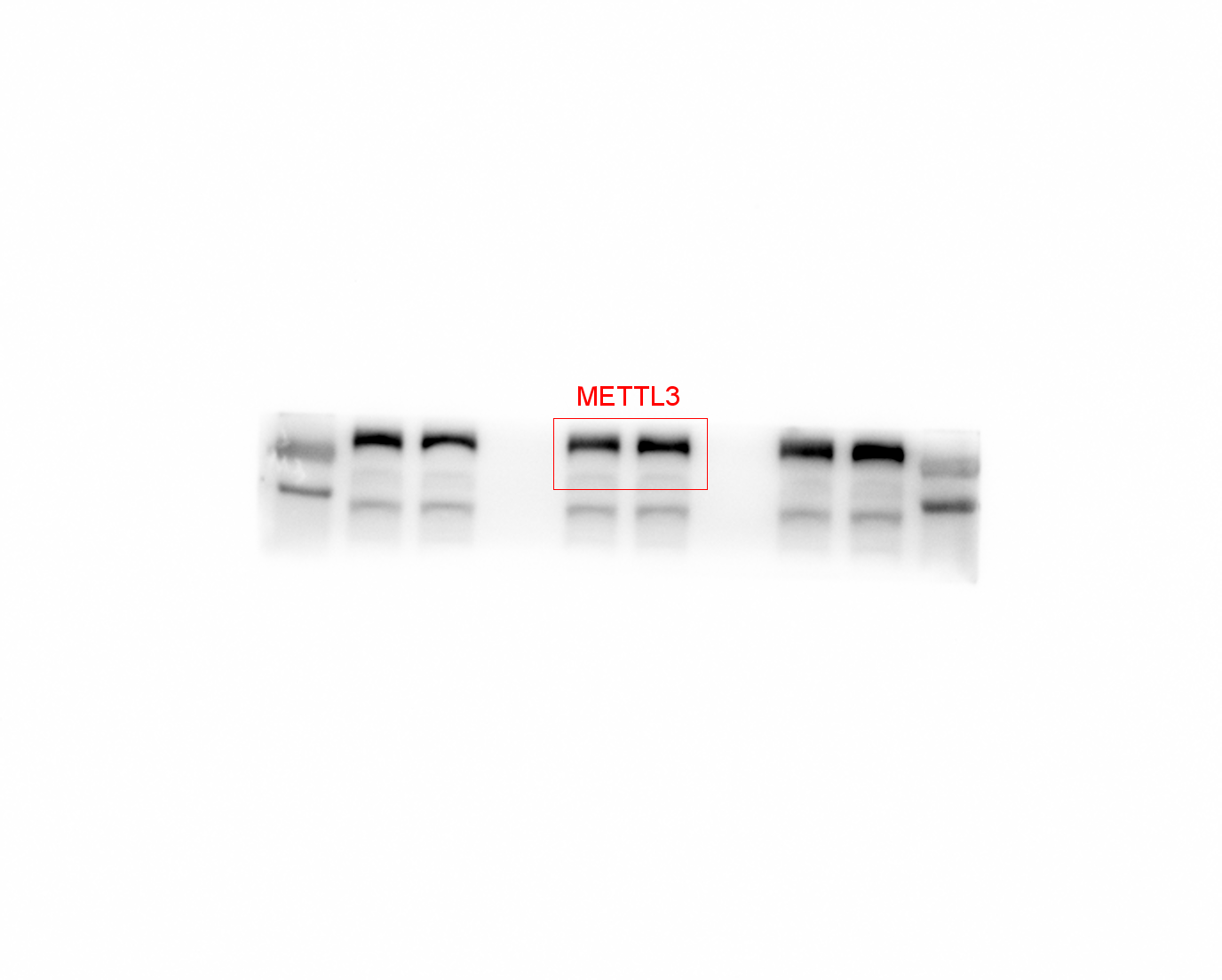

Supplement: Supplementary file 2 — Source Data Fig. 1 [file 44321_2024_25_MOESM2_ESM.zip › figure 1/1G/1G/1G METTL3 mark.Tif]

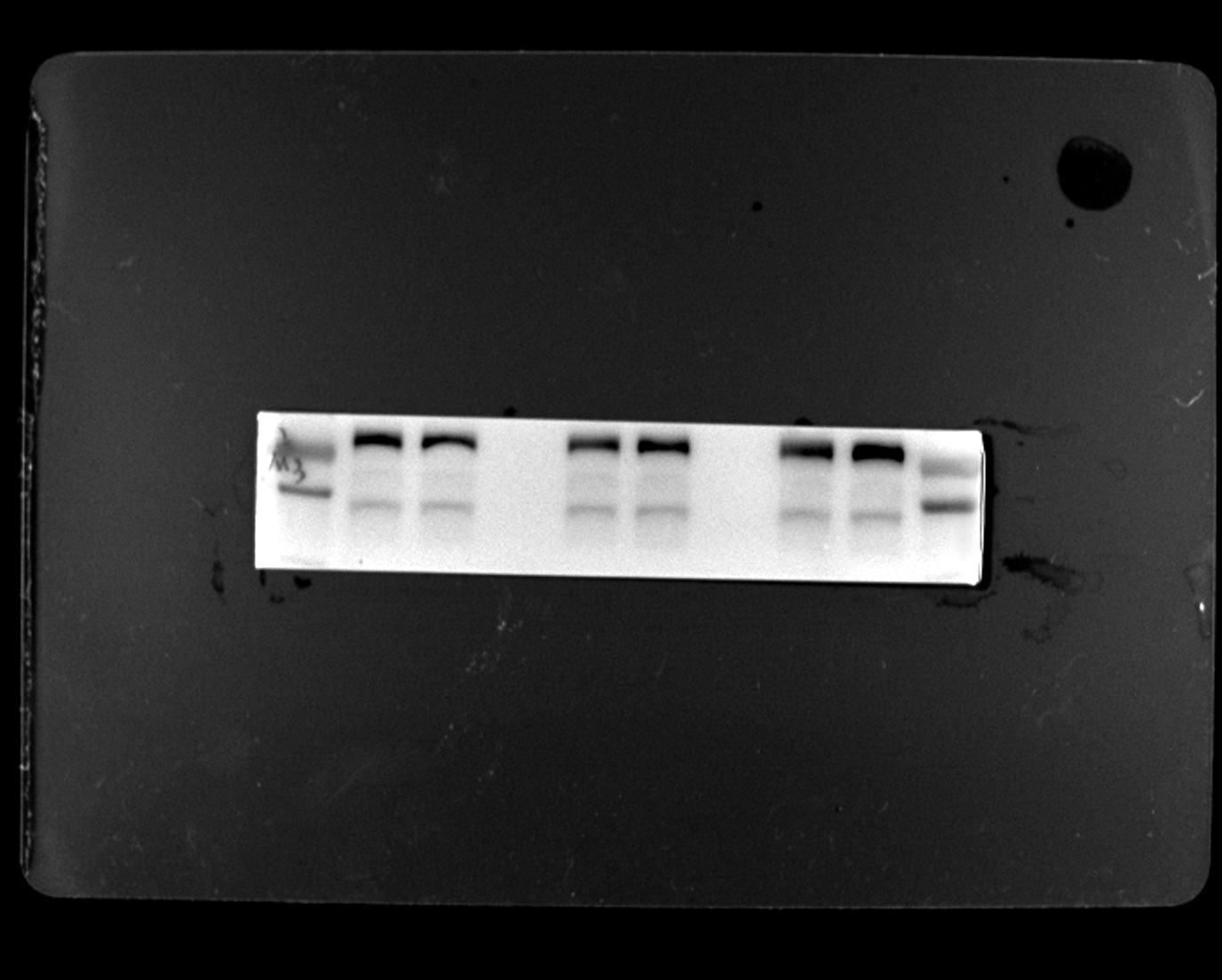

Supplement: Supplementary file 2 — Source Data Fig. 1 [file 44321_2024_25_MOESM2_ESM.zip › figure 1/1G/1G/1G METTL3.Tif]

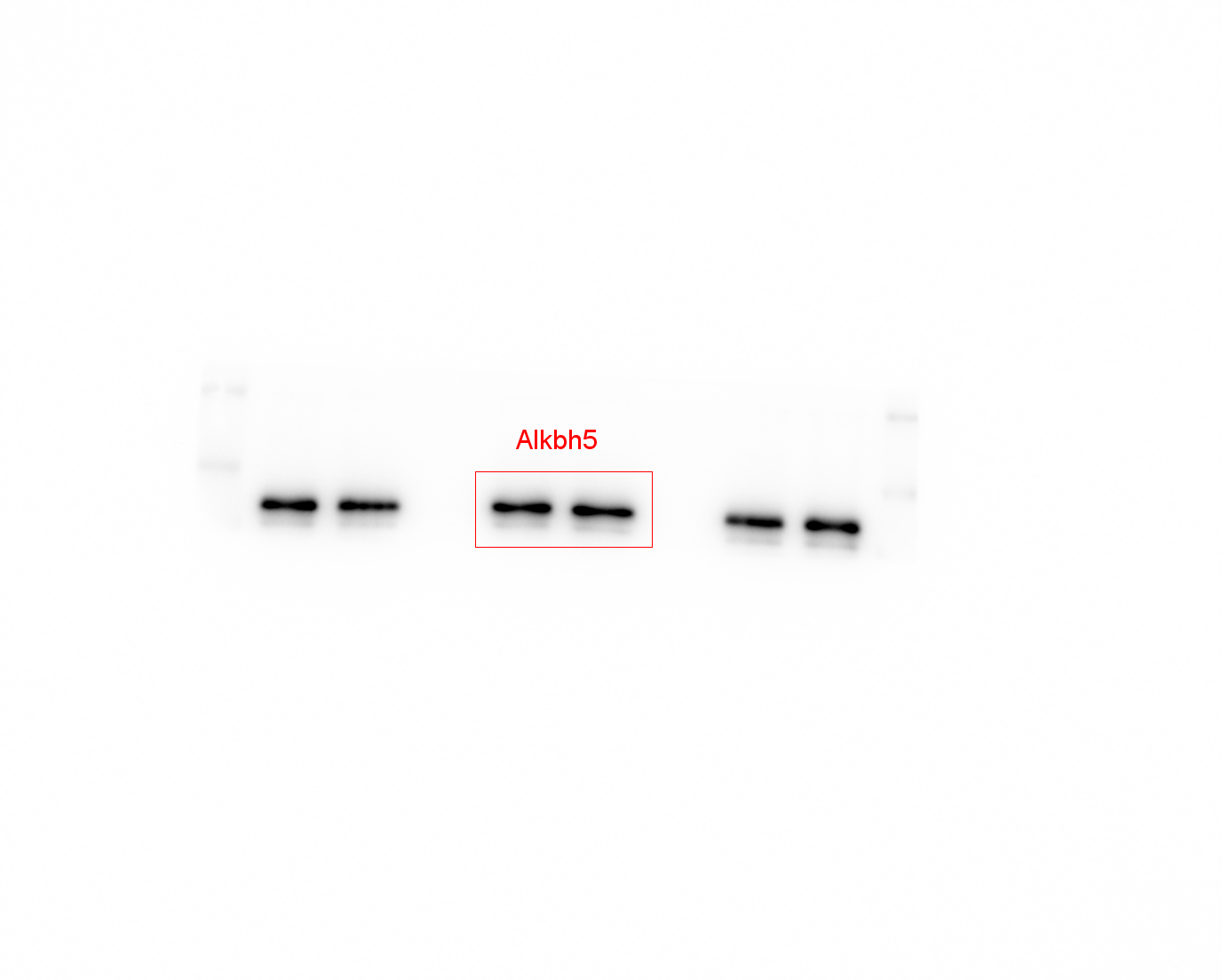

Supplement: Supplementary file 2 — Source Data Fig. 1 [file 44321_2024_25_MOESM2_ESM.zip › figure 1/1H/1H/1H Alkbh5 mark.Tif]

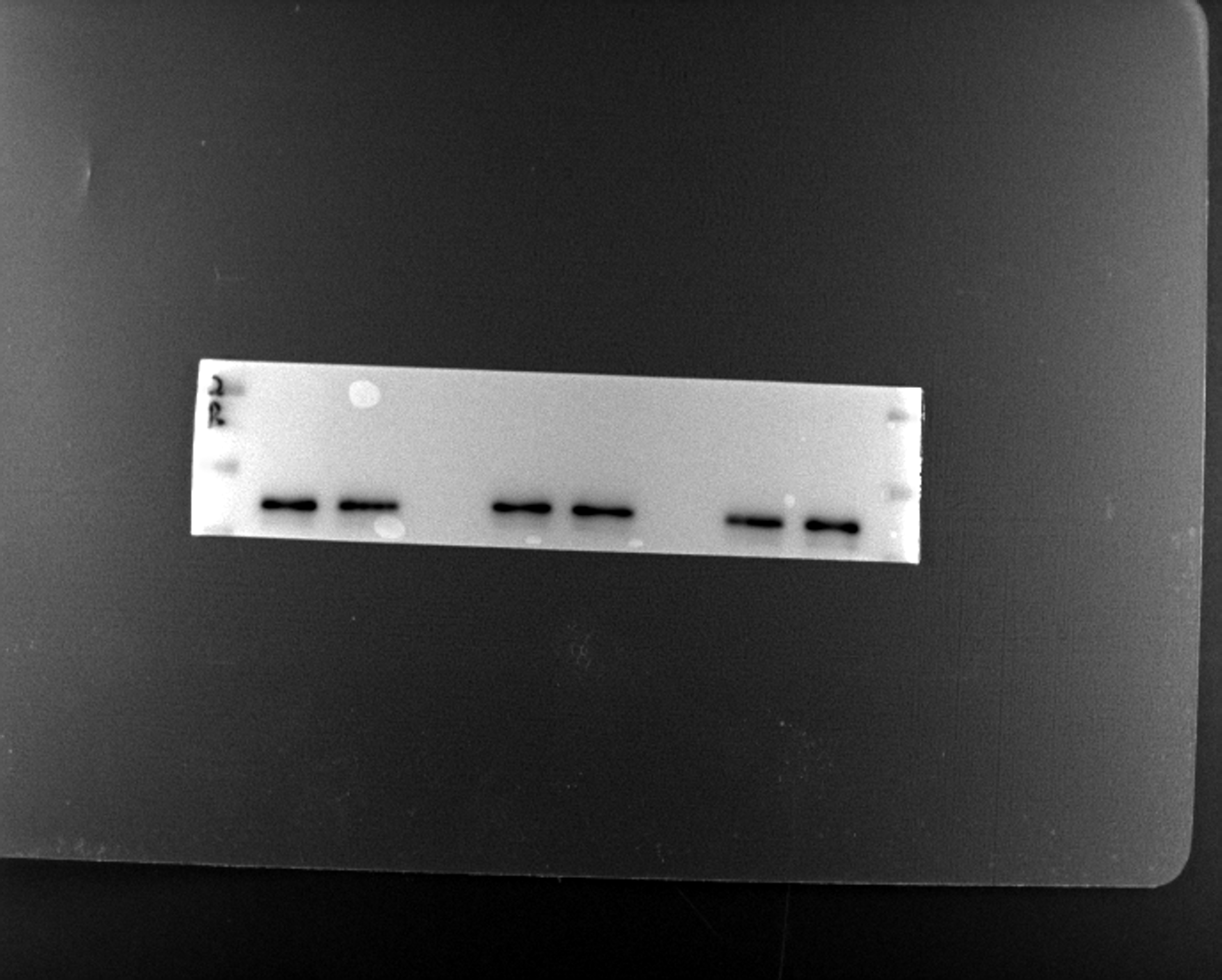

Supplement: Supplementary file 2 — Source Data Fig. 1 [file 44321_2024_25_MOESM2_ESM.zip › figure 1/1H/1H/1H Alkbh5.Tif]

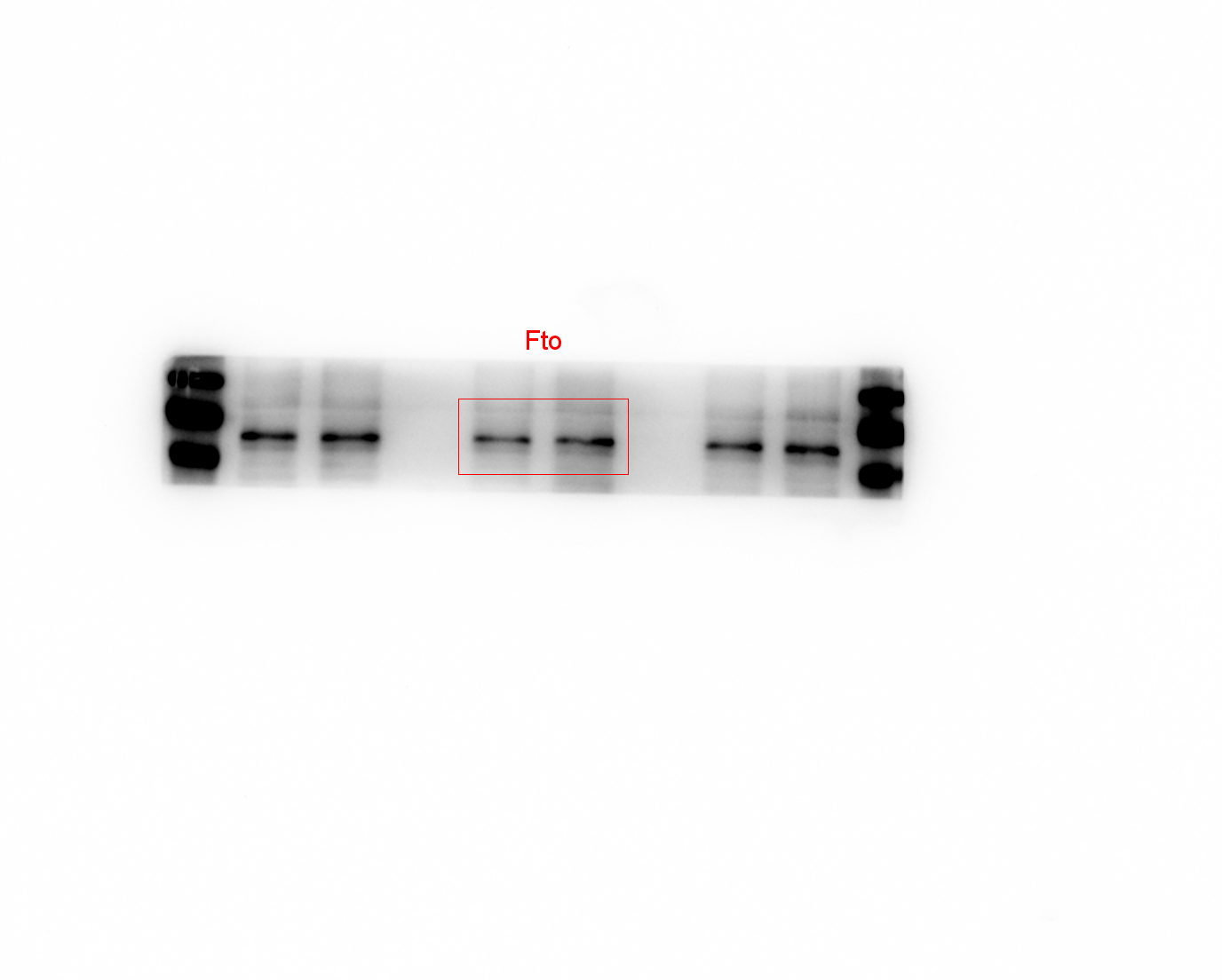

Supplement: Supplementary file 2 — Source Data Fig. 1 [file 44321_2024_25_MOESM2_ESM.zip › figure 1/1H/1H/1H Fto mark.Tif]

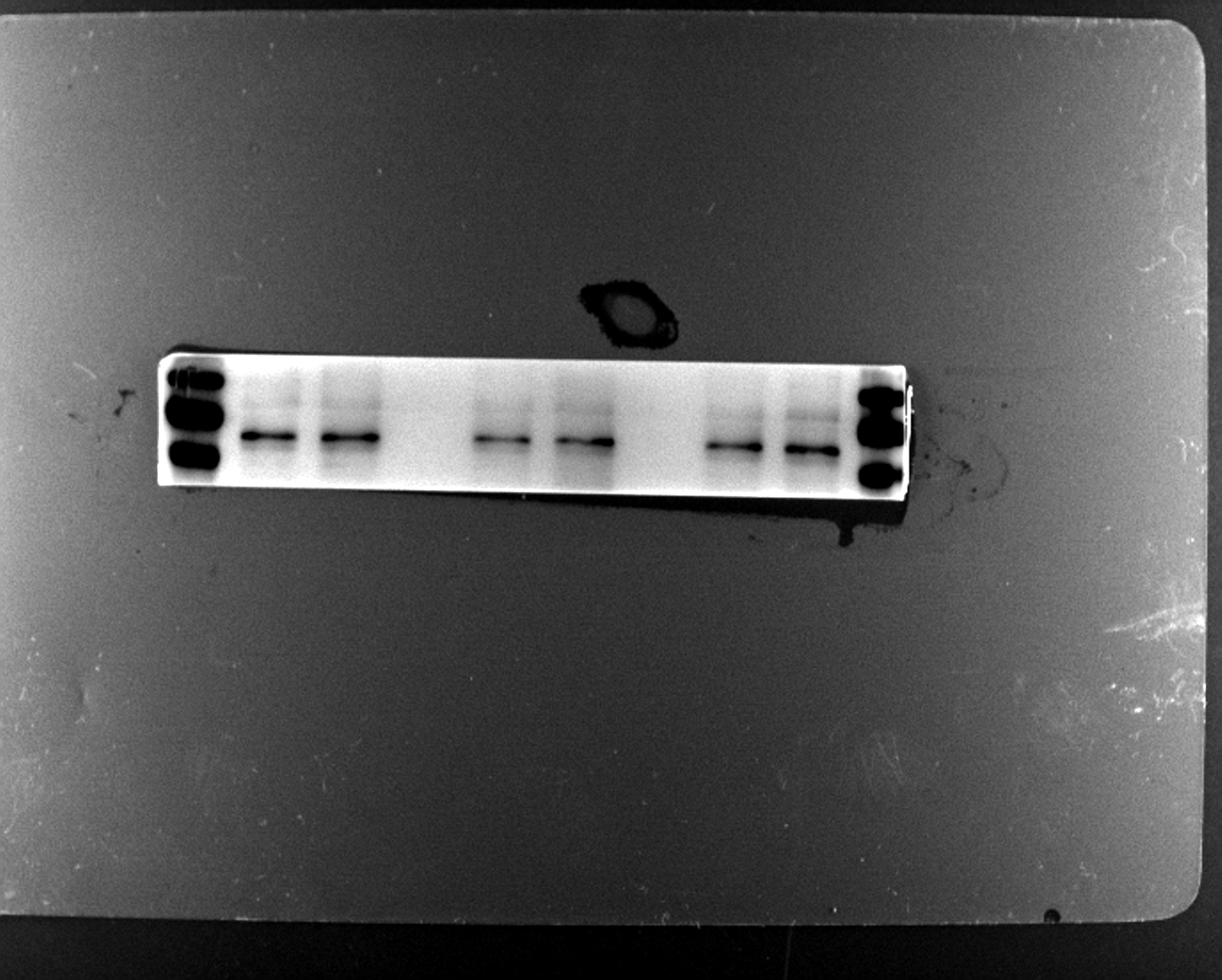

Supplement: Supplementary file 2 — Source Data Fig. 1 [file 44321_2024_25_MOESM2_ESM.zip › figure 1/1H/1H/1H Fto.Tif]

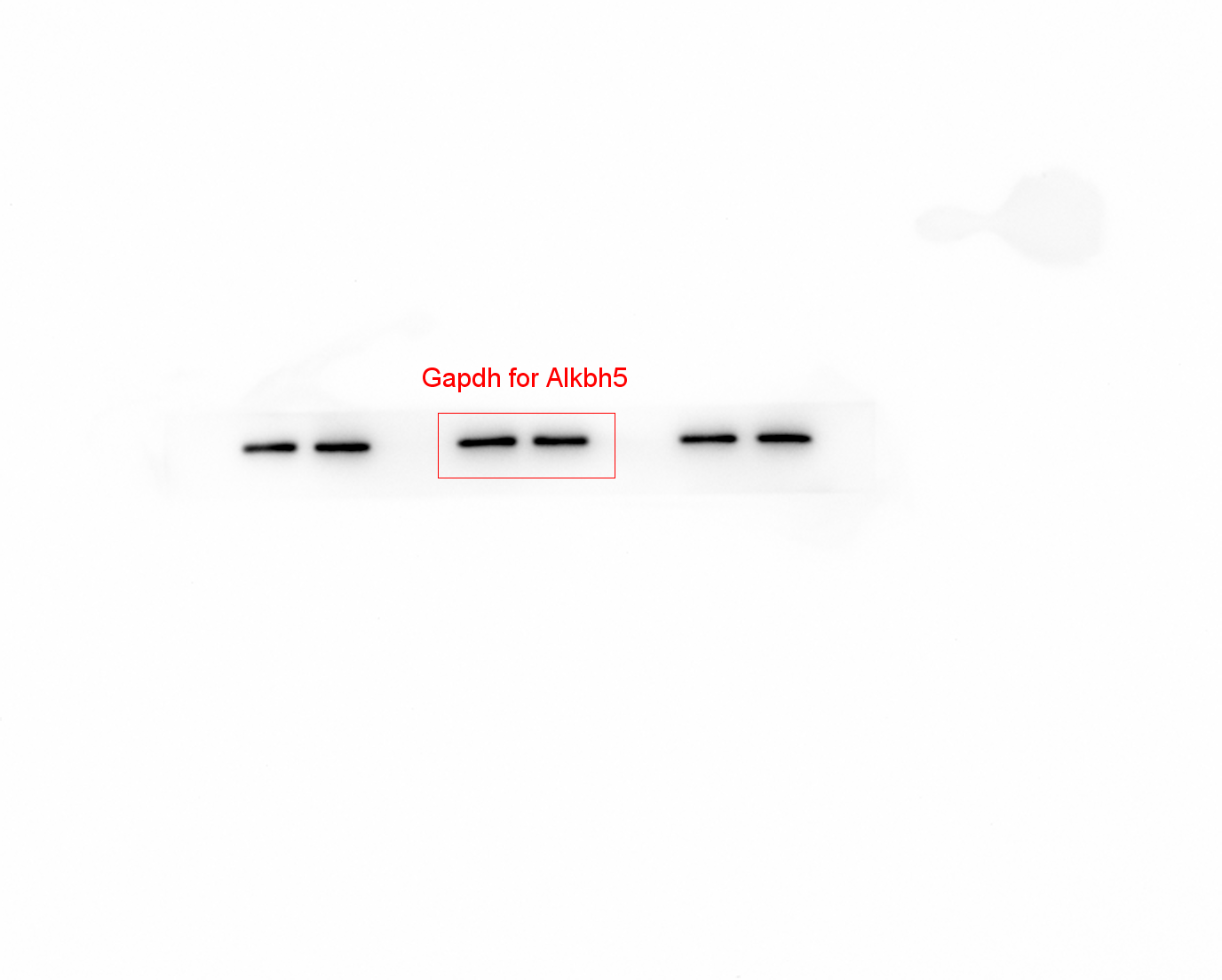

Supplement: Supplementary file 2 — Source Data Fig. 1 [file 44321_2024_25_MOESM2_ESM.zip › figure 1/1H/1H/1H Gapdh for Alkbh5 mark.Tif]

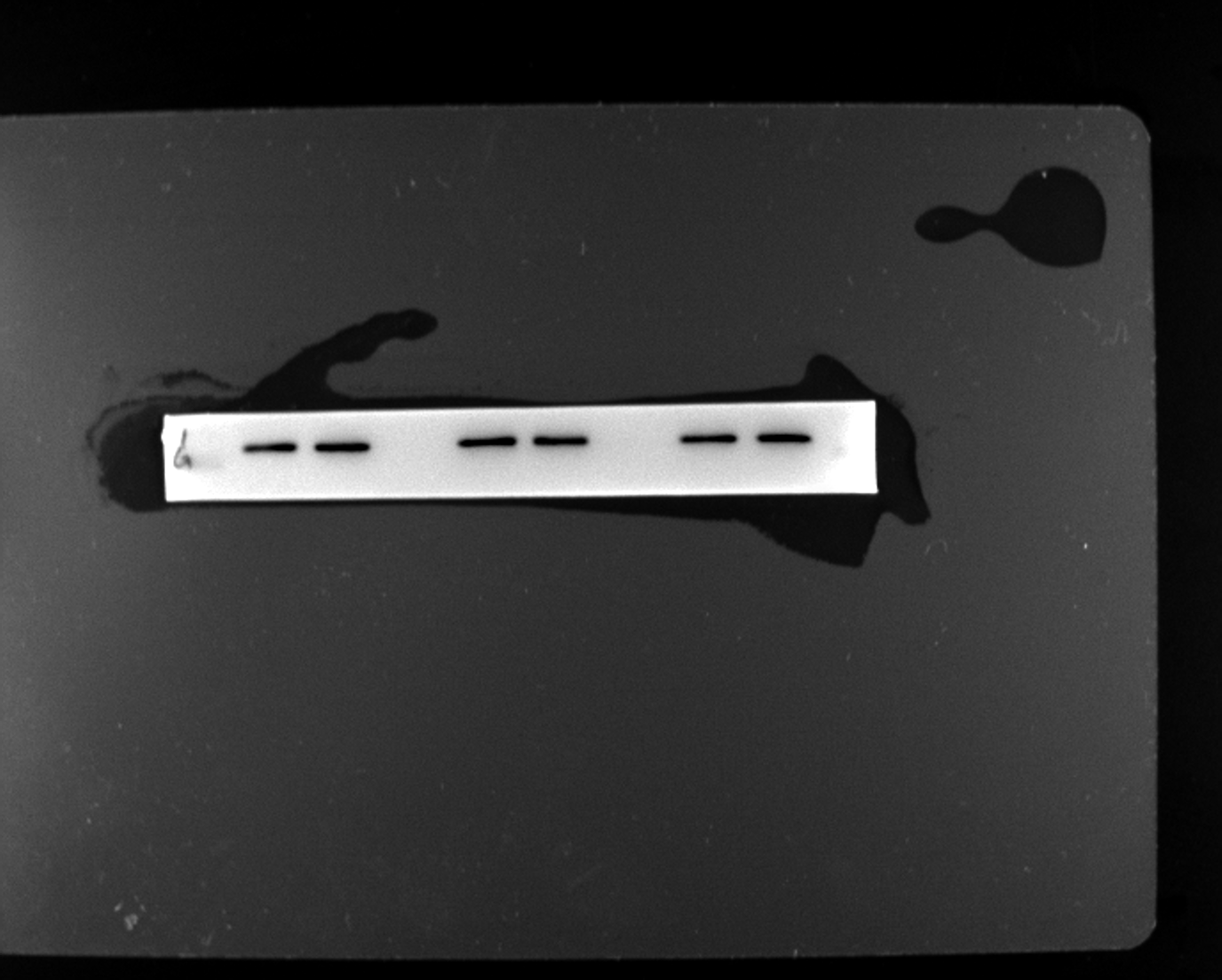

Supplement: Supplementary file 2 — Source Data Fig. 1 [file 44321_2024_25_MOESM2_ESM.zip › figure 1/1H/1H/1H Gapdh for Alkbh5.Tif]

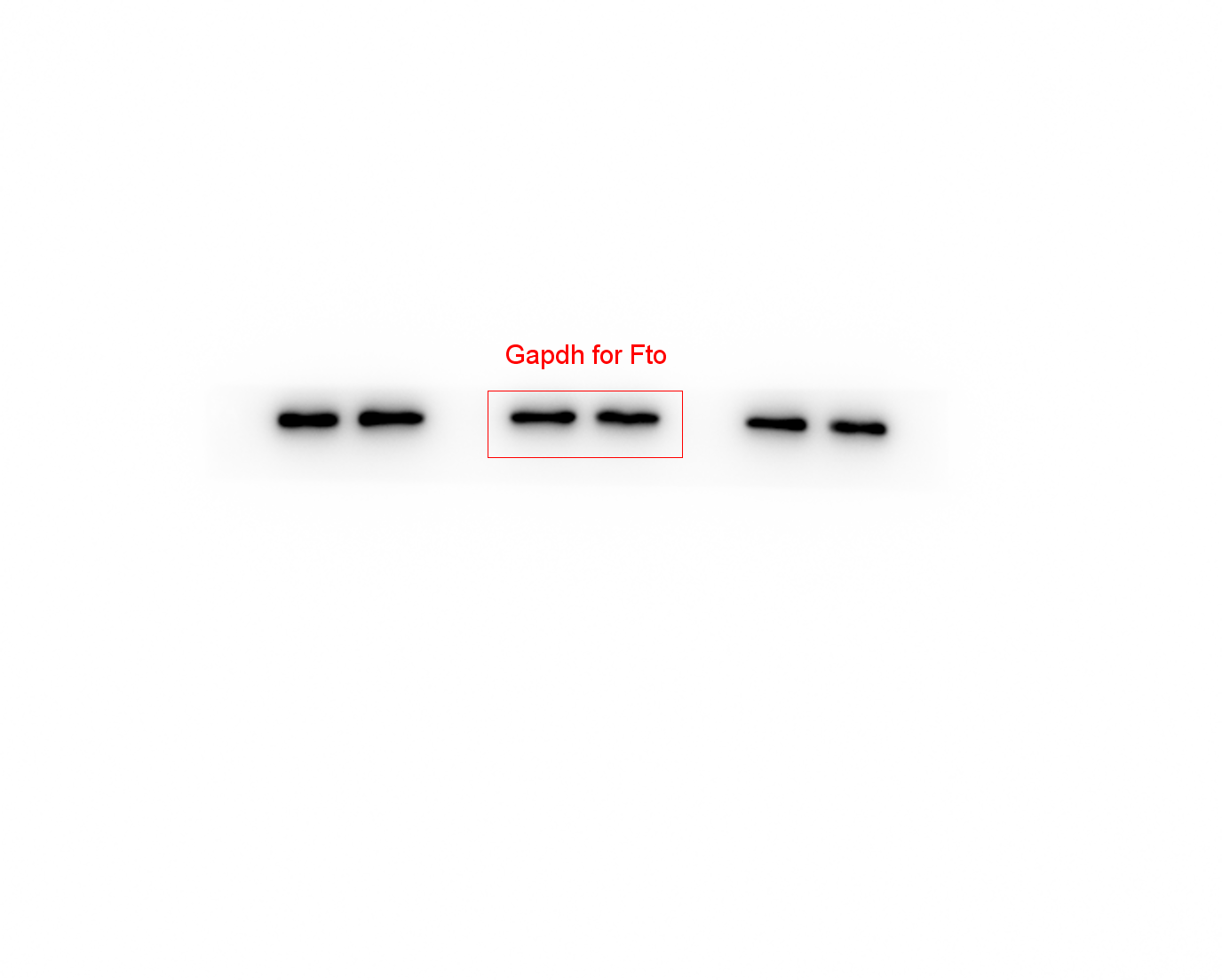

Supplement: Supplementary file 2 — Source Data Fig. 1 [file 44321_2024_25_MOESM2_ESM.zip › figure 1/1H/1H/1H Gapdh for Fto mark.Tif]

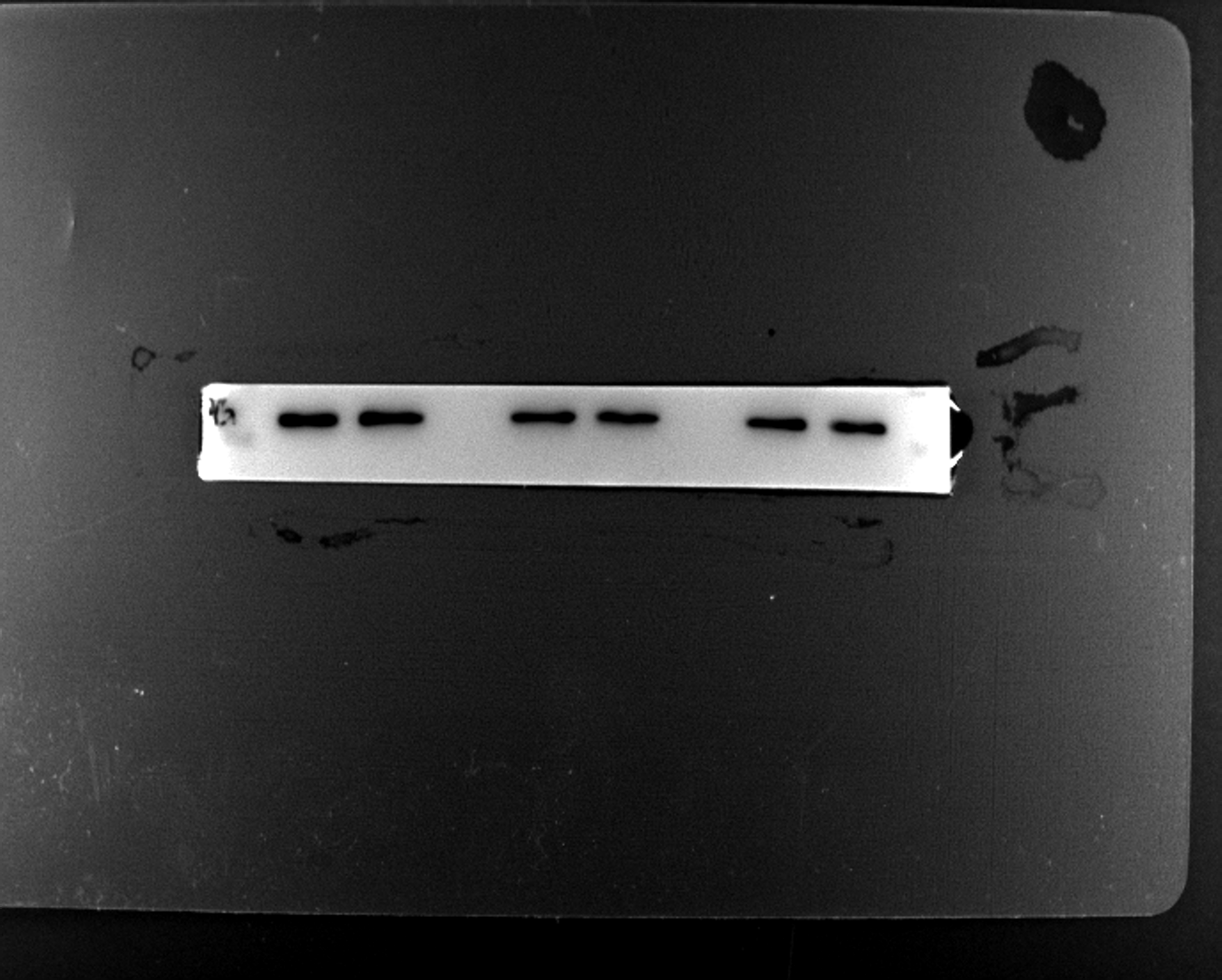

Supplement: Supplementary file 2 — Source Data Fig. 1 [file 44321_2024_25_MOESM2_ESM.zip › figure 1/1H/1H/1H Gapdh for Fto.Tif]

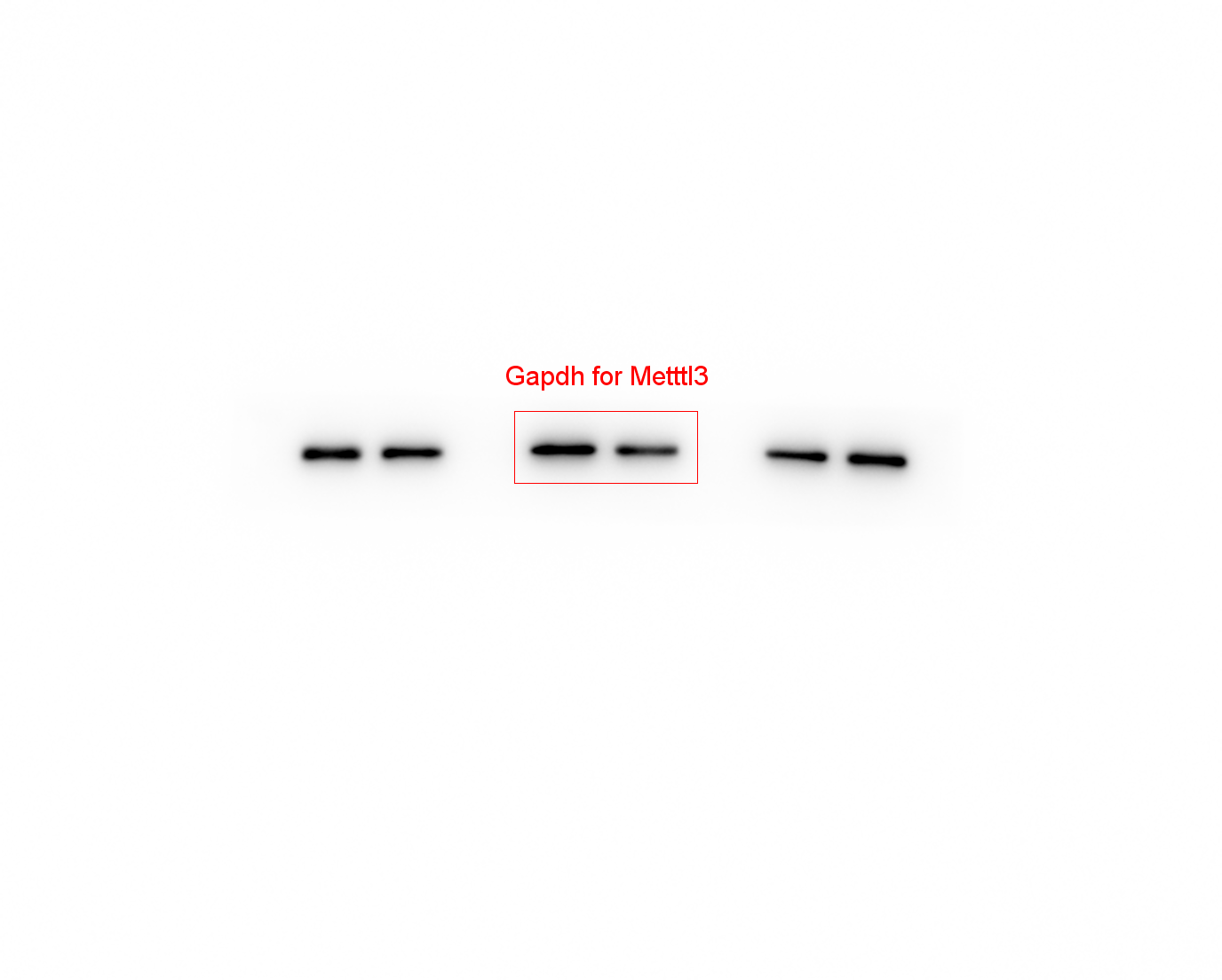

Supplement: Supplementary file 2 — Source Data Fig. 1 [file 44321_2024_25_MOESM2_ESM.zip › figure 1/1H/1H/1H Gapdh for Mettl3 mark.Tif]

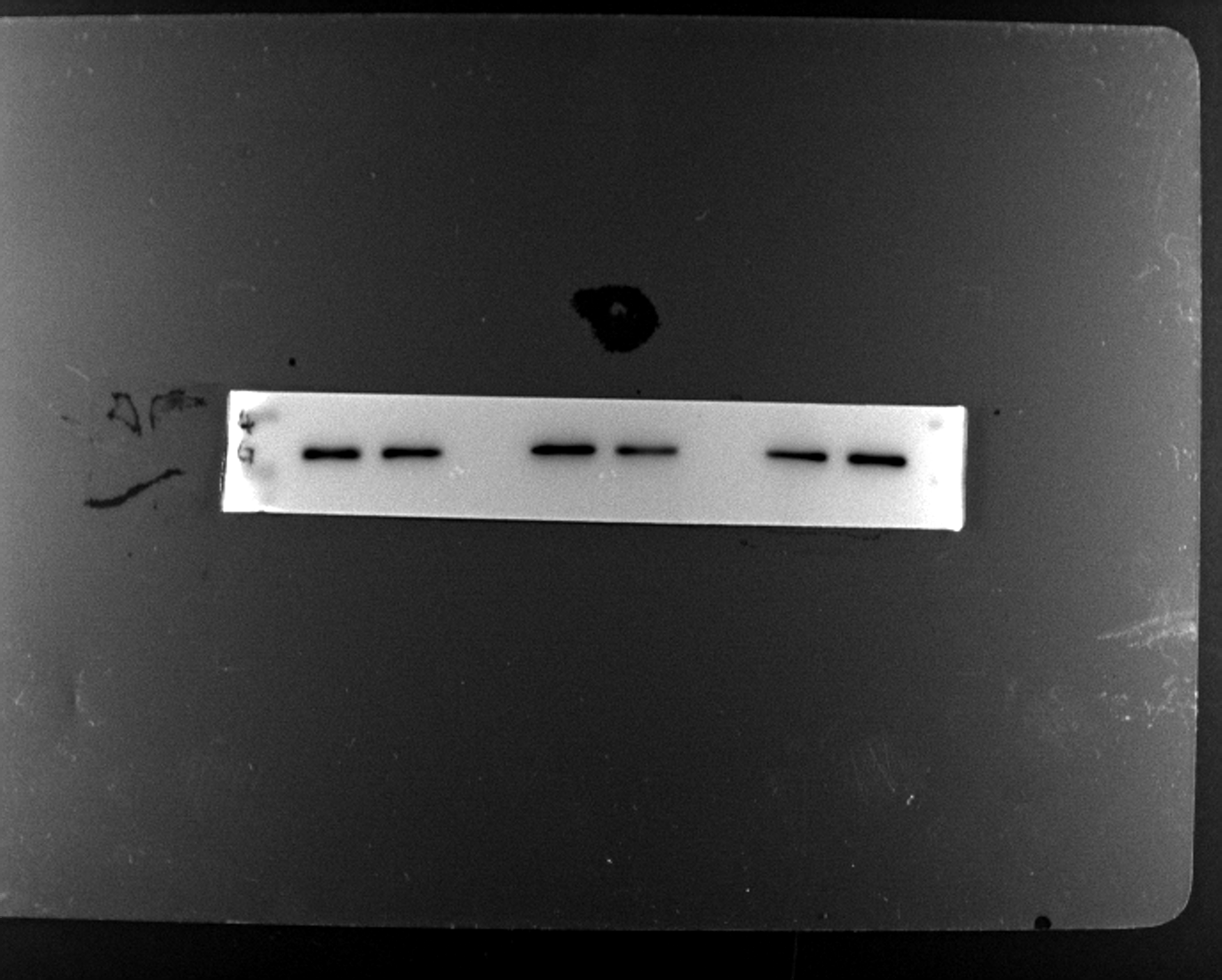

Supplement: Supplementary file 2 — Source Data Fig. 1 [file 44321_2024_25_MOESM2_ESM.zip › figure 1/1H/1H/1H Gapdh for Mettl3.Tif]

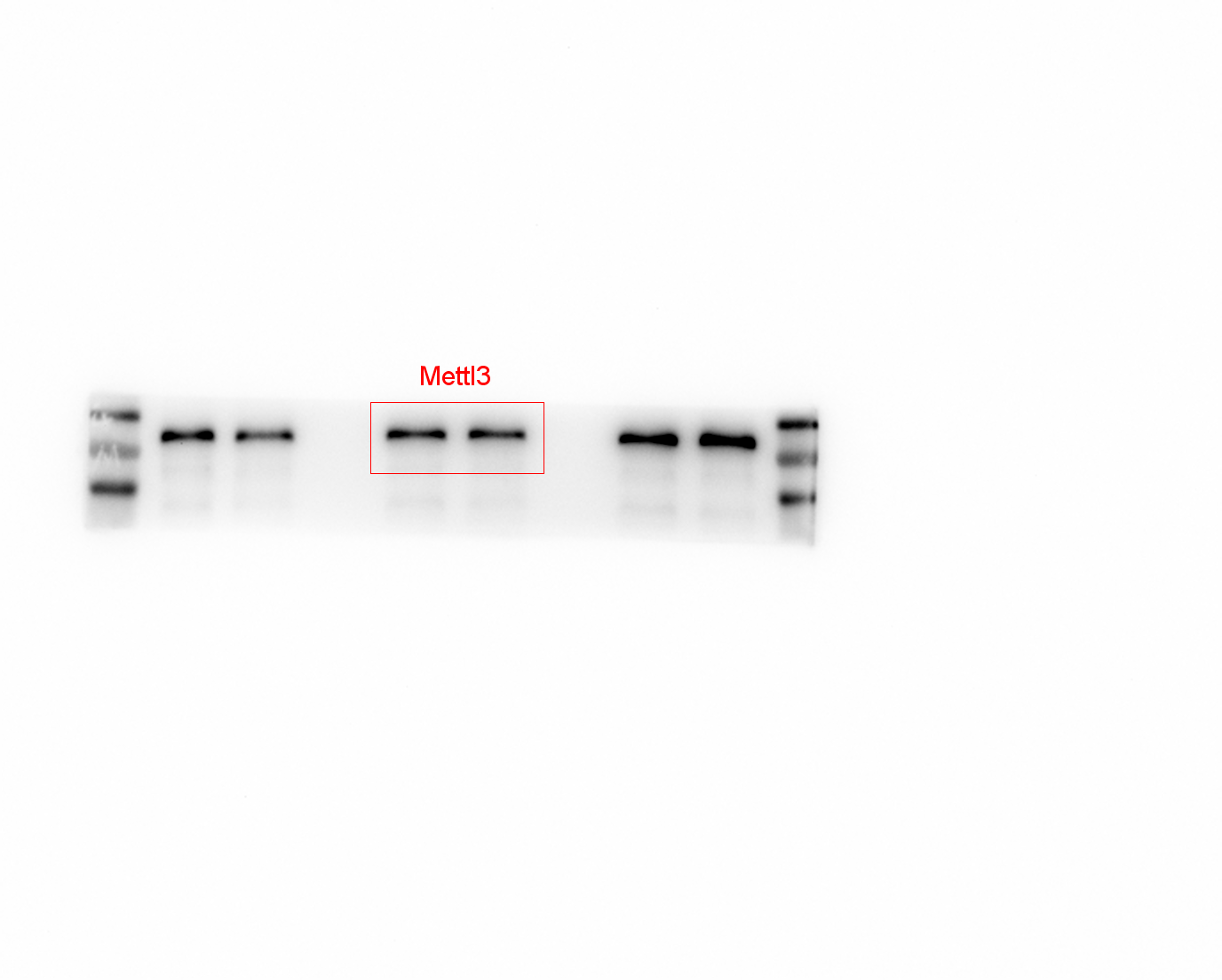

Supplement: Supplementary file 2 — Source Data Fig. 1 [file 44321_2024_25_MOESM2_ESM.zip › figure 1/1H/1H/1H Mettl3 mark.Tif]

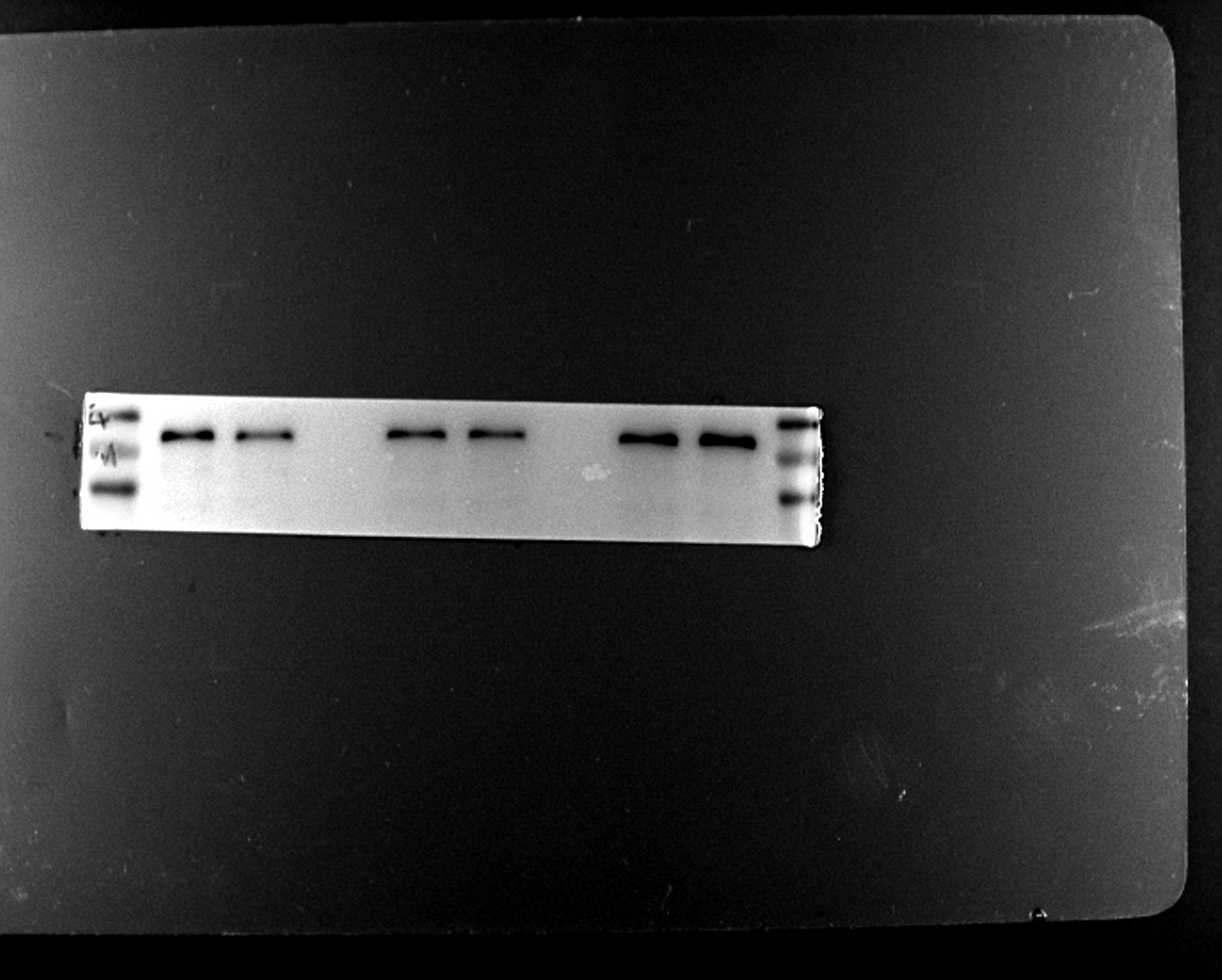

Supplement: Supplementary file 2 — Source Data Fig. 1 [file 44321_2024_25_MOESM2_ESM.zip › figure 1/1H/1H/1H Mettl3.Tif]

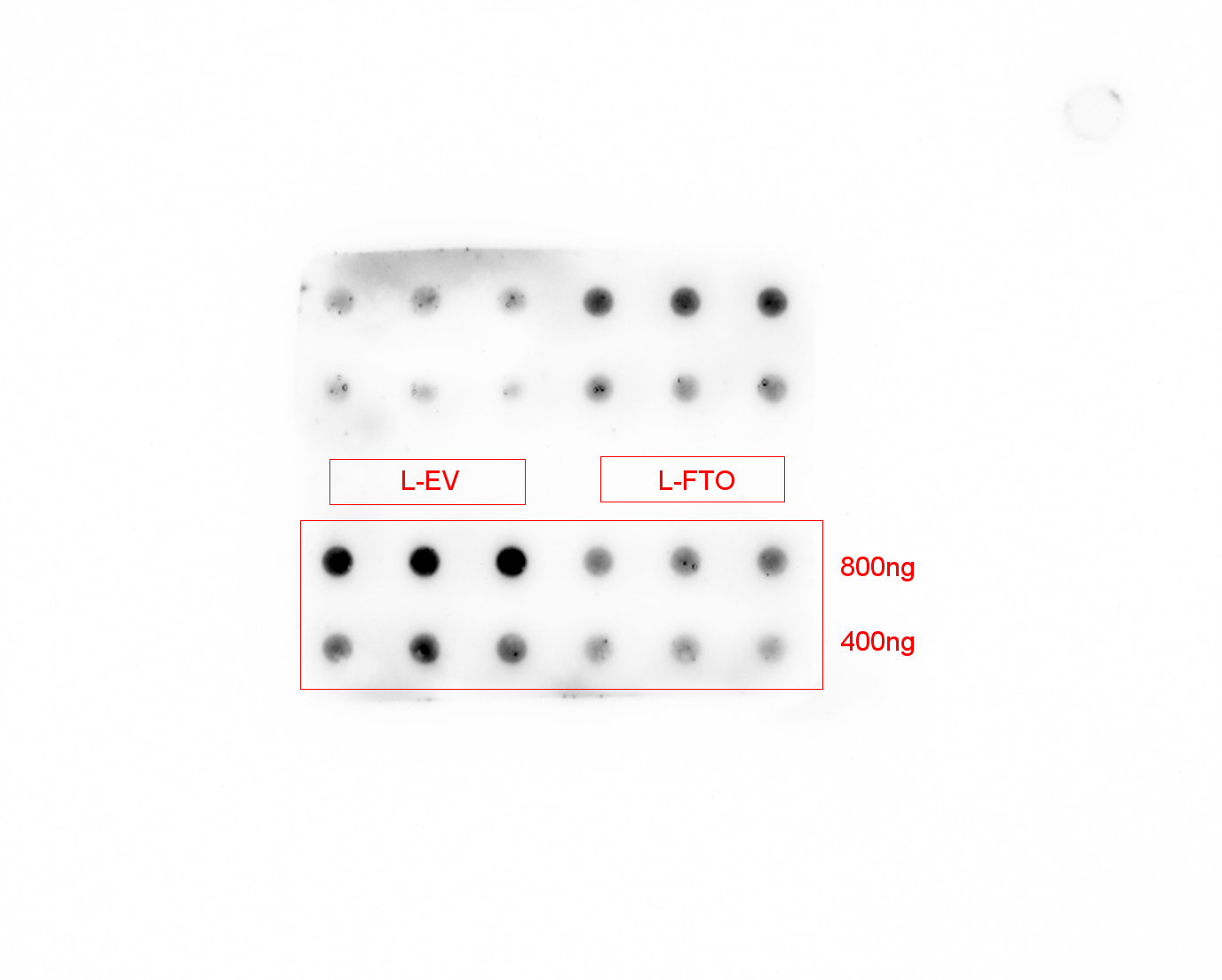

Supplement: Supplementary file 2 — Source Data Fig. 1 [file 44321_2024_25_MOESM2_ESM.zip › figure 1/1I/1I/1I m6A mark.Tif]

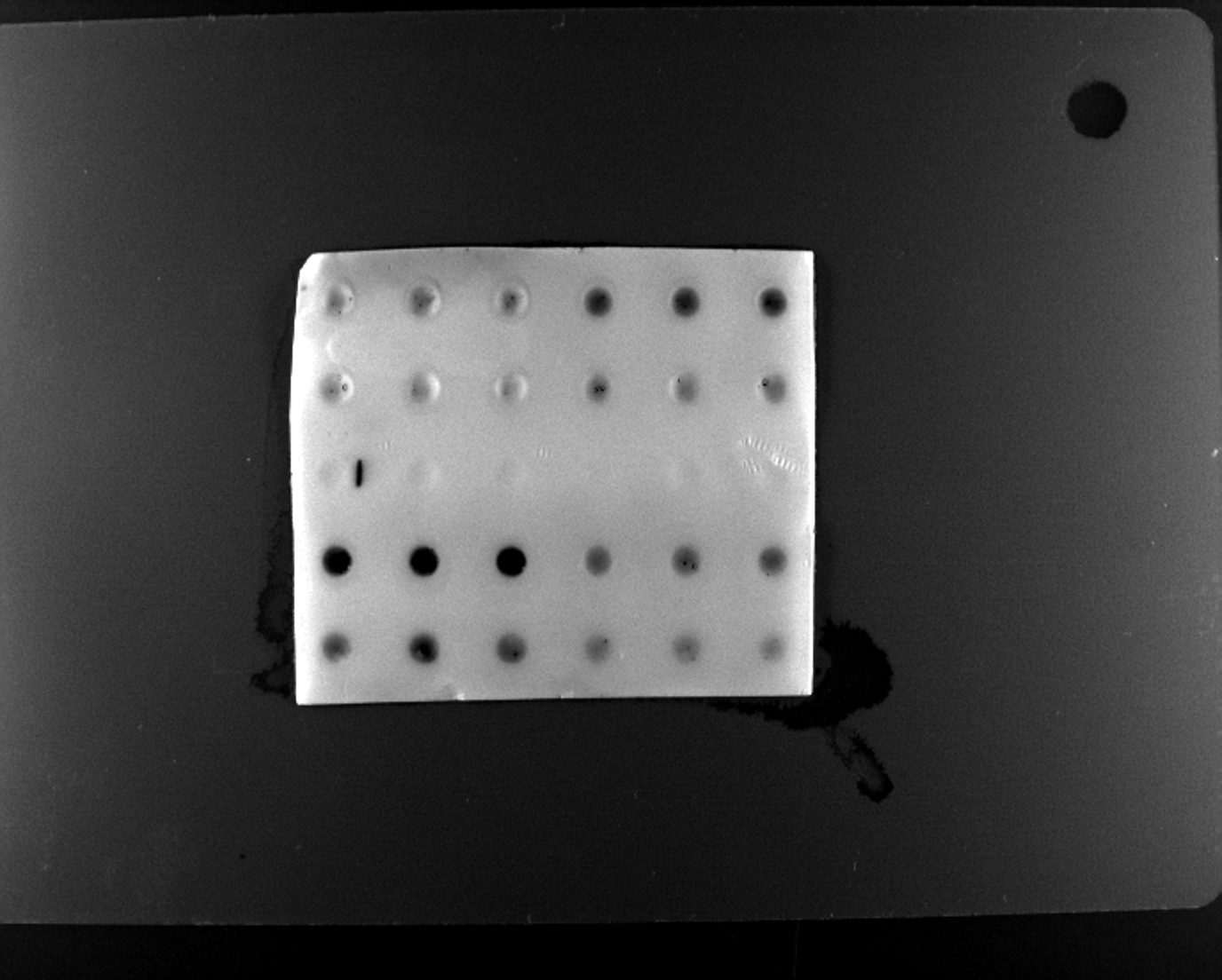

Supplement: Supplementary file 2 — Source Data Fig. 1 [file 44321_2024_25_MOESM2_ESM.zip › figure 1/1I/1I/1I m6A.Tif]

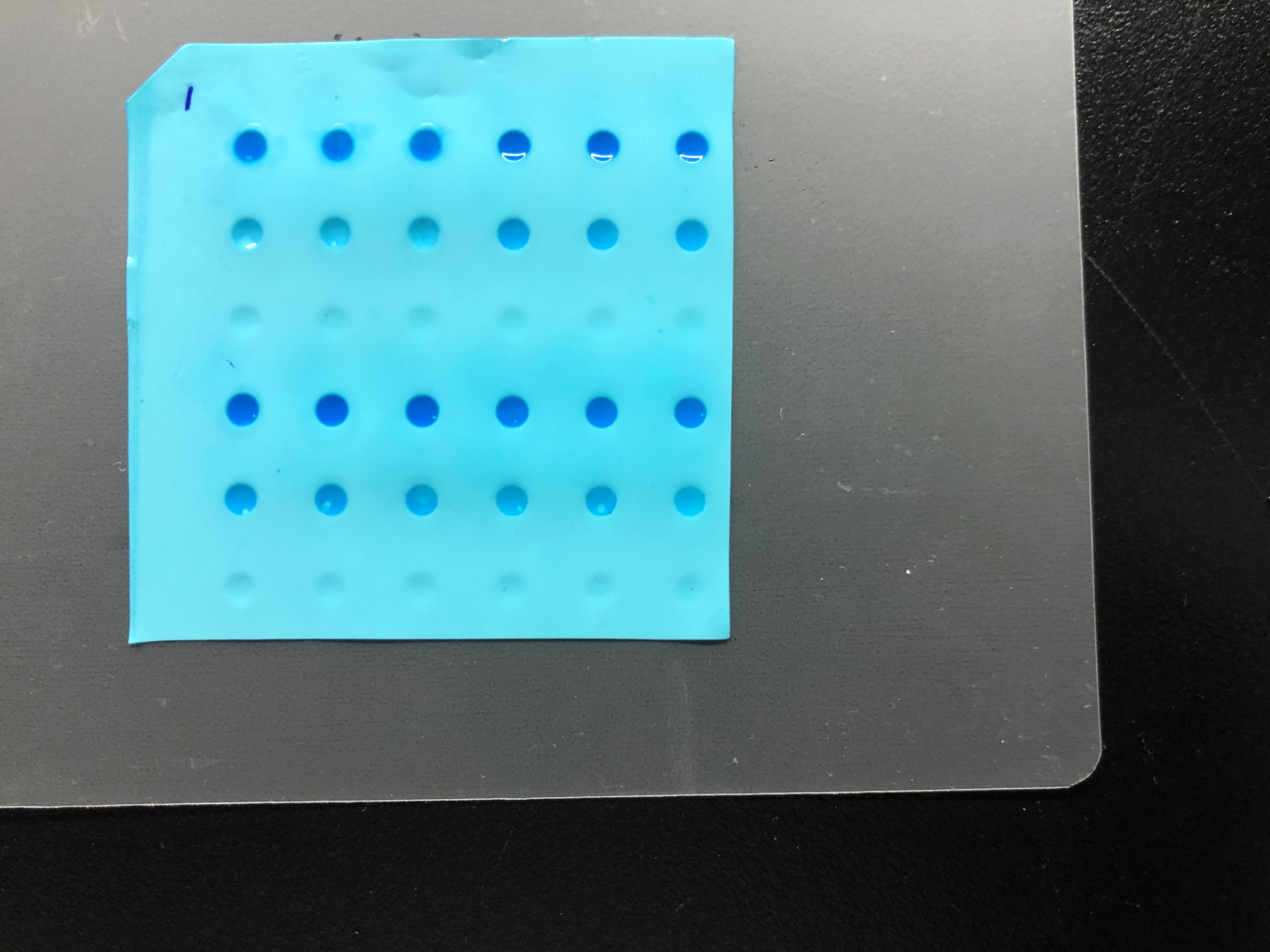

Supplement: Supplementary file 2 — Source Data Fig. 1 [file 44321_2024_25_MOESM2_ESM.zip › figure 1/1I/1I/1I MB.tif]

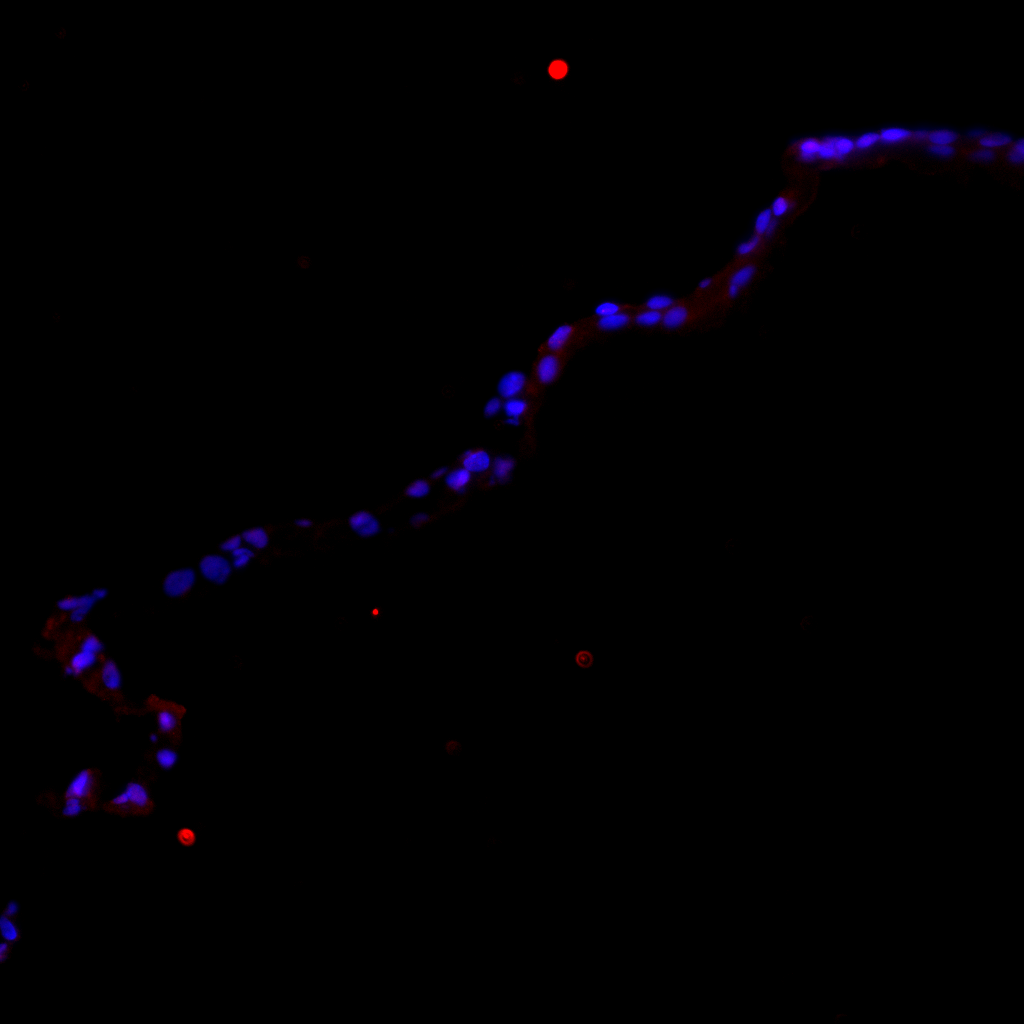

Supplement: Supplementary file 2 — Source Data Fig. 1 [file 44321_2024_25_MOESM2_ESM.zip › figure 1/1J/1J/1J ERM FTO DAPI.tif]

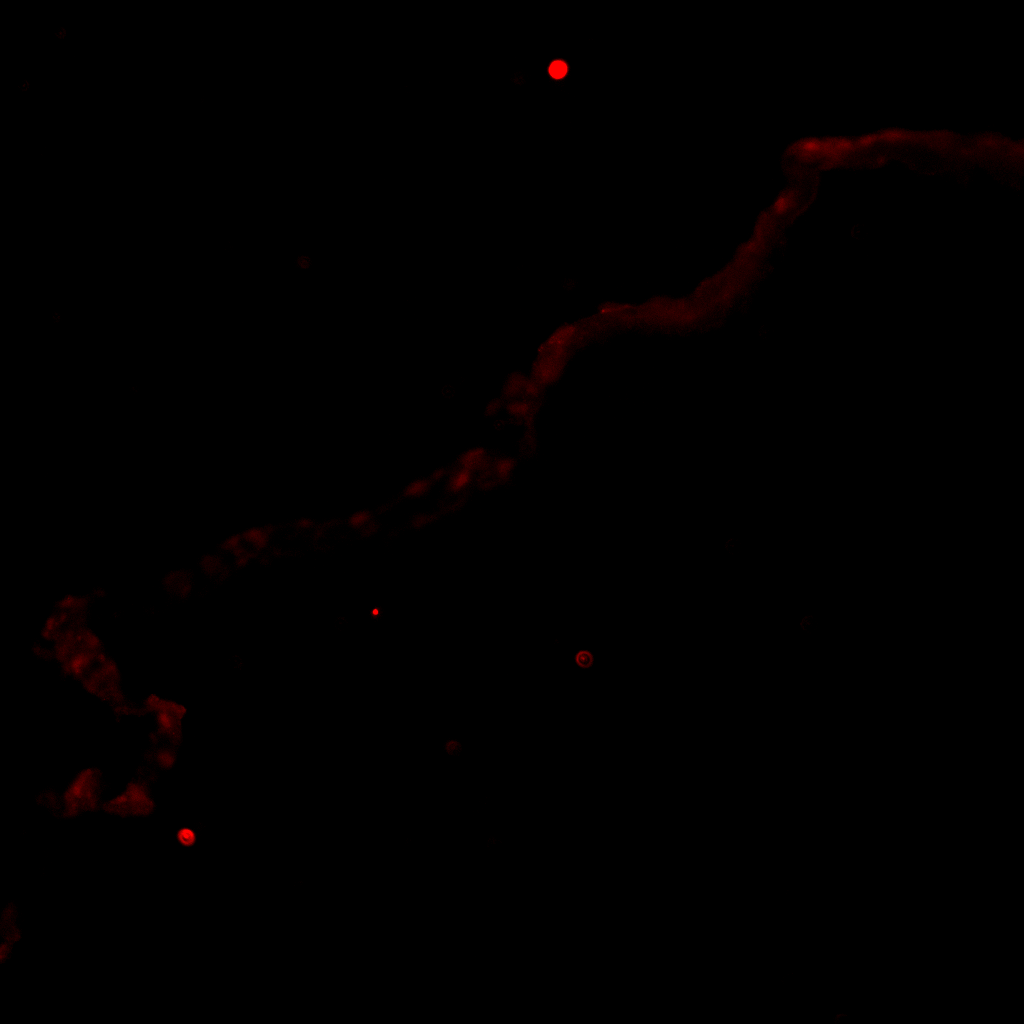

Supplement: Supplementary file 2 — Source Data Fig. 1 [file 44321_2024_25_MOESM2_ESM.zip › figure 1/1J/1J/1J ERM FTO.tif]

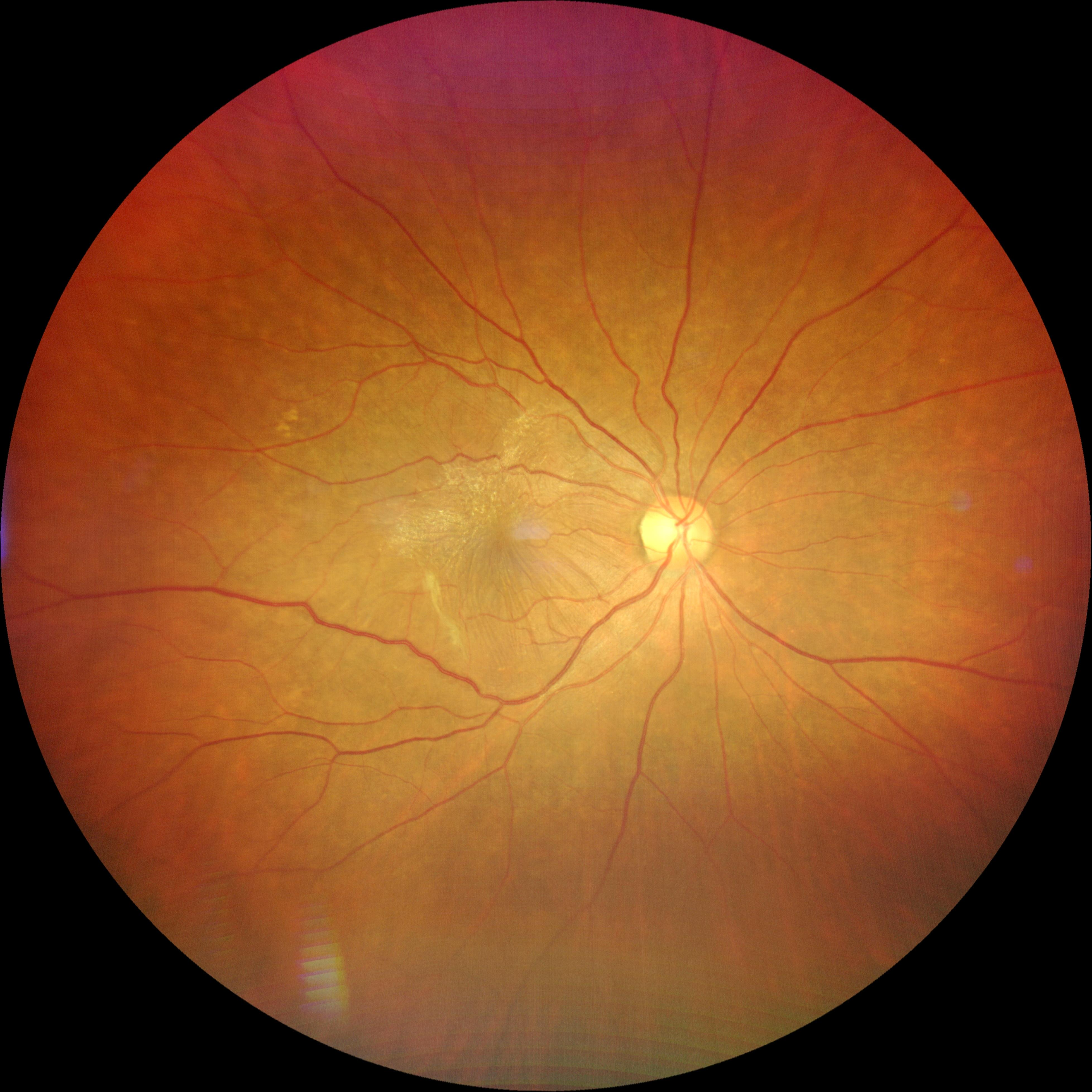

Supplement: Supplementary file 2 — Source Data Fig. 1 [file 44321_2024_25_MOESM2_ESM.zip › figure 1/1J/1J/1J ERM Fundus.tif]

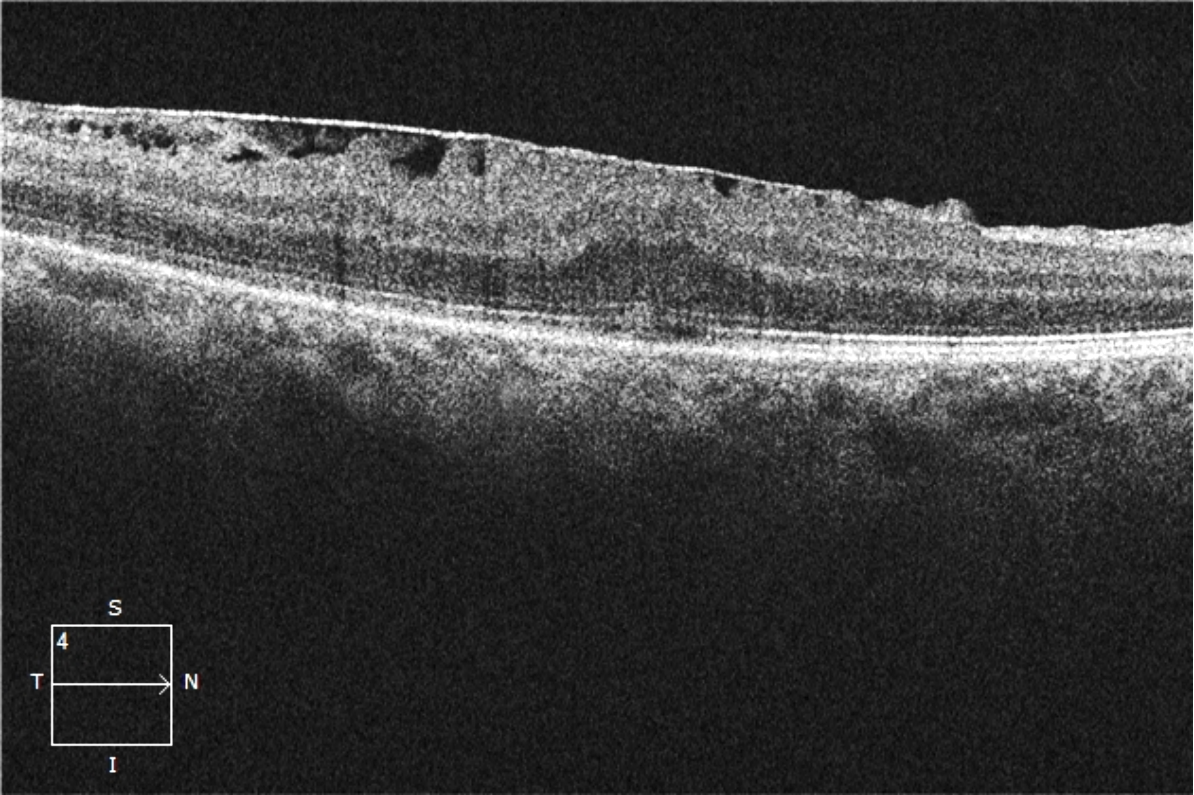

Supplement: Supplementary file 2 — Source Data Fig. 1 [file 44321_2024_25_MOESM2_ESM.zip › figure 1/1J/1J/1J ERM OCT.tif]

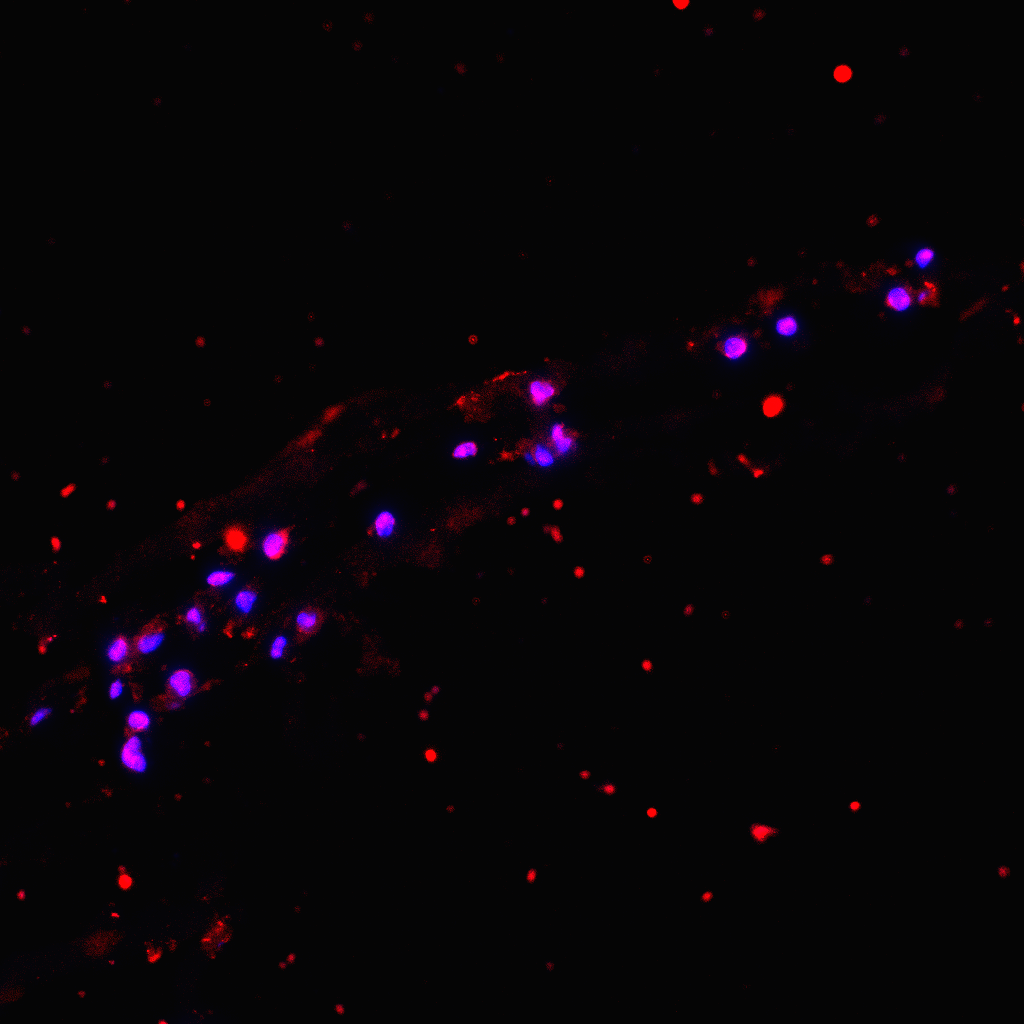

Supplement: Supplementary file 2 — Source Data Fig. 1 [file 44321_2024_25_MOESM2_ESM.zip › figure 1/1J/1J/1J PDR FTO DAPI.tif]

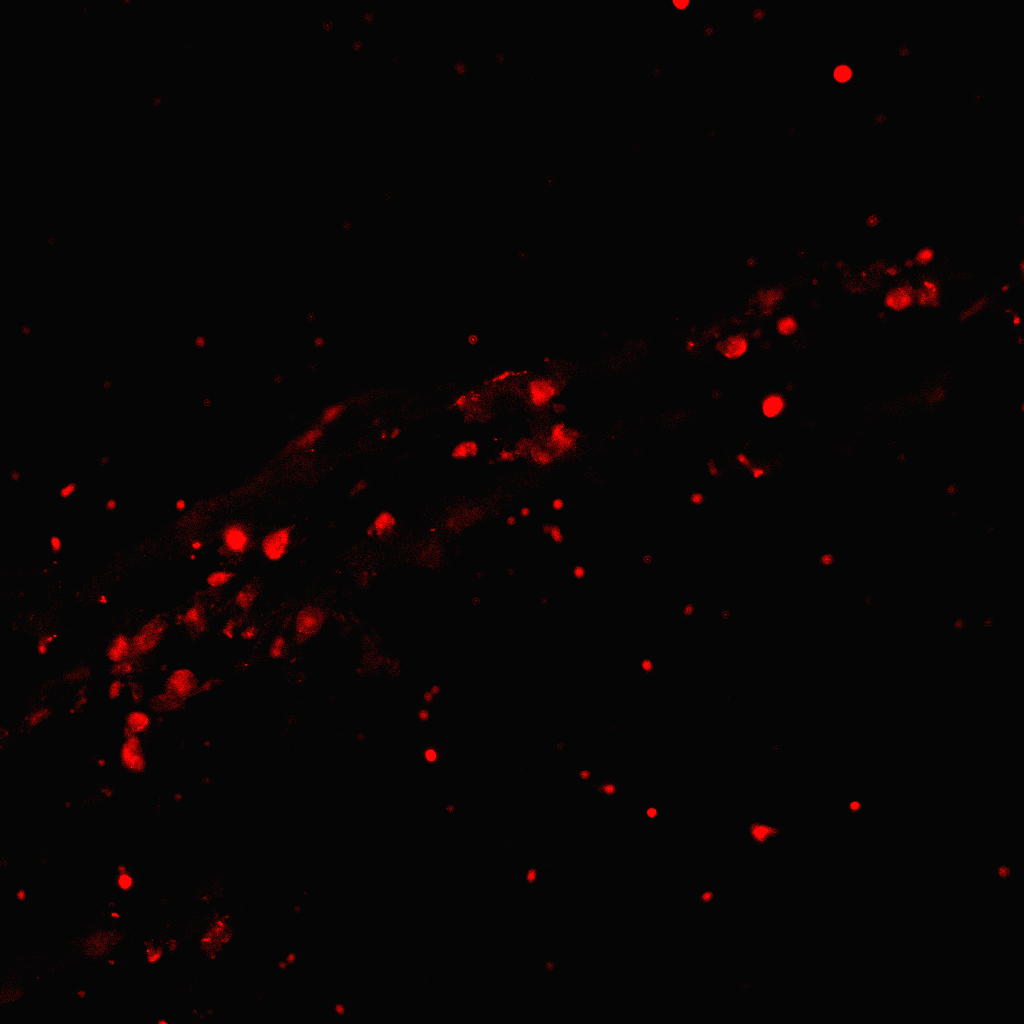

Supplement: Supplementary file 2 — Source Data Fig. 1 [file 44321_2024_25_MOESM2_ESM.zip › figure 1/1J/1J/1J PDR FTO.tif]

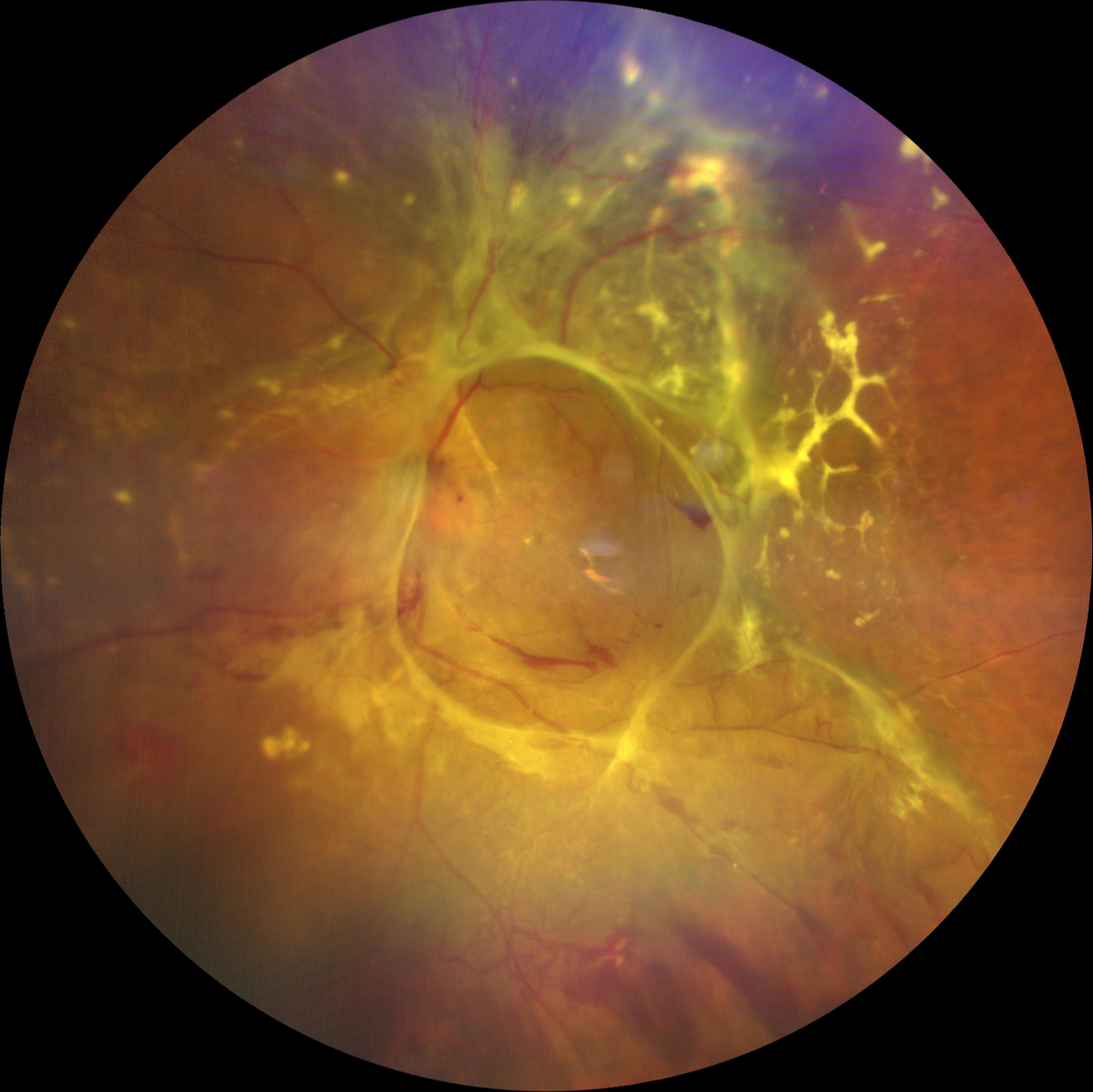

Supplement: Supplementary file 2 — Source Data Fig. 1 [file 44321_2024_25_MOESM2_ESM.zip › figure 1/1J/1J/1J PDR Fundus.tif]

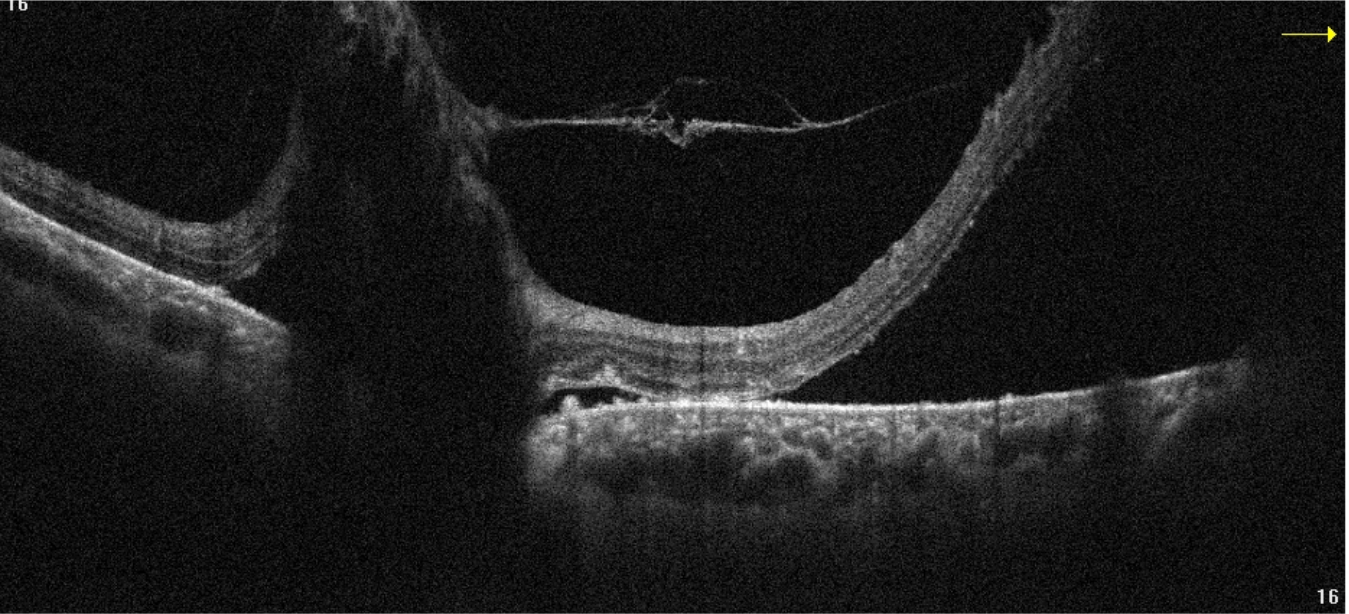

Supplement: Supplementary file 2 — Source Data Fig. 1 [file 44321_2024_25_MOESM2_ESM.zip › figure 1/1J/1J/1J PDR OCT.tif]

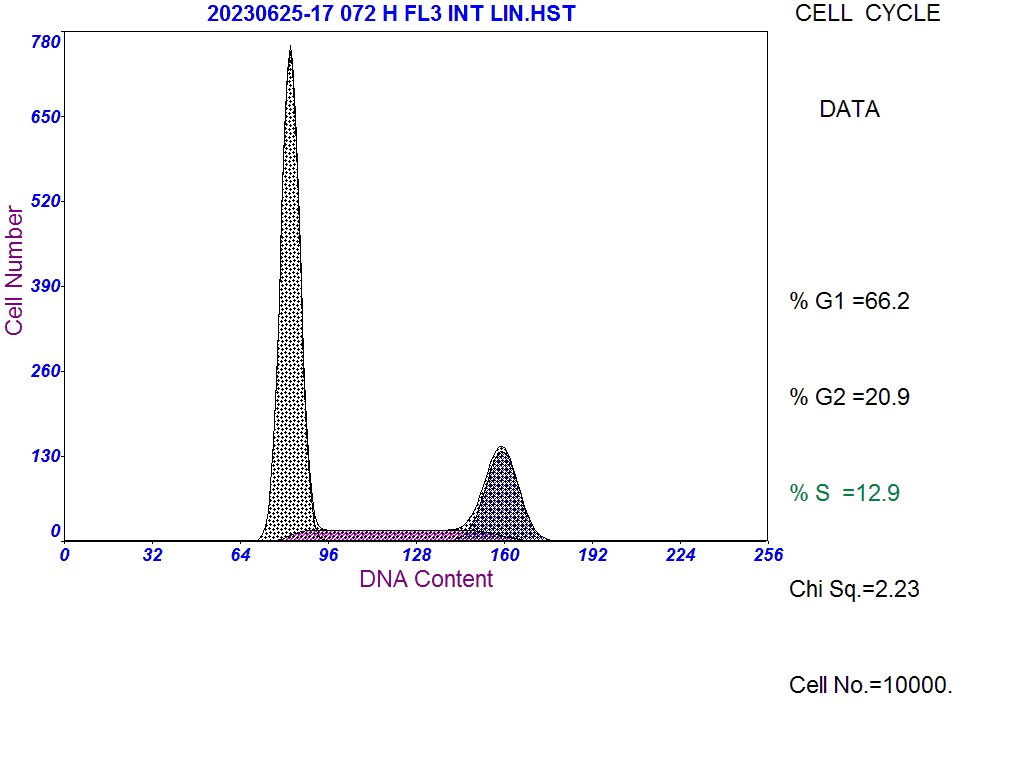

Supplement: Supplementary file 3 — Source Data Fig. 2 [file 44321_2024_25_MOESM3_ESM.zip › figure 2/2D/2D Ctrl.JPG]

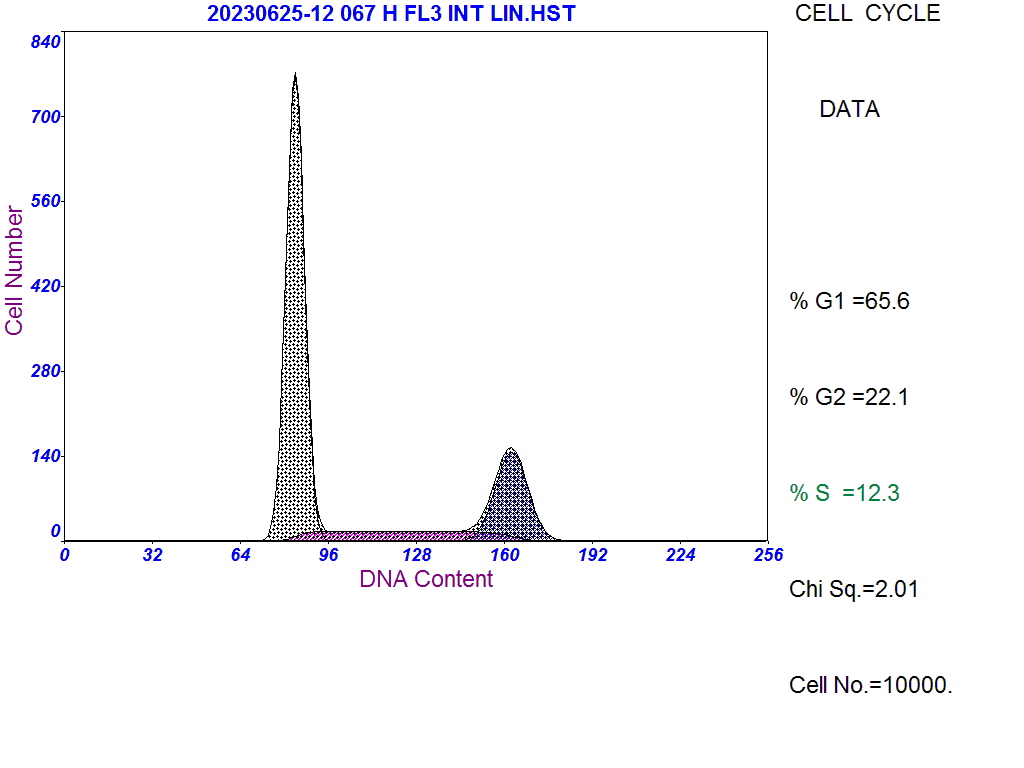

Supplement: Supplementary file 3 — Source Data Fig. 2 [file 44321_2024_25_MOESM3_ESM.zip › figure 2/2D/2D L-EV.JPG]

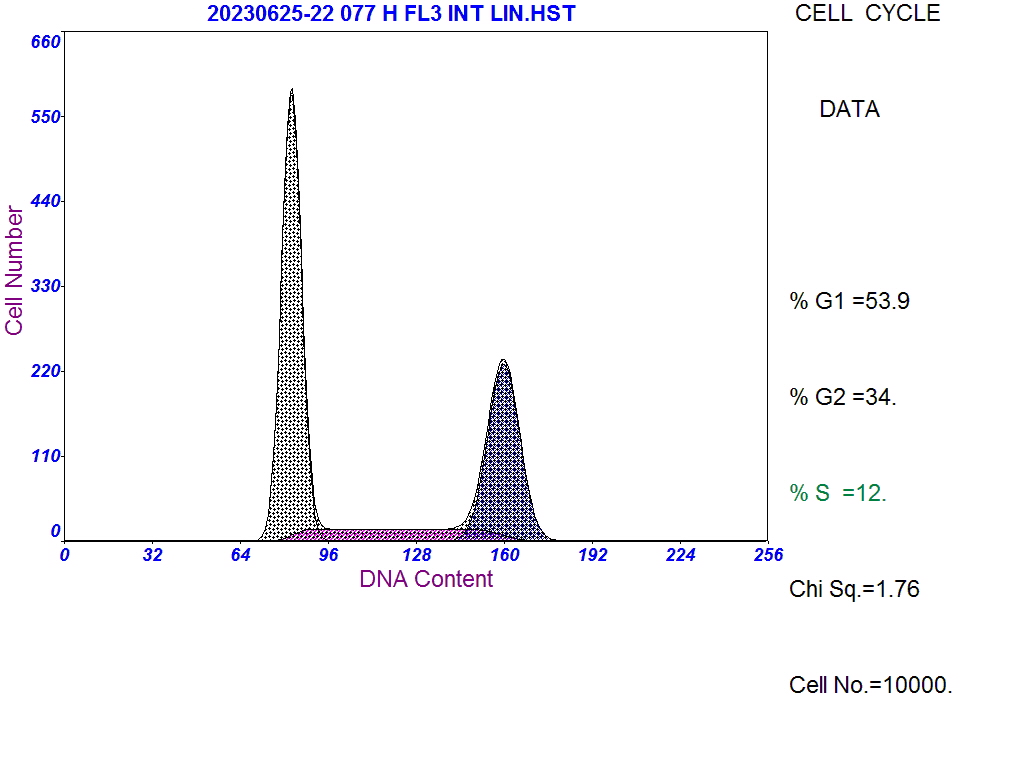

Supplement: Supplementary file 3 — Source Data Fig. 2 [file 44321_2024_25_MOESM3_ESM.zip › figure 2/2D/2D L-FTO.JPG]

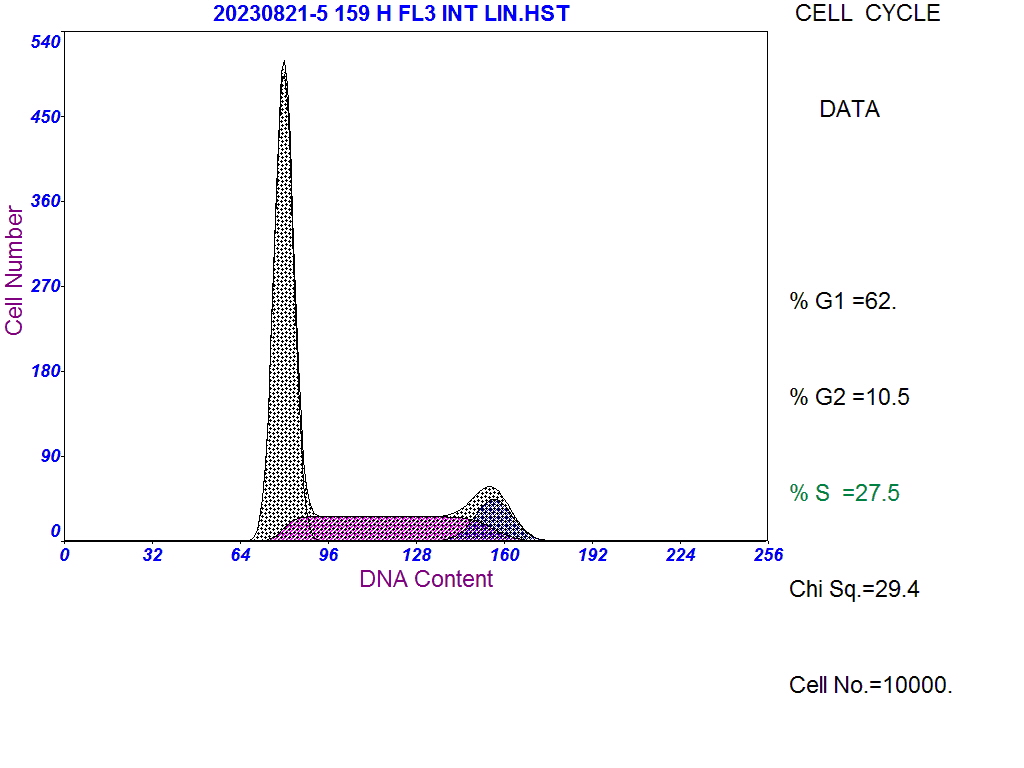

Supplement: Supplementary file 3 — Source Data Fig. 2 [file 44321_2024_25_MOESM3_ESM.zip › figure 2/2E/2E Ctrl.JPG]

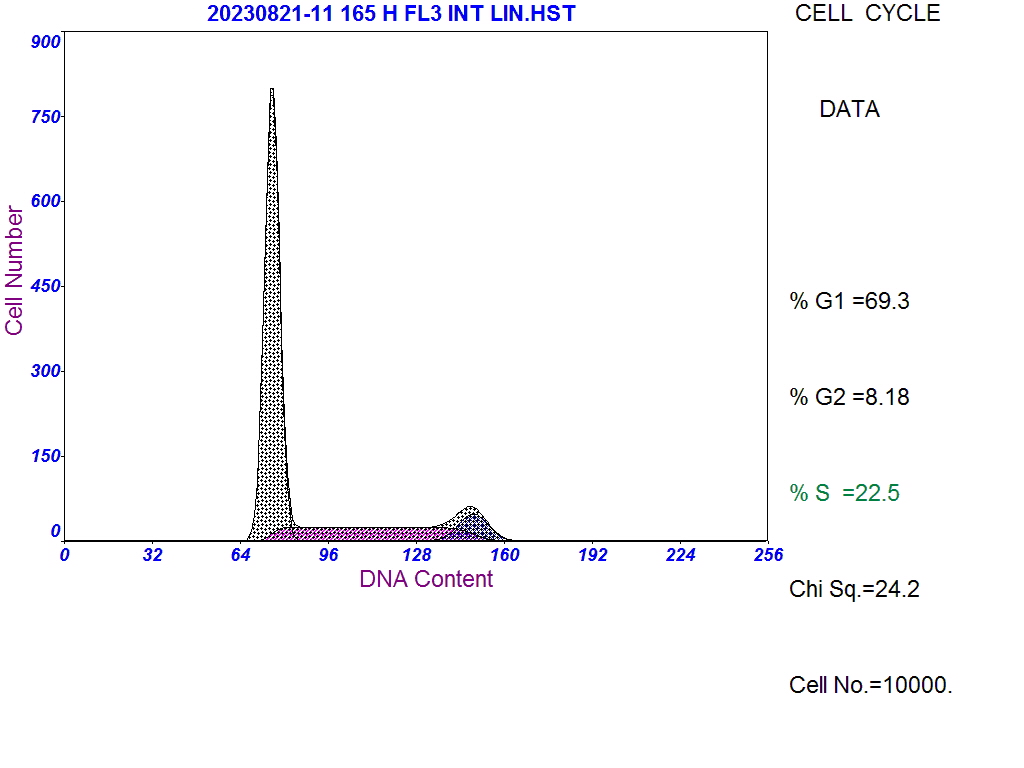

Supplement: Supplementary file 3 — Source Data Fig. 2 [file 44321_2024_25_MOESM3_ESM.zip › figure 2/2E/2E FTO-siRNA.JPG]

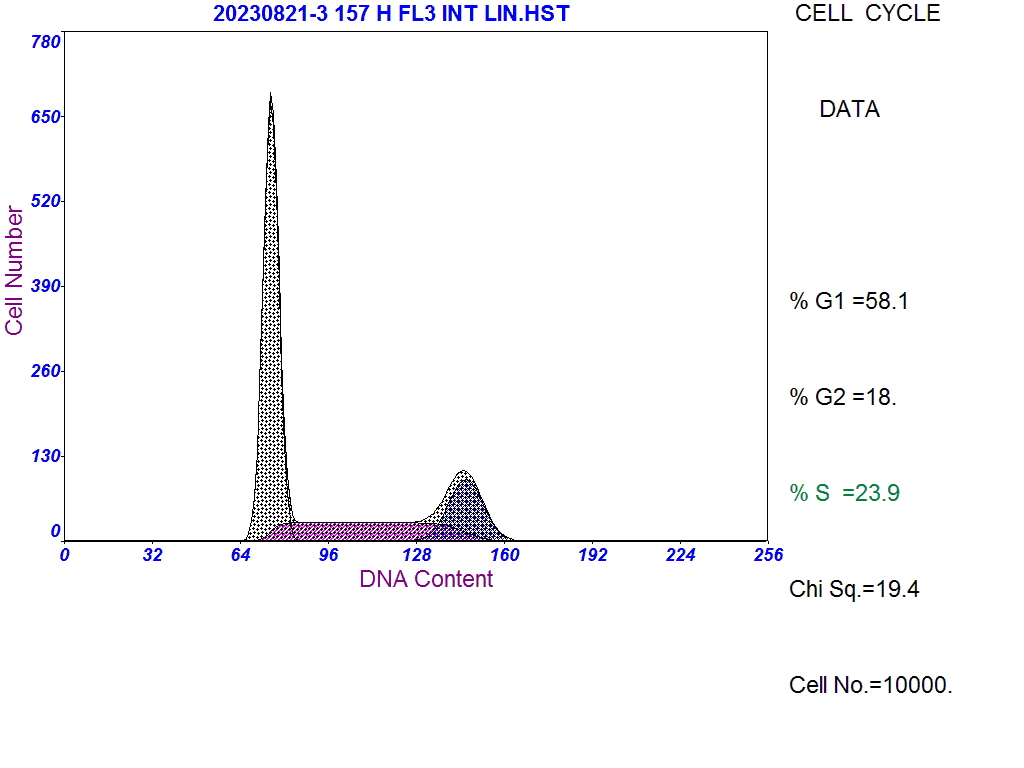

Supplement: Supplementary file 3 — Source Data Fig. 2 [file 44321_2024_25_MOESM3_ESM.zip › figure 2/2E/2E scramble siRNA.JPG]

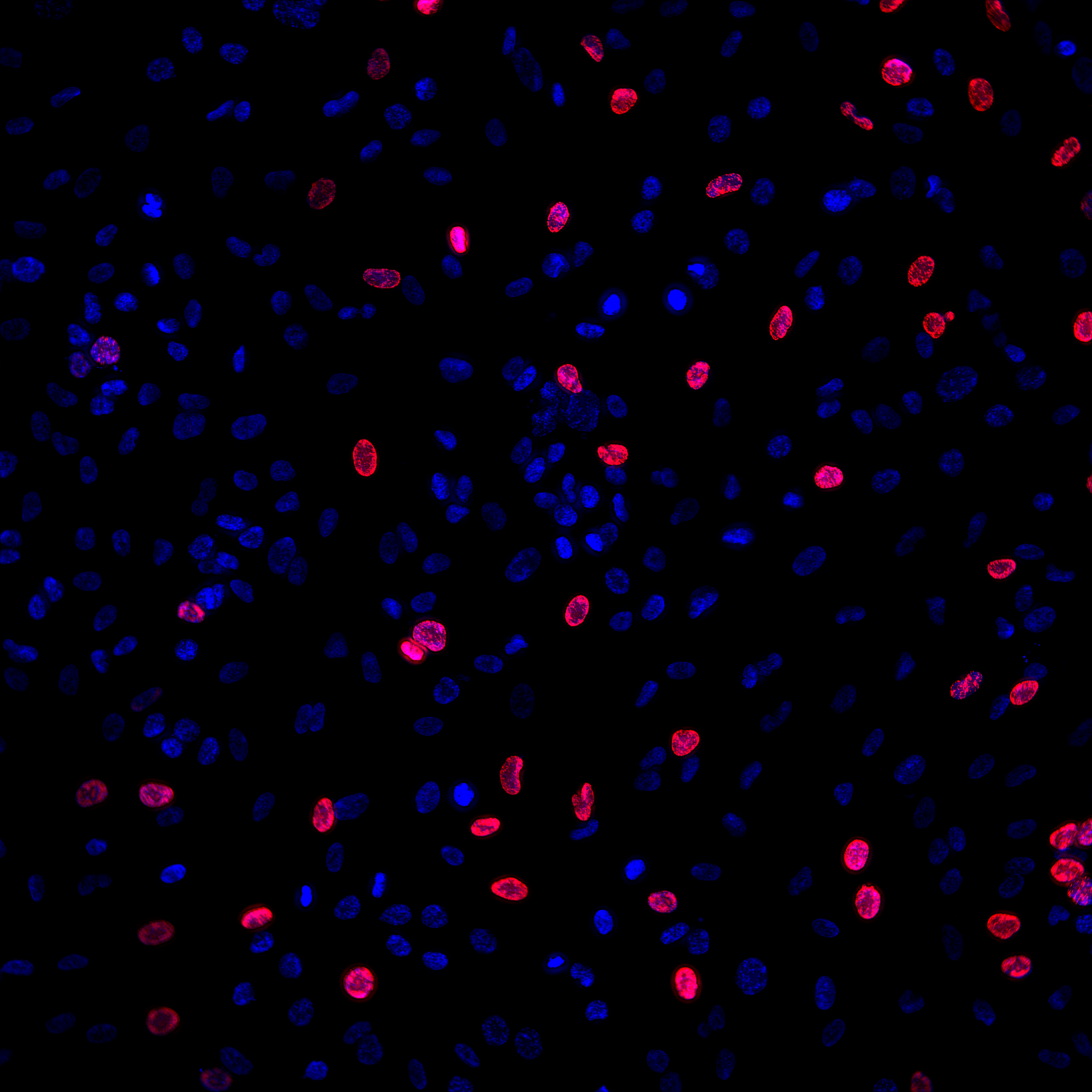

Supplement: Supplementary file 3 — Source Data Fig. 2 [file 44321_2024_25_MOESM3_ESM.zip › figure 2/2F/2F Ctrl EdU DAPI.tif]

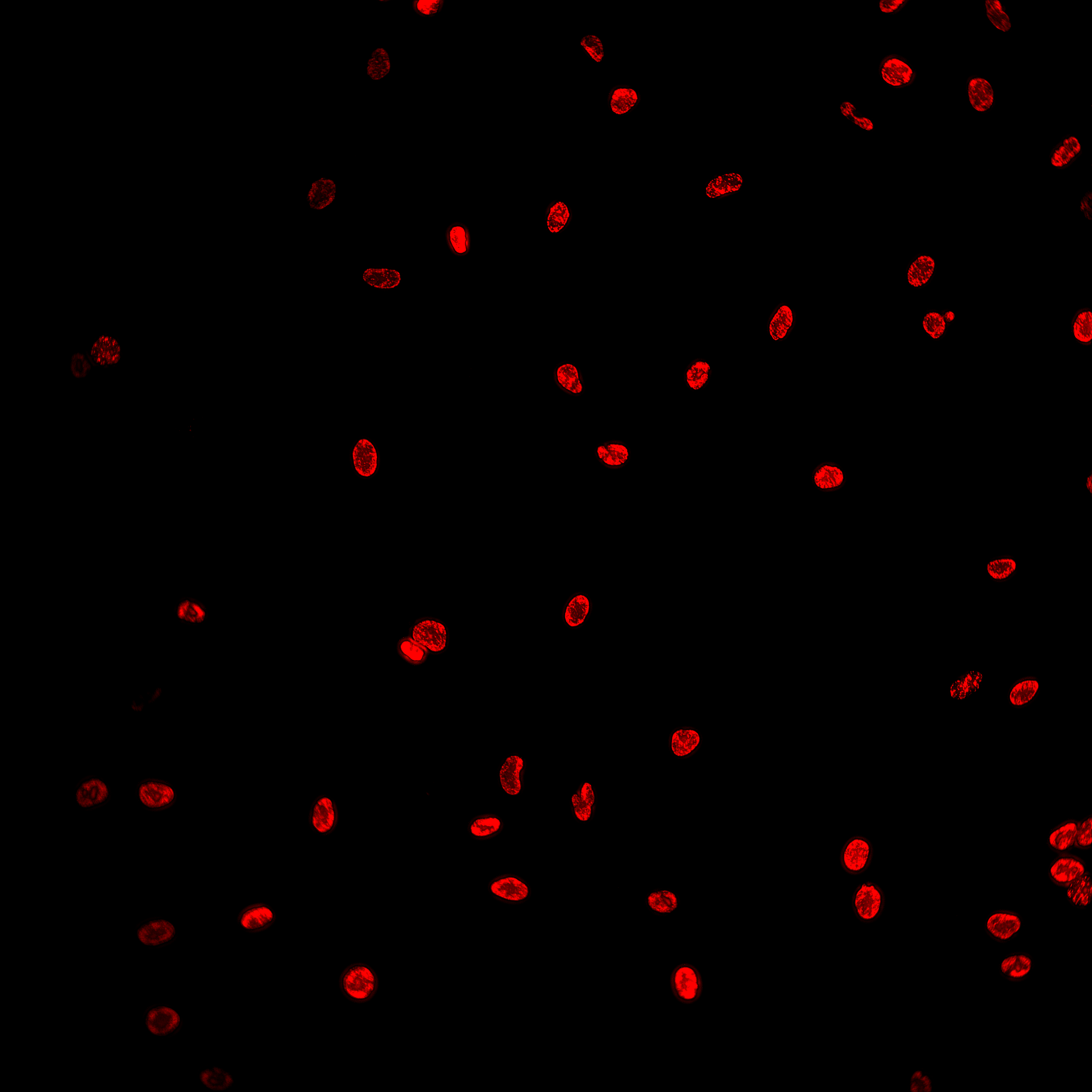

Supplement: Supplementary file 3 — Source Data Fig. 2 [file 44321_2024_25_MOESM3_ESM.zip › figure 2/2F/2F Ctrl EdU.tif]

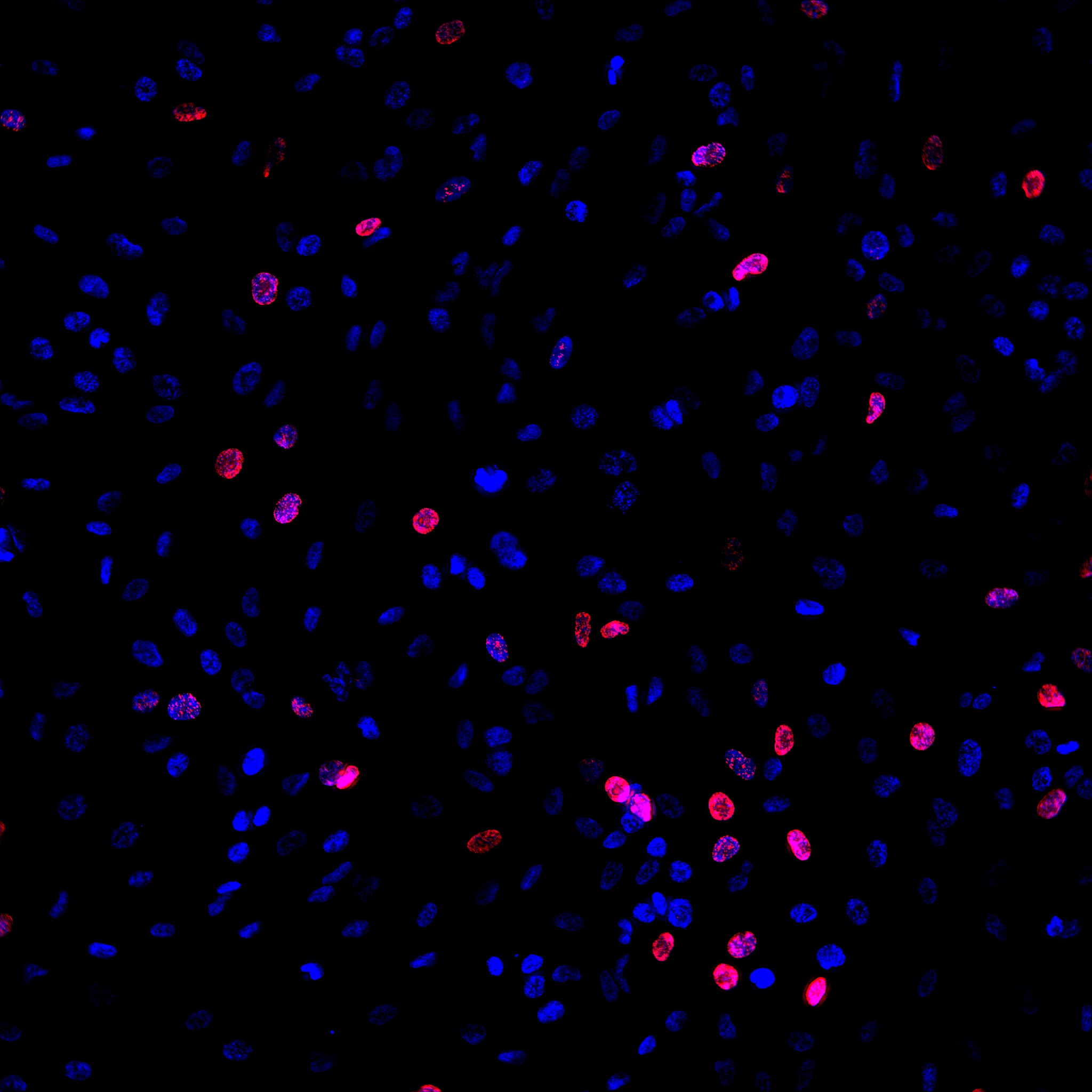

Supplement: Supplementary file 3 — Source Data Fig. 2 [file 44321_2024_25_MOESM3_ESM.zip › figure 2/2F/2F L-EV EdU DAPI.tif]

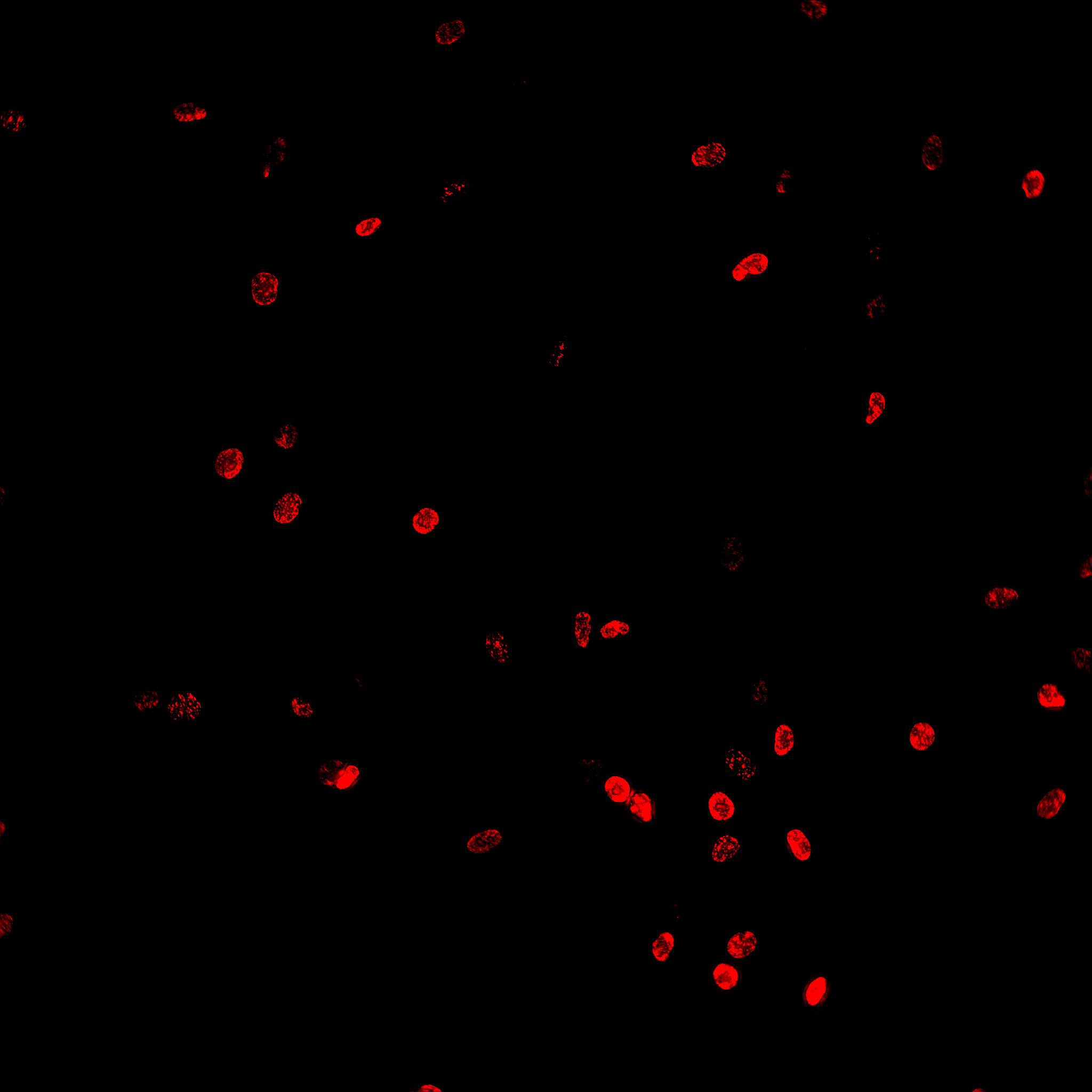

Supplement: Supplementary file 3 — Source Data Fig. 2 [file 44321_2024_25_MOESM3_ESM.zip › figure 2/2F/2F L-EV EdU.tif]

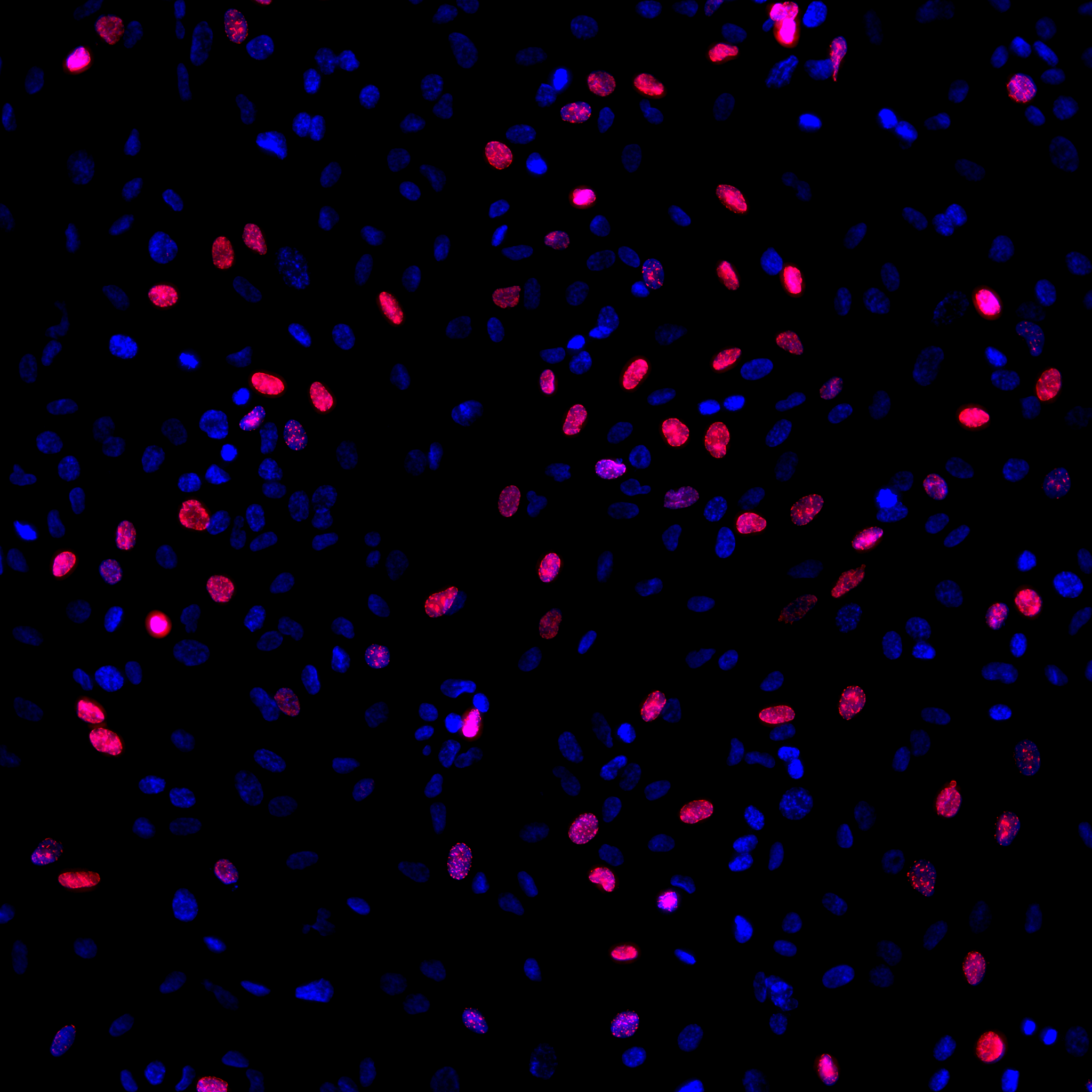

Supplement: Supplementary file 3 — Source Data Fig. 2 [file 44321_2024_25_MOESM3_ESM.zip › figure 2/2F/2F L-FTO EdU DAPI.tif]

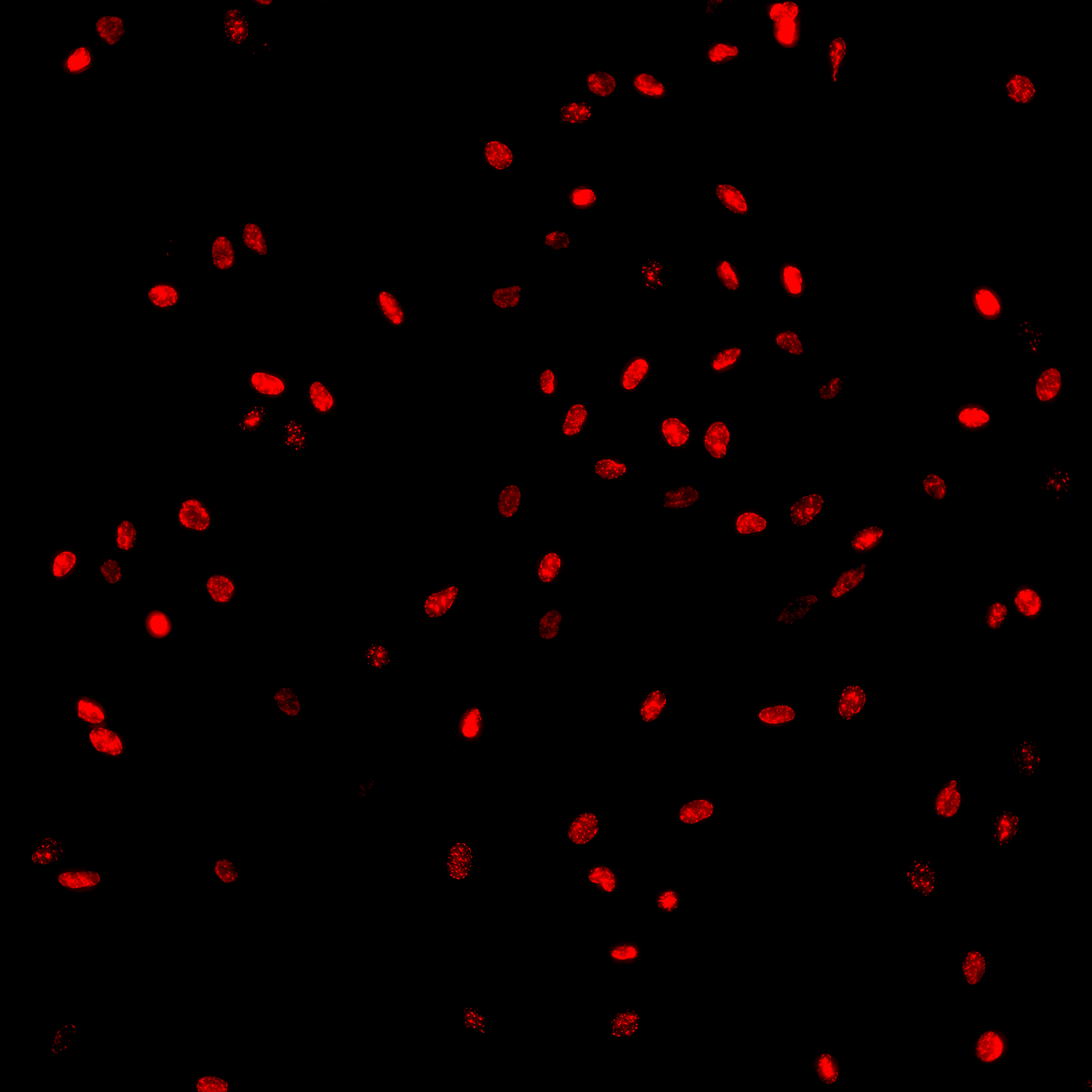

Supplement: Supplementary file 3 — Source Data Fig. 2 [file 44321_2024_25_MOESM3_ESM.zip › figure 2/2F/2F L-FTO EdU.tif]

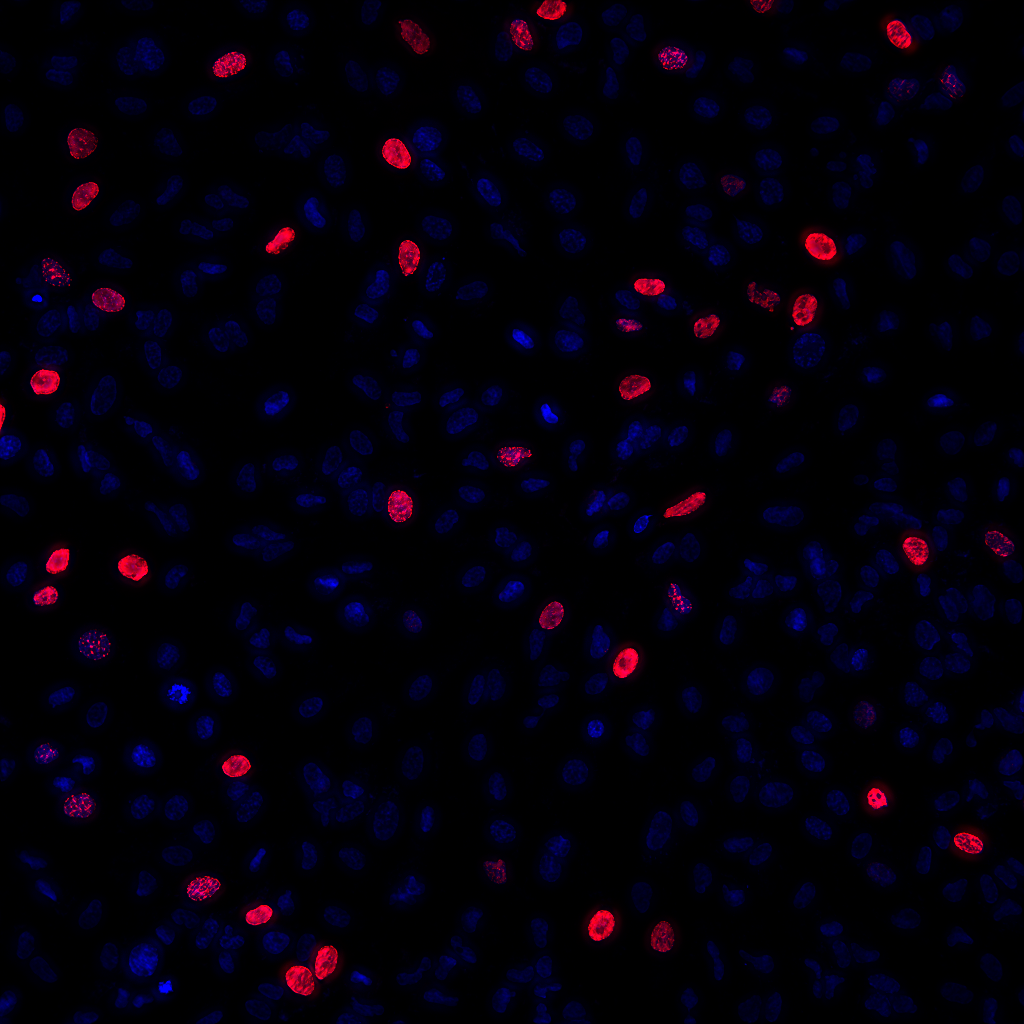

Supplement: Supplementary file 3 — Source Data Fig. 2 [file 44321_2024_25_MOESM3_ESM.zip › figure 2/2G/2G Ctrl EdU DAPI.tif]

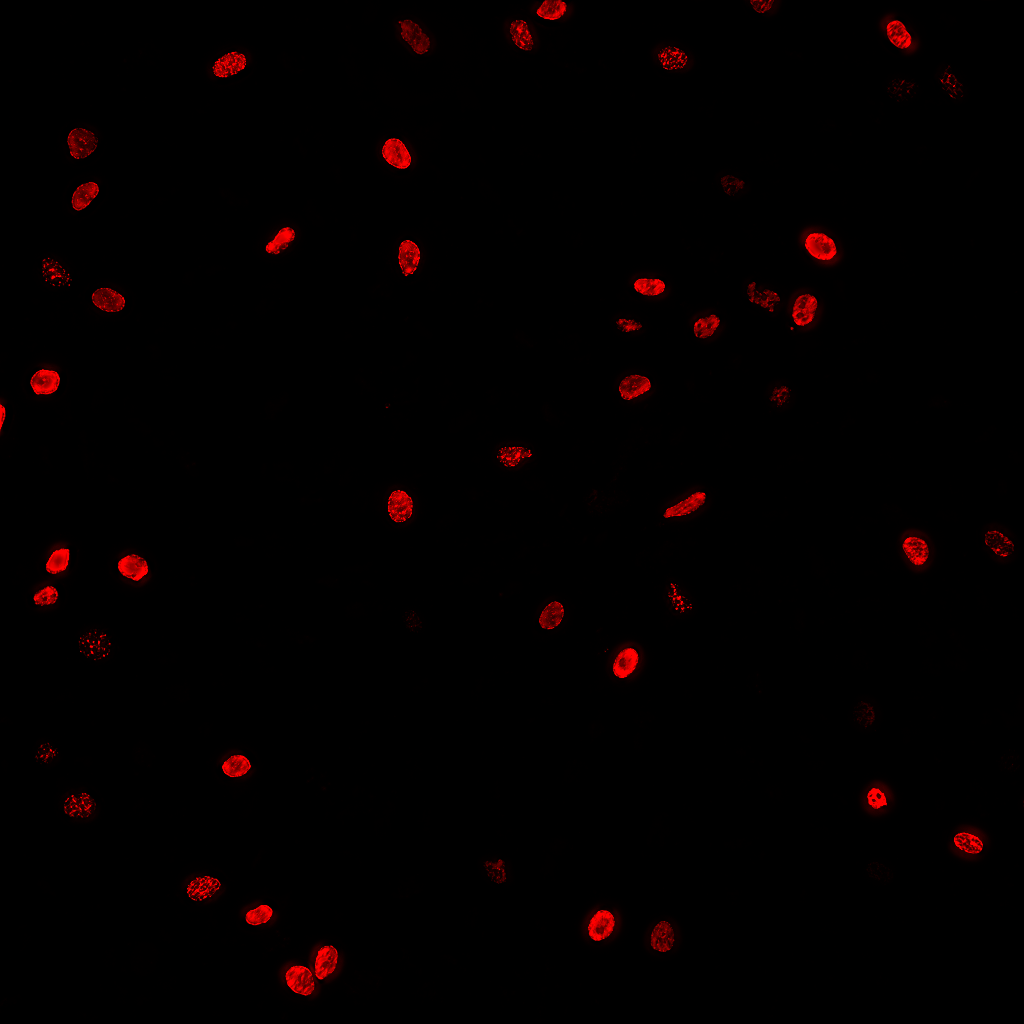

Supplement: Supplementary file 3 — Source Data Fig. 2 [file 44321_2024_25_MOESM3_ESM.zip › figure 2/2G/2G Ctrl EdU.tif]

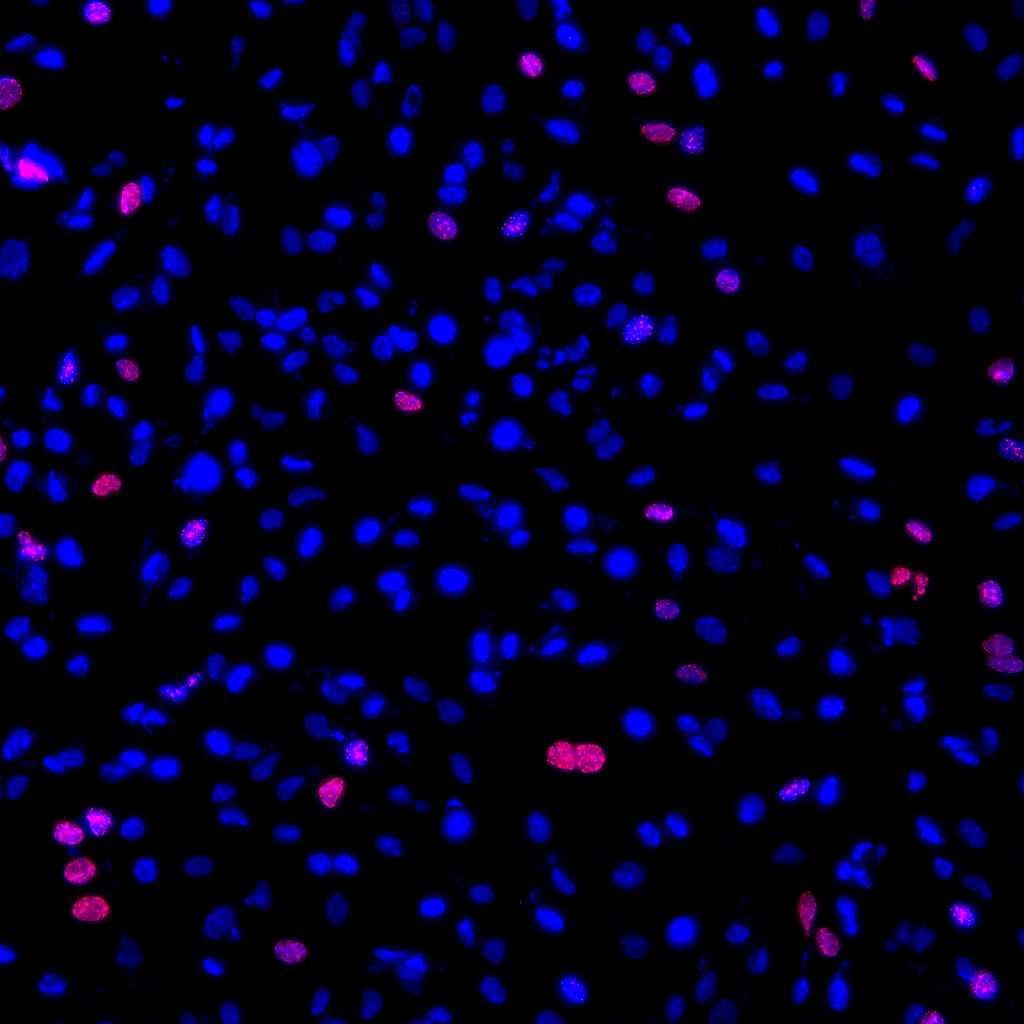

Supplement: Supplementary file 3 — Source Data Fig. 2 [file 44321_2024_25_MOESM3_ESM.zip › figure 2/2G/2G FTO-siRNA EdU DAPI.tif]

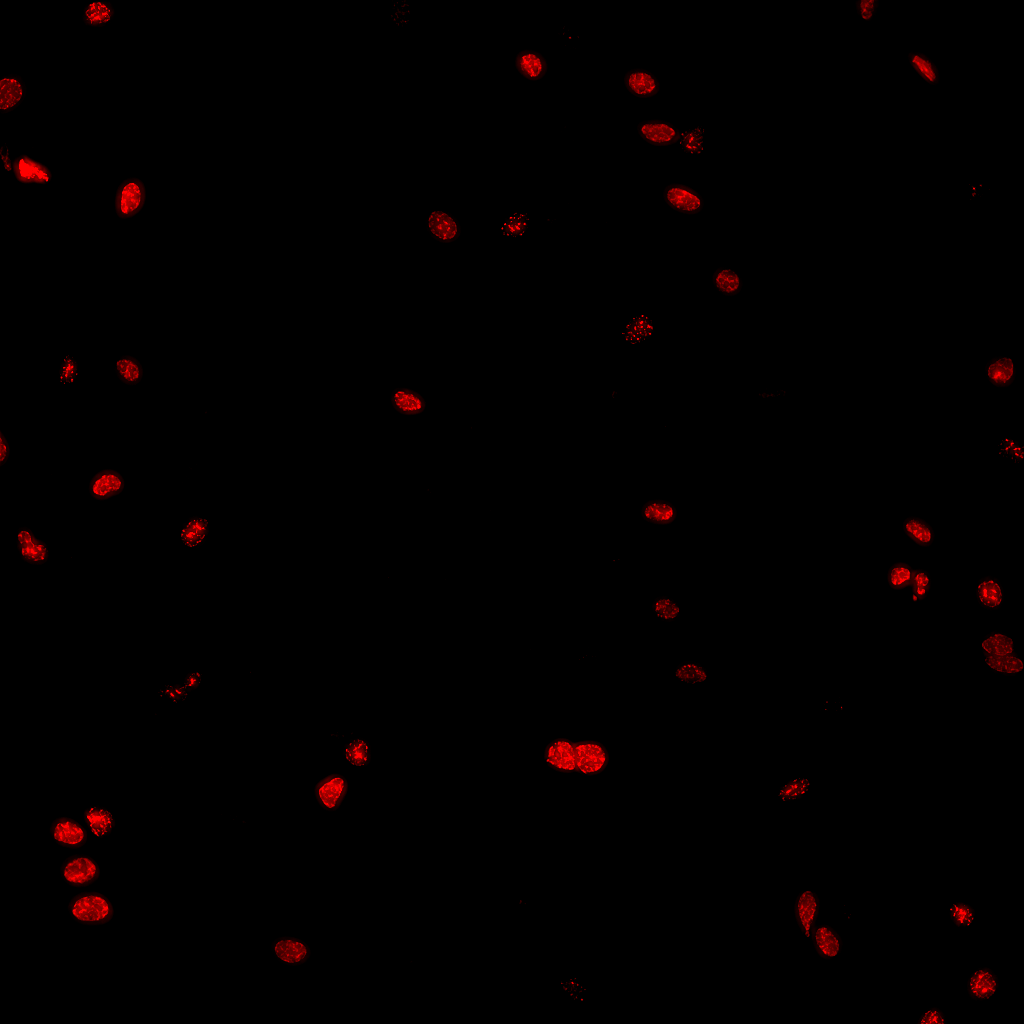

Supplement: Supplementary file 3 — Source Data Fig. 2 [file 44321_2024_25_MOESM3_ESM.zip › figure 2/2G/2G FTO-siRNA EdU.tif]

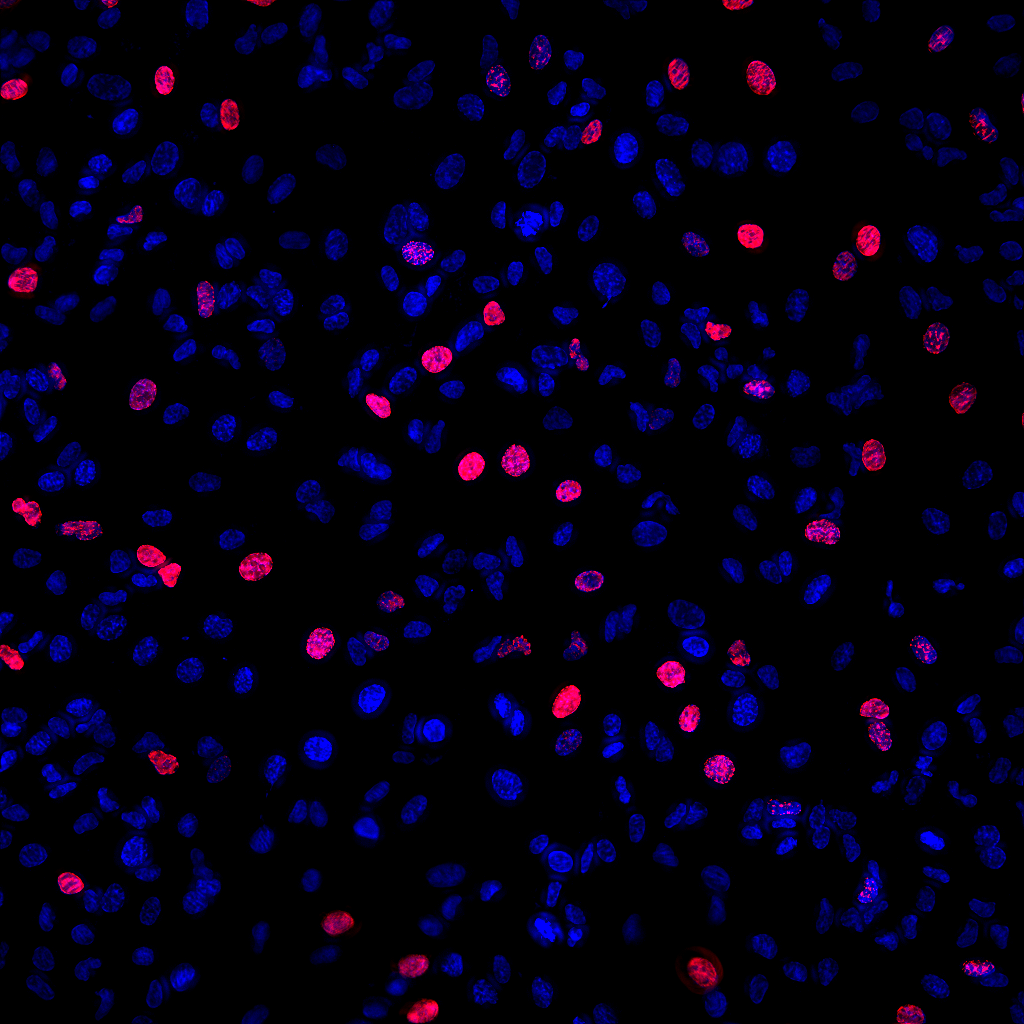

Supplement: Supplementary file 3 — Source Data Fig. 2 [file 44321_2024_25_MOESM3_ESM.zip › figure 2/2G/2G scramble siRNA EdU DAPI.tif]

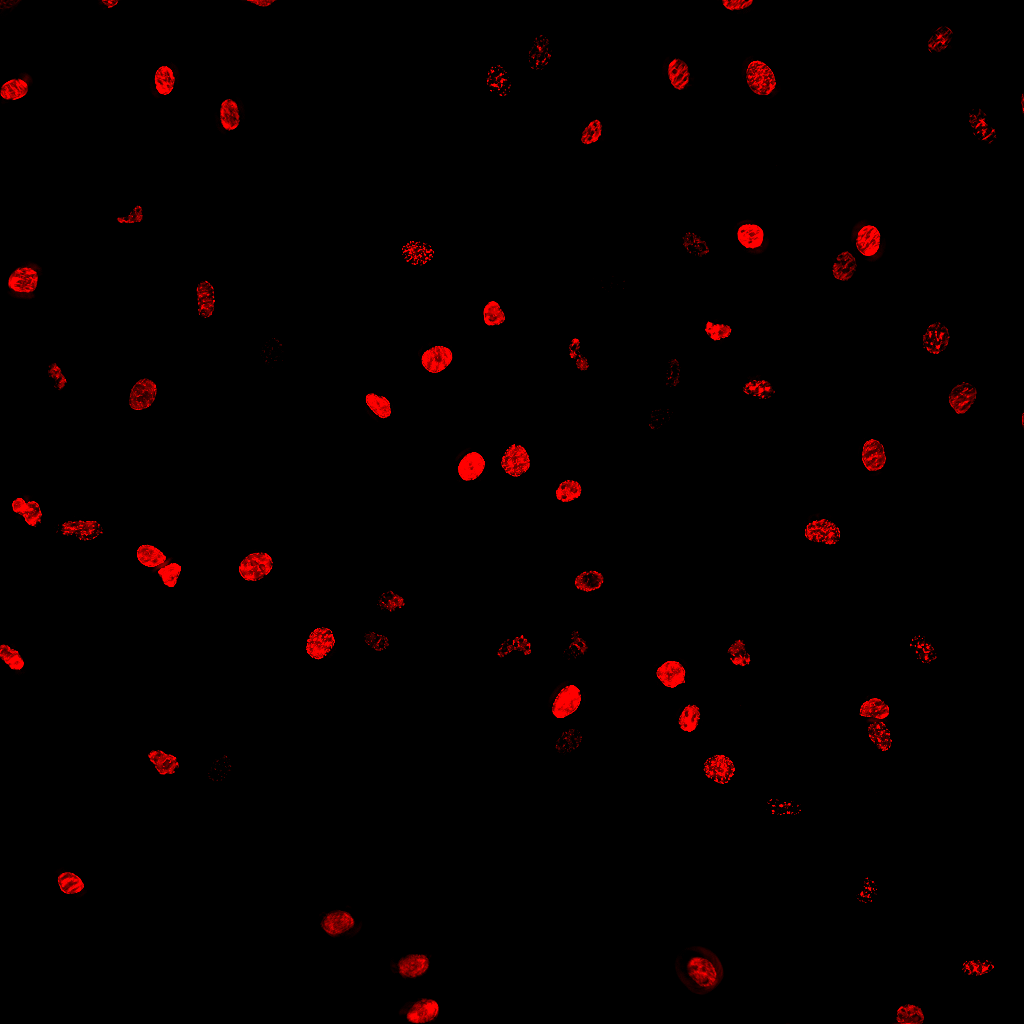

Supplement: Supplementary file 3 — Source Data Fig. 2 [file 44321_2024_25_MOESM3_ESM.zip › figure 2/2G/2G scramble siRNA EdU.tif]

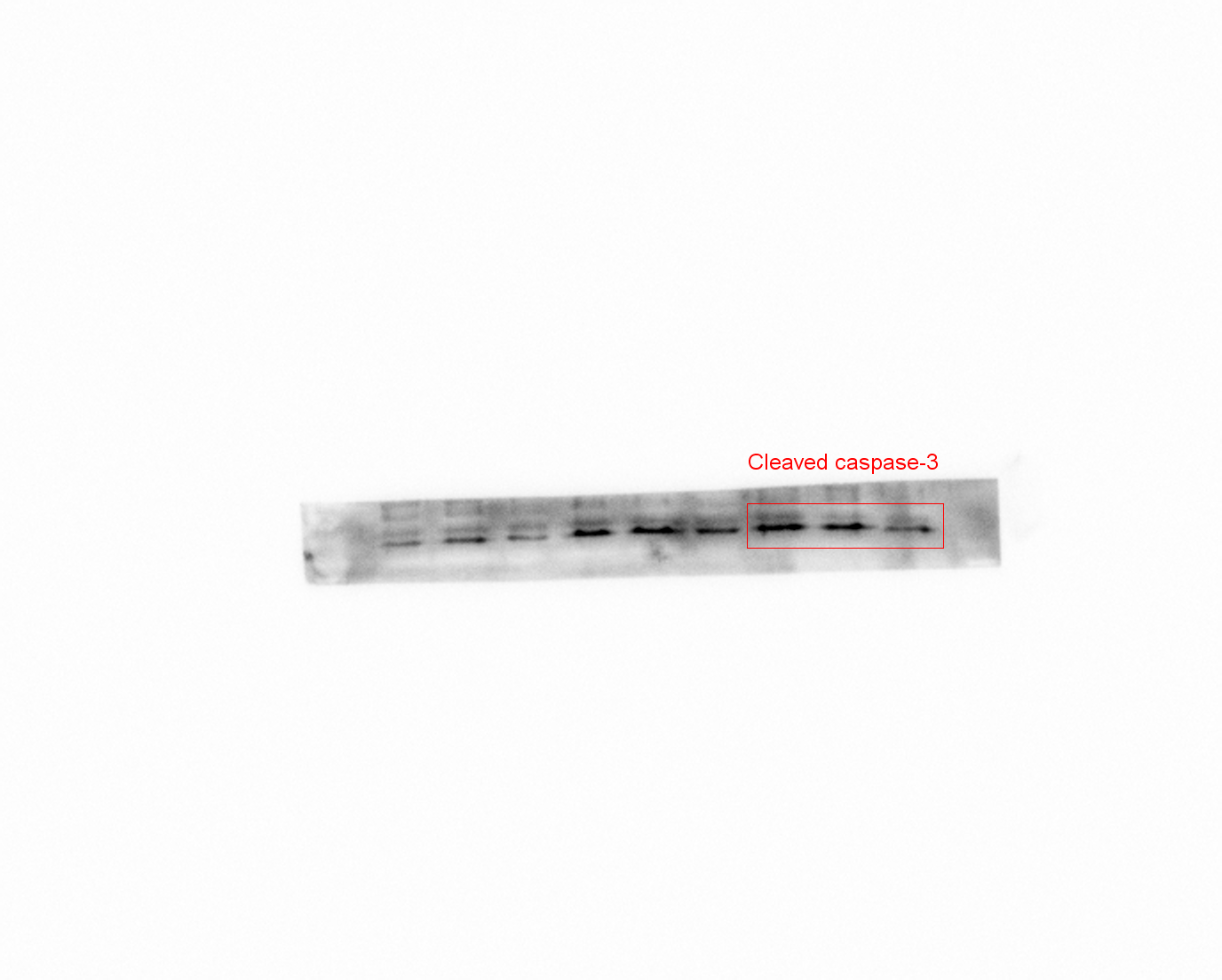

Supplement: Supplementary file 3 — Source Data Fig. 2 [file 44321_2024_25_MOESM3_ESM.zip › figure 2/2H/2H Cleaved caspase-3 mark.Tif]

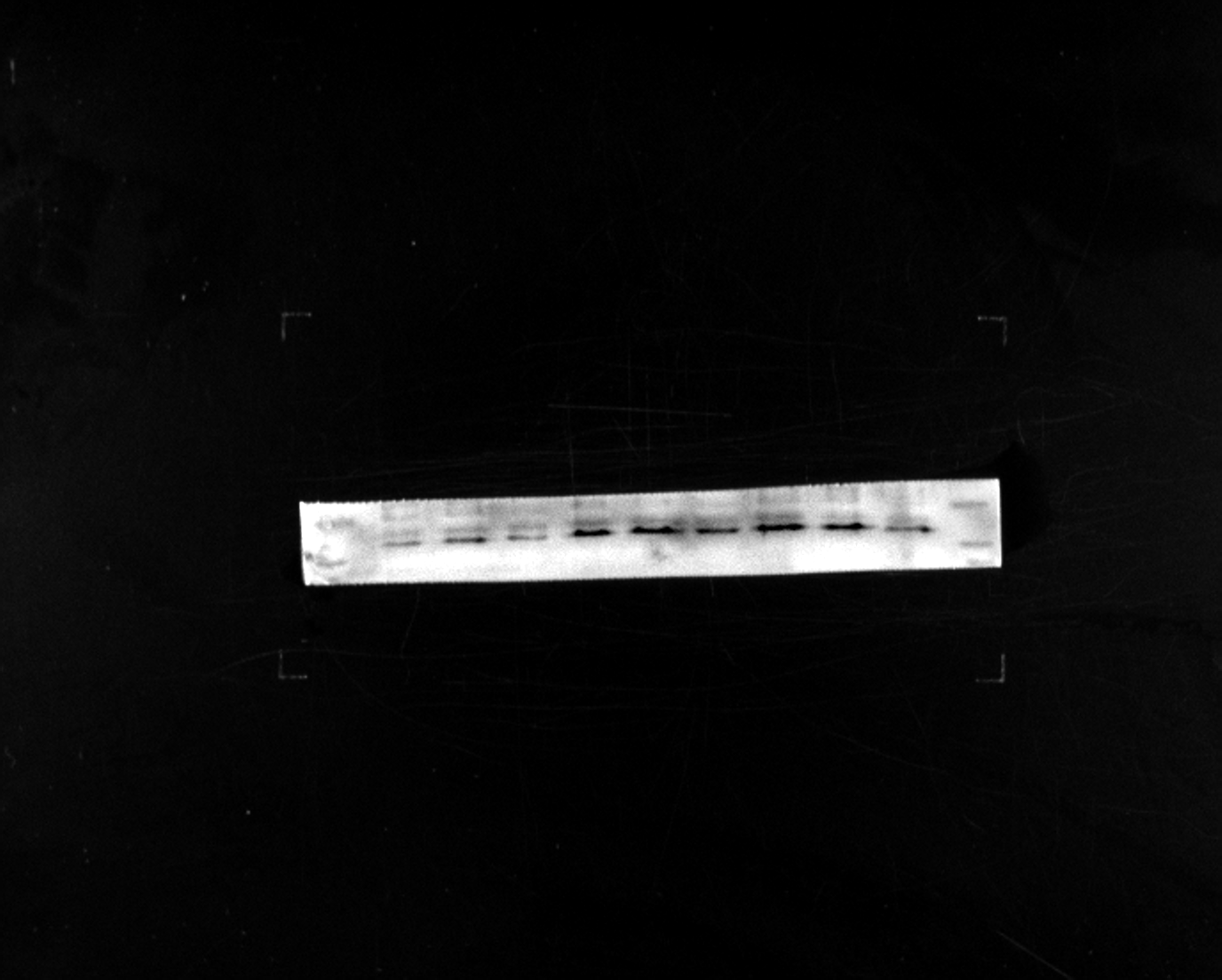

Supplement: Supplementary file 3 — Source Data Fig. 2 [file 44321_2024_25_MOESM3_ESM.zip › figure 2/2H/2H Cleaved caspase-3.Tif]

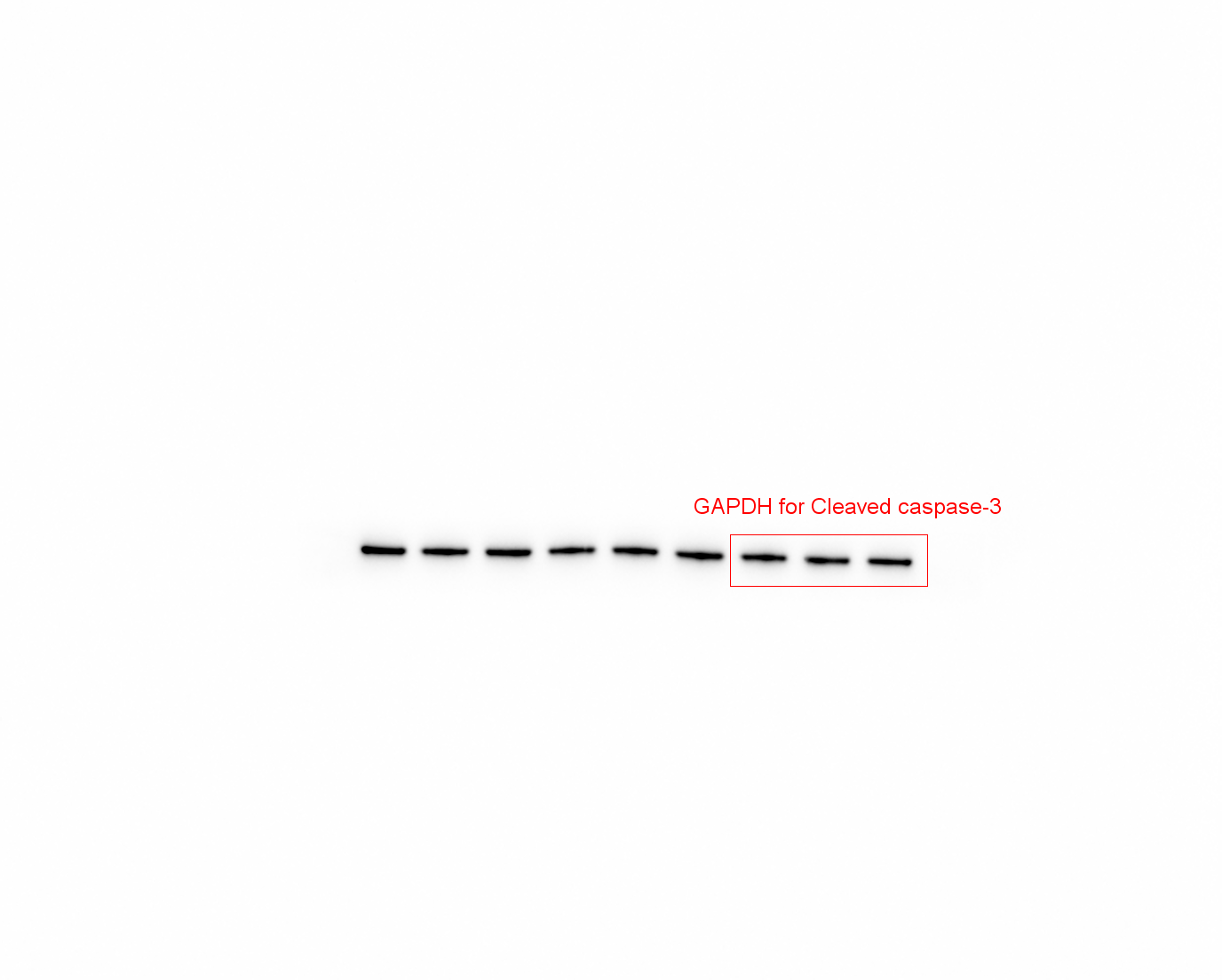

Supplement: Supplementary file 3 — Source Data Fig. 2 [file 44321_2024_25_MOESM3_ESM.zip › figure 2/2H/2H GAPDH for Cleaved caspase-3 mark.Tif]

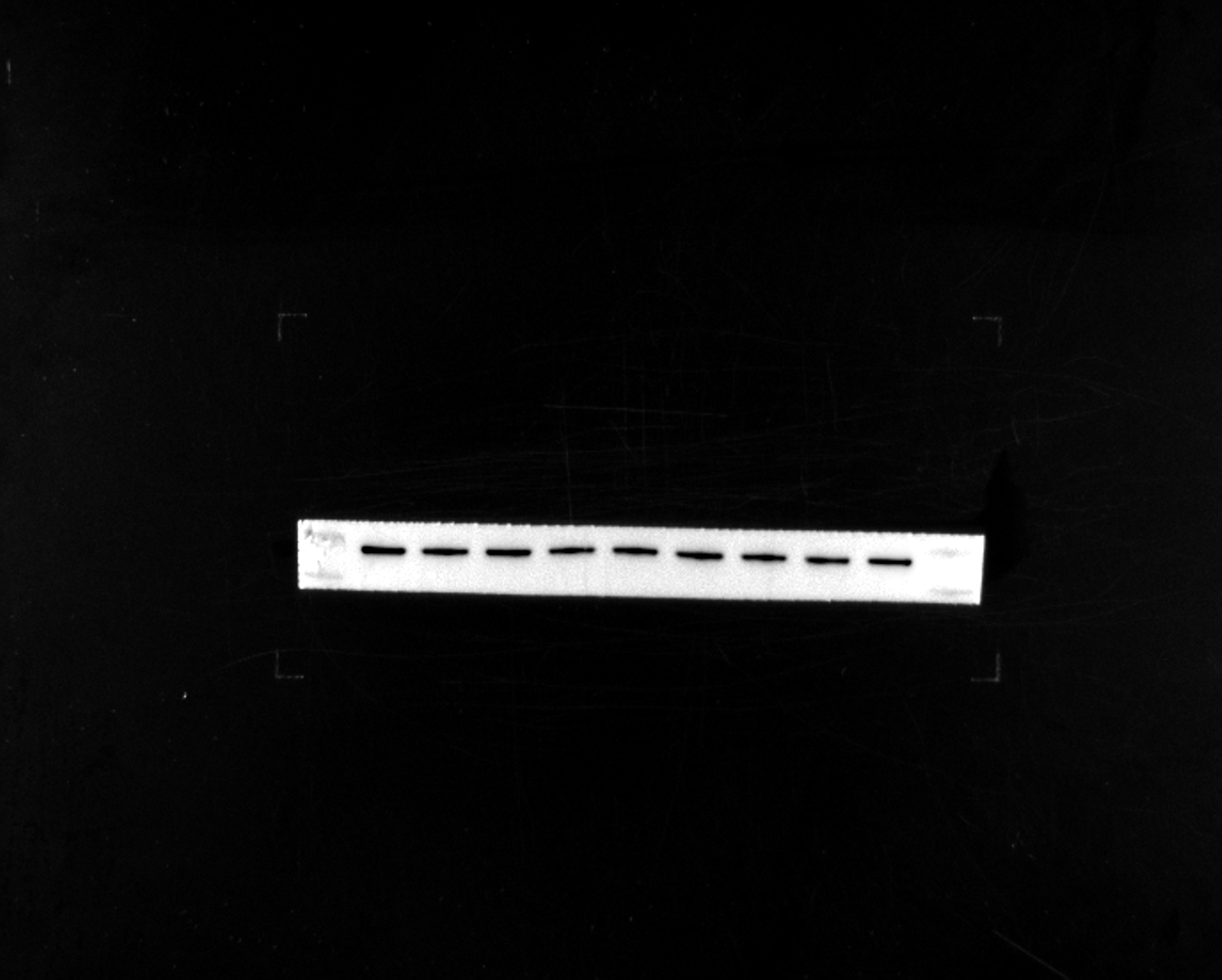

Supplement: Supplementary file 3 — Source Data Fig. 2 [file 44321_2024_25_MOESM3_ESM.zip › figure 2/2H/2H GAPDH for Cleaved caspase-3.Tif]

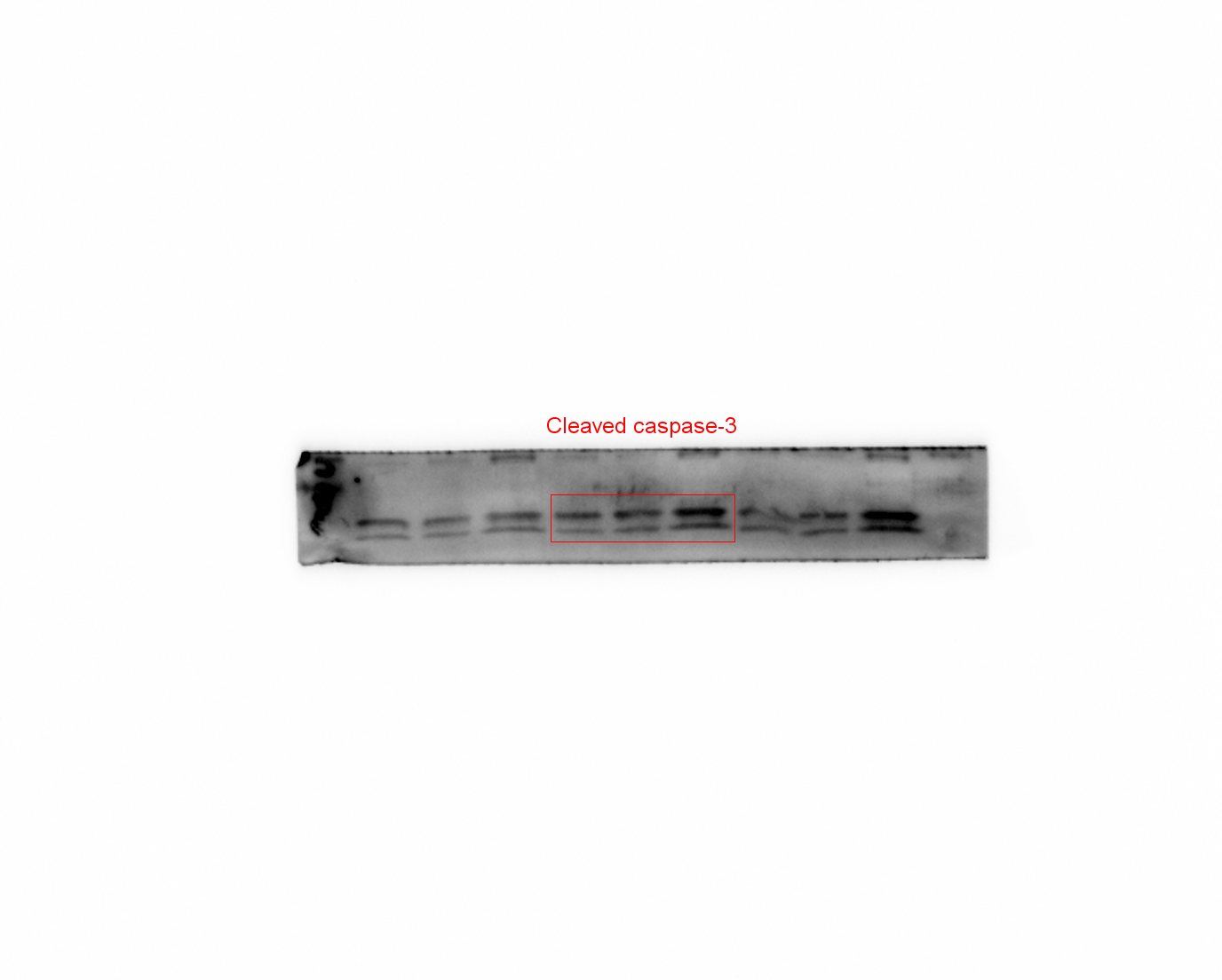

Supplement: Supplementary file 3 — Source Data Fig. 2 [file 44321_2024_25_MOESM3_ESM.zip › figure 2/2I/2I Cleaved caspase-3 mark.Tif]

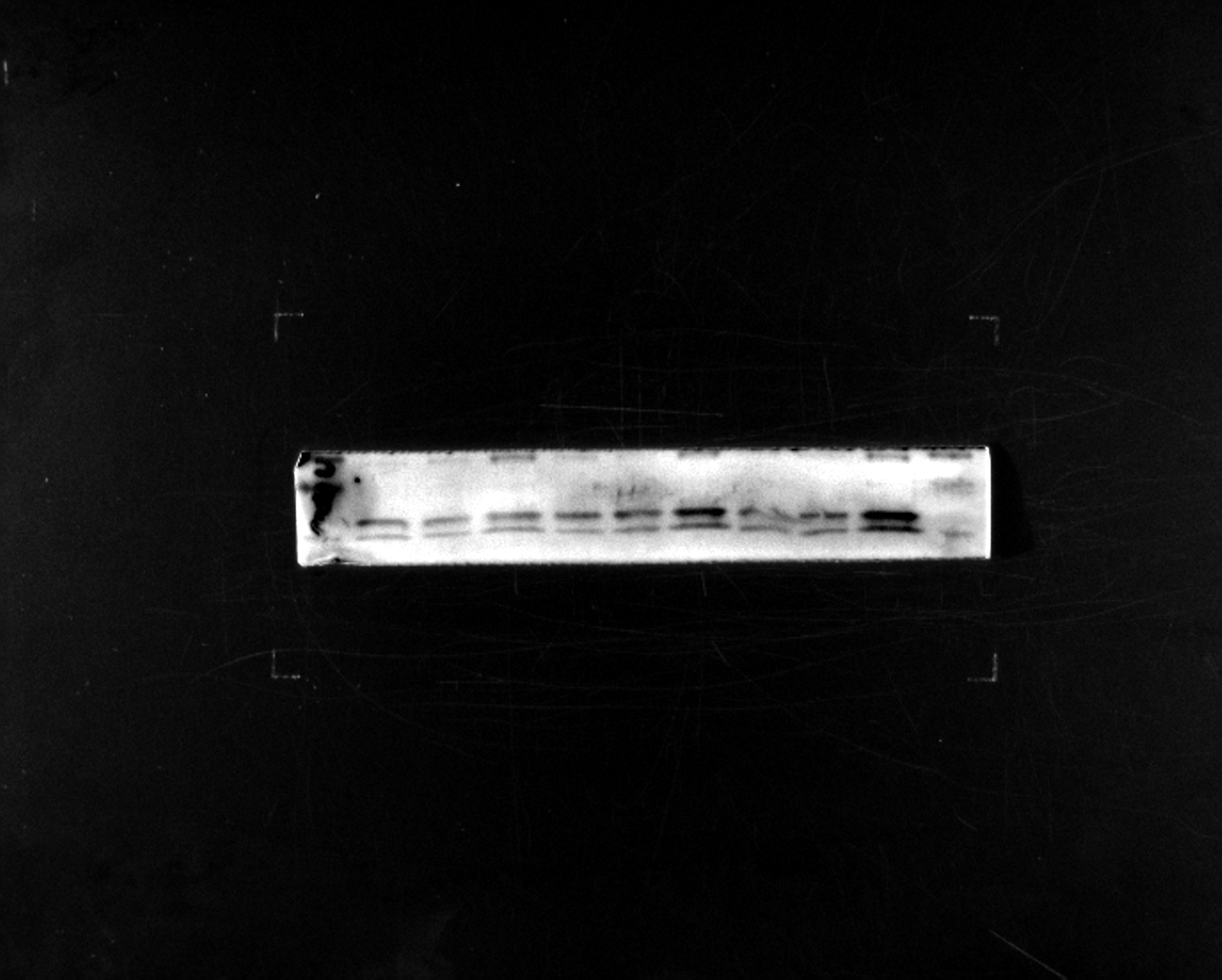

Supplement: Supplementary file 3 — Source Data Fig. 2 [file 44321_2024_25_MOESM3_ESM.zip › figure 2/2I/2I Cleaved caspase-3.Tif]

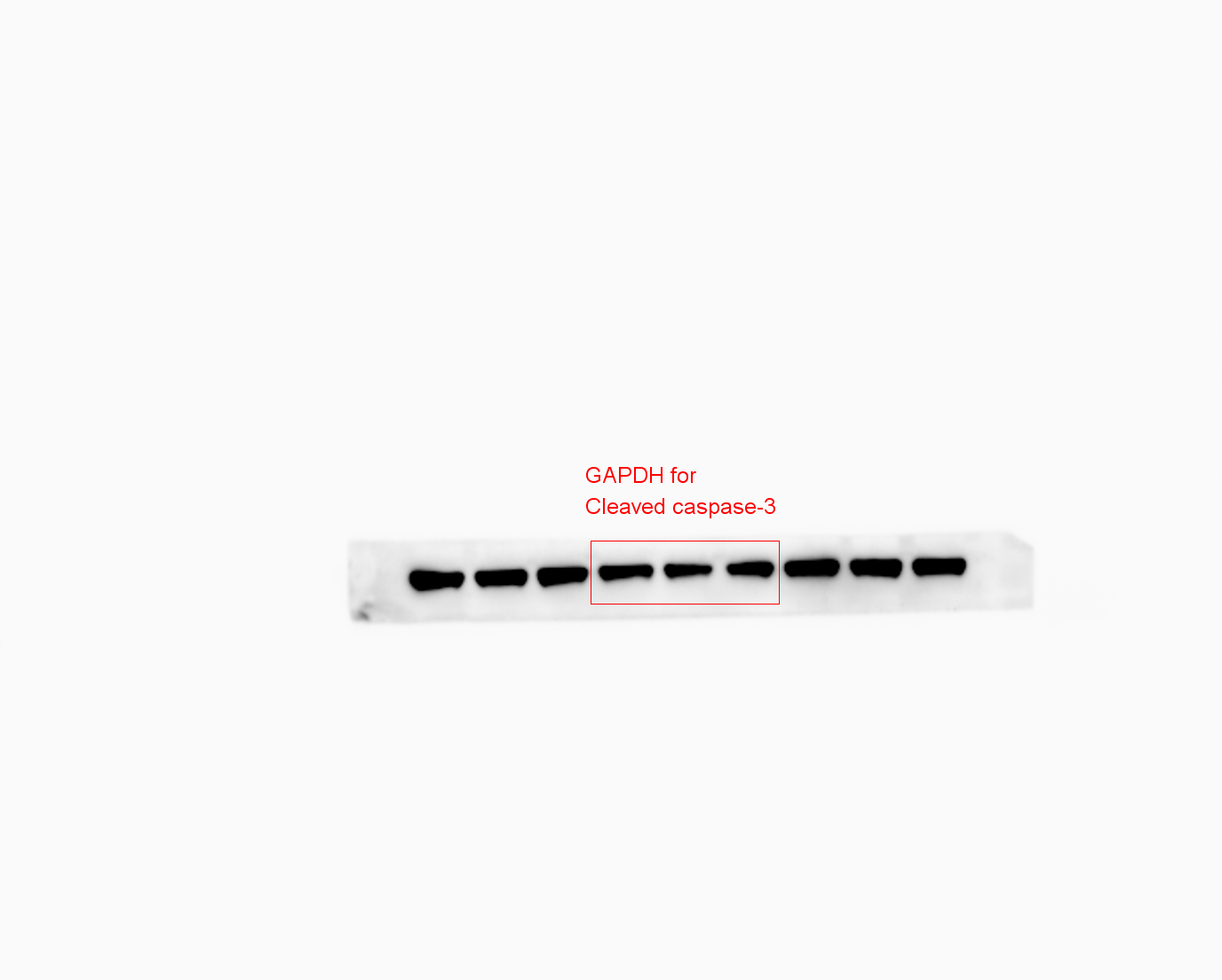

Supplement: Supplementary file 3 — Source Data Fig. 2 [file 44321_2024_25_MOESM3_ESM.zip › figure 2/2I/2I GAPDH for Cleaved caspase-3 mark.Tif]

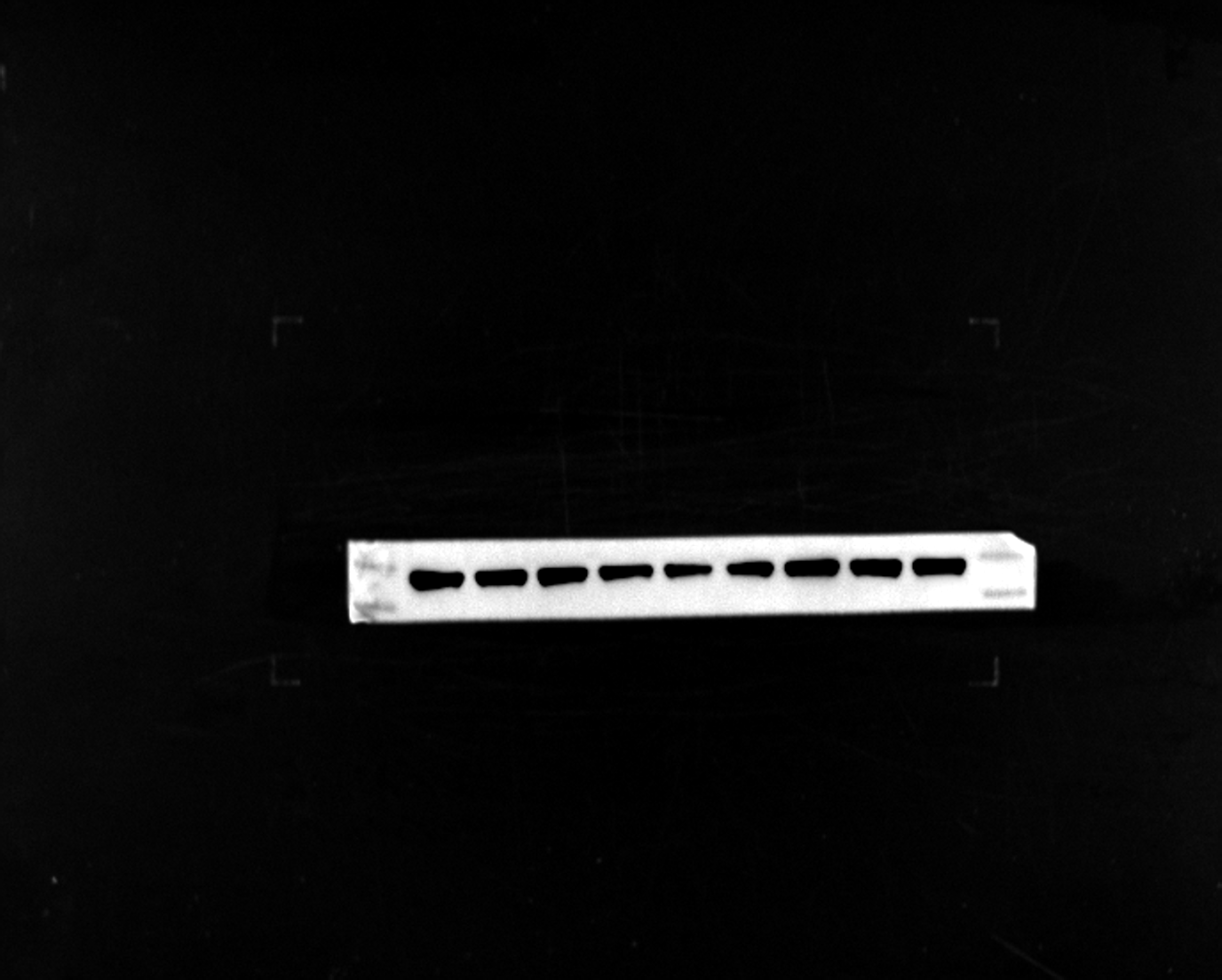

Supplement: Supplementary file 3 — Source Data Fig. 2 [file 44321_2024_25_MOESM3_ESM.zip › figure 2/2I/2I GAPDH for Cleaved caspase-3.Tif]

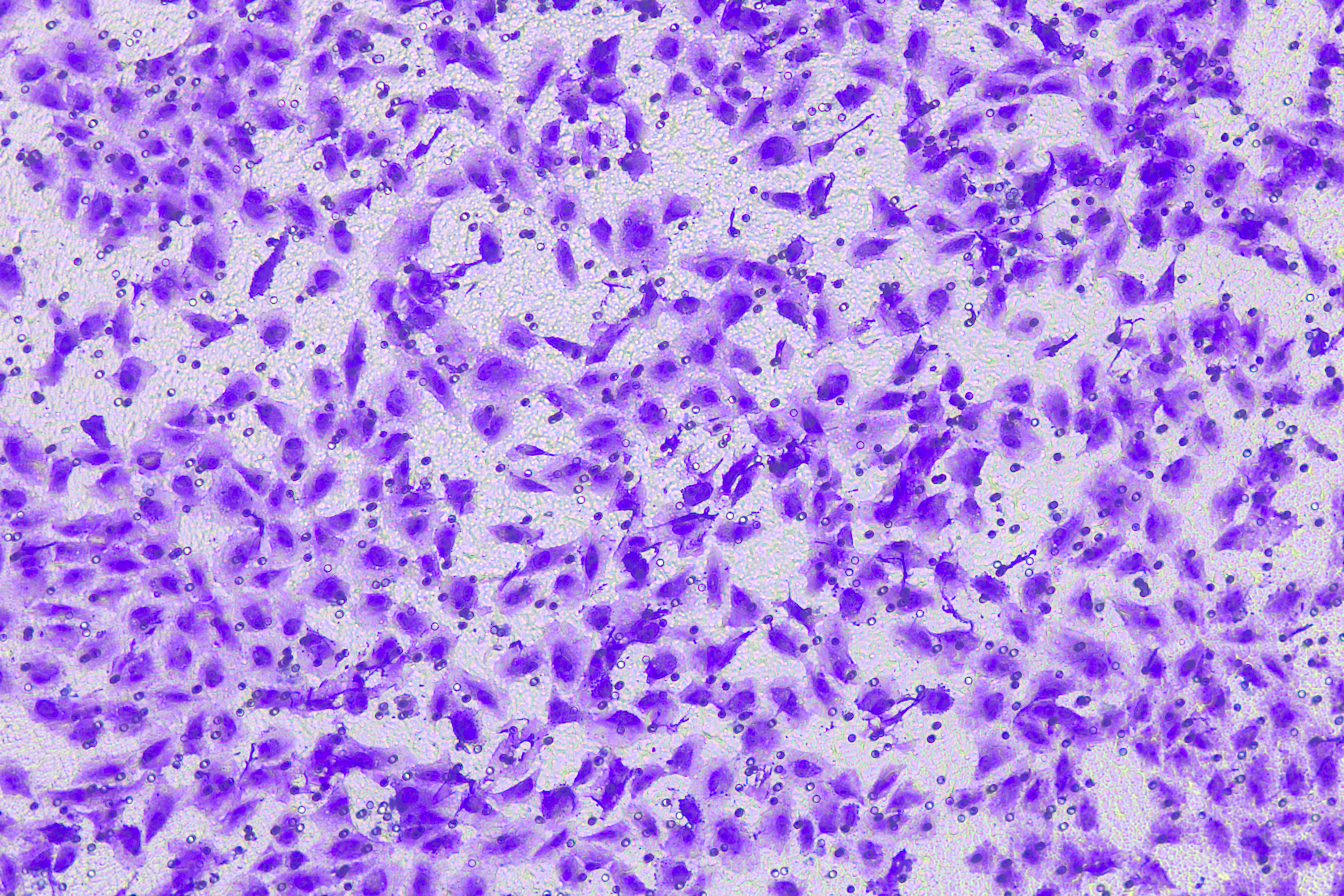

Supplement: Supplementary file 3 — Source Data Fig. 2 [file 44321_2024_25_MOESM3_ESM.zip › figure 2/2L/2L Ctrl.tif]

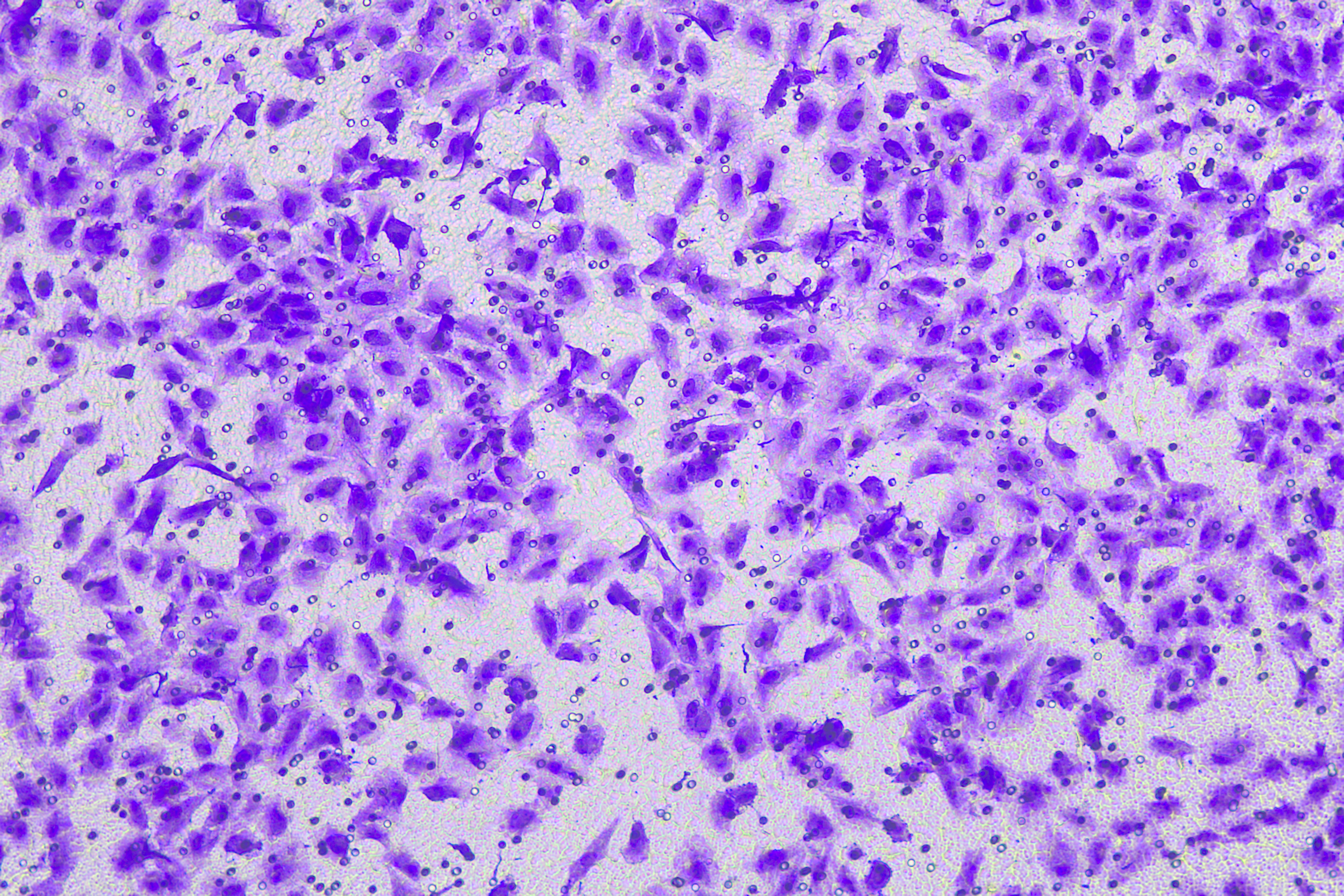

Supplement: Supplementary file 3 — Source Data Fig. 2 [file 44321_2024_25_MOESM3_ESM.zip › figure 2/2L/2L L-EV.tif]

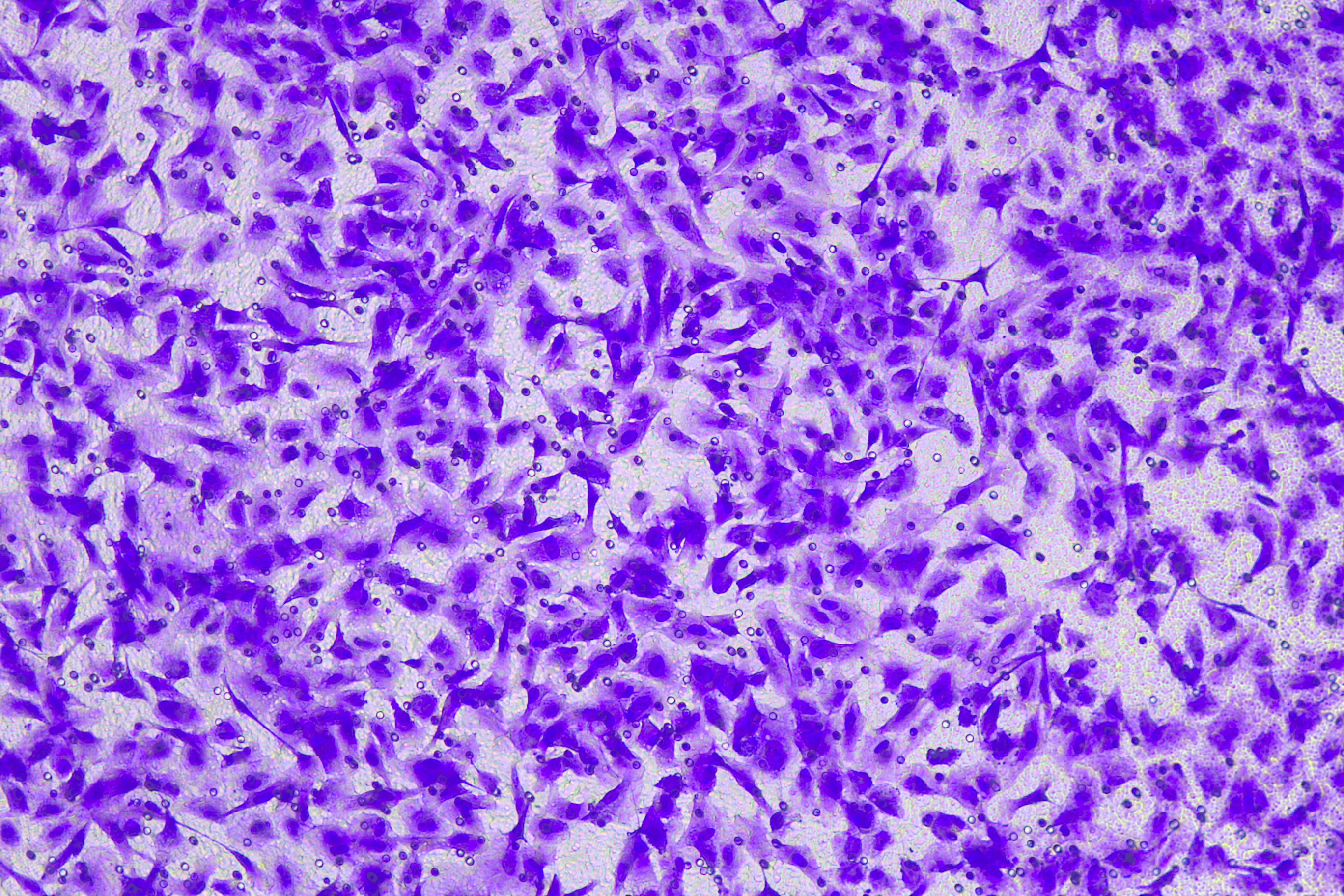

Supplement: Supplementary file 3 — Source Data Fig. 2 [file 44321_2024_25_MOESM3_ESM.zip › figure 2/2L/2L L-FTO.tif]

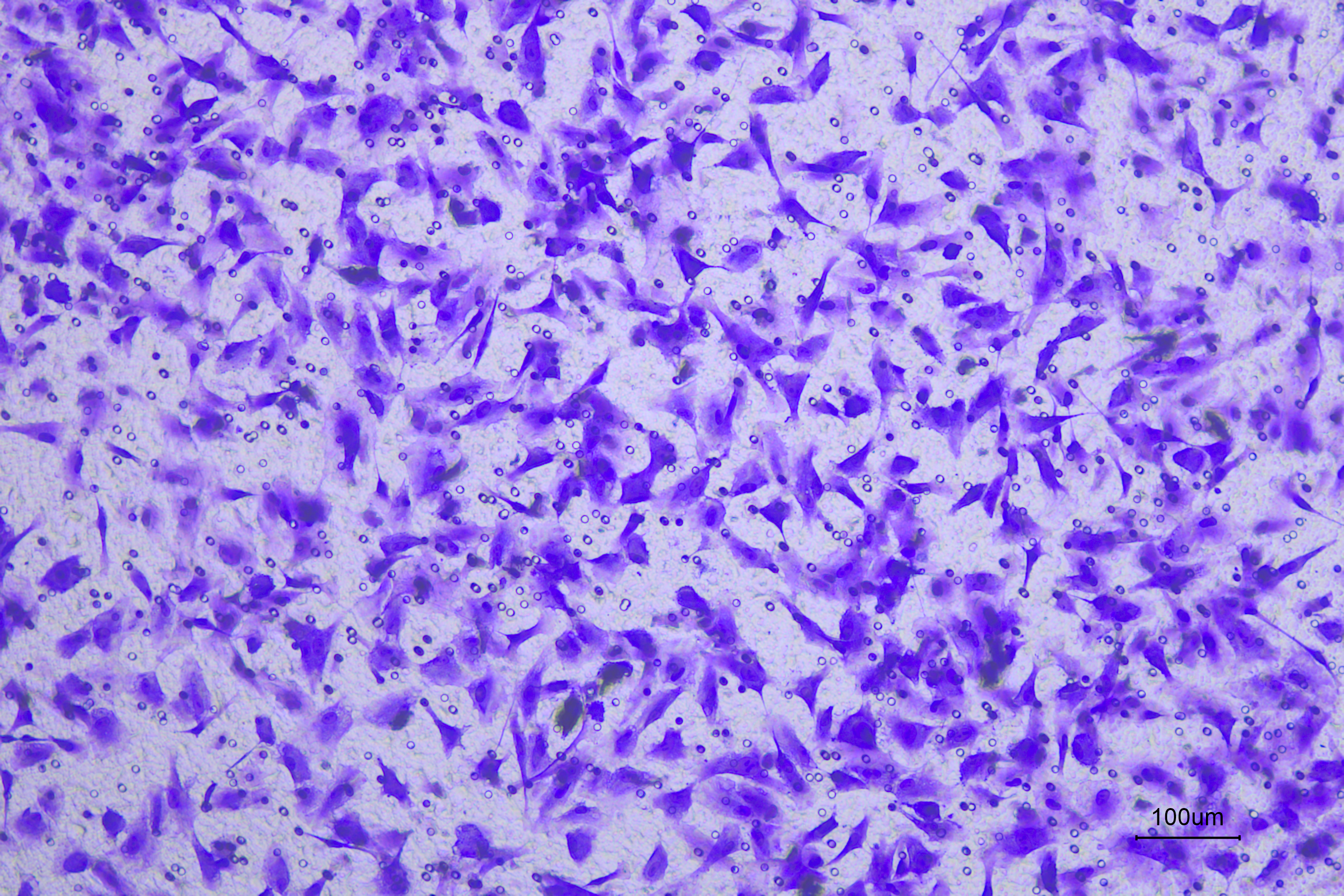

Supplement: Supplementary file 3 — Source Data Fig. 2 [file 44321_2024_25_MOESM3_ESM.zip › figure 2/2M/2M Ctrl.tif]

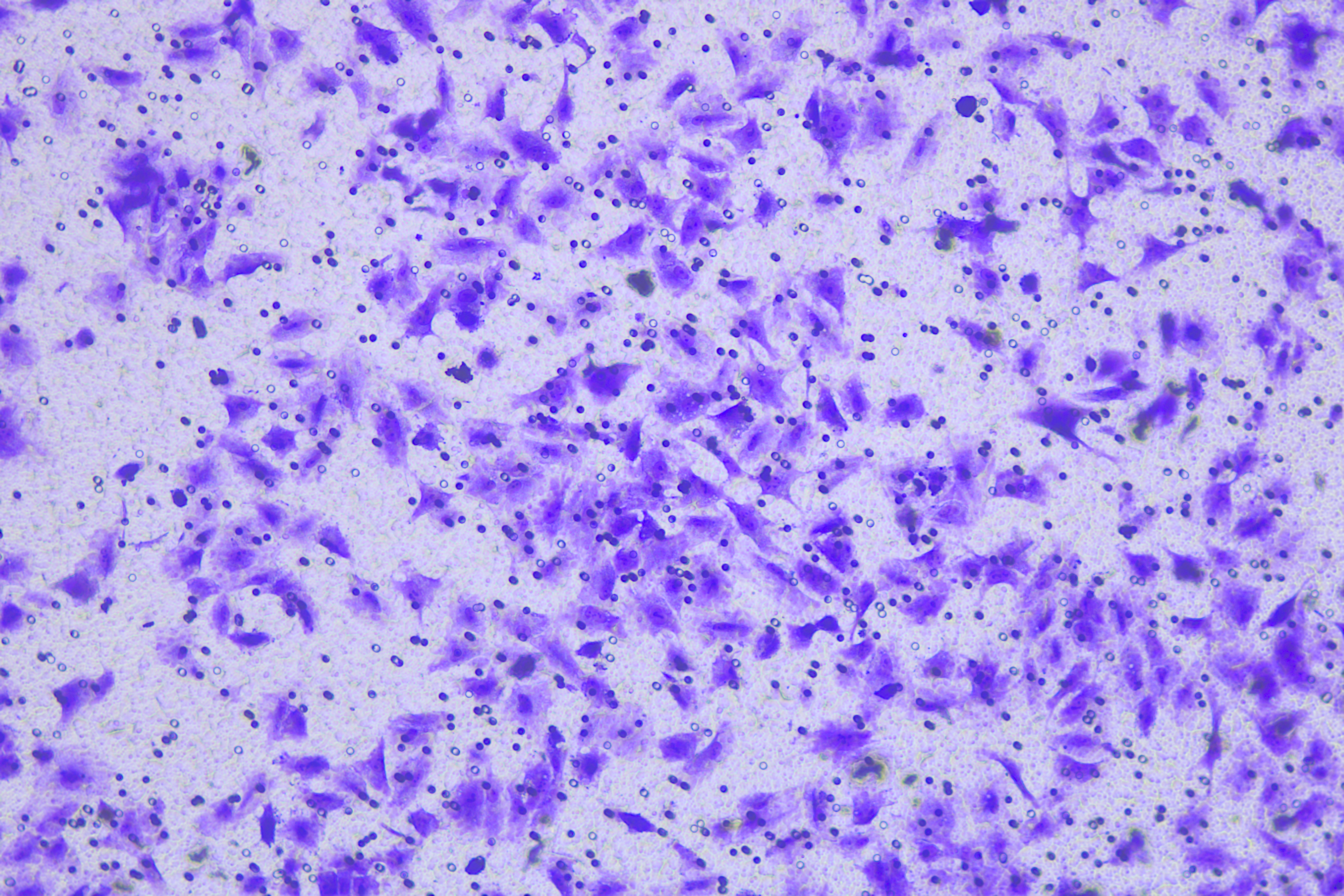

Supplement: Supplementary file 3 — Source Data Fig. 2 [file 44321_2024_25_MOESM3_ESM.zip › figure 2/2M/2M FTO-siRNA.tif]

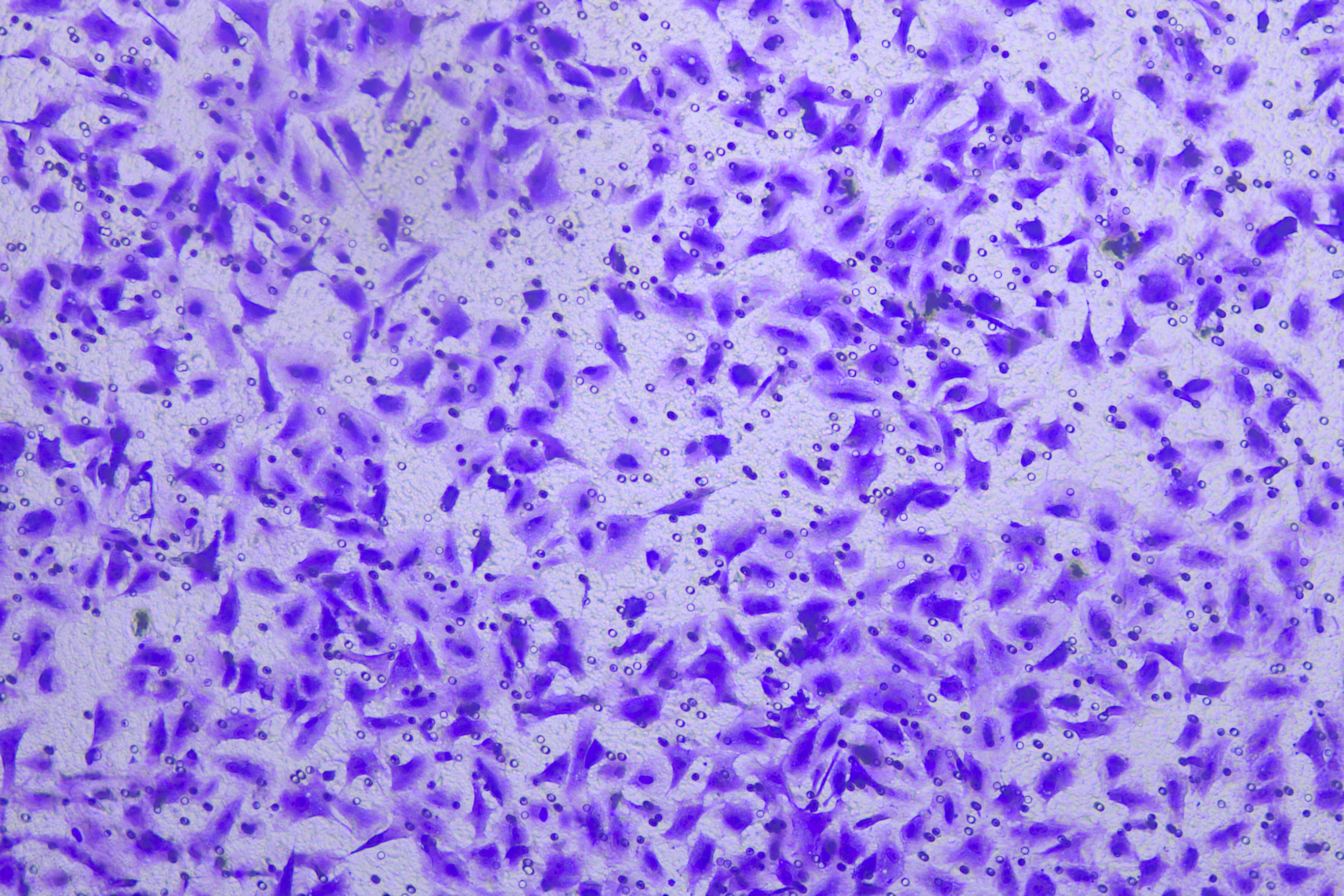

Supplement: Supplementary file 3 — Source Data Fig. 2 [file 44321_2024_25_MOESM3_ESM.zip › figure 2/2M/2M scramble siRNA.tif]

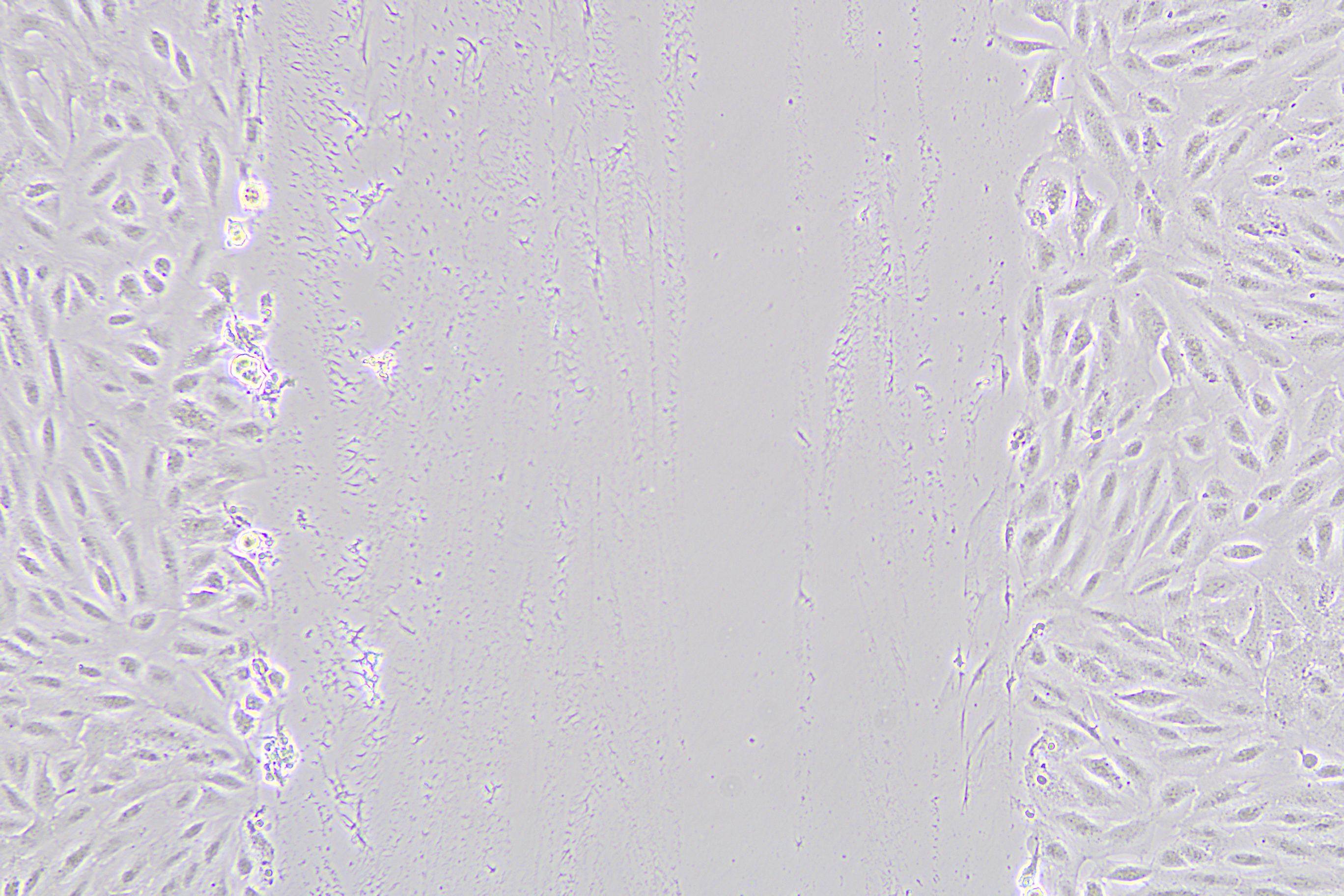

Supplement: Supplementary file 3 — Source Data Fig. 2 [file 44321_2024_25_MOESM3_ESM.zip › figure 2/2N/2N Ctrl 0h.tif]

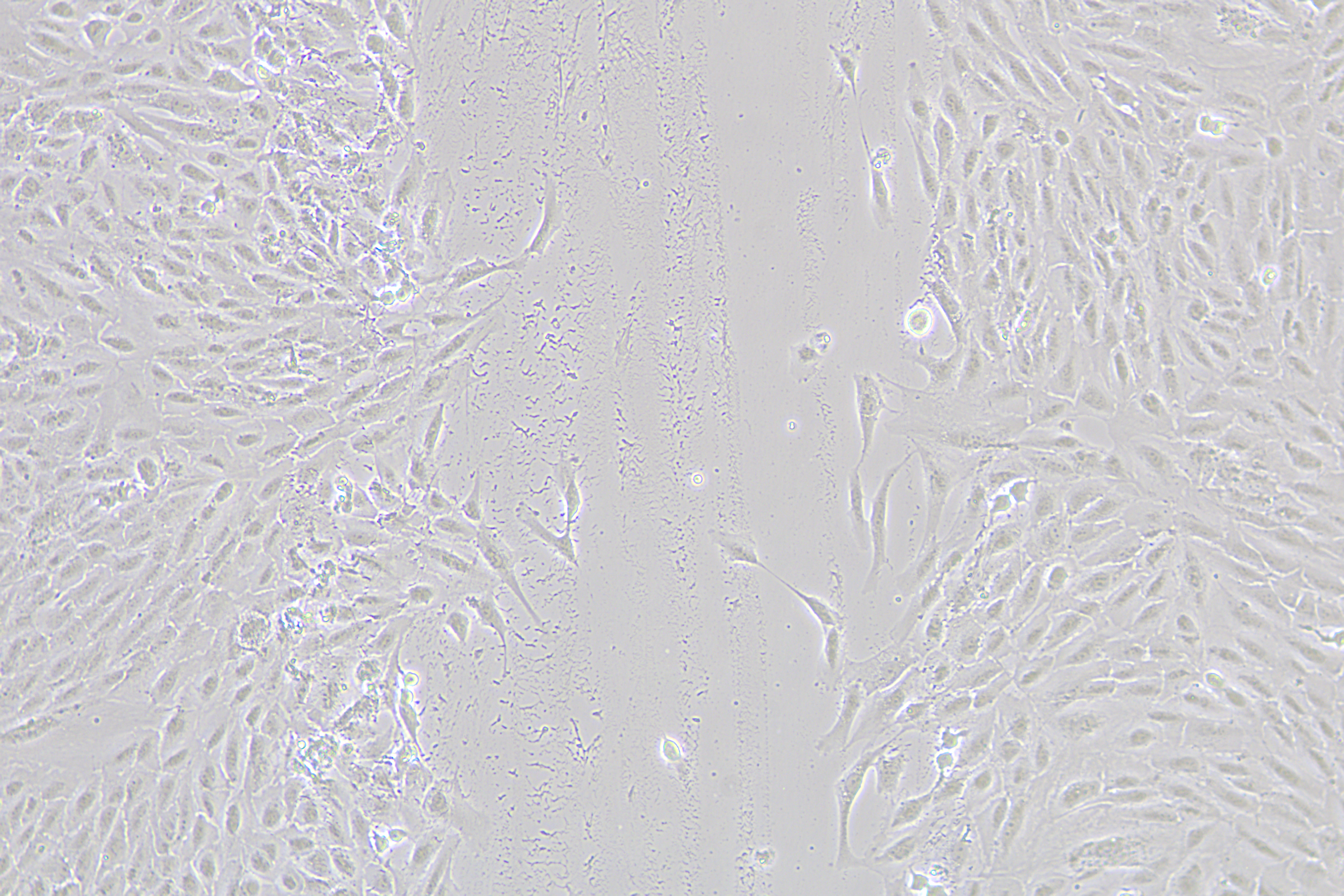

Supplement: Supplementary file 3 — Source Data Fig. 2 [file 44321_2024_25_MOESM3_ESM.zip › figure 2/2N/2N Ctrl 24h.tif]

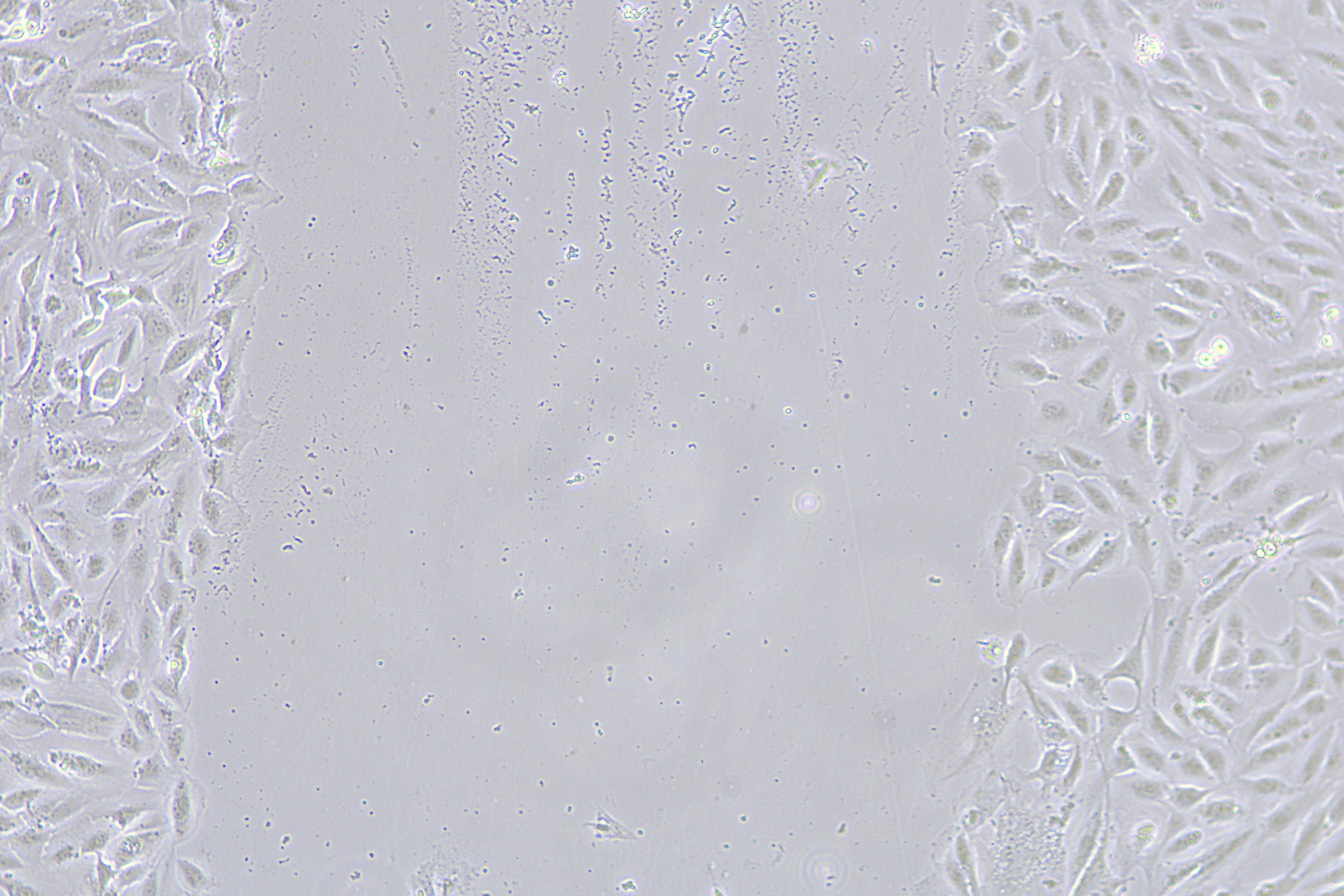

Supplement: Supplementary file 3 — Source Data Fig. 2 [file 44321_2024_25_MOESM3_ESM.zip › figure 2/2N/2N L-EV 0h.tif]

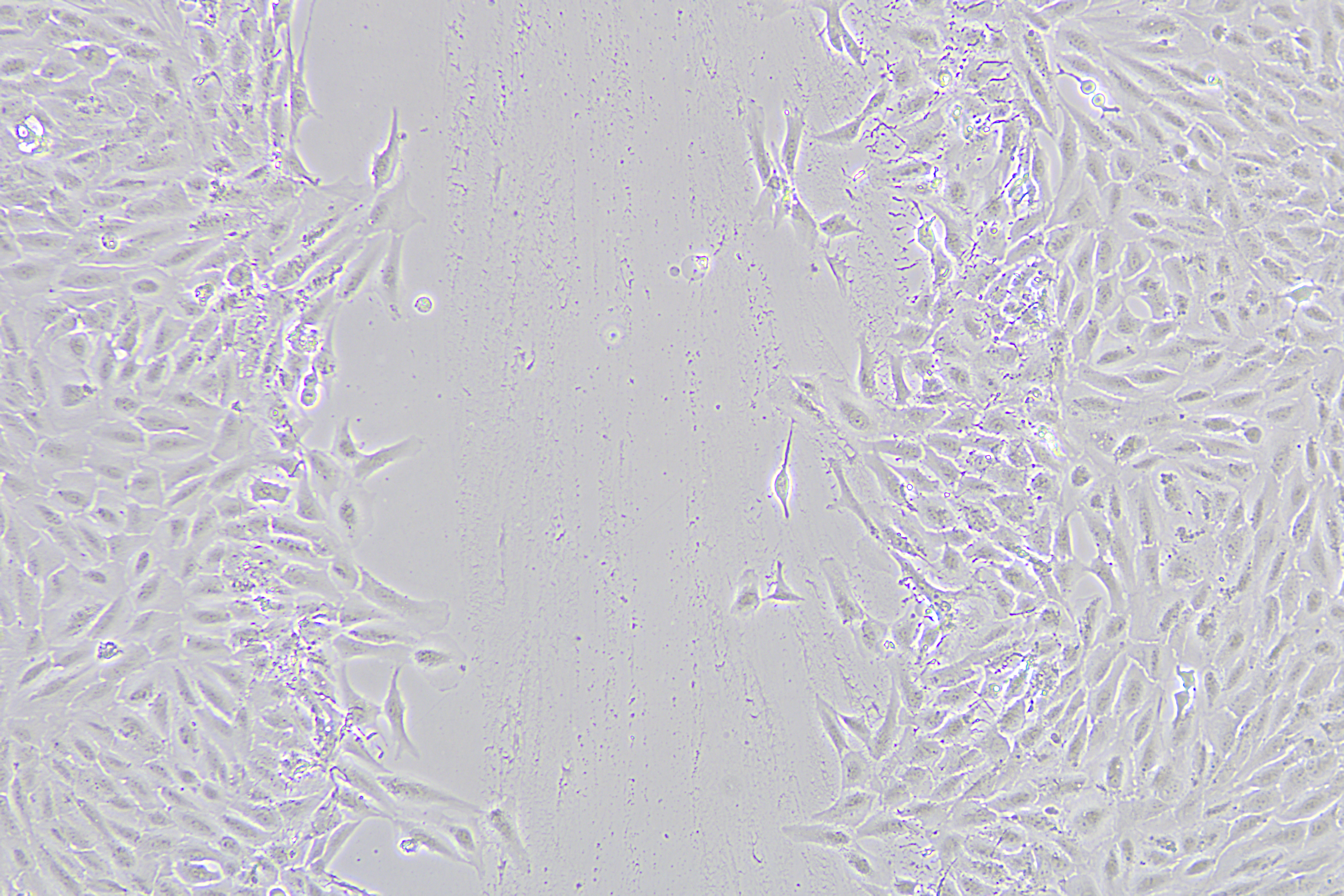

Supplement: Supplementary file 3 — Source Data Fig. 2 [file 44321_2024_25_MOESM3_ESM.zip › figure 2/2N/2N L-EV 24h.tif]

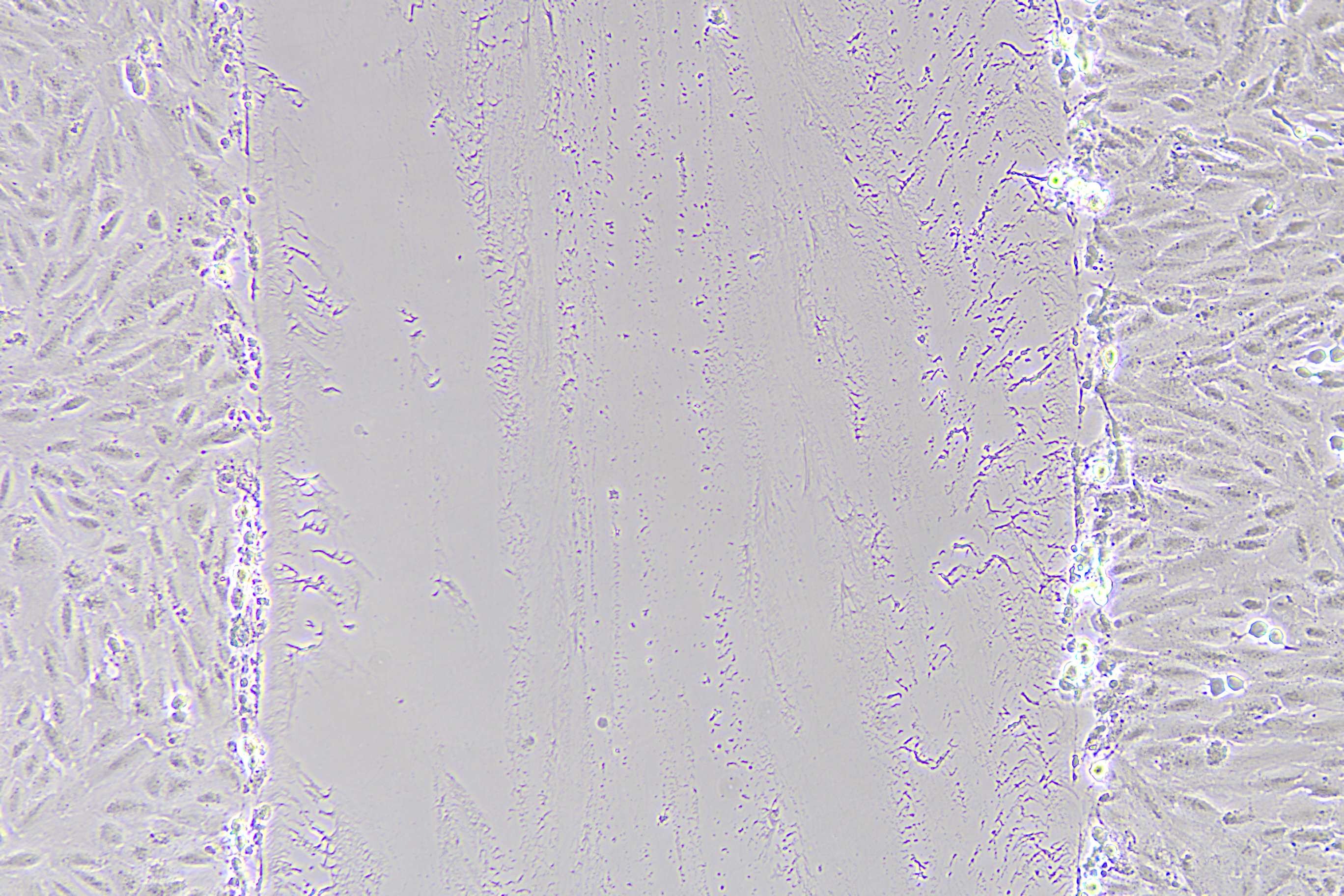

Supplement: Supplementary file 3 — Source Data Fig. 2 [file 44321_2024_25_MOESM3_ESM.zip › figure 2/2N/2N L-FTO 0h.tif]

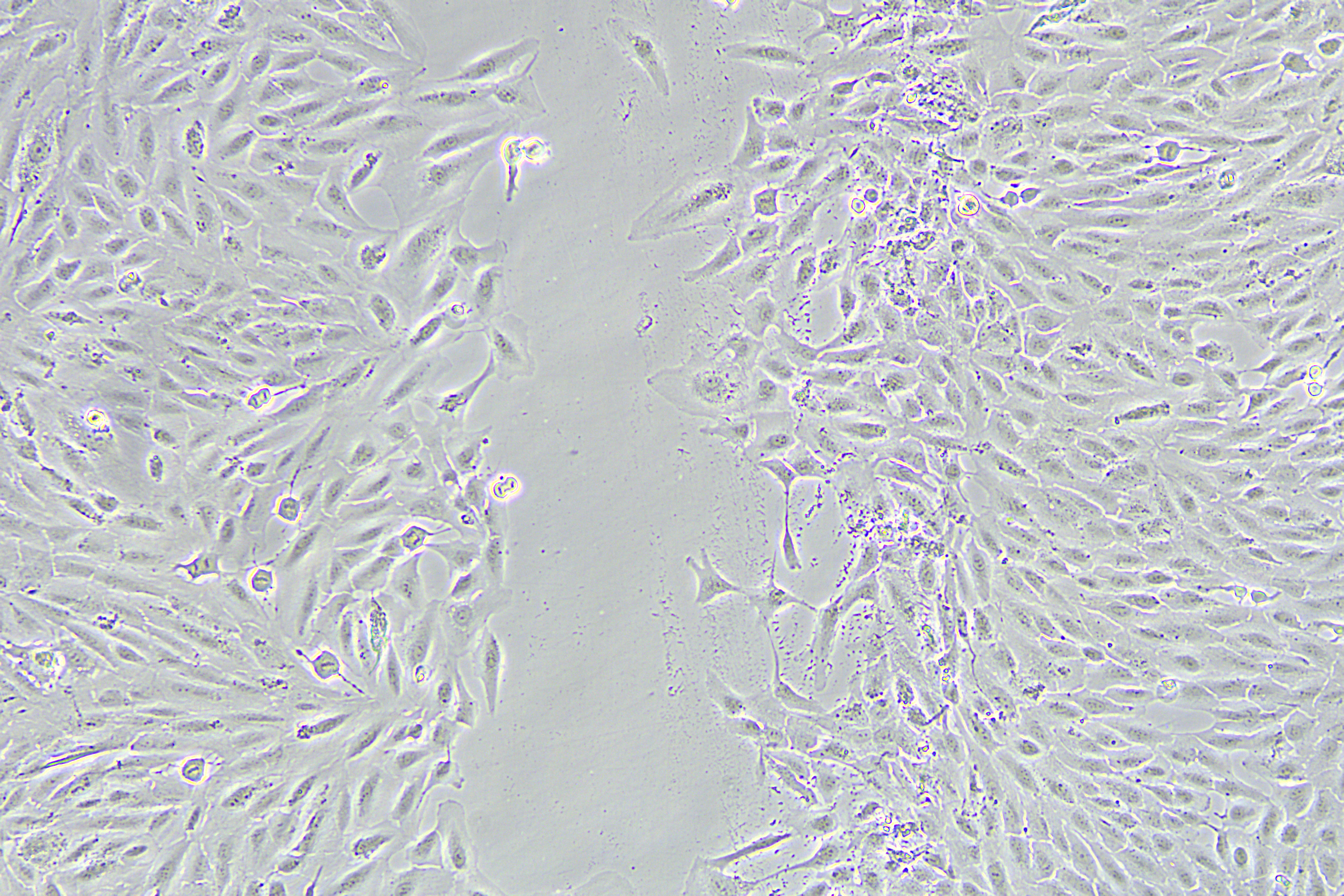

Supplement: Supplementary file 3 — Source Data Fig. 2 [file 44321_2024_25_MOESM3_ESM.zip › figure 2/2N/2N L-FTO 24h.tif]

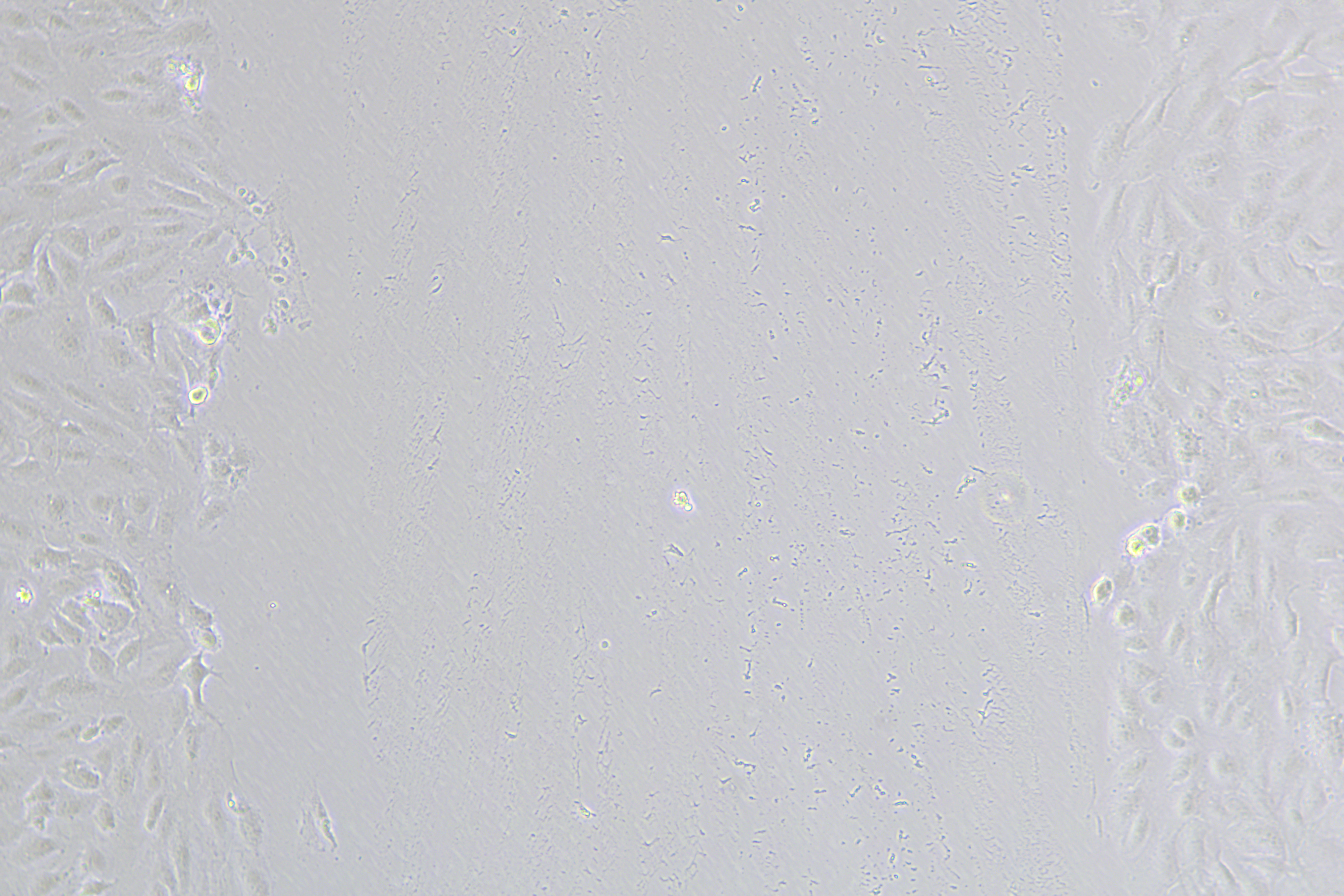

Supplement: Supplementary file 3 — Source Data Fig. 2 [file 44321_2024_25_MOESM3_ESM.zip › figure 2/2O/2O Ctrl 0h.tif]

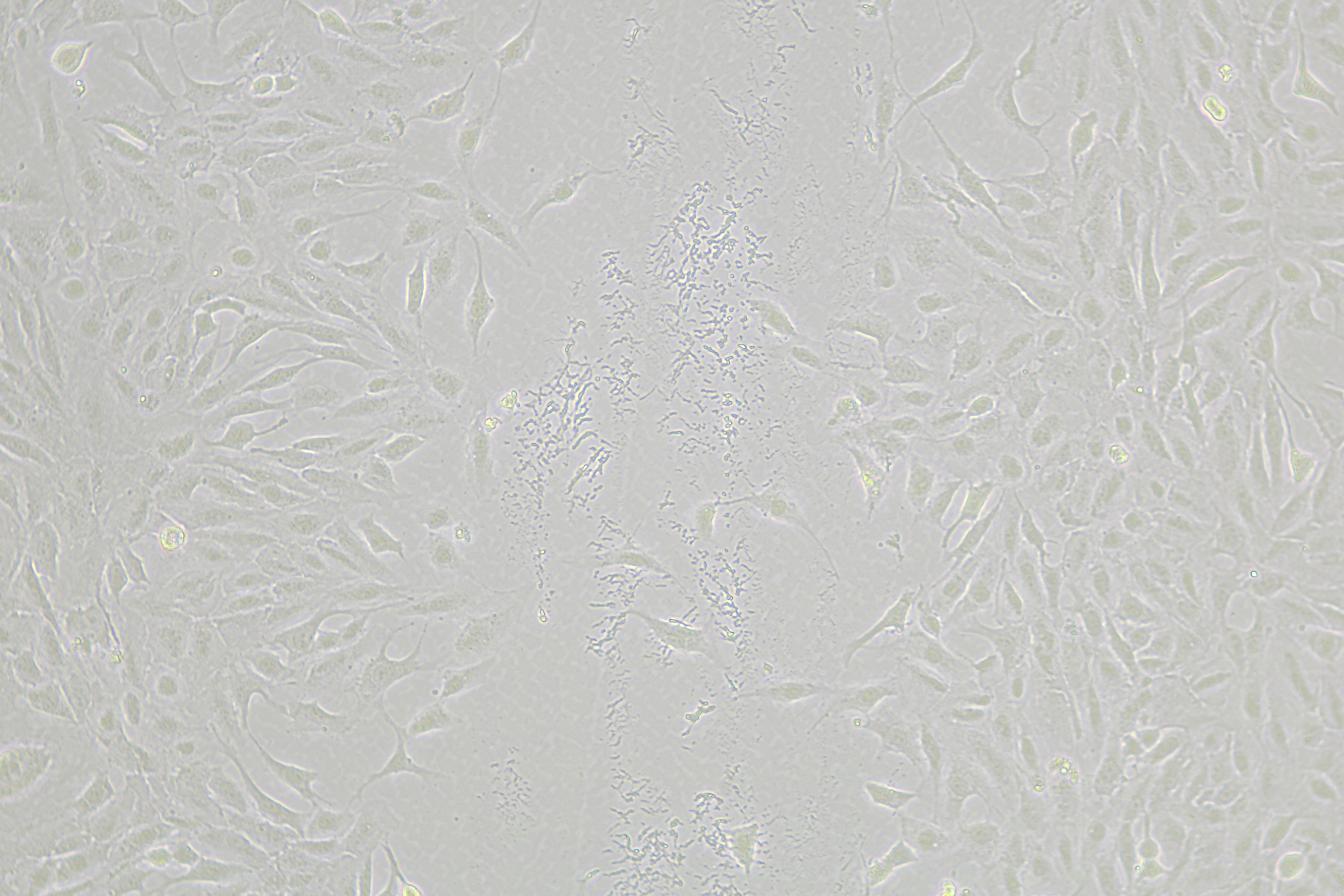

Supplement: Supplementary file 3 — Source Data Fig. 2 [file 44321_2024_25_MOESM3_ESM.zip › figure 2/2O/2O Ctrl 24h.tif]

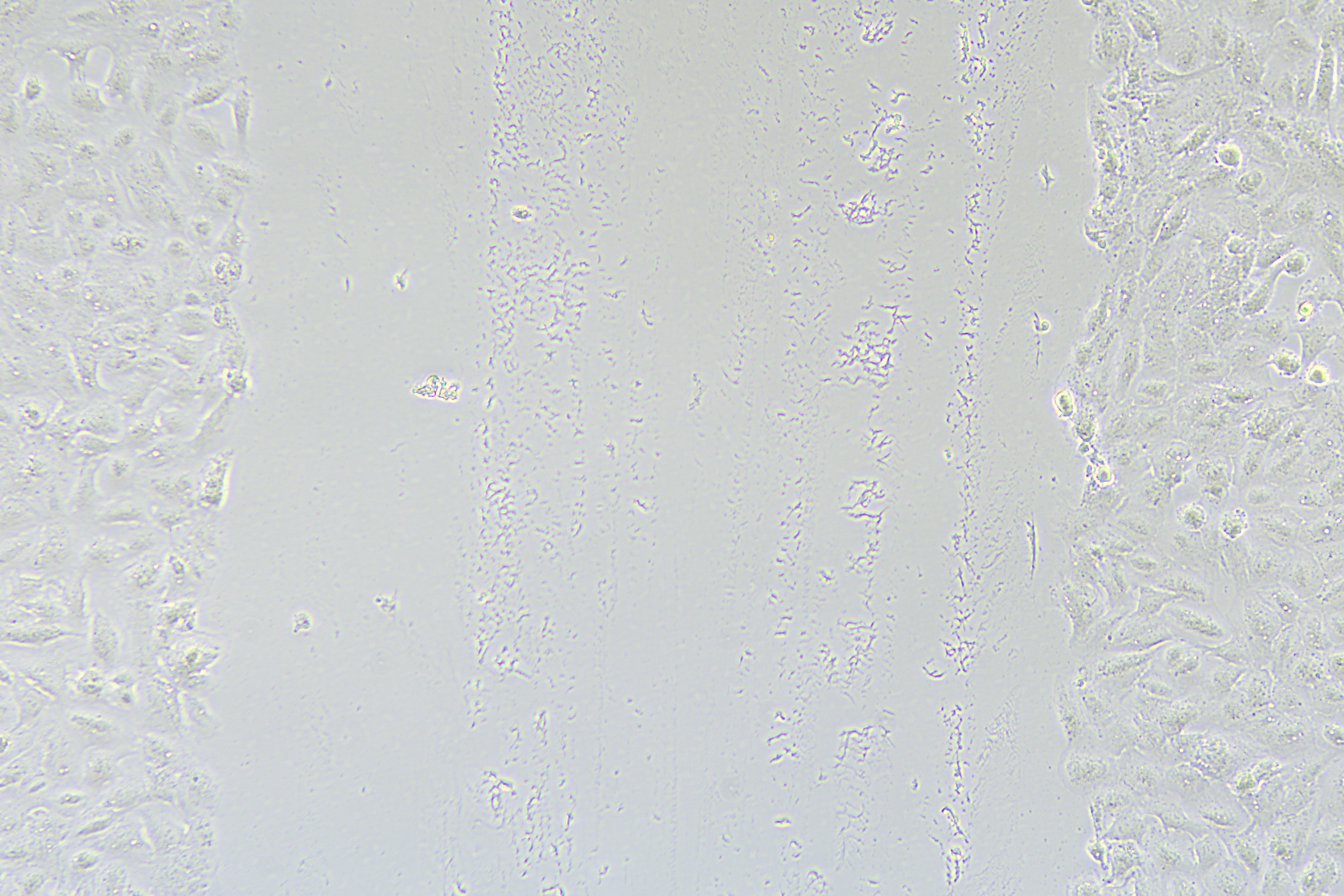

Supplement: Supplementary file 3 — Source Data Fig. 2 [file 44321_2024_25_MOESM3_ESM.zip › figure 2/2O/2O FTO-siRNA 0h.tif]

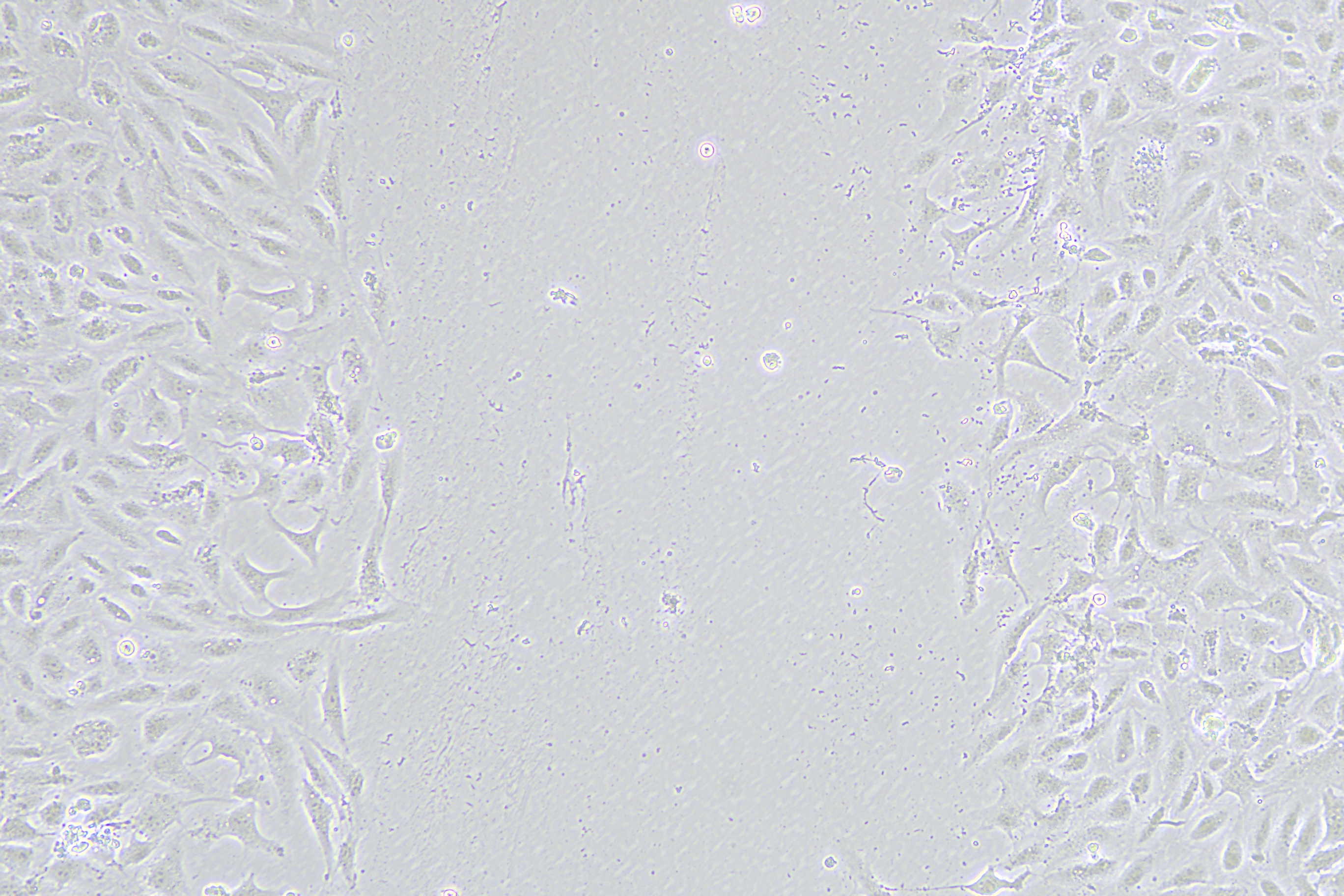

Supplement: Supplementary file 3 — Source Data Fig. 2 [file 44321_2024_25_MOESM3_ESM.zip › figure 2/2O/2O FTO-siRNA 24h.tif]

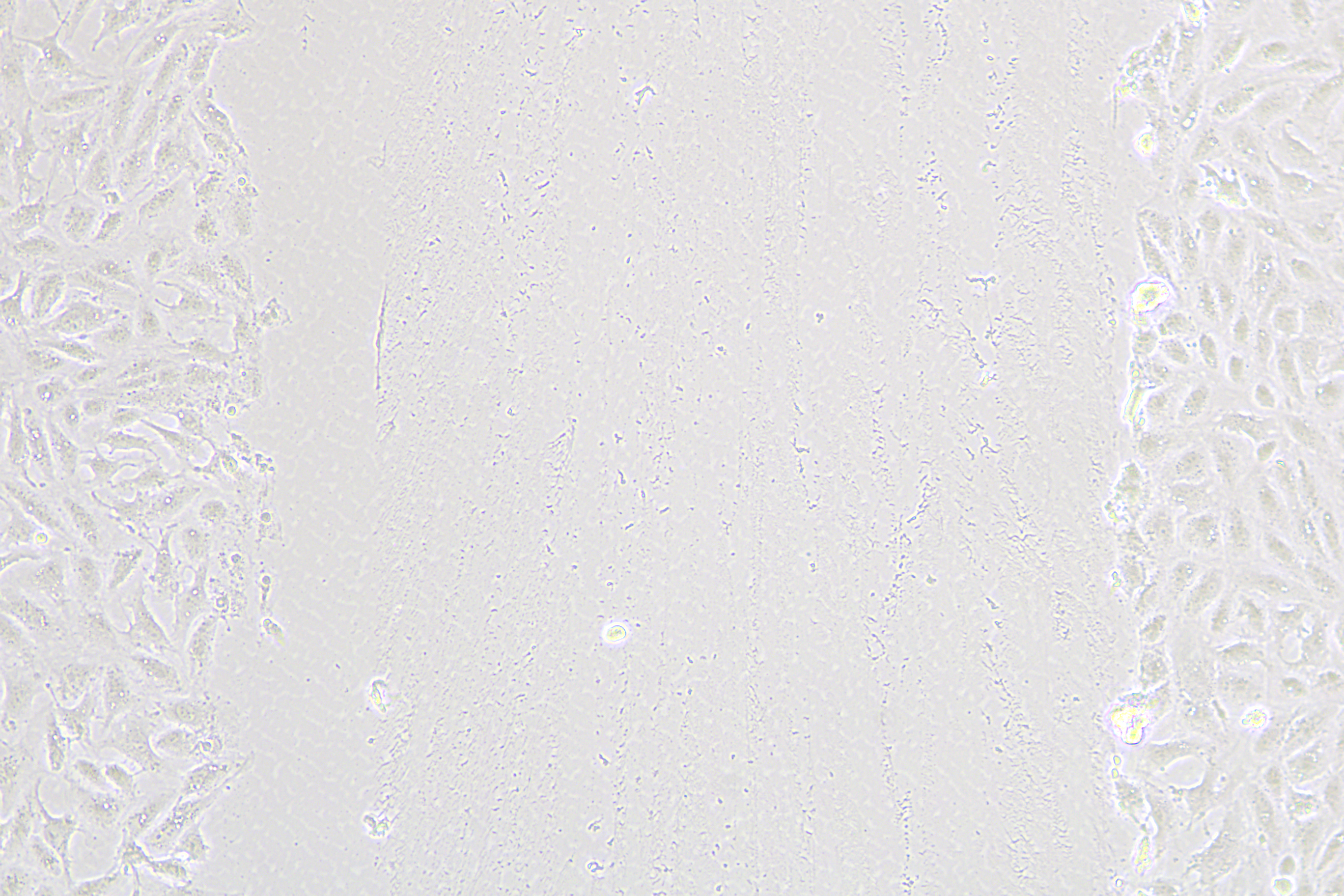

Supplement: Supplementary file 3 — Source Data Fig. 2 [file 44321_2024_25_MOESM3_ESM.zip › figure 2/2O/2O scramble siRNA 0h.tif]

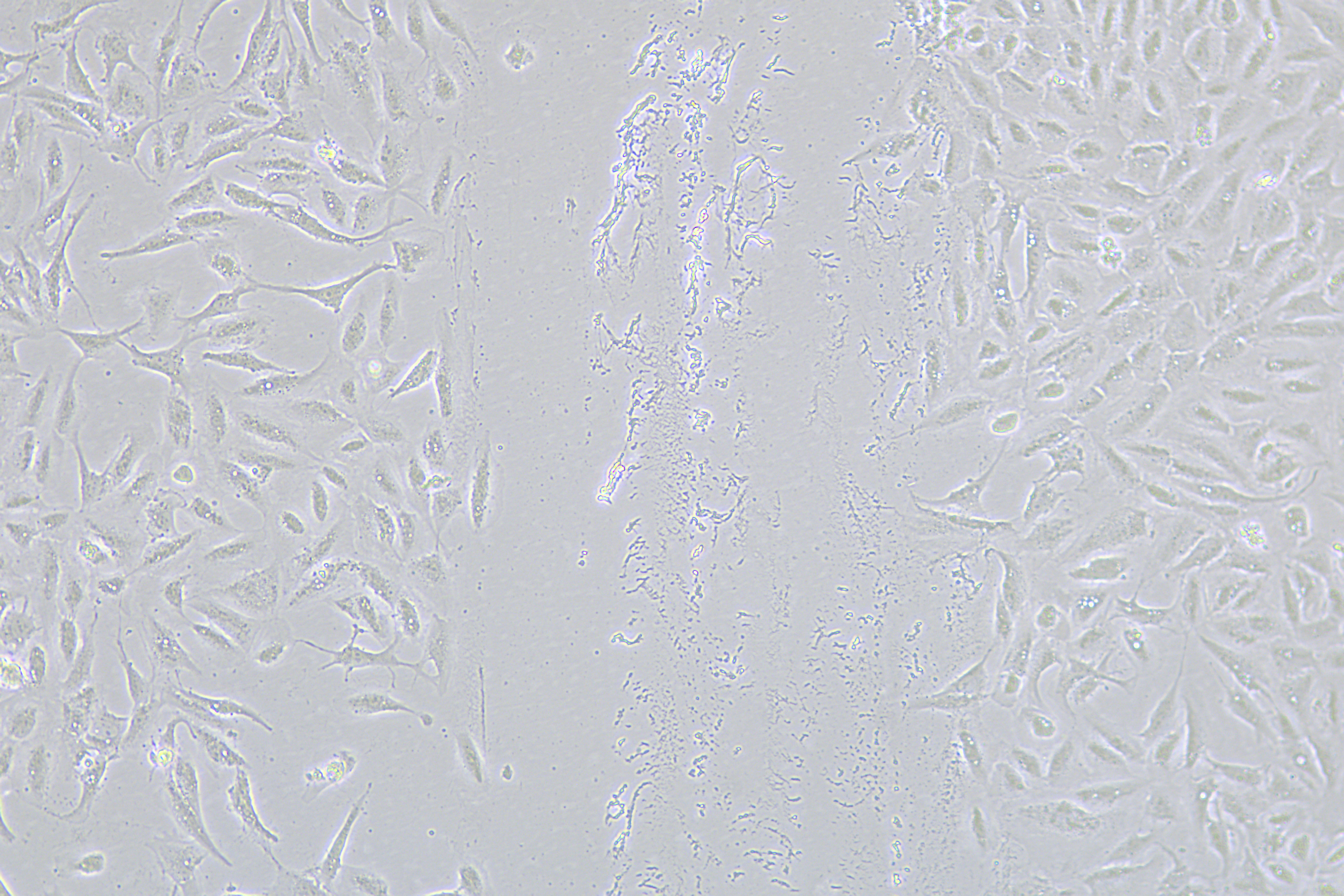

Supplement: Supplementary file 3 — Source Data Fig. 2 [file 44321_2024_25_MOESM3_ESM.zip › figure 2/2O/2O scramble siRNA 24h.tif]

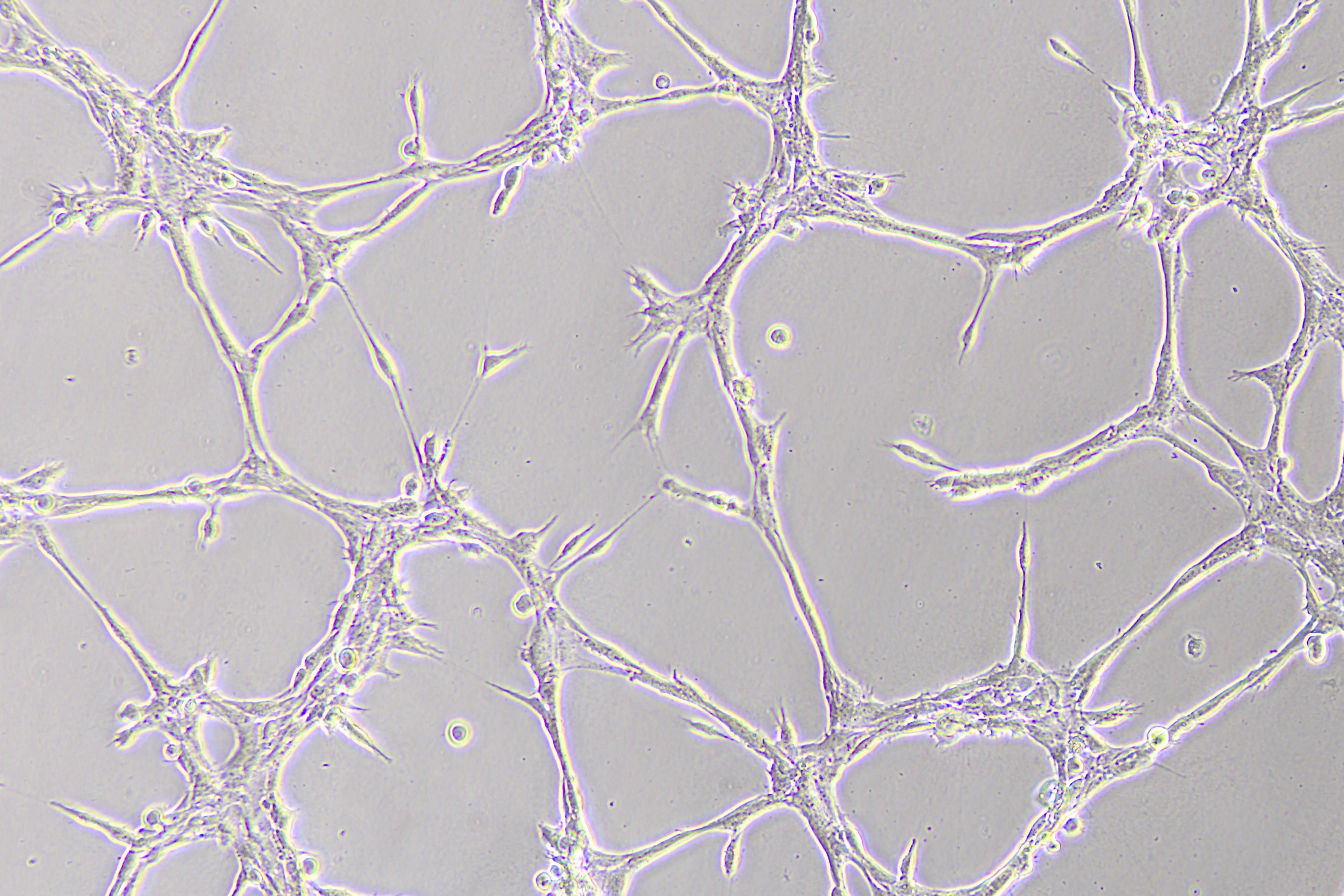

Supplement: Supplementary file 3 — Source Data Fig. 2 [file 44321_2024_25_MOESM3_ESM.zip › figure 2/2R/2R Ctrl.tif]

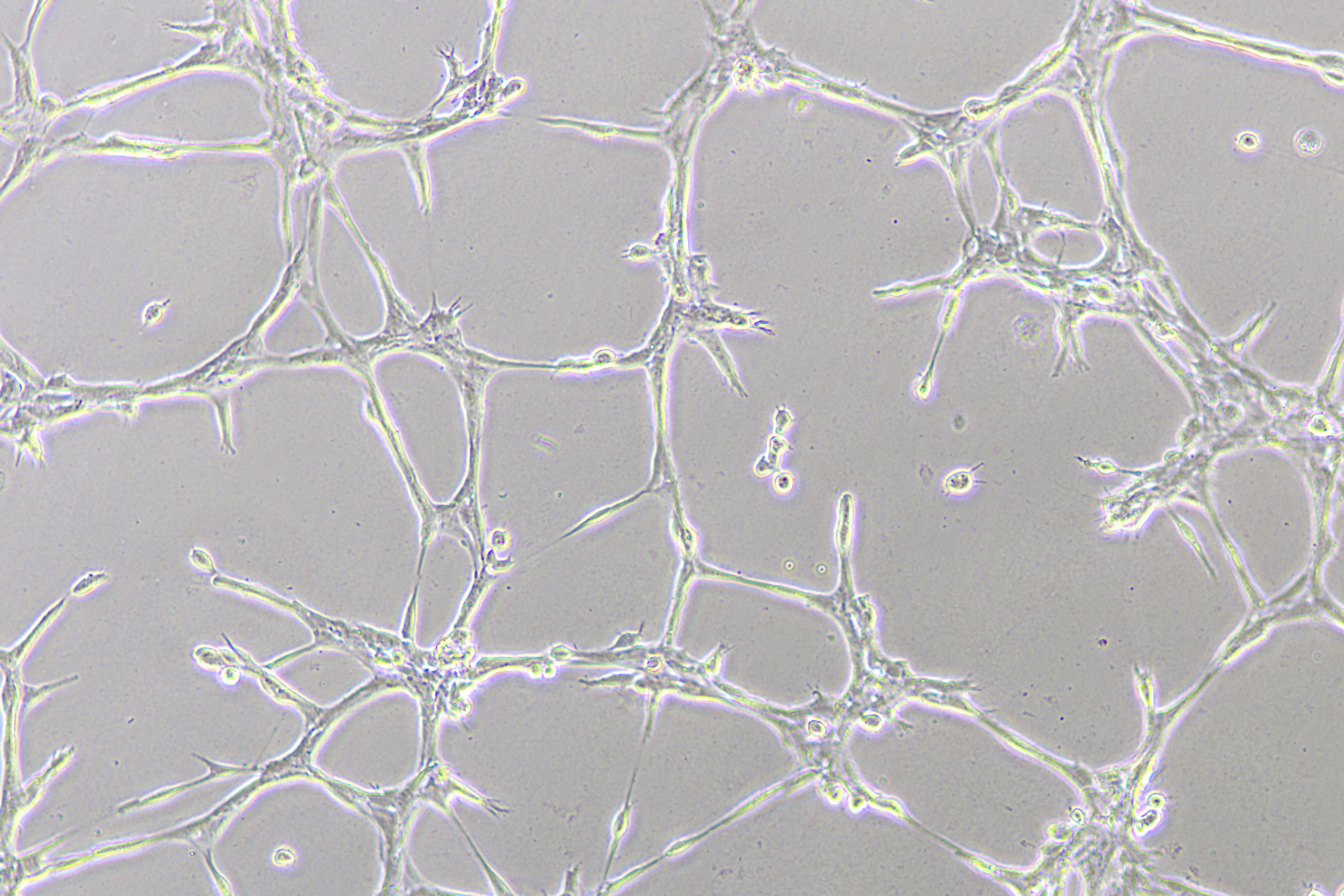

Supplement: Supplementary file 3 — Source Data Fig. 2 [file 44321_2024_25_MOESM3_ESM.zip › figure 2/2R/2R L-EV.tif]

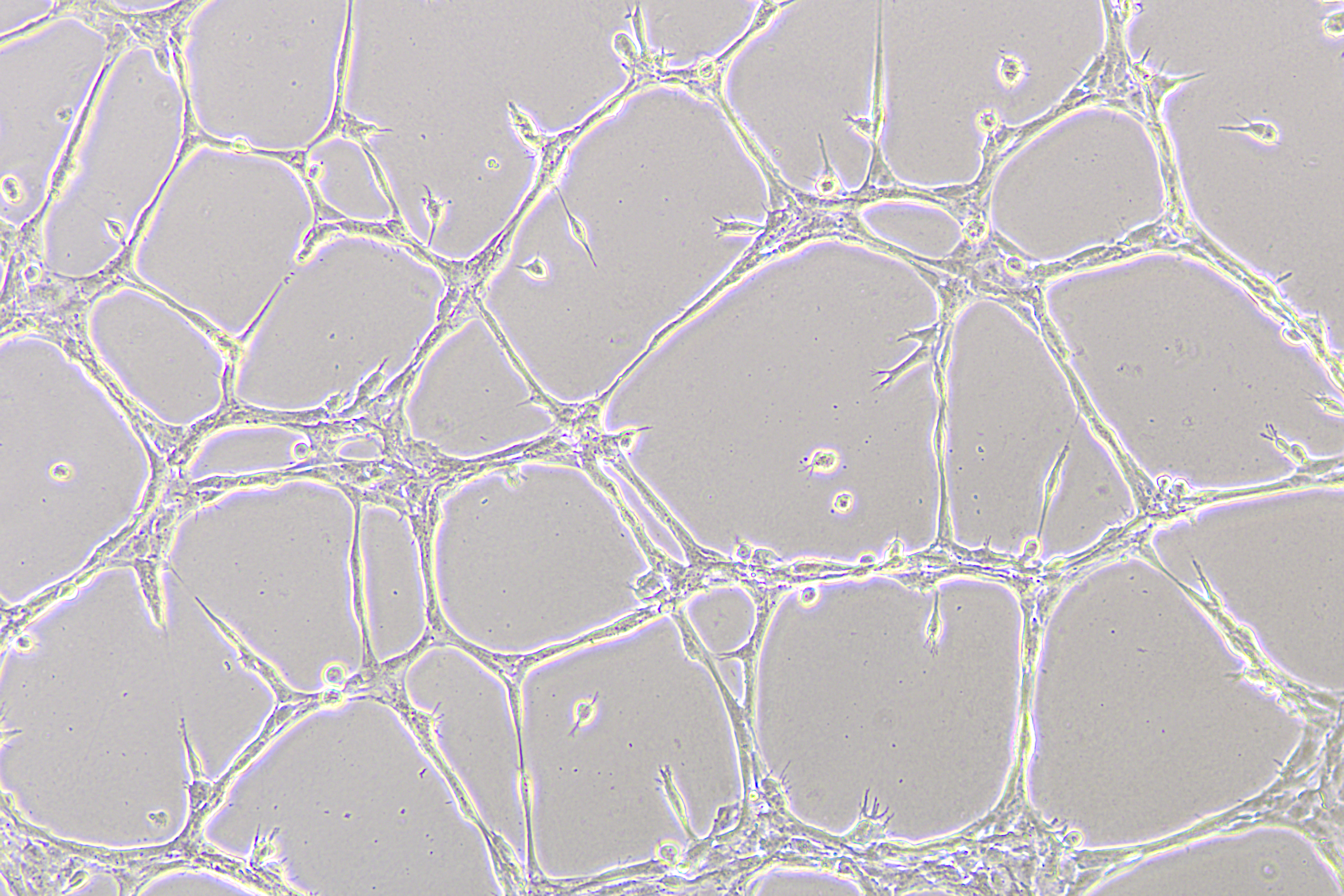

Supplement: Supplementary file 3 — Source Data Fig. 2 [file 44321_2024_25_MOESM3_ESM.zip › figure 2/2R/2R L-FTO.tif]

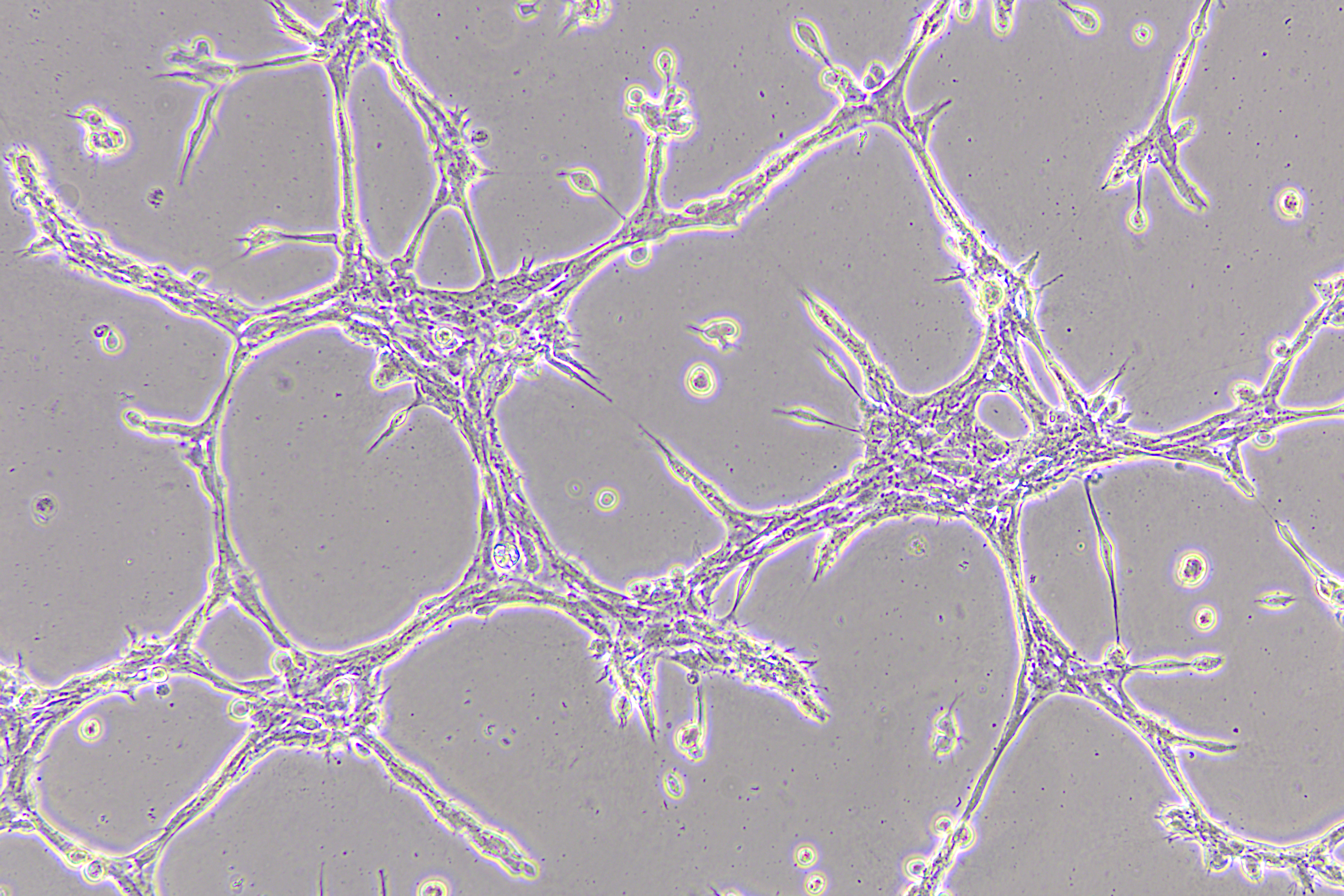

Supplement: Supplementary file 3 — Source Data Fig. 2 [file 44321_2024_25_MOESM3_ESM.zip › figure 2/2S/2S Ctrl.tif]

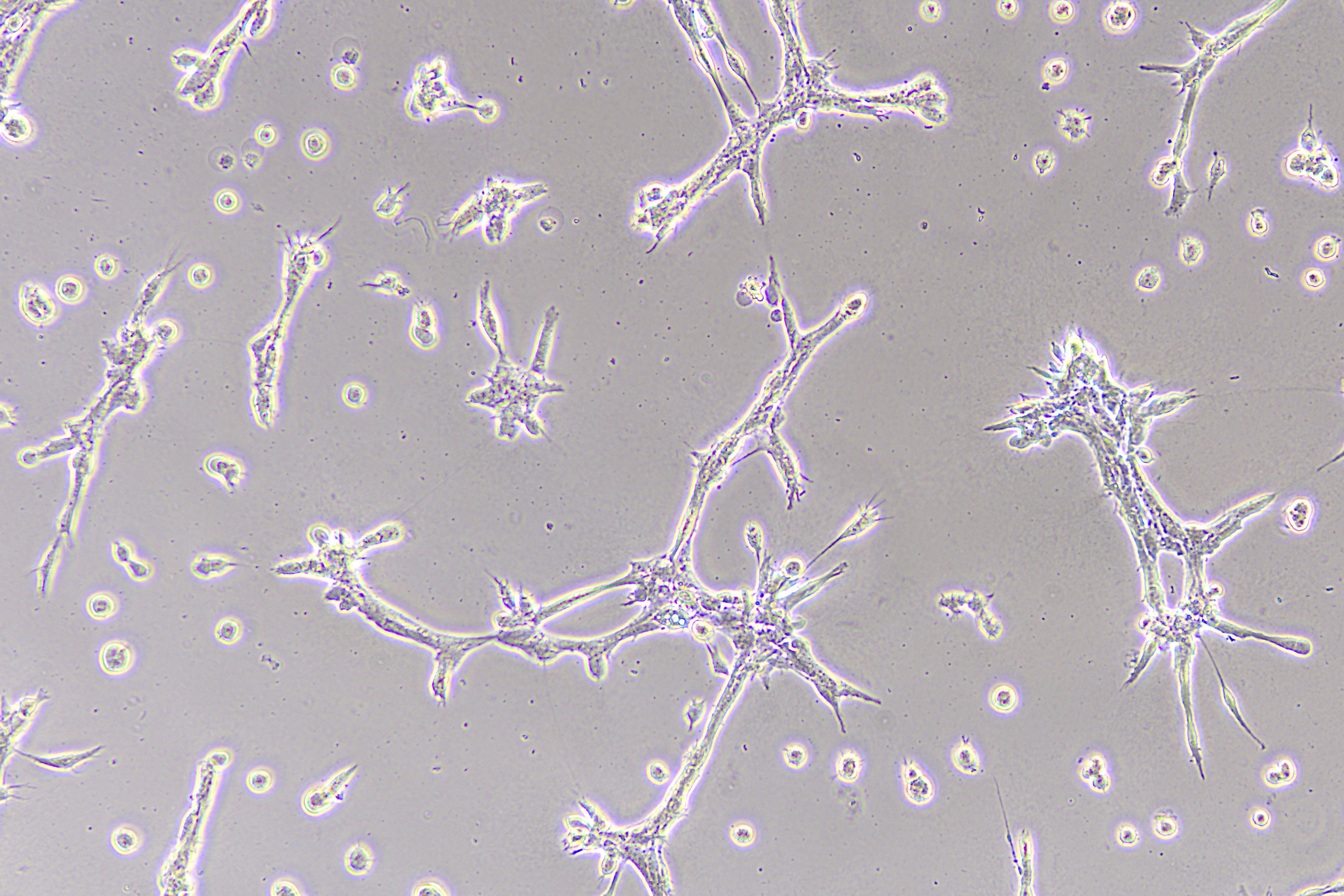

Supplement: Supplementary file 3 — Source Data Fig. 2 [file 44321_2024_25_MOESM3_ESM.zip › figure 2/2S/2S FTO-siRNA.tif]

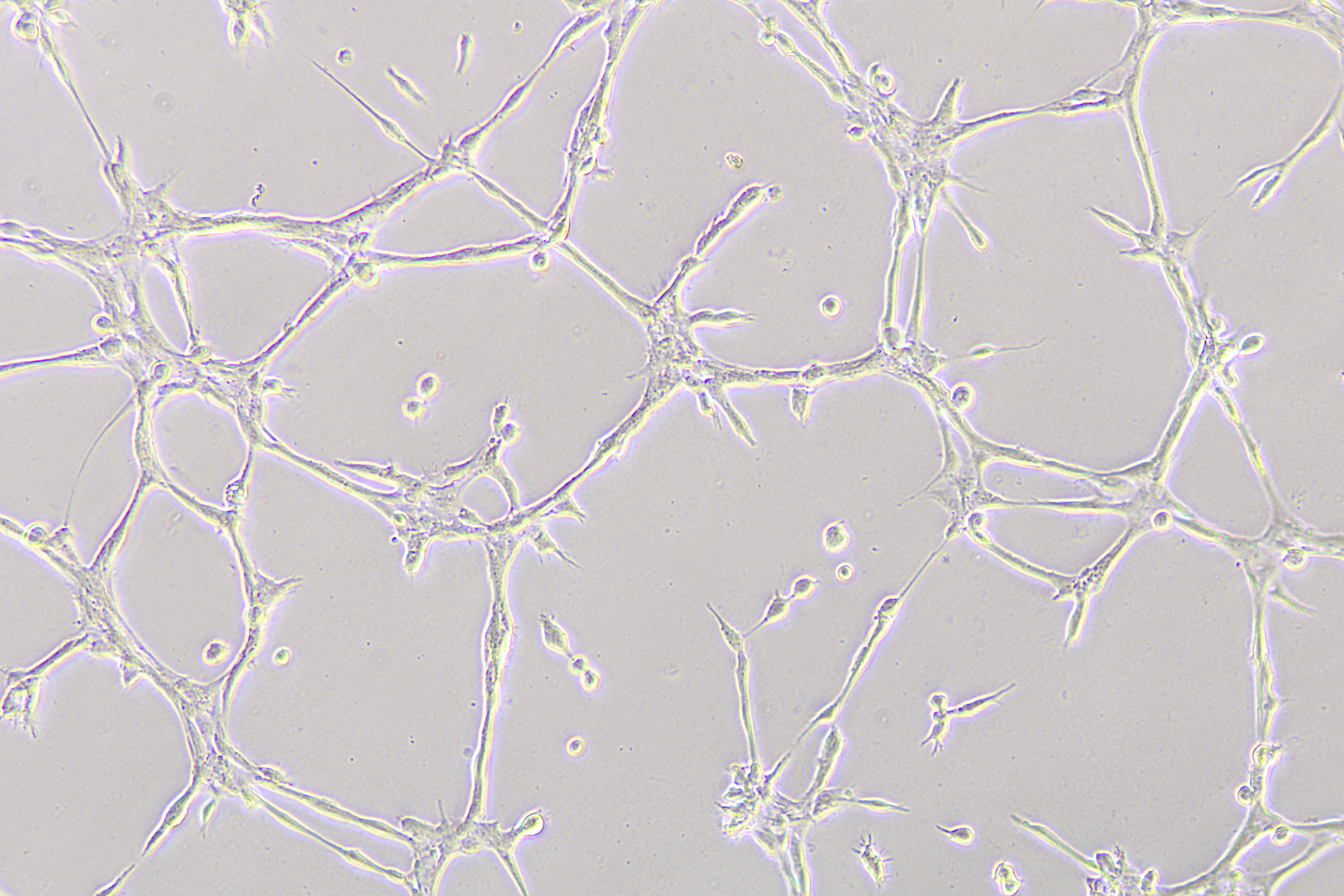

Supplement: Supplementary file 3 — Source Data Fig. 2 [file 44321_2024_25_MOESM3_ESM.zip › figure 2/2S/2S scramble siRNA.tif]

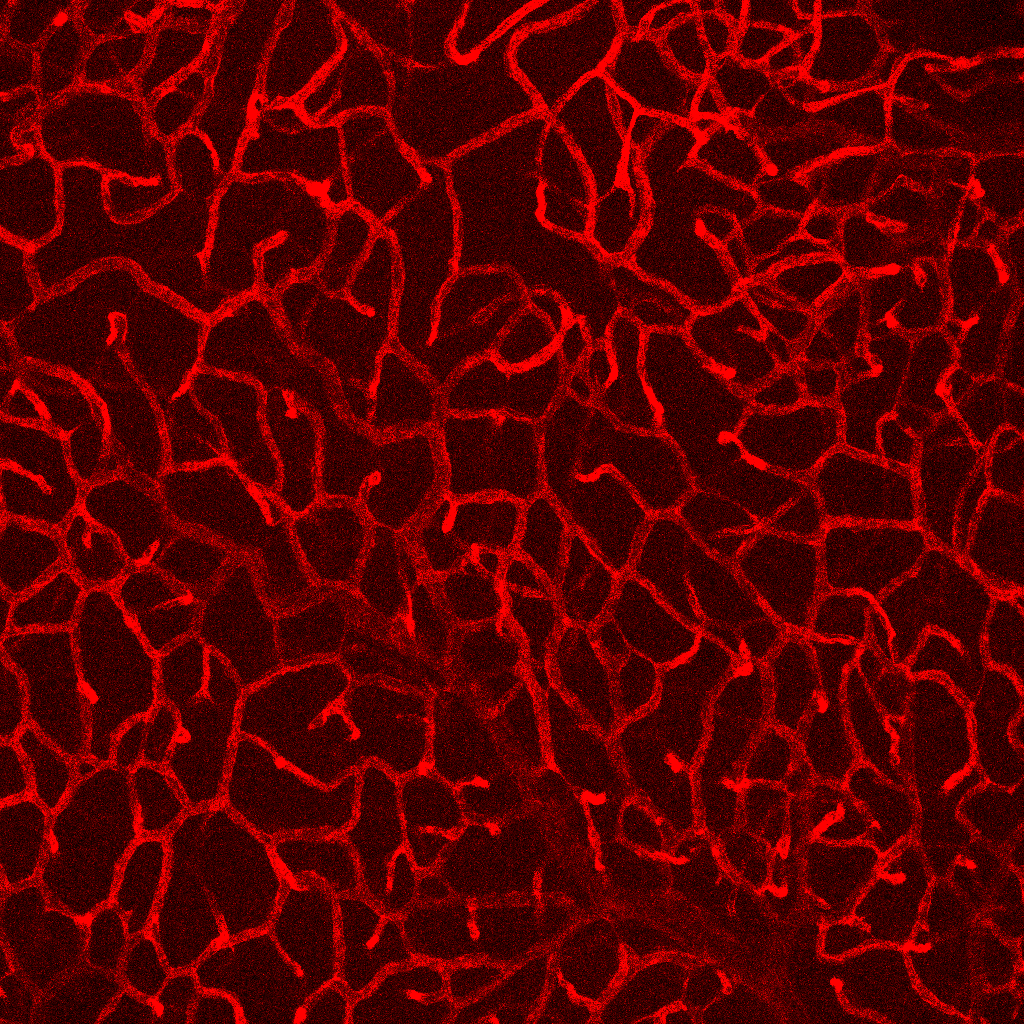

Supplement: Supplementary file 4 — Source Data Fig. 3 [file 44321_2024_25_MOESM4_ESM.zip › figure 3/3B/3B IB4 Ctrl deep.tif]

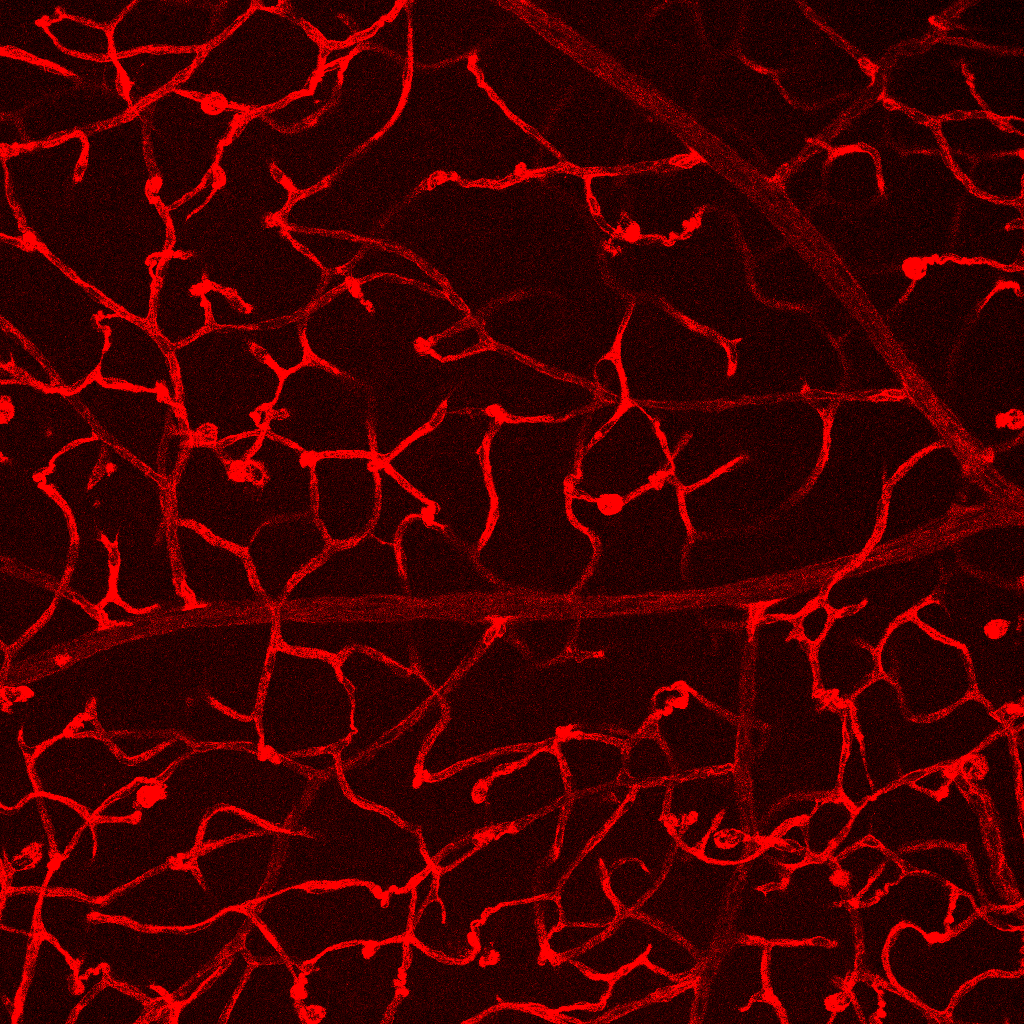

Supplement: Supplementary file 4 — Source Data Fig. 3 [file 44321_2024_25_MOESM4_ESM.zip › figure 3/3B/3B IB4 Ctrl superficial.tif]

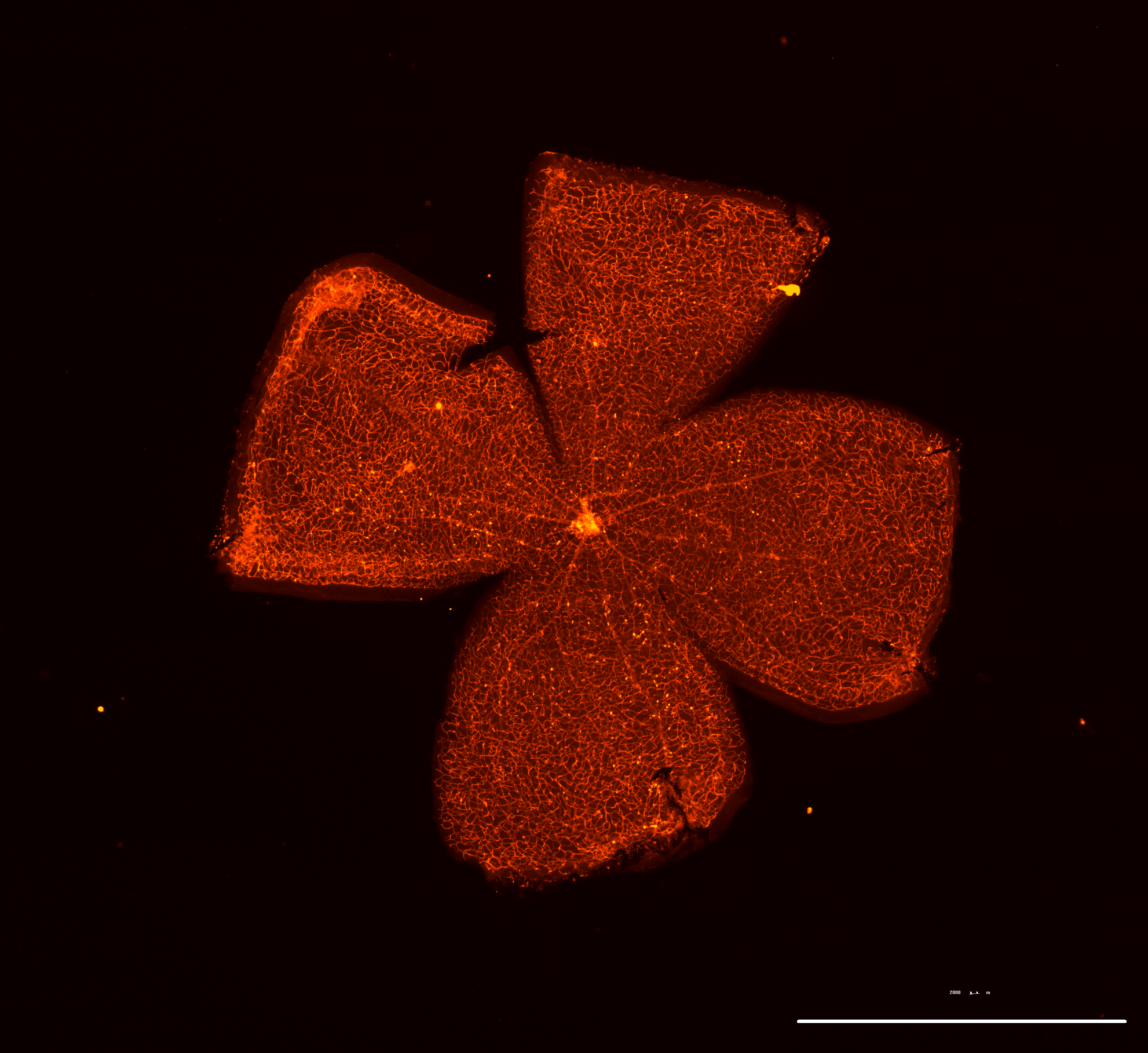

Supplement: Supplementary file 4 — Source Data Fig. 3 [file 44321_2024_25_MOESM4_ESM.zip › figure 3/3B/3B IB4 Ctrl upper line.tif]

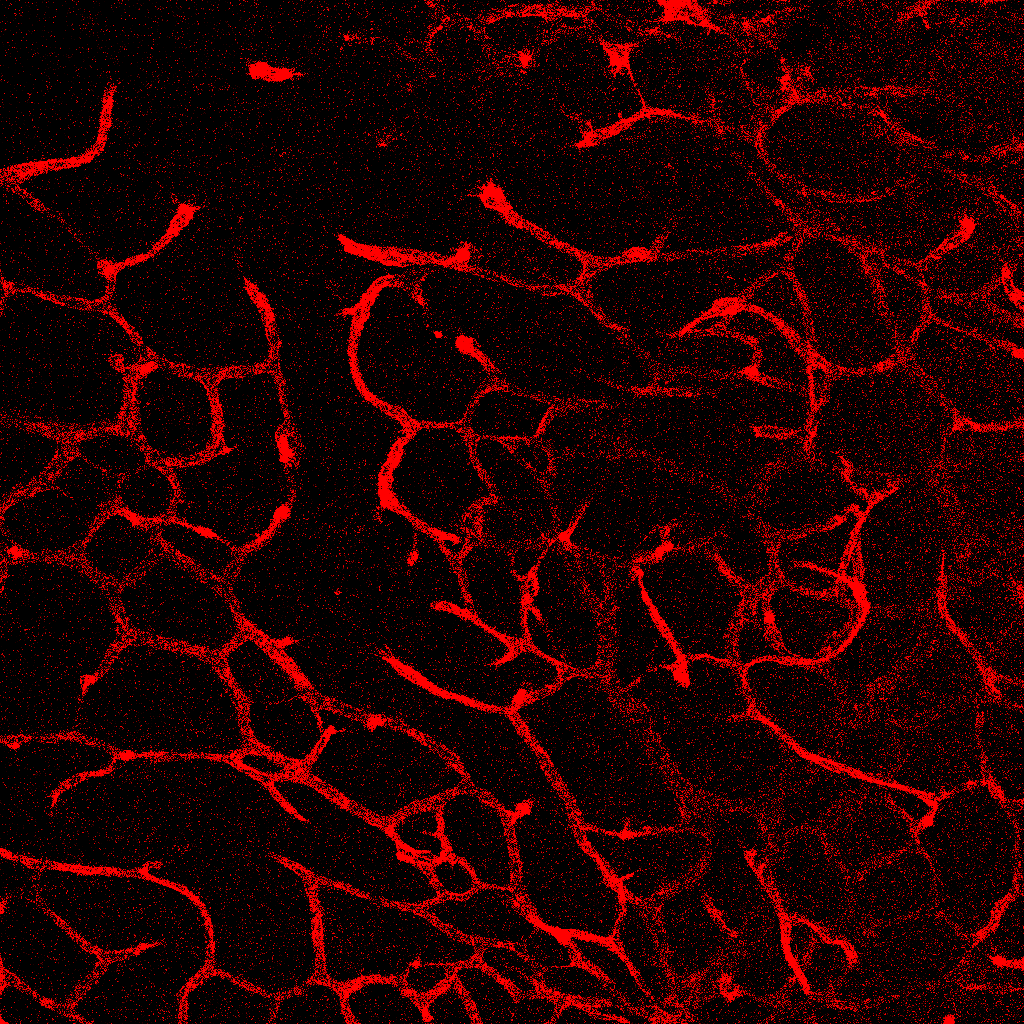

Supplement: Supplementary file 4 — Source Data Fig. 3 [file 44321_2024_25_MOESM4_ESM.zip › figure 3/3B/3B IB4 OIR deep.tif]

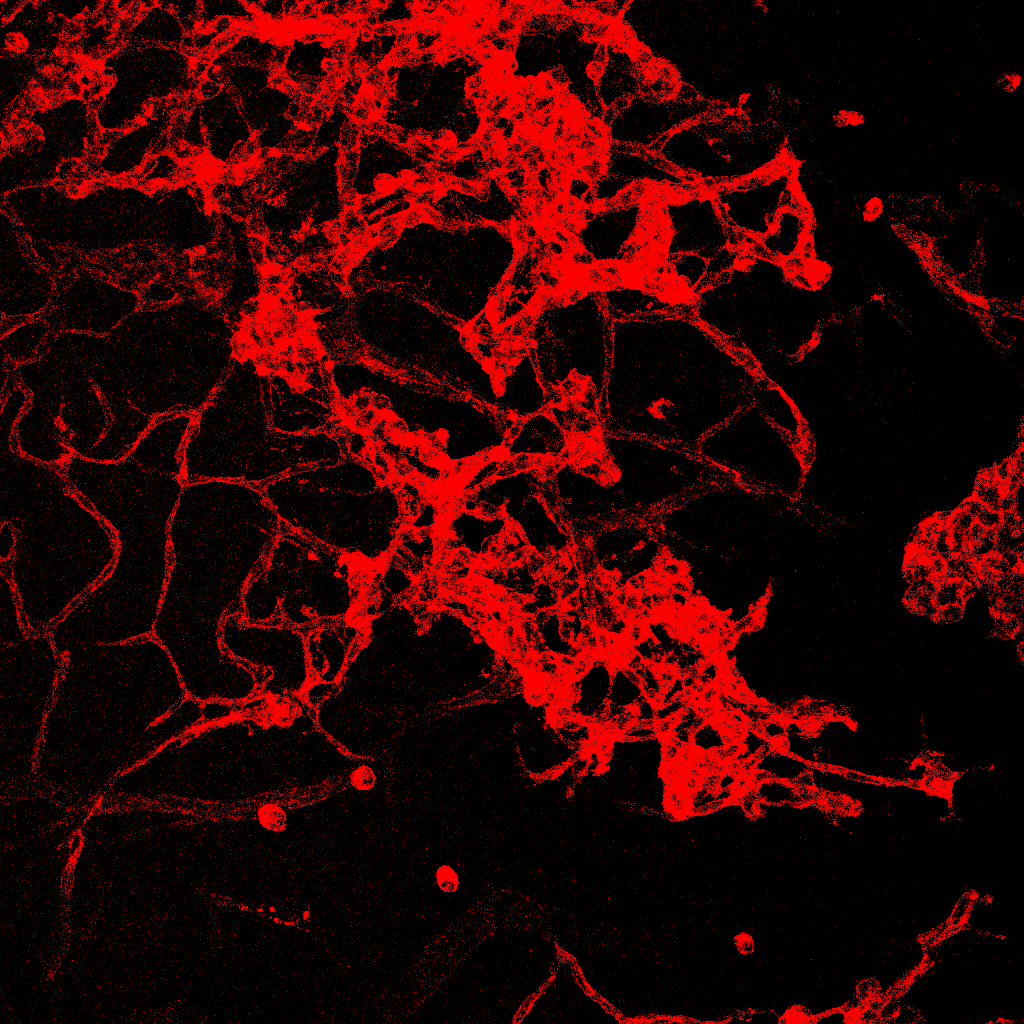

Supplement: Supplementary file 4 — Source Data Fig. 3 [file 44321_2024_25_MOESM4_ESM.zip › figure 3/3B/3B IB4 OIR superficial.tif]

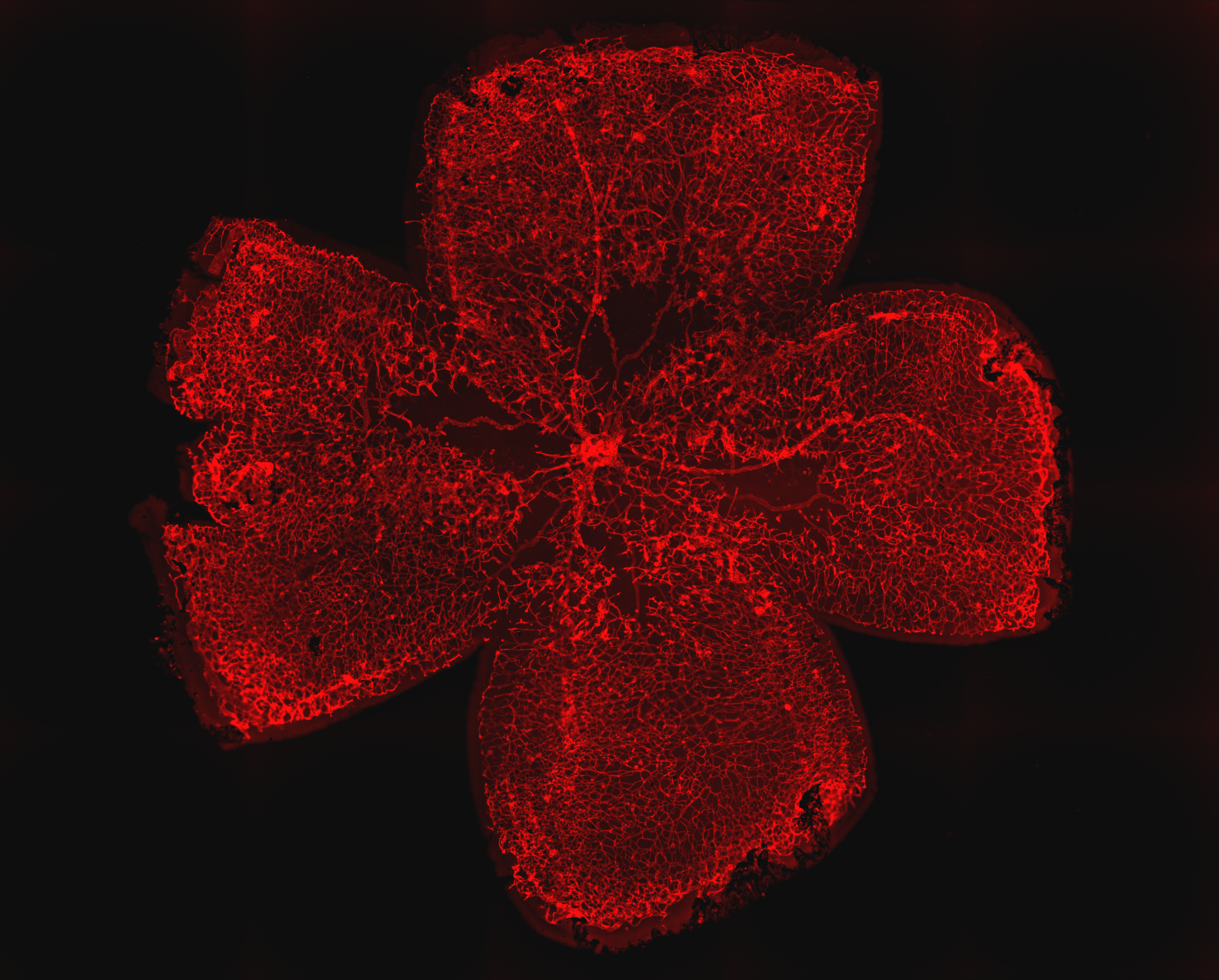

Supplement: Supplementary file 4 — Source Data Fig. 3 [file 44321_2024_25_MOESM4_ESM.zip › figure 3/3B/3B IB4 OIR upper line.tif]

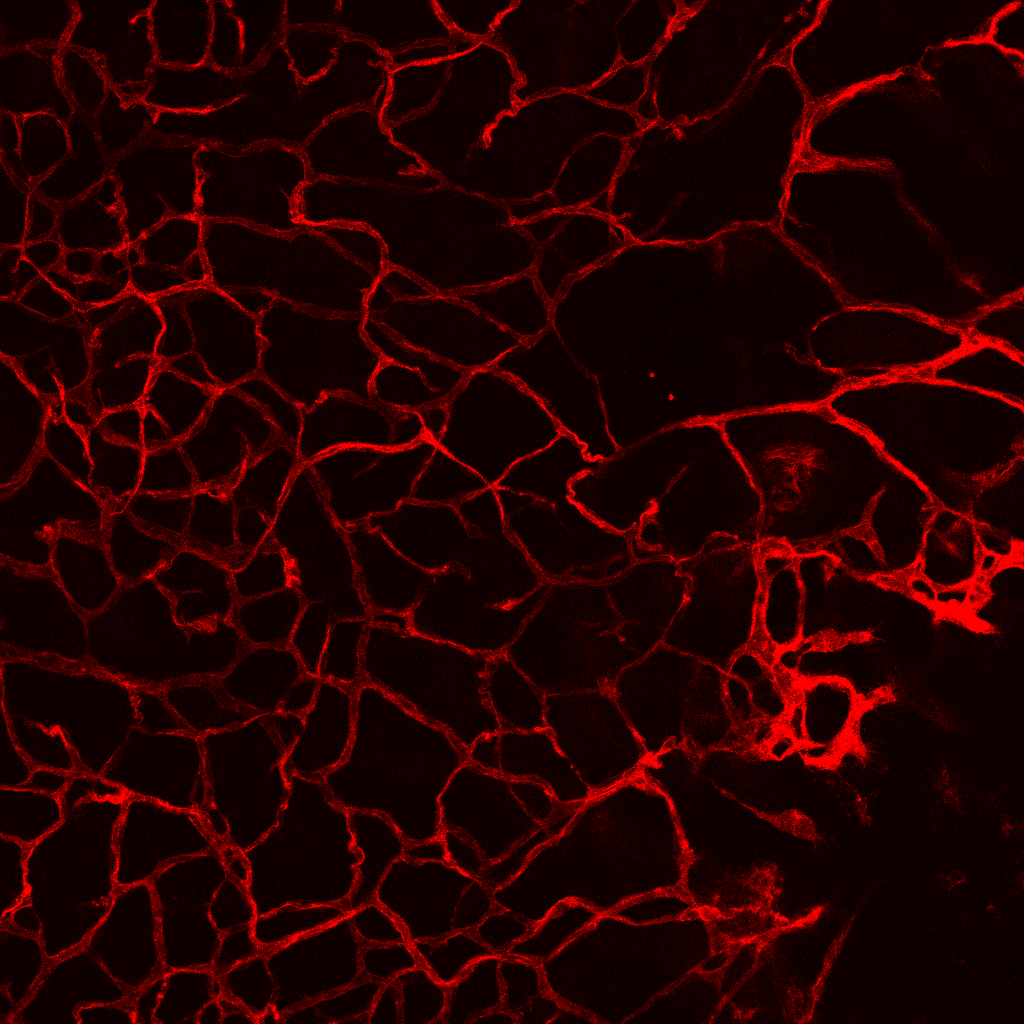

Supplement: Supplementary file 4 — Source Data Fig. 3 [file 44321_2024_25_MOESM4_ESM.zip › figure 3/3B/3B IB4 OIR+AAV-blank deep.tif]

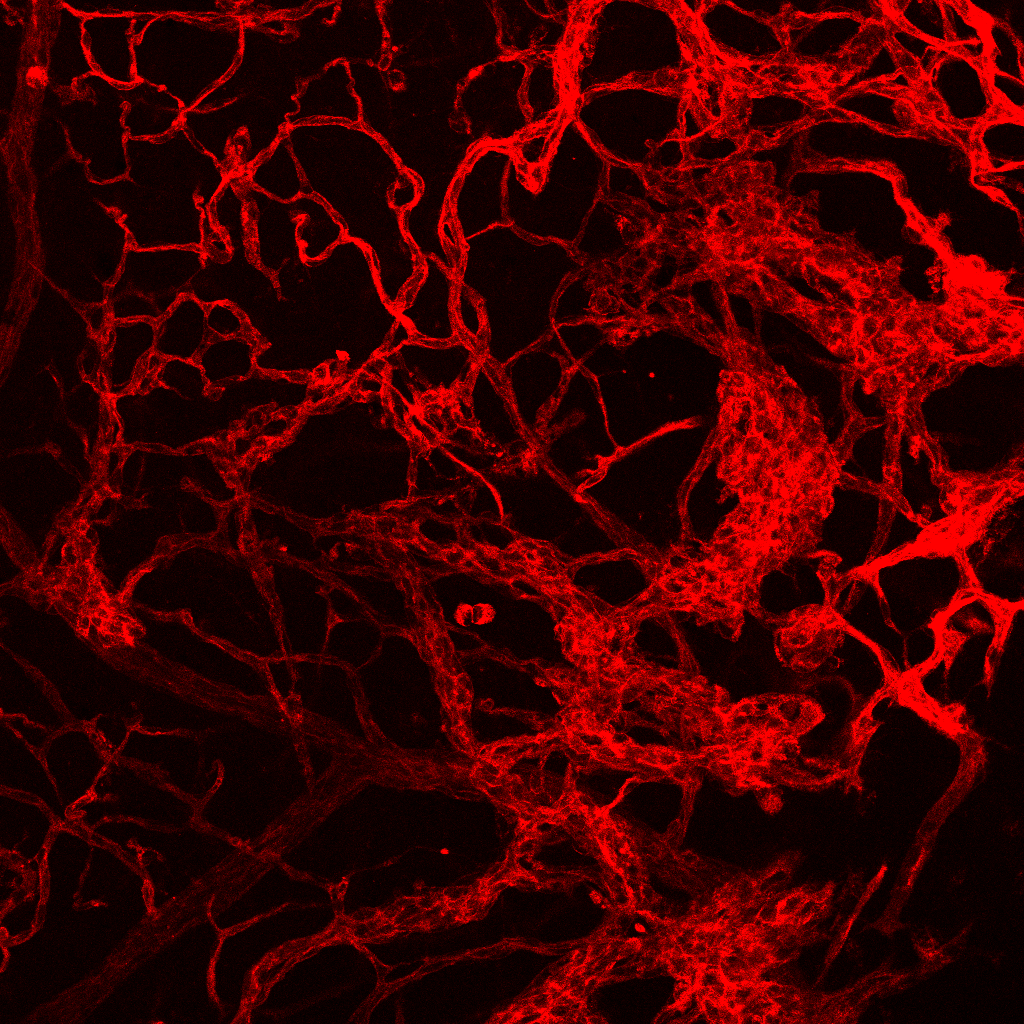

Supplement: Supplementary file 4 — Source Data Fig. 3 [file 44321_2024_25_MOESM4_ESM.zip › figure 3/3B/3B IB4 OIR+AAV-blank superficial.tif]

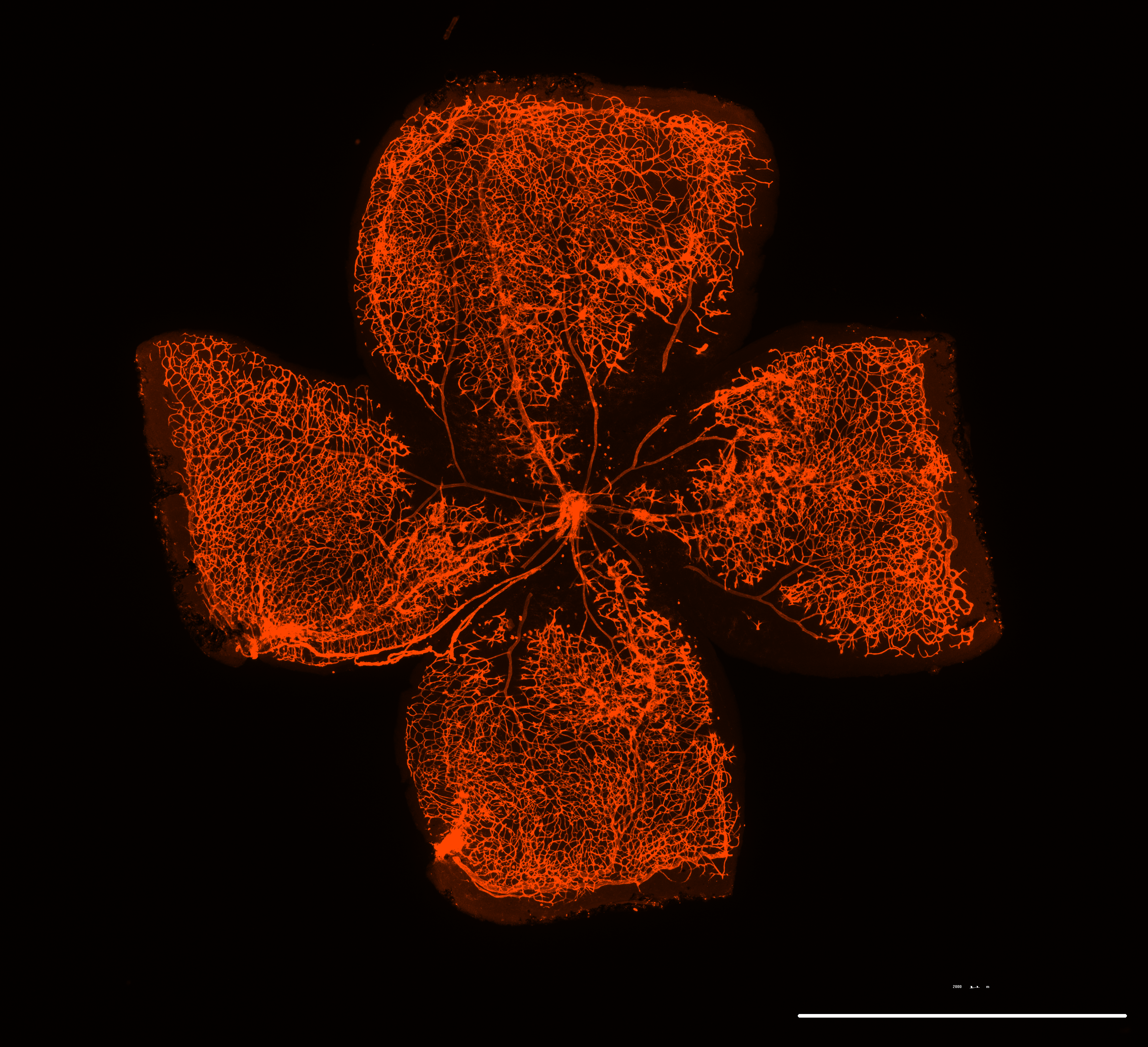

Supplement: Supplementary file 4 — Source Data Fig. 3 [file 44321_2024_25_MOESM4_ESM.zip › figure 3/3B/3B IB4 OIR+AAV-blank upper line.tif]
