# Supplementary figures and images for: Lactylation-driven FTO targets CDK2 to aggravate microvascular anomalies in diabetic retinopathy (part 2 of 4)
Source: EMBO Mol Med. 2024 Jan 31;16(2):294–318. doi: 10.1038/s44321-024-00025-1 (PMC10897304; doi:10.1038/s44321-024-00025-1)

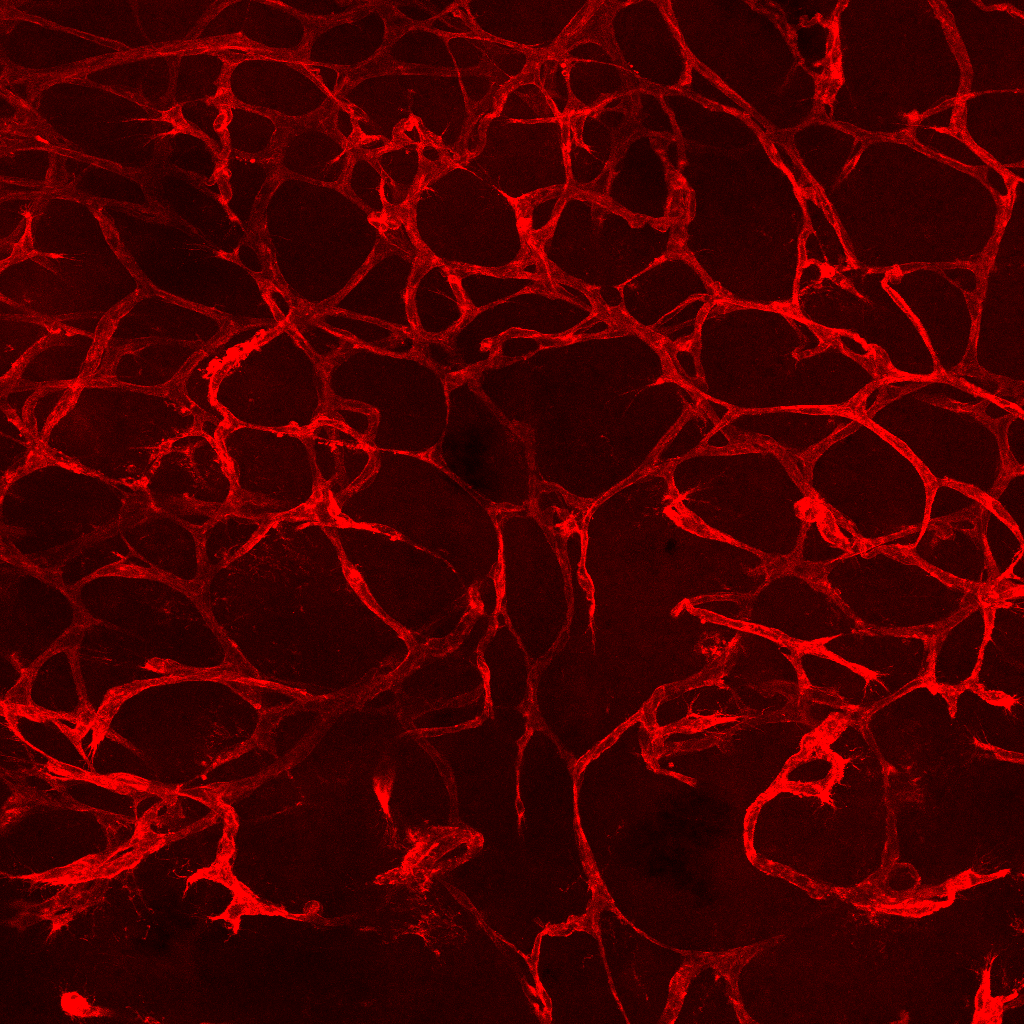

Supplement: Supplementary file 4 — Source Data Fig. 3 [file 44321_2024_25_MOESM4_ESM.zip › figure 3/3B/3B IB4 OIR+AAV-Fto deep.tiff]

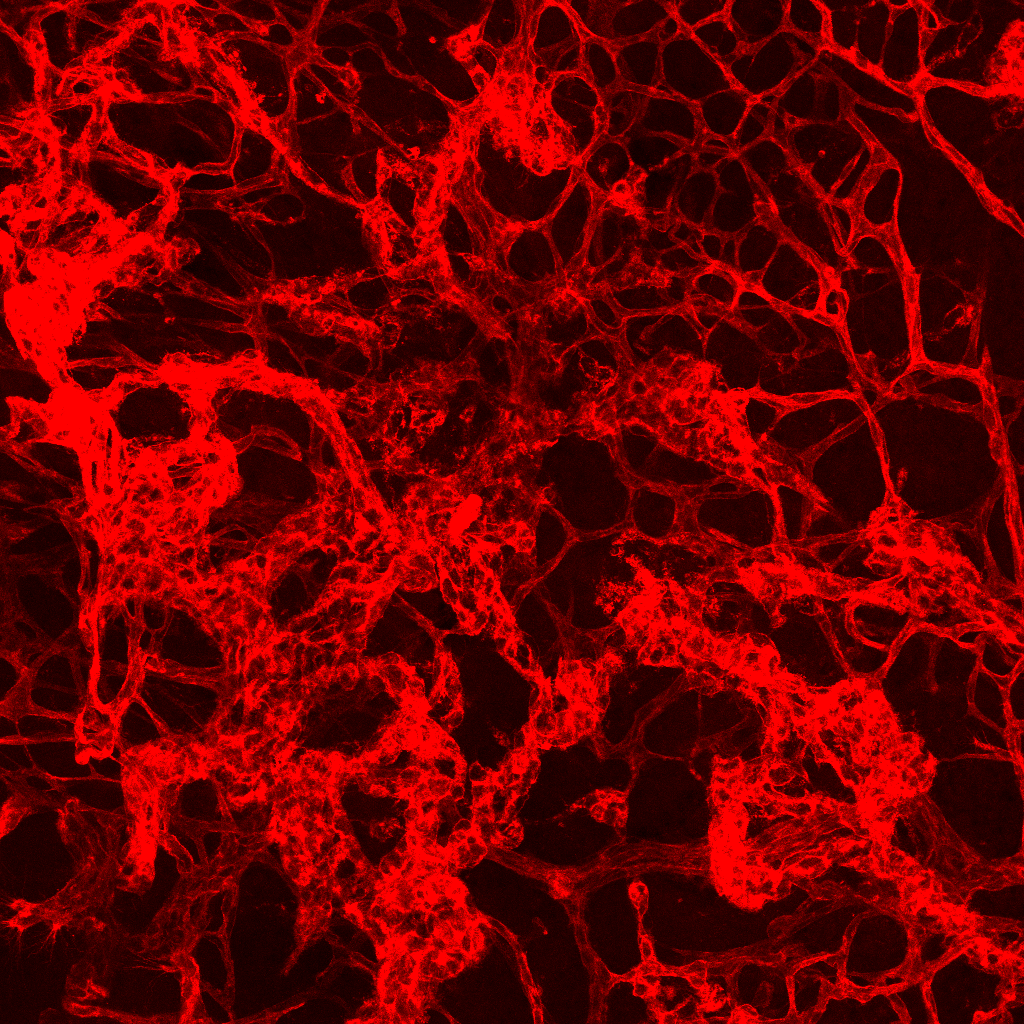

Supplement: Supplementary file 4 — Source Data Fig. 3 [file 44321_2024_25_MOESM4_ESM.zip › figure 3/3B/3B IB4 OIR+AAV-Fto superficial.tiff]

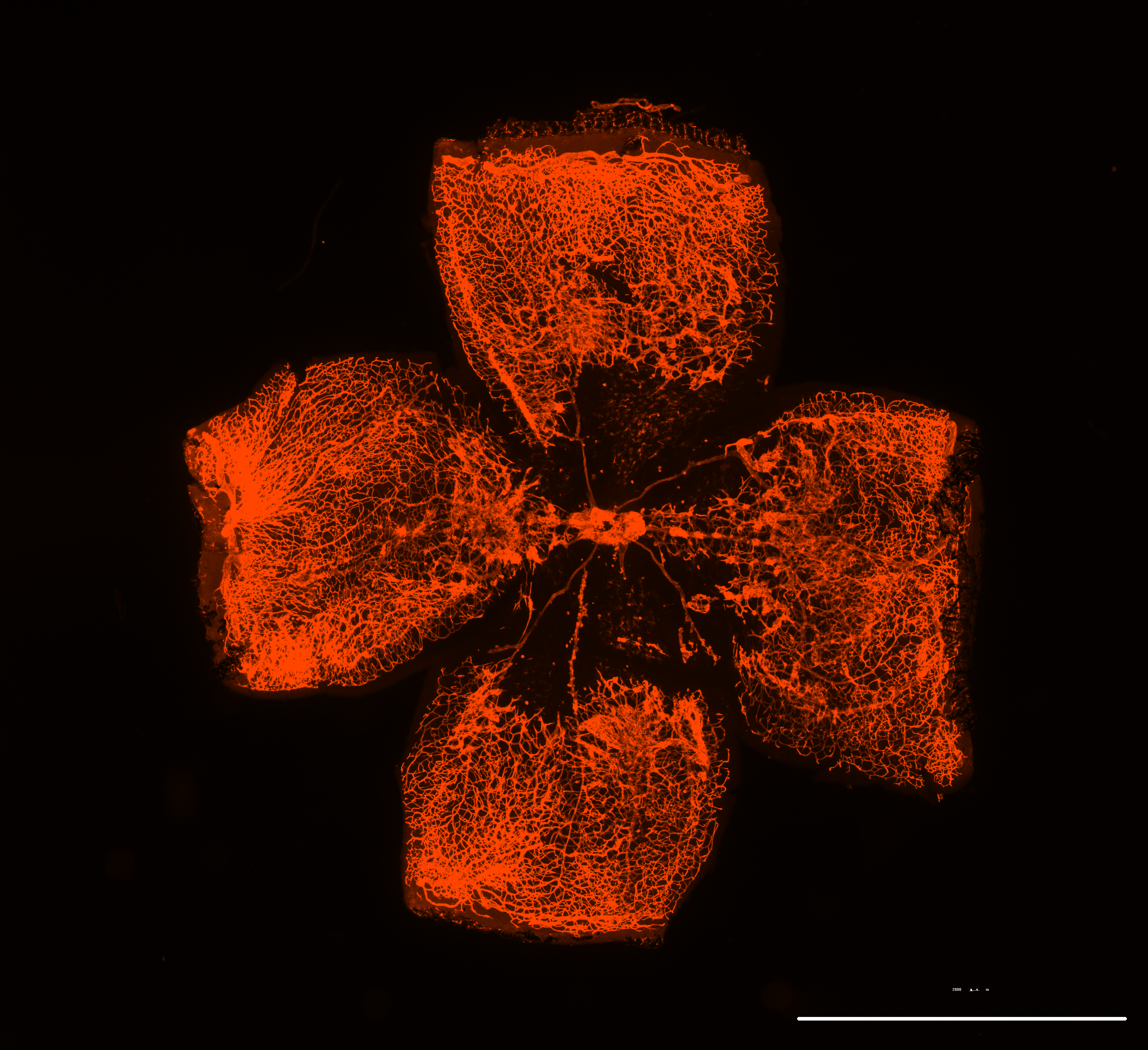

Supplement: Supplementary file 4 — Source Data Fig. 3 [file 44321_2024_25_MOESM4_ESM.zip › figure 3/3B/3B IB4 OIR+AAV-Fto upper line.tif]

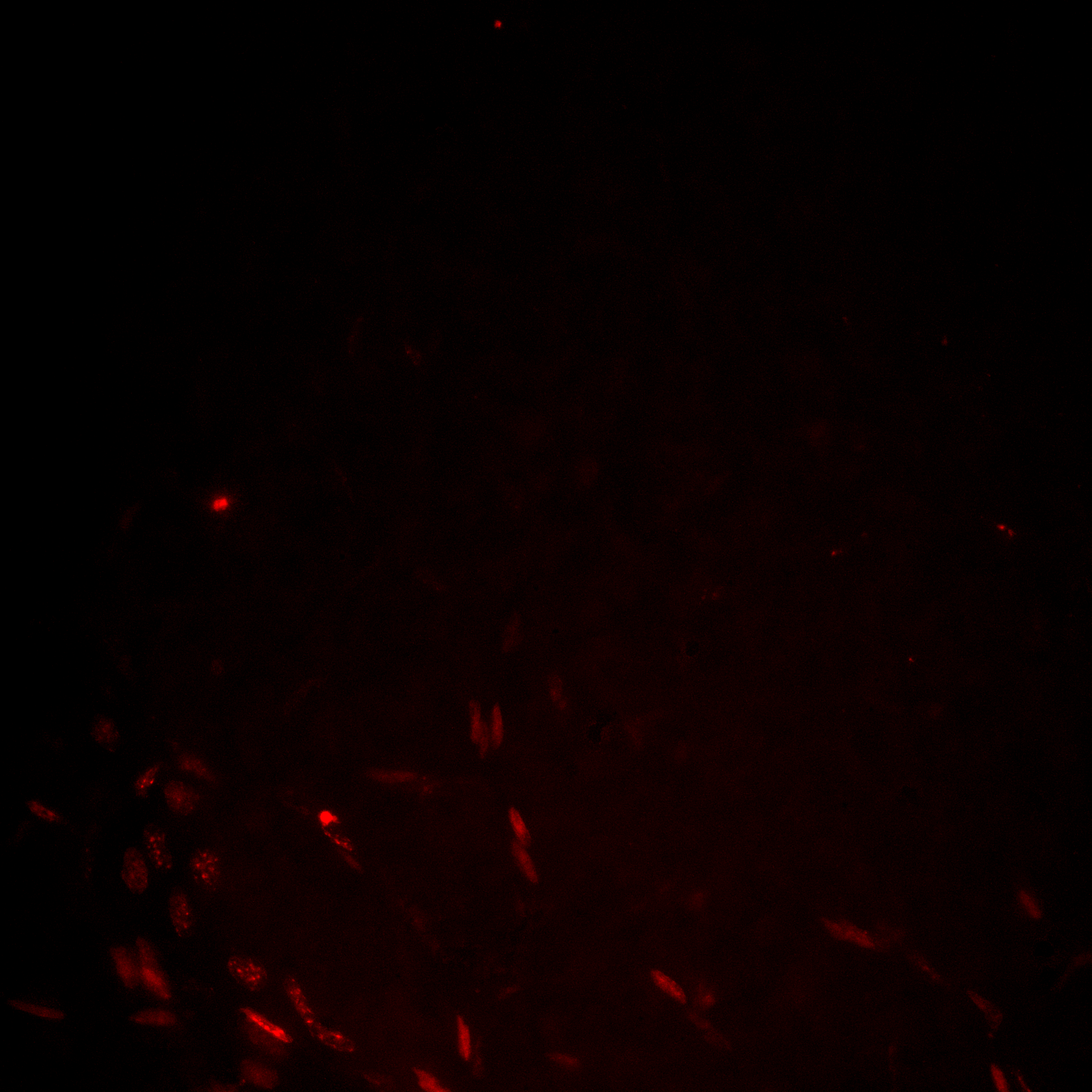

Supplement: Supplementary file 4 — Source Data Fig. 3 [file 44321_2024_25_MOESM4_ESM.zip › figure 3/3C/3C Ctrl EdU.tif]

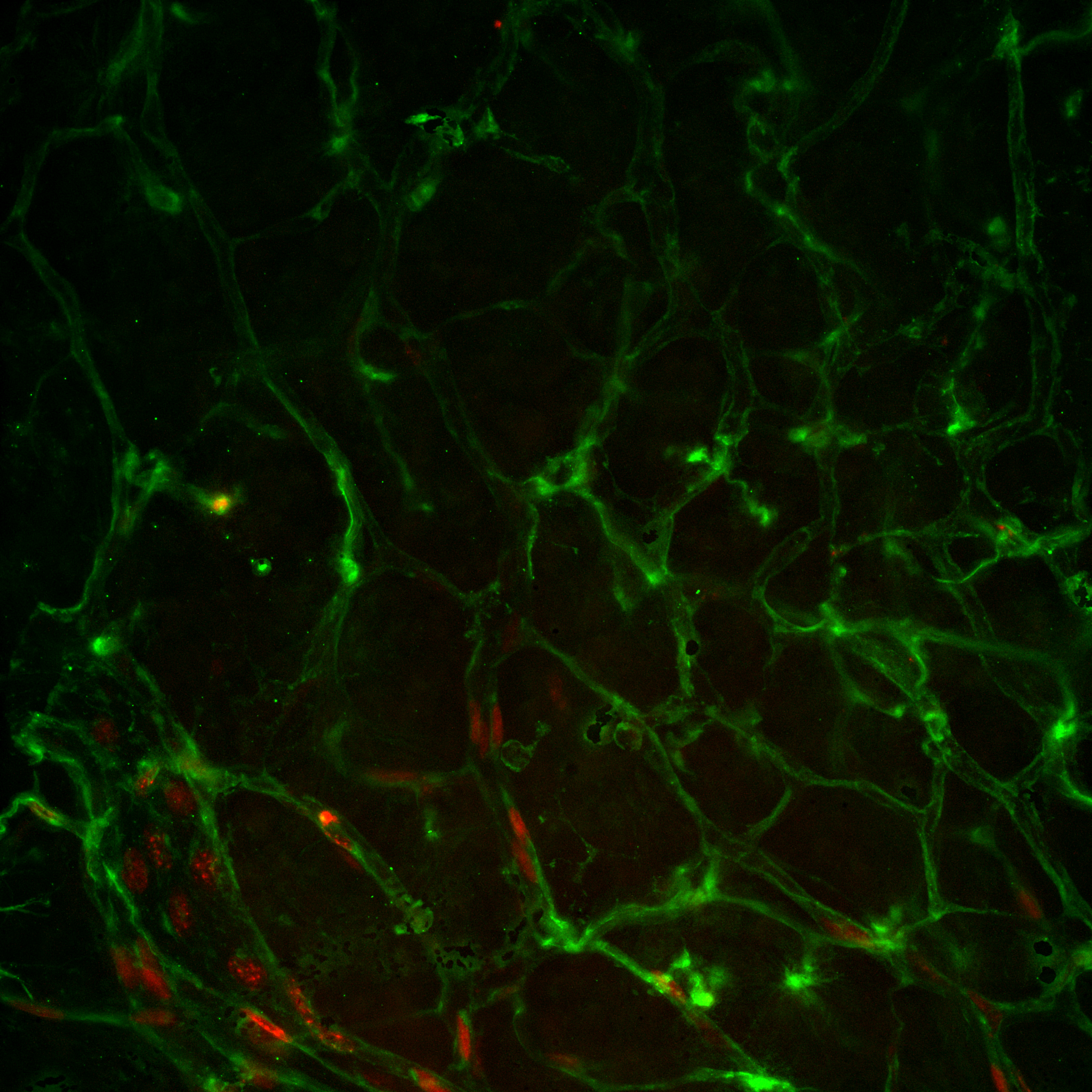

Supplement: Supplementary file 4 — Source Data Fig. 3 [file 44321_2024_25_MOESM4_ESM.zip › figure 3/3C/3C Ctrl EdU+IB4.tif]

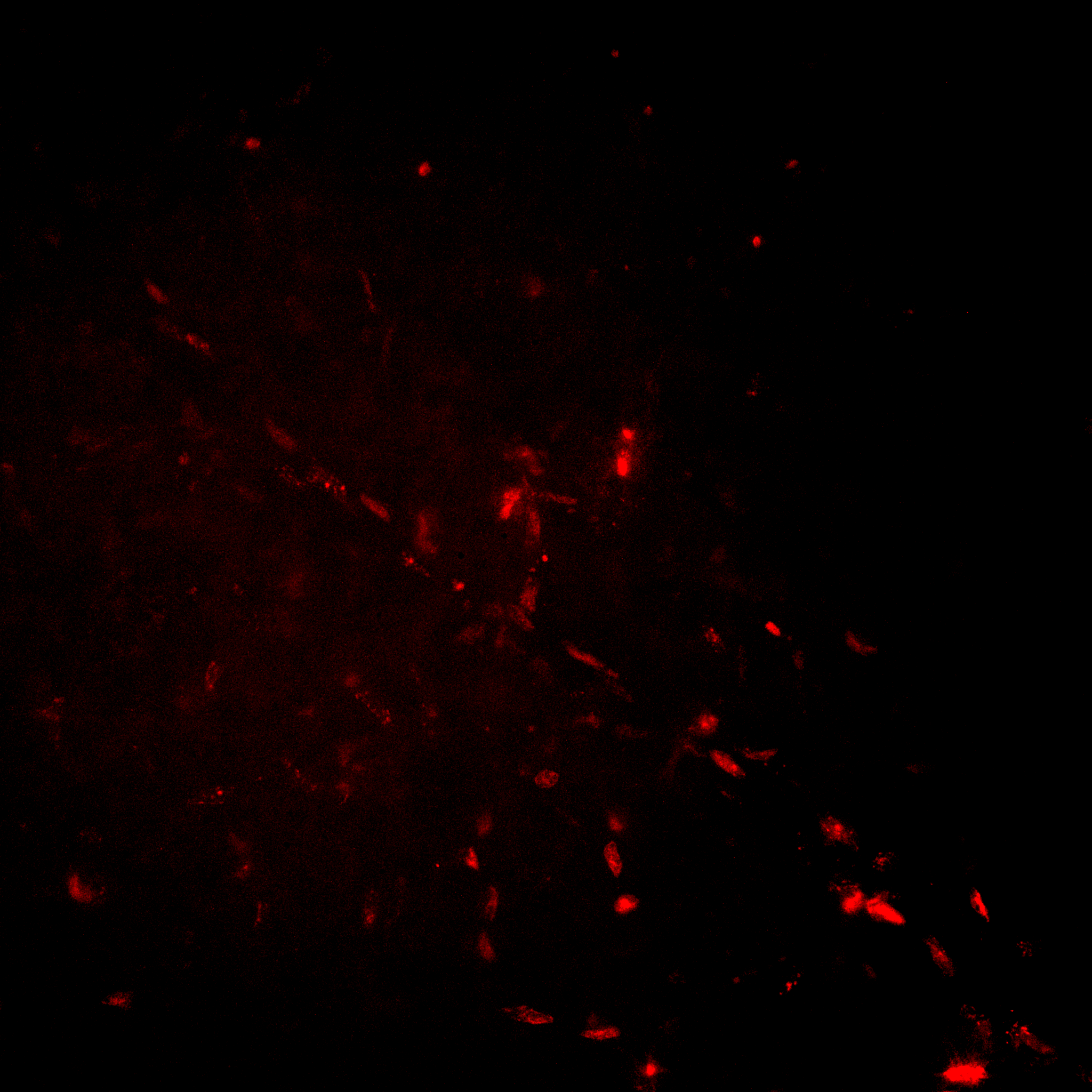

Supplement: Supplementary file 4 — Source Data Fig. 3 [file 44321_2024_25_MOESM4_ESM.zip › figure 3/3C/3C OIR EdU.tif]

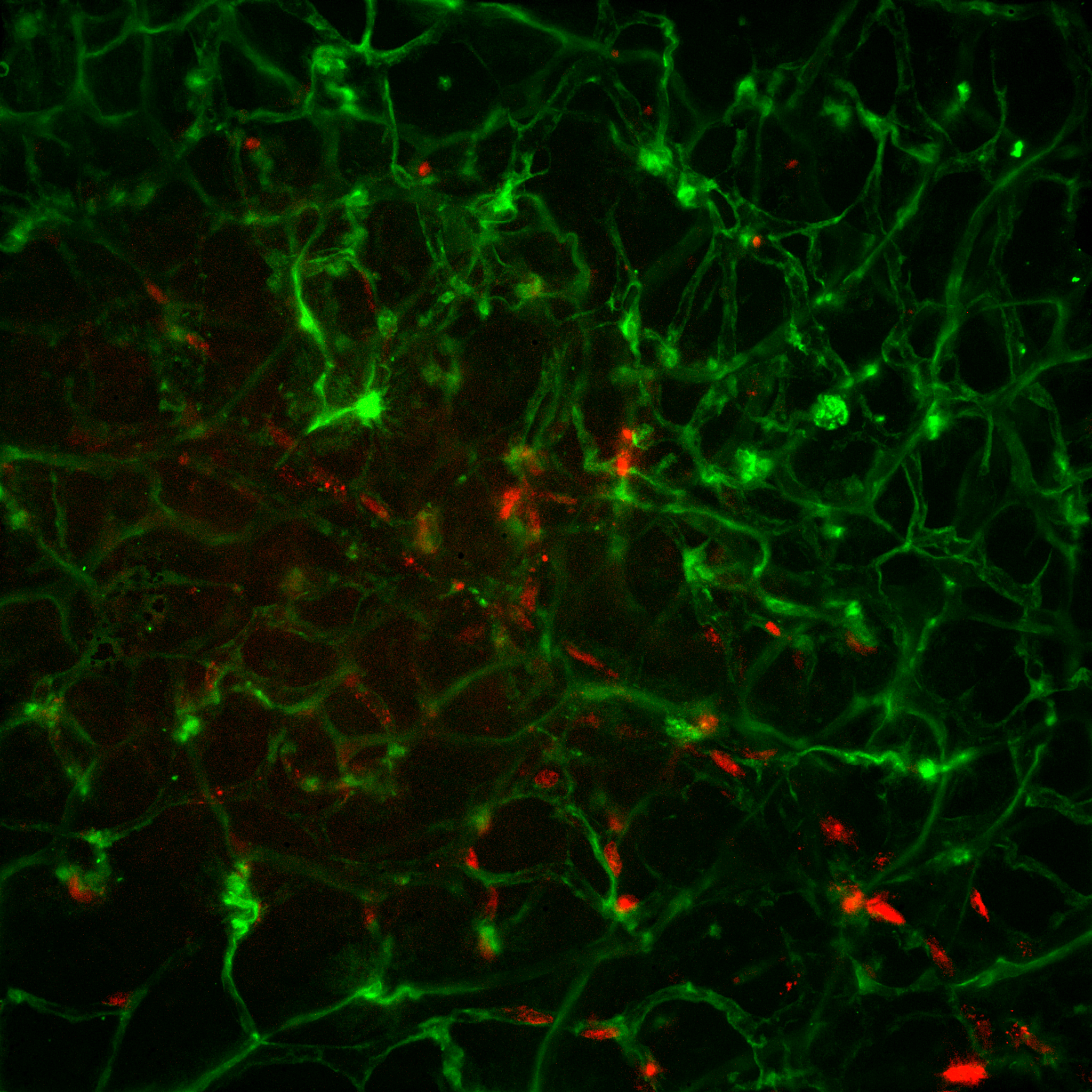

Supplement: Supplementary file 4 — Source Data Fig. 3 [file 44321_2024_25_MOESM4_ESM.zip › figure 3/3C/3C OIR EdU+IB4.tif]

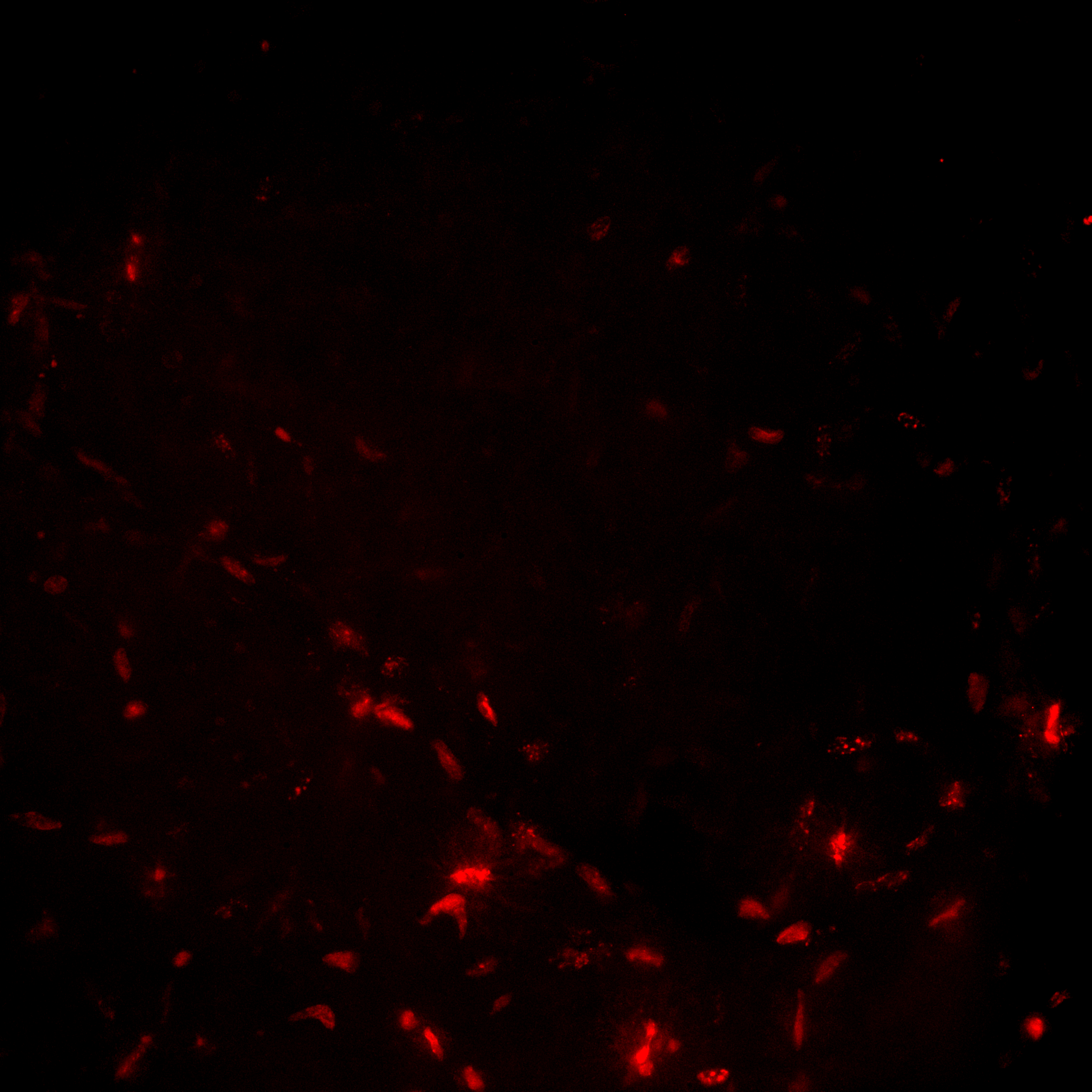

Supplement: Supplementary file 4 — Source Data Fig. 3 [file 44321_2024_25_MOESM4_ESM.zip › figure 3/3C/3C OIR+AAV-blank EdU.tif]

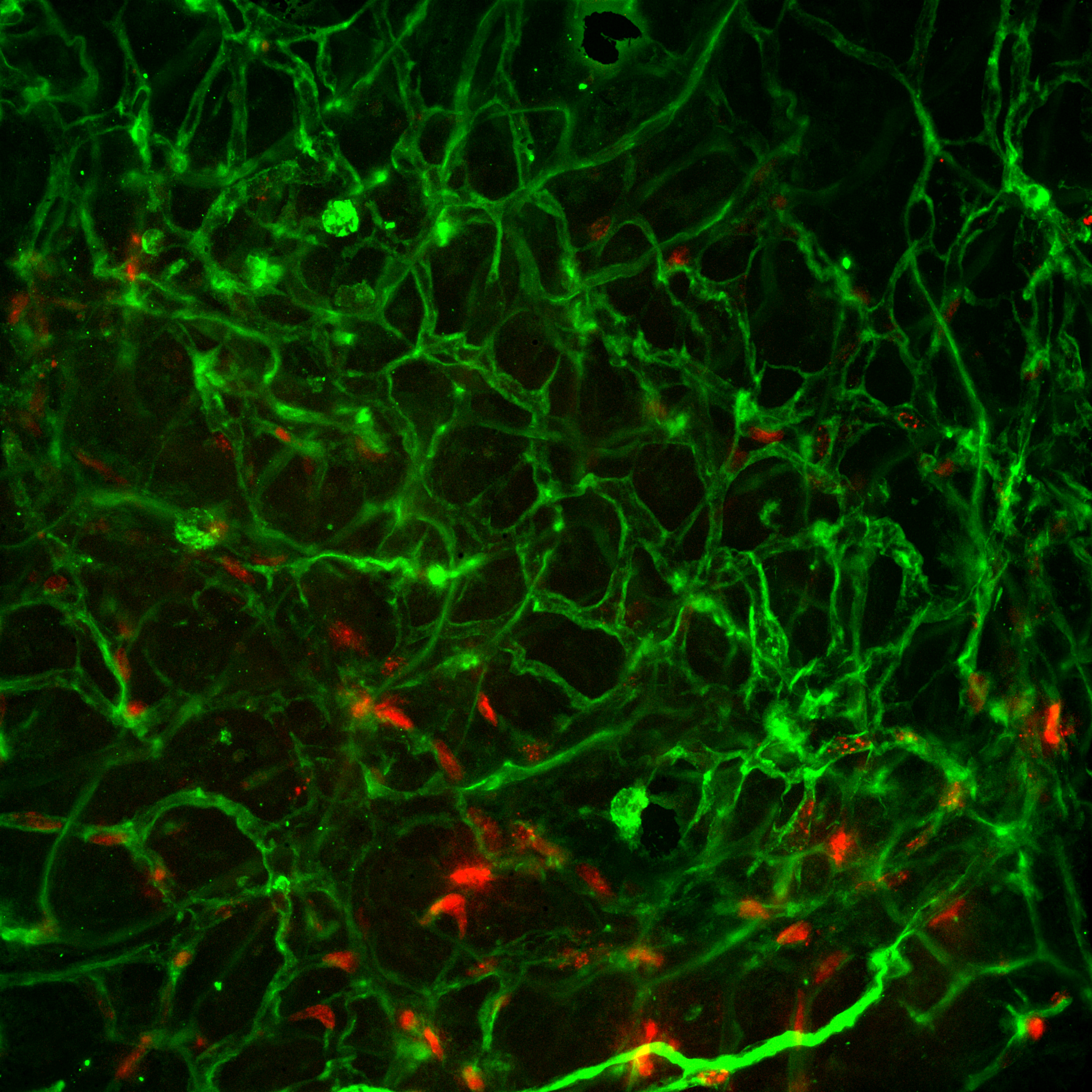

Supplement: Supplementary file 4 — Source Data Fig. 3 [file 44321_2024_25_MOESM4_ESM.zip › figure 3/3C/3C OIR+AAV-blank EdU+IB4.tif]

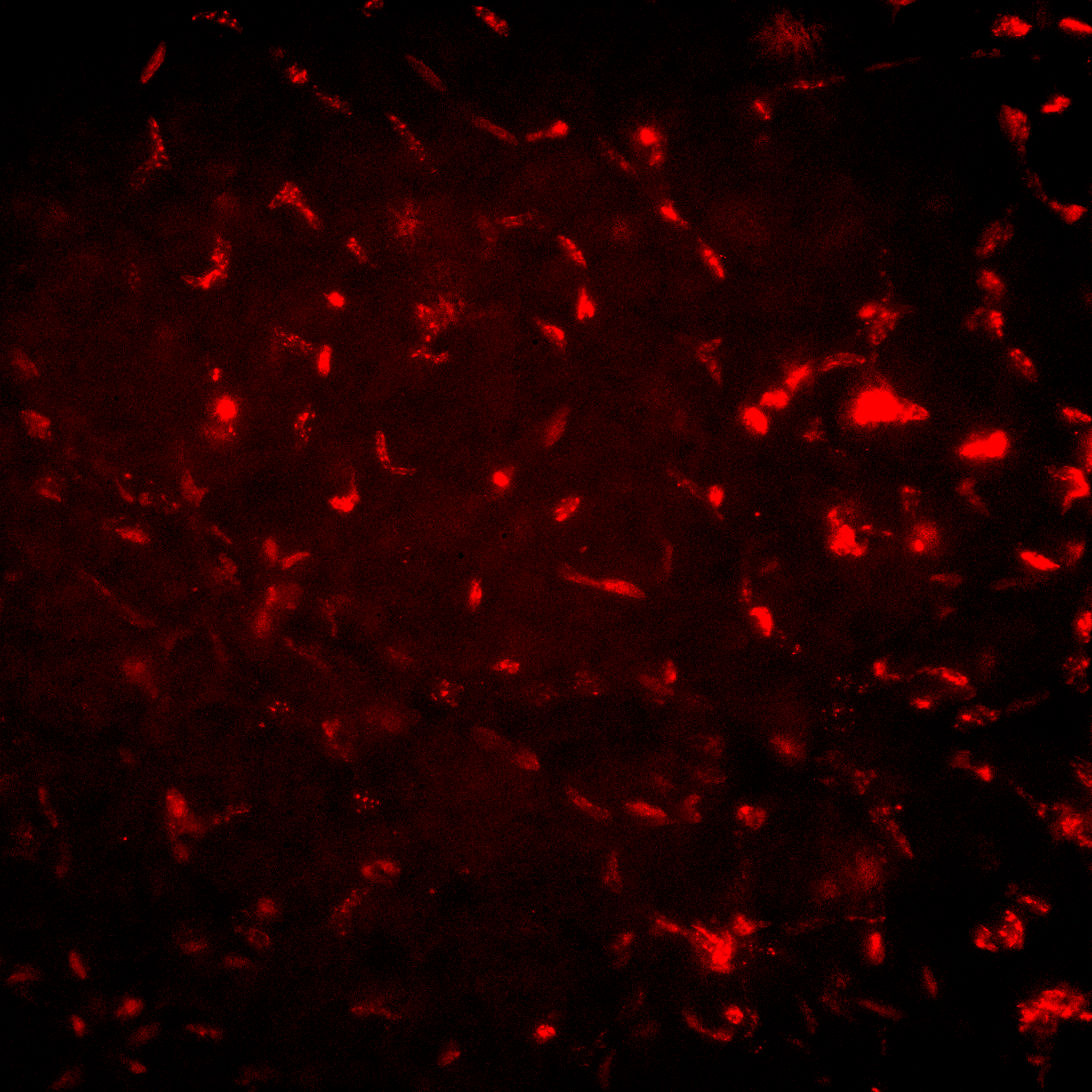

Supplement: Supplementary file 4 — Source Data Fig. 3 [file 44321_2024_25_MOESM4_ESM.zip › figure 3/3C/3C OIR+AAV-Fto EdU.tif]

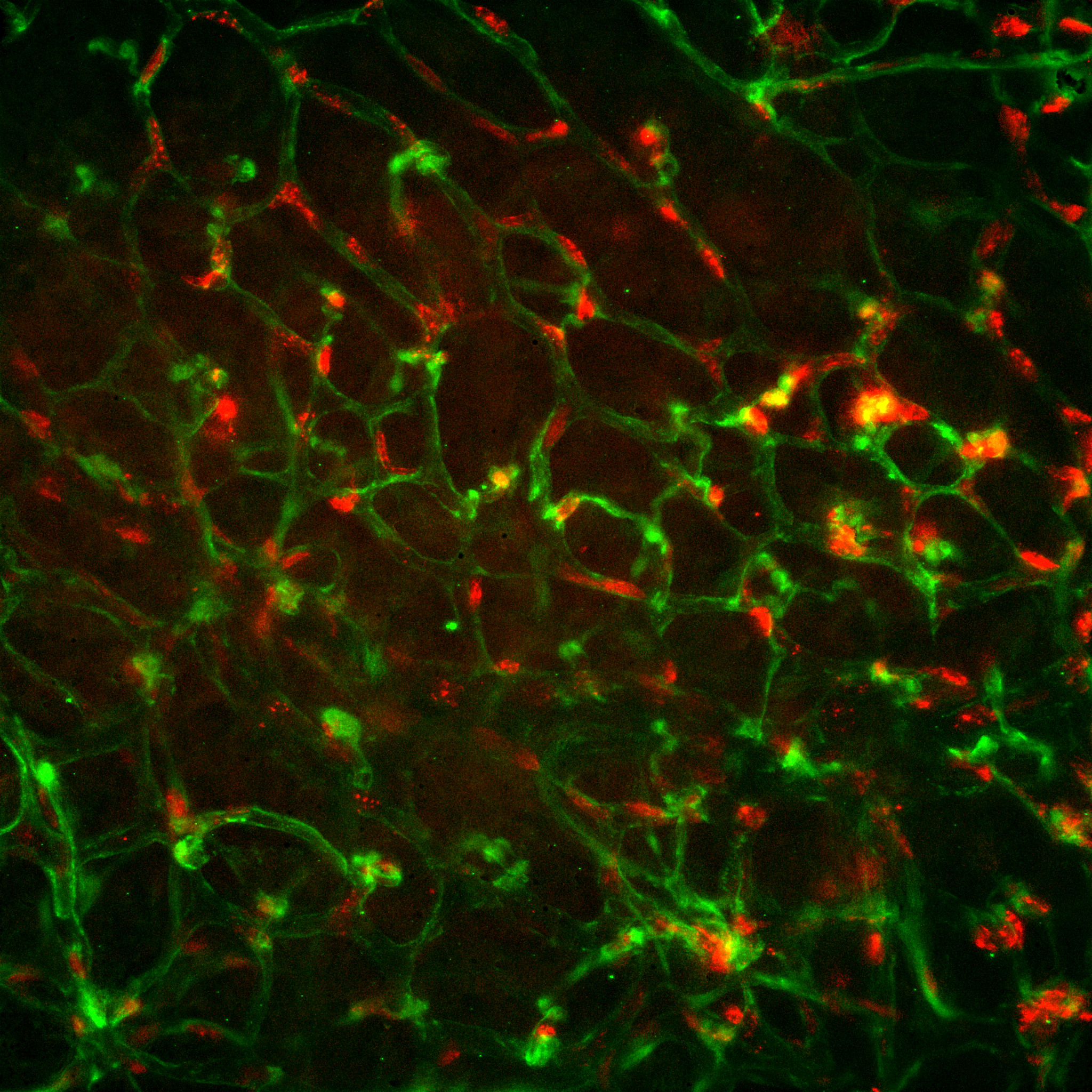

Supplement: Supplementary file 4 — Source Data Fig. 3 [file 44321_2024_25_MOESM4_ESM.zip › figure 3/3C/3C OIR+AAV-Fto EdU+IB4.tif]

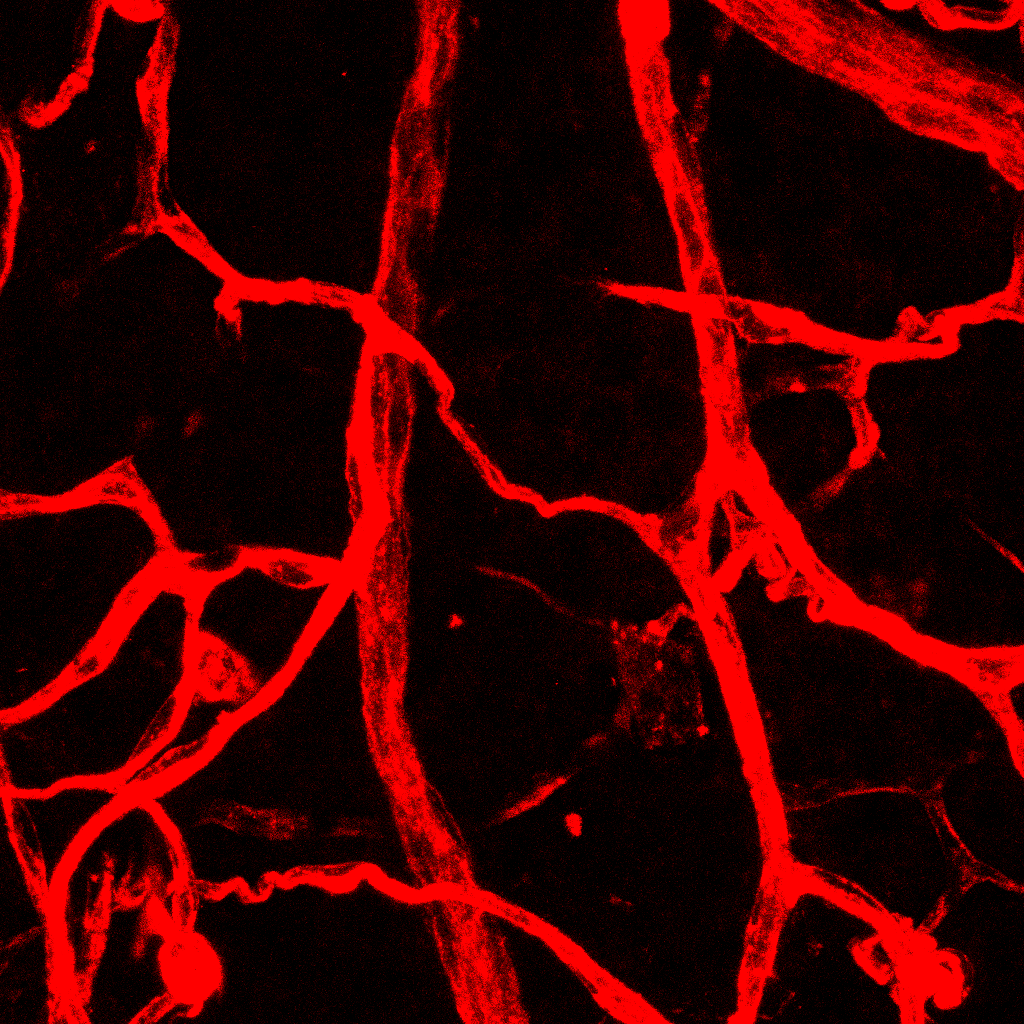

Supplement: Supplementary file 4 — Source Data Fig. 3 [file 44321_2024_25_MOESM4_ESM.zip › figure 3/3D/3D IB4 tip cell Ctrl.tif]

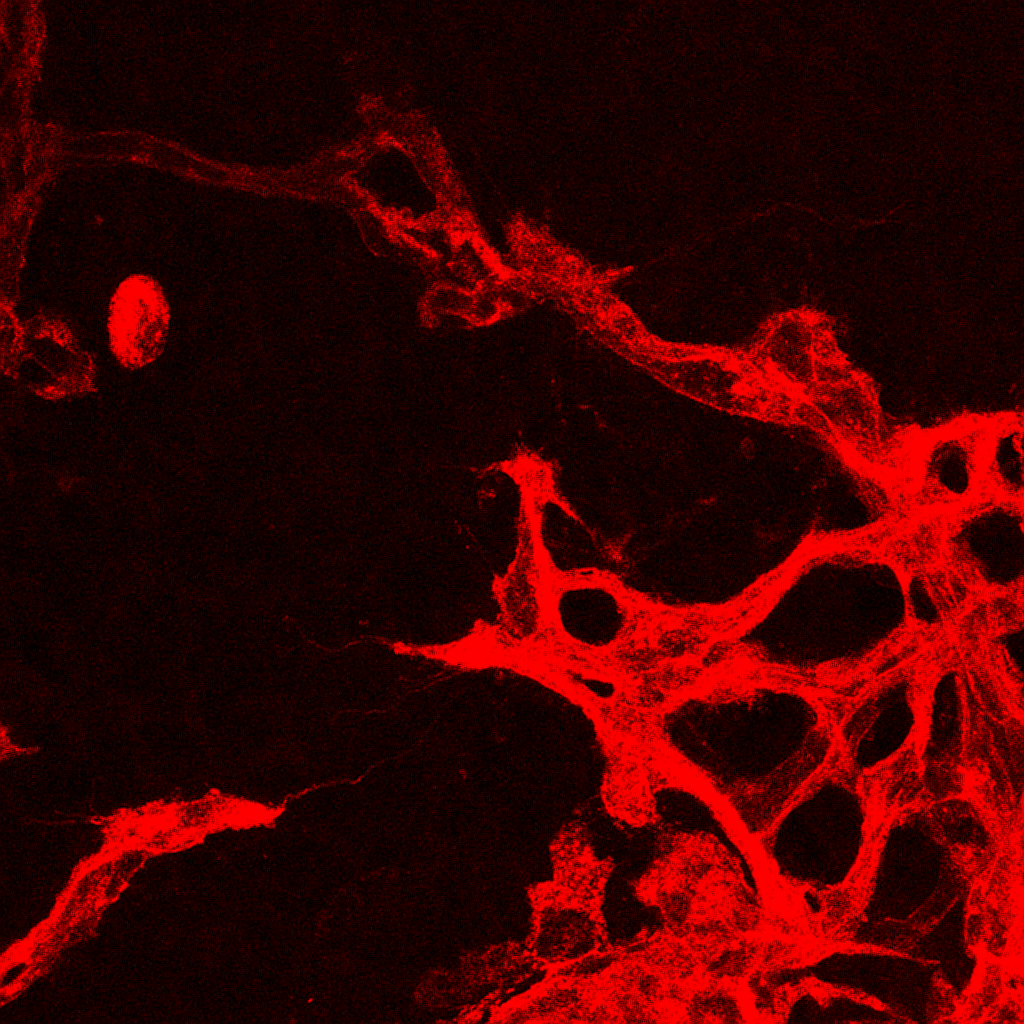

Supplement: Supplementary file 4 — Source Data Fig. 3 [file 44321_2024_25_MOESM4_ESM.zip › figure 3/3D/3D IB4 tip cell OIR.tif]

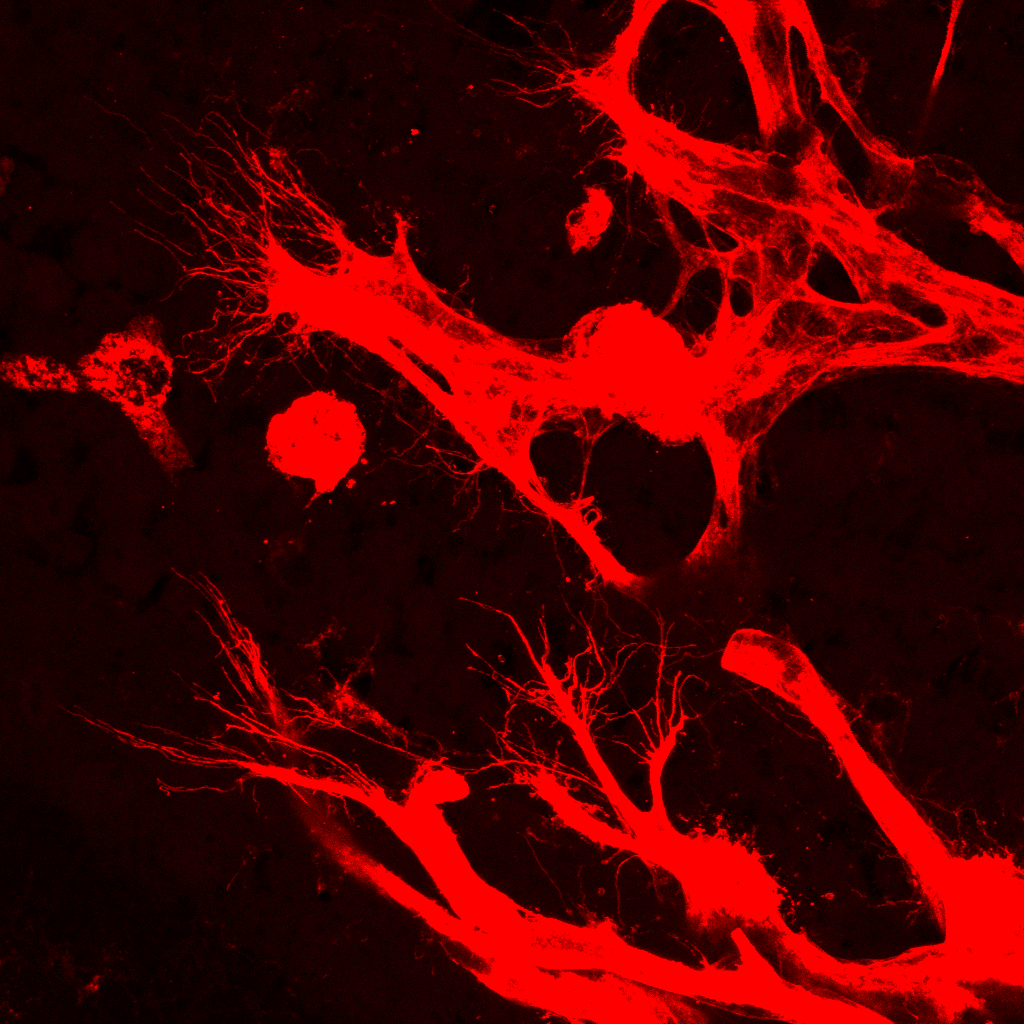

Supplement: Supplementary file 4 — Source Data Fig. 3 [file 44321_2024_25_MOESM4_ESM.zip › figure 3/3D/3D IB4 tip cell OIR+AAV-Fto.tif]

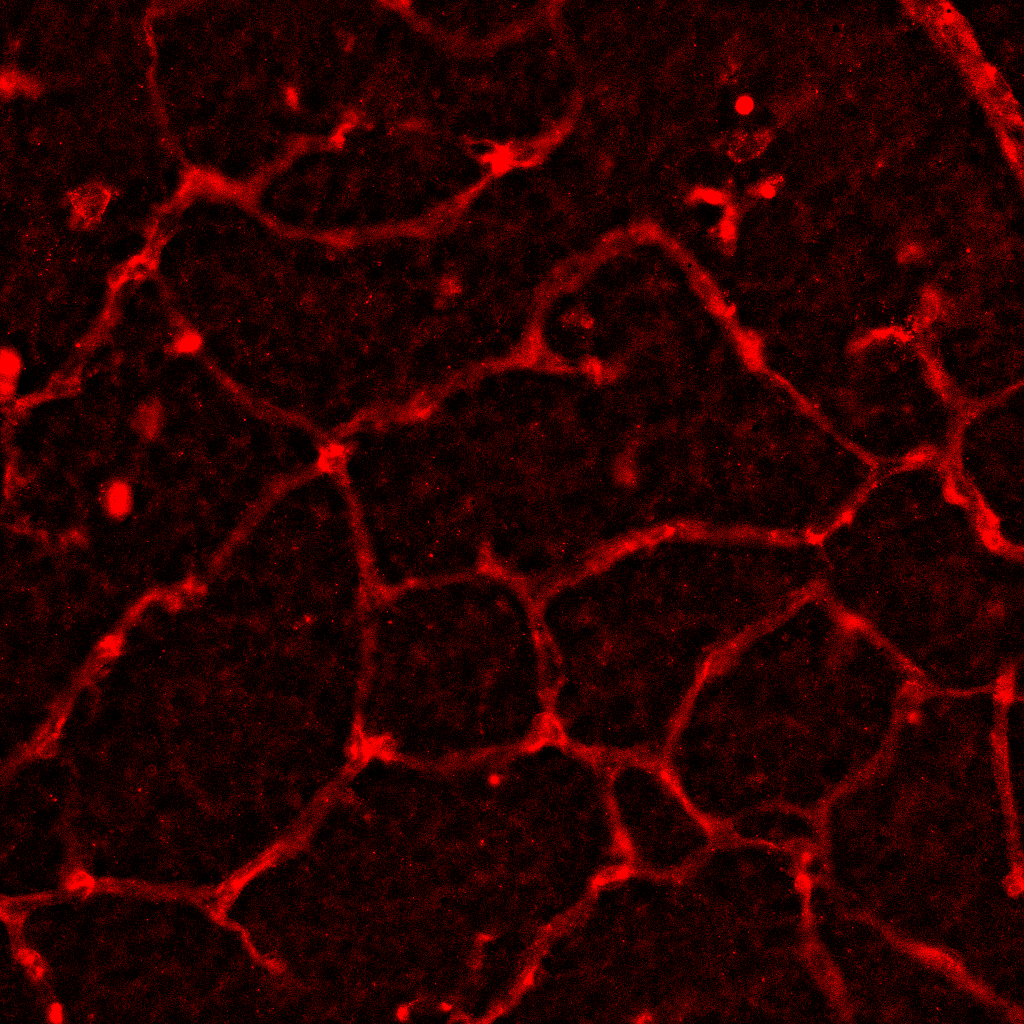

Supplement: Supplementary file 4 — Source Data Fig. 3 [file 44321_2024_25_MOESM4_ESM.zip › figure 3/3G/3G Ctrl PDGFRa┬.tif]

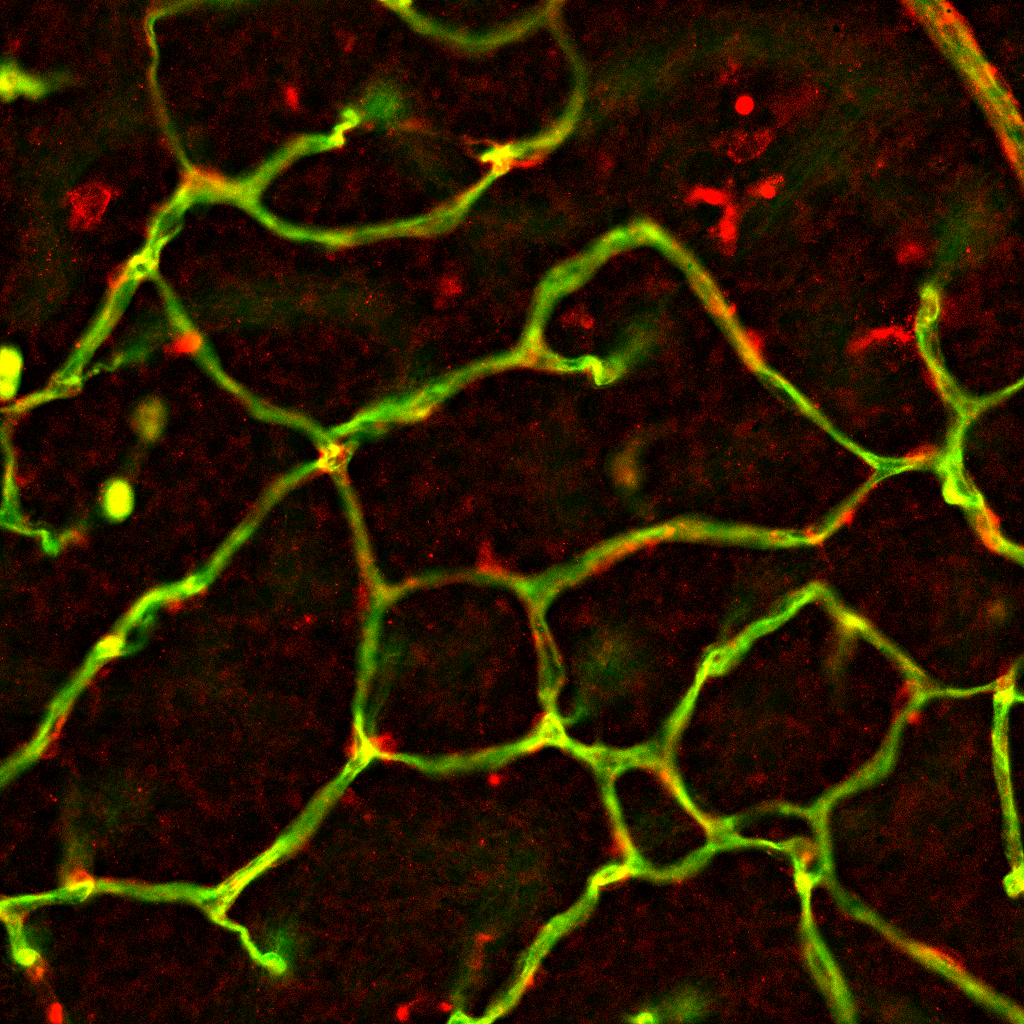

Supplement: Supplementary file 4 — Source Data Fig. 3 [file 44321_2024_25_MOESM4_ESM.zip › figure 3/3G/3G Ctrl PDGFRa┬+IB4.tif]

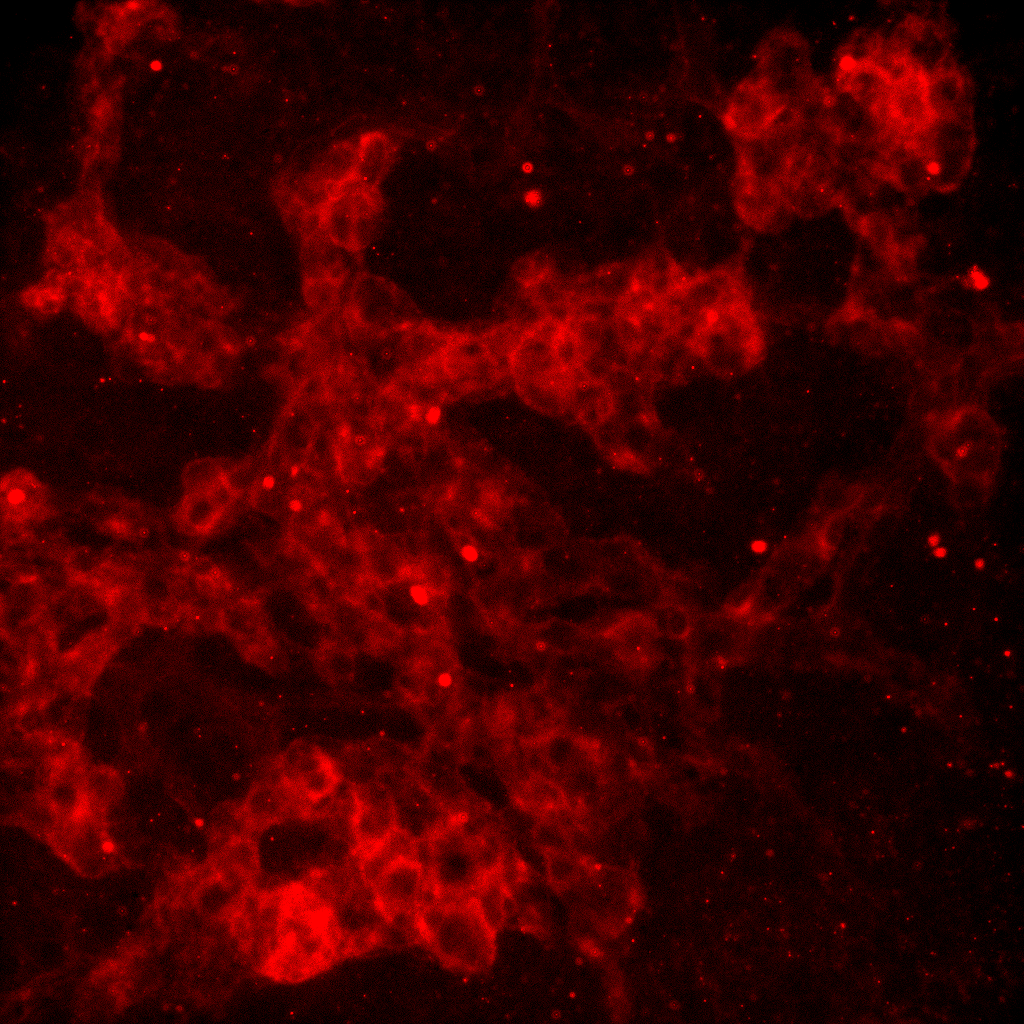

Supplement: Supplementary file 4 — Source Data Fig. 3 [file 44321_2024_25_MOESM4_ESM.zip › figure 3/3G/3G OIR PDGFRa┬.tif]

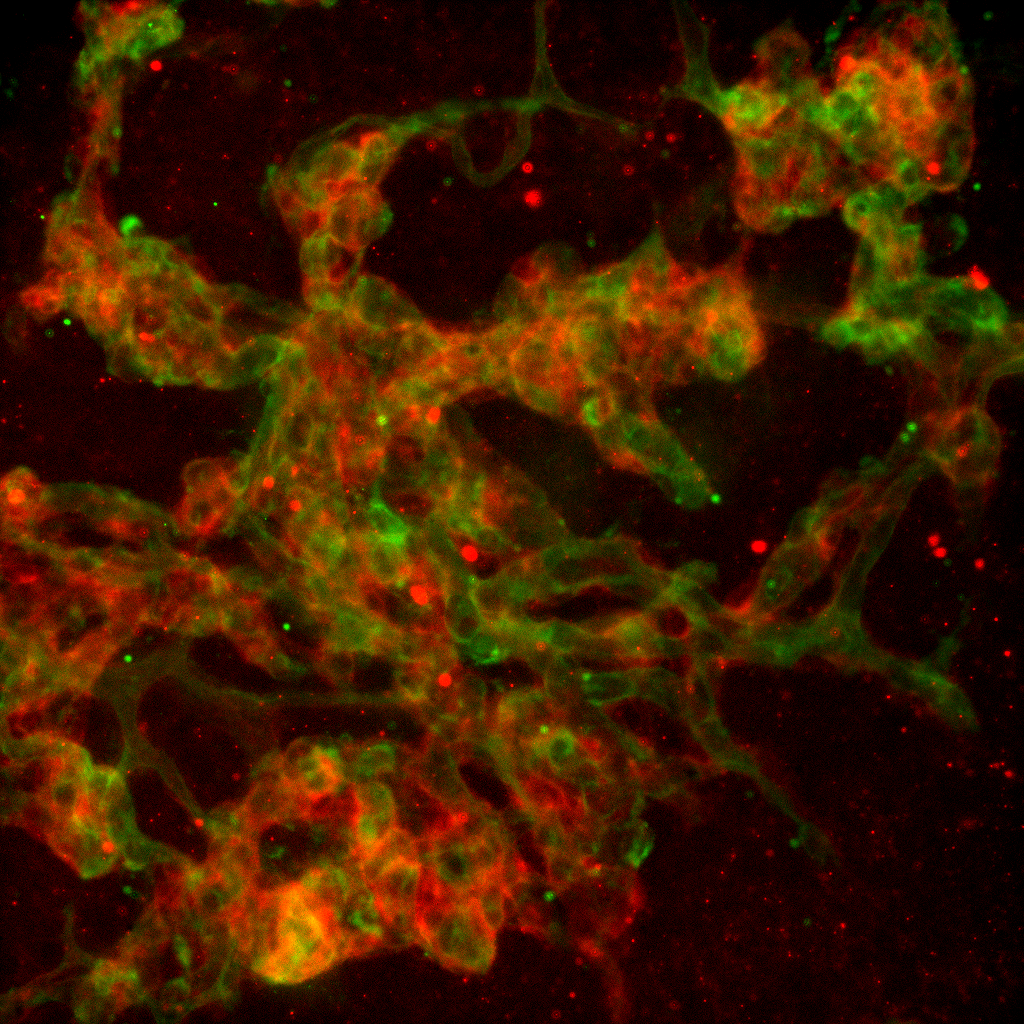

Supplement: Supplementary file 4 — Source Data Fig. 3 [file 44321_2024_25_MOESM4_ESM.zip › figure 3/3G/3G OIR PDGFRa┬+IB4.tif]

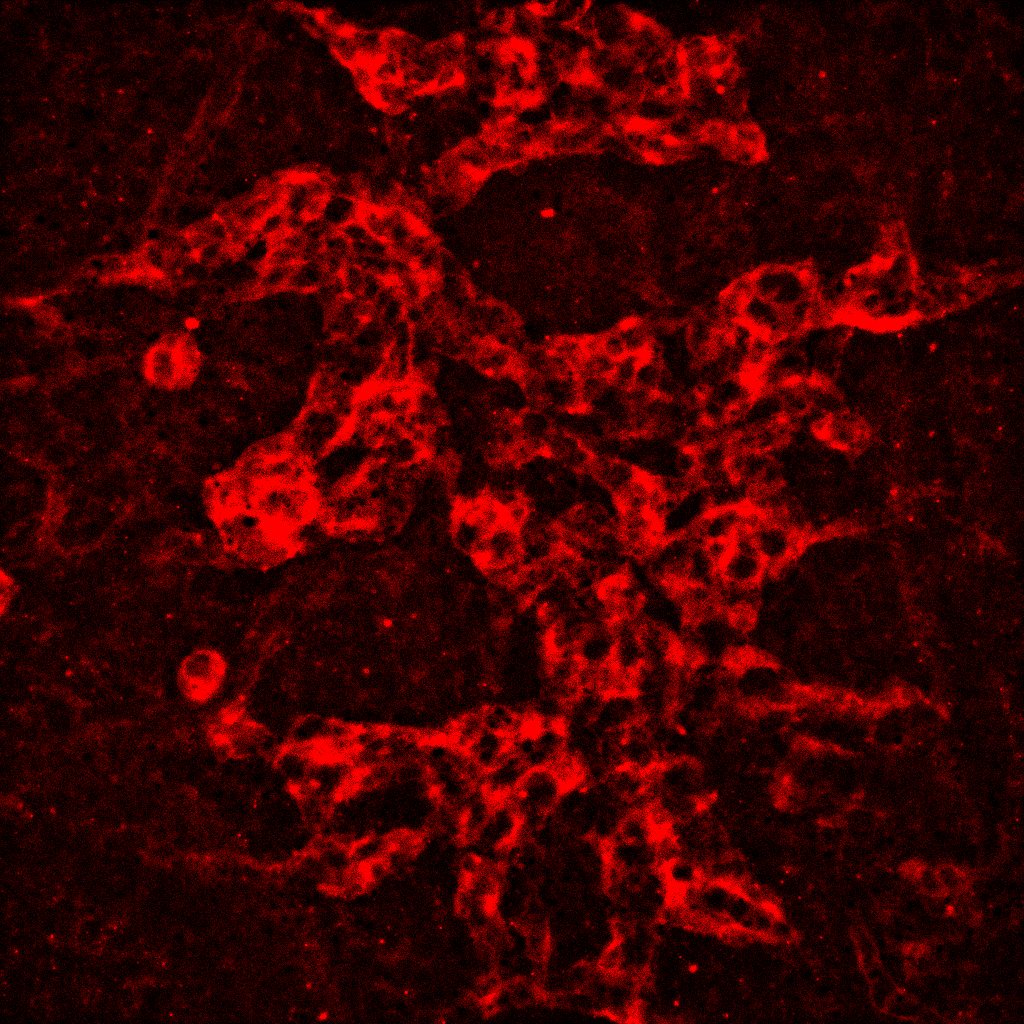

Supplement: Supplementary file 4 — Source Data Fig. 3 [file 44321_2024_25_MOESM4_ESM.zip › figure 3/3G/3G OIR+AAV-blank PDGFRa┬.tif]

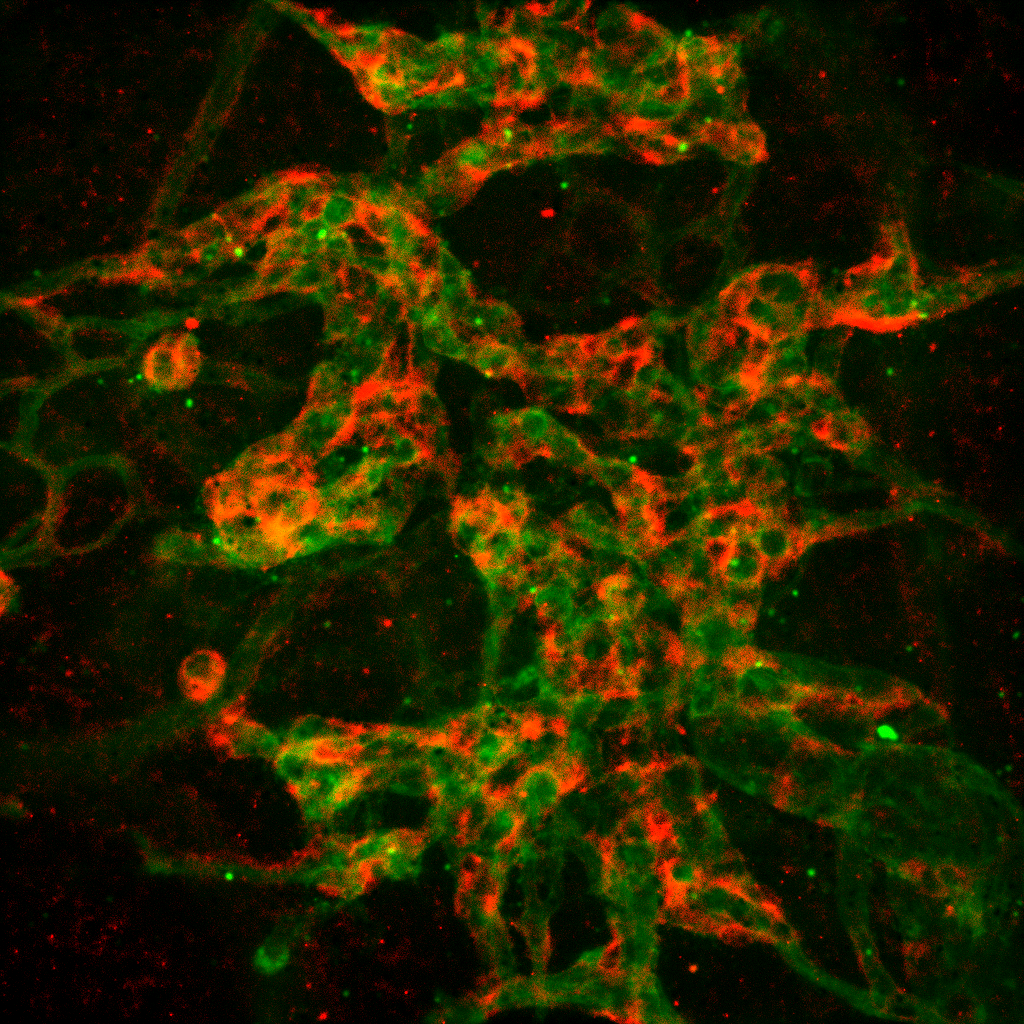

Supplement: Supplementary file 4 — Source Data Fig. 3 [file 44321_2024_25_MOESM4_ESM.zip › figure 3/3G/3G OIR+AAV-blank PDGFRa┬+IB4.tif]

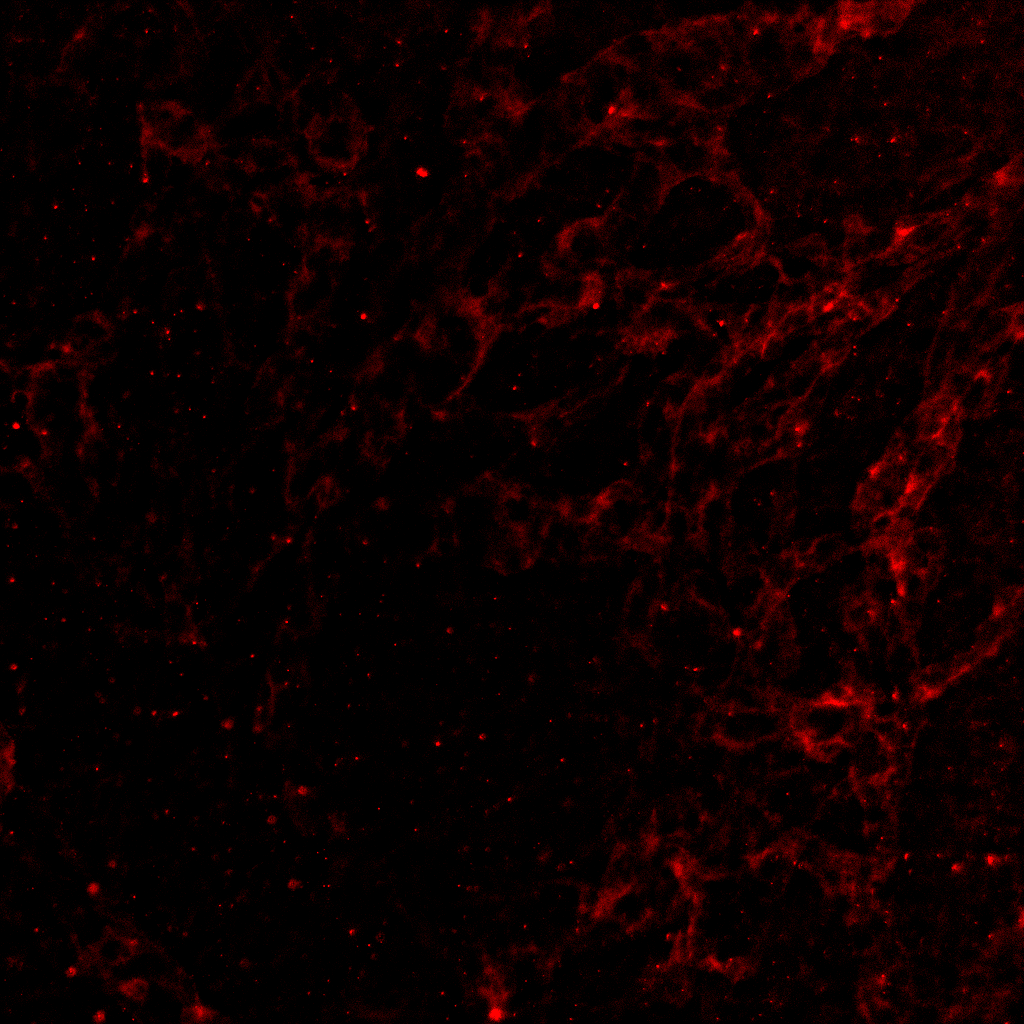

Supplement: Supplementary file 4 — Source Data Fig. 3 [file 44321_2024_25_MOESM4_ESM.zip › figure 3/3G/3G OIR+AAV-Fto PDGFRa┬.tif]

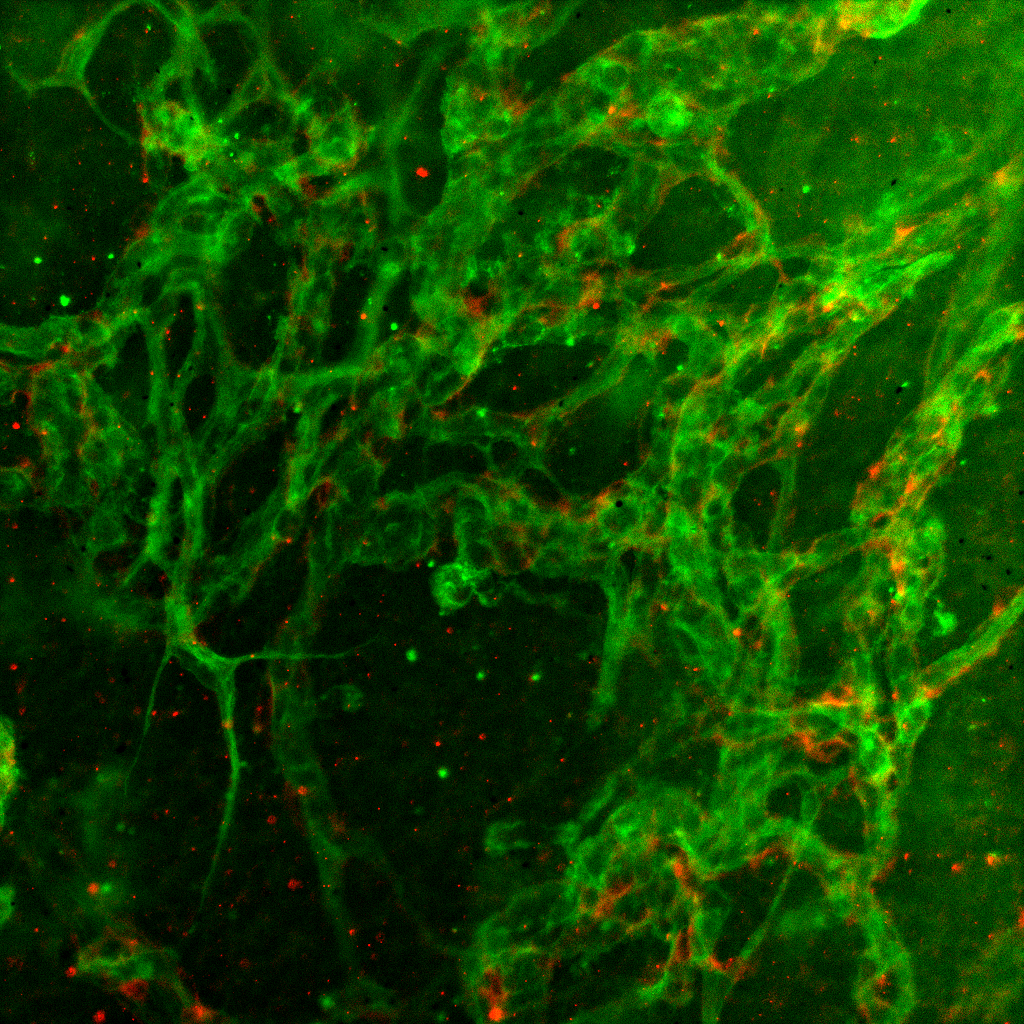

Supplement: Supplementary file 4 — Source Data Fig. 3 [file 44321_2024_25_MOESM4_ESM.zip › figure 3/3G/3G OIR+AAV-Fto PDGFRa┬+IB4.tif]

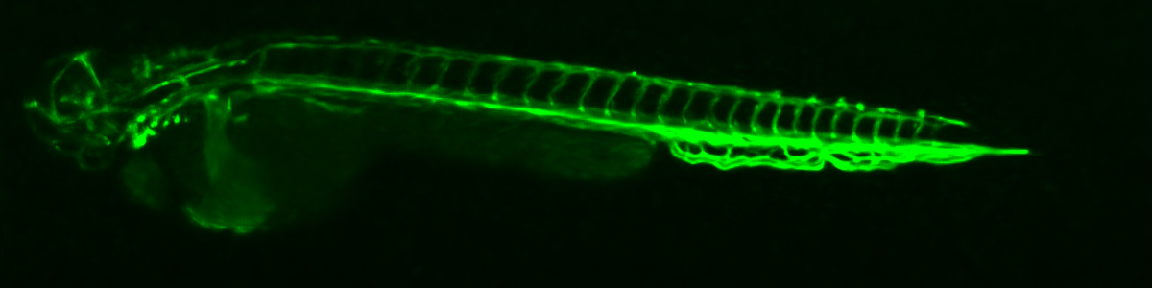

Supplement: Supplementary file 4 — Source Data Fig. 3 [file 44321_2024_25_MOESM4_ESM.zip › figure 3/3I/3I Antisense mRNA 1.tif]

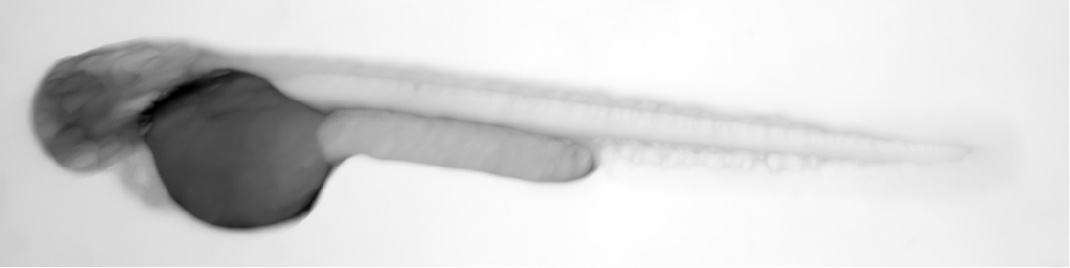

Supplement: Supplementary file 4 — Source Data Fig. 3 [file 44321_2024_25_MOESM4_ESM.zip › figure 3/3I/3I Antisense mRNA 2.tif]

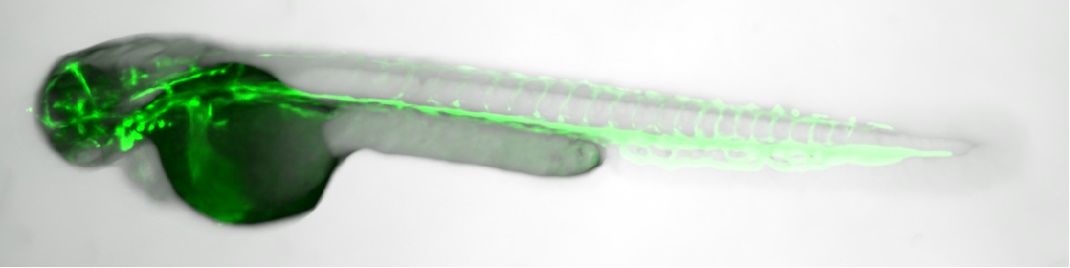

Supplement: Supplementary file 4 — Source Data Fig. 3 [file 44321_2024_25_MOESM4_ESM.zip › figure 3/3I/3I Antisense mRNA 3.tif]

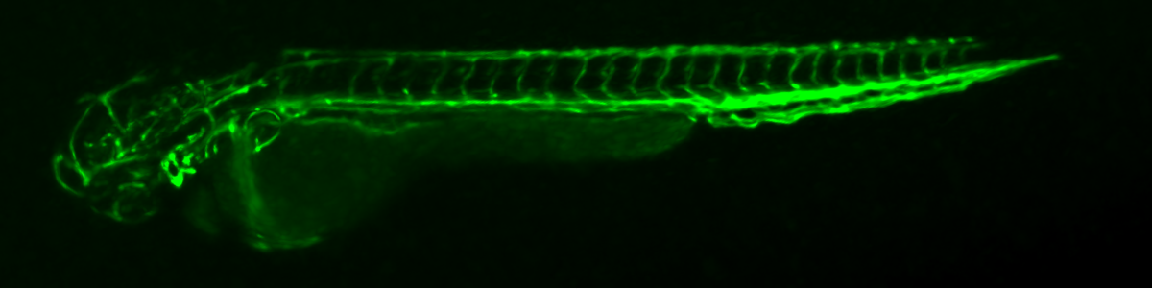

Supplement: Supplementary file 4 — Source Data Fig. 3 [file 44321_2024_25_MOESM4_ESM.zip › figure 3/3I/3I Ctrl 1.tif]

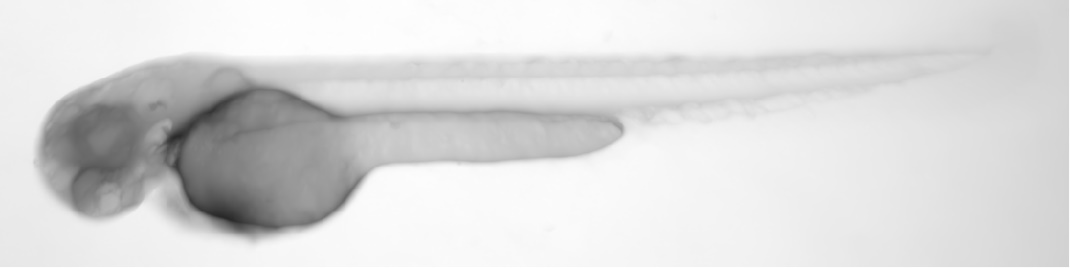

Supplement: Supplementary file 4 — Source Data Fig. 3 [file 44321_2024_25_MOESM4_ESM.zip › figure 3/3I/3I Ctrl 2.tif]

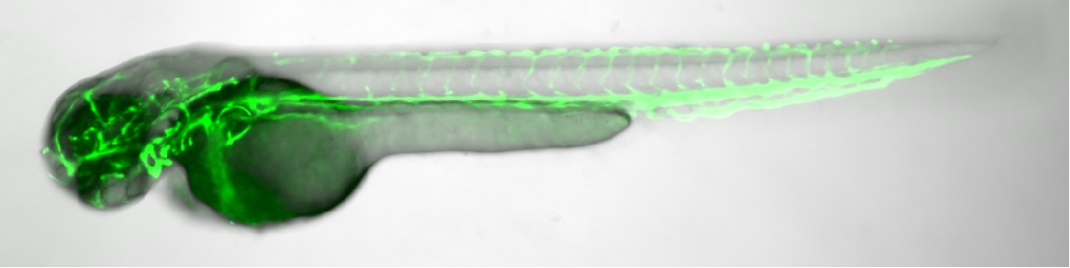

Supplement: Supplementary file 4 — Source Data Fig. 3 [file 44321_2024_25_MOESM4_ESM.zip › figure 3/3I/3I Ctrl 3.tif]

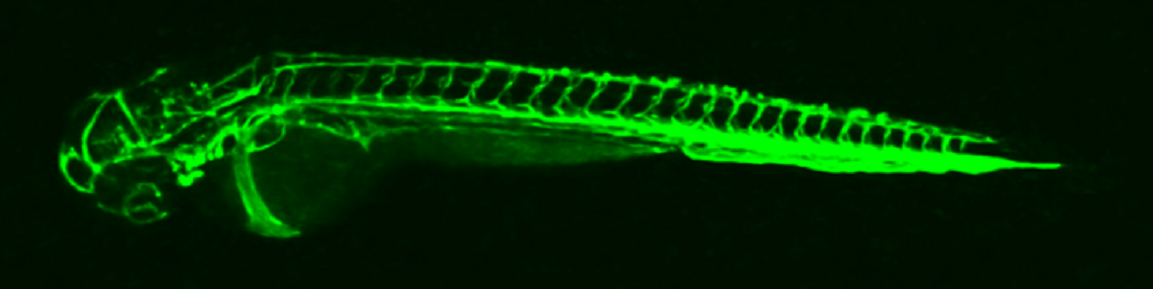

Supplement: Supplementary file 4 — Source Data Fig. 3 [file 44321_2024_25_MOESM4_ESM.zip › figure 3/3I/3I fto mRNA 120ng 1.tif]

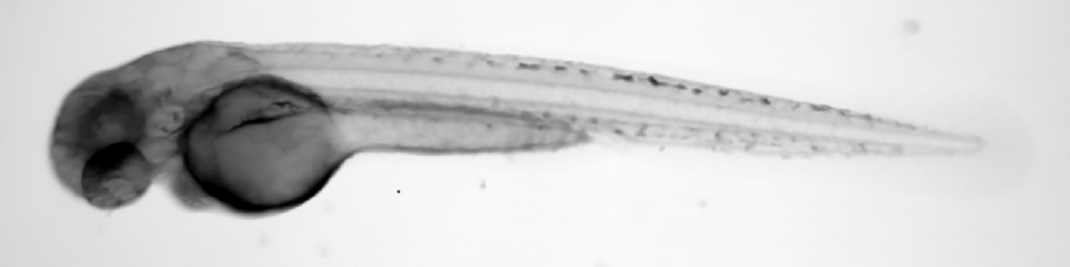

Supplement: Supplementary file 4 — Source Data Fig. 3 [file 44321_2024_25_MOESM4_ESM.zip › figure 3/3I/3I fto mRNA 120ng 2.tif]

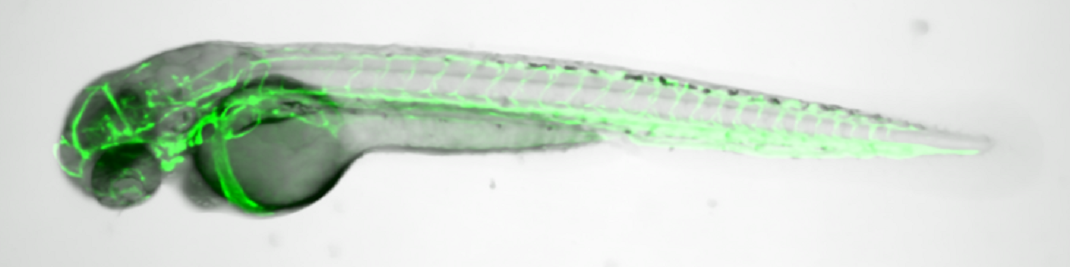

Supplement: Supplementary file 4 — Source Data Fig. 3 [file 44321_2024_25_MOESM4_ESM.zip › figure 3/3I/3I fto mRNA 120ng 3.tif]

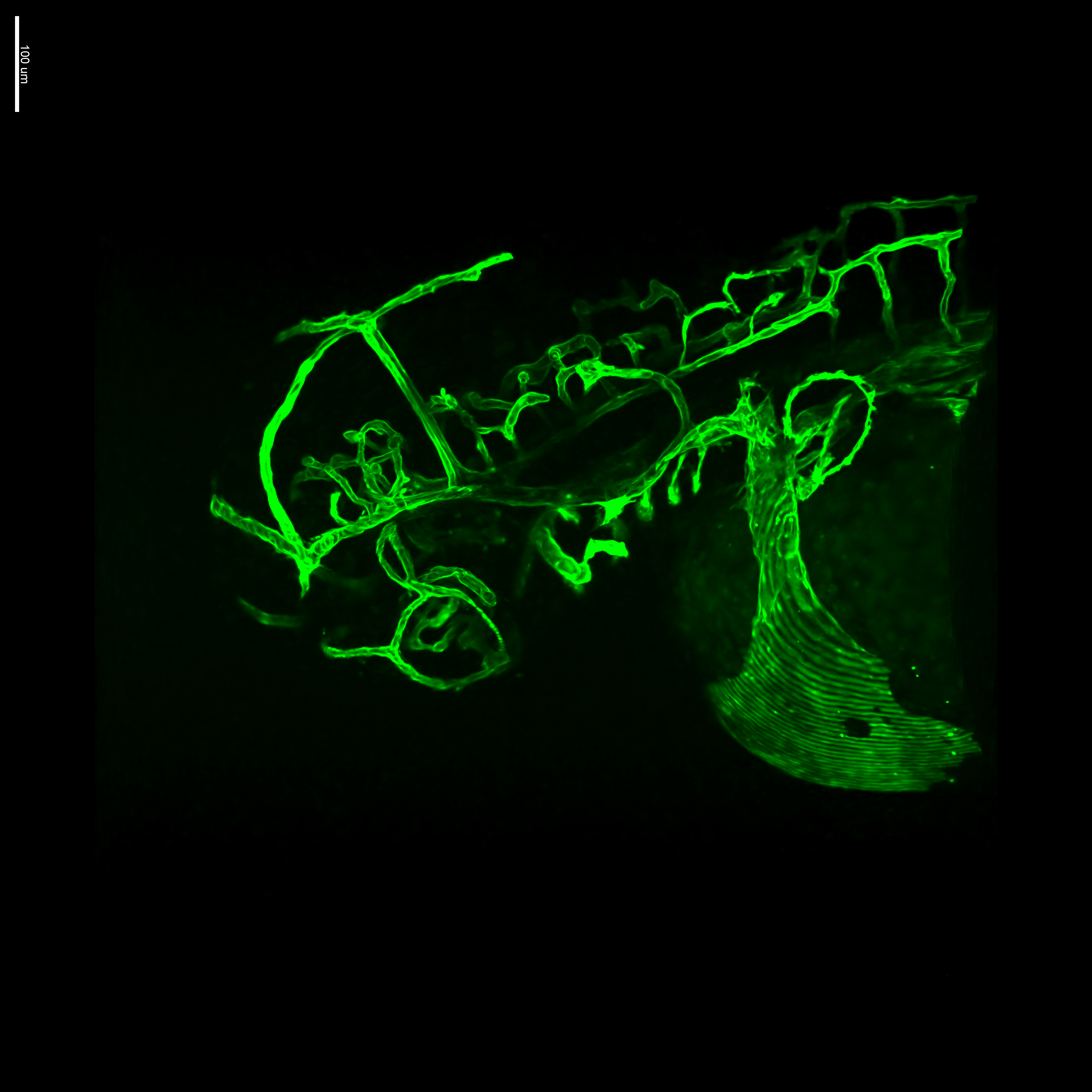

Supplement: Supplementary file 4 — Source Data Fig. 3 [file 44321_2024_25_MOESM4_ESM.zip › figure 3/3J/3J 3% glucose lower line.tif]

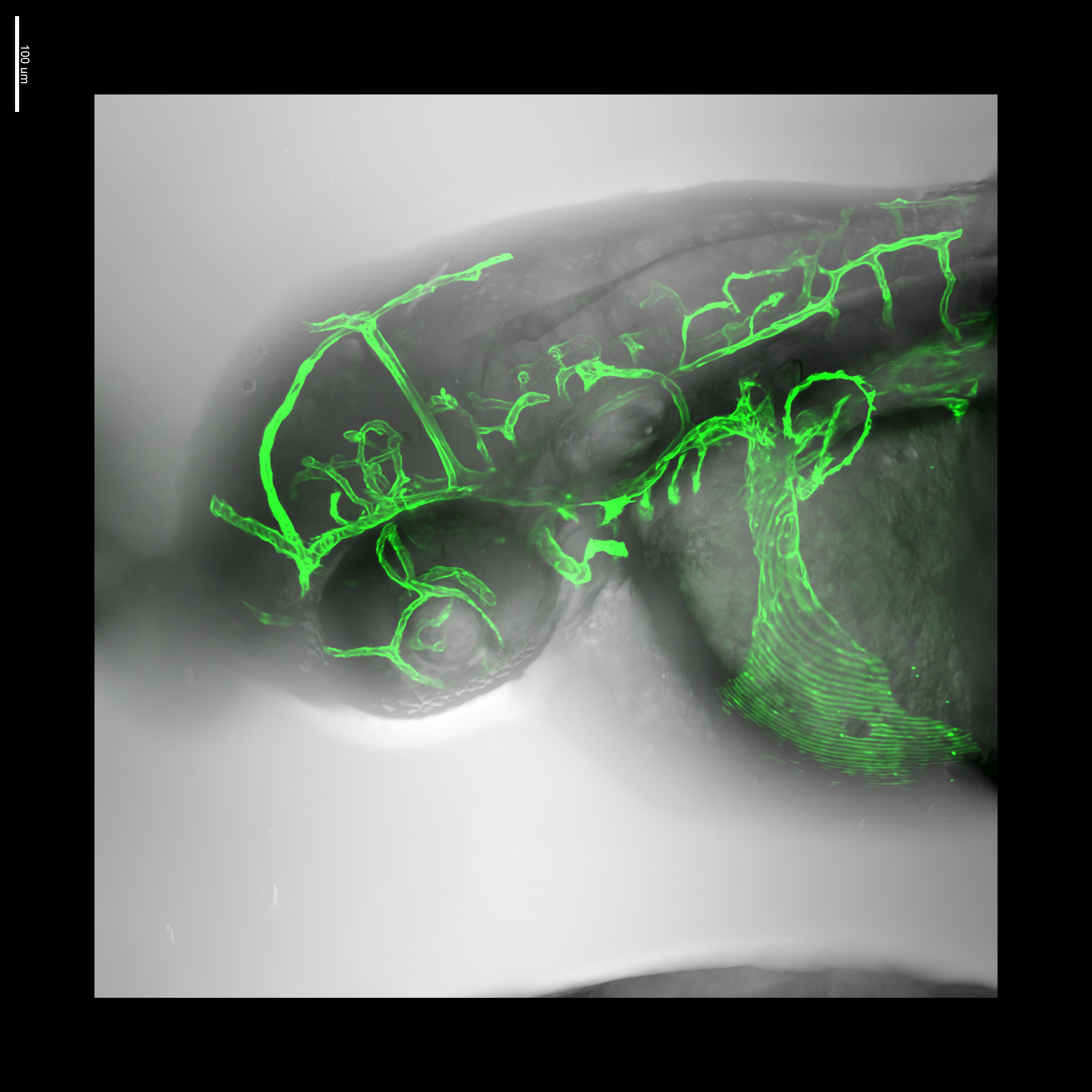

Supplement: Supplementary file 4 — Source Data Fig. 3 [file 44321_2024_25_MOESM4_ESM.zip › figure 3/3J/3J 3% glucose upper line.tif]

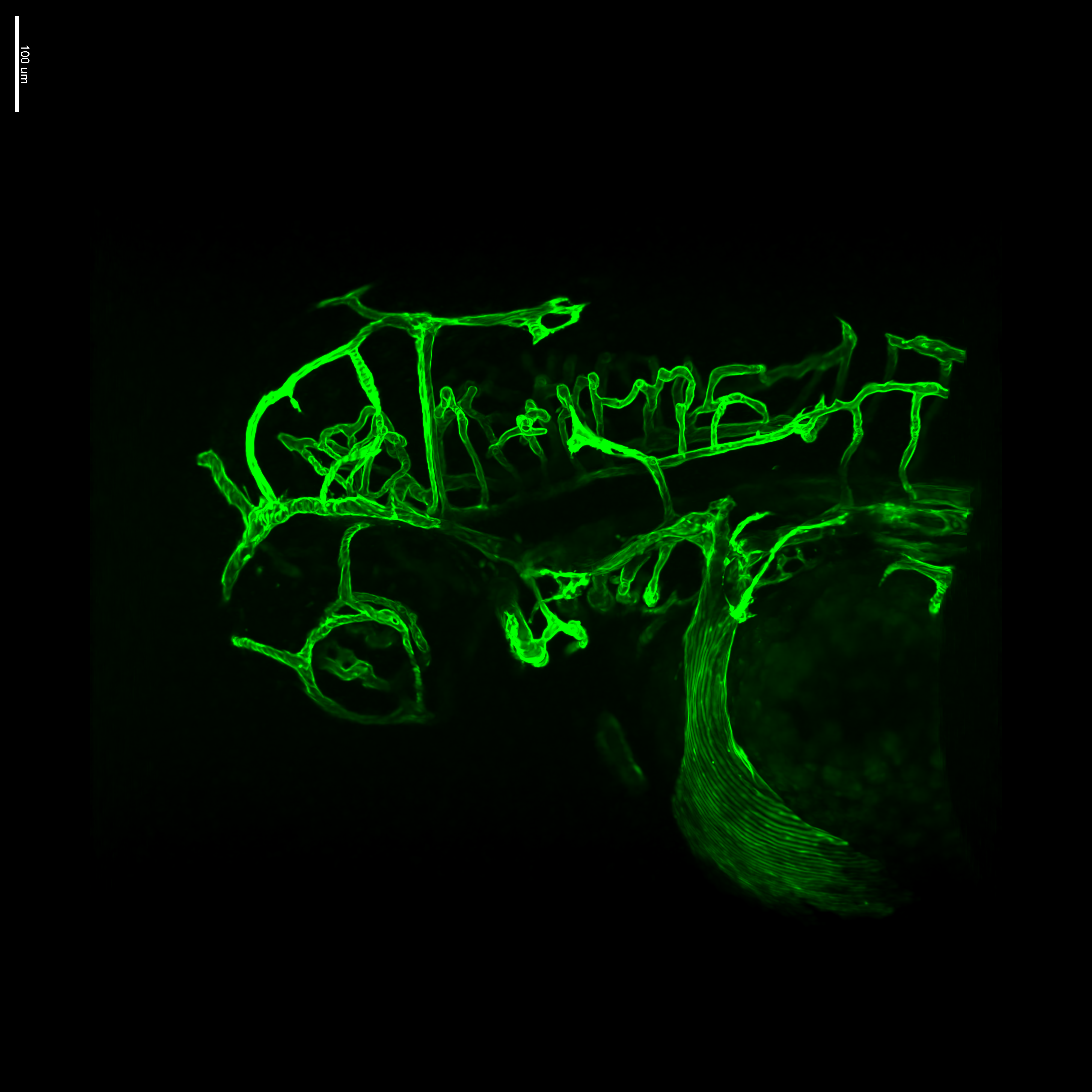

Supplement: Supplementary file 4 — Source Data Fig. 3 [file 44321_2024_25_MOESM4_ESM.zip › figure 3/3J/3J Ctrl lower line.tif]

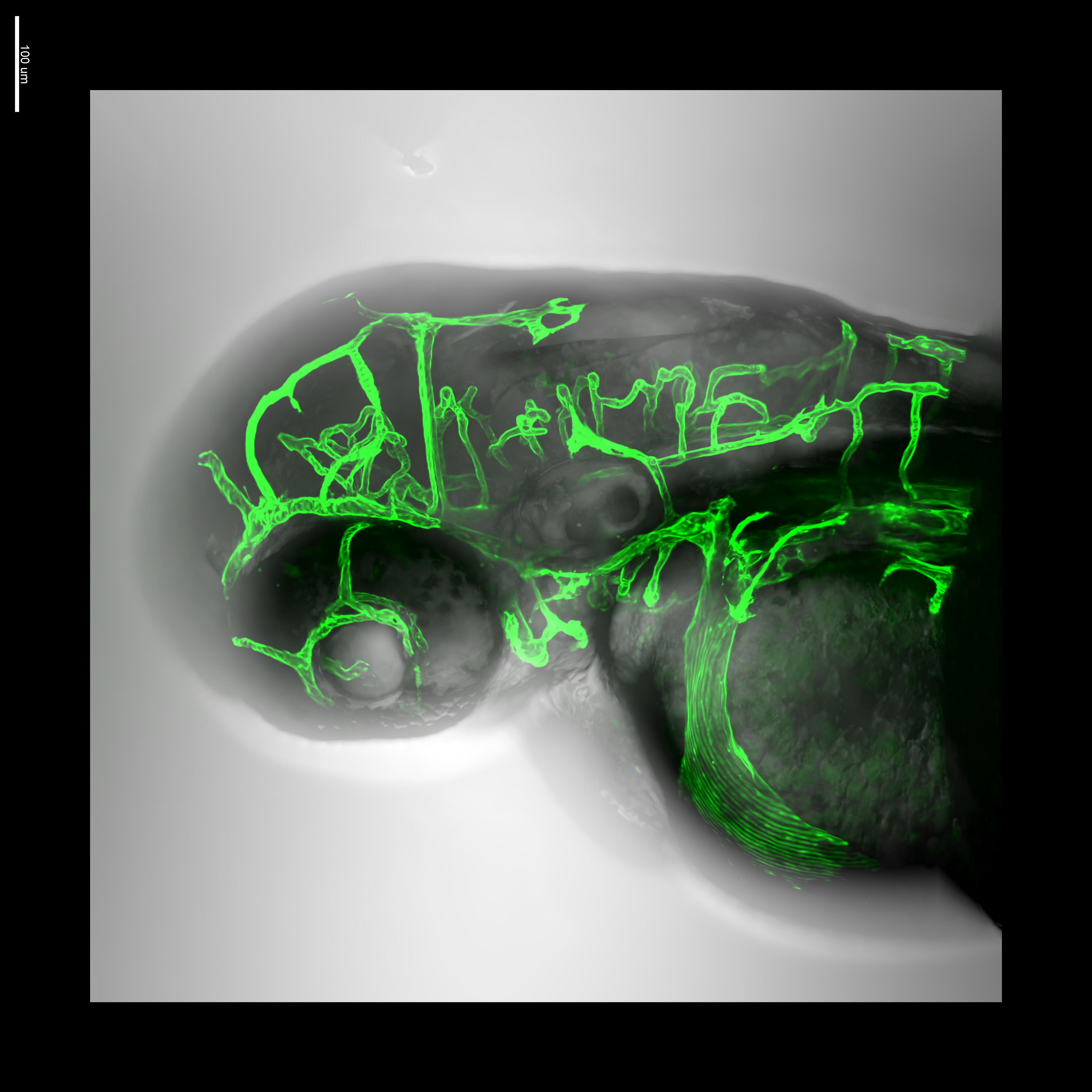

Supplement: Supplementary file 4 — Source Data Fig. 3 [file 44321_2024_25_MOESM4_ESM.zip › figure 3/3J/3J Ctrl upper line.tif]

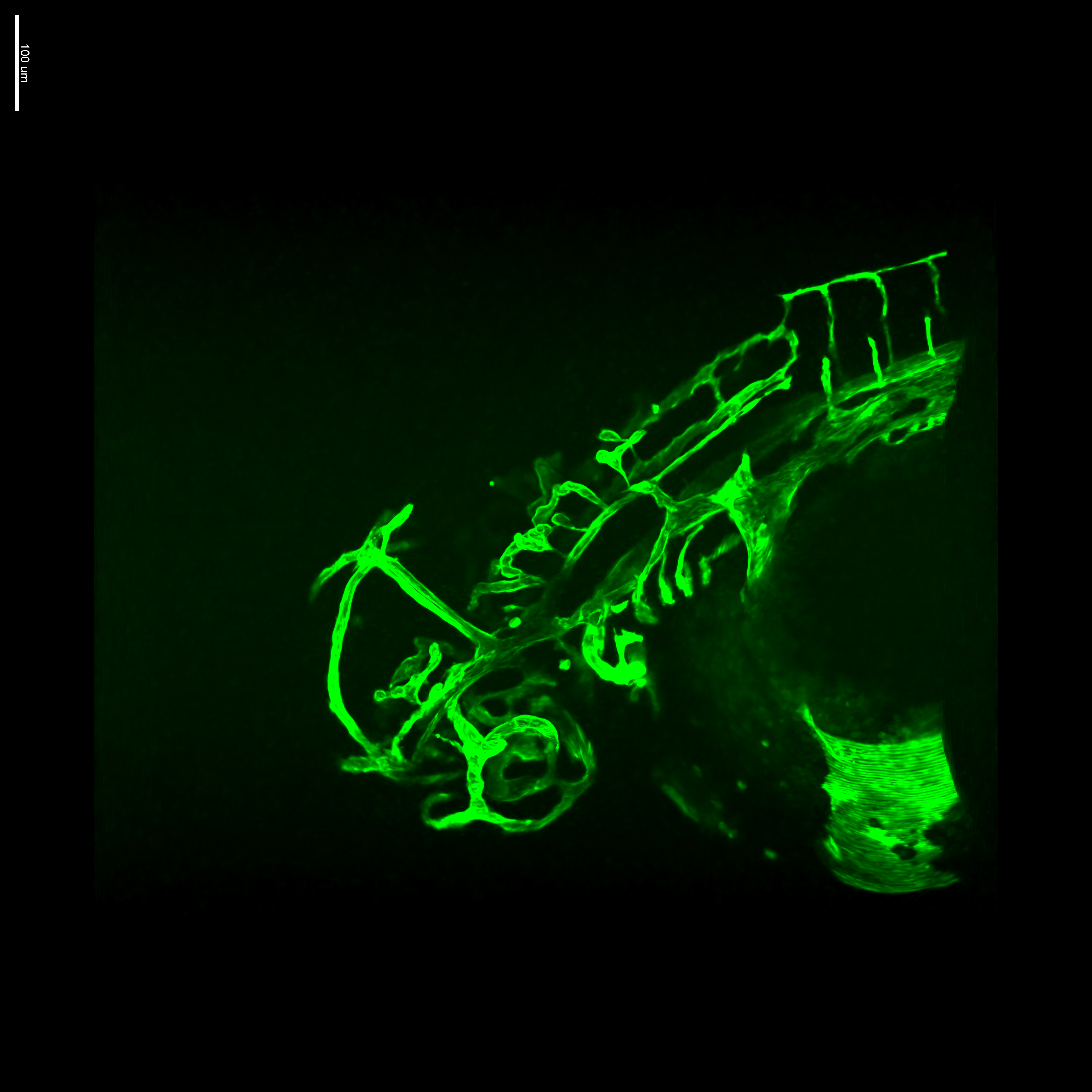

Supplement: Supplementary file 4 — Source Data Fig. 3 [file 44321_2024_25_MOESM4_ESM.zip › figure 3/3J/3J fto mRNA+3% glucose lower line.tif]

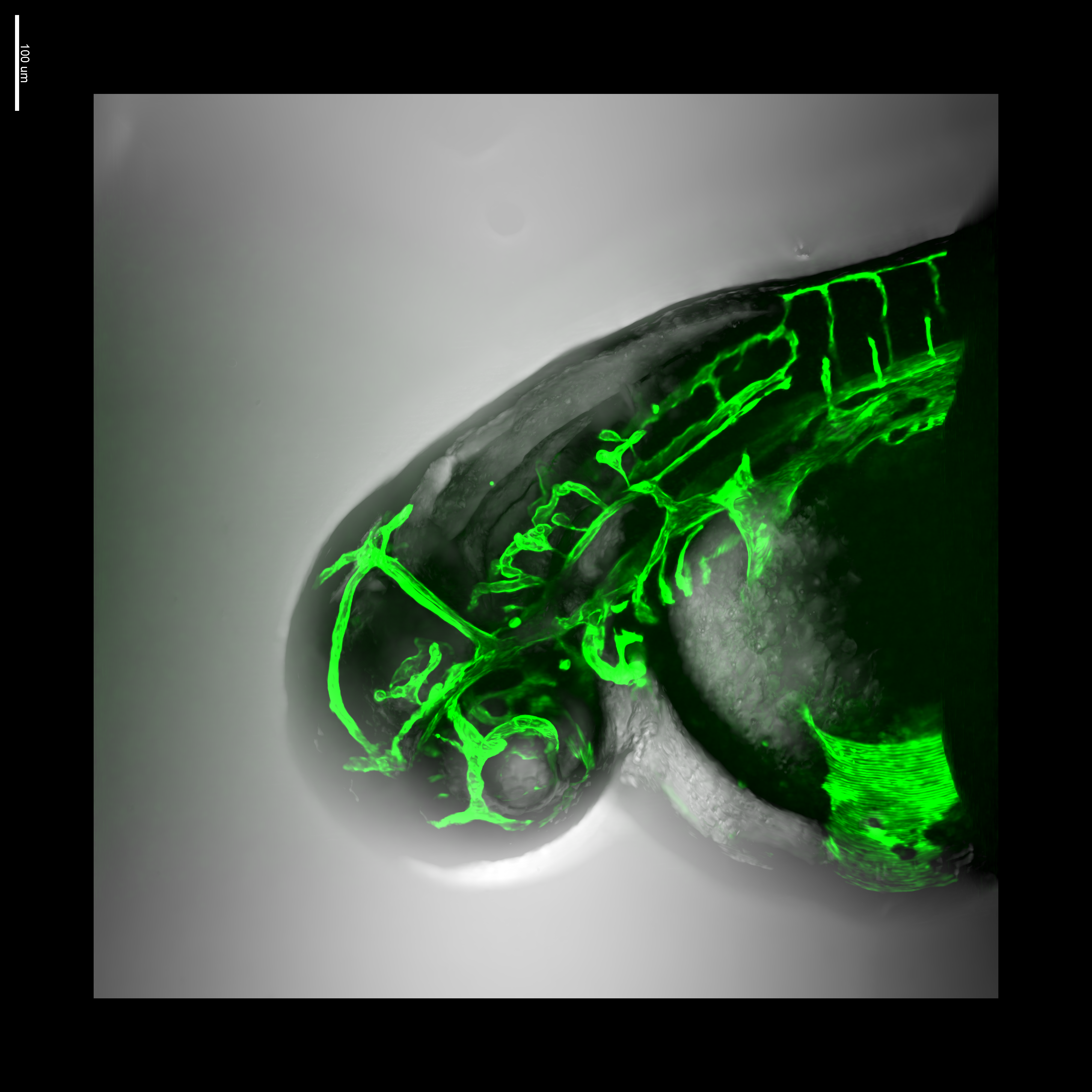

Supplement: Supplementary file 4 — Source Data Fig. 3 [file 44321_2024_25_MOESM4_ESM.zip › figure 3/3J/3J fto mRNA+3% glucose upper line.tif]

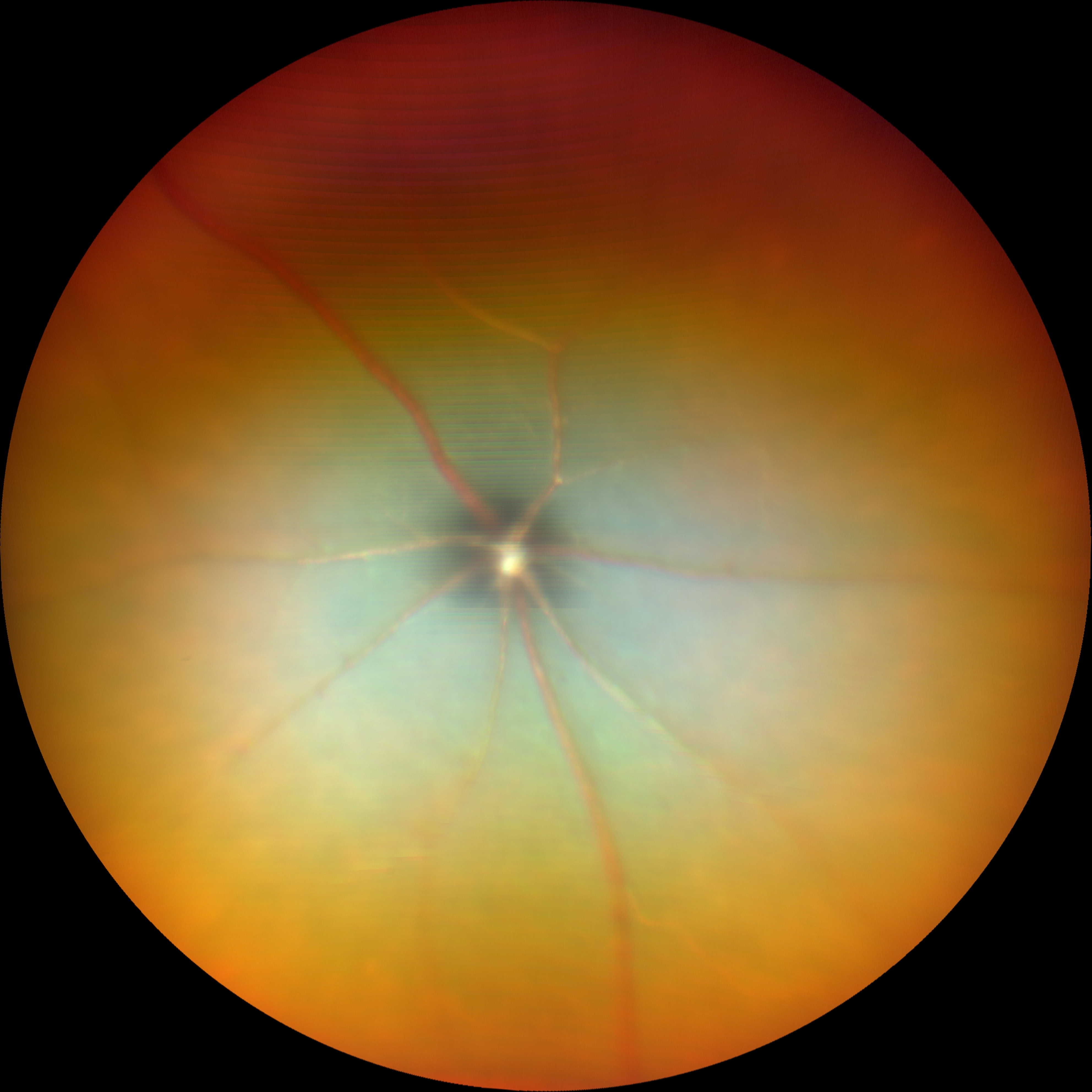

Supplement: Supplementary file 5 — Source Data Fig. 4 [file 44321_2024_25_MOESM5_ESM.zip › figure 4/4B/4B Ctrl 3.5M.tif]

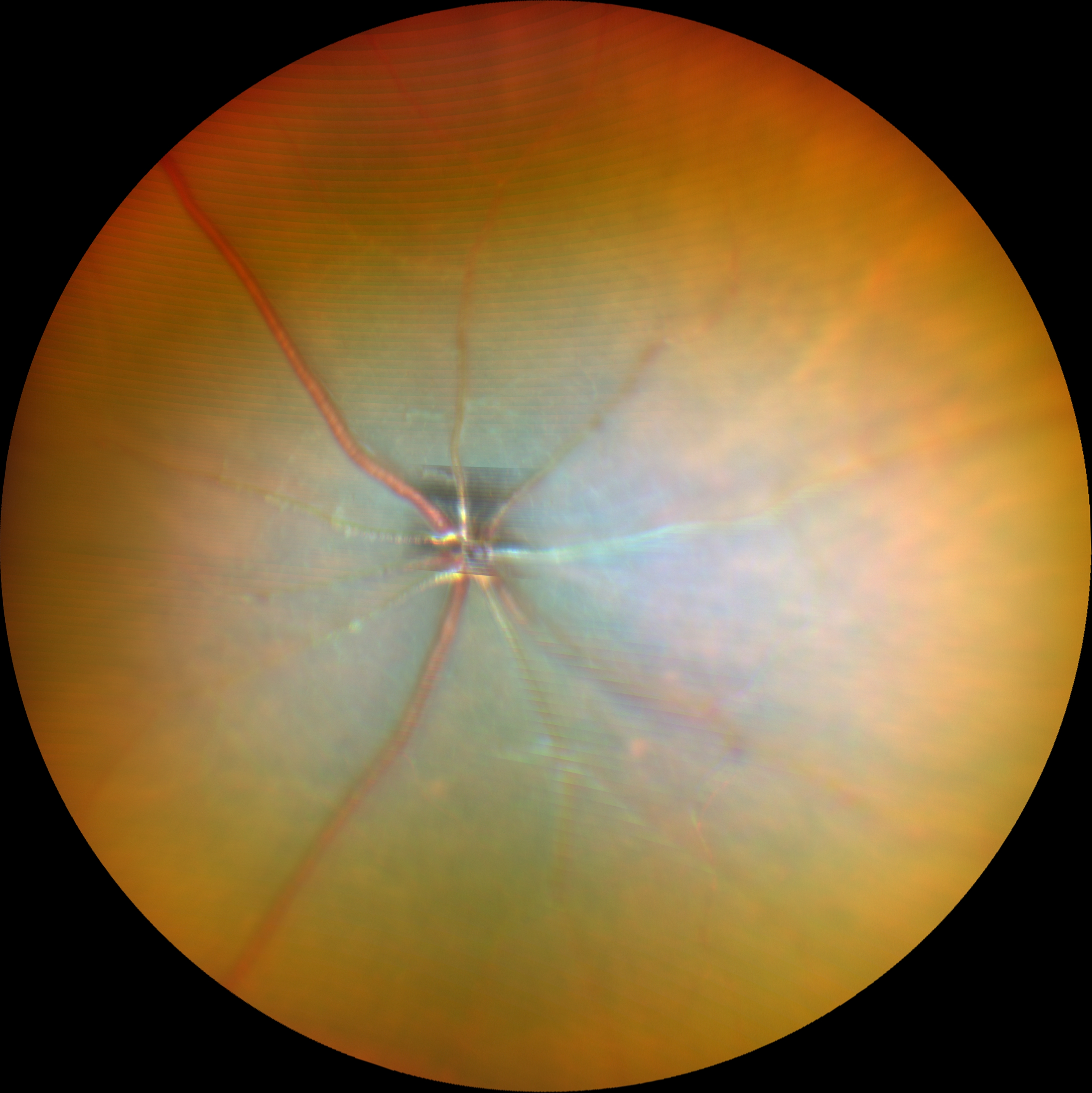

Supplement: Supplementary file 5 — Source Data Fig. 4 [file 44321_2024_25_MOESM5_ESM.zip › figure 4/4B/4B Ctrl 5M.tif]

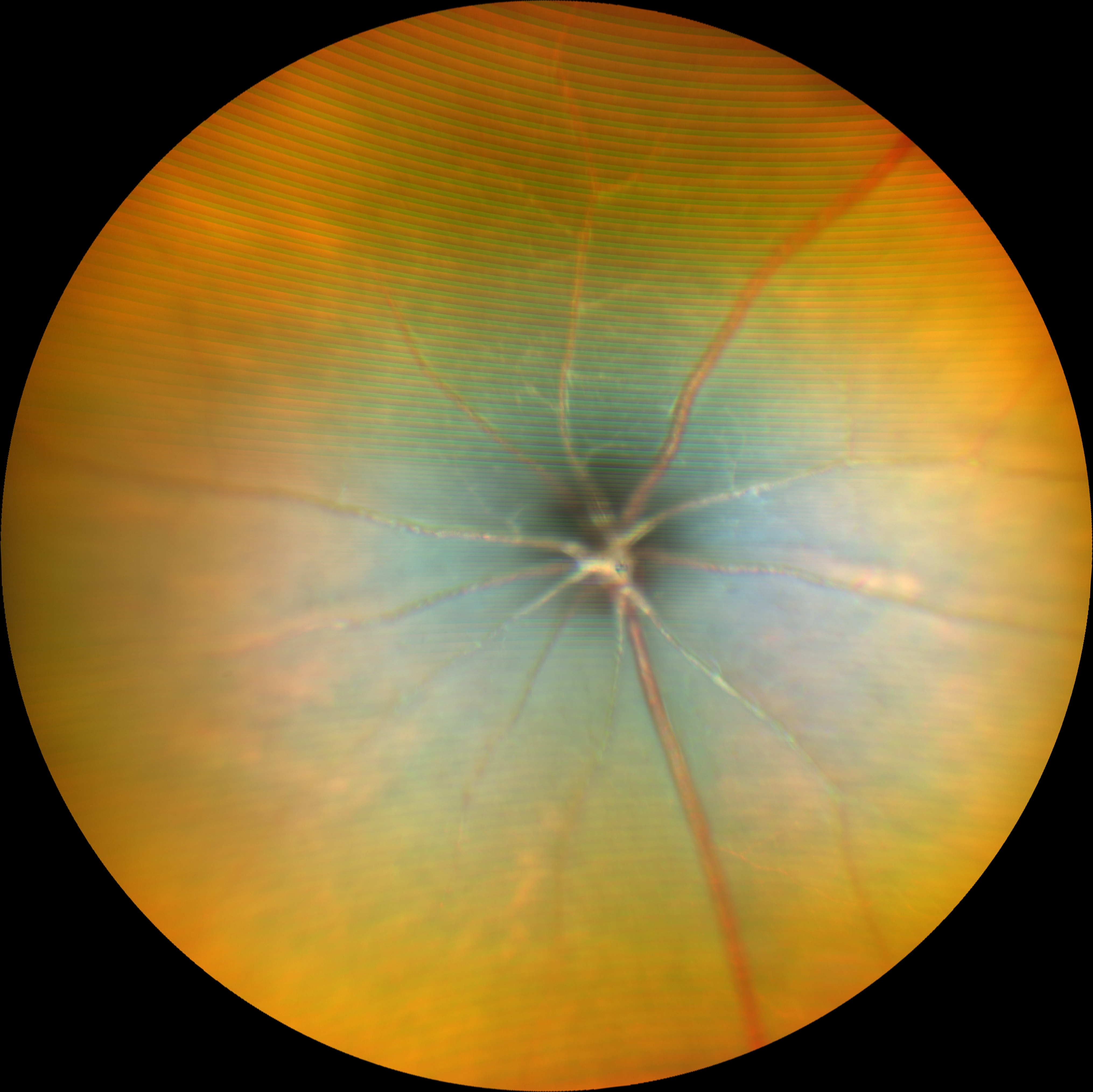

Supplement: Supplementary file 5 — Source Data Fig. 4 [file 44321_2024_25_MOESM5_ESM.zip › figure 4/4B/4B STZ 3.5M.tif]

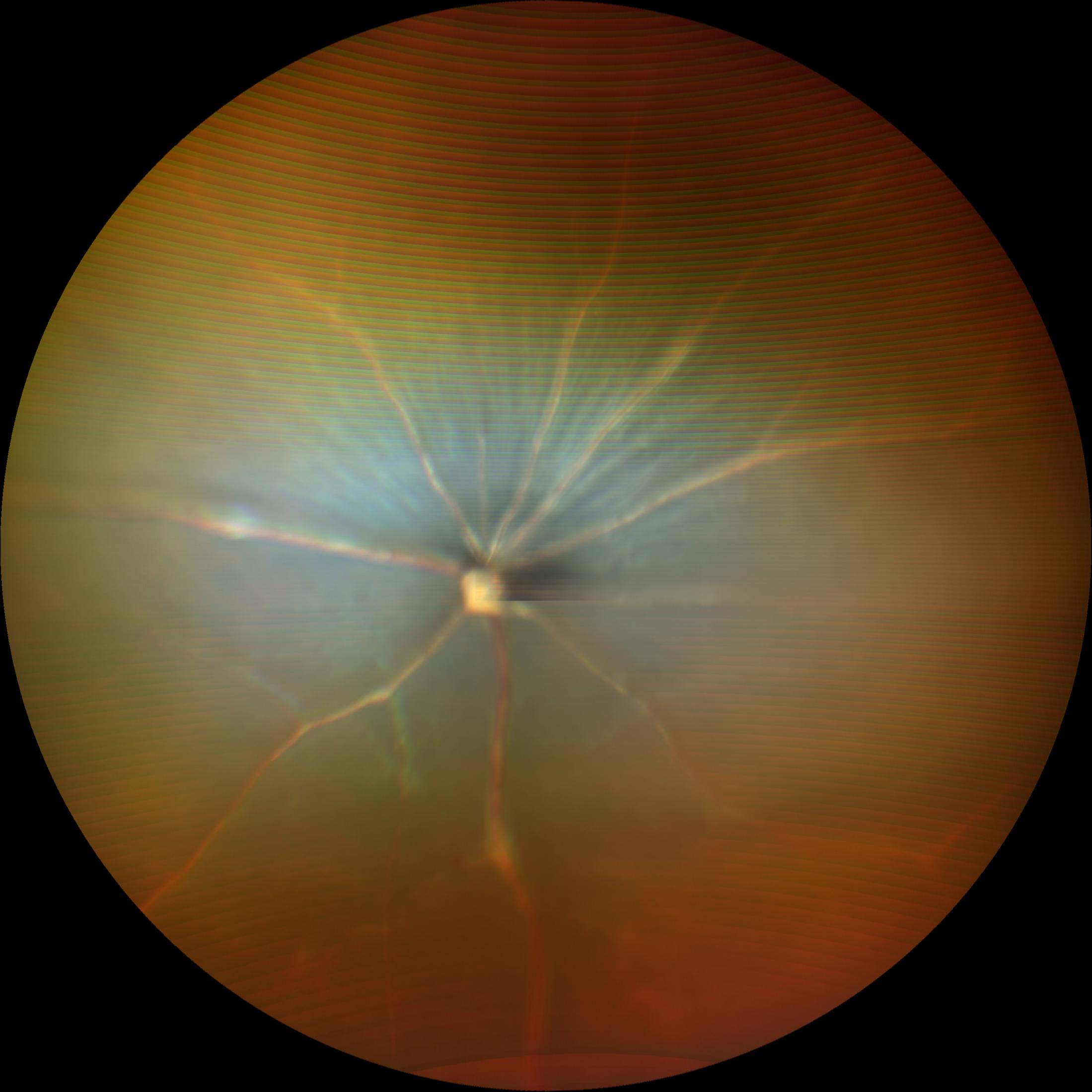

Supplement: Supplementary file 5 — Source Data Fig. 4 [file 44321_2024_25_MOESM5_ESM.zip › figure 4/4B/4B STZ 5M.tif]

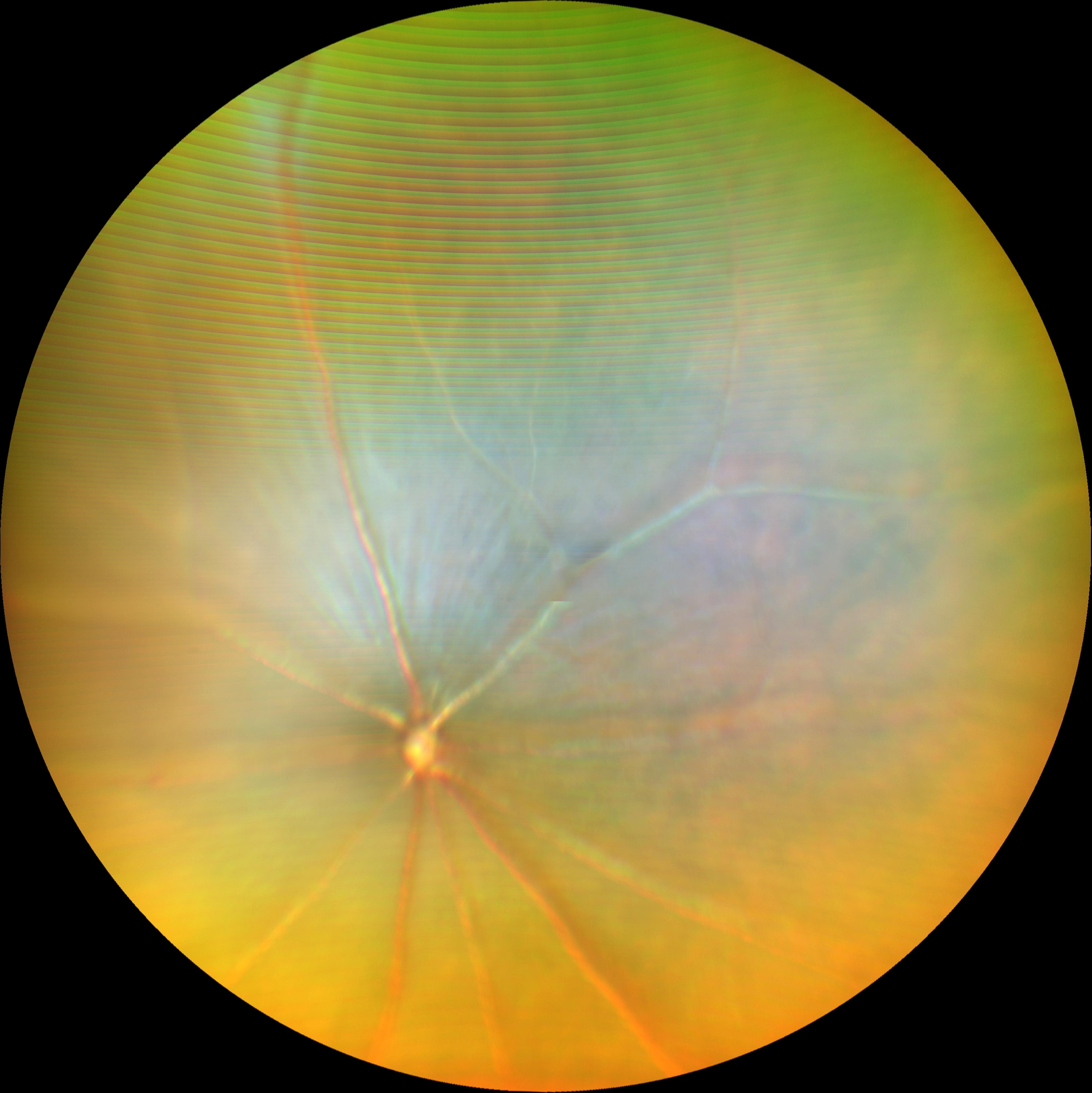

Supplement: Supplementary file 5 — Source Data Fig. 4 [file 44321_2024_25_MOESM5_ESM.zip › figure 4/4B/4B STZ+AAV-blank 3.5M.tif]

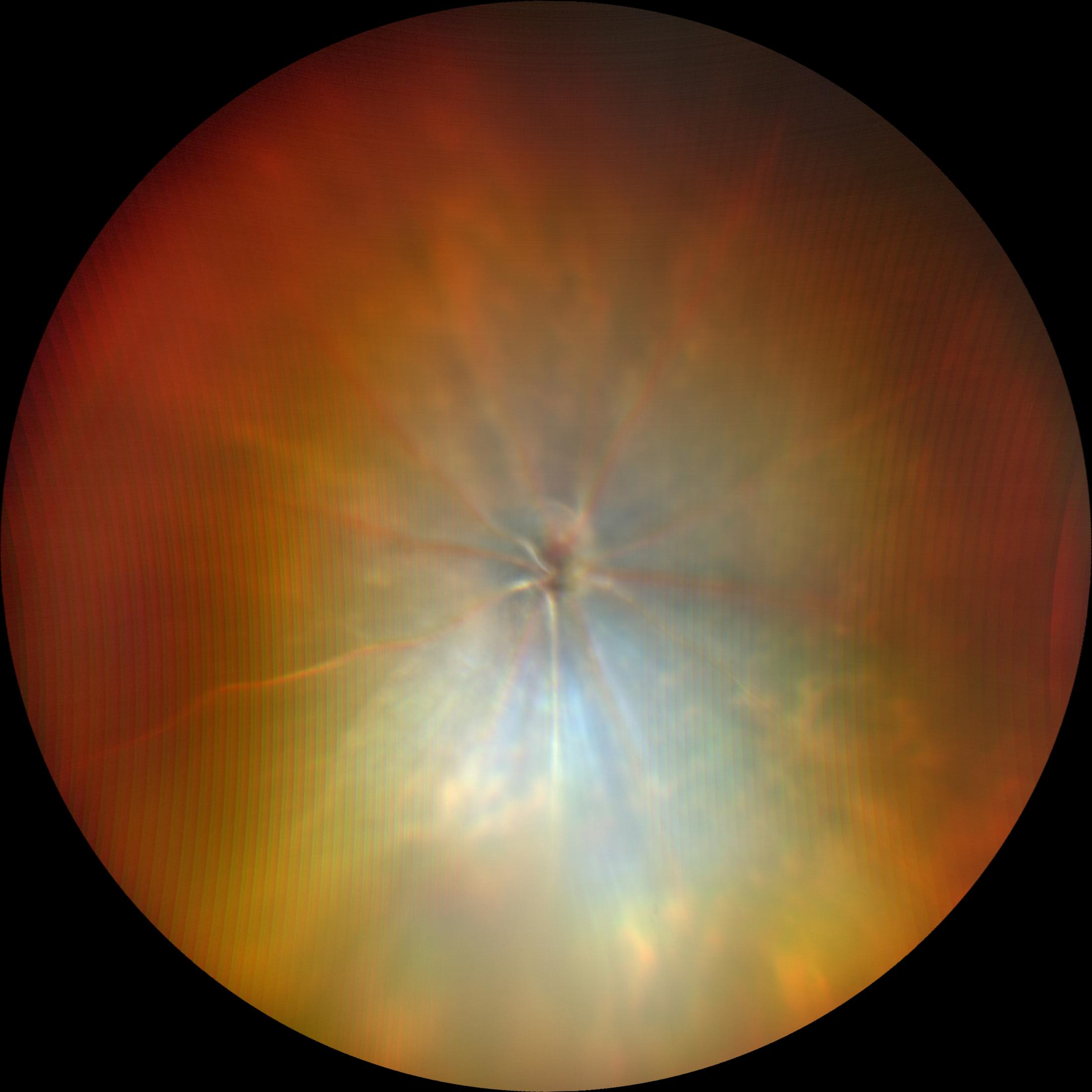

Supplement: Supplementary file 5 — Source Data Fig. 4 [file 44321_2024_25_MOESM5_ESM.zip › figure 4/4B/4B STZ+AAV-blank 5M.tif]

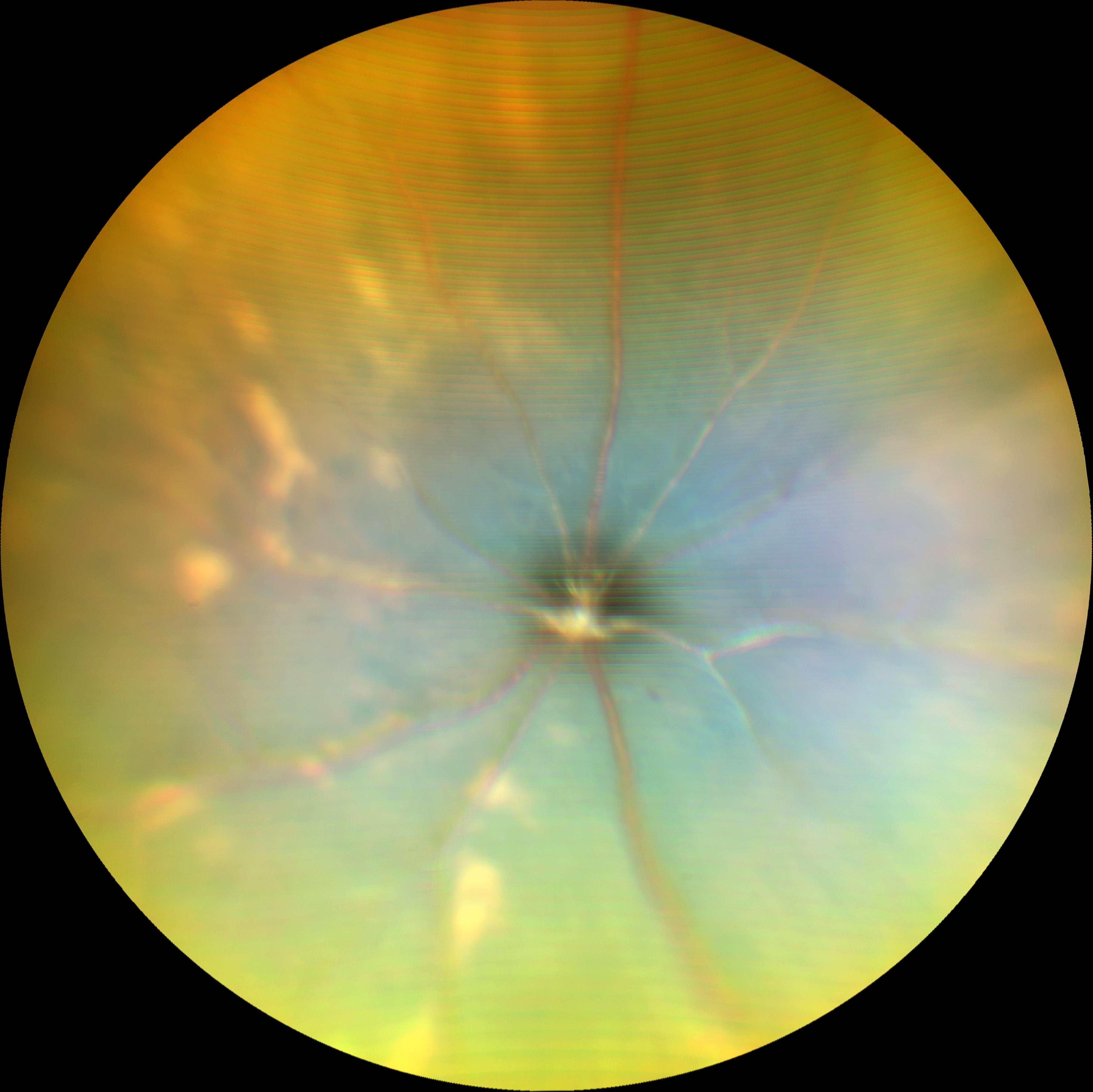

Supplement: Supplementary file 5 — Source Data Fig. 4 [file 44321_2024_25_MOESM5_ESM.zip › figure 4/4B/4B STZ+AAV-Fto 3.5M.tif]

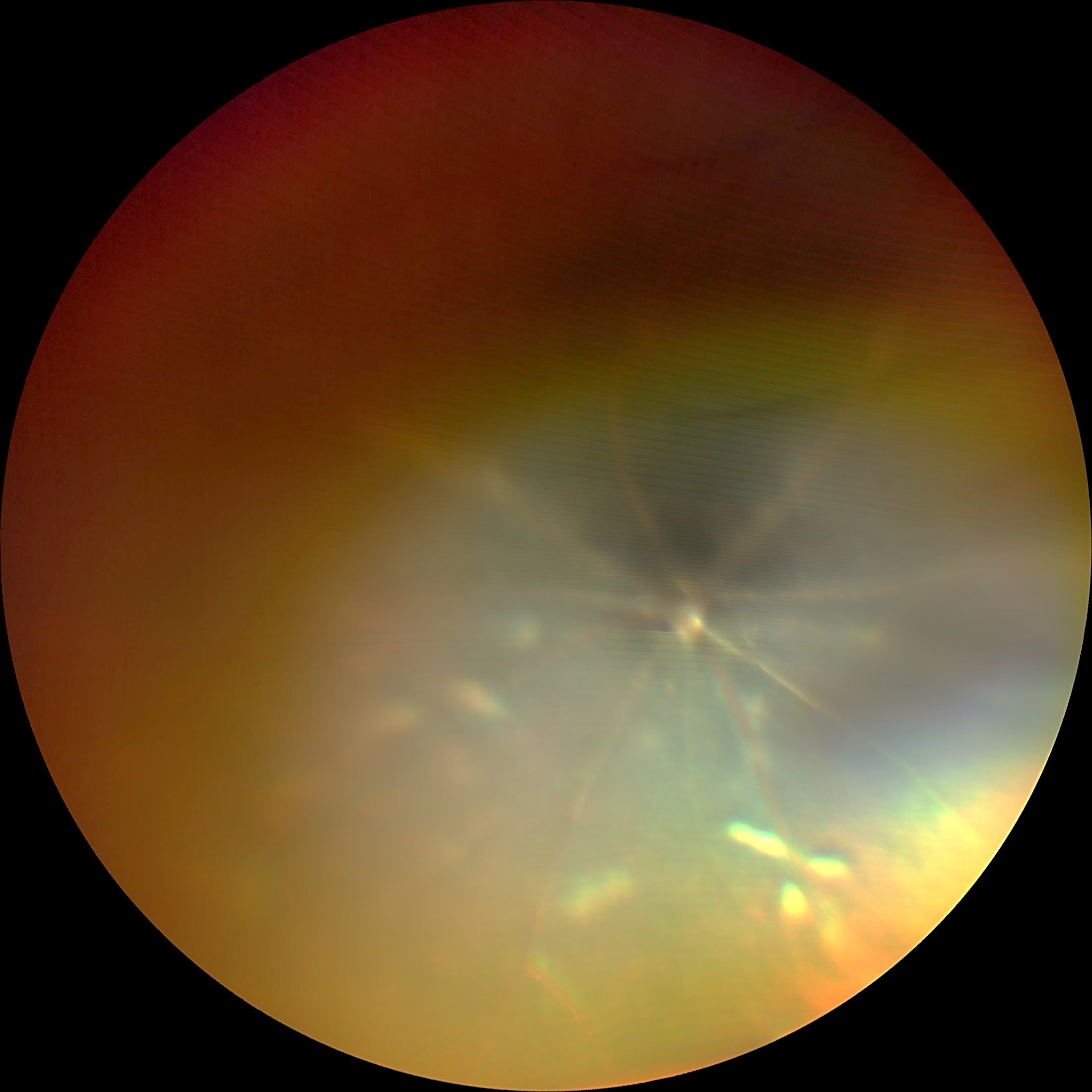

Supplement: Supplementary file 5 — Source Data Fig. 4 [file 44321_2024_25_MOESM5_ESM.zip › figure 4/4B/4B STZ+AAV-Fto 5M.tif]

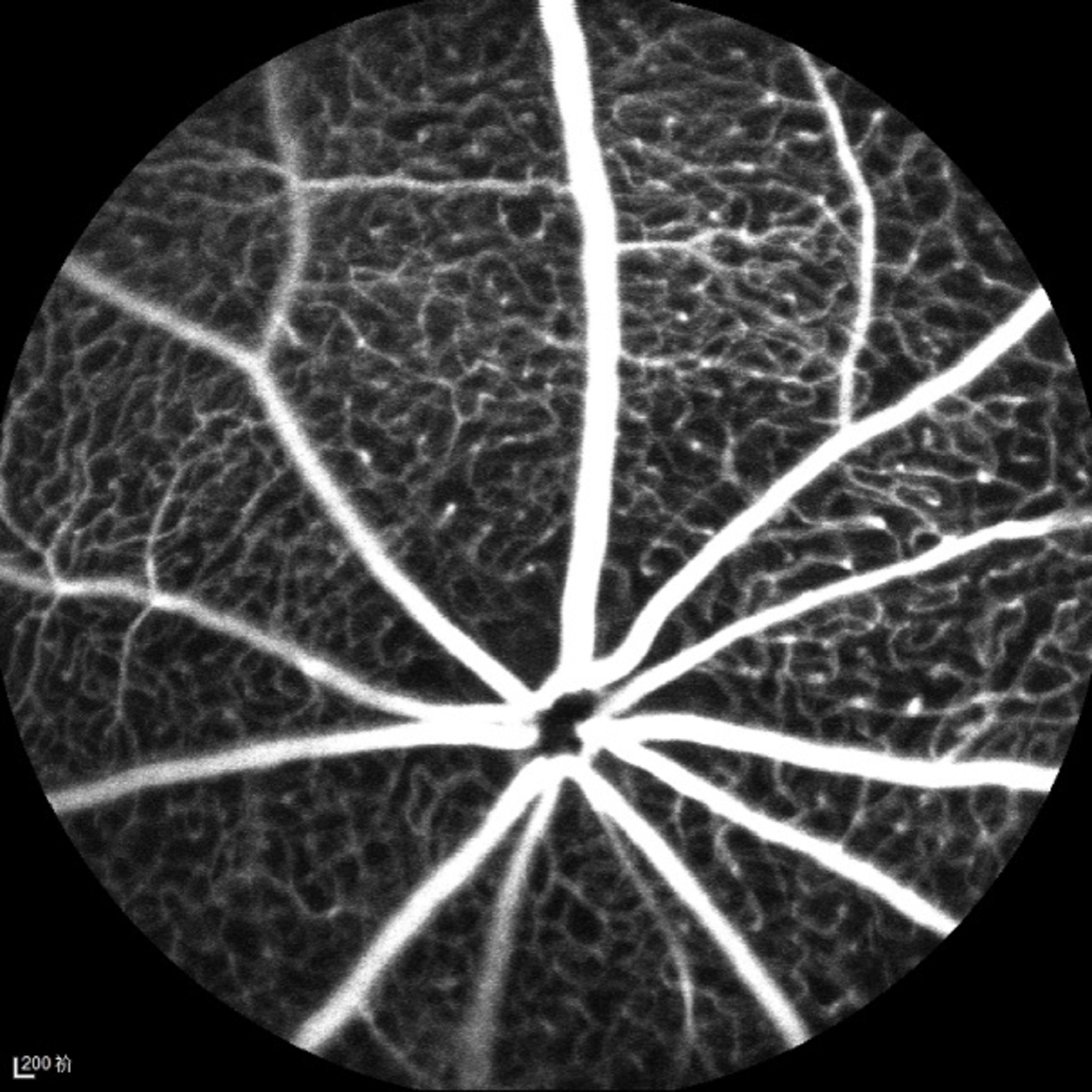

Supplement: Supplementary file 5 — Source Data Fig. 4 [file 44321_2024_25_MOESM5_ESM.zip › figure 4/4C/4C Ctrl.tif]

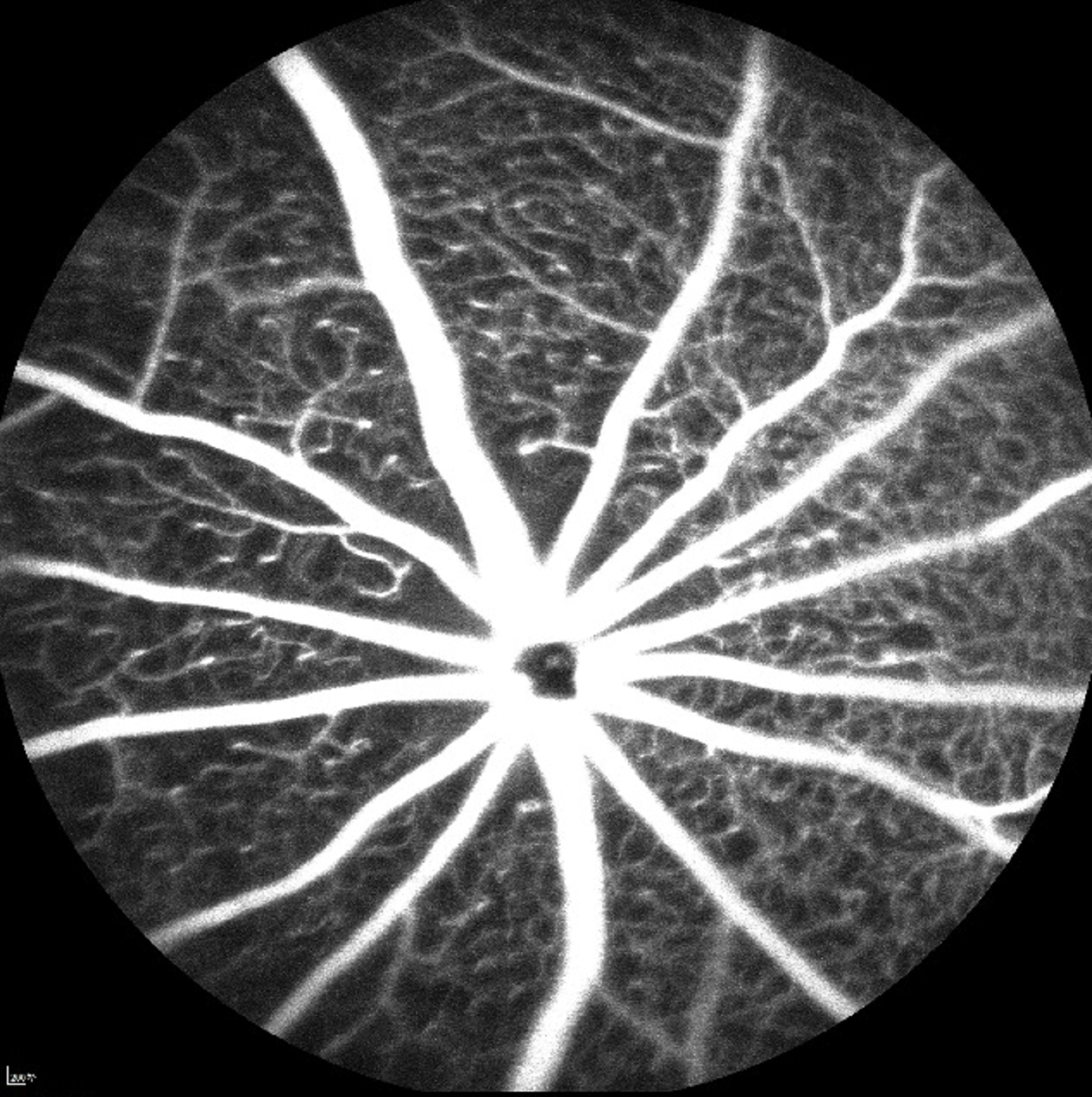

Supplement: Supplementary file 5 — Source Data Fig. 4 [file 44321_2024_25_MOESM5_ESM.zip › figure 4/4C/4C STZ.tif]

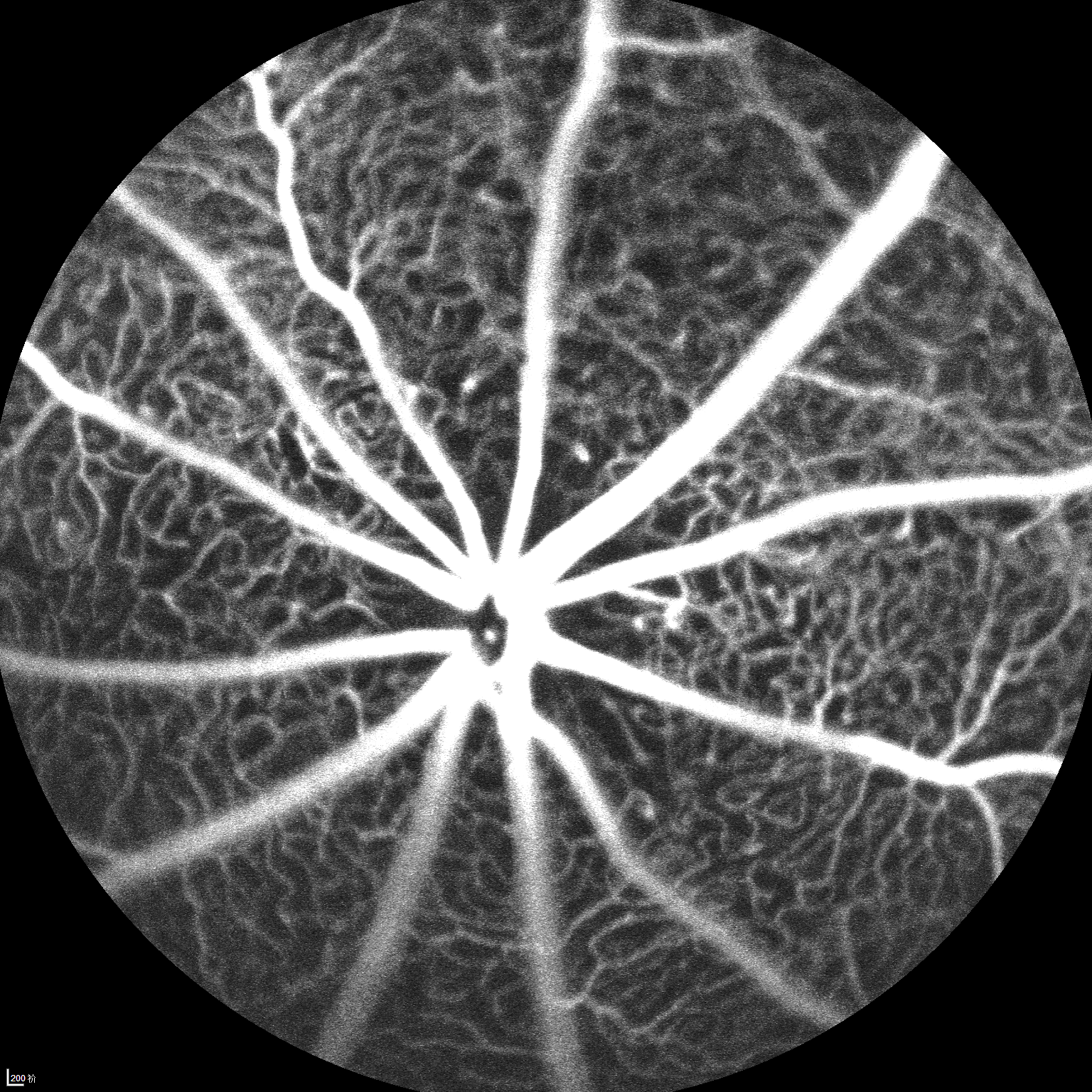

Supplement: Supplementary file 5 — Source Data Fig. 4 [file 44321_2024_25_MOESM5_ESM.zip › figure 4/4C/4C STZ+AAV-blank.tif]

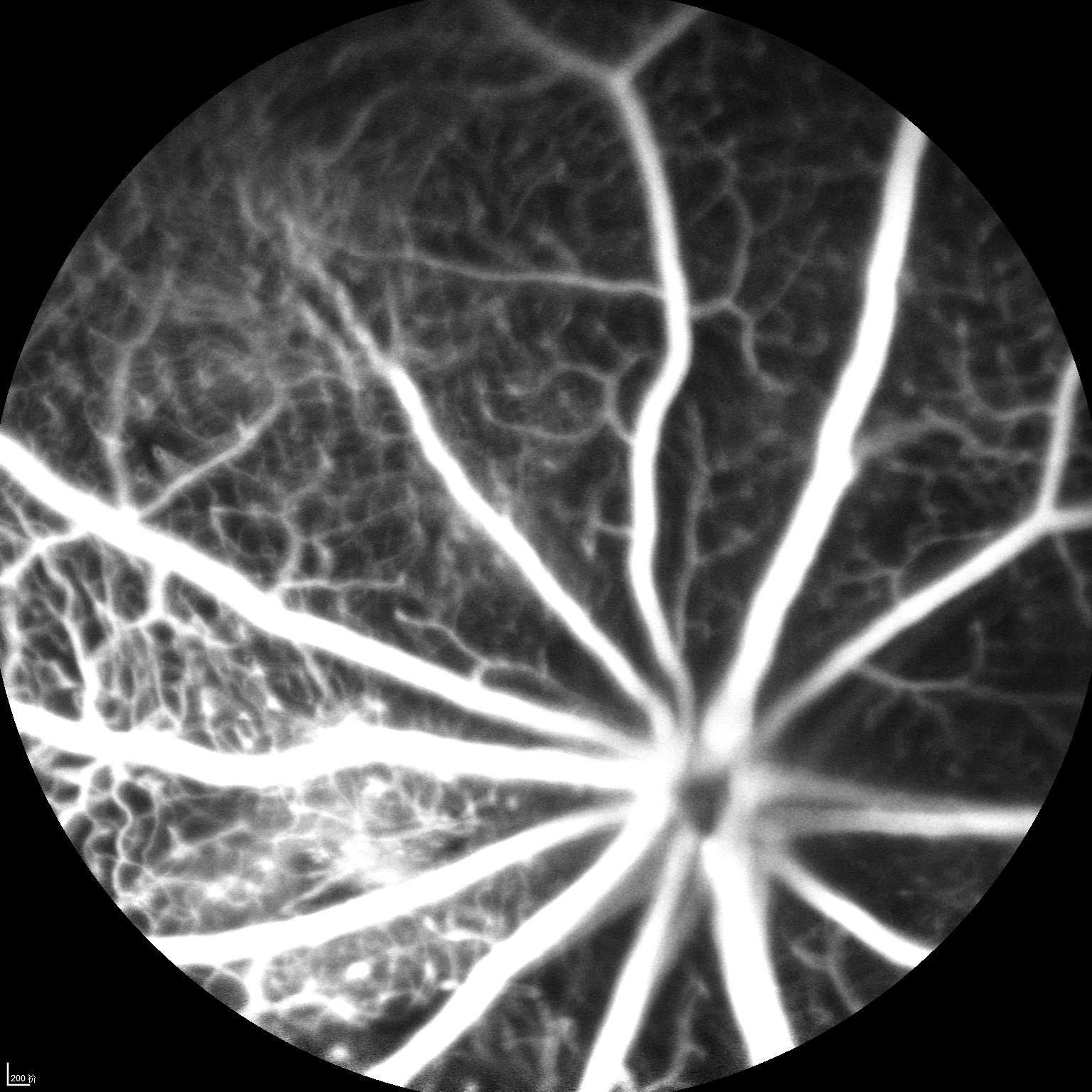

Supplement: Supplementary file 5 — Source Data Fig. 4 [file 44321_2024_25_MOESM5_ESM.zip › figure 4/4C/4C STZ+AAV-Fto.tif]

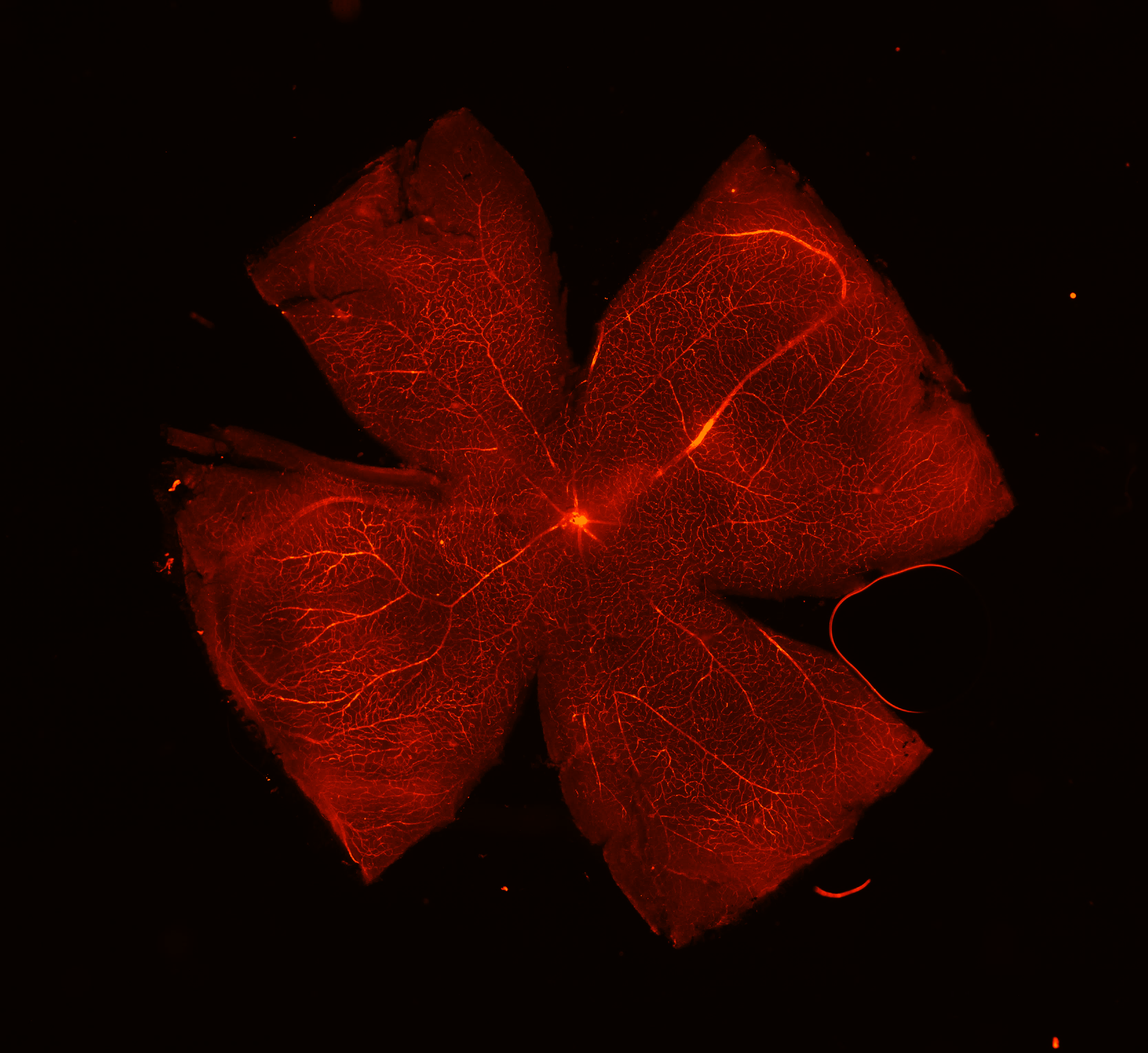

Supplement: Supplementary file 5 — Source Data Fig. 4 [file 44321_2024_25_MOESM5_ESM.zip › figure 4/4D/4D Ctrl.tif]

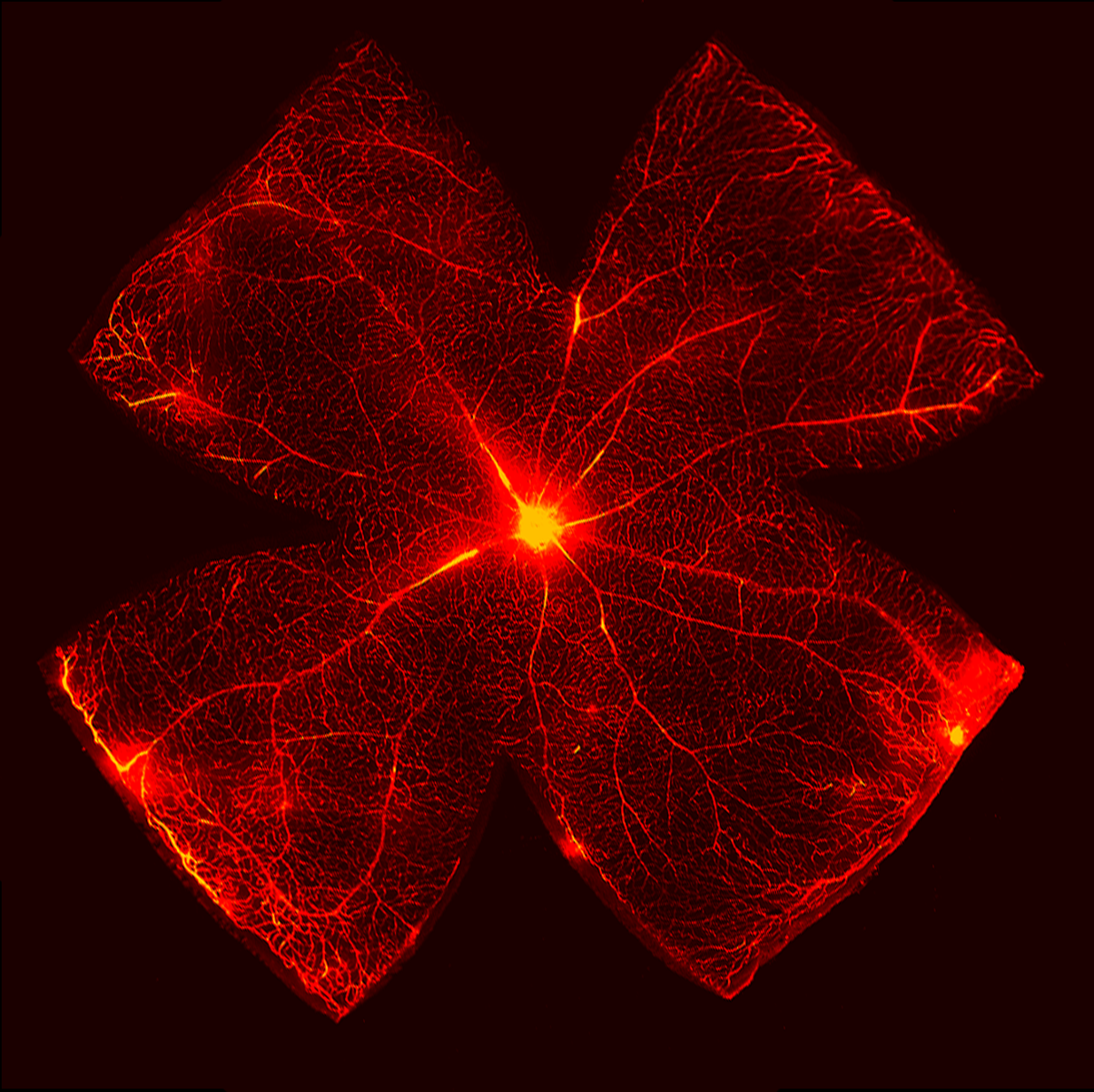

Supplement: Supplementary file 5 — Source Data Fig. 4 [file 44321_2024_25_MOESM5_ESM.zip › figure 4/4D/4D STZ.tif]

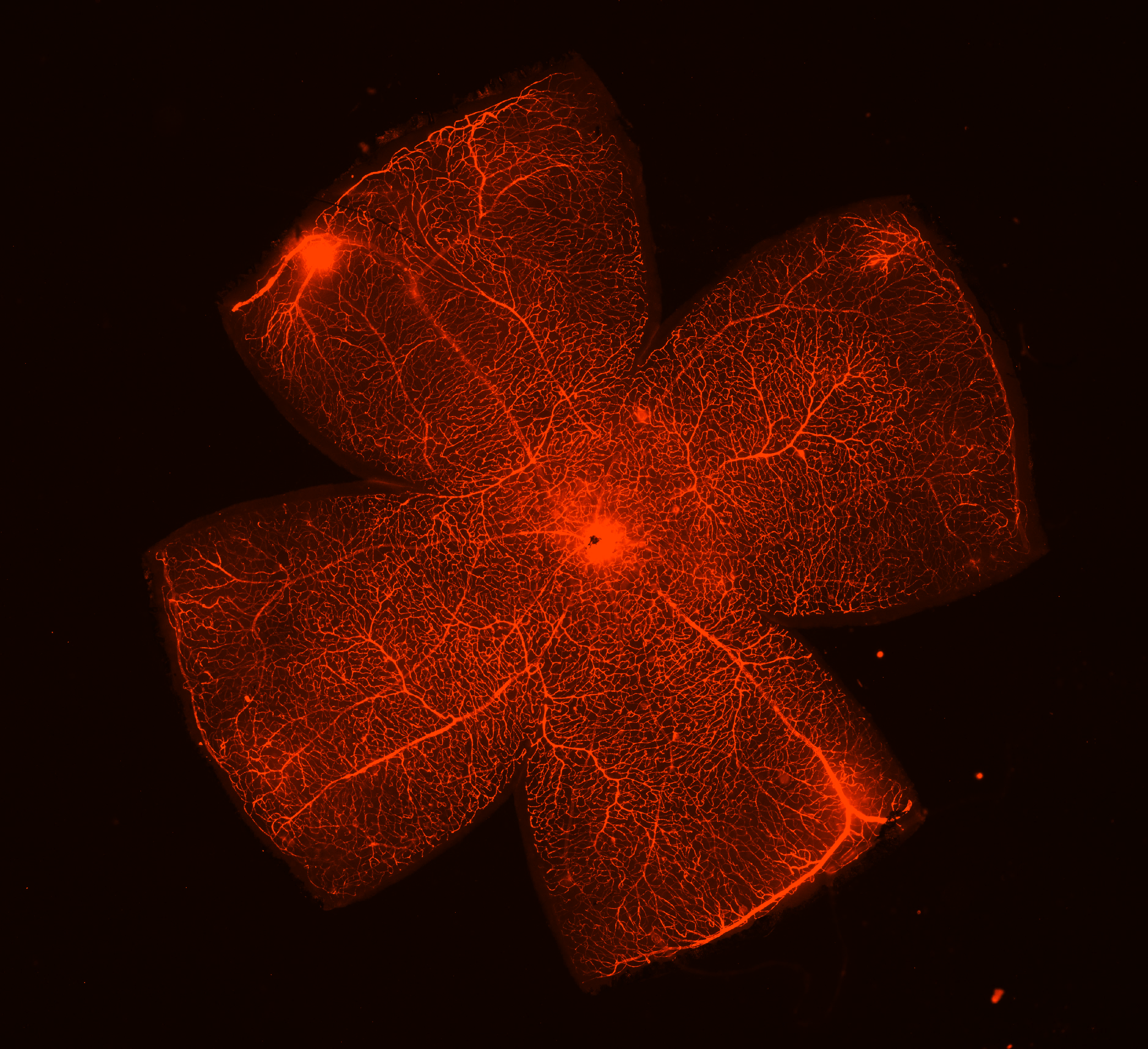

Supplement: Supplementary file 5 — Source Data Fig. 4 [file 44321_2024_25_MOESM5_ESM.zip › figure 4/4D/4D STZ+AAV-blank.tif]

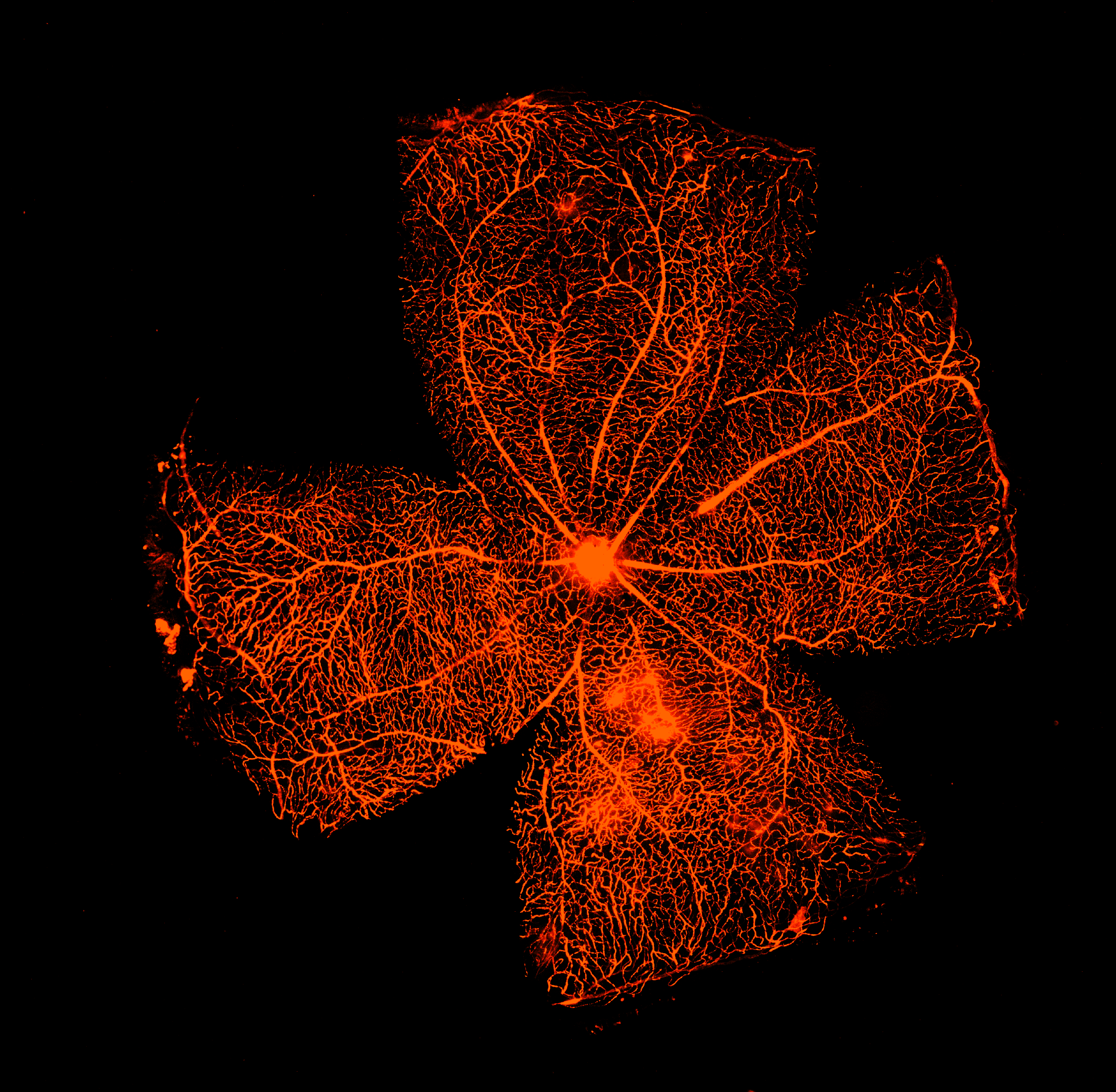

Supplement: Supplementary file 5 — Source Data Fig. 4 [file 44321_2024_25_MOESM5_ESM.zip › figure 4/4D/4D STZ+AAV-Fto.tif]

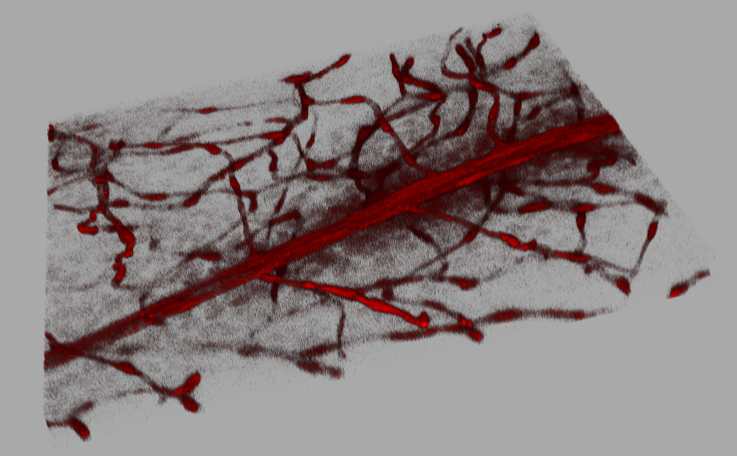

Supplement: Supplementary file 5 — Source Data Fig. 4 [file 44321_2024_25_MOESM5_ESM.zip › figure 4/4E/4E Ctrl 3D.tif]

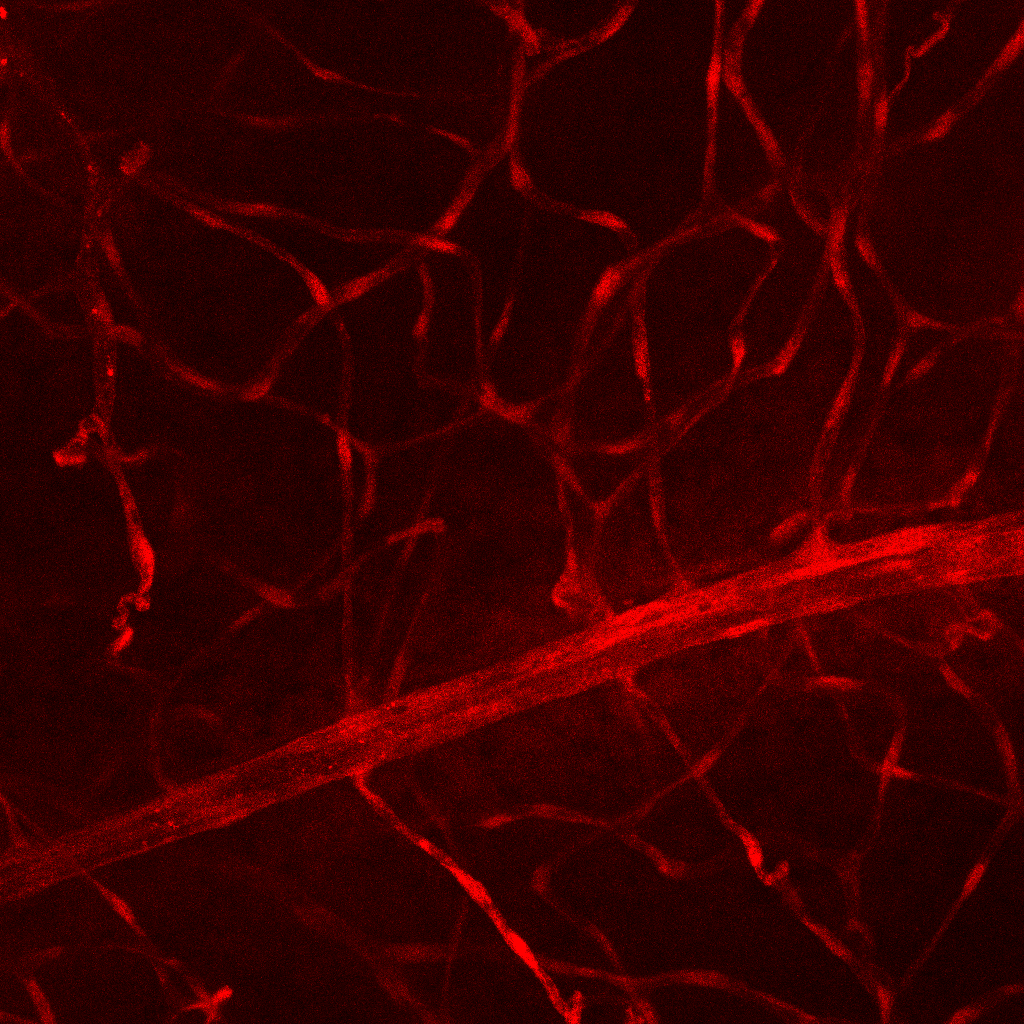

Supplement: Supplementary file 5 — Source Data Fig. 4 [file 44321_2024_25_MOESM5_ESM.zip › figure 4/4E/4E Ctrl lower line.tif]

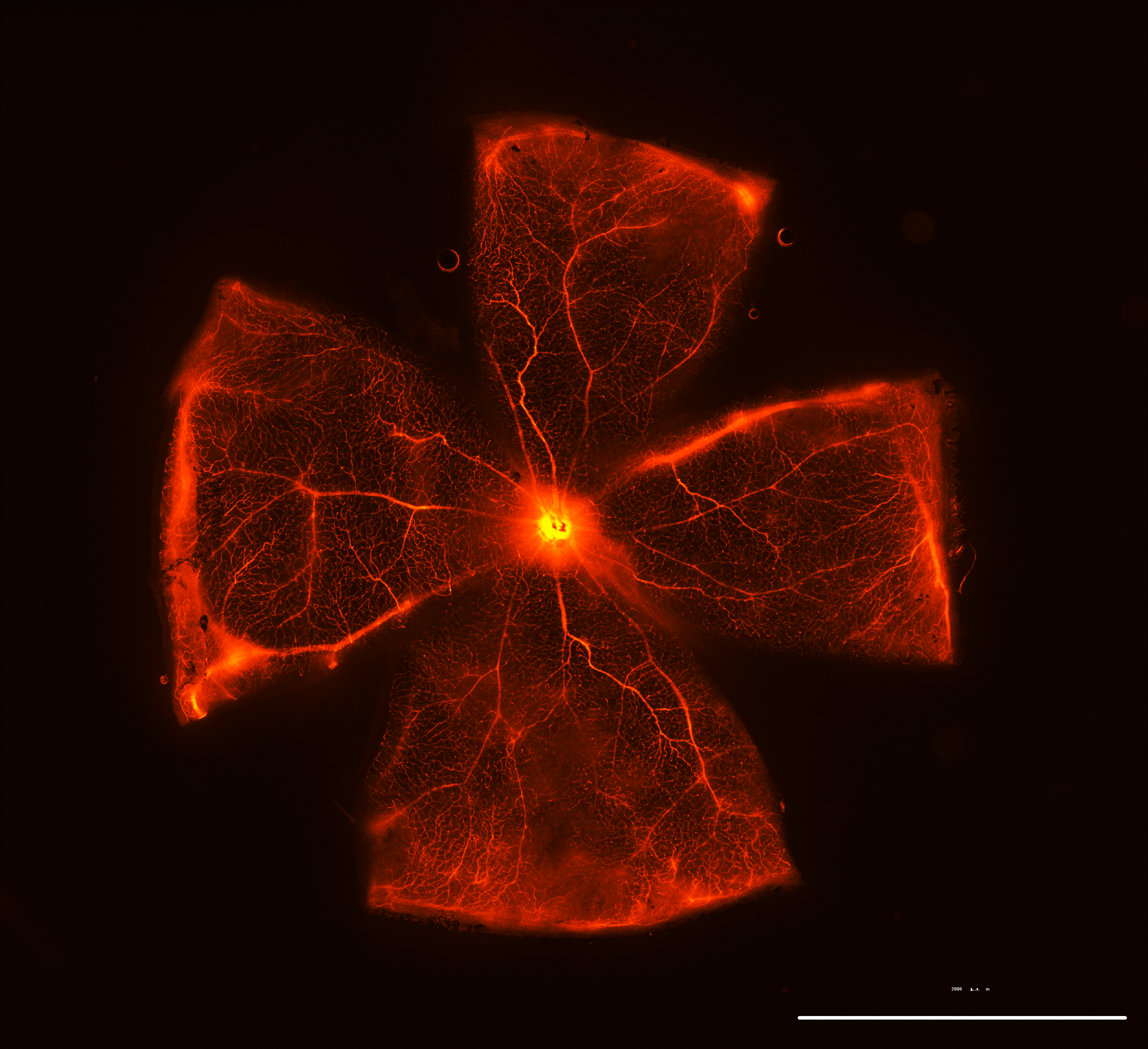

Supplement: Supplementary file 5 — Source Data Fig. 4 [file 44321_2024_25_MOESM5_ESM.zip › figure 4/4E/4E Ctrl upper line.tif]

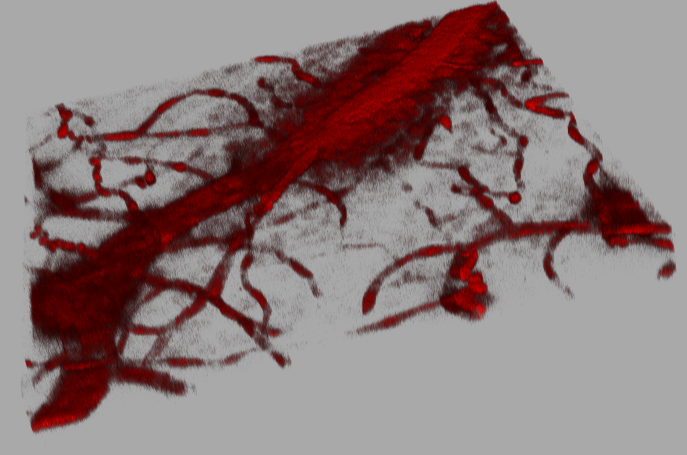

Supplement: Supplementary file 5 — Source Data Fig. 4 [file 44321_2024_25_MOESM5_ESM.zip › figure 4/4E/4E STZ 3D.tif]

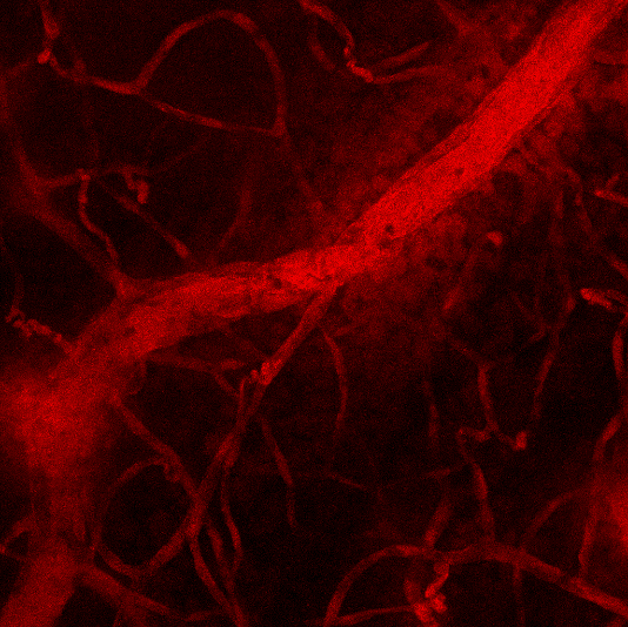

Supplement: Supplementary file 5 — Source Data Fig. 4 [file 44321_2024_25_MOESM5_ESM.zip › figure 4/4E/4E STZ lower line.tif]

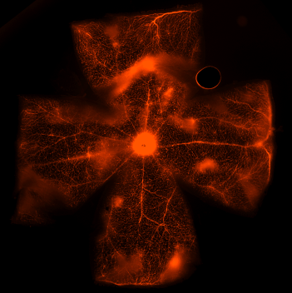

Supplement: Supplementary file 5 — Source Data Fig. 4 [file 44321_2024_25_MOESM5_ESM.zip › figure 4/4E/4E STZ upper line.tif]

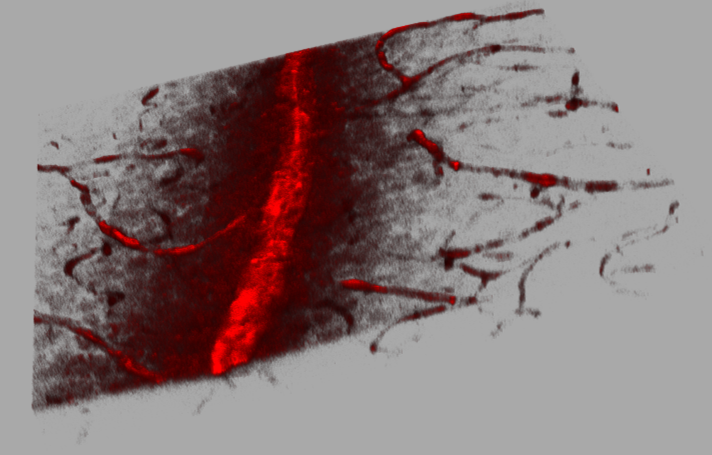

Supplement: Supplementary file 5 — Source Data Fig. 4 [file 44321_2024_25_MOESM5_ESM.zip › figure 4/4E/4E STZ+AAV-blank 3D.tif]

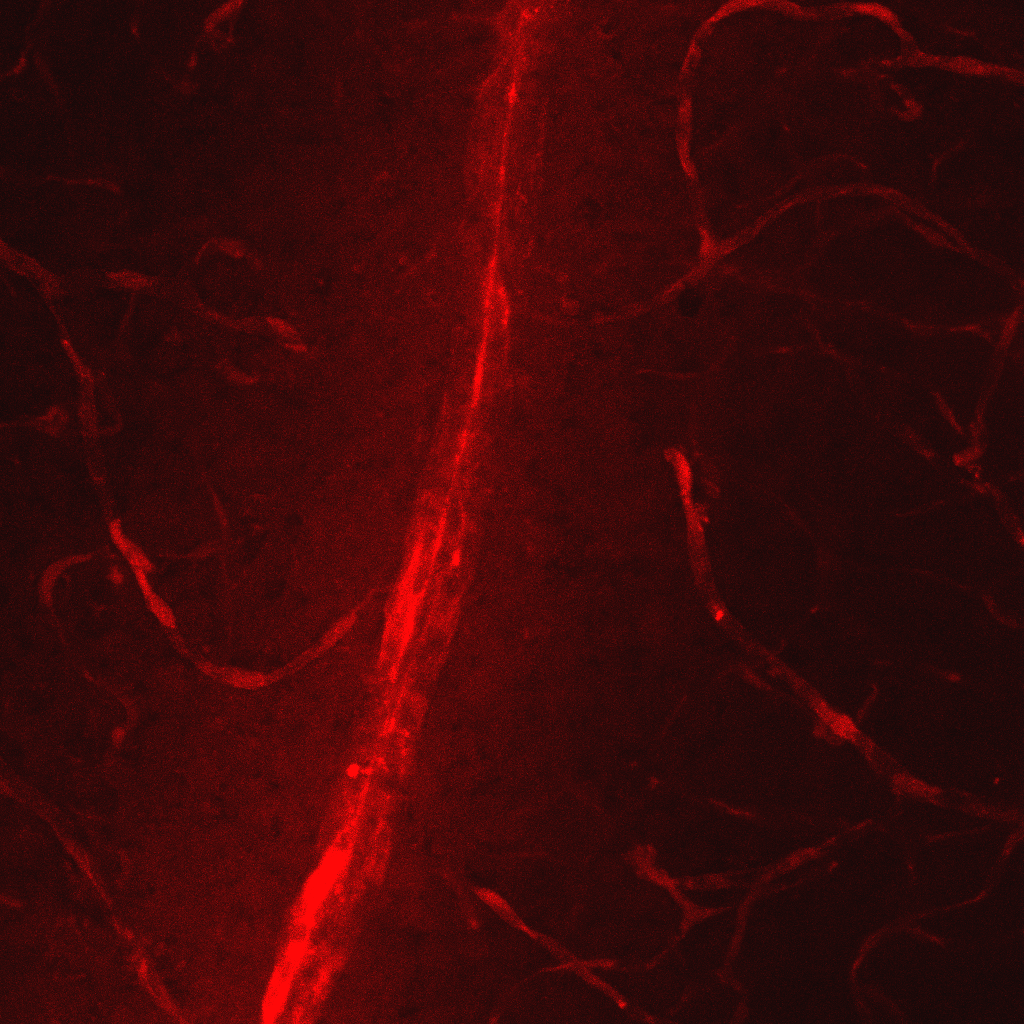

Supplement: Supplementary file 5 — Source Data Fig. 4 [file 44321_2024_25_MOESM5_ESM.zip › figure 4/4E/4E STZ+AAV-blank lower line.tif]

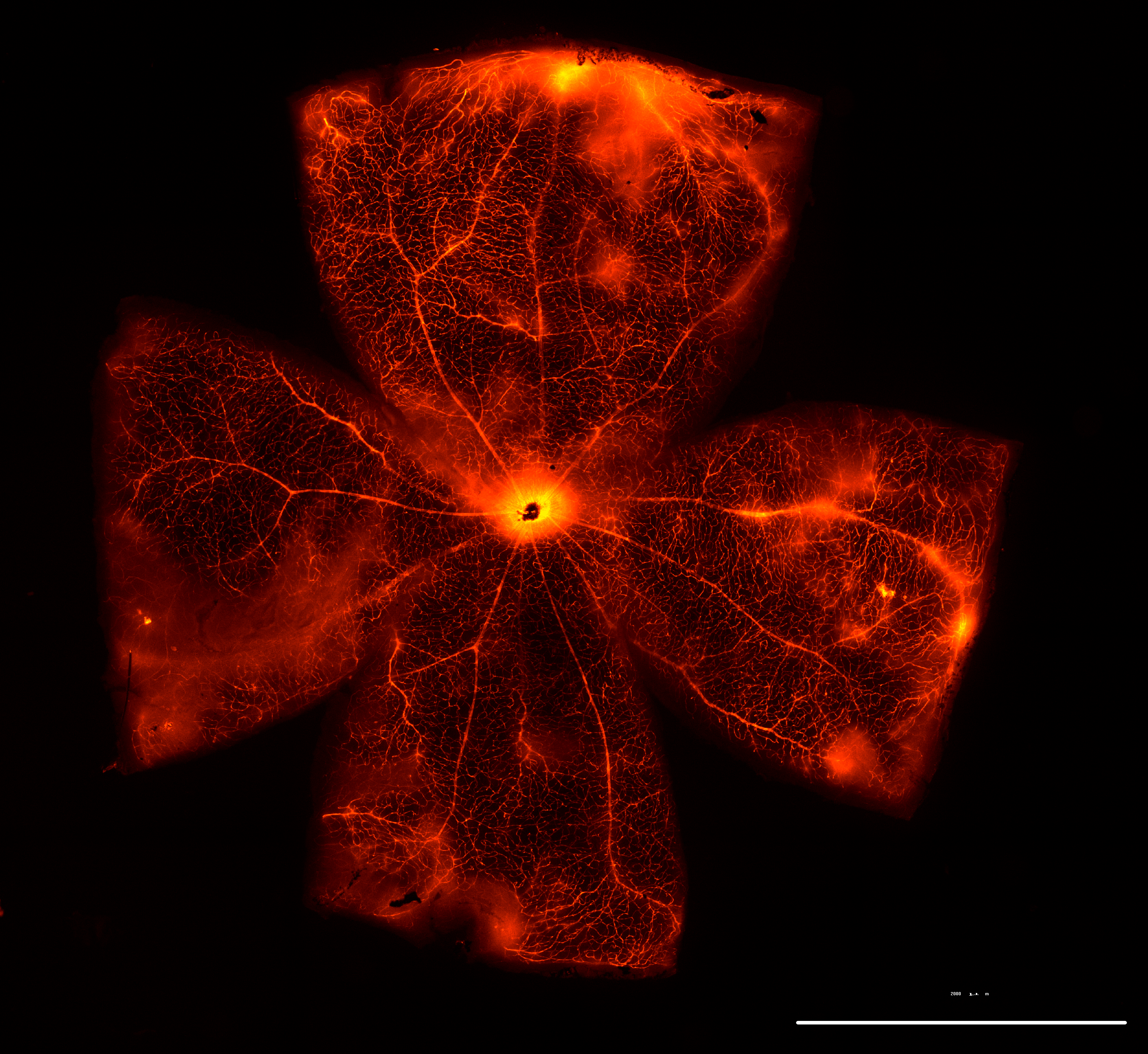

Supplement: Supplementary file 5 — Source Data Fig. 4 [file 44321_2024_25_MOESM5_ESM.zip › figure 4/4E/4E STZ+AAV-blank upper line.tif]

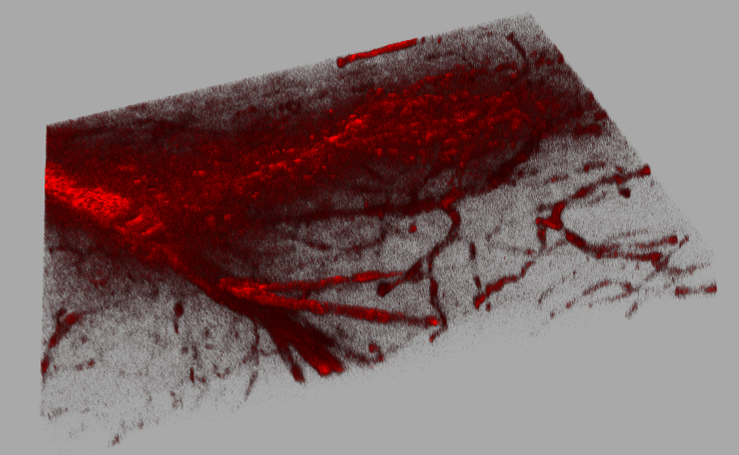

Supplement: Supplementary file 5 — Source Data Fig. 4 [file 44321_2024_25_MOESM5_ESM.zip › figure 4/4E/4E STZ+AAV-Fto 3D.tif]

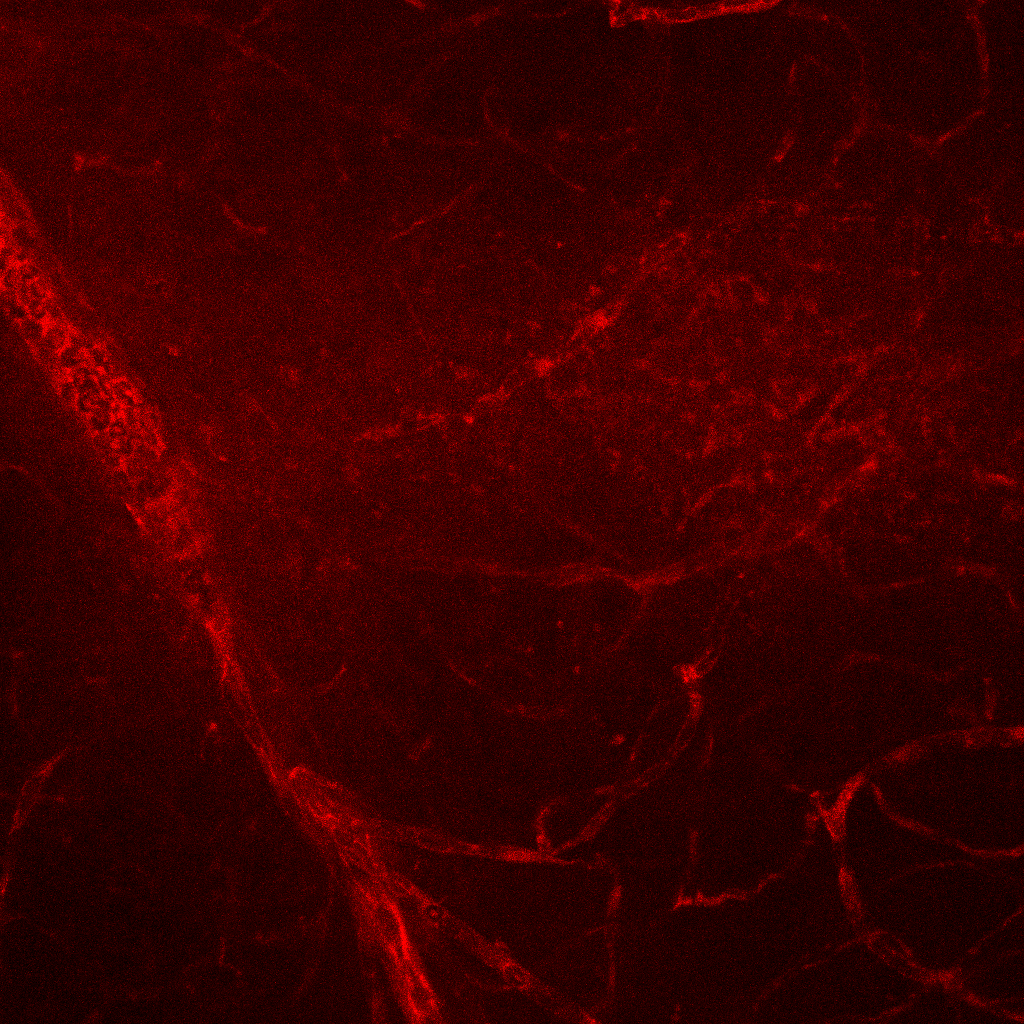

Supplement: Supplementary file 5 — Source Data Fig. 4 [file 44321_2024_25_MOESM5_ESM.zip › figure 4/4E/4E STZ+AAV-Fto lower line.tif]

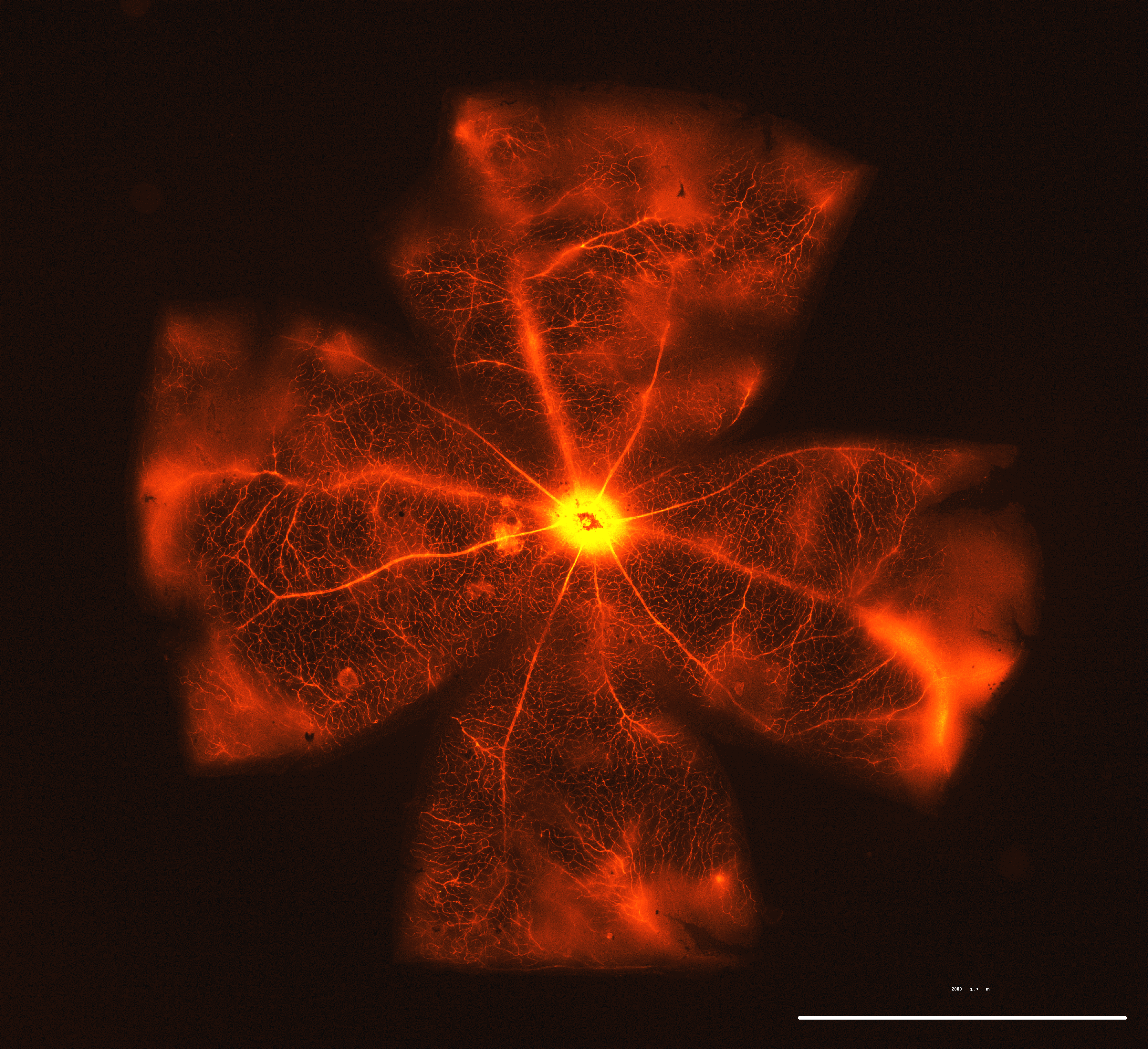

Supplement: Supplementary file 5 — Source Data Fig. 4 [file 44321_2024_25_MOESM5_ESM.zip › figure 4/4E/4E STZ+AAV-Fto upper line.tif]

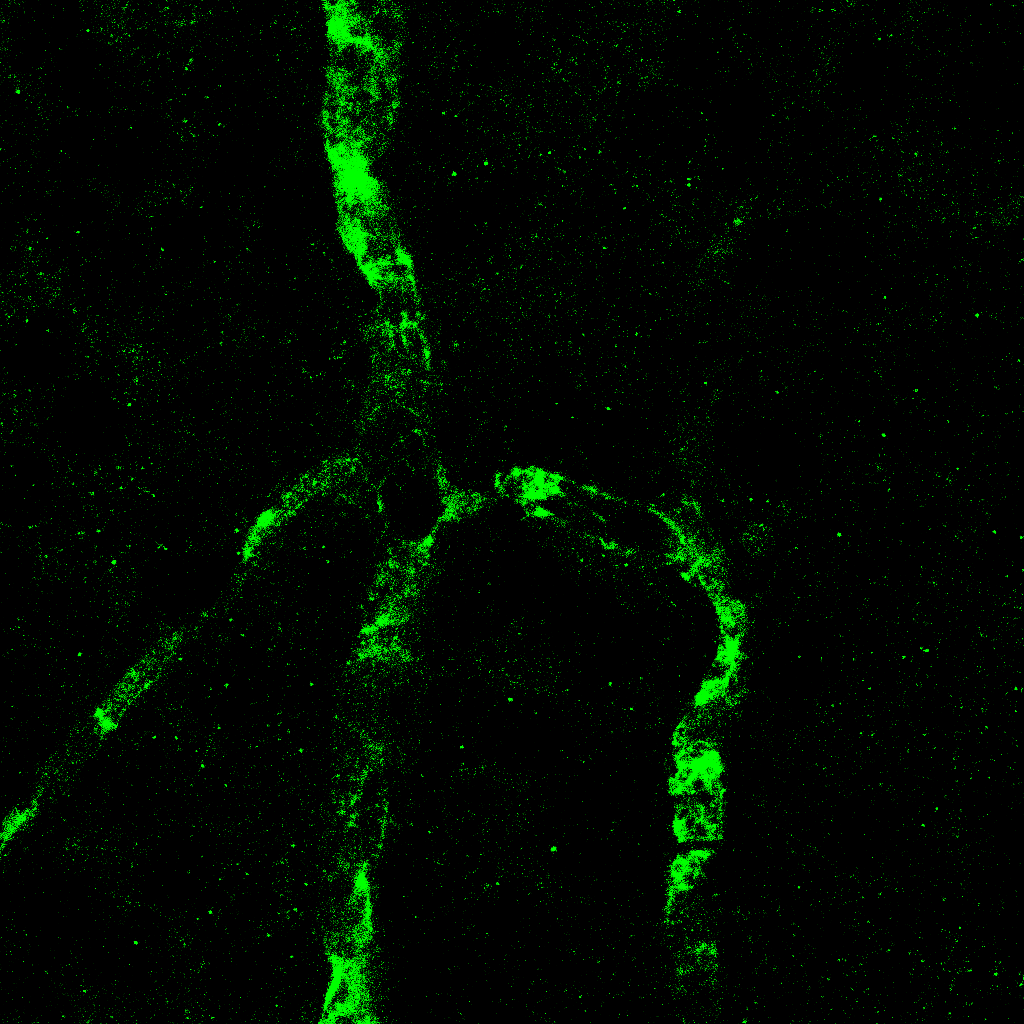

Supplement: Supplementary file 5 — Source Data Fig. 4 [file 44321_2024_25_MOESM5_ESM.zip › figure 4/4F/4F Ctrl VE cadherin lower.tif]

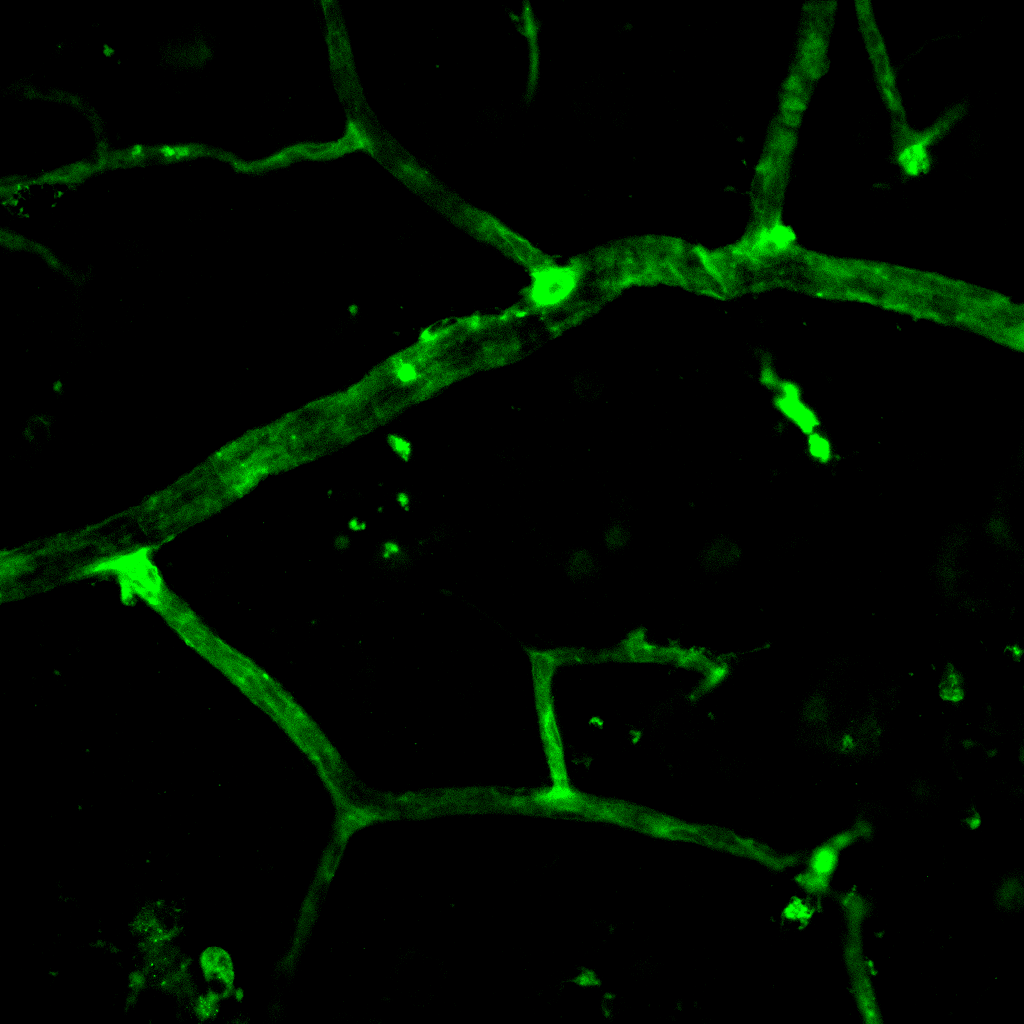

Supplement: Supplementary file 5 — Source Data Fig. 4 [file 44321_2024_25_MOESM5_ESM.zip › figure 4/4F/4F Ctrl VE cadherin upper.tif]

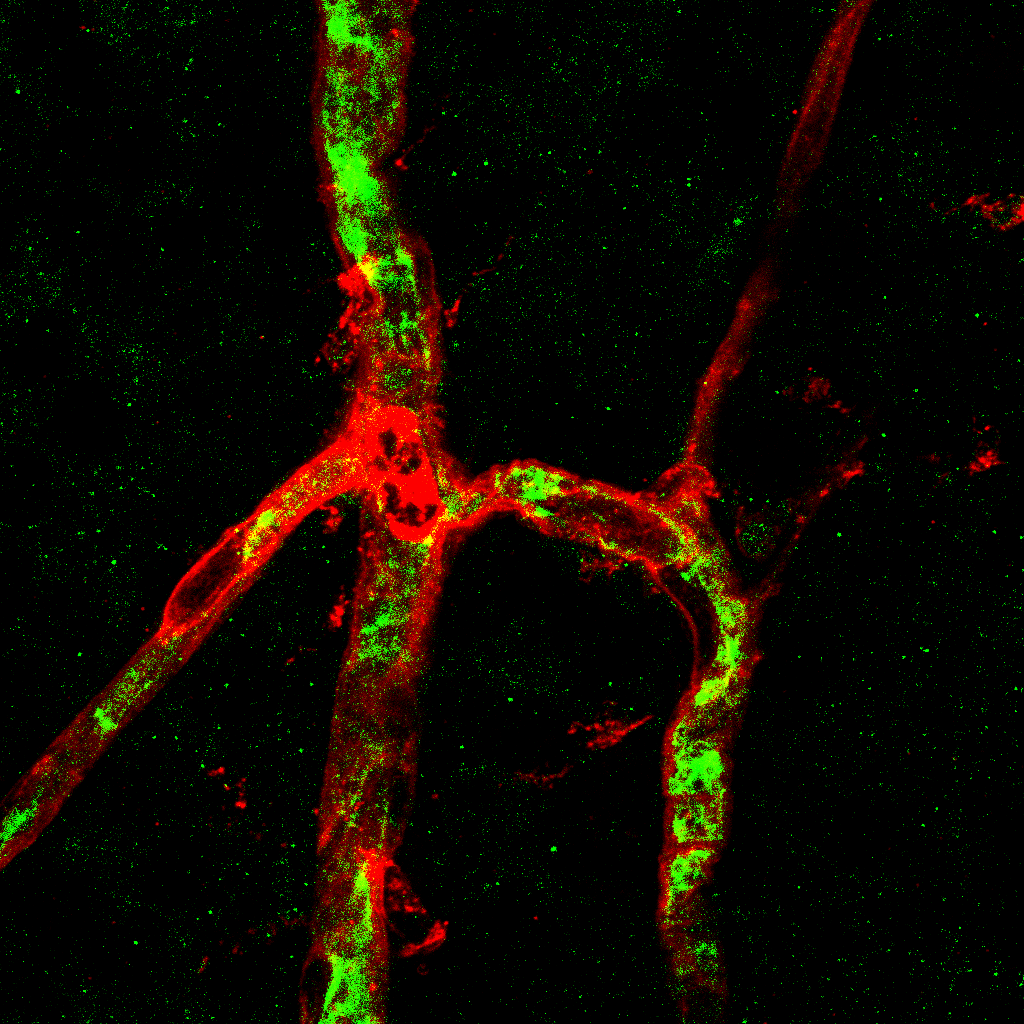

Supplement: Supplementary file 5 — Source Data Fig. 4 [file 44321_2024_25_MOESM5_ESM.zip › figure 4/4F/4F Ctrl VE cadherin+IB4 lower.tif]

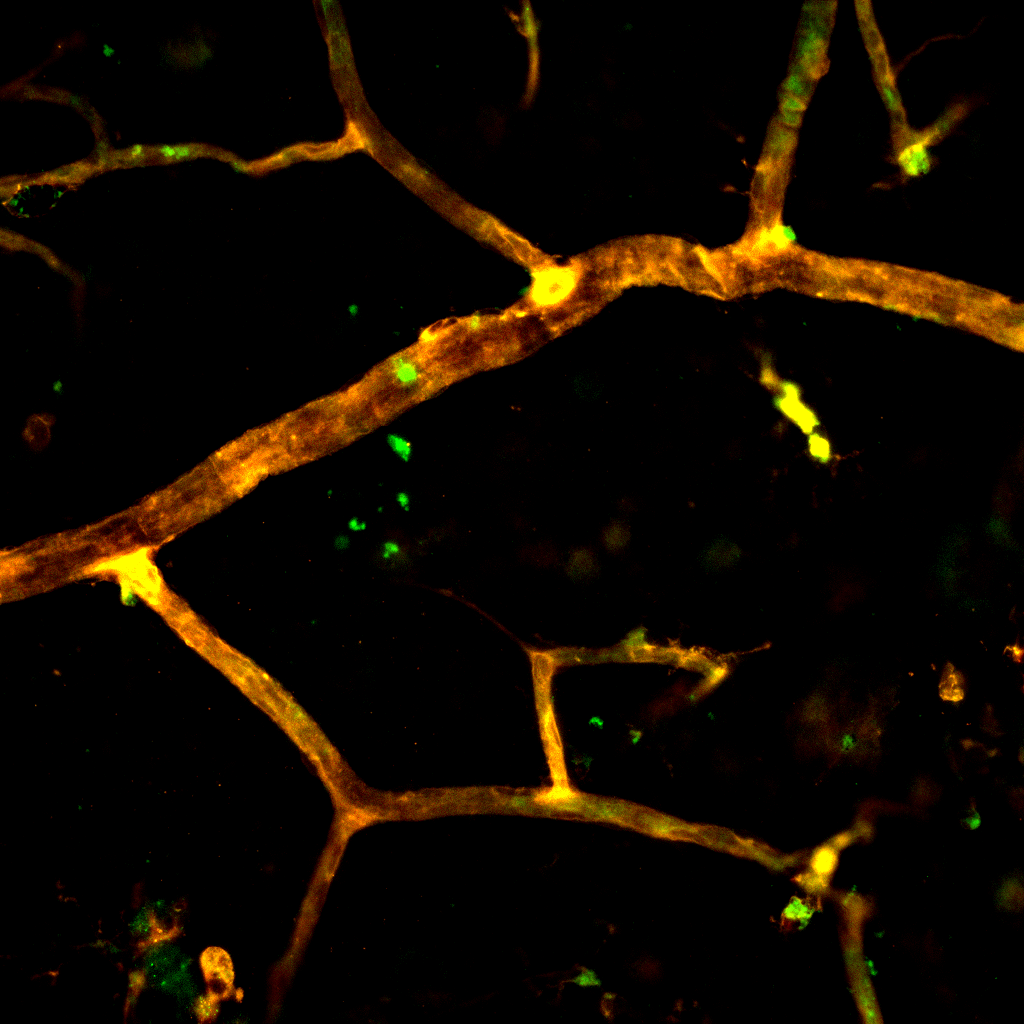

Supplement: Supplementary file 5 — Source Data Fig. 4 [file 44321_2024_25_MOESM5_ESM.zip › figure 4/4F/4F Ctrl VE cadherin+IB4 upper.tif]

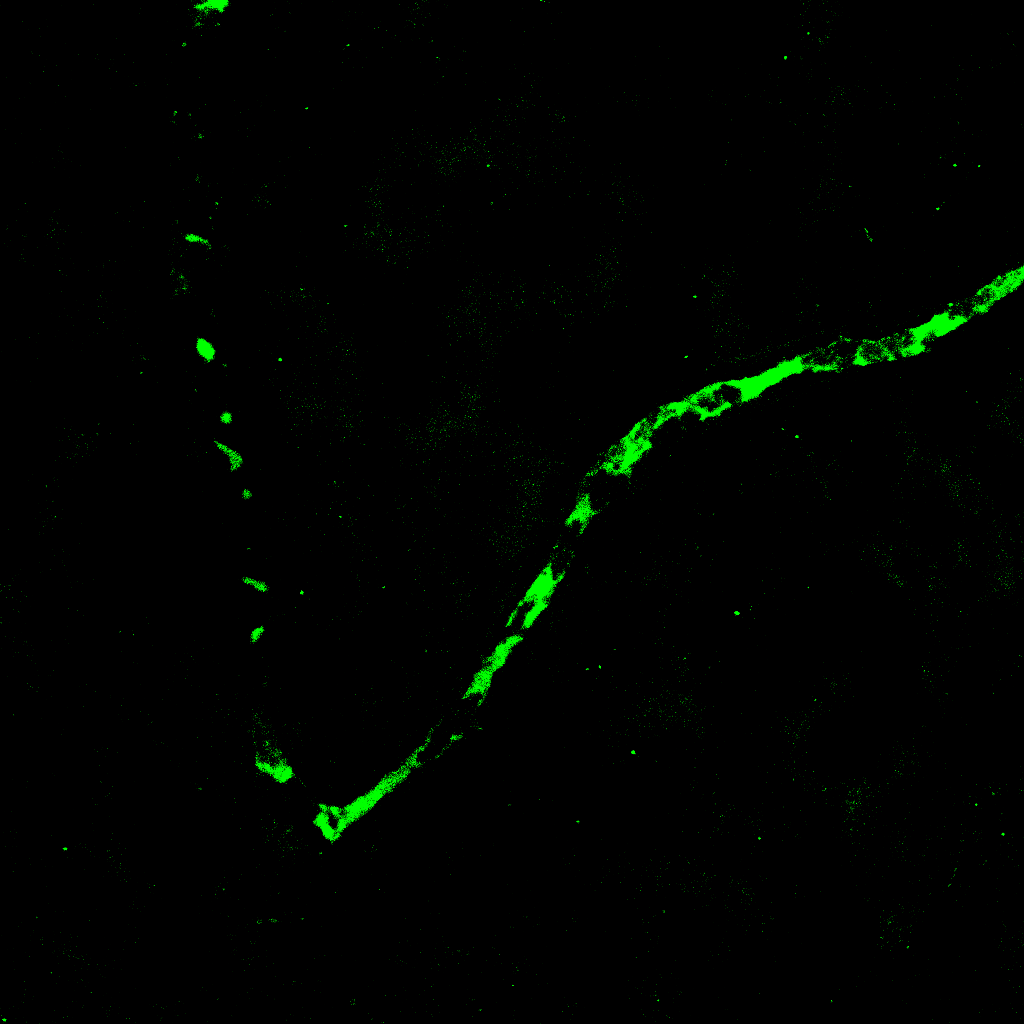

Supplement: Supplementary file 5 — Source Data Fig. 4 [file 44321_2024_25_MOESM5_ESM.zip › figure 4/4F/4F STZ VE cadherin lower.tif]

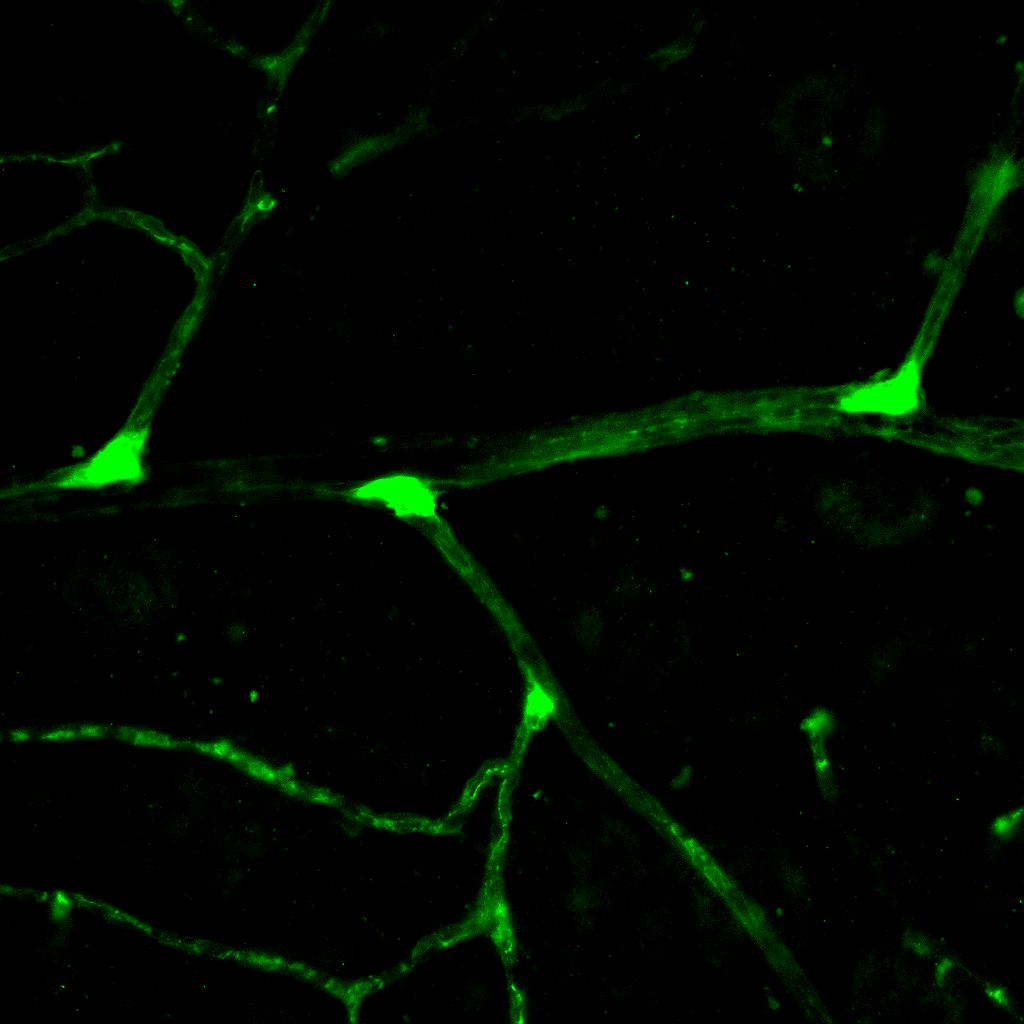

Supplement: Supplementary file 5 — Source Data Fig. 4 [file 44321_2024_25_MOESM5_ESM.zip › figure 4/4F/4F STZ VE cadherin upper.tif]

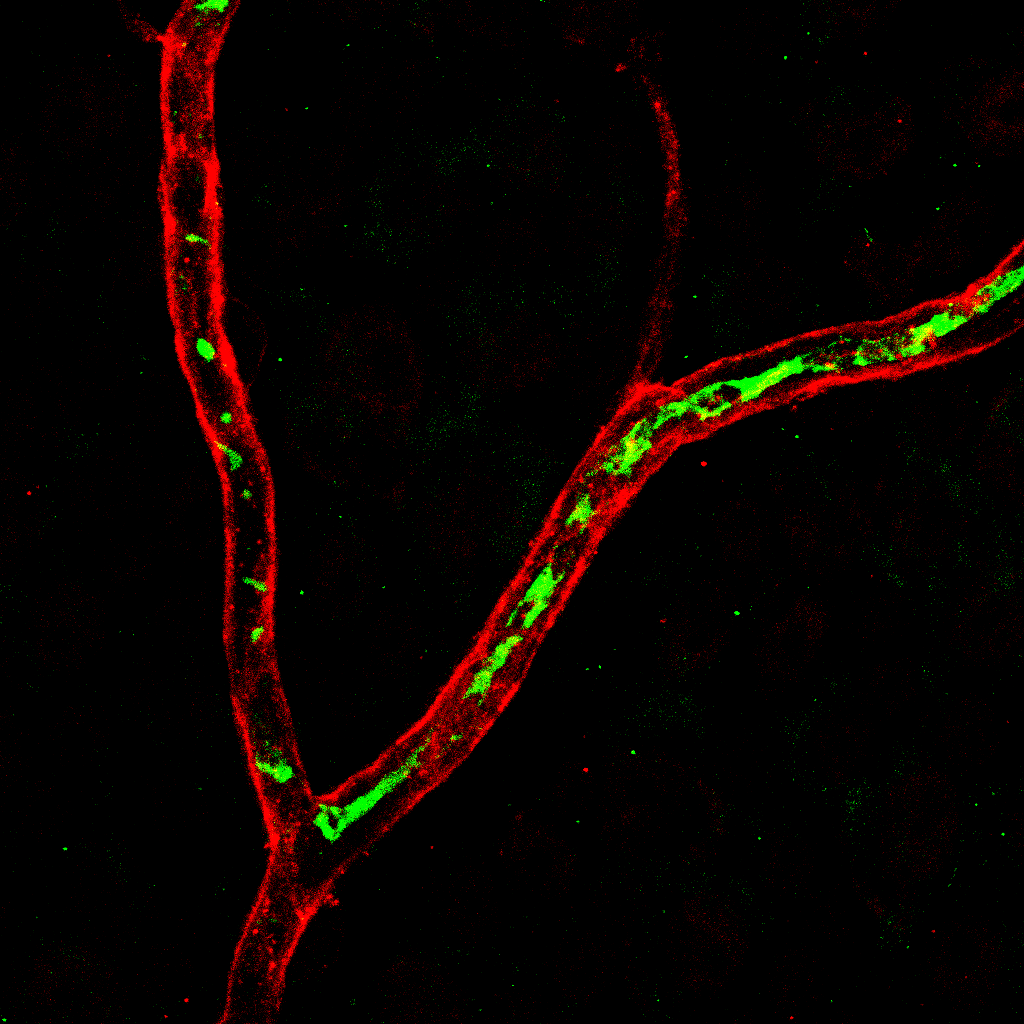

Supplement: Supplementary file 5 — Source Data Fig. 4 [file 44321_2024_25_MOESM5_ESM.zip › figure 4/4F/4F STZ VE cadherin+IB4 lower.tif]

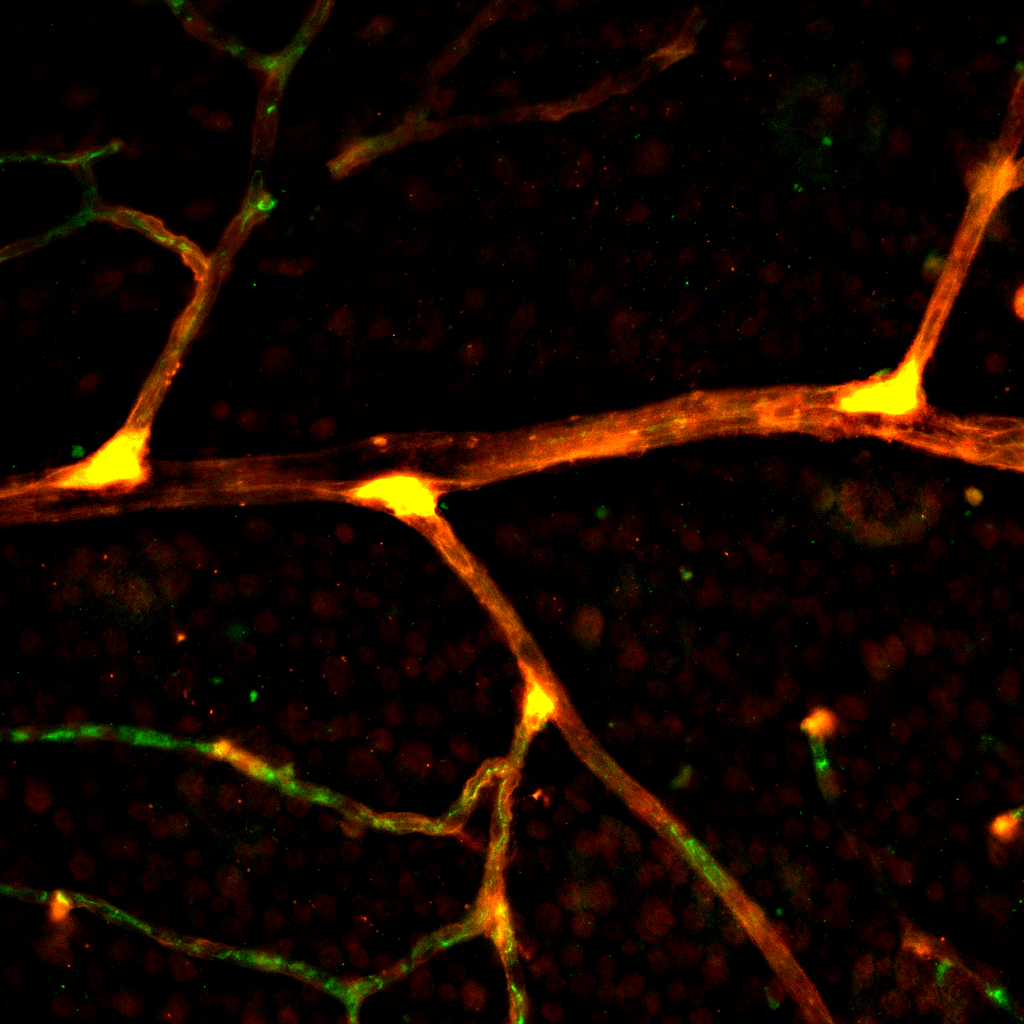

Supplement: Supplementary file 5 — Source Data Fig. 4 [file 44321_2024_25_MOESM5_ESM.zip › figure 4/4F/4F STZ VE cadherin+IB4 upper.tif]

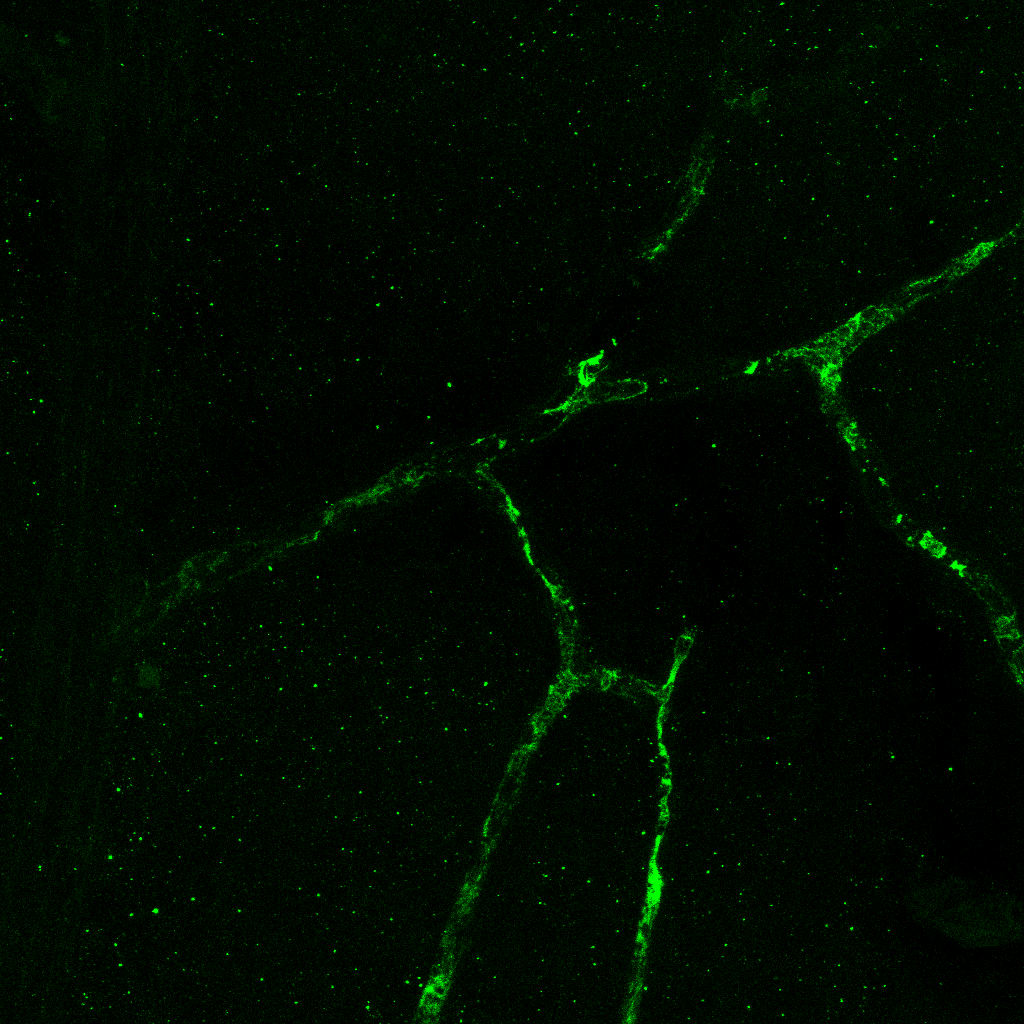

Supplement: Supplementary file 5 — Source Data Fig. 4 [file 44321_2024_25_MOESM5_ESM.zip › figure 4/4F/4F STZ+AAV-blank VE cadherin lower.tif]

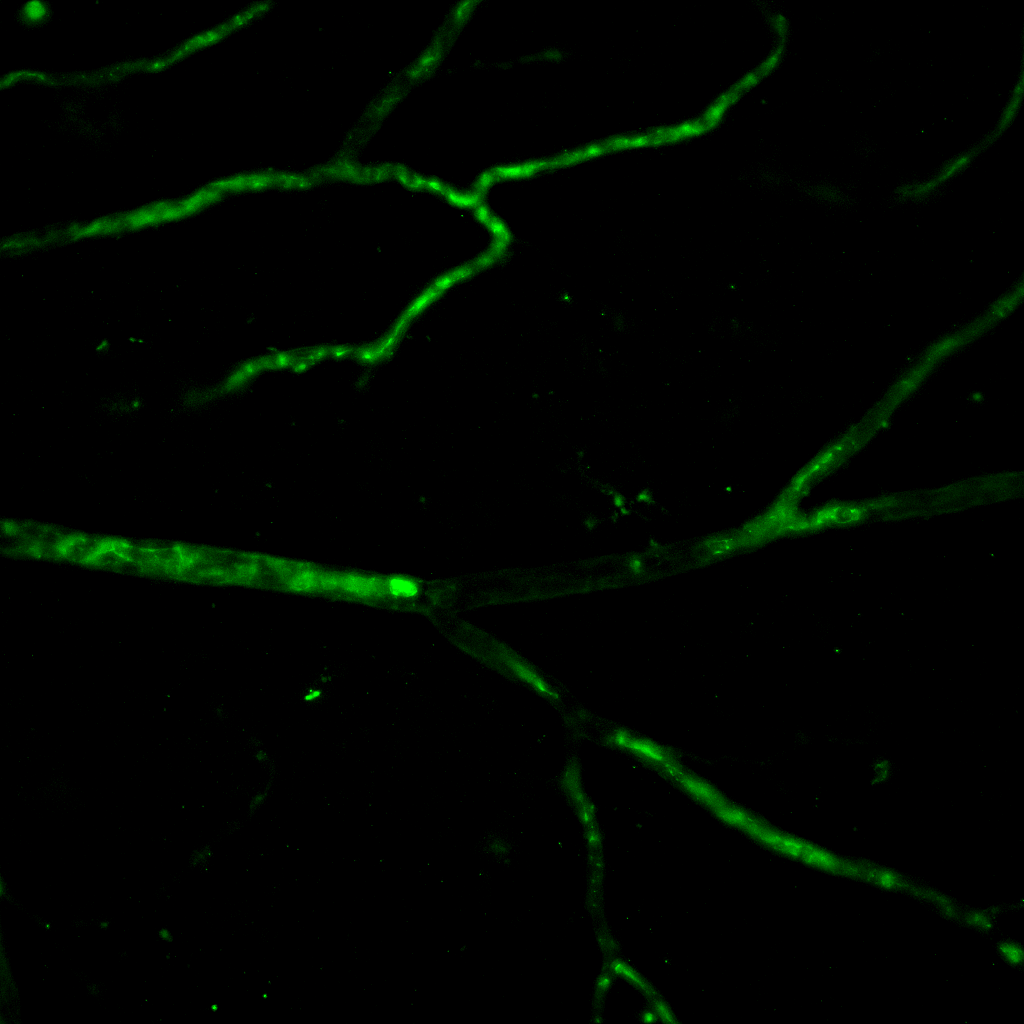

Supplement: Supplementary file 5 — Source Data Fig. 4 [file 44321_2024_25_MOESM5_ESM.zip › figure 4/4F/4F STZ+AAV-blank VE cadherin upper.tif]

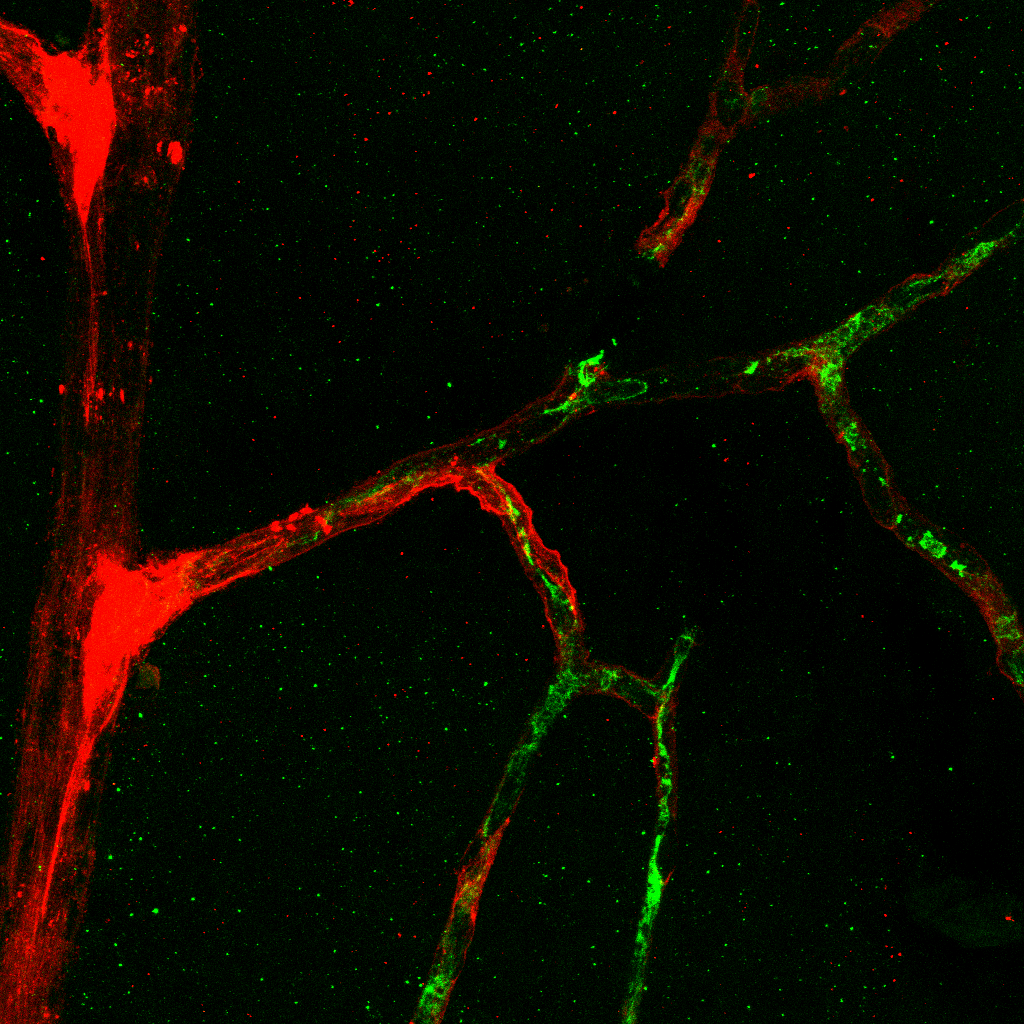

Supplement: Supplementary file 5 — Source Data Fig. 4 [file 44321_2024_25_MOESM5_ESM.zip › figure 4/4F/4F STZ+AAV-blank VE cadherin+IB4 lower.tif]

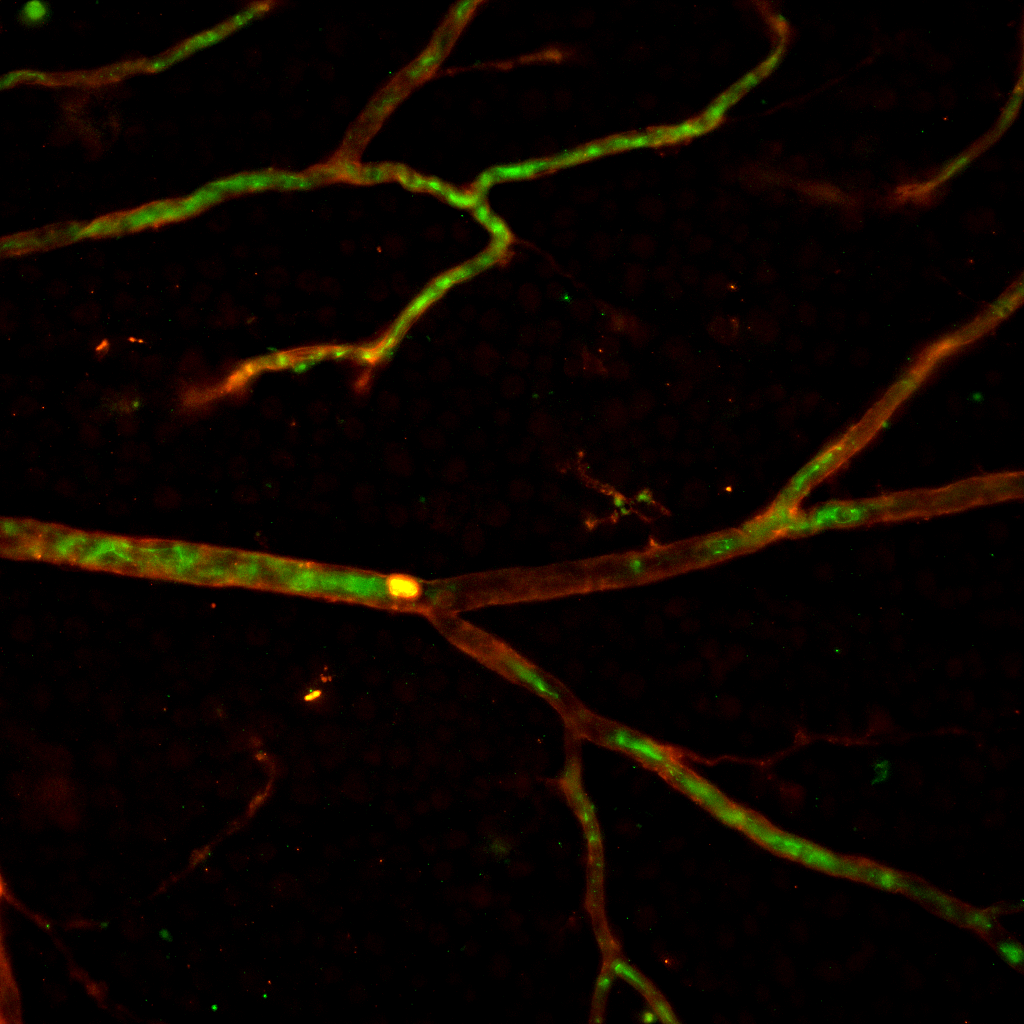

Supplement: Supplementary file 5 — Source Data Fig. 4 [file 44321_2024_25_MOESM5_ESM.zip › figure 4/4F/4F STZ+AAV-blank VE cadherin+IB4 upper.tif]

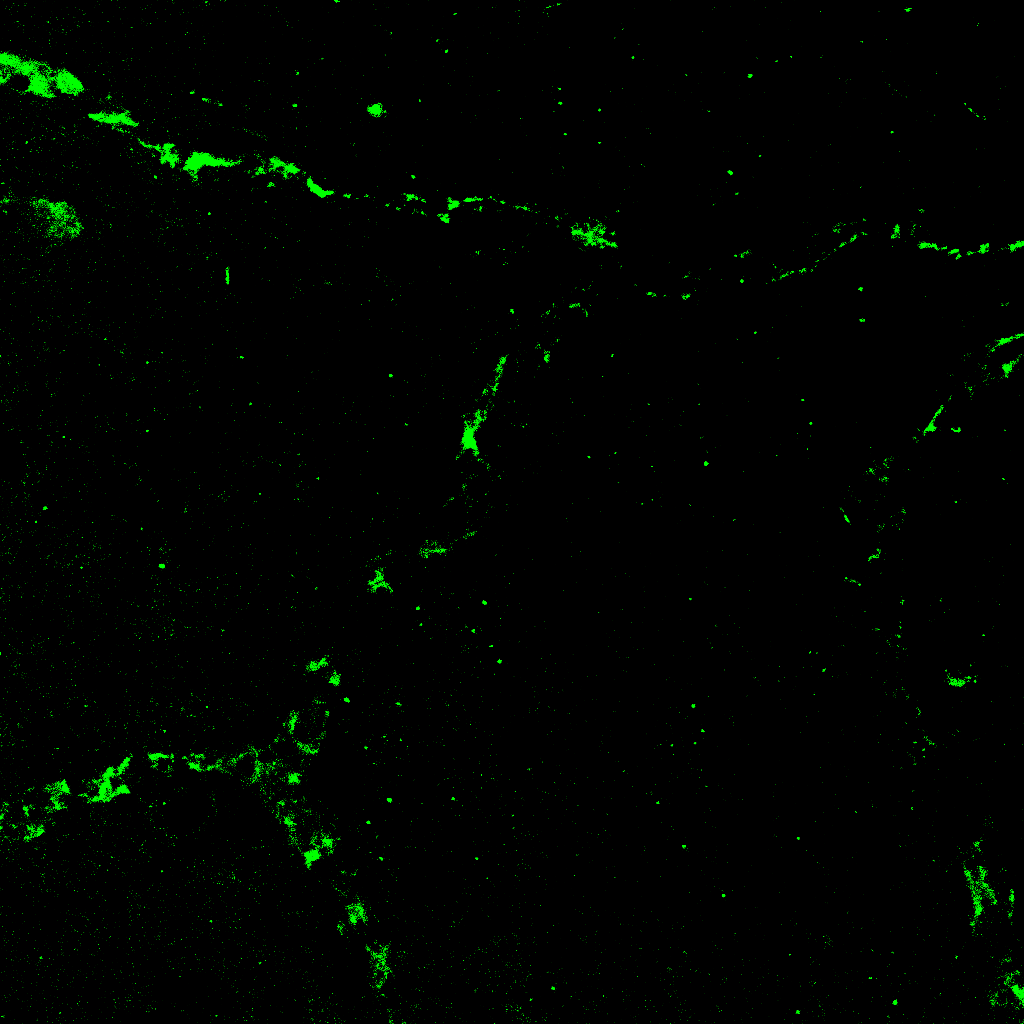

Supplement: Supplementary file 5 — Source Data Fig. 4 [file 44321_2024_25_MOESM5_ESM.zip › figure 4/4F/4F STZ+AAV-Fto VE cadherin lower.tif]

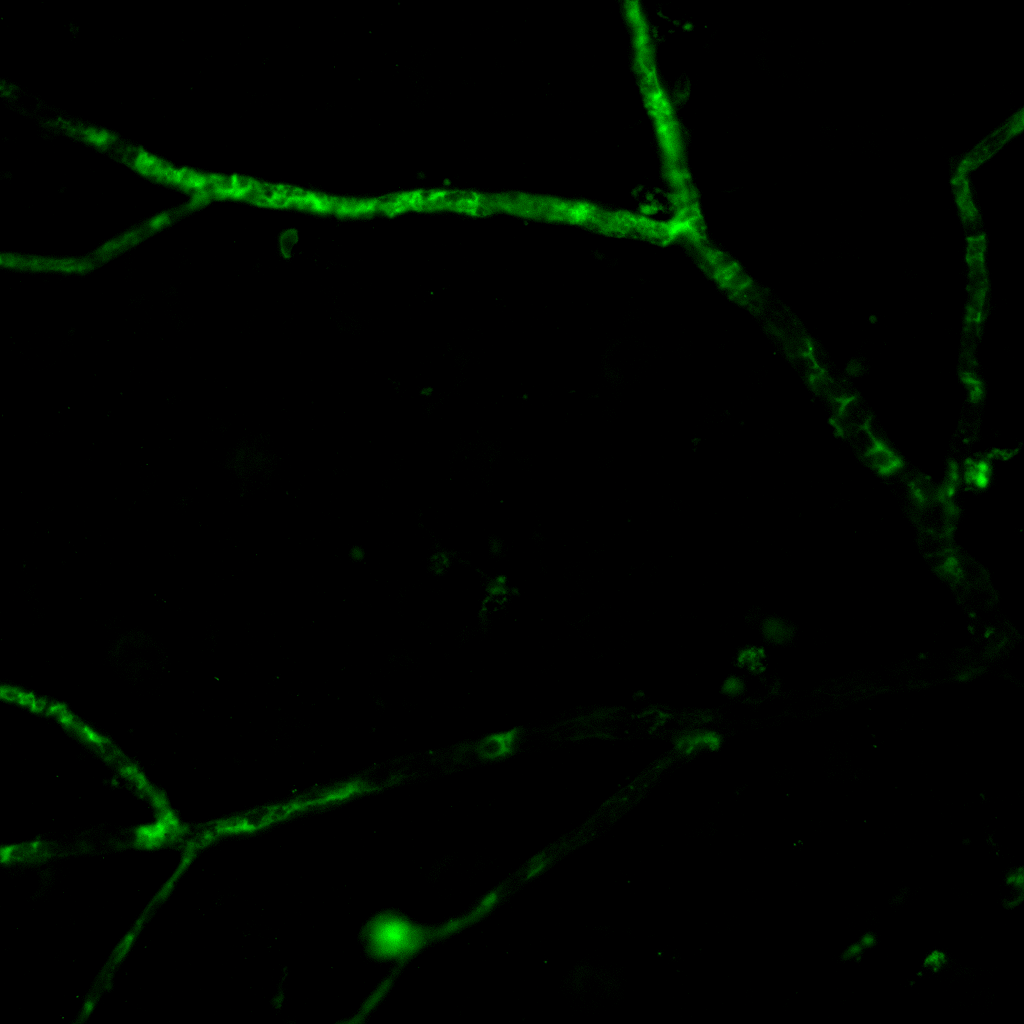

Supplement: Supplementary file 5 — Source Data Fig. 4 [file 44321_2024_25_MOESM5_ESM.zip › figure 4/4F/4F STZ+AAV-Fto VE cadherin upper.tif]

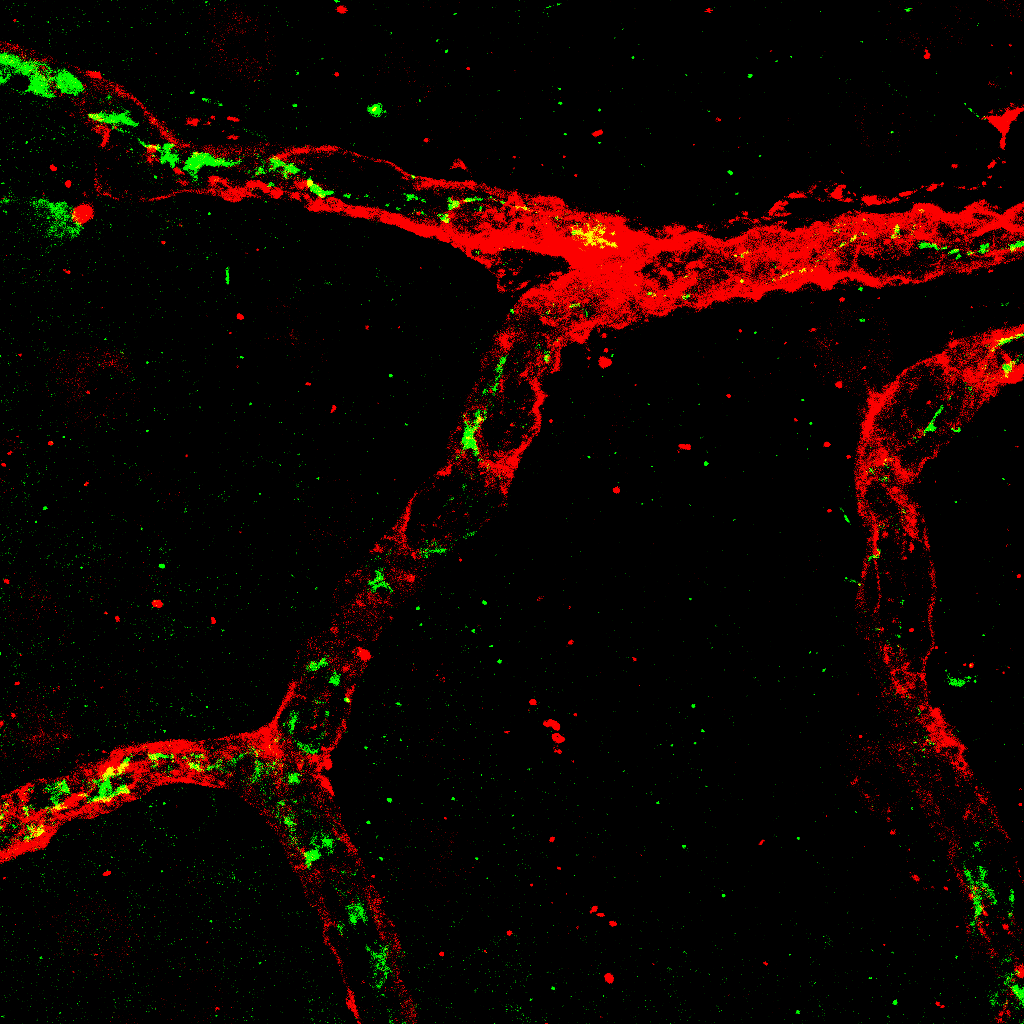

Supplement: Supplementary file 5 — Source Data Fig. 4 [file 44321_2024_25_MOESM5_ESM.zip › figure 4/4F/4F STZ+AAV-Fto VE cadherin+IB4 lower.tif]

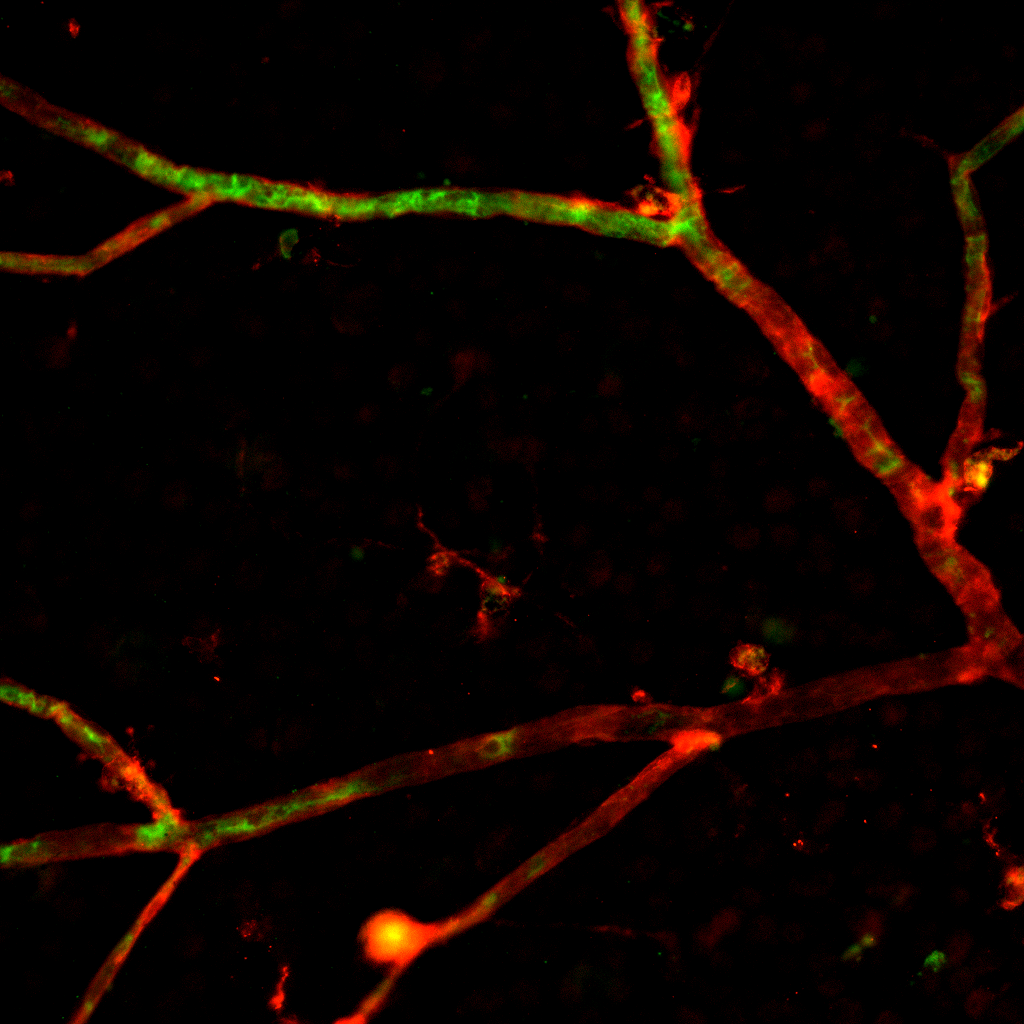

Supplement: Supplementary file 5 — Source Data Fig. 4 [file 44321_2024_25_MOESM5_ESM.zip › figure 4/4F/4F STZ+AAV-Fto VE cadherin+IB4 upper.tif]

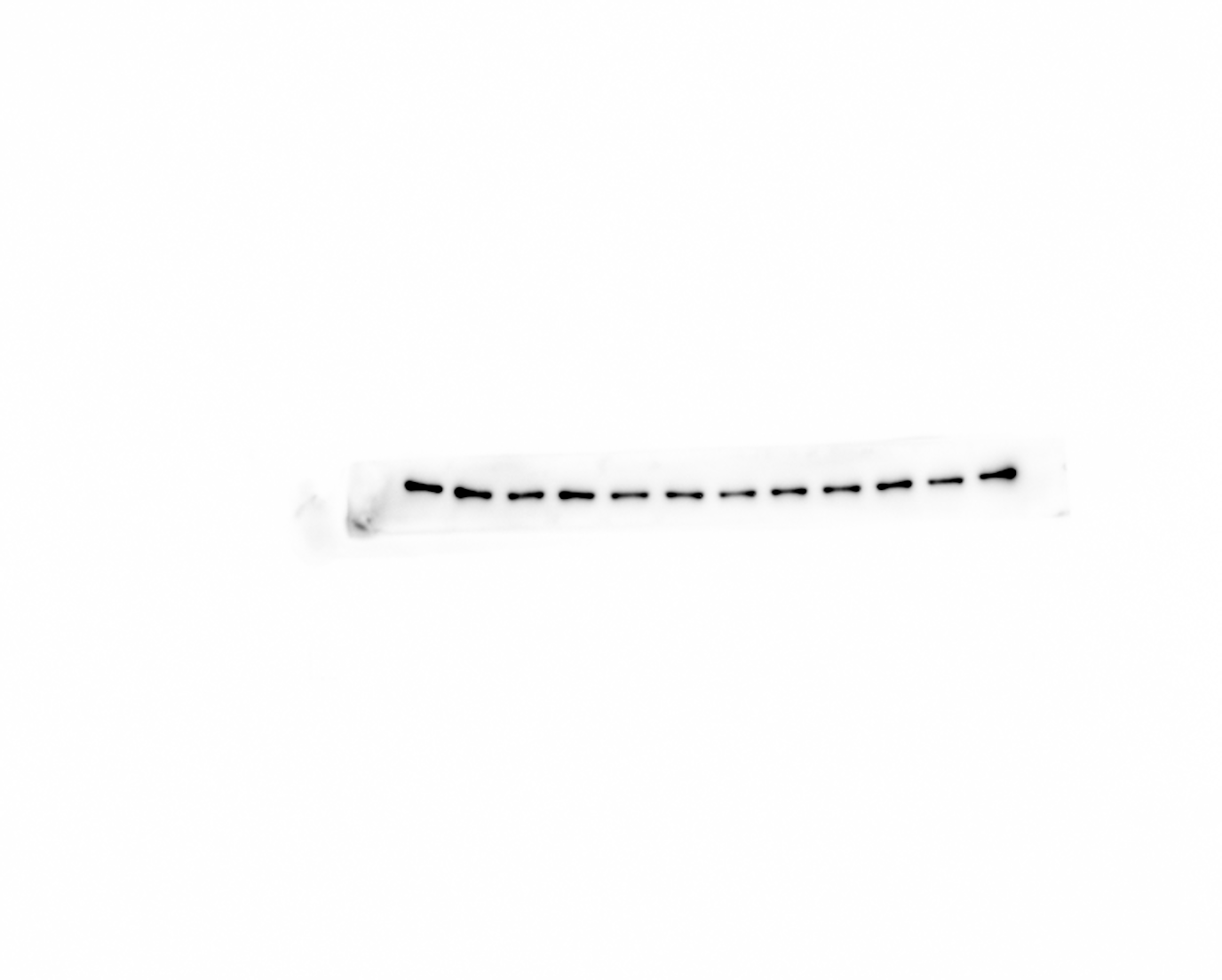

Supplement: Supplementary file 5 — Source Data Fig. 4 [file 44321_2024_25_MOESM5_ESM.zip › figure 4/4G/4G Gapdh for ZO-1 mark replicate.Tif]

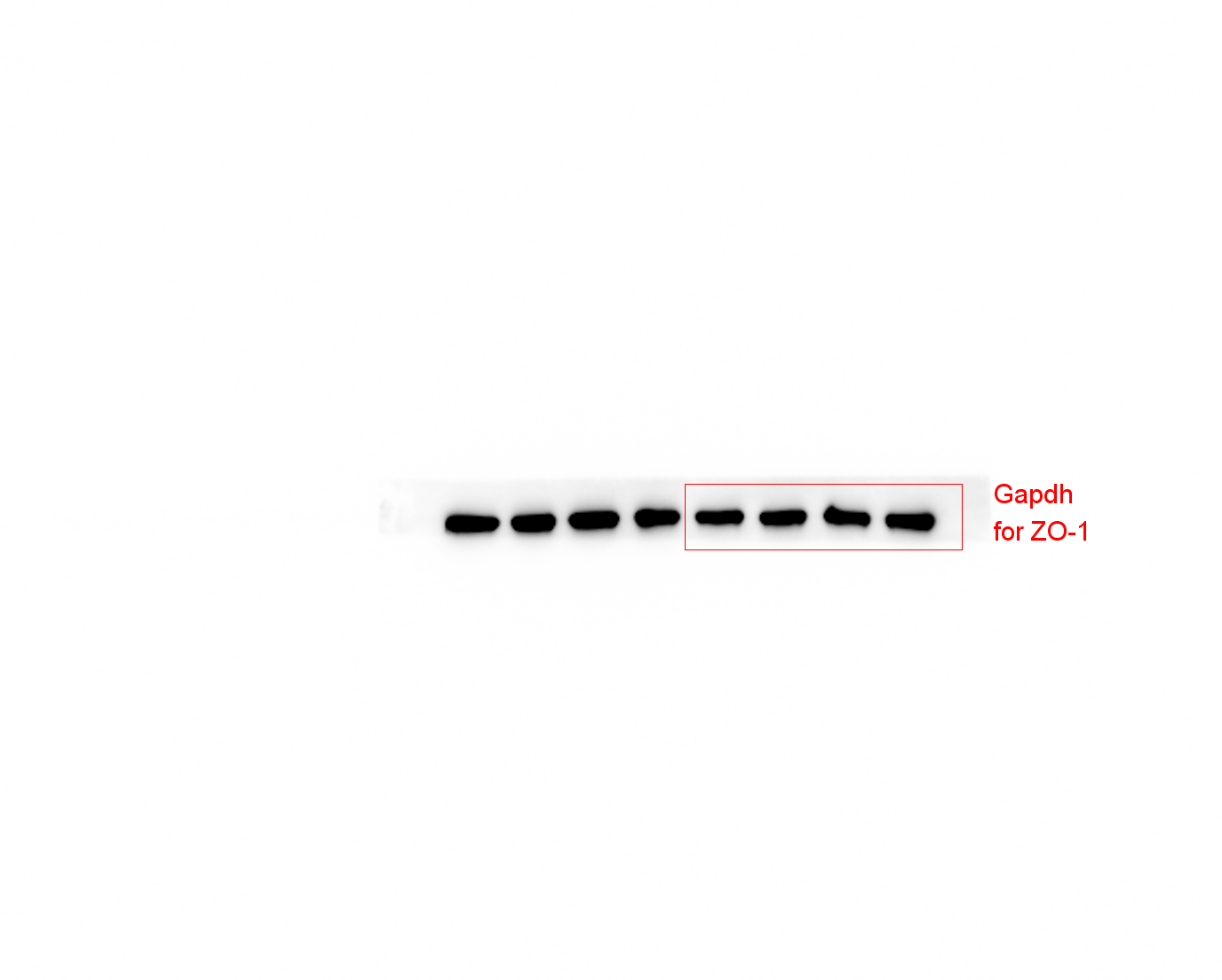

Supplement: Supplementary file 5 — Source Data Fig. 4 [file 44321_2024_25_MOESM5_ESM.zip › figure 4/4G/4G Gapdh for ZO-1 mark.Tif]

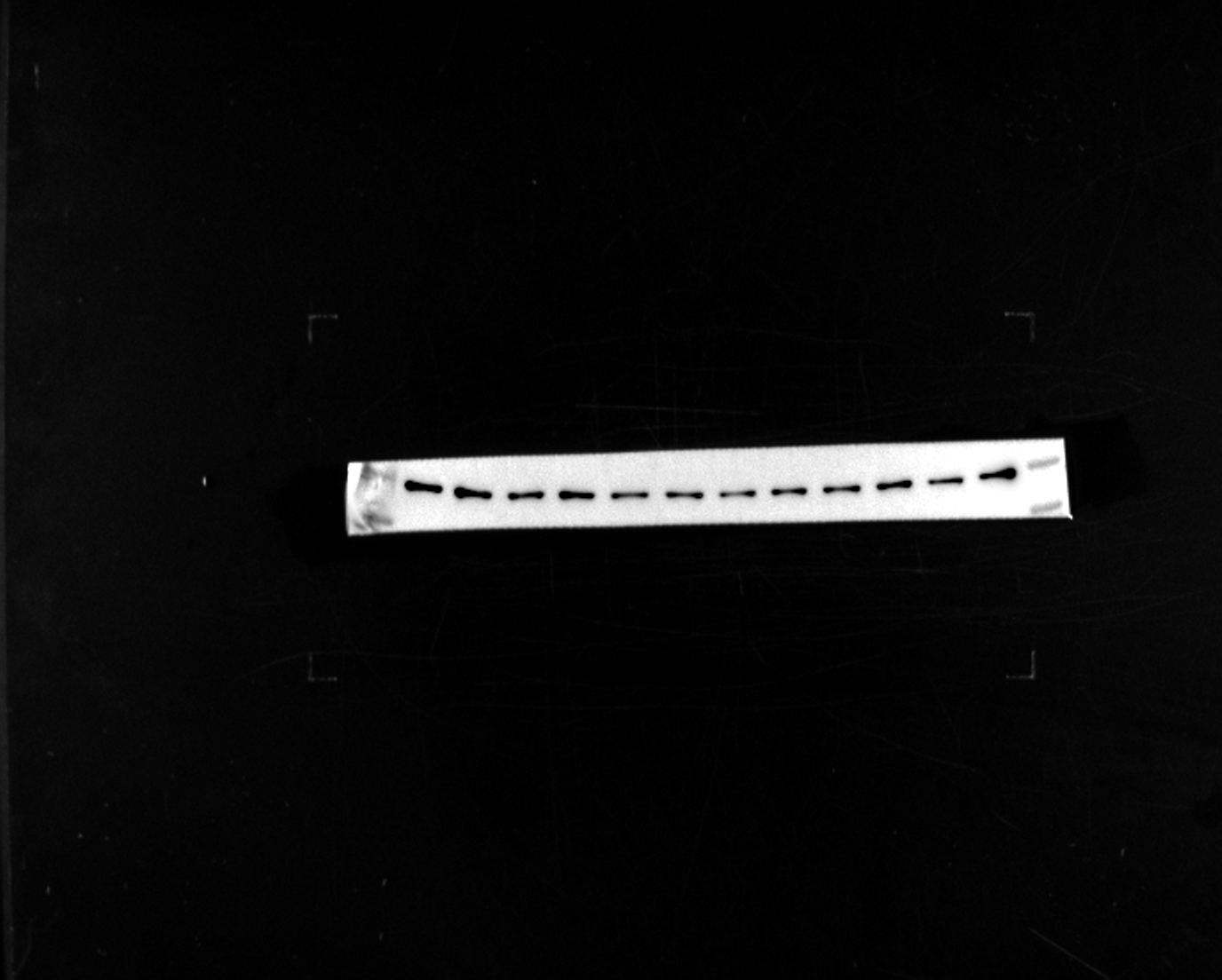

Supplement: Supplementary file 5 — Source Data Fig. 4 [file 44321_2024_25_MOESM5_ESM.zip › figure 4/4G/4G Gapdh for ZO-1 replicate.Tif]

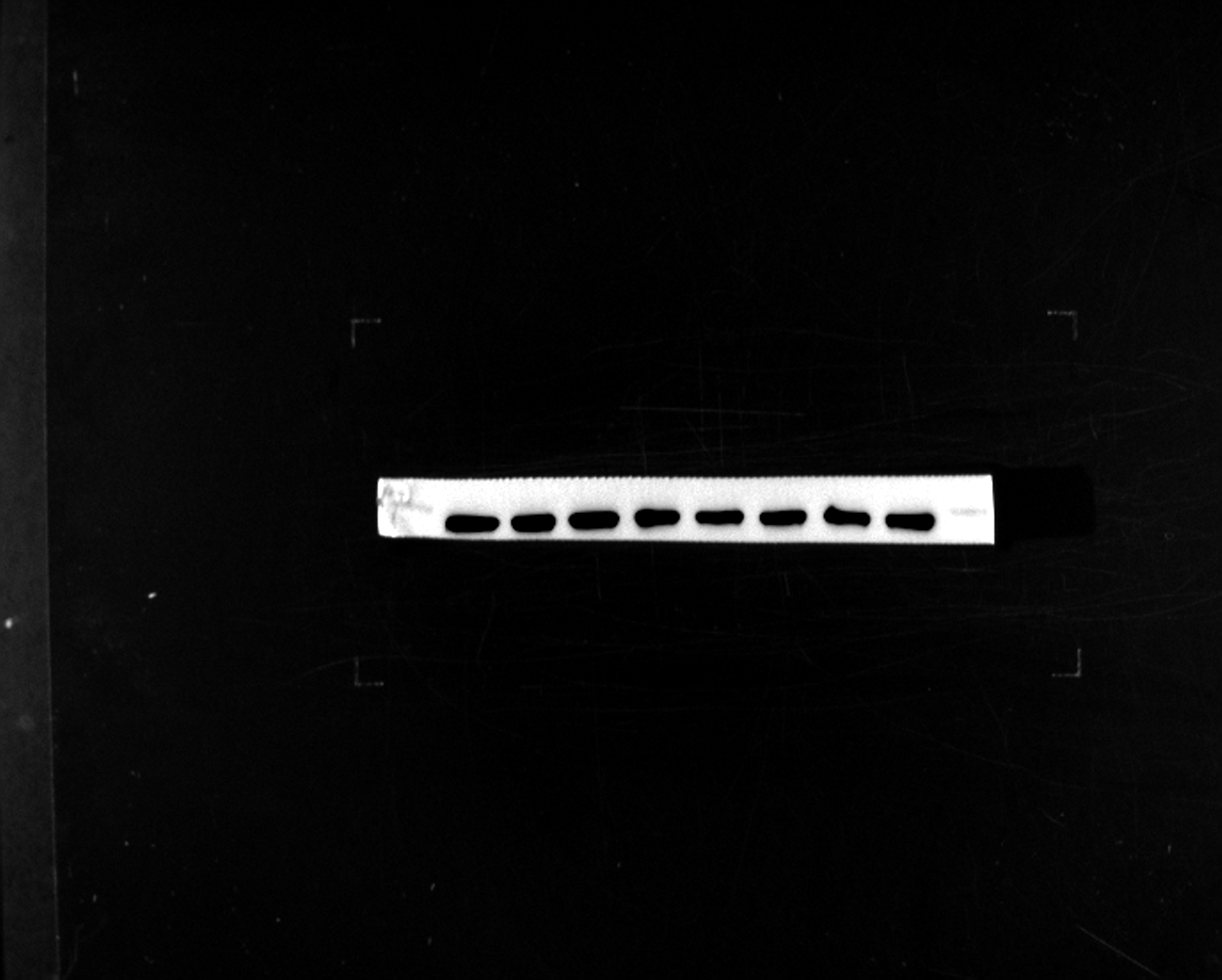

Supplement: Supplementary file 5 — Source Data Fig. 4 [file 44321_2024_25_MOESM5_ESM.zip › figure 4/4G/4G Gapdh for ZO-1.Tif]

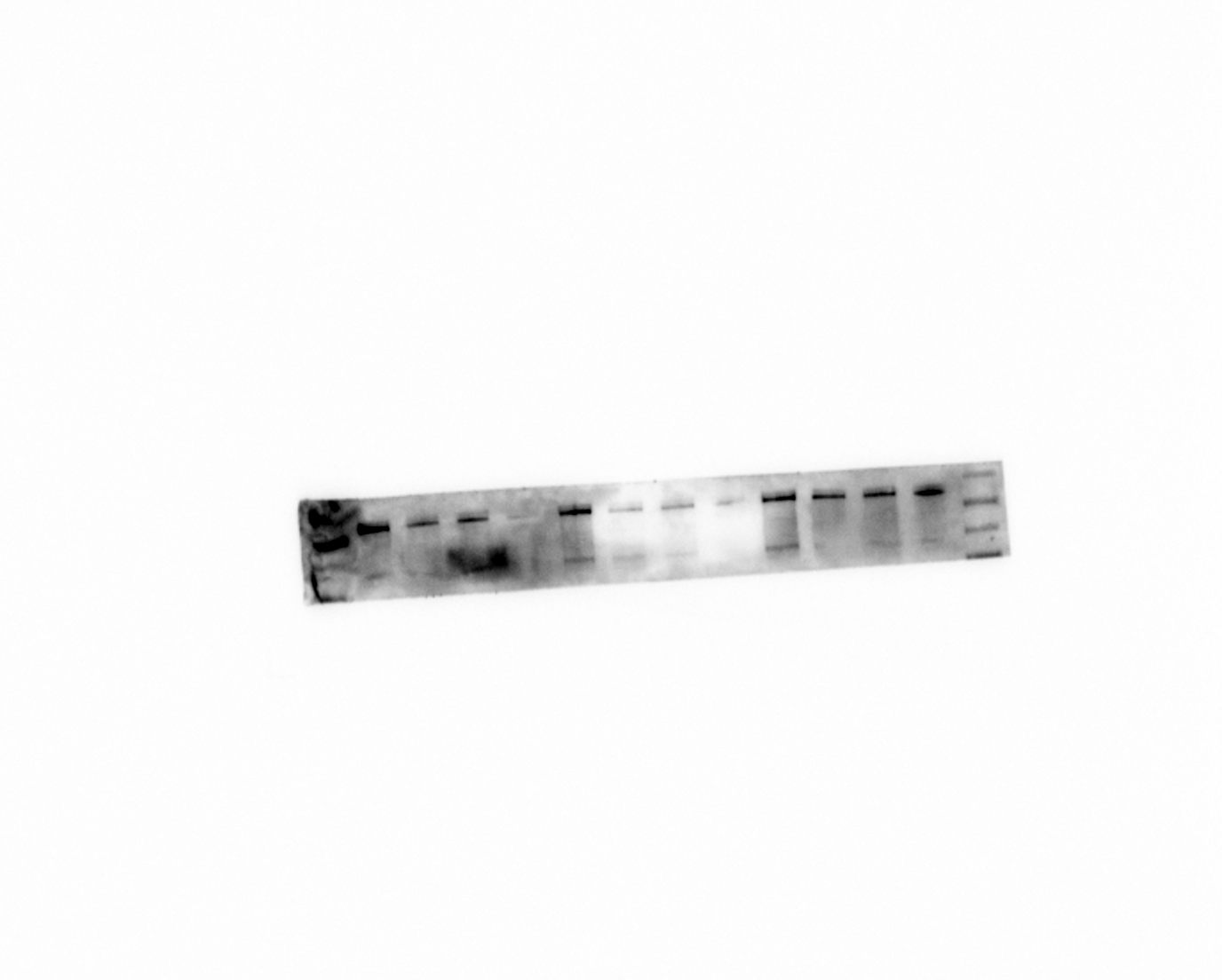

Supplement: Supplementary file 5 — Source Data Fig. 4 [file 44321_2024_25_MOESM5_ESM.zip › figure 4/4G/4G ZO-1 mark replicate.Tif]

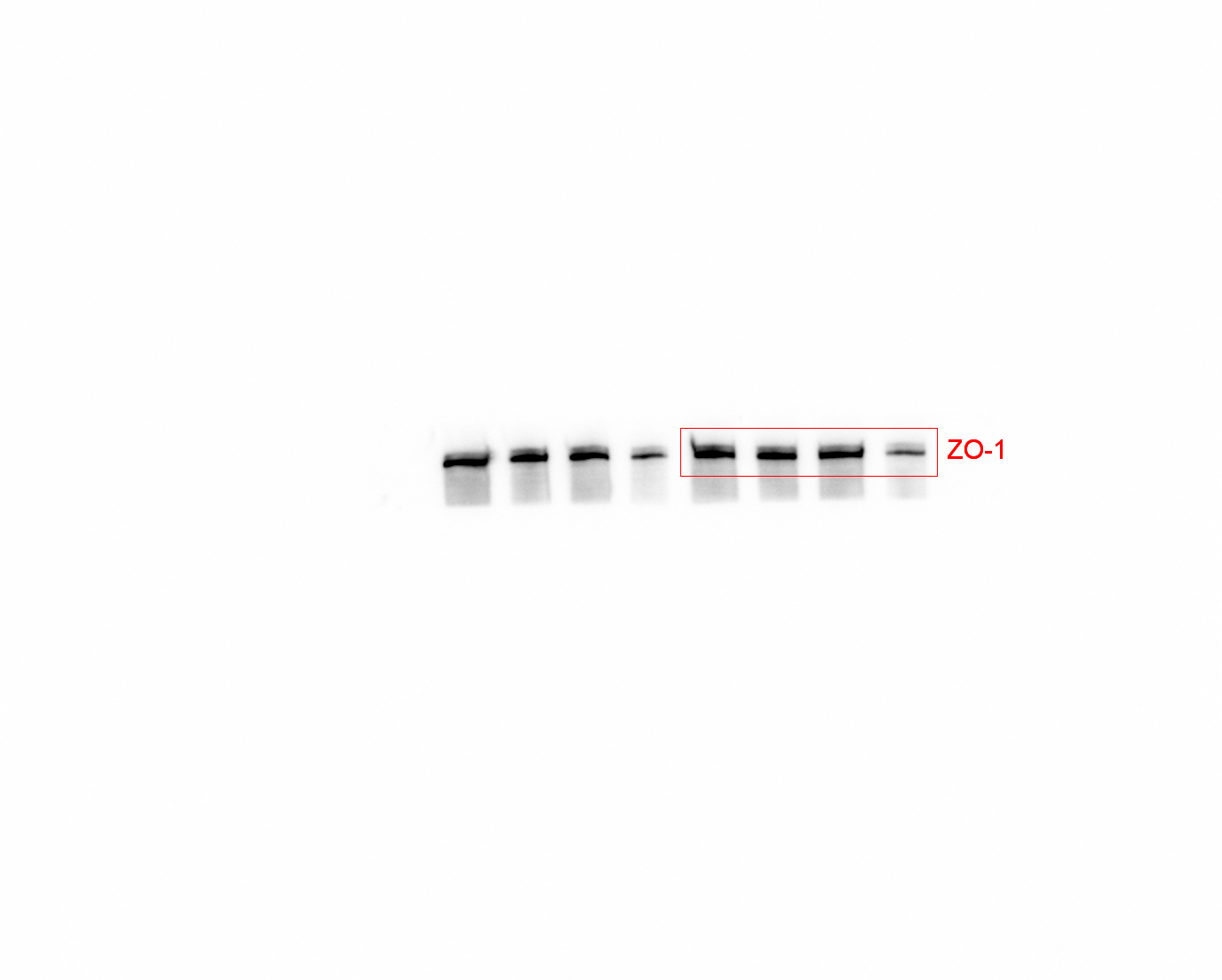

Supplement: Supplementary file 5 — Source Data Fig. 4 [file 44321_2024_25_MOESM5_ESM.zip › figure 4/4G/4G ZO-1 mark.Tif]

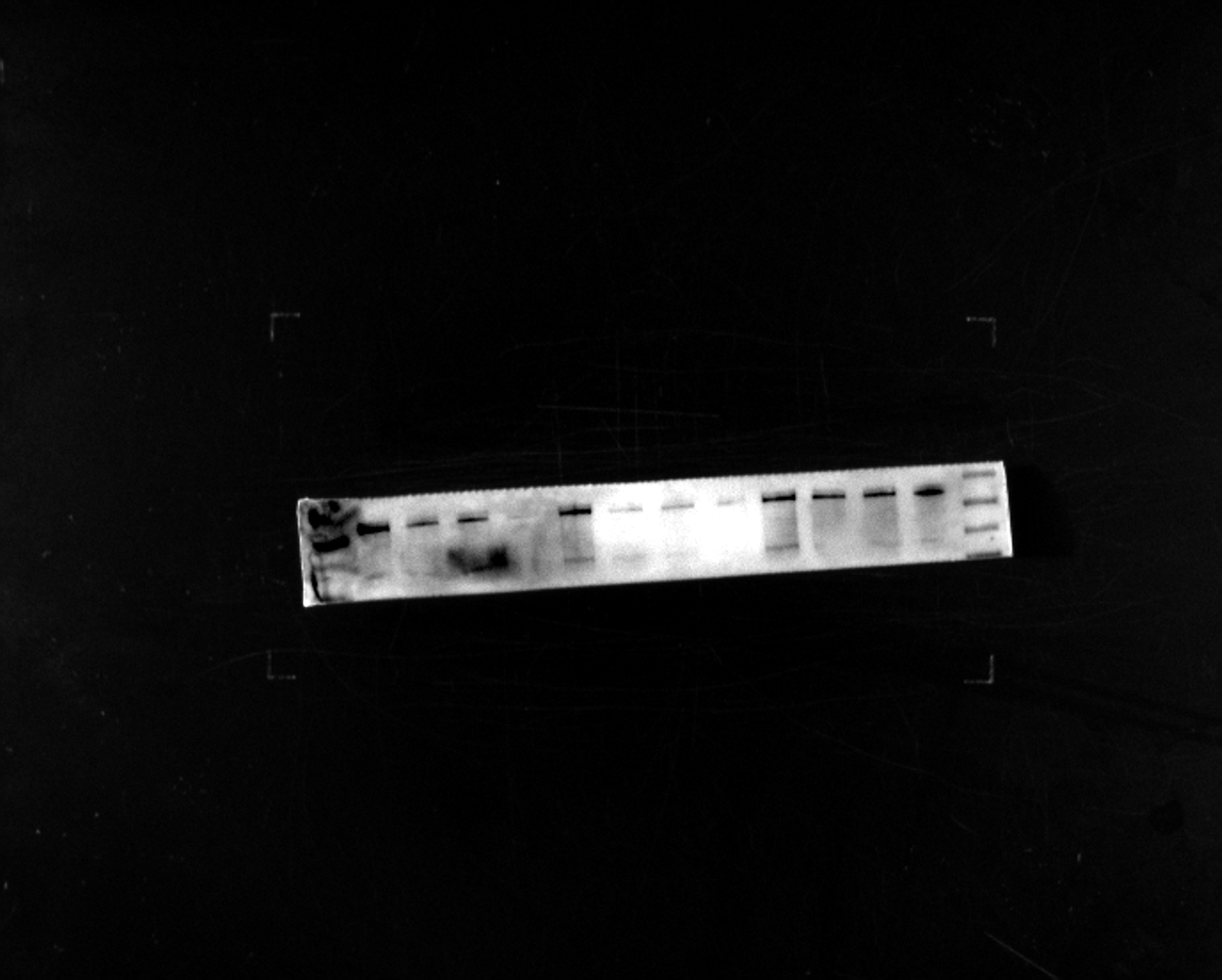

Supplement: Supplementary file 5 — Source Data Fig. 4 [file 44321_2024_25_MOESM5_ESM.zip › figure 4/4G/4G ZO-1 replicate.Tif]

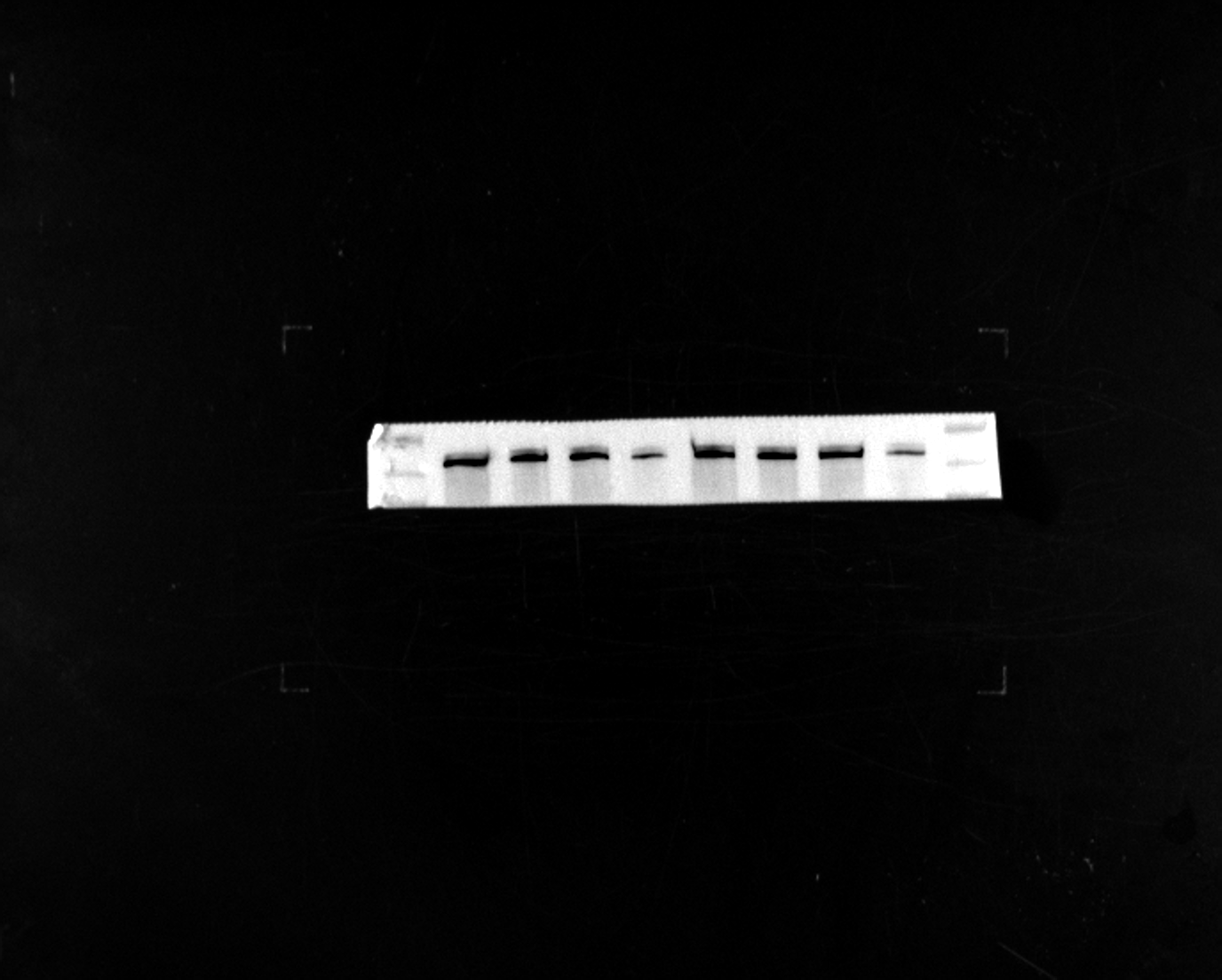

Supplement: Supplementary file 5 — Source Data Fig. 4 [file 44321_2024_25_MOESM5_ESM.zip › figure 4/4G/4G ZO-1.Tif]

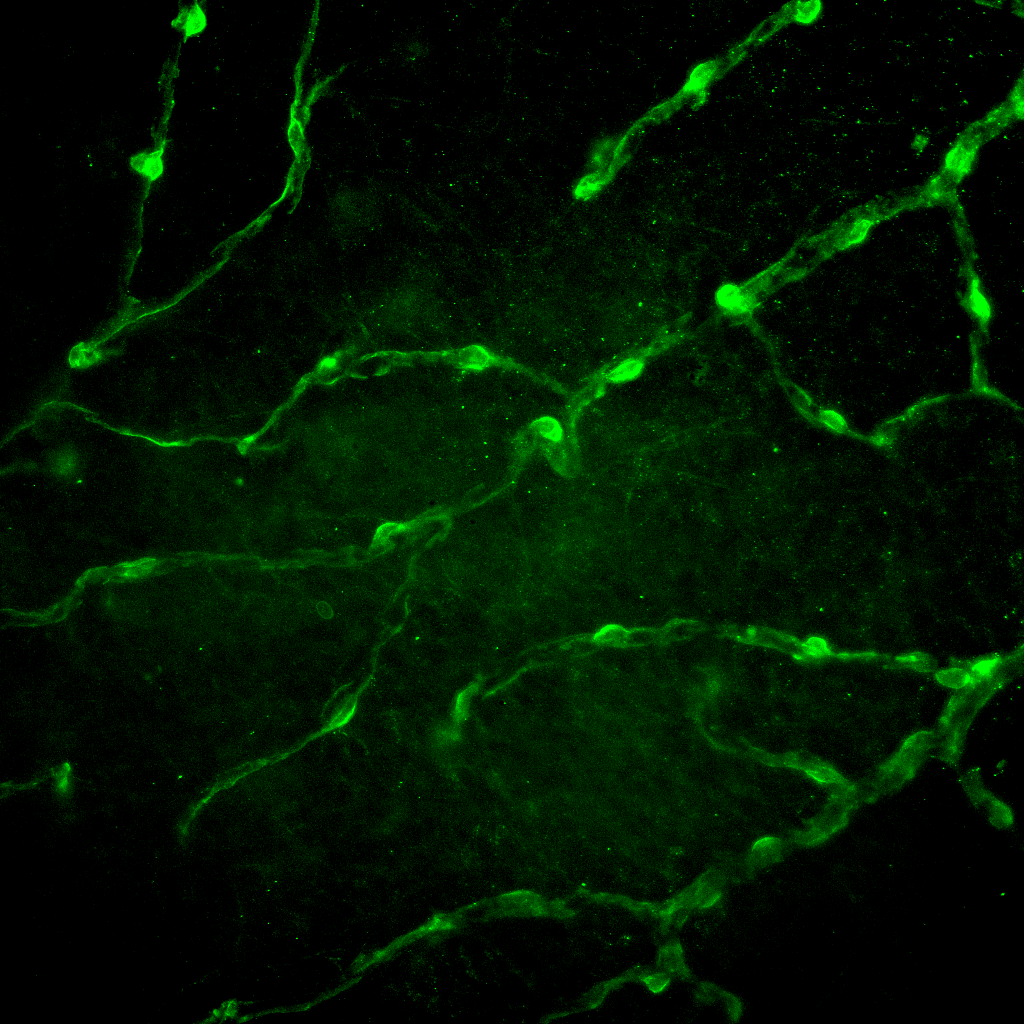

Supplement: Supplementary file 5 — Source Data Fig. 4 [file 44321_2024_25_MOESM5_ESM.zip › figure 4/4H/4H Ctrl NG2.tif]

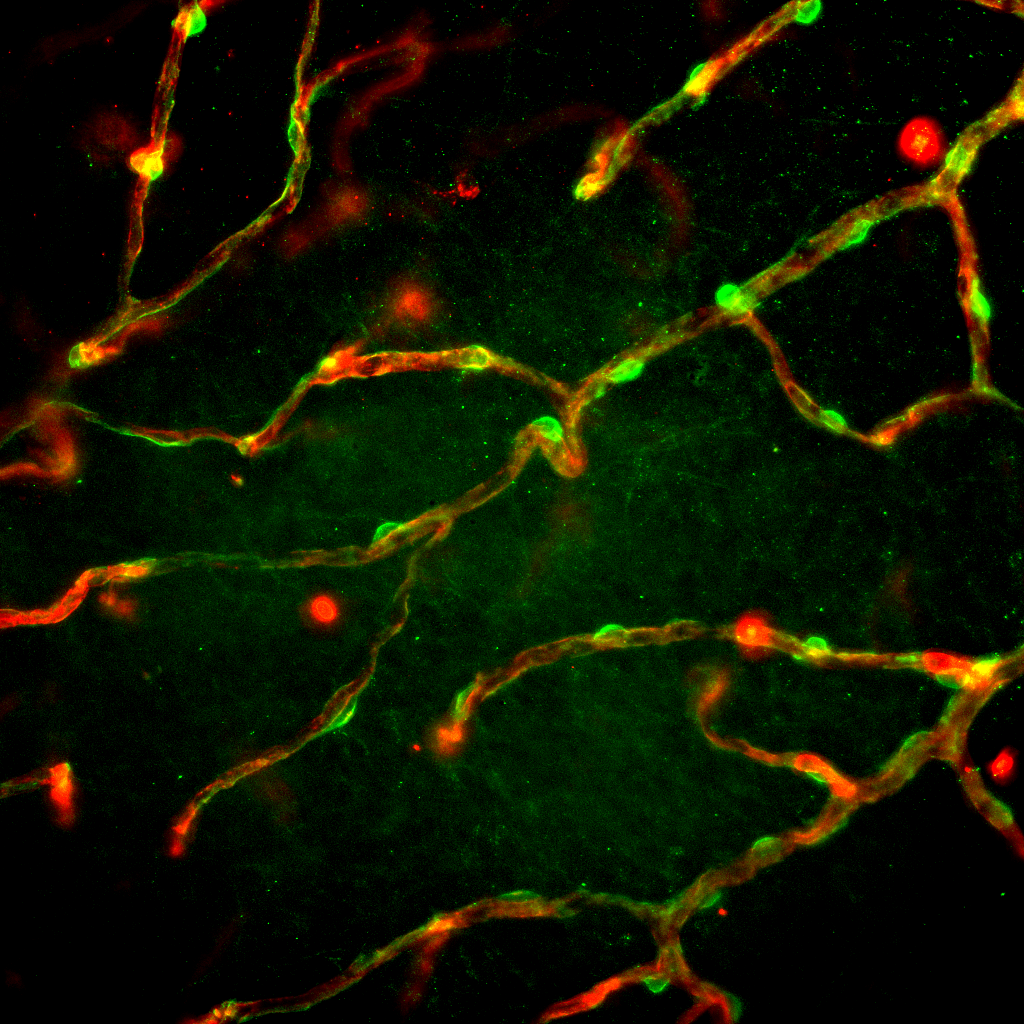

Supplement: Supplementary file 5 — Source Data Fig. 4 [file 44321_2024_25_MOESM5_ESM.zip › figure 4/4H/4H Ctrl NG2+IB4.tif]

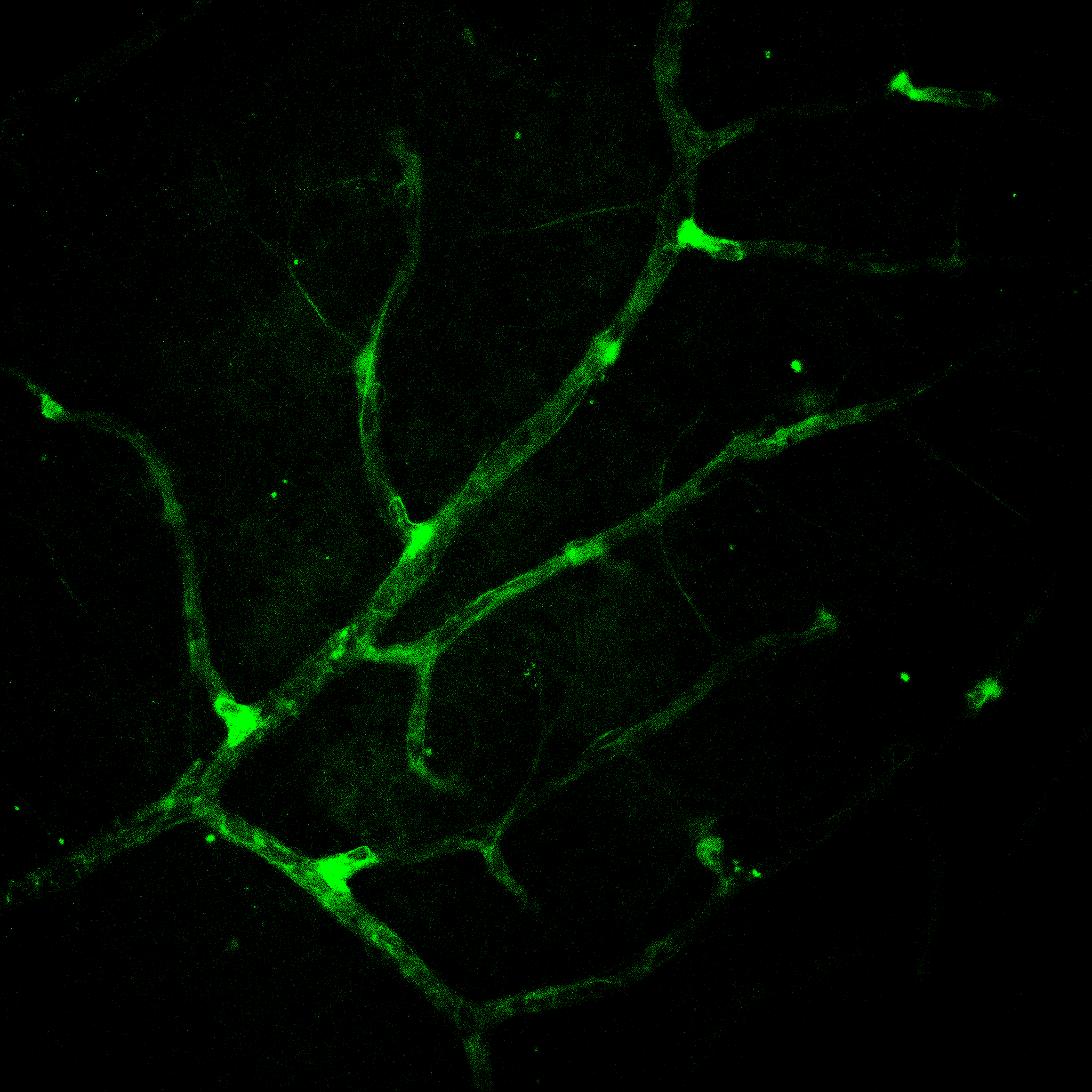

Supplement: Supplementary file 5 — Source Data Fig. 4 [file 44321_2024_25_MOESM5_ESM.zip › figure 4/4H/4H STZ NG2.tif]

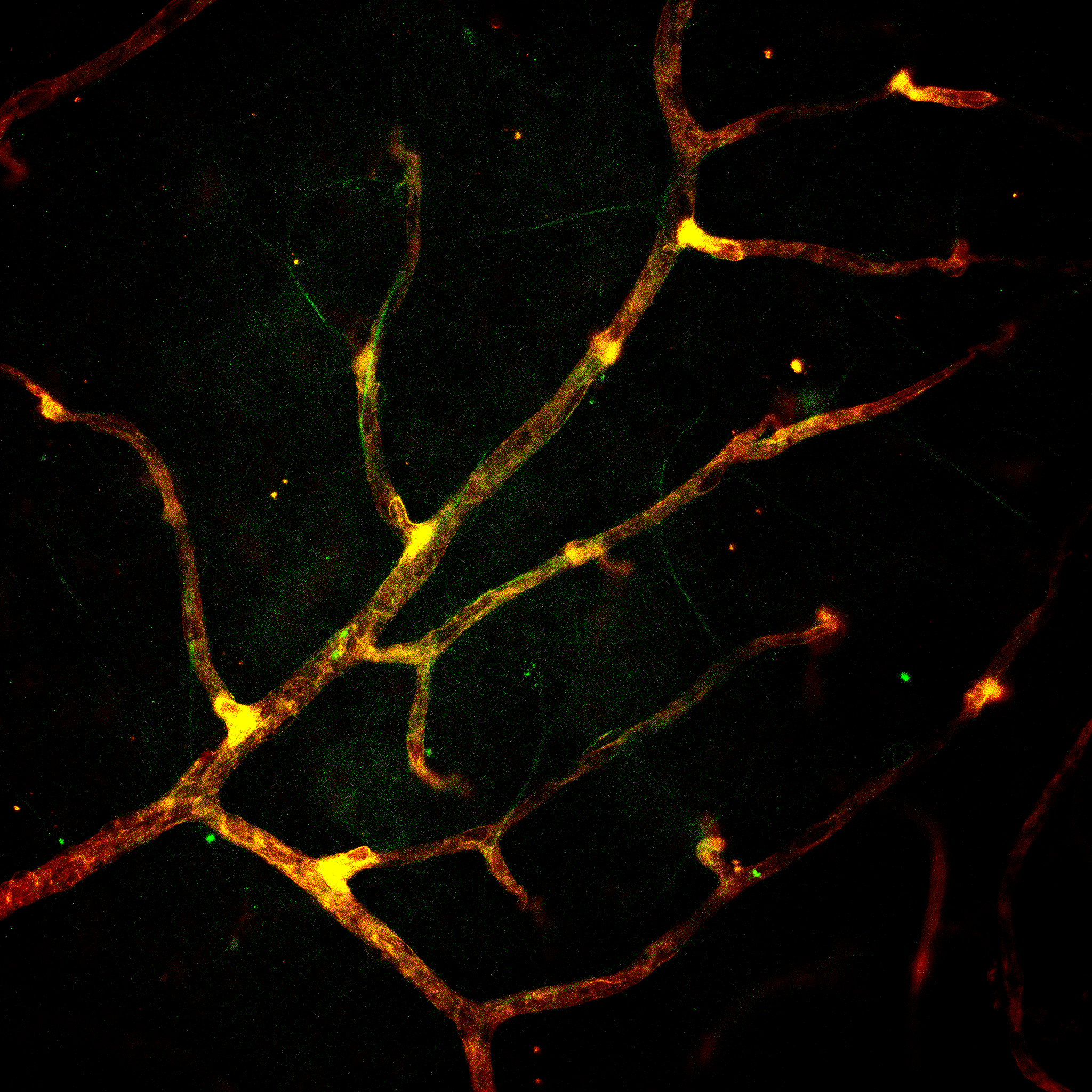

Supplement: Supplementary file 5 — Source Data Fig. 4 [file 44321_2024_25_MOESM5_ESM.zip › figure 4/4H/4H STZ NG2+IB4.tif]

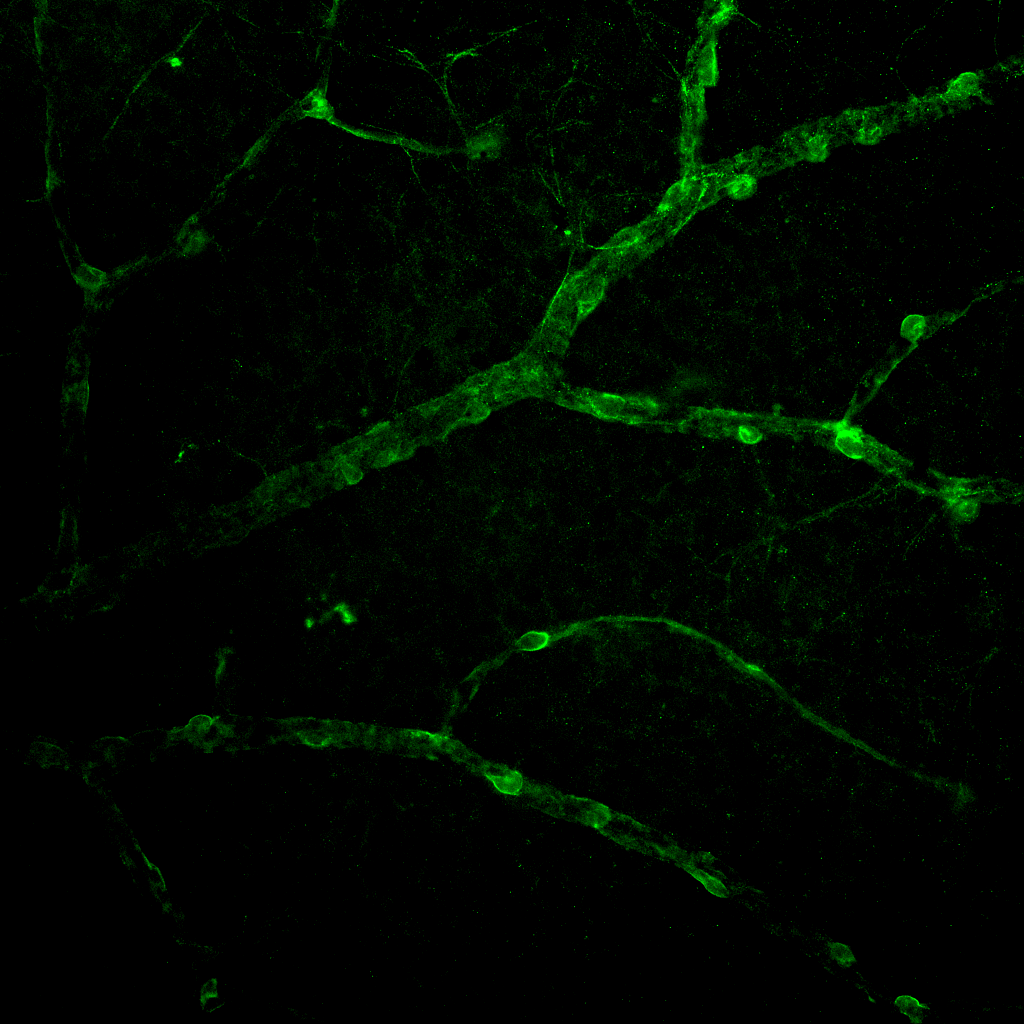

Supplement: Supplementary file 5 — Source Data Fig. 4 [file 44321_2024_25_MOESM5_ESM.zip › figure 4/4H/4H STZ+AAV-blank NG2.tif]

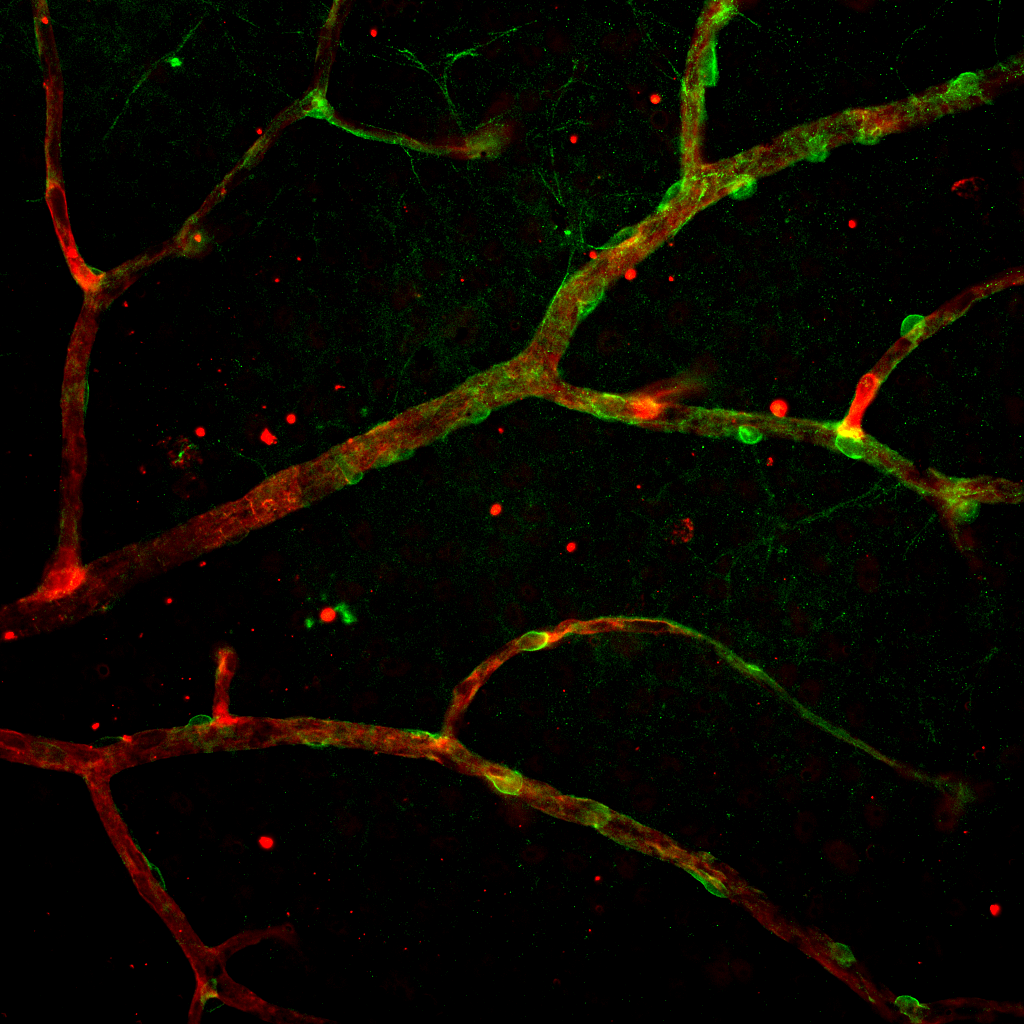

Supplement: Supplementary file 5 — Source Data Fig. 4 [file 44321_2024_25_MOESM5_ESM.zip › figure 4/4H/4H STZ+AAV-blank NG2+IB4.tif]

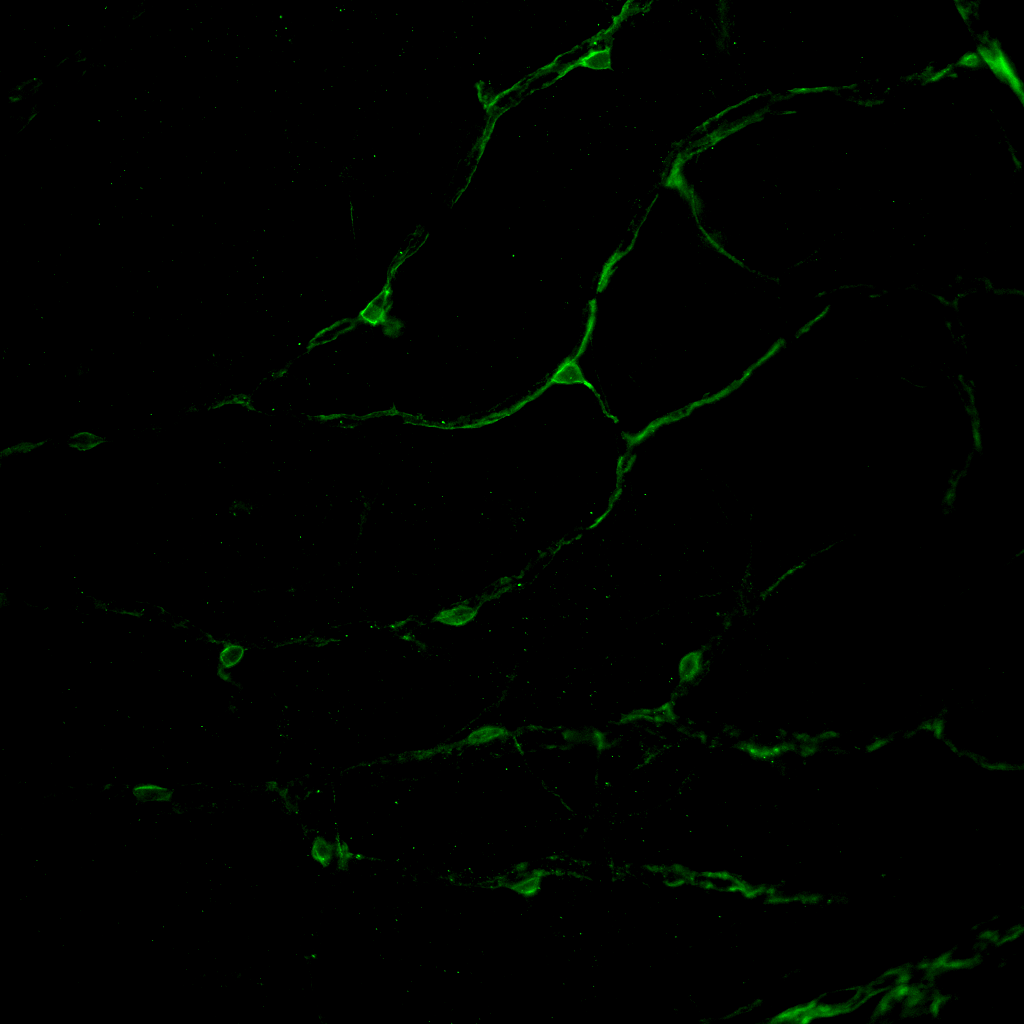

Supplement: Supplementary file 5 — Source Data Fig. 4 [file 44321_2024_25_MOESM5_ESM.zip › figure 4/4H/4H STZ+AAV-Fto NG2.tif]

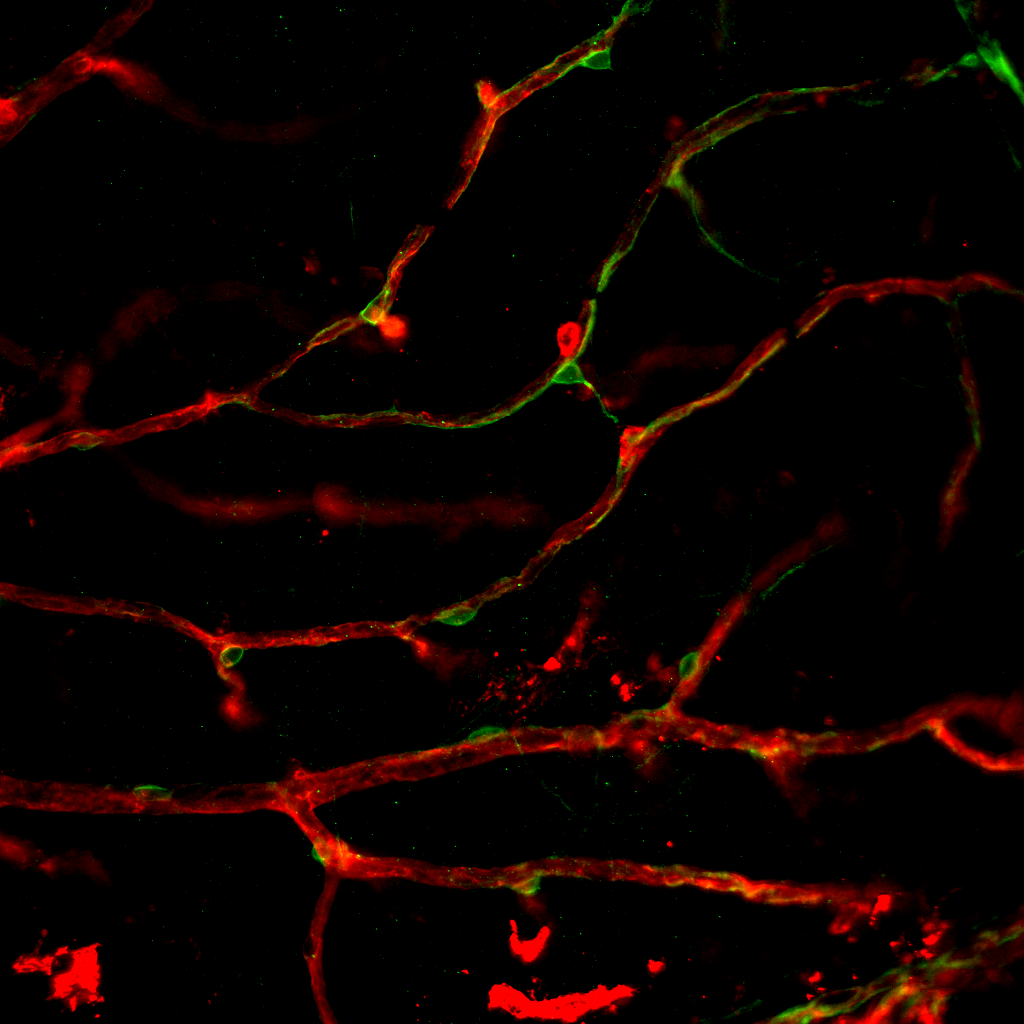

Supplement: Supplementary file 5 — Source Data Fig. 4 [file 44321_2024_25_MOESM5_ESM.zip › figure 4/4H/4H STZ+AAV-Fto NG2+IB4.tif]

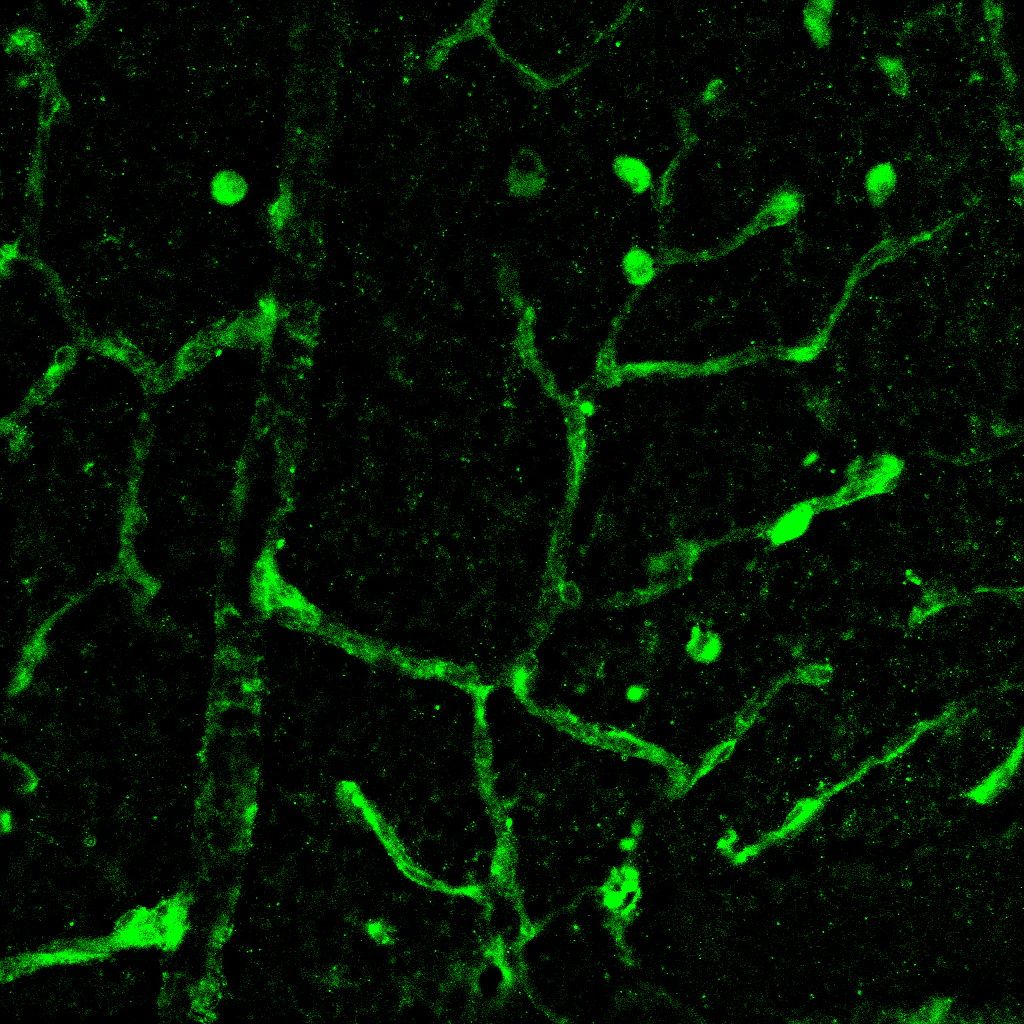

Supplement: Supplementary file 5 — Source Data Fig. 4 [file 44321_2024_25_MOESM5_ESM.zip › figure 4/4I/4I Ctrl PDGFRB.tif]

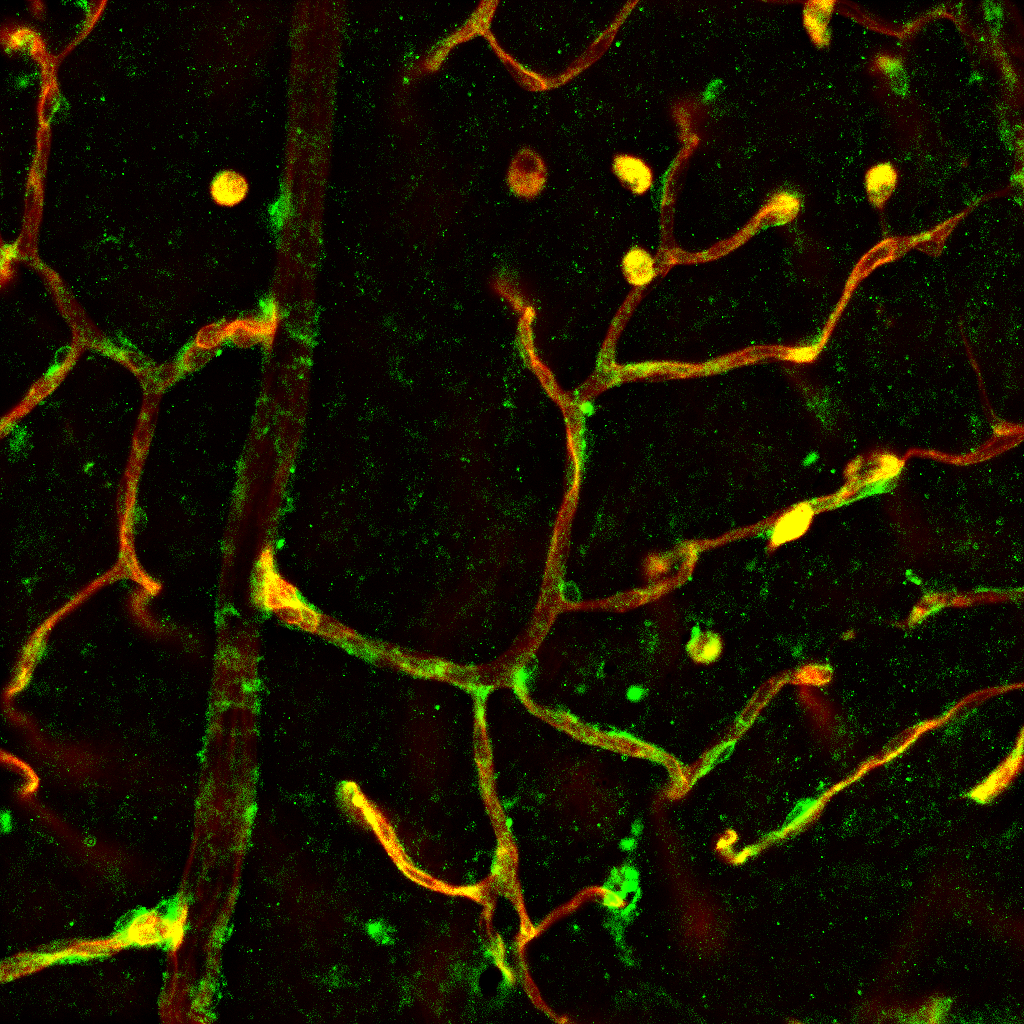

Supplement: Supplementary file 5 — Source Data Fig. 4 [file 44321_2024_25_MOESM5_ESM.zip › figure 4/4I/4I Ctrl PDGFRB+IB4.tif]

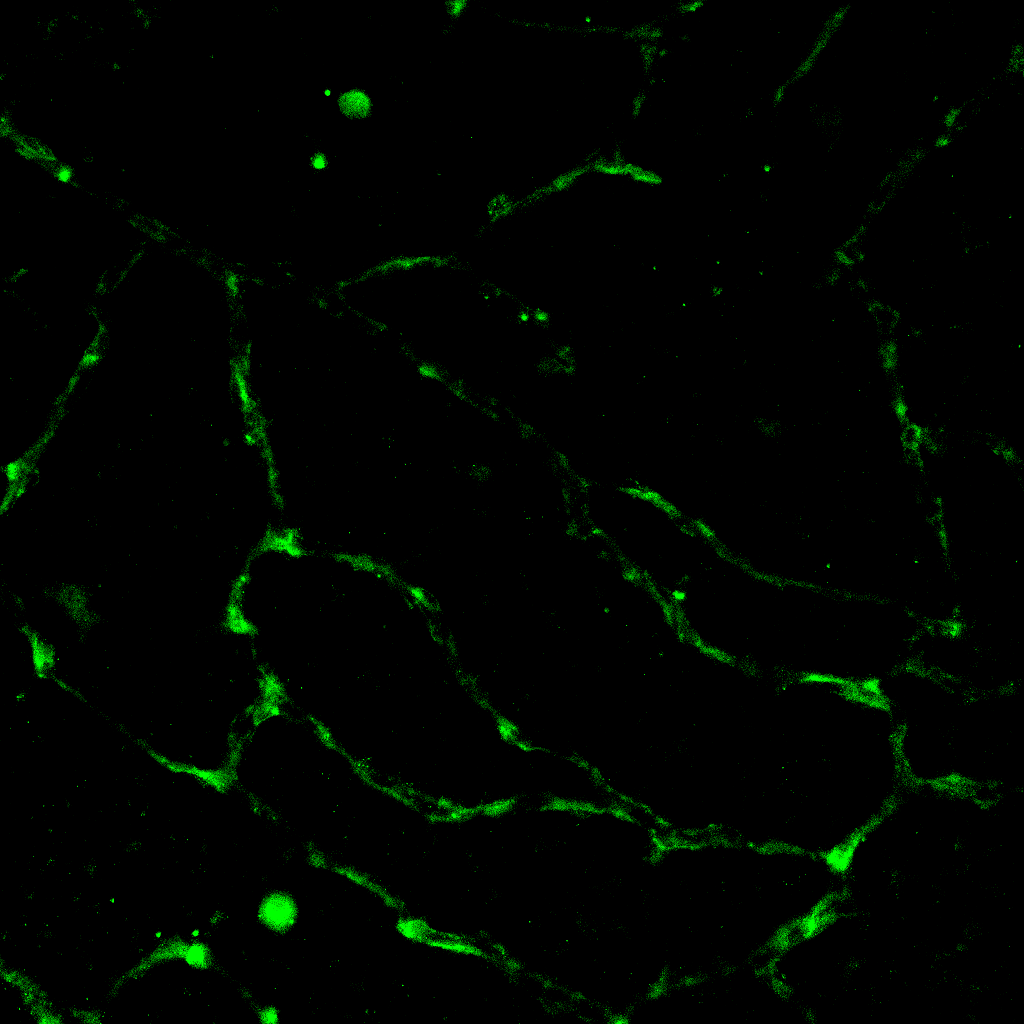

Supplement: Supplementary file 5 — Source Data Fig. 4 [file 44321_2024_25_MOESM5_ESM.zip › figure 4/4I/4I STZ PDGFRB.tif]
