# Supplementary figures and images for: Lactylation-driven FTO targets CDK2 to aggravate microvascular anomalies in diabetic retinopathy (part 3 of 4)
Source: EMBO Mol Med. 2024 Jan 31;16(2):294–318. doi: 10.1038/s44321-024-00025-1 (PMC10897304; doi:10.1038/s44321-024-00025-1)

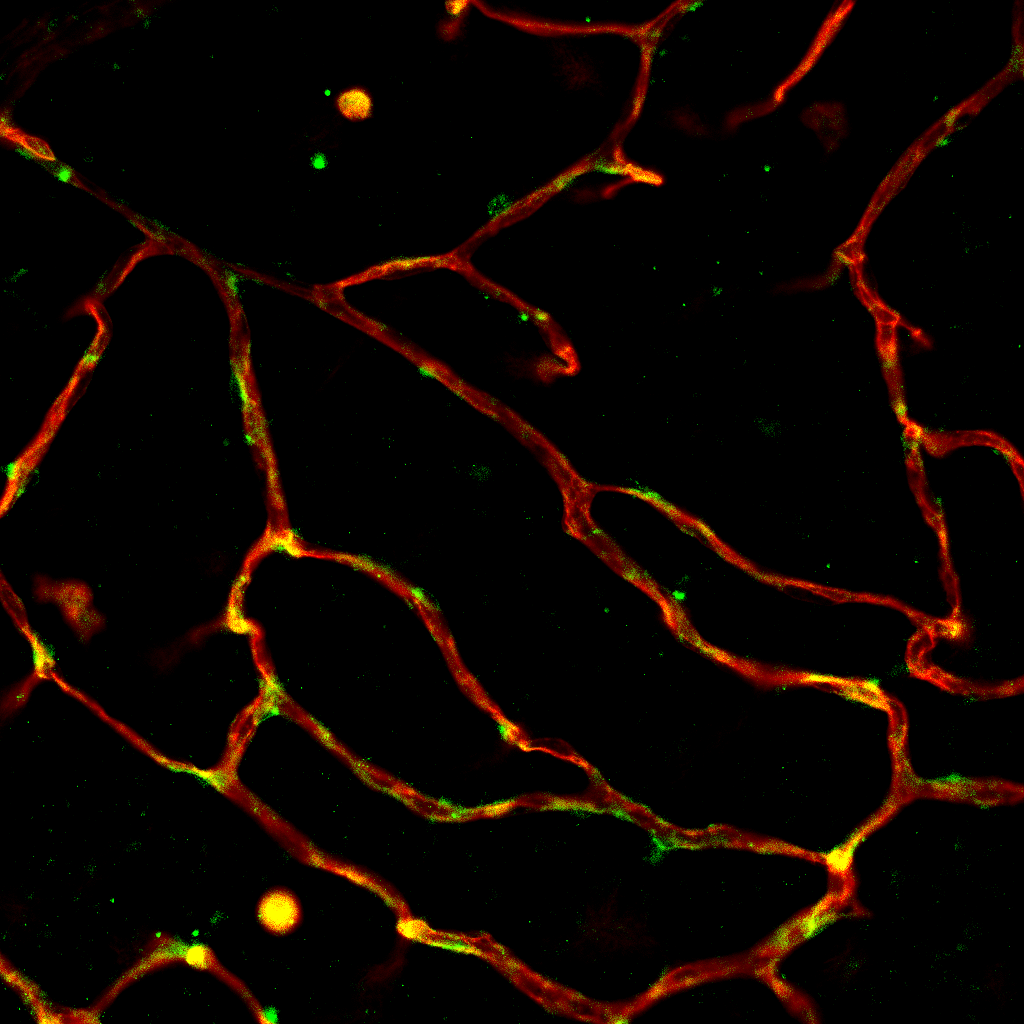

Supplement: Supplementary file 5 — Source Data Fig. 4 [file 44321_2024_25_MOESM5_ESM.zip › figure 4/4I/4I STZ PDGFRB+IB4.tif]

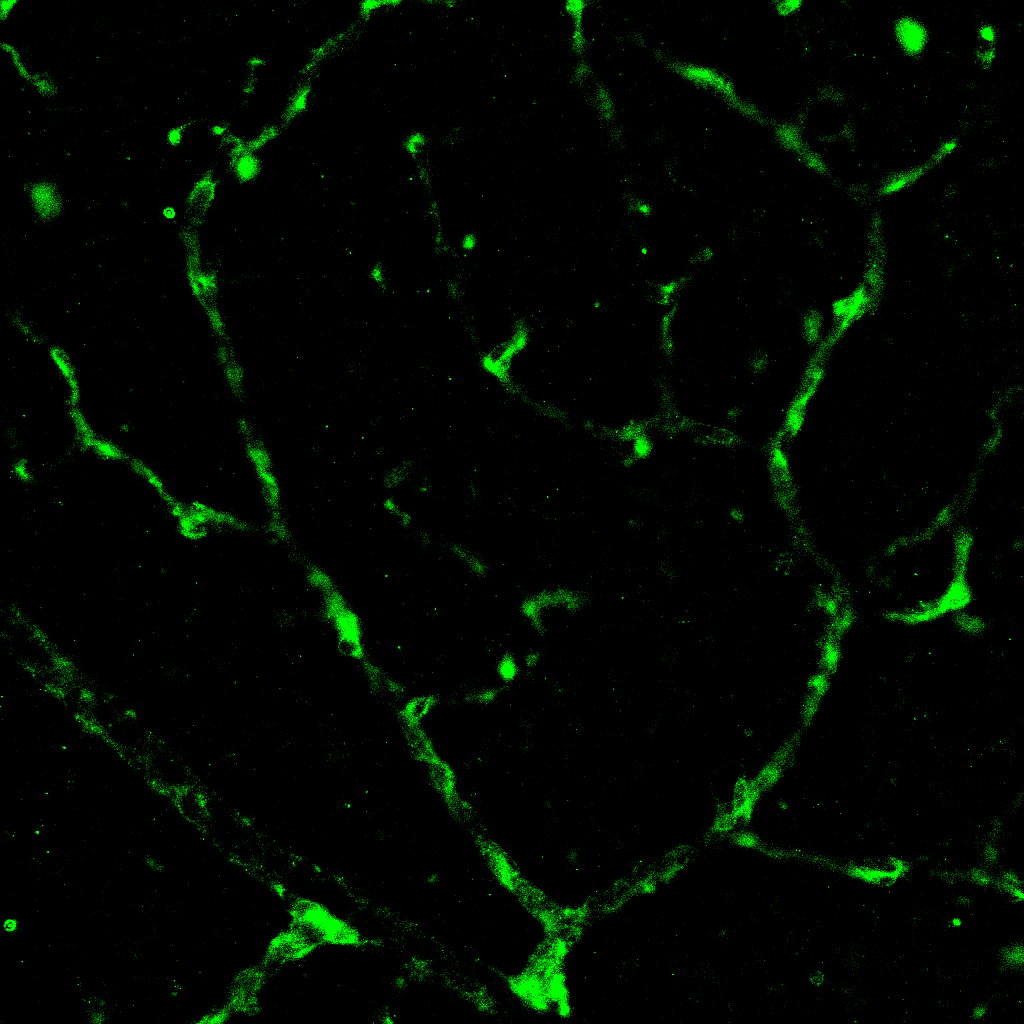

Supplement: Supplementary file 5 — Source Data Fig. 4 [file 44321_2024_25_MOESM5_ESM.zip › figure 4/4I/4I STZ+AAV-blank PDGFRB.tif]

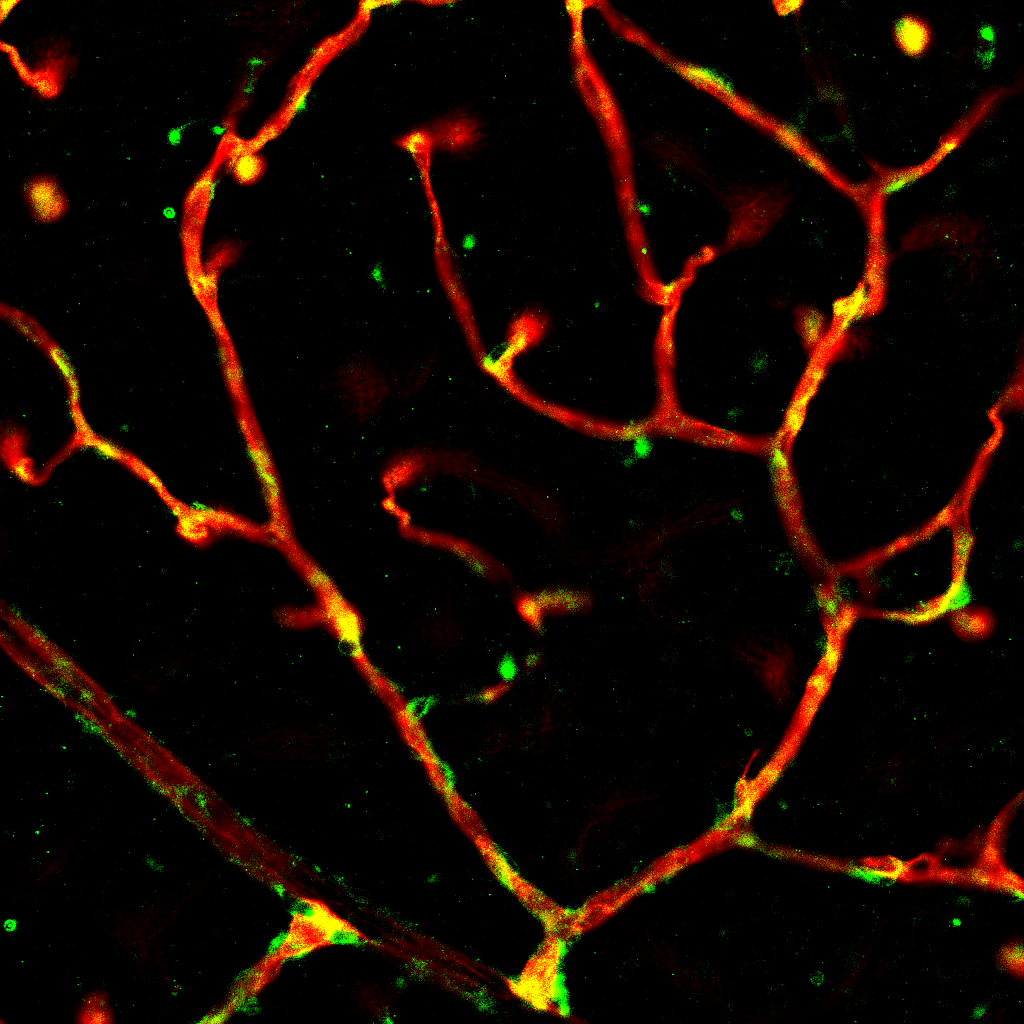

Supplement: Supplementary file 5 — Source Data Fig. 4 [file 44321_2024_25_MOESM5_ESM.zip › figure 4/4I/4I STZ+AAV-blank PDGFRB+IB4.tif]

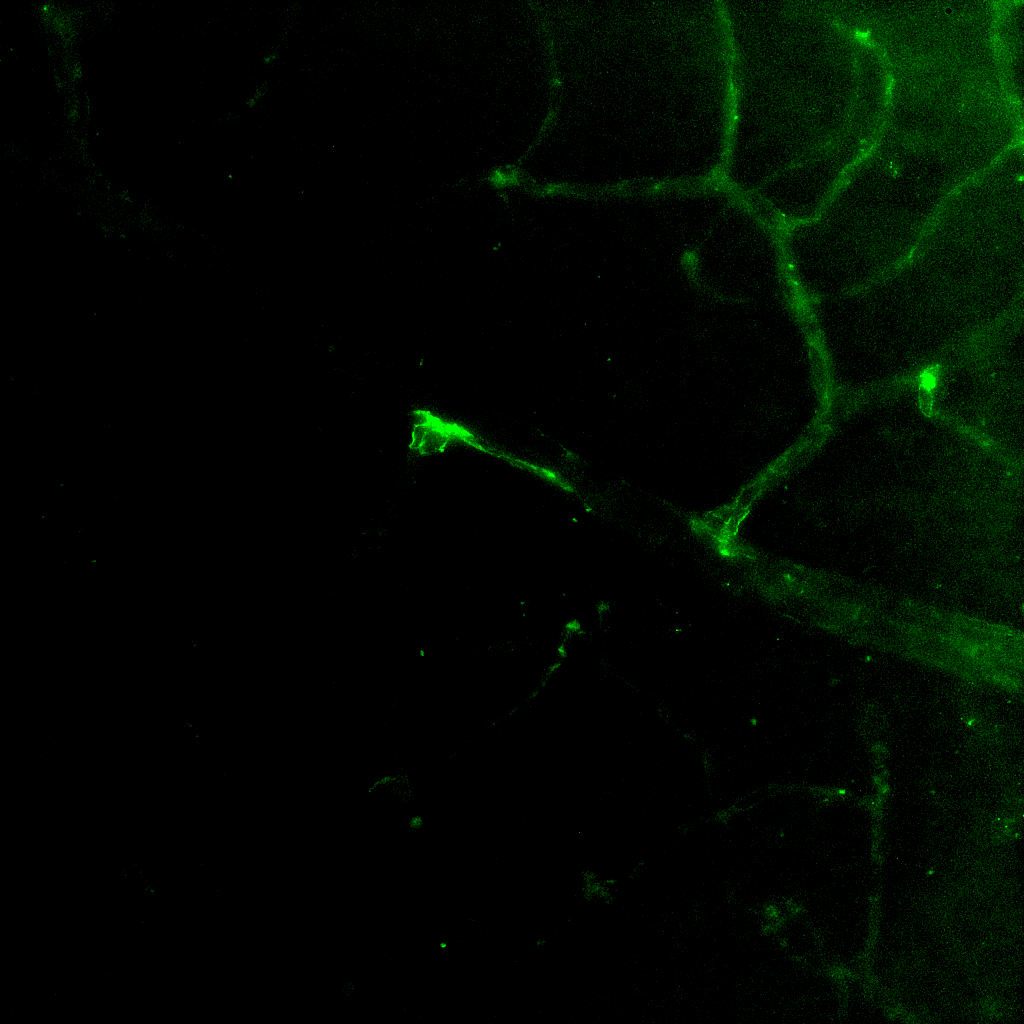

Supplement: Supplementary file 5 — Source Data Fig. 4 [file 44321_2024_25_MOESM5_ESM.zip › figure 4/4I/4I STZ+AAV-Fto PDGFRB.tif]

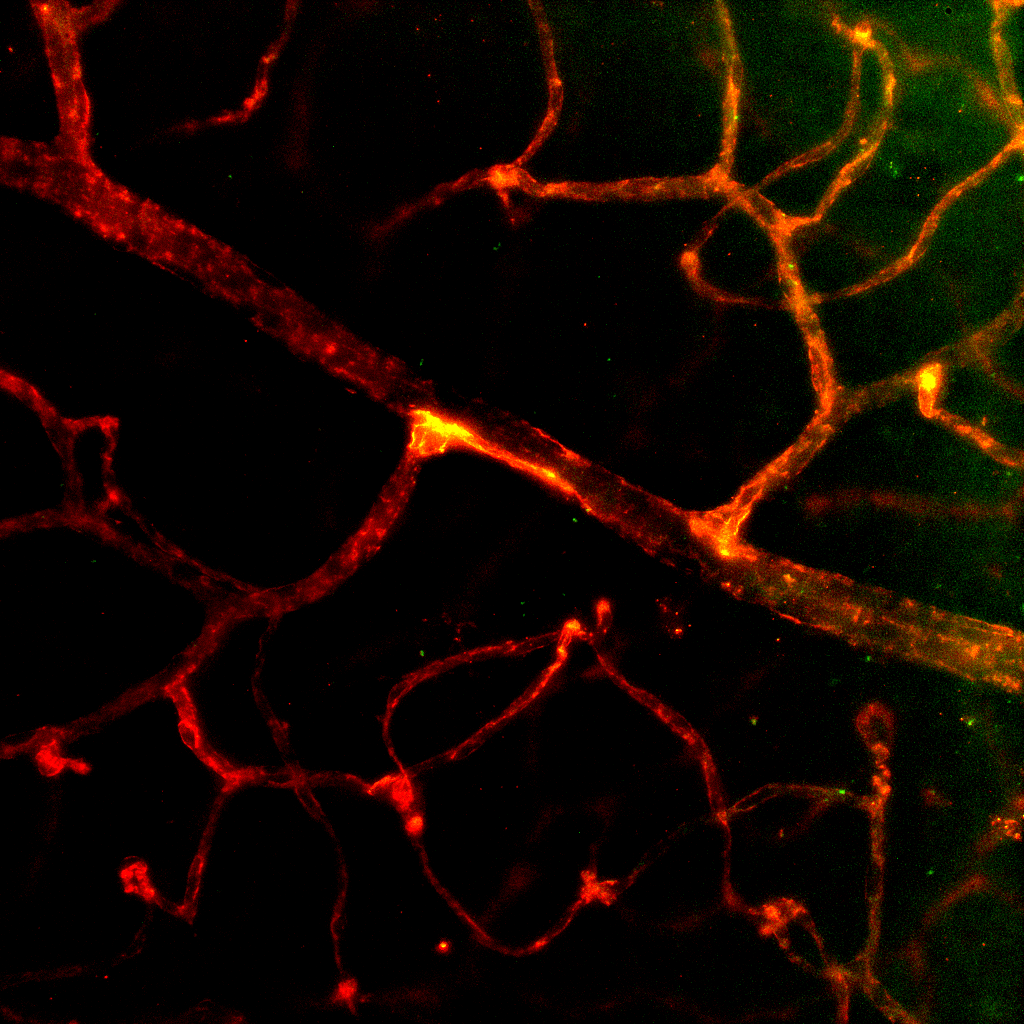

Supplement: Supplementary file 5 — Source Data Fig. 4 [file 44321_2024_25_MOESM5_ESM.zip › figure 4/4I/4I STZ+AAV-Fto PDGFRB+IB4.tif]

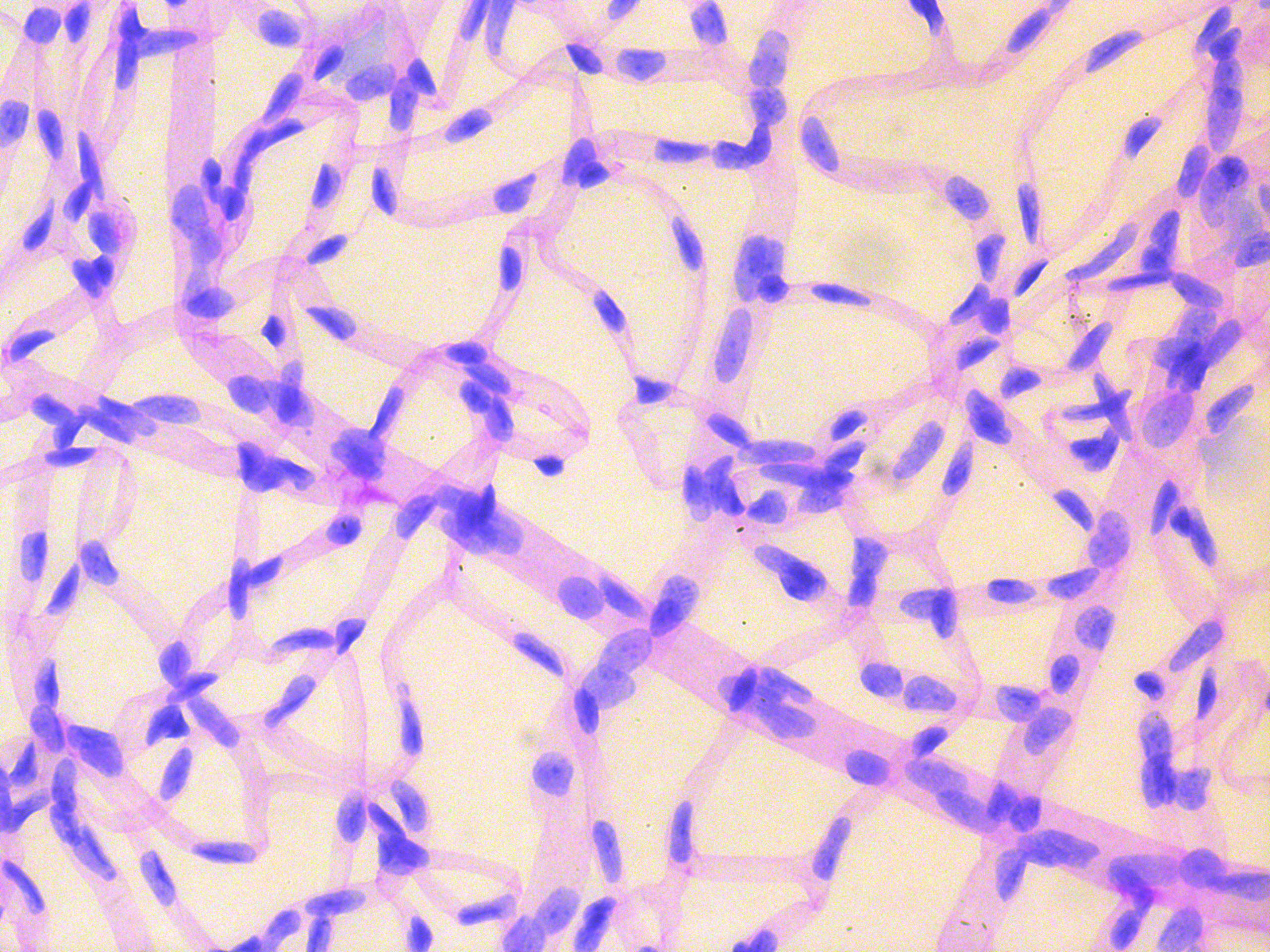

Supplement: Supplementary file 5 — Source Data Fig. 4 [file 44321_2024_25_MOESM5_ESM.zip › figure 4/4J/4J Ctrl.tif]

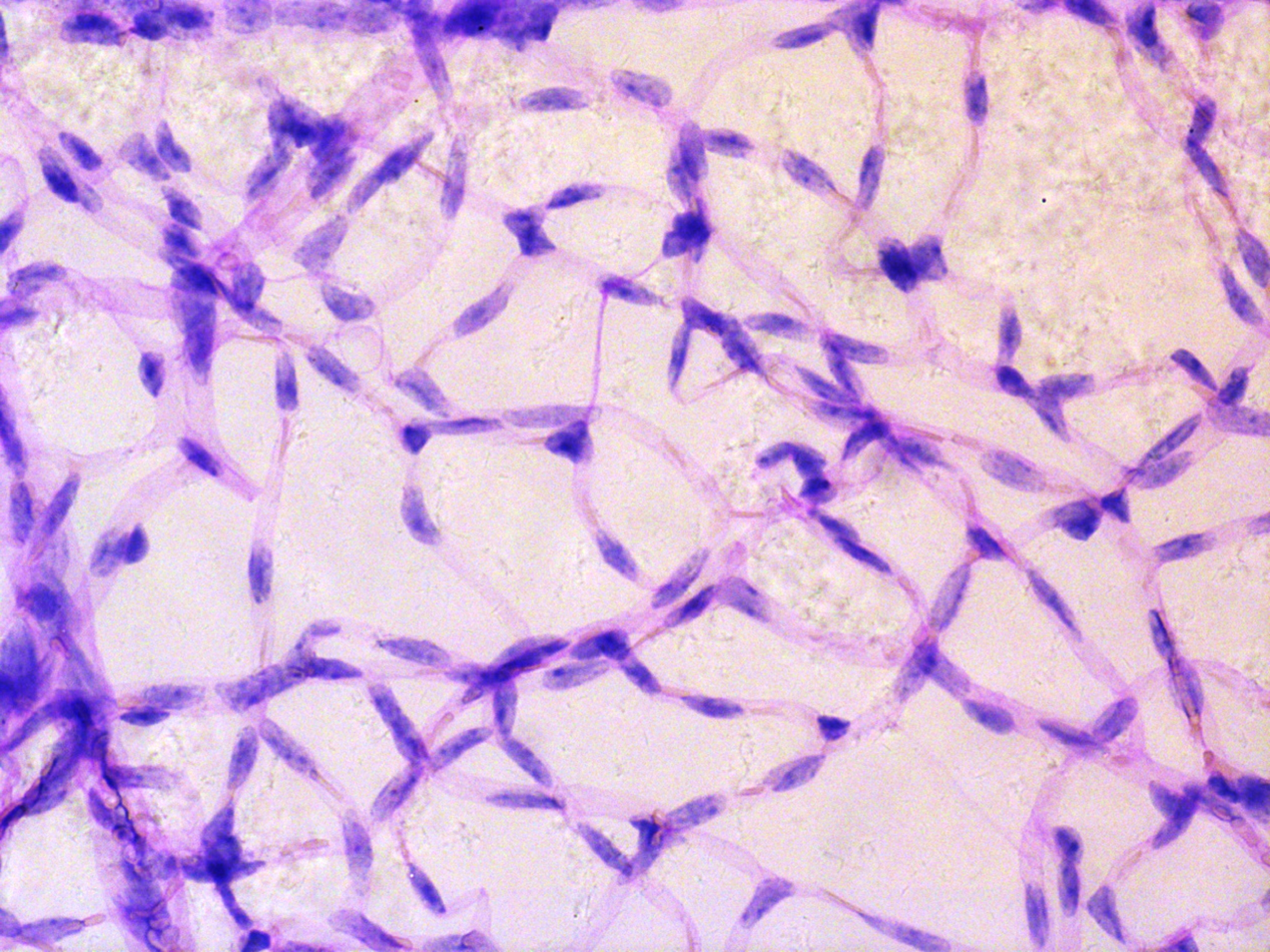

Supplement: Supplementary file 5 — Source Data Fig. 4 [file 44321_2024_25_MOESM5_ESM.zip › figure 4/4J/4J STZ PAS.tif]

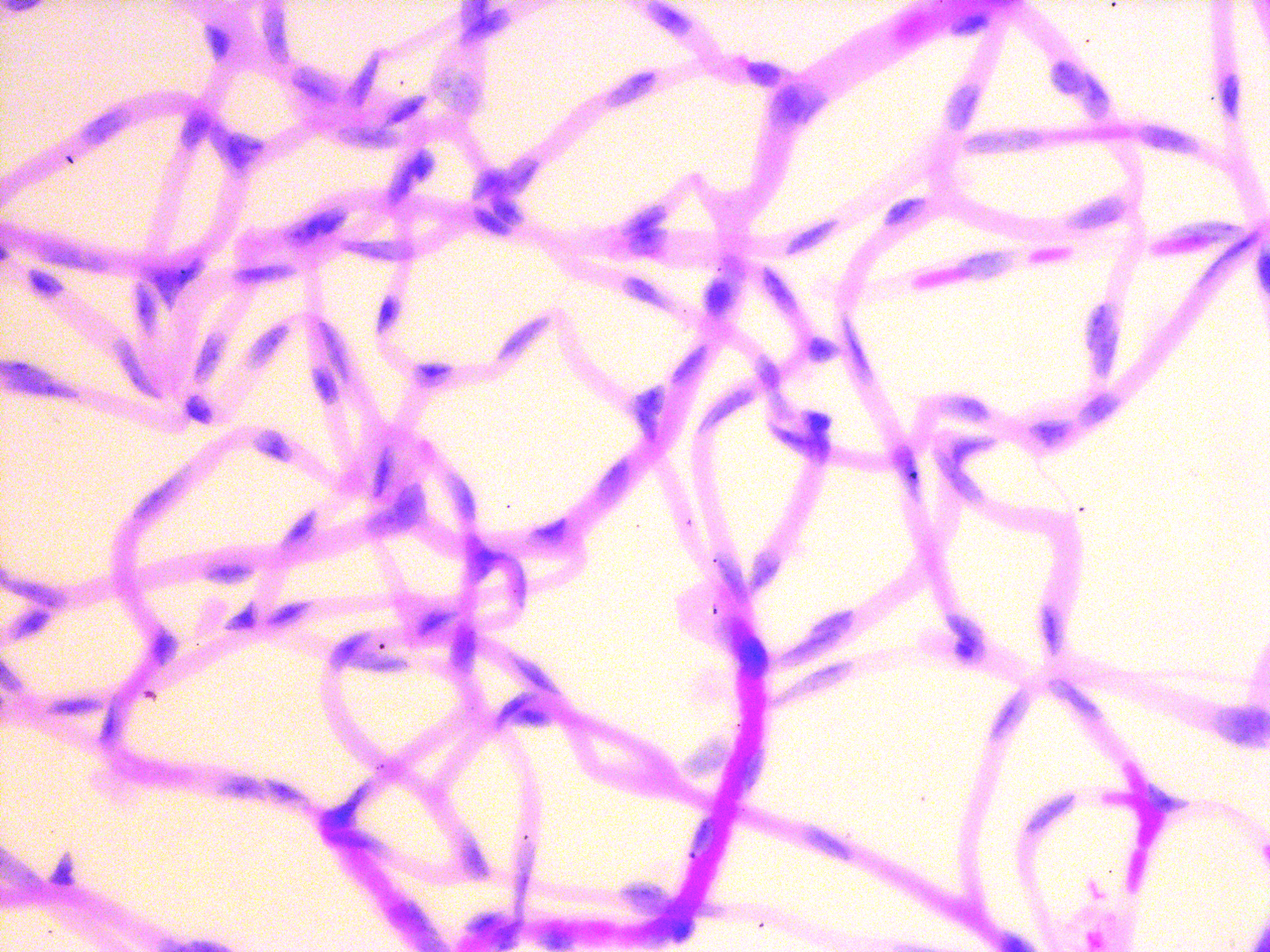

Supplement: Supplementary file 5 — Source Data Fig. 4 [file 44321_2024_25_MOESM5_ESM.zip › figure 4/4J/4J STZ+AAV-blank.tif]

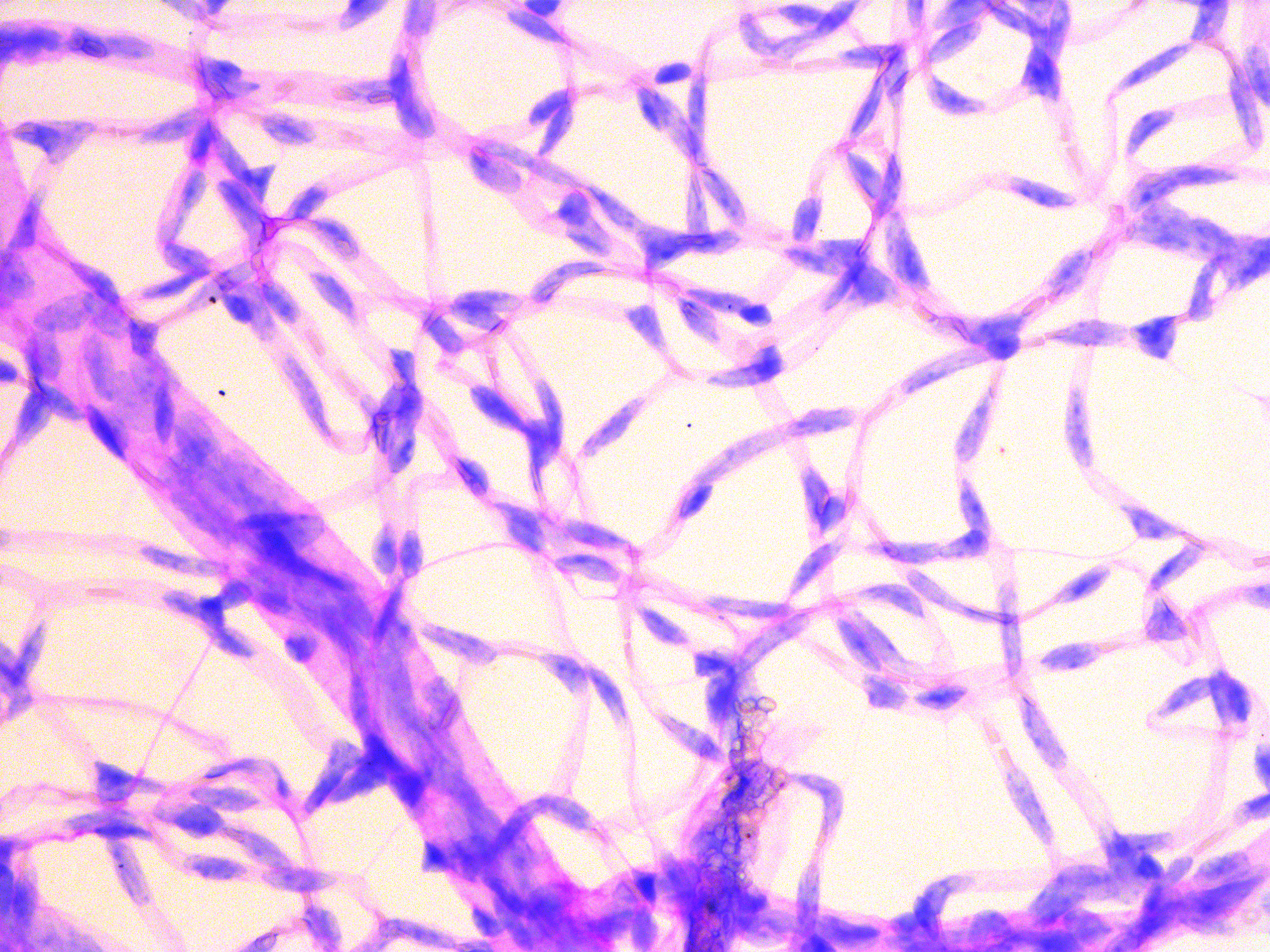

Supplement: Supplementary file 5 — Source Data Fig. 4 [file 44321_2024_25_MOESM5_ESM.zip › figure 4/4J/4J STZ+AAV-Fto.tif]

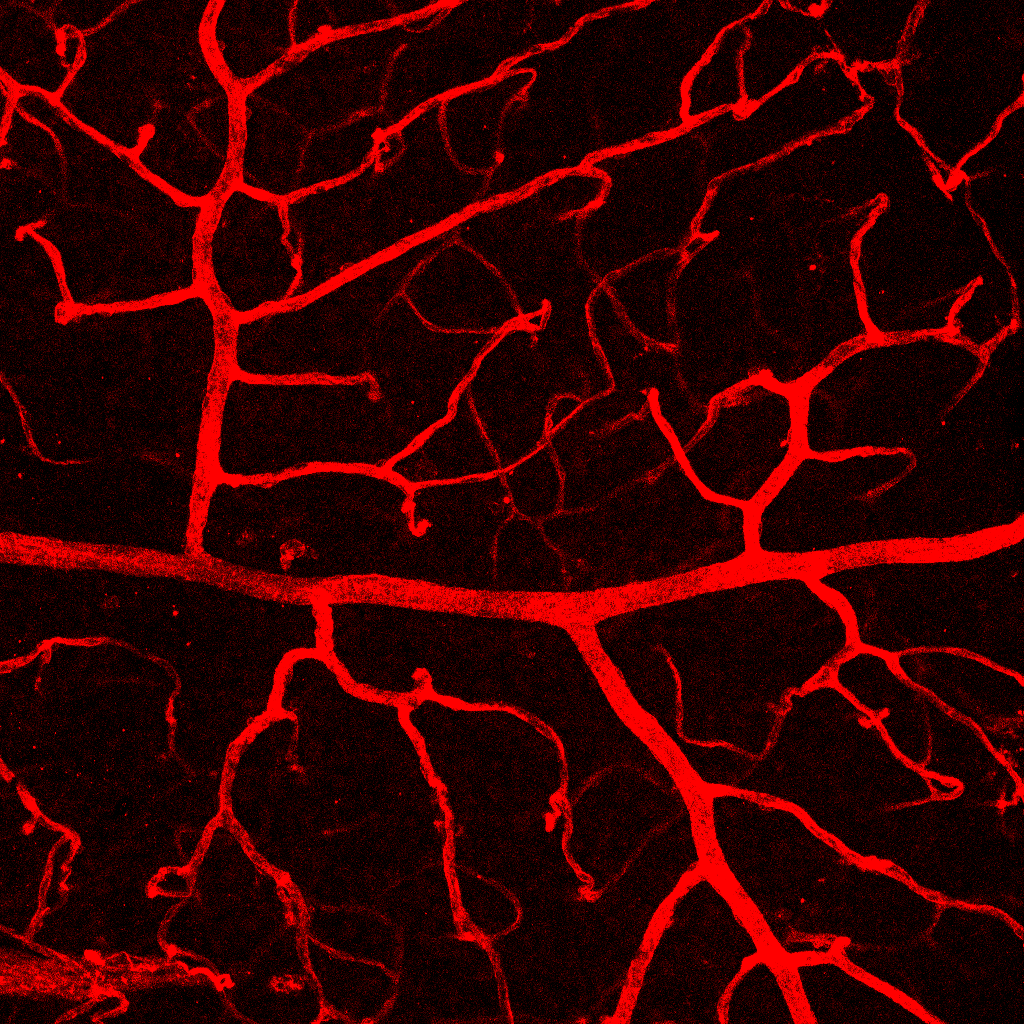

Supplement: Supplementary file 5 — Source Data Fig. 4 [file 44321_2024_25_MOESM5_ESM.zip › figure 4/4K/4K Ctrl lower line left.tif]

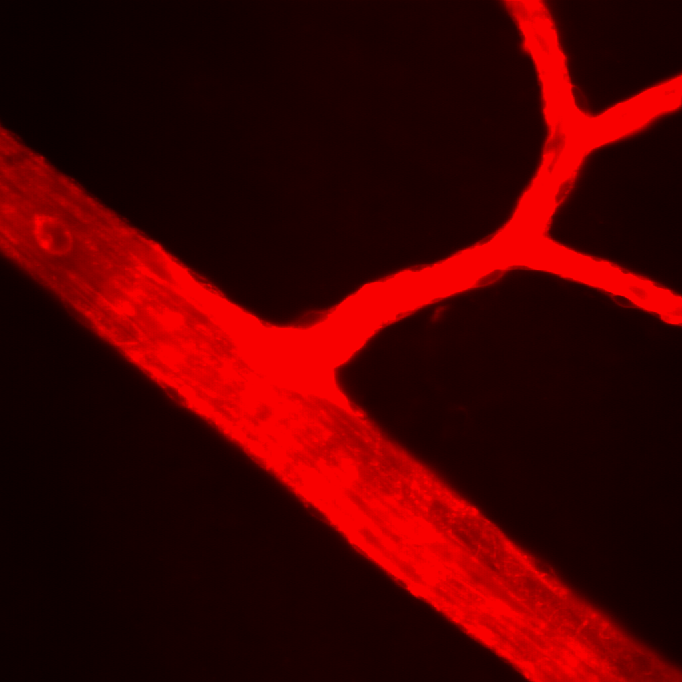

Supplement: Supplementary file 5 — Source Data Fig. 4 [file 44321_2024_25_MOESM5_ESM.zip › figure 4/4K/4K Ctrl lower line right.tif]

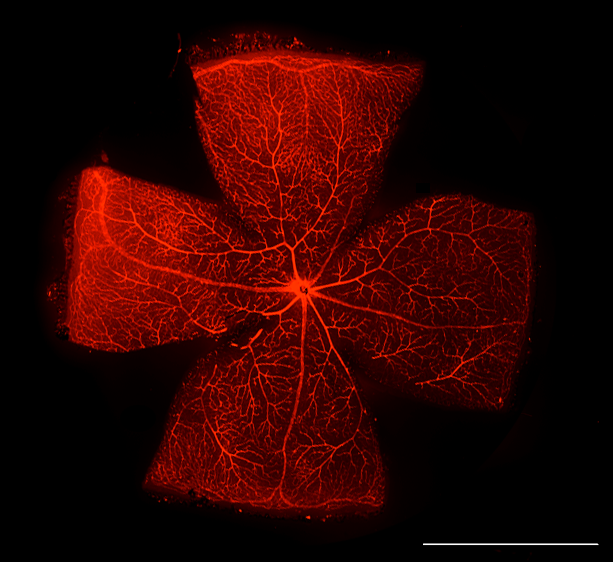

Supplement: Supplementary file 5 — Source Data Fig. 4 [file 44321_2024_25_MOESM5_ESM.zip › figure 4/4K/4K Ctrl upper line.tif]

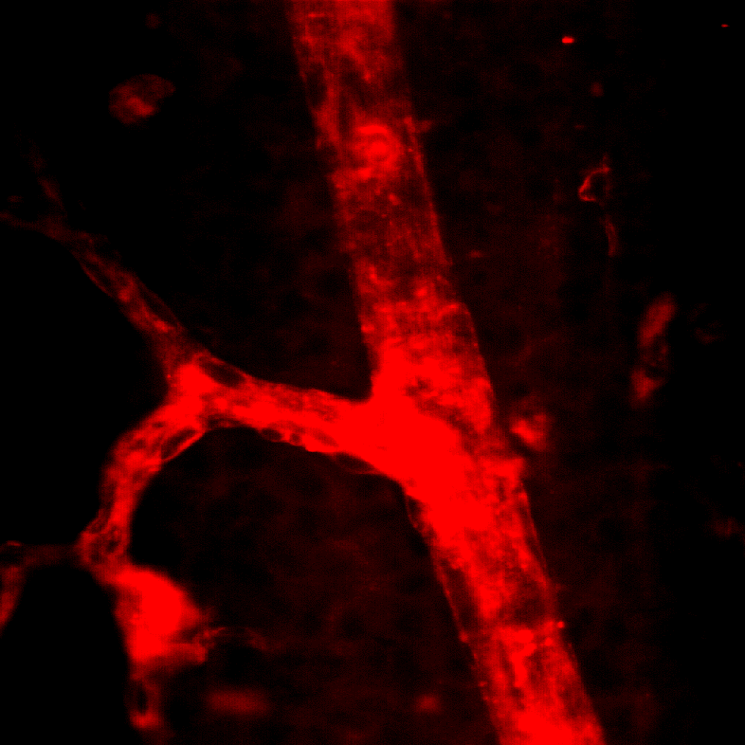

Supplement: Supplementary file 5 — Source Data Fig. 4 [file 44321_2024_25_MOESM5_ESM.zip › figure 4/4K/4K STZ IB4 lower line right.tif]

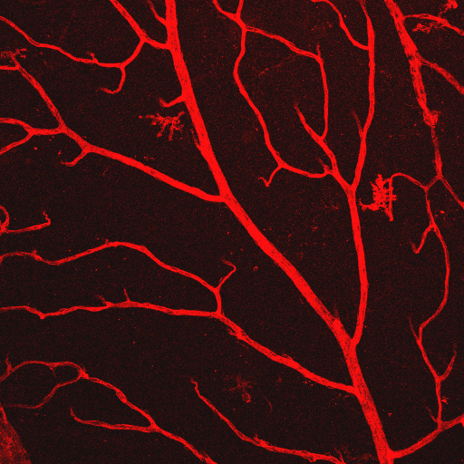

Supplement: Supplementary file 5 — Source Data Fig. 4 [file 44321_2024_25_MOESM5_ESM.zip › figure 4/4K/4K STZ lower line left.tif]

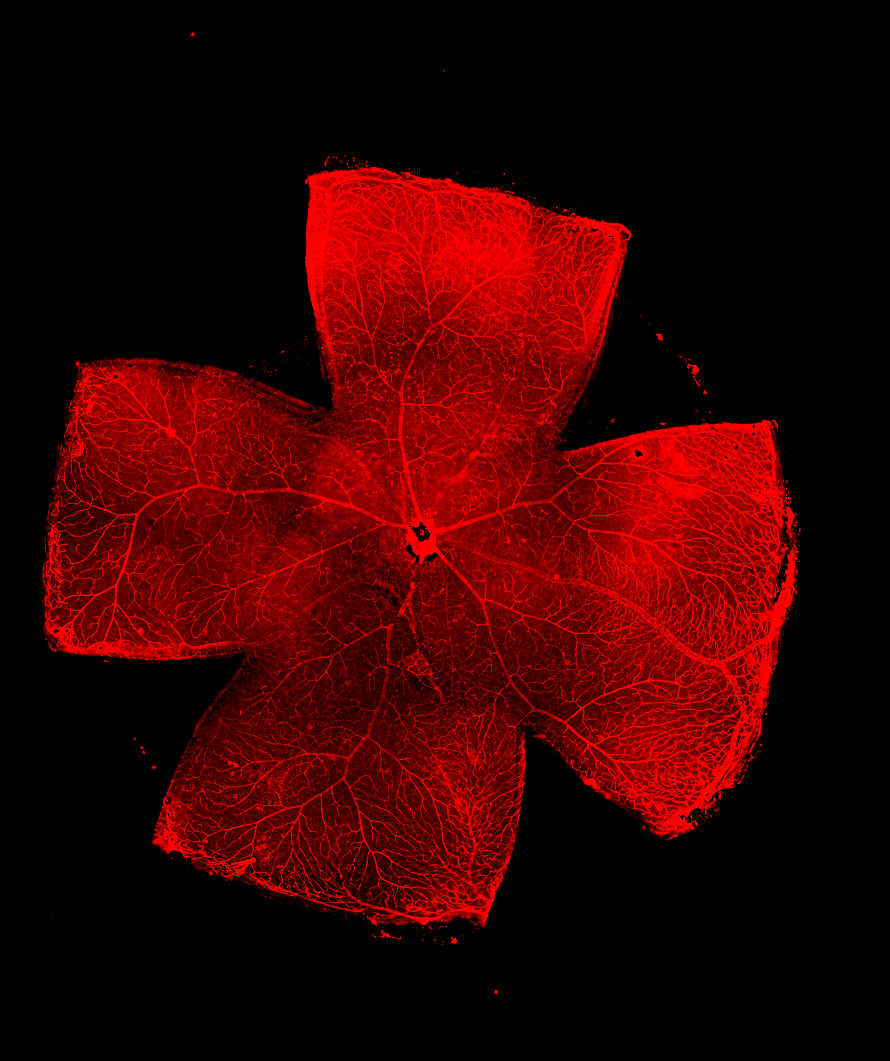

Supplement: Supplementary file 5 — Source Data Fig. 4 [file 44321_2024_25_MOESM5_ESM.zip › figure 4/4K/4K STZ upper line.tif]

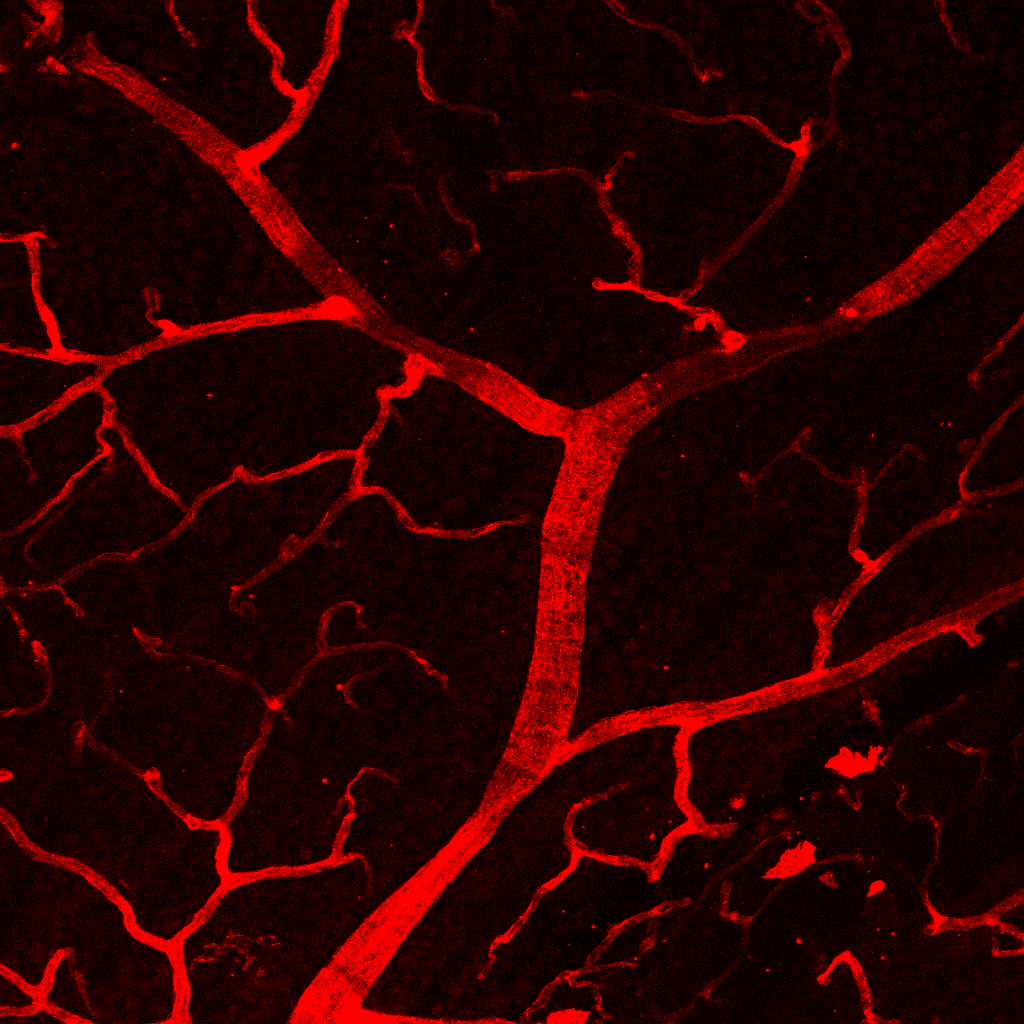

Supplement: Supplementary file 5 — Source Data Fig. 4 [file 44321_2024_25_MOESM5_ESM.zip › figure 4/4K/4K STZ+AAV-blank lower line left.tif]

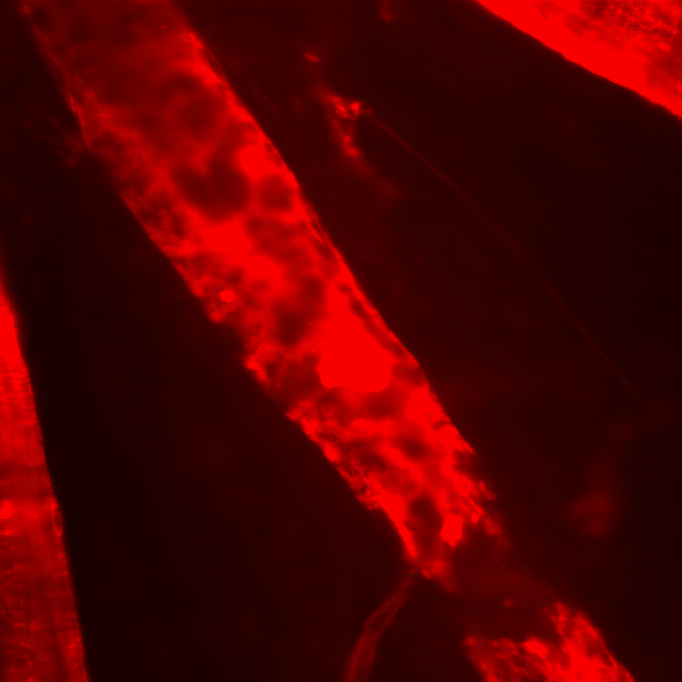

Supplement: Supplementary file 5 — Source Data Fig. 4 [file 44321_2024_25_MOESM5_ESM.zip › figure 4/4K/4K STZ+AAV-blank lower line right.tif]

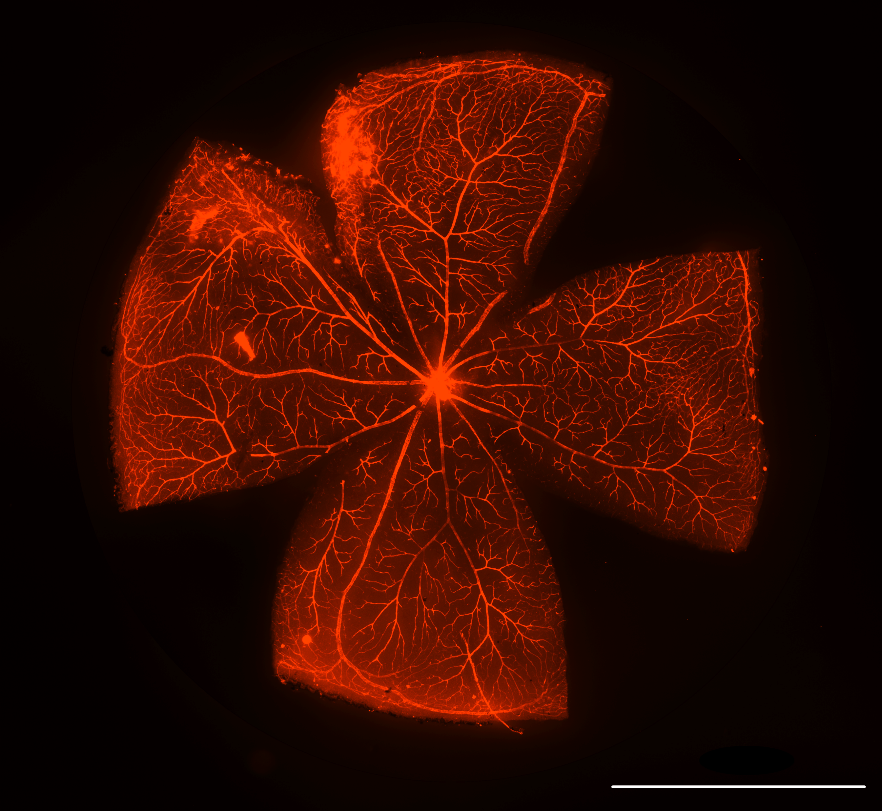

Supplement: Supplementary file 5 — Source Data Fig. 4 [file 44321_2024_25_MOESM5_ESM.zip › figure 4/4K/4K STZ+AAV-blank upper line.tif]

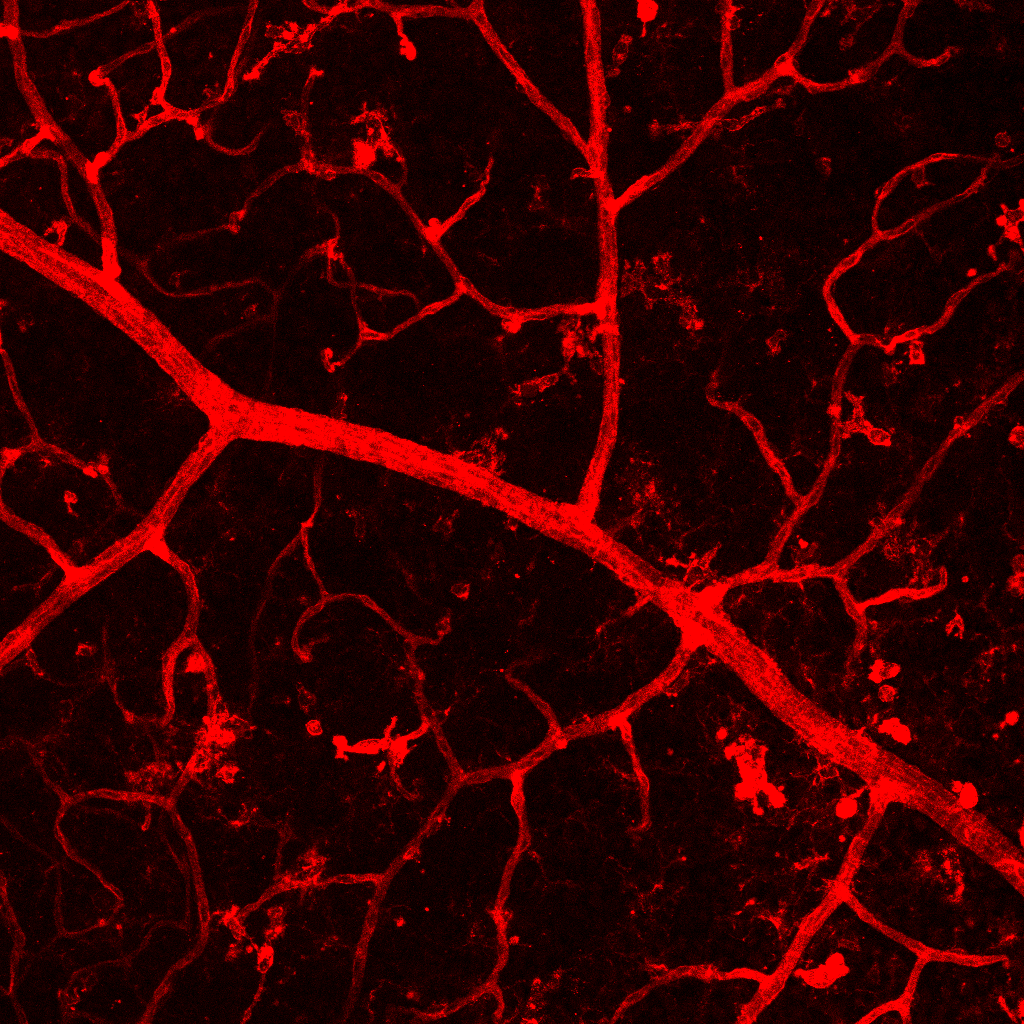

Supplement: Supplementary file 5 — Source Data Fig. 4 [file 44321_2024_25_MOESM5_ESM.zip › figure 4/4K/4K STZ+AAV-Fto lower line left.tif]

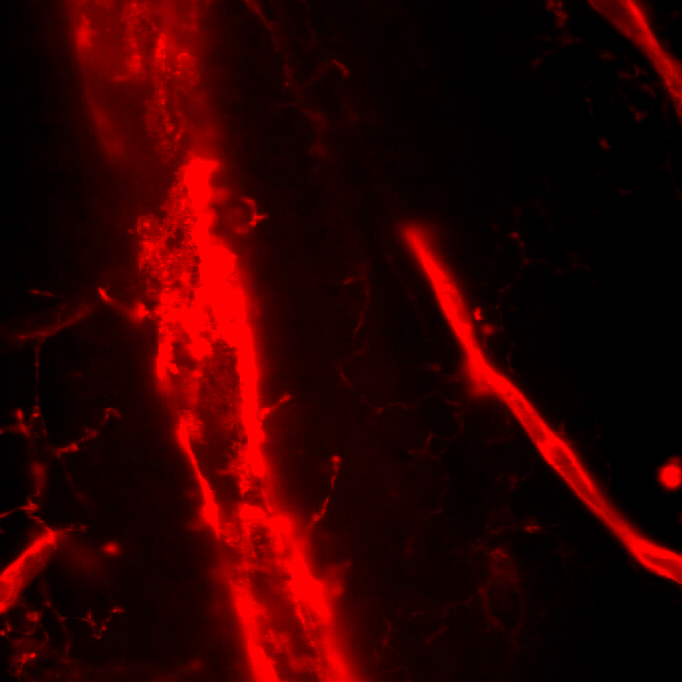

Supplement: Supplementary file 5 — Source Data Fig. 4 [file 44321_2024_25_MOESM5_ESM.zip › figure 4/4K/4K STZ+AAV-Fto lower line right.tif]

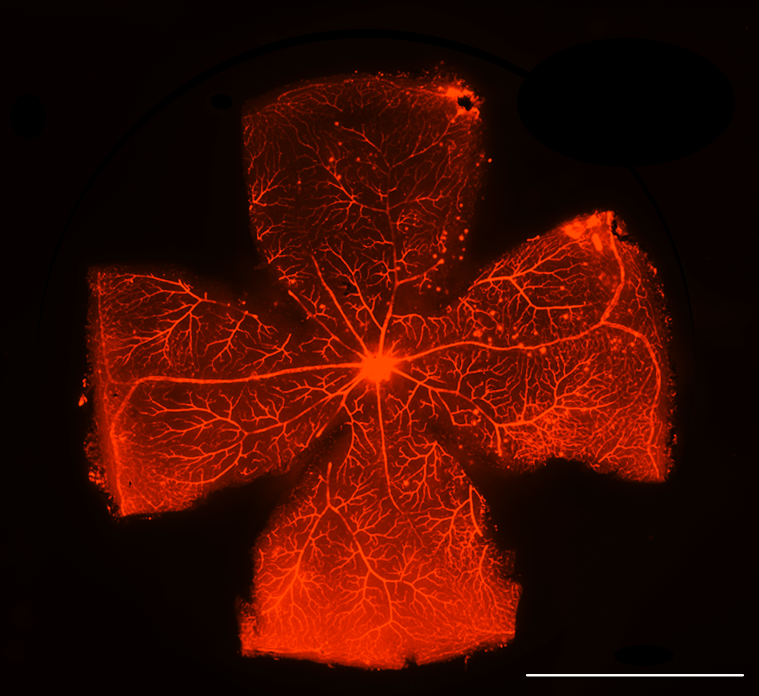

Supplement: Supplementary file 5 — Source Data Fig. 4 [file 44321_2024_25_MOESM5_ESM.zip › figure 4/4K/4K STZ+AAV-Fto upper line.tif]

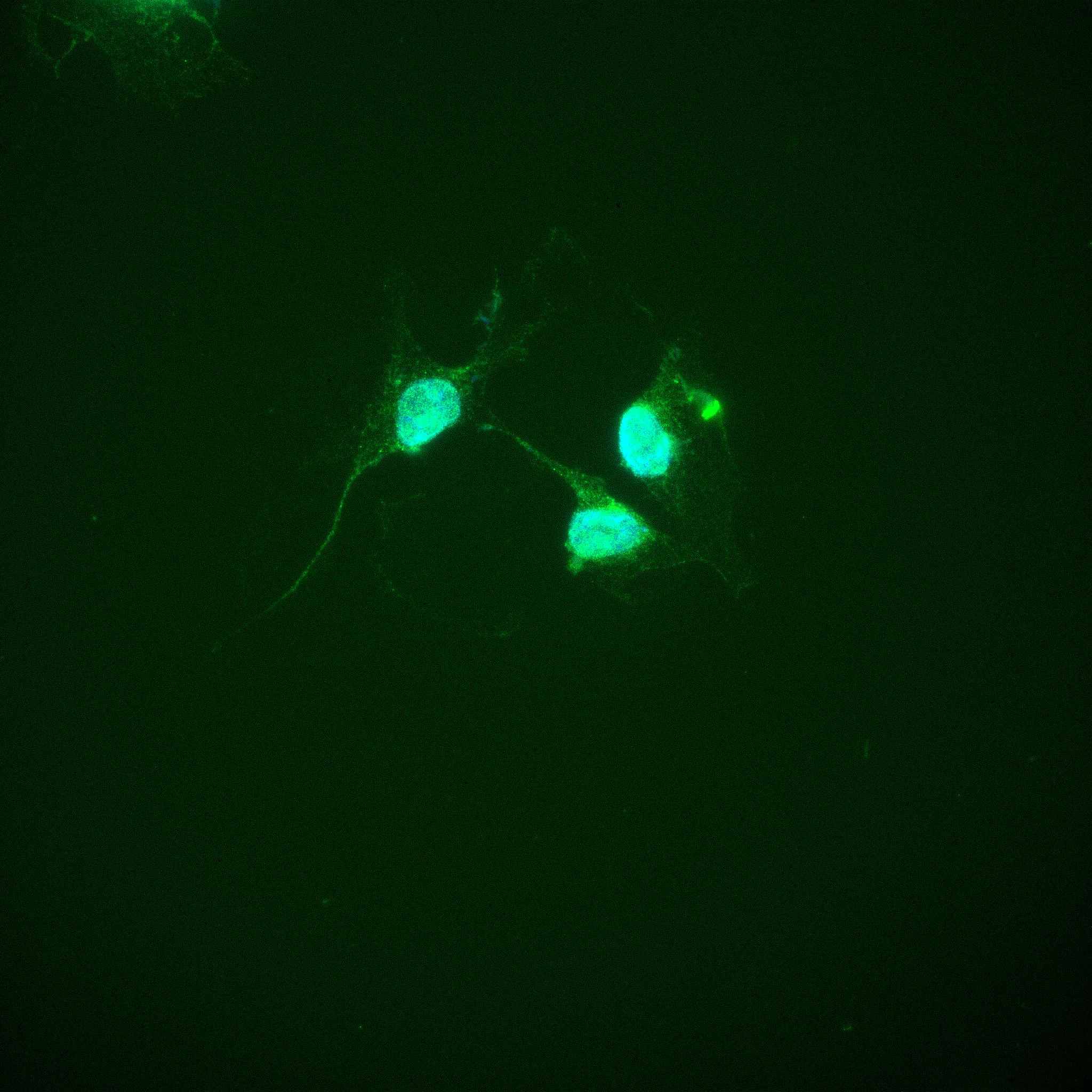

Supplement: Supplementary file 6 — Source Data Fig. 5 [file 44321_2024_25_MOESM6_ESM.zip › figure 5/5F/5F L-EV Iba1 DAPI.tif]

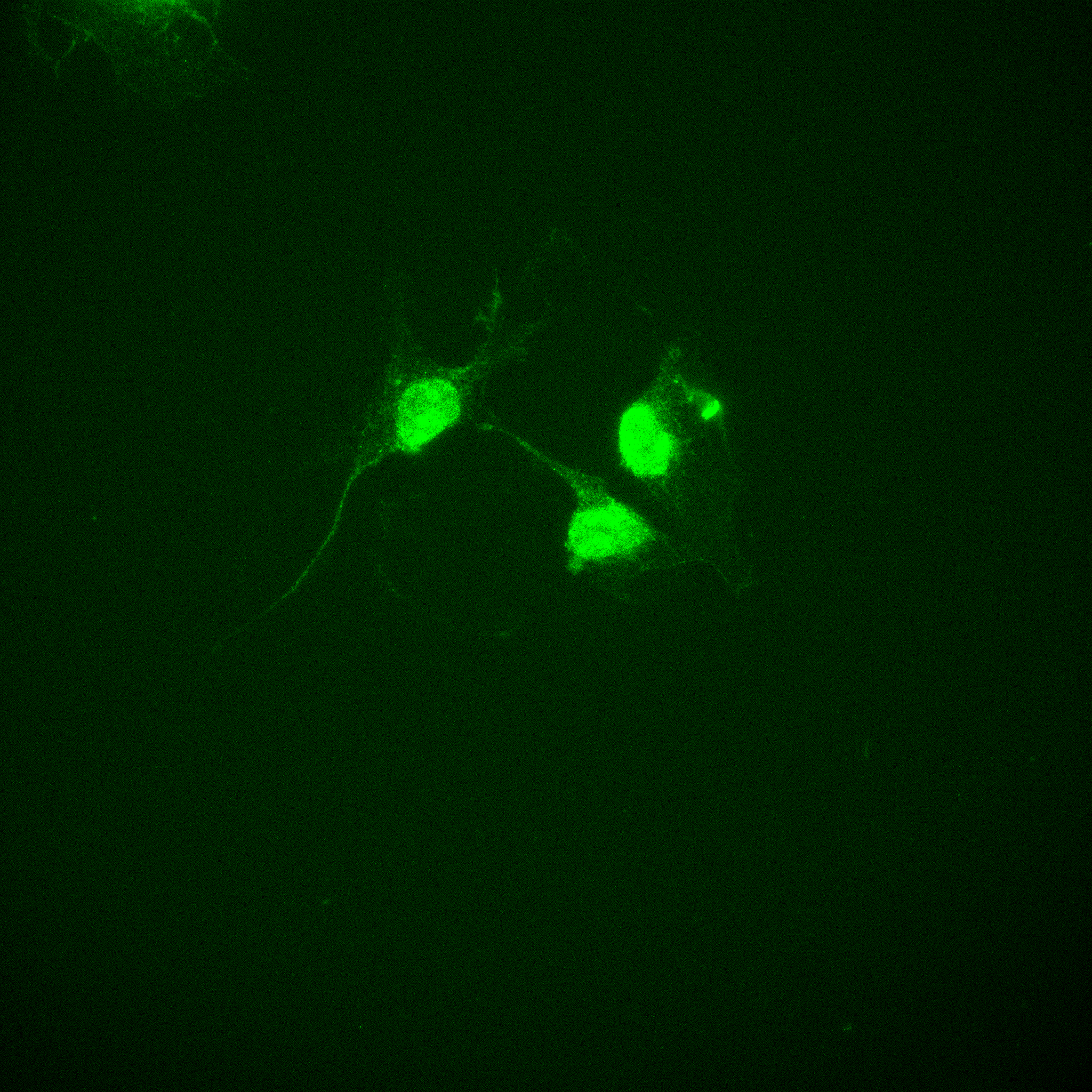

Supplement: Supplementary file 6 — Source Data Fig. 5 [file 44321_2024_25_MOESM6_ESM.zip › figure 5/5F/5F L-EV Iba1.tif]

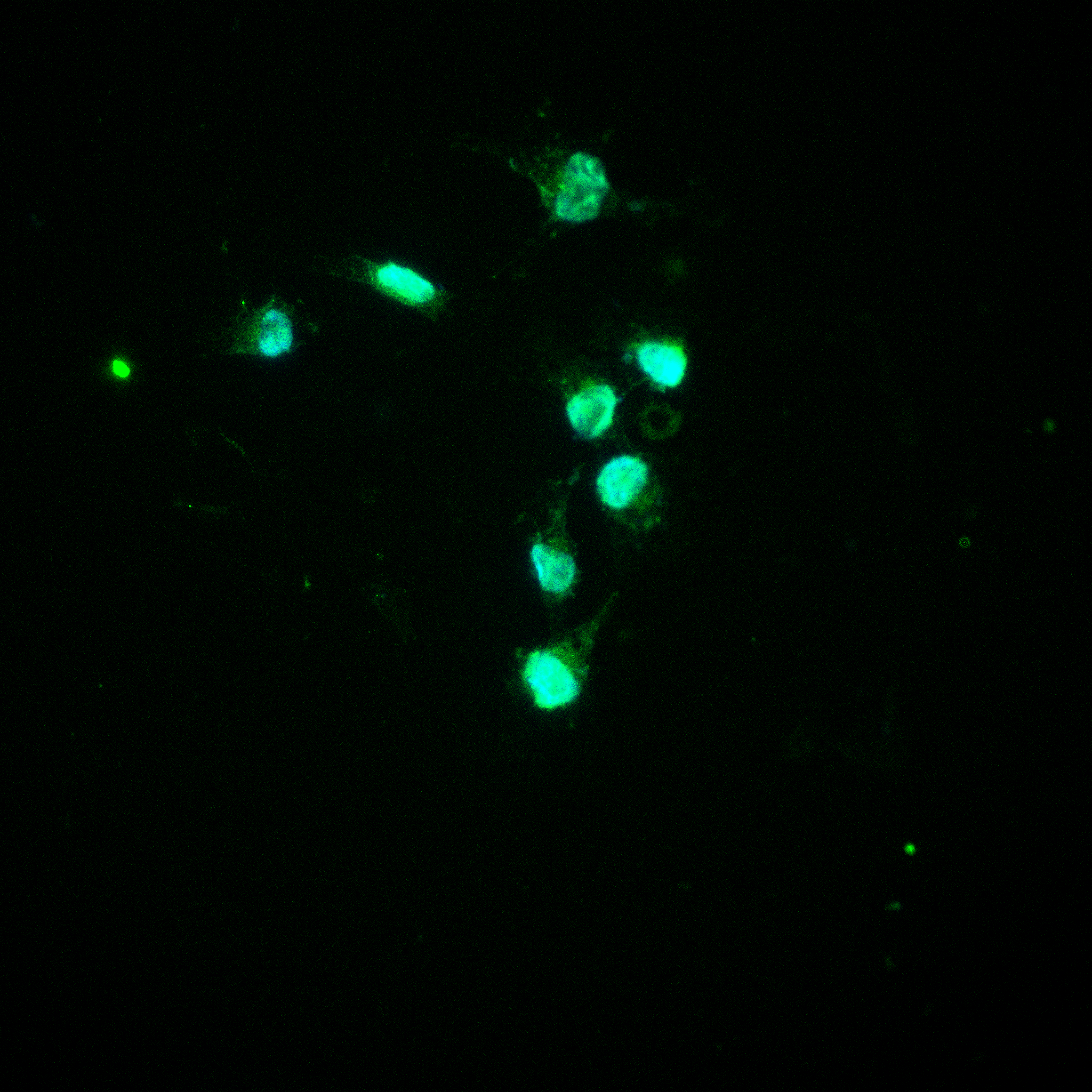

Supplement: Supplementary file 6 — Source Data Fig. 5 [file 44321_2024_25_MOESM6_ESM.zip › figure 5/5F/5F L-FTO Iba1 DAPI.tif]

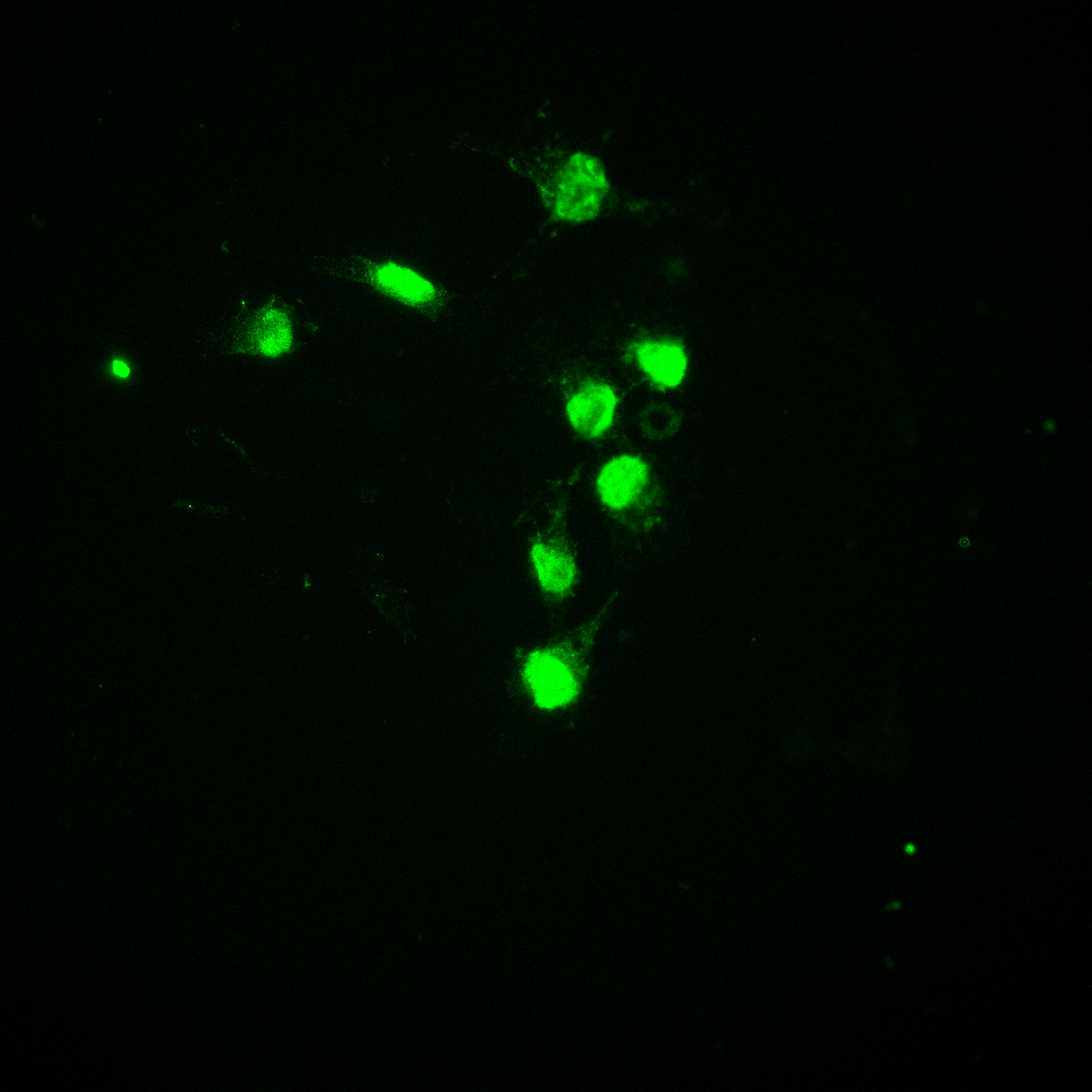

Supplement: Supplementary file 6 — Source Data Fig. 5 [file 44321_2024_25_MOESM6_ESM.zip › figure 5/5F/5F L-FTO Iba1.tif]

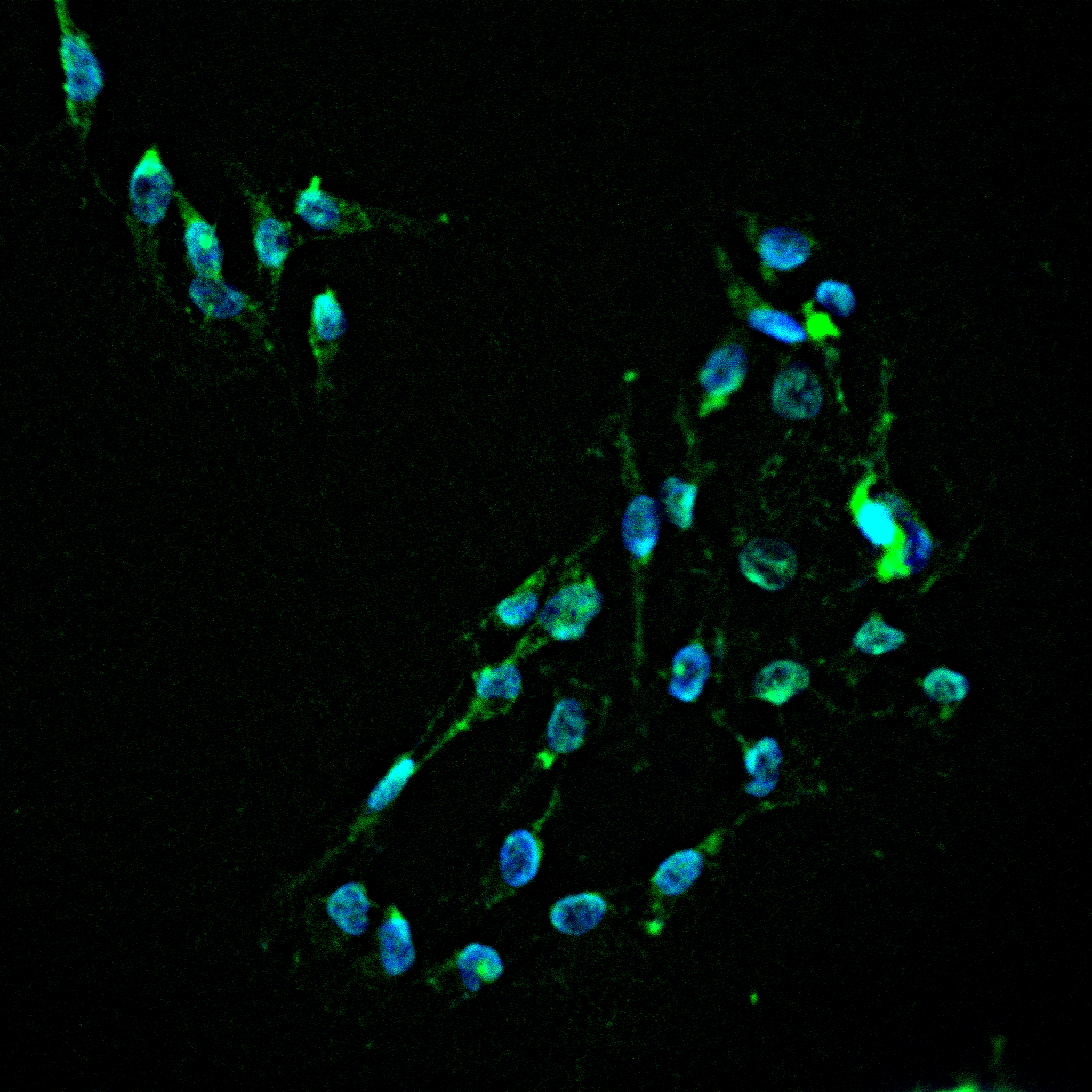

Supplement: Supplementary file 6 — Source Data Fig. 5 [file 44321_2024_25_MOESM6_ESM.zip › figure 5/5G/5G L-EV TMEM119 DAPI.tif]

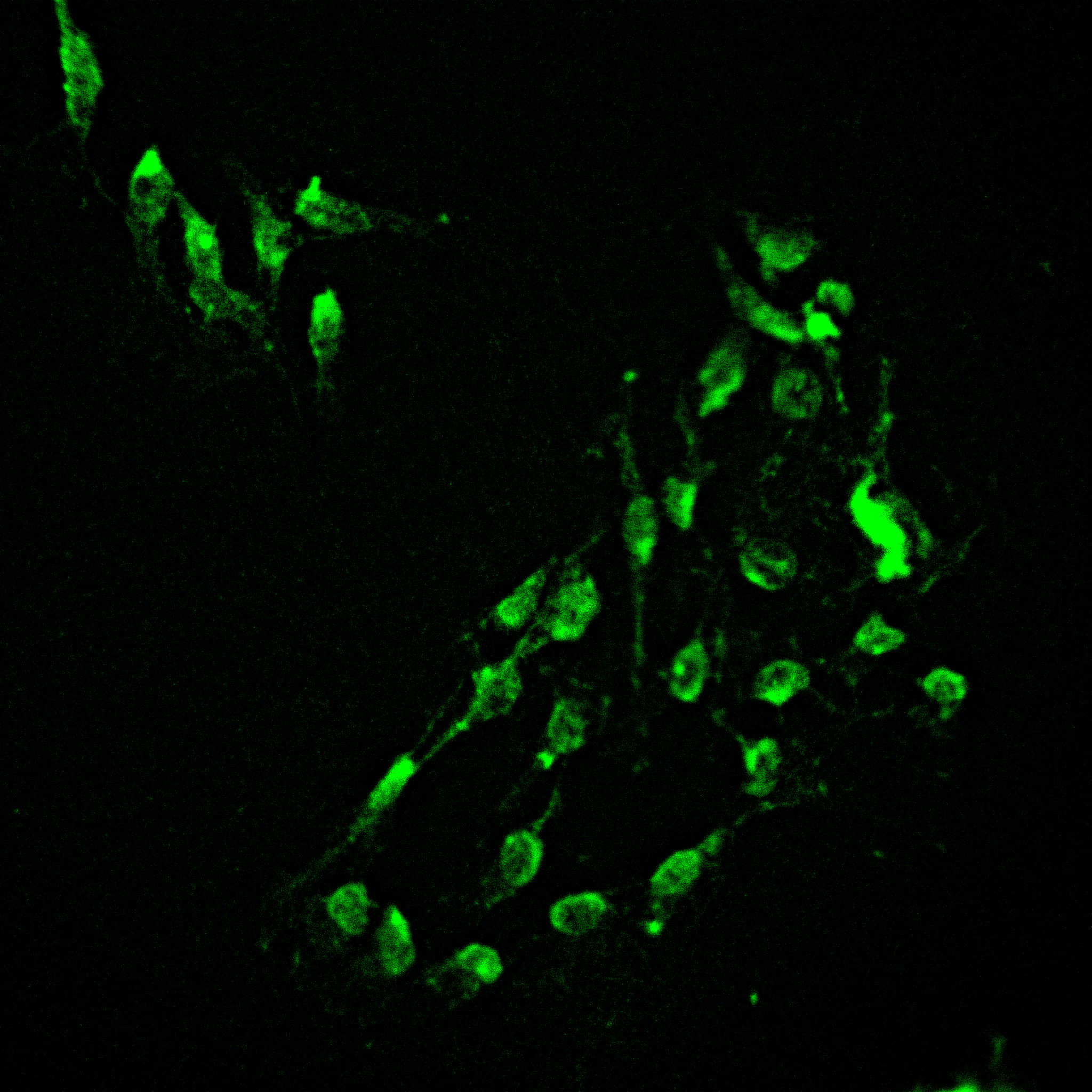

Supplement: Supplementary file 6 — Source Data Fig. 5 [file 44321_2024_25_MOESM6_ESM.zip › figure 5/5G/5G L-EV TMEM119.tif]

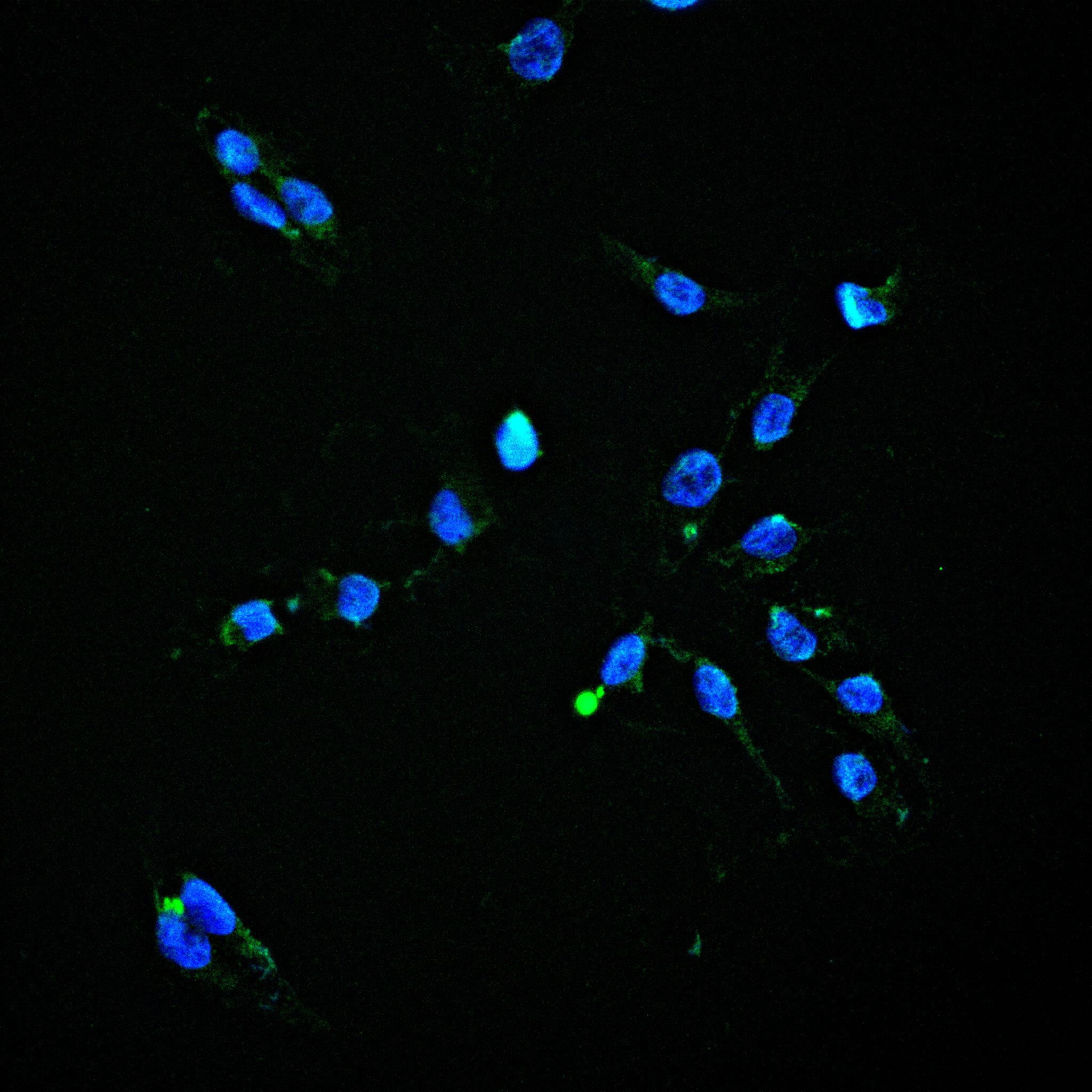

Supplement: Supplementary file 6 — Source Data Fig. 5 [file 44321_2024_25_MOESM6_ESM.zip › figure 5/5G/5G L-FTO TMEM119 DAPI.tif]

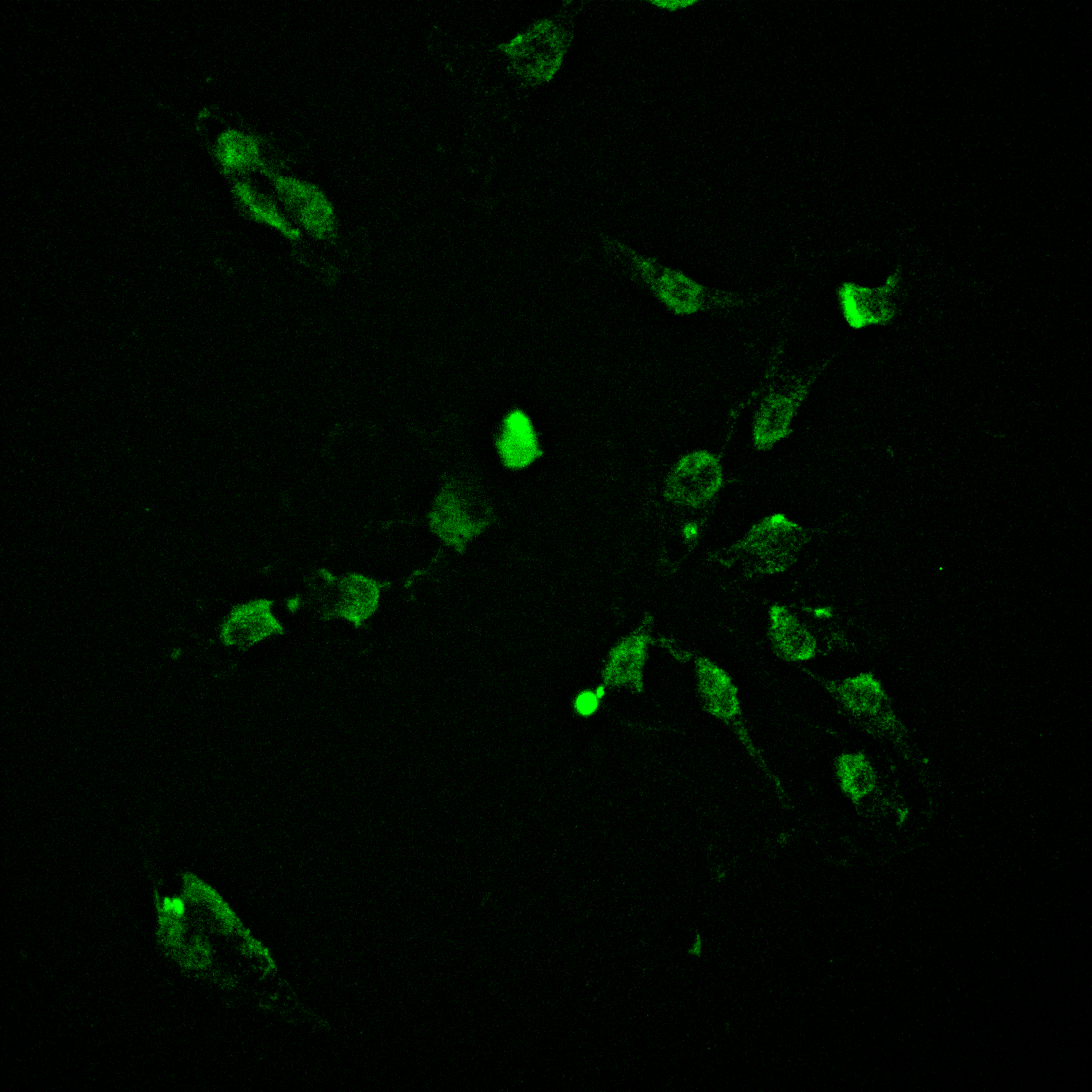

Supplement: Supplementary file 6 — Source Data Fig. 5 [file 44321_2024_25_MOESM6_ESM.zip › figure 5/5G/5G L-FTO TMEM119.tif]

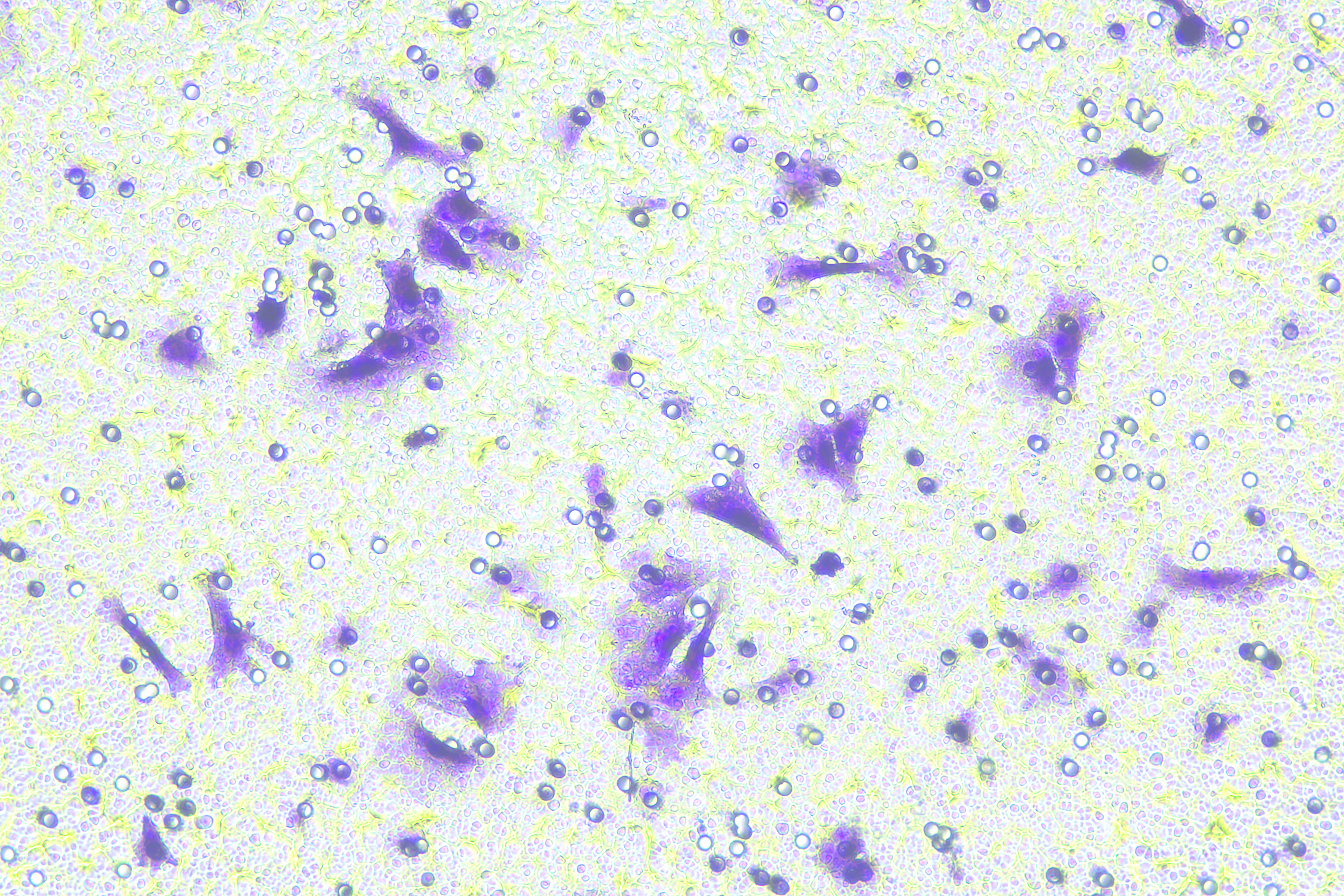

Supplement: Supplementary file 6 — Source Data Fig. 5 [file 44321_2024_25_MOESM6_ESM.zip › figure 5/5I/5I L-EV.tif]

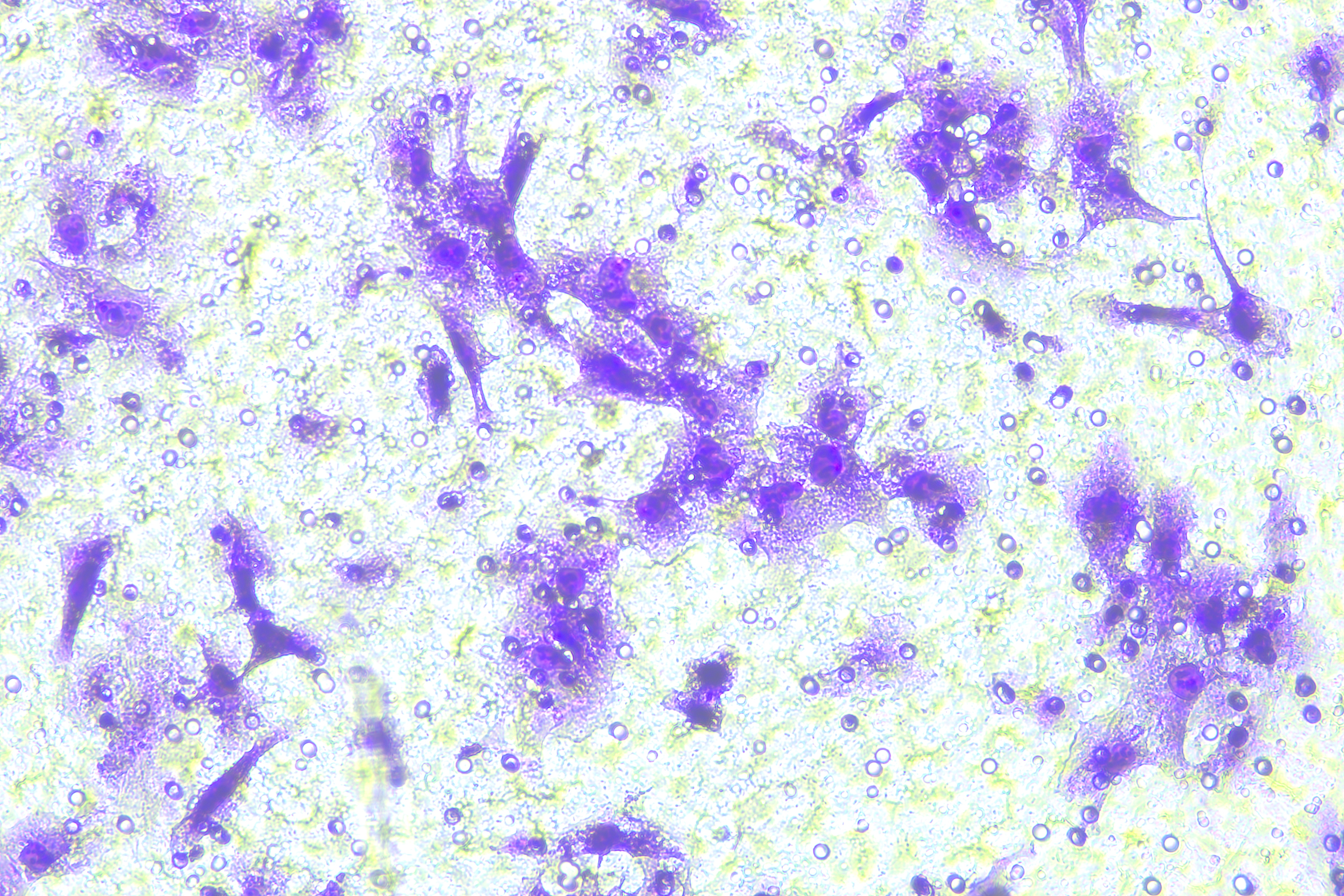

Supplement: Supplementary file 6 — Source Data Fig. 5 [file 44321_2024_25_MOESM6_ESM.zip › figure 5/5I/5I L-FTO.tif]

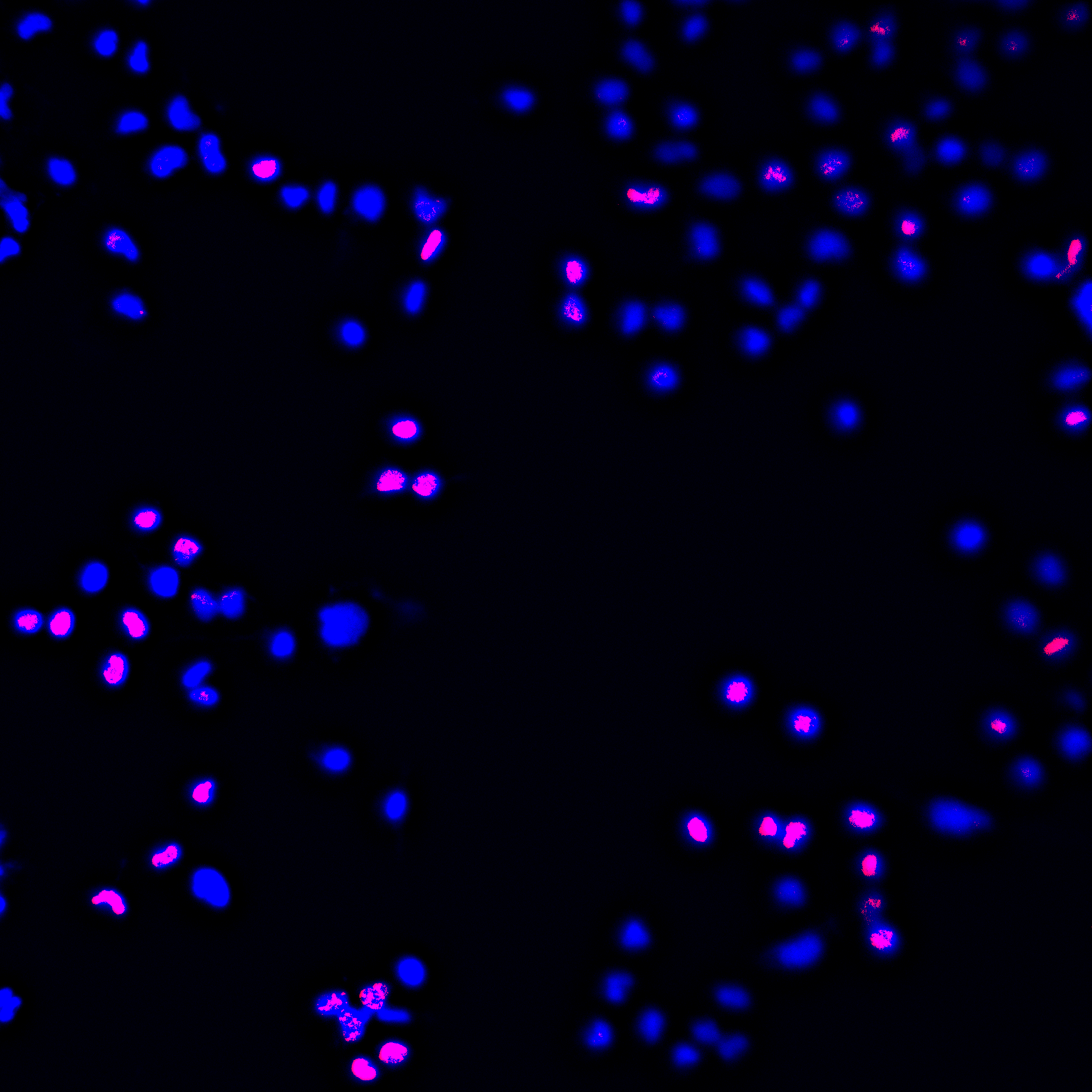

Supplement: Supplementary file 6 — Source Data Fig. 5 [file 44321_2024_25_MOESM6_ESM.zip › figure 5/5J/5J L-EV EdU DAPI.tif]

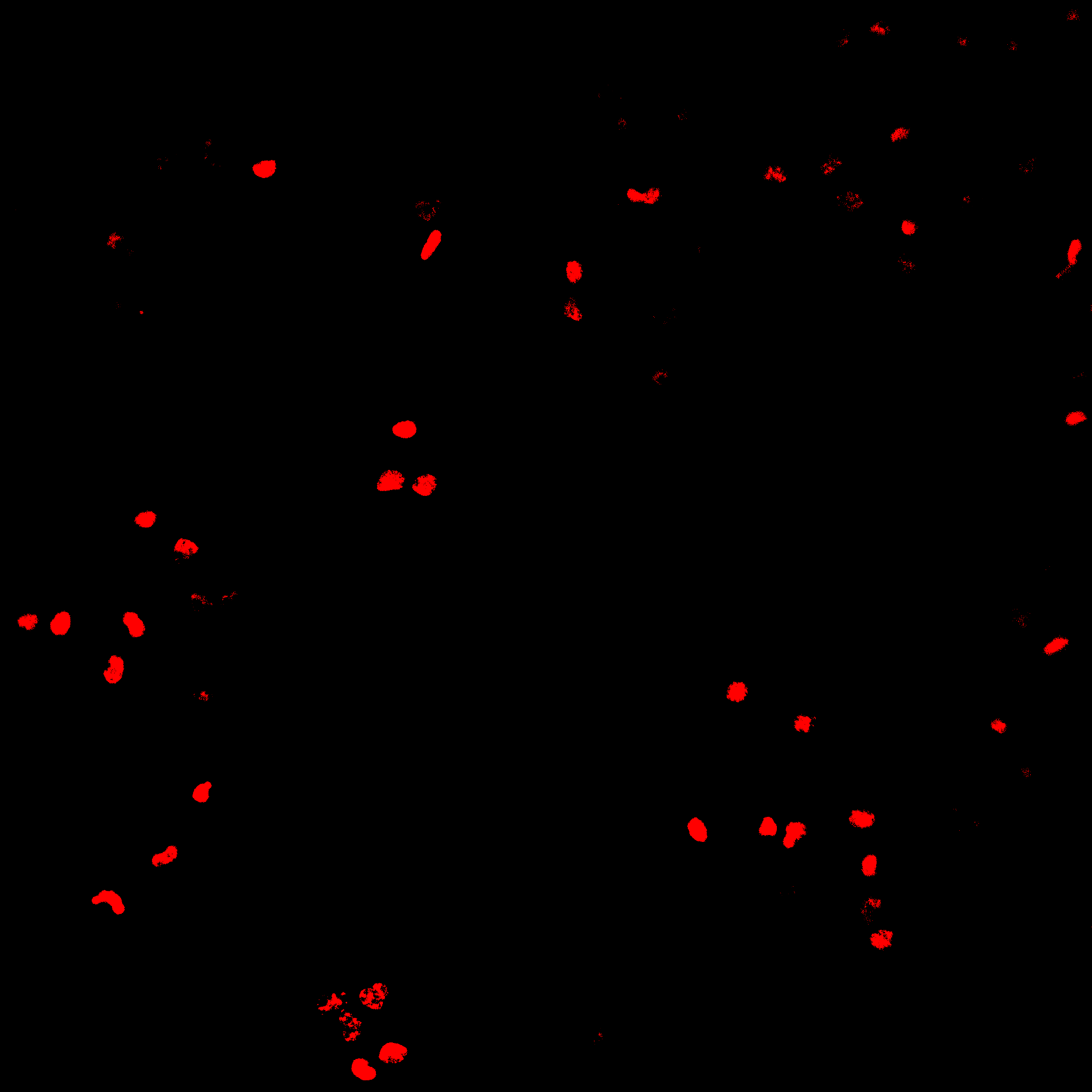

Supplement: Supplementary file 6 — Source Data Fig. 5 [file 44321_2024_25_MOESM6_ESM.zip › figure 5/5J/5J L-EV EdU.tif]

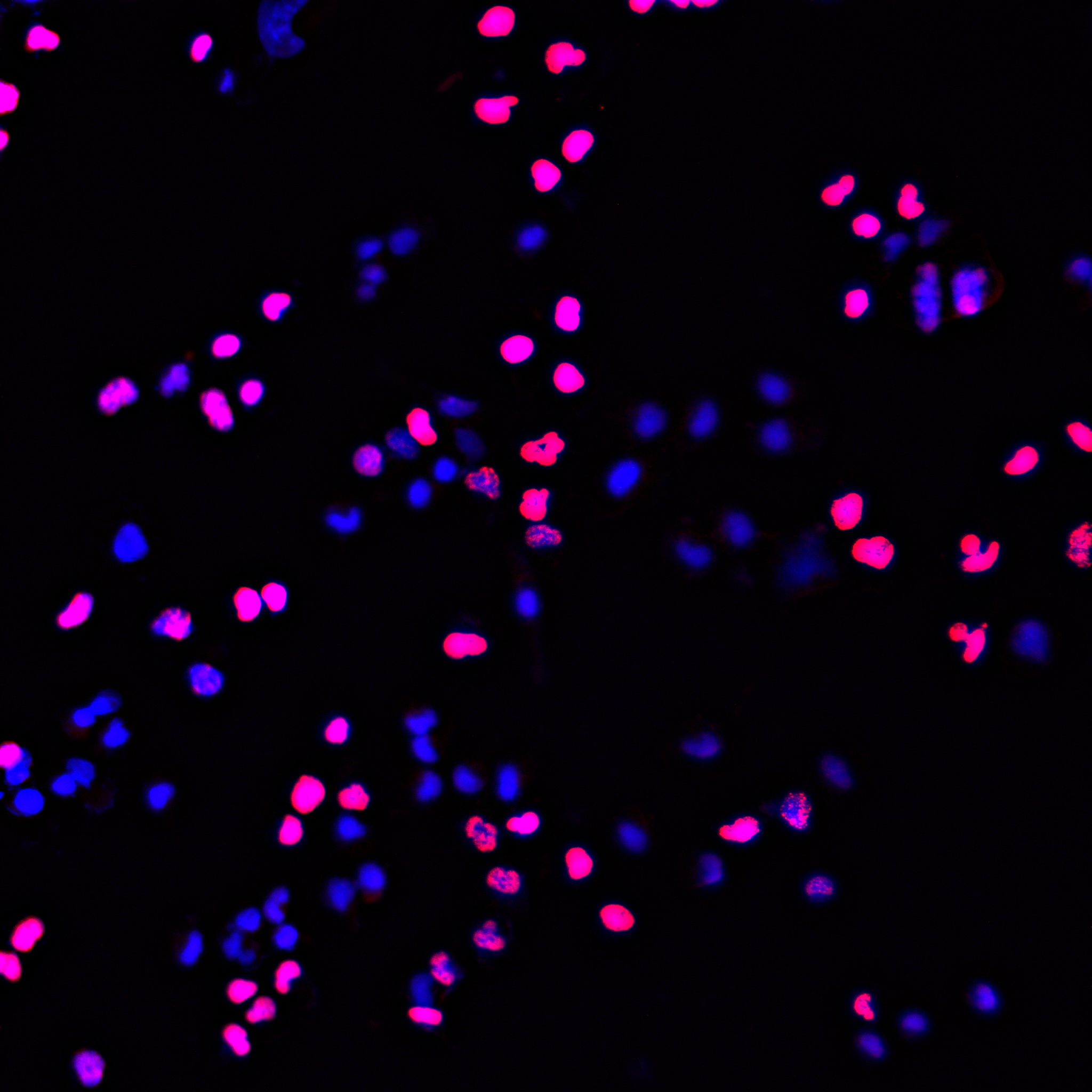

Supplement: Supplementary file 6 — Source Data Fig. 5 [file 44321_2024_25_MOESM6_ESM.zip › figure 5/5J/5J L-FTO EdU DAPI.tif]

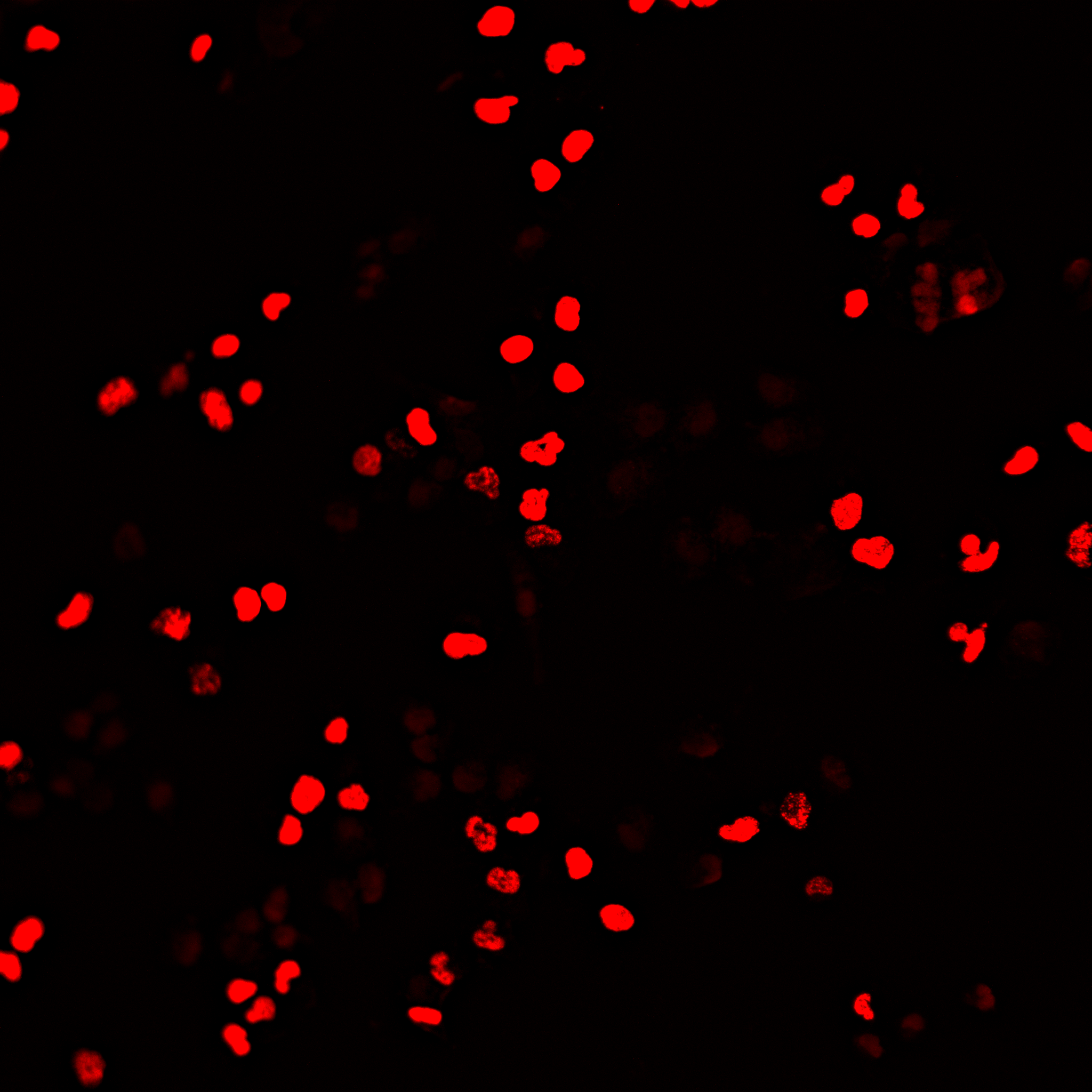

Supplement: Supplementary file 6 — Source Data Fig. 5 [file 44321_2024_25_MOESM6_ESM.zip › figure 5/5J/5J L-FTO EdU.tif]

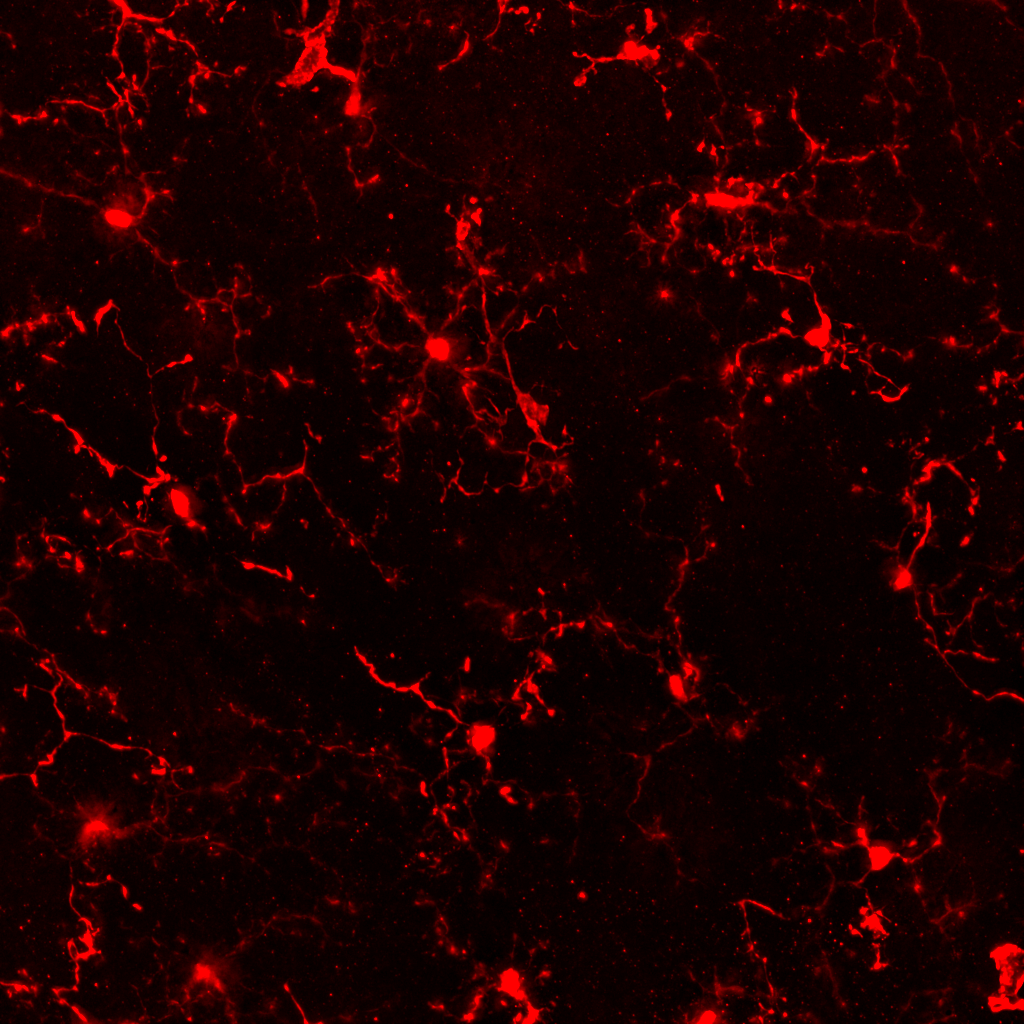

Supplement: Supplementary file 7 — Source Data Fig. 6 [file 44321_2024_25_MOESM7_ESM.zip › figure 6/6B/6B Ctrl lower line.tif]

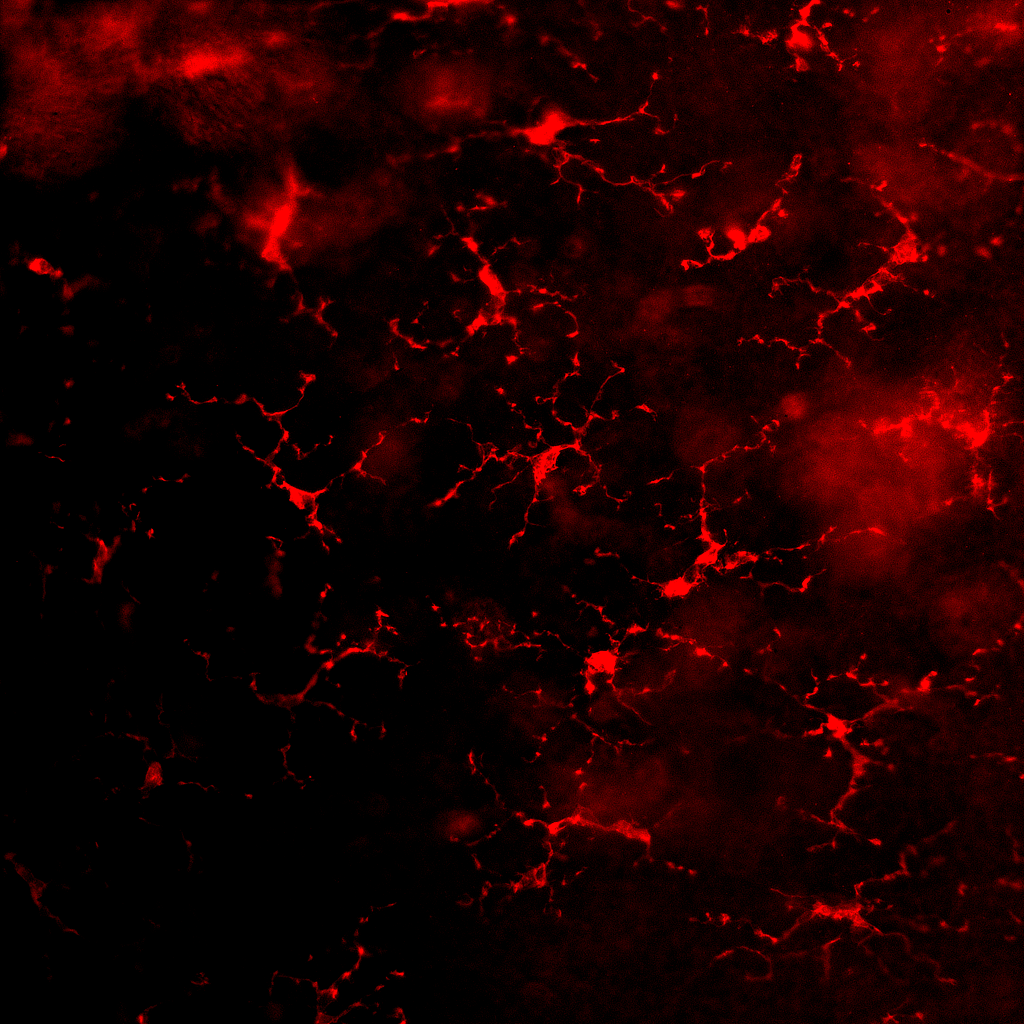

Supplement: Supplementary file 7 — Source Data Fig. 6 [file 44321_2024_25_MOESM7_ESM.zip › figure 6/6B/6B STZ lower line.tif]

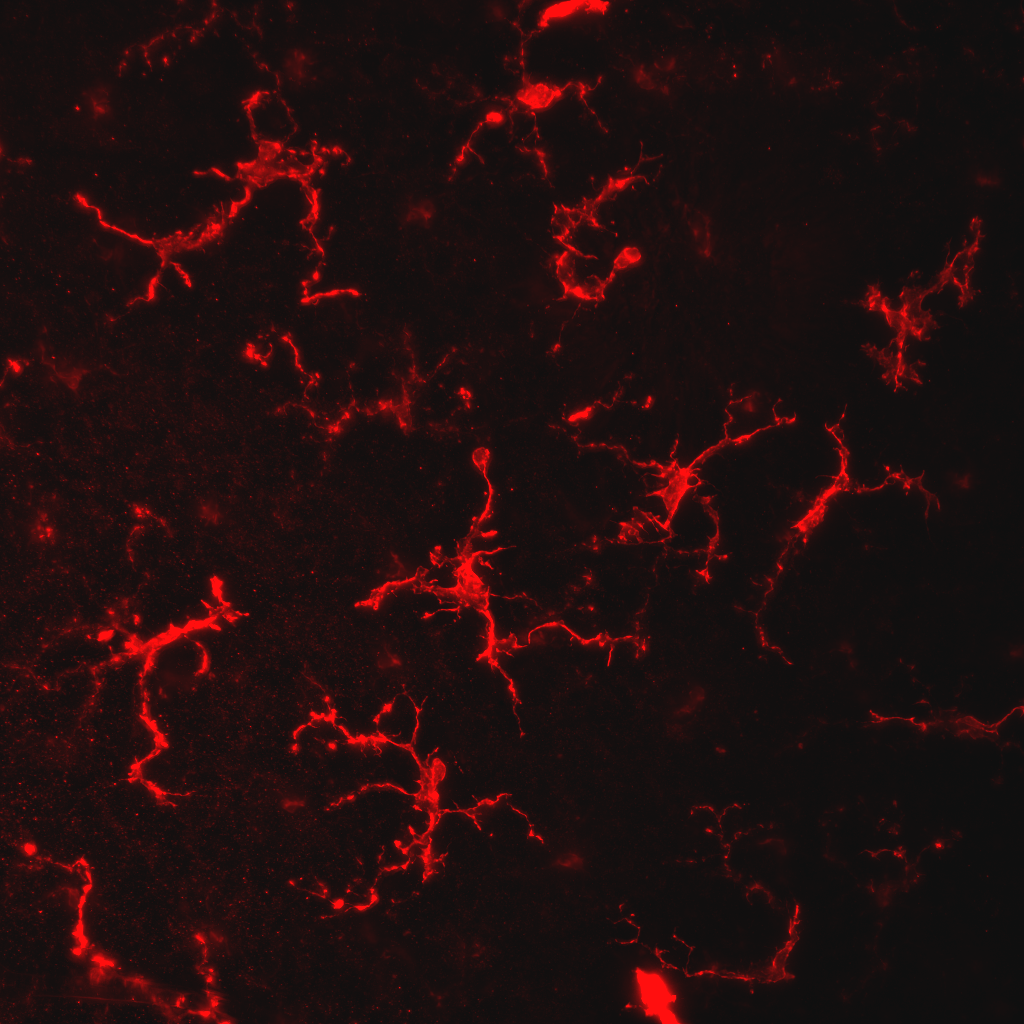

Supplement: Supplementary file 7 — Source Data Fig. 6 [file 44321_2024_25_MOESM7_ESM.zip › figure 6/6B/6B STZ+AAV-blank lower line.tif]

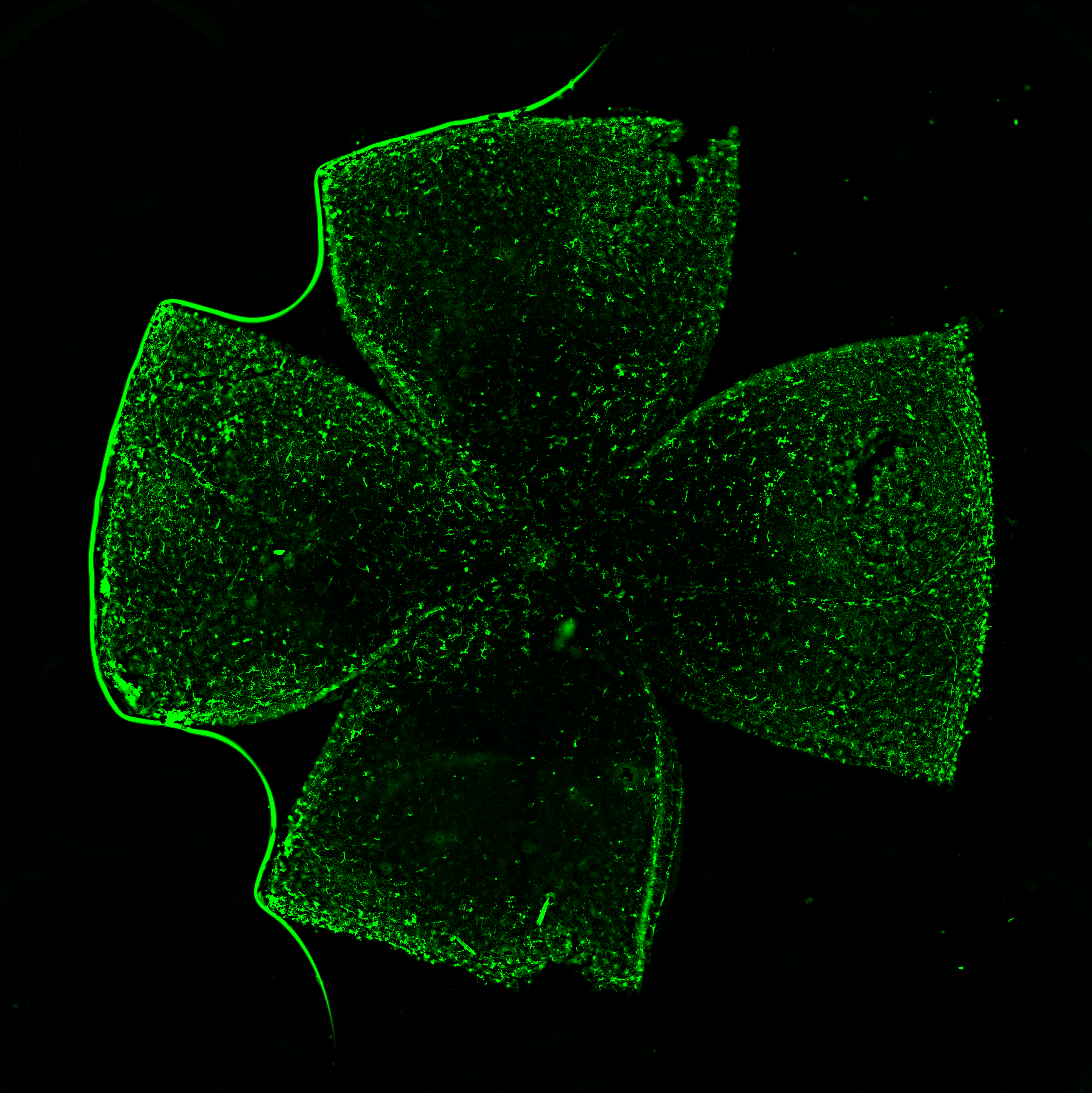

Supplement: Supplementary file 7 — Source Data Fig. 6 [file 44321_2024_25_MOESM7_ESM.zip › figure 6/6B/6B STZ+AAV-blank upper line.tif]

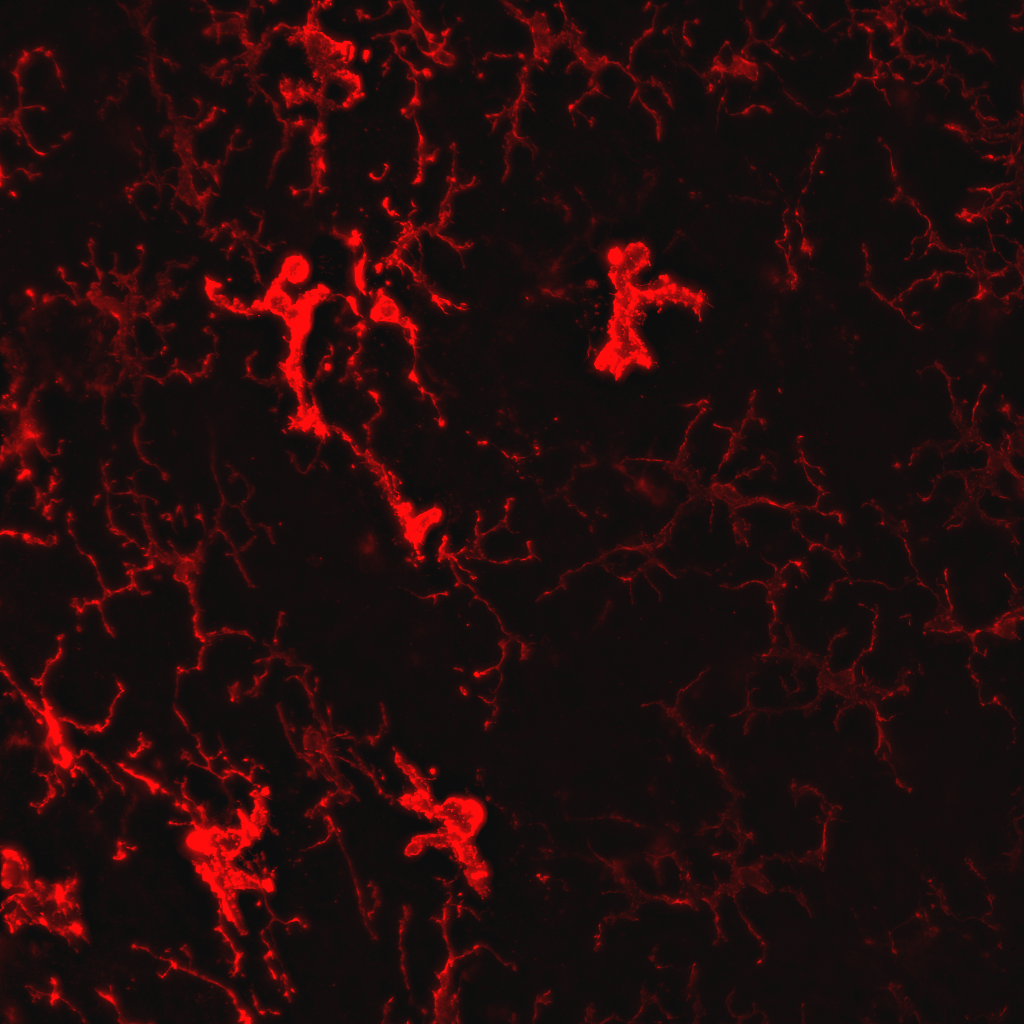

Supplement: Supplementary file 7 — Source Data Fig. 6 [file 44321_2024_25_MOESM7_ESM.zip › figure 6/6B/6B STZ+AAV-Fto lower line.tif]

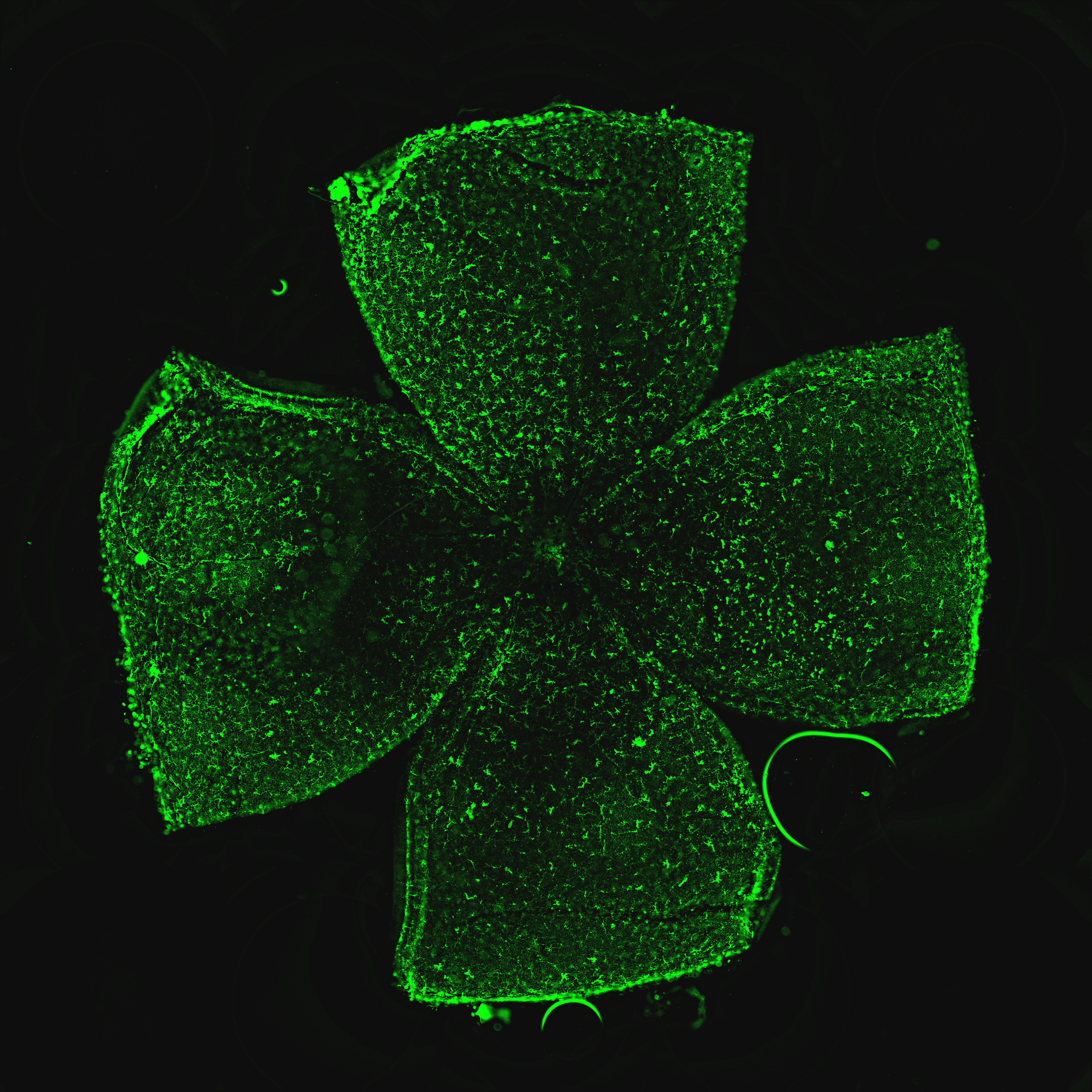

Supplement: Supplementary file 7 — Source Data Fig. 6 [file 44321_2024_25_MOESM7_ESM.zip › figure 6/6B/6B STZ+AAV-Fto upper line.tif]

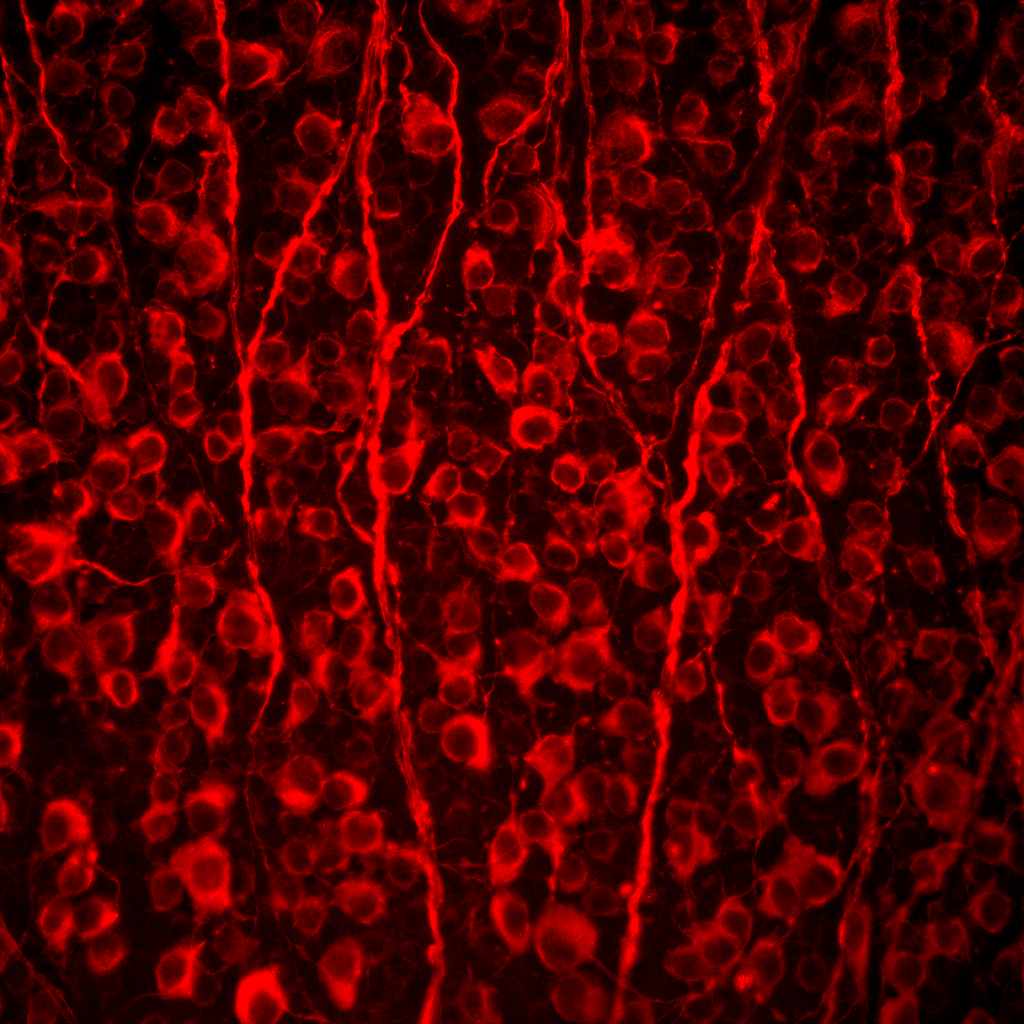

Supplement: Supplementary file 7 — Source Data Fig. 6 [file 44321_2024_25_MOESM7_ESM.zip › figure 6/6K/6K Ctrl.tif]

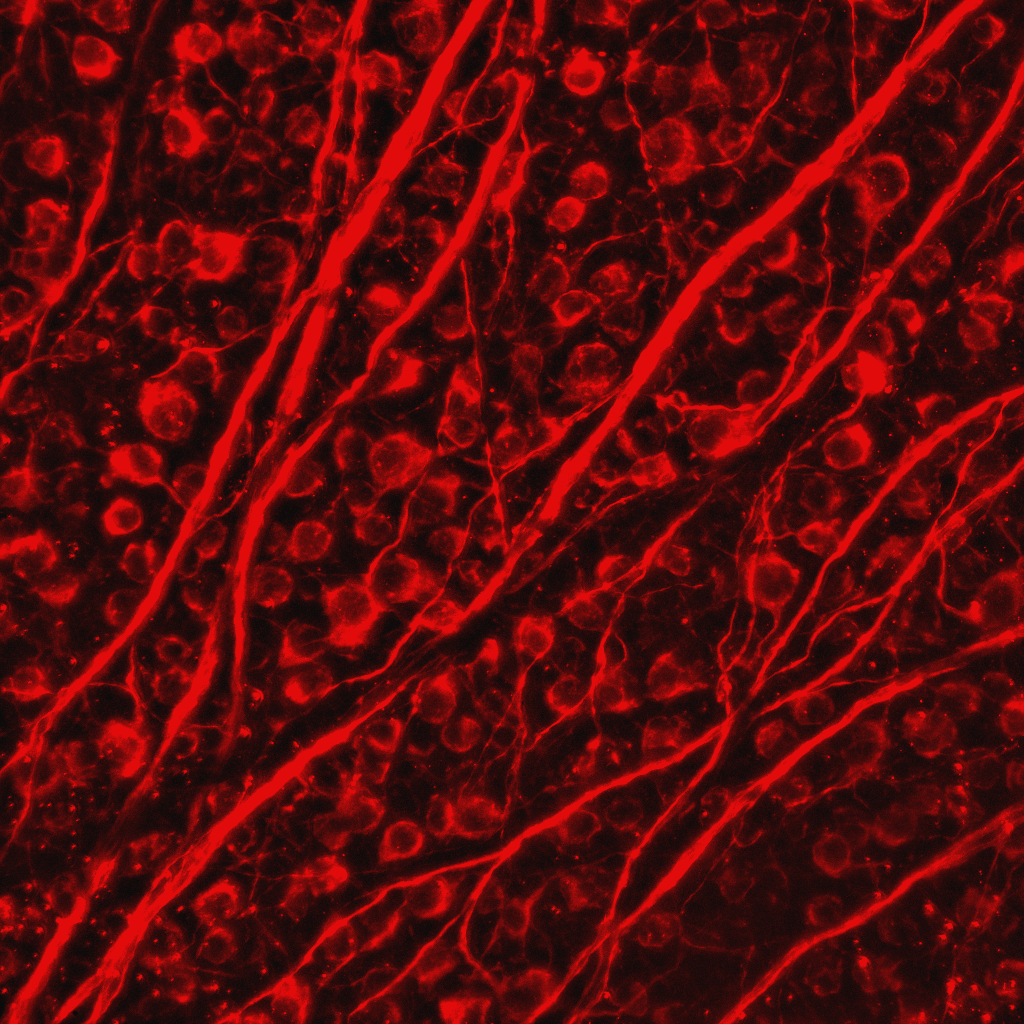

Supplement: Supplementary file 7 — Source Data Fig. 6 [file 44321_2024_25_MOESM7_ESM.zip › figure 6/6K/6K STZ.tif]

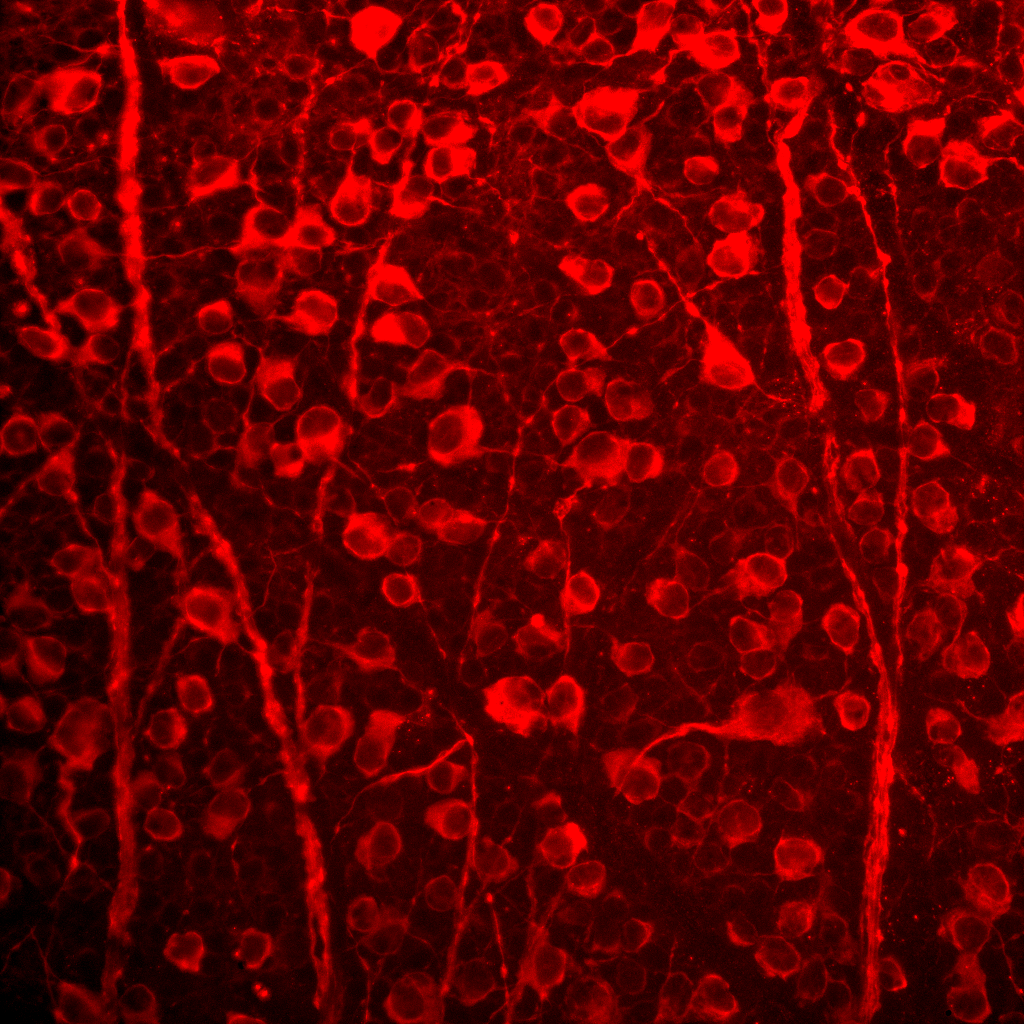

Supplement: Supplementary file 7 — Source Data Fig. 6 [file 44321_2024_25_MOESM7_ESM.zip › figure 6/6K/6K STZ+AAV-blank.tif]

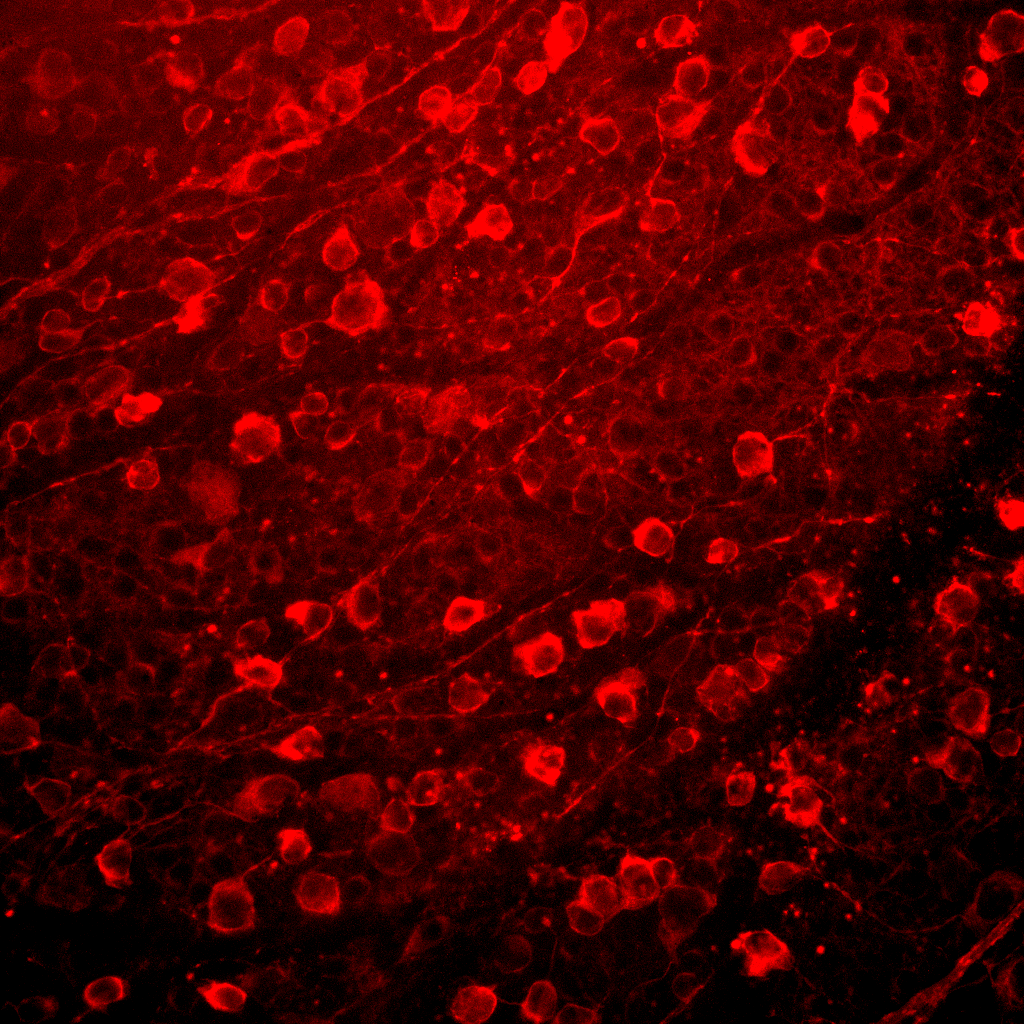

Supplement: Supplementary file 7 — Source Data Fig. 6 [file 44321_2024_25_MOESM7_ESM.zip › figure 6/6K/6K STZ+AAV-Fto.tif]

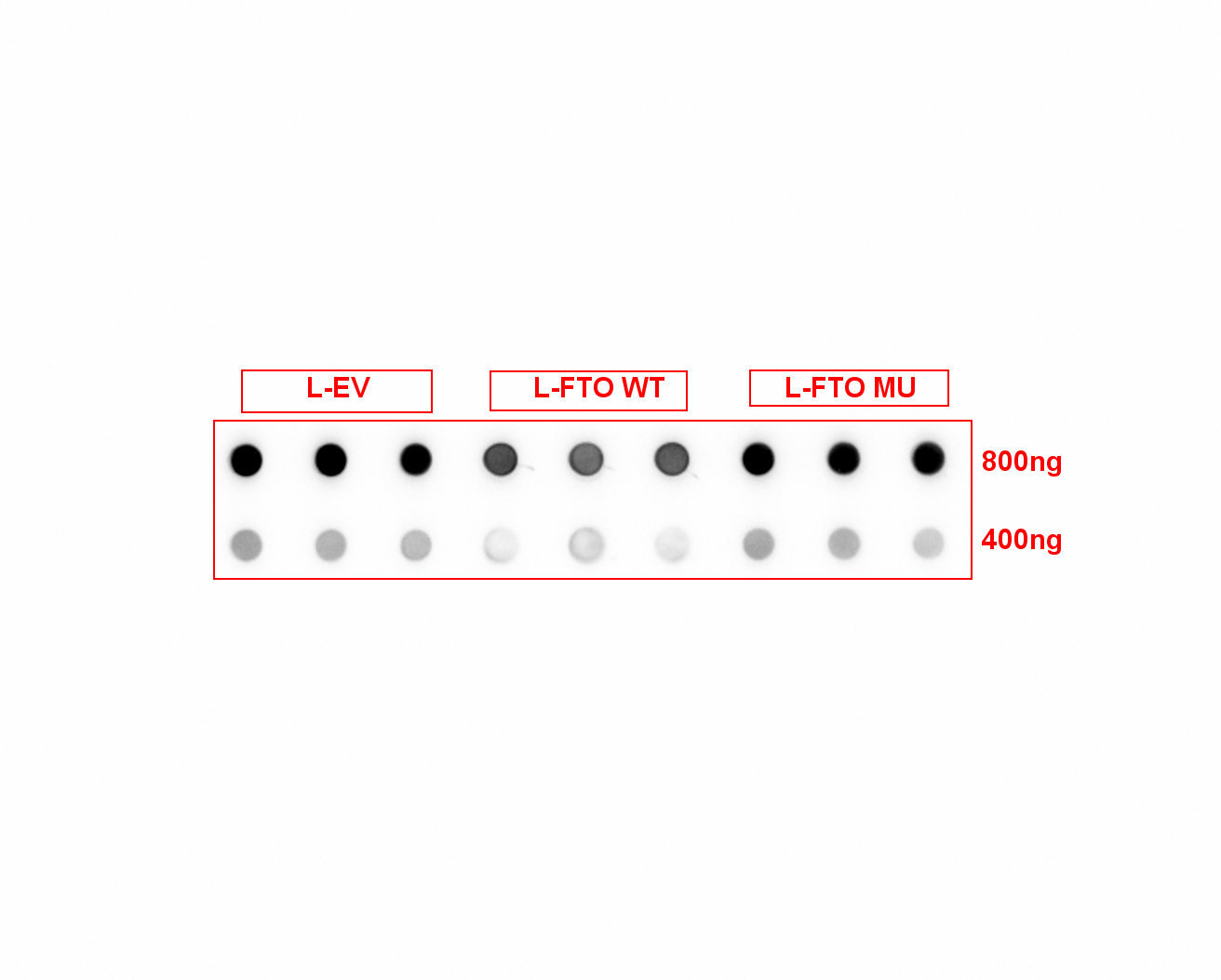

Supplement: Supplementary file 8 — Source Data Fig. 7 [file 44321_2024_25_MOESM8_ESM.zip › figure 7/7A/7A m6A mark.Tif]

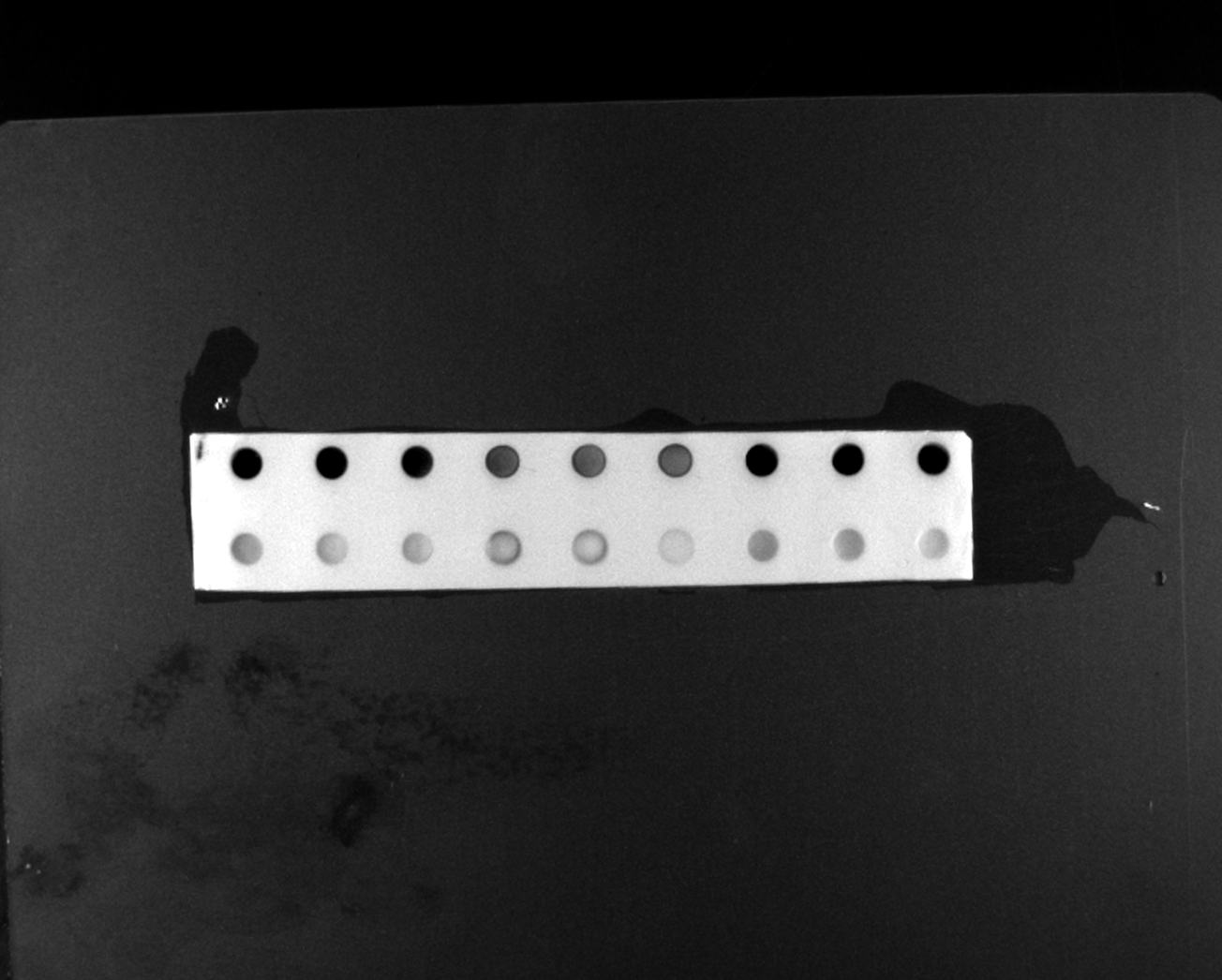

Supplement: Supplementary file 8 — Source Data Fig. 7 [file 44321_2024_25_MOESM8_ESM.zip › figure 7/7A/7A m6A.Tif]

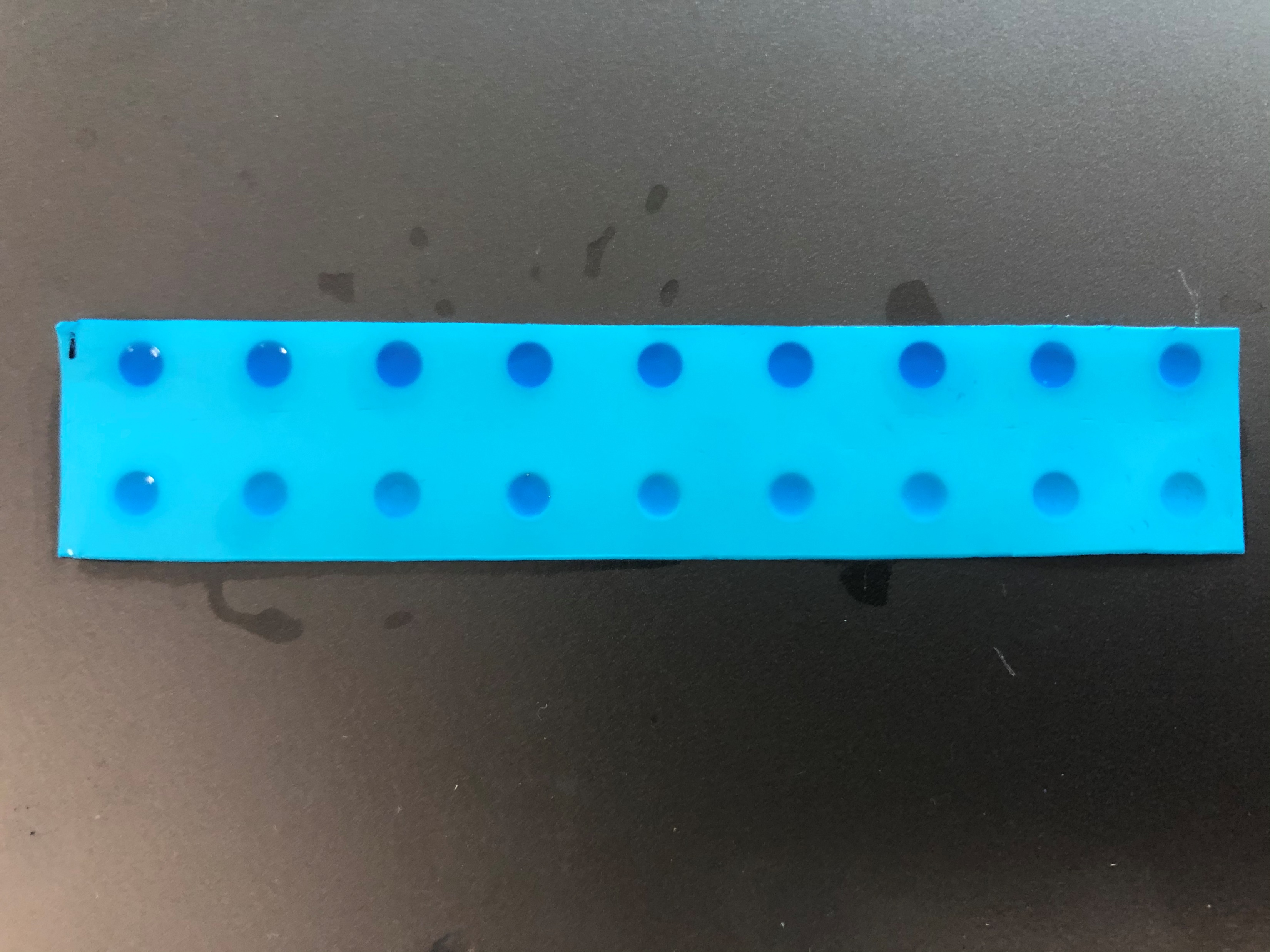

Supplement: Supplementary file 8 — Source Data Fig. 7 [file 44321_2024_25_MOESM8_ESM.zip › figure 7/7A/7A MB.tif]

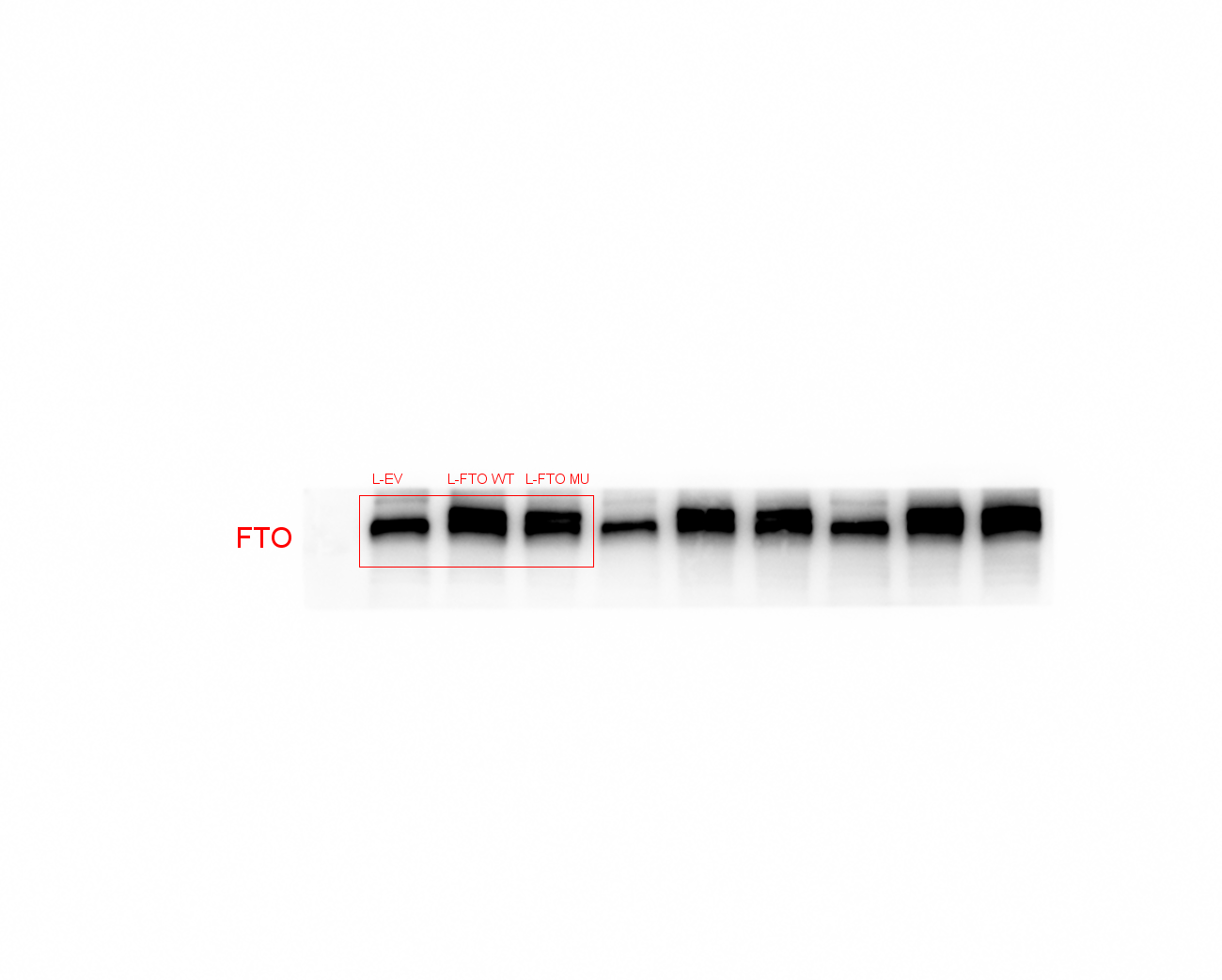

Supplement: Supplementary file 8 — Source Data Fig. 7 [file 44321_2024_25_MOESM8_ESM.zip › figure 7/7B/7B FTO mark.Tif]

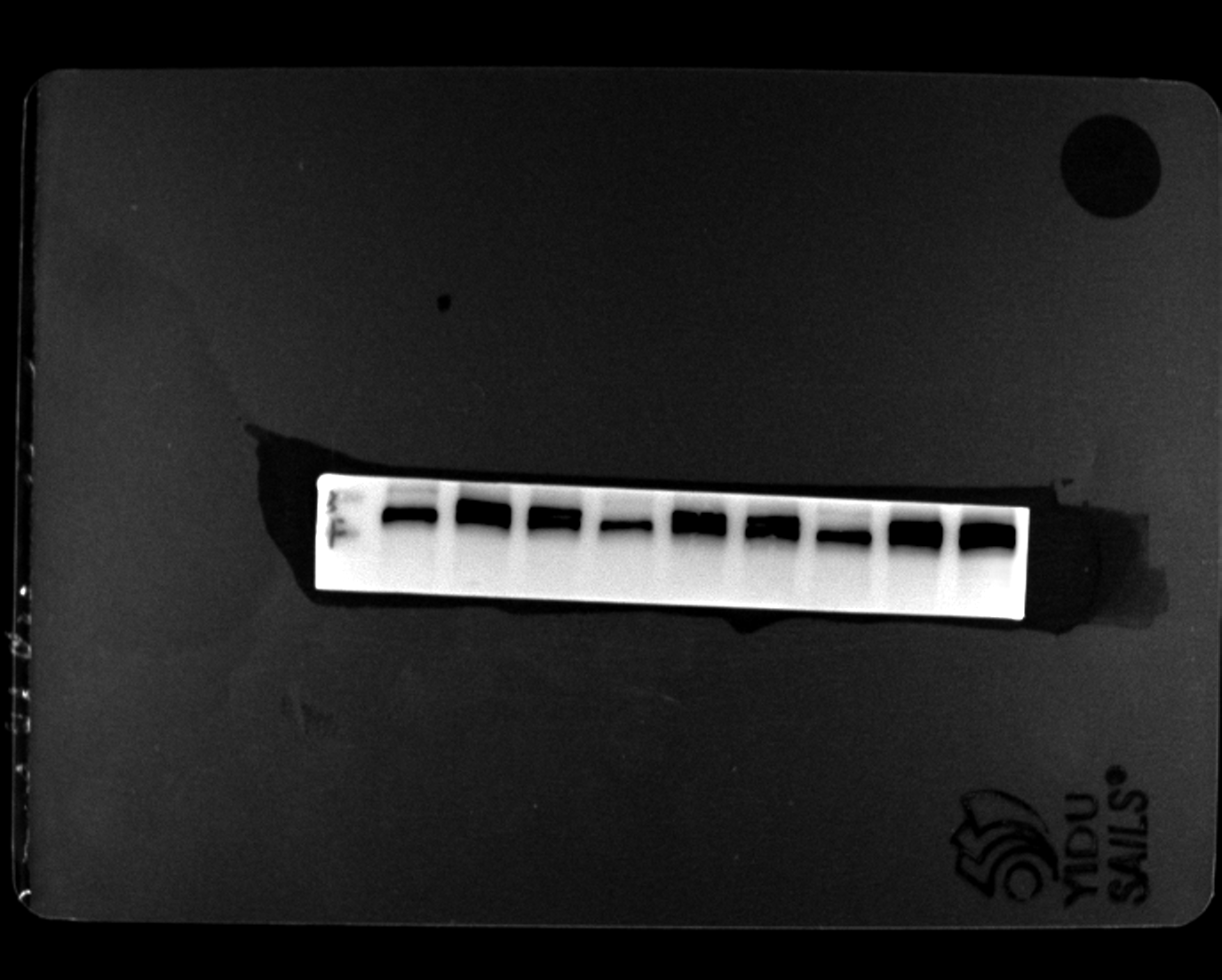

Supplement: Supplementary file 8 — Source Data Fig. 7 [file 44321_2024_25_MOESM8_ESM.zip › figure 7/7B/7B FTO.Tif]

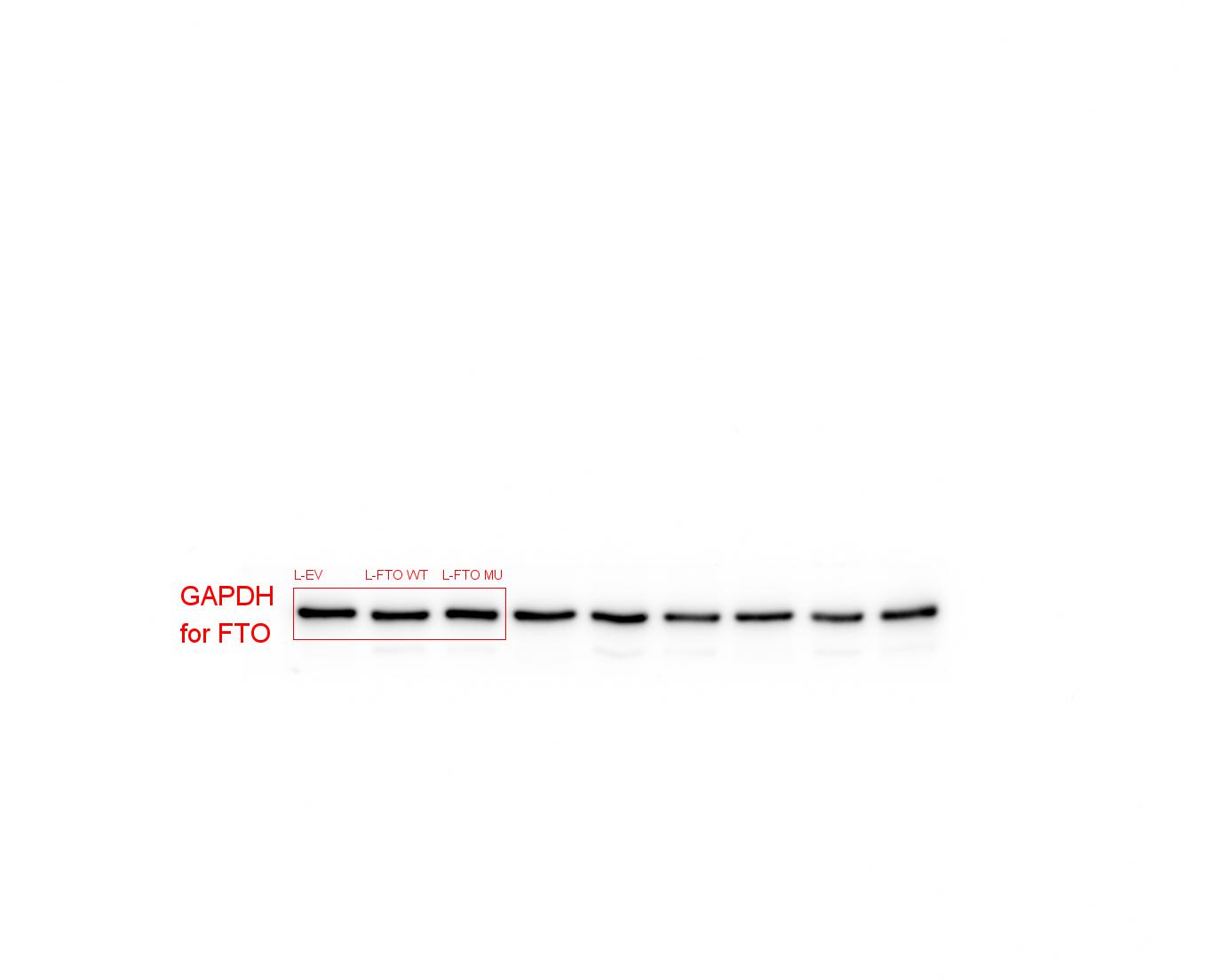

Supplement: Supplementary file 8 — Source Data Fig. 7 [file 44321_2024_25_MOESM8_ESM.zip › figure 7/7B/7B GAPDH for FTO mark.Tif]

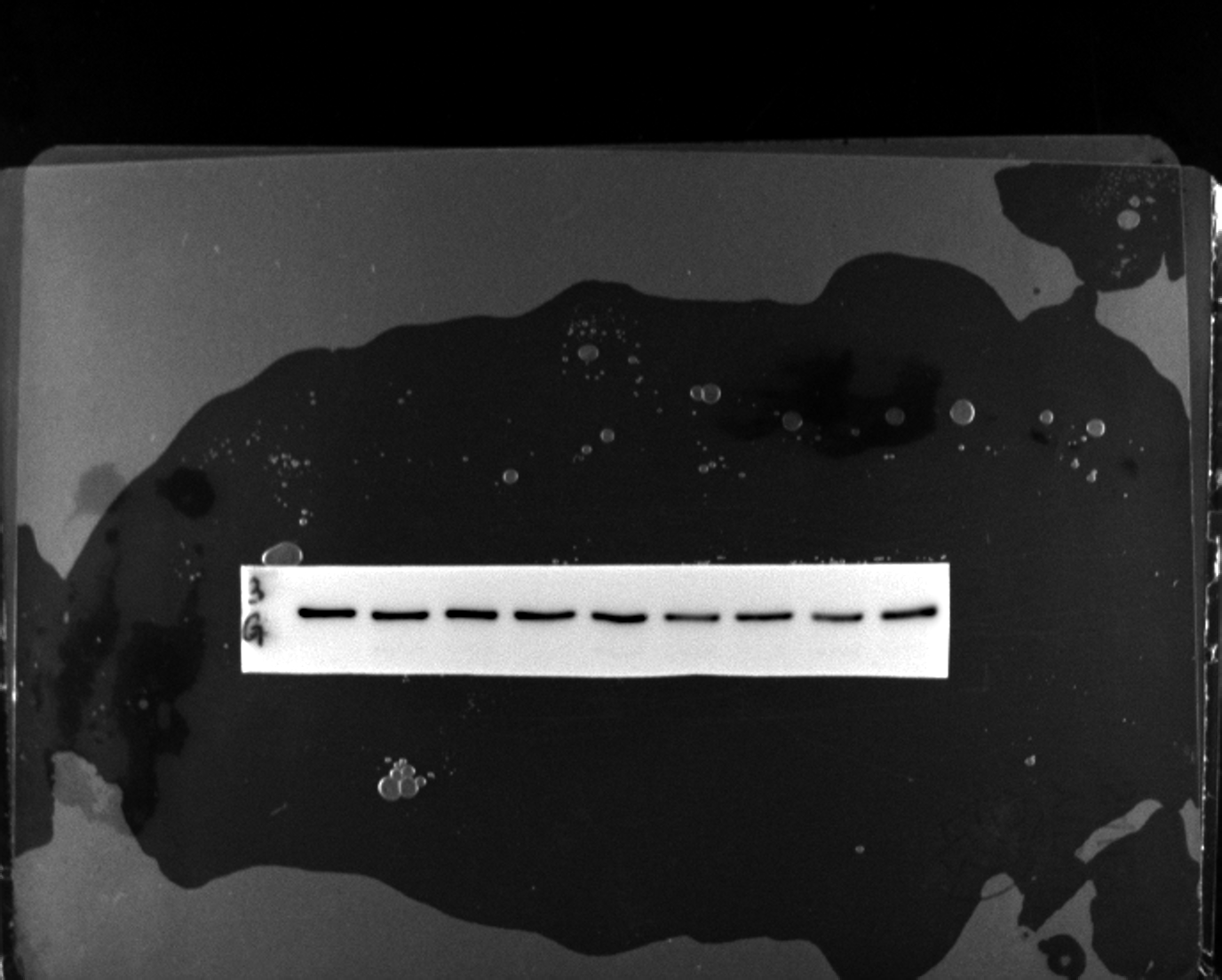

Supplement: Supplementary file 8 — Source Data Fig. 7 [file 44321_2024_25_MOESM8_ESM.zip › figure 7/7B/7B GAPDH for FTO.Tif]

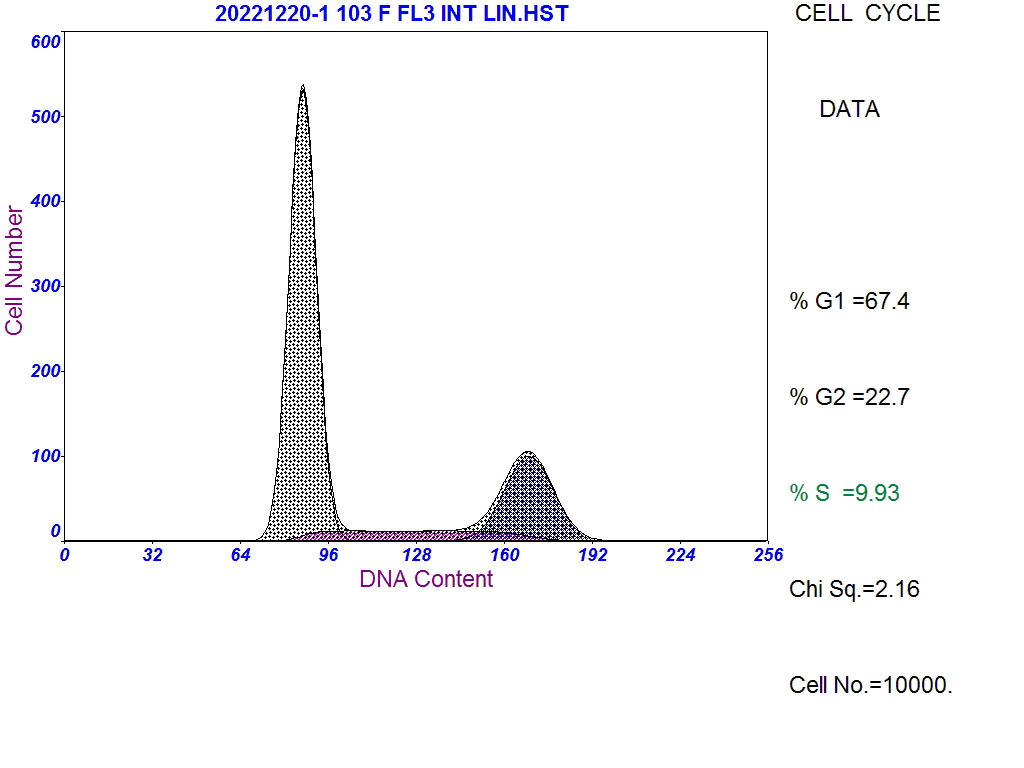

Supplement: Supplementary file 8 — Source Data Fig. 7 [file 44321_2024_25_MOESM8_ESM.zip › figure 7/7C/7C L-EV.JPG]

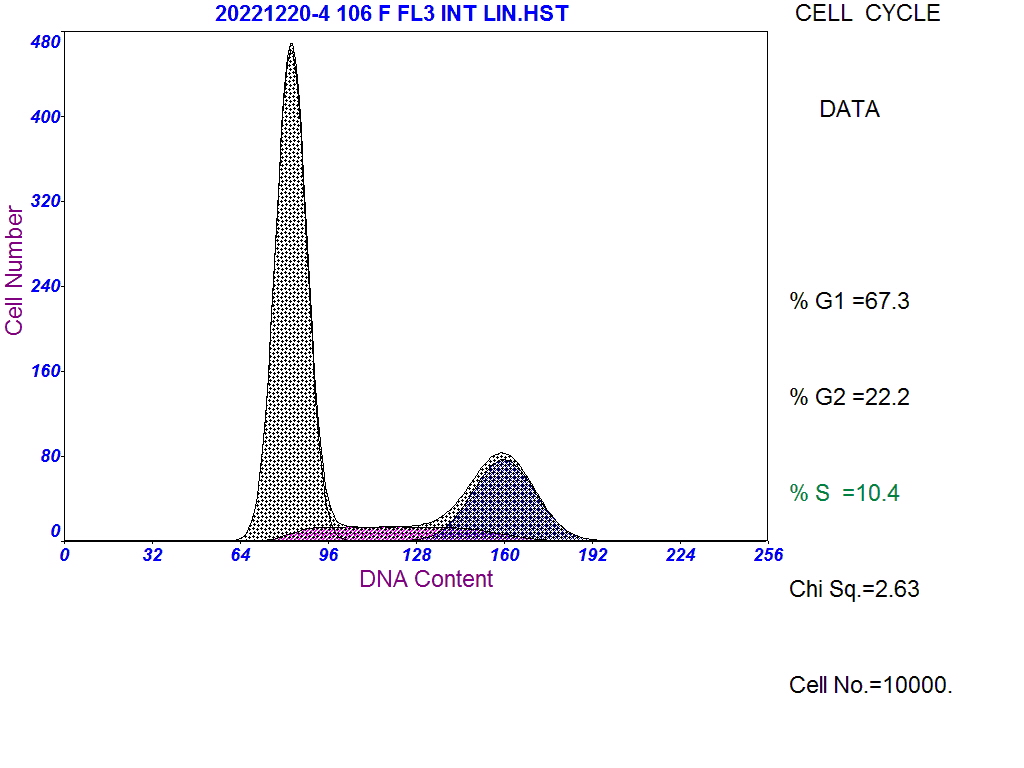

Supplement: Supplementary file 8 — Source Data Fig. 7 [file 44321_2024_25_MOESM8_ESM.zip › figure 7/7C/7C L-FTO MU.JPG]

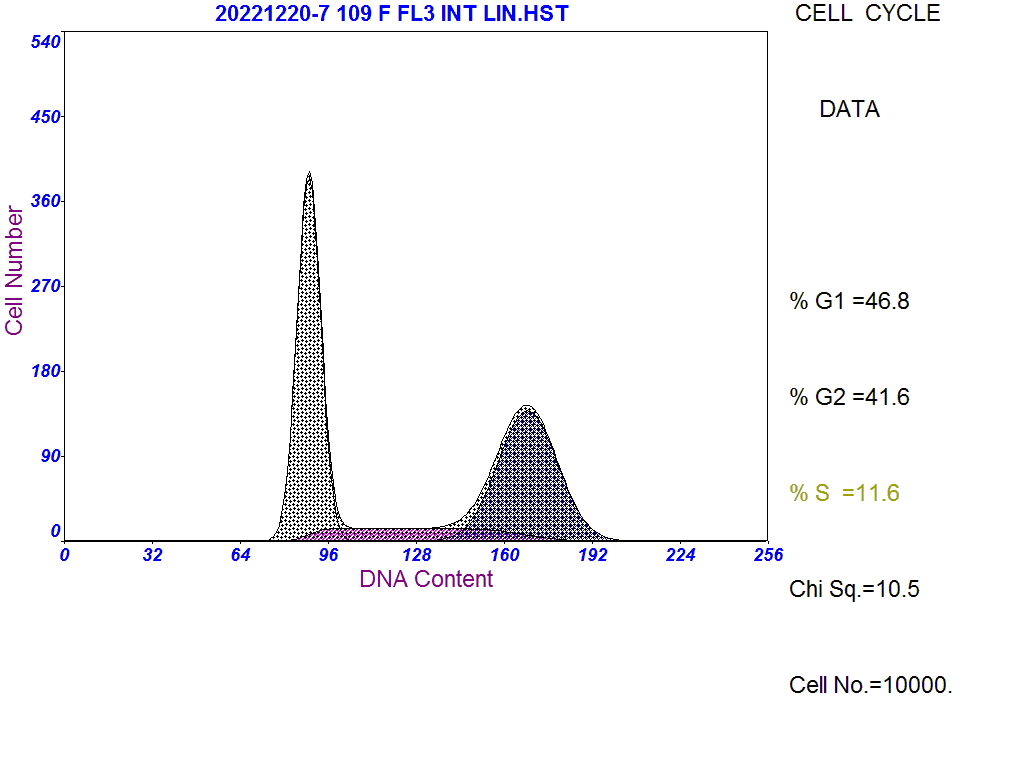

Supplement: Supplementary file 8 — Source Data Fig. 7 [file 44321_2024_25_MOESM8_ESM.zip › figure 7/7C/7C L-FTO WT.JPG]

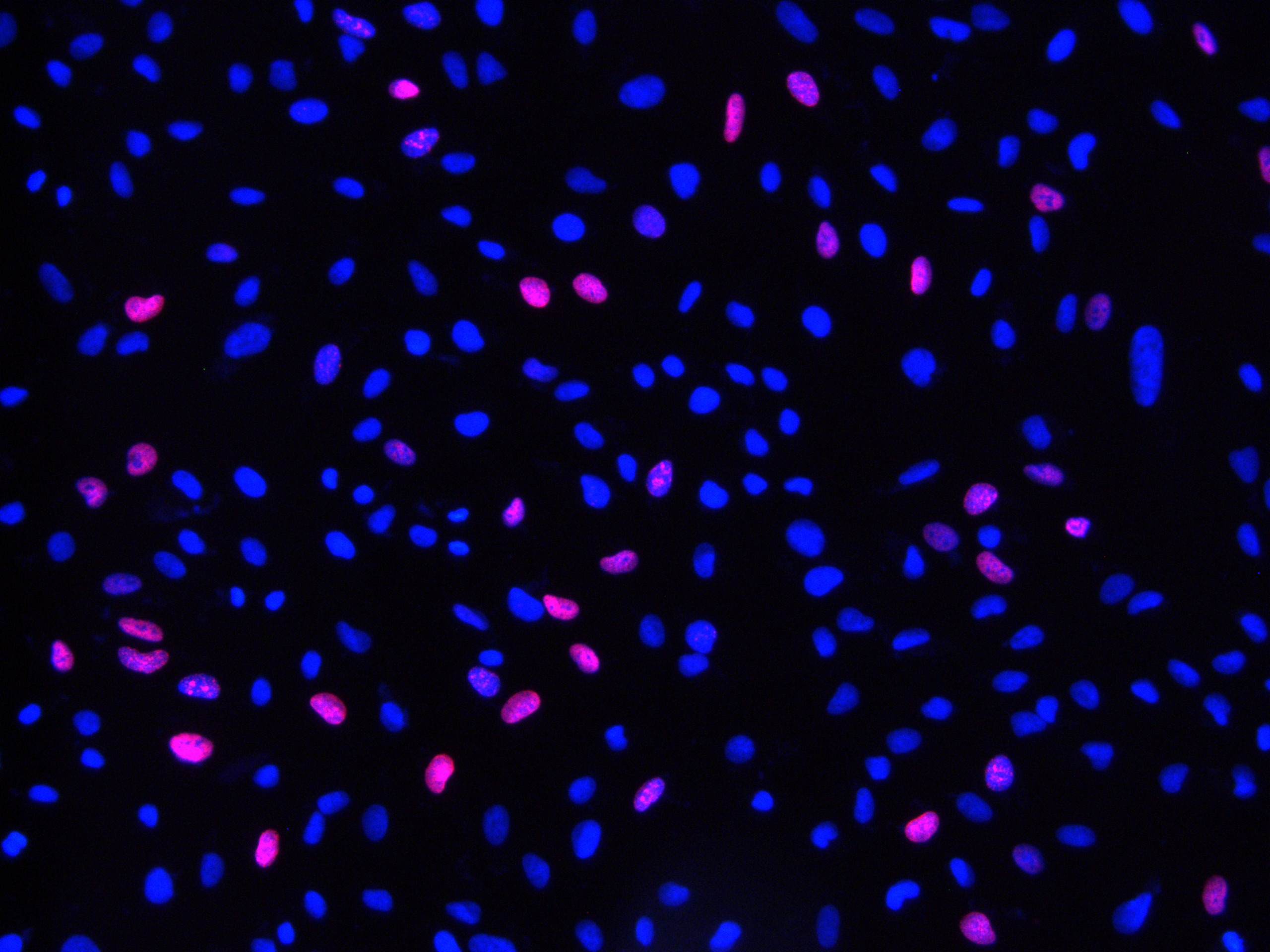

Supplement: Supplementary file 8 — Source Data Fig. 7 [file 44321_2024_25_MOESM8_ESM.zip › figure 7/7D/7D L-EV EdU DAPI.tif]

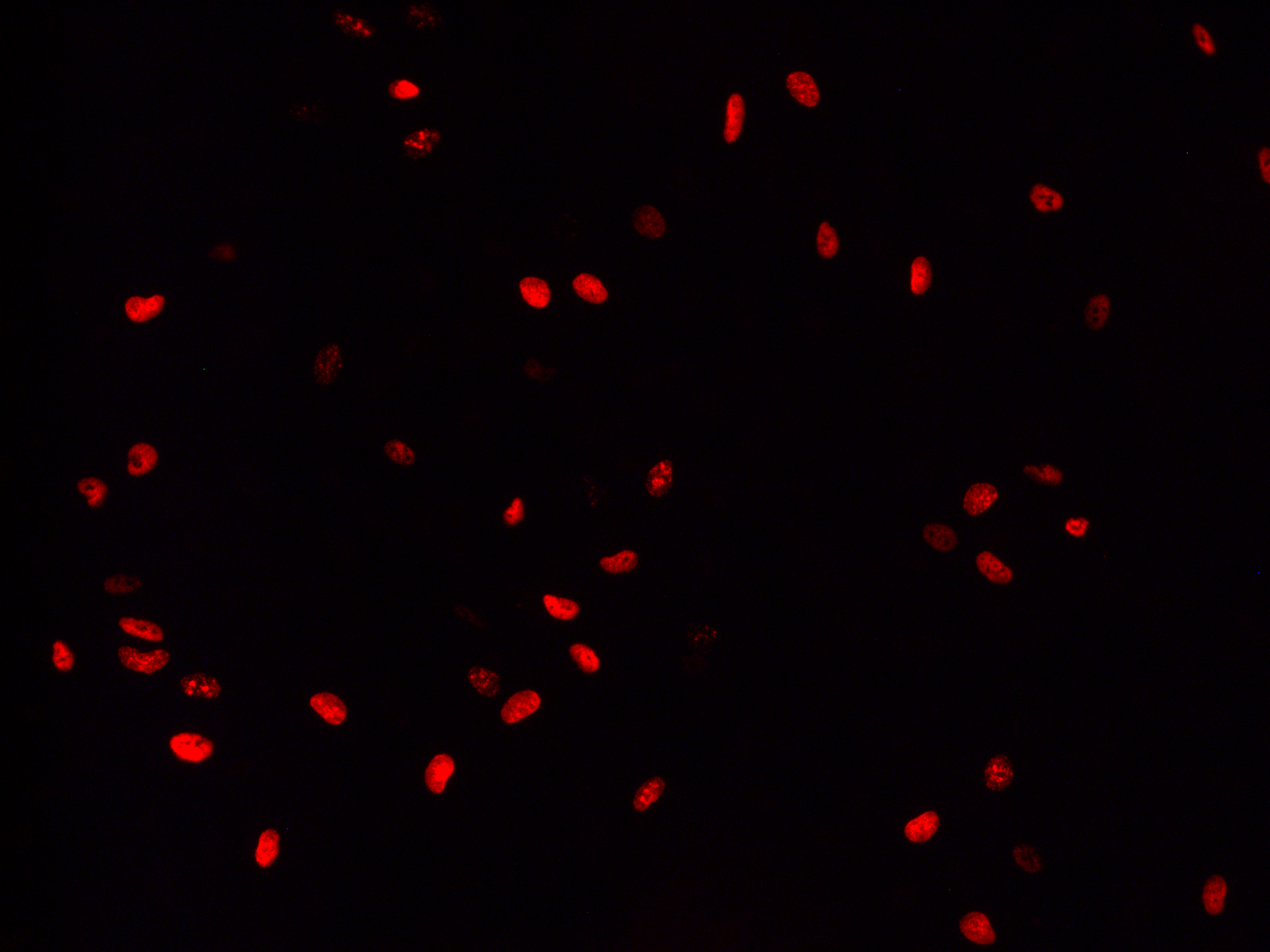

Supplement: Supplementary file 8 — Source Data Fig. 7 [file 44321_2024_25_MOESM8_ESM.zip › figure 7/7D/7D L-EV EdU.tif]

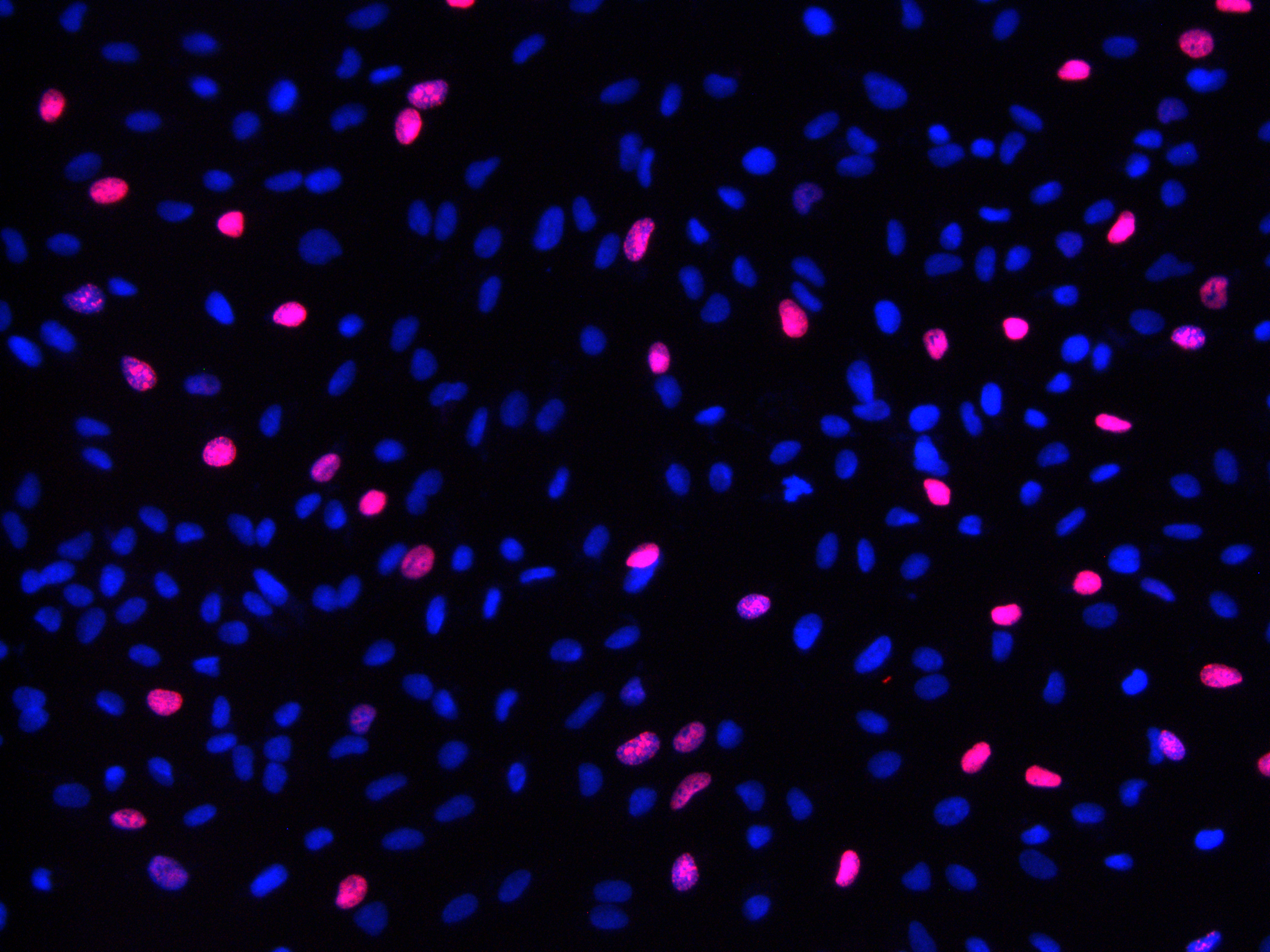

Supplement: Supplementary file 8 — Source Data Fig. 7 [file 44321_2024_25_MOESM8_ESM.zip › figure 7/7D/7D L-FTO MU EdU DAPI.tif]

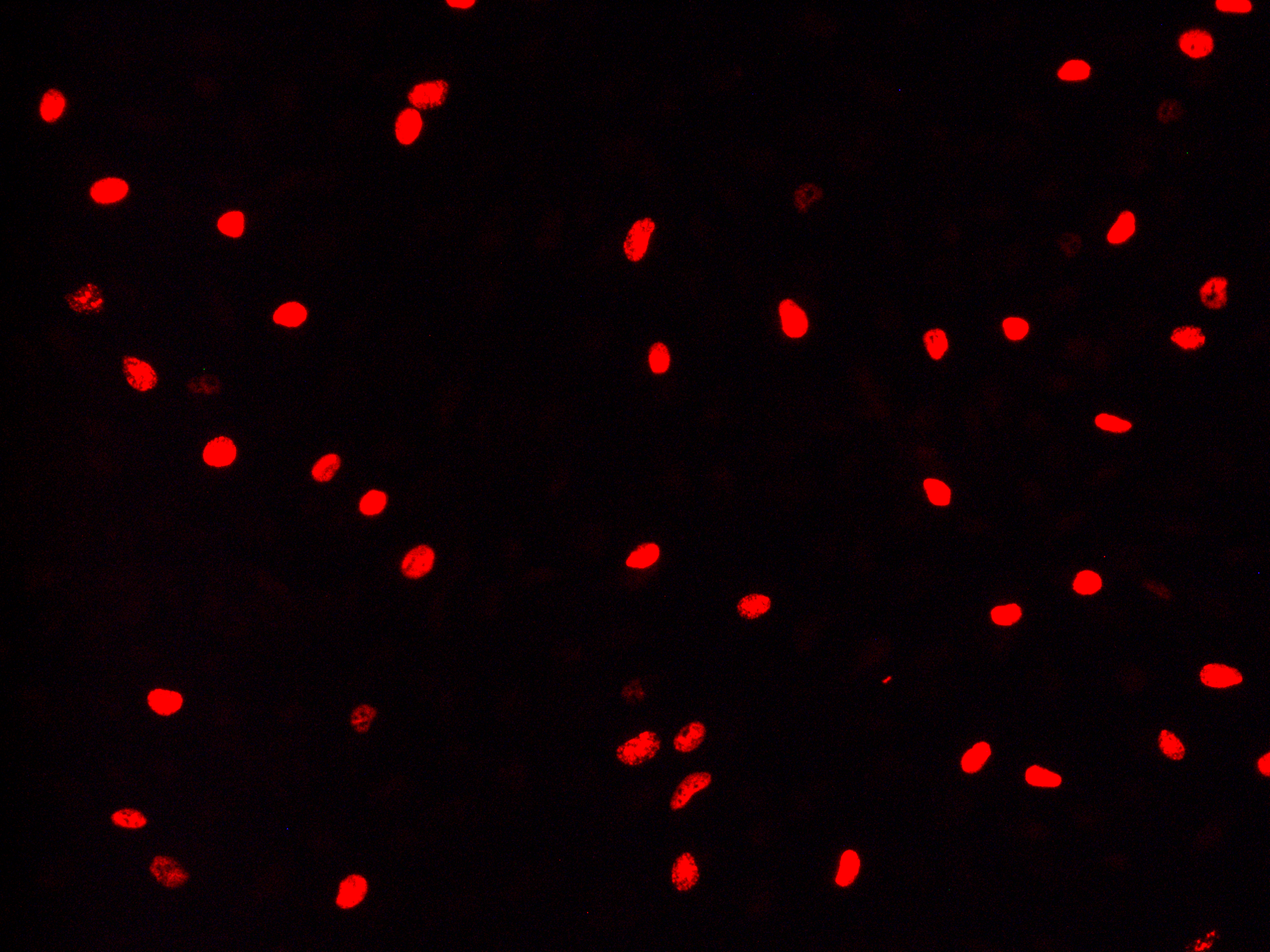

Supplement: Supplementary file 8 — Source Data Fig. 7 [file 44321_2024_25_MOESM8_ESM.zip › figure 7/7D/7D L-FTO MU EdU.tif]

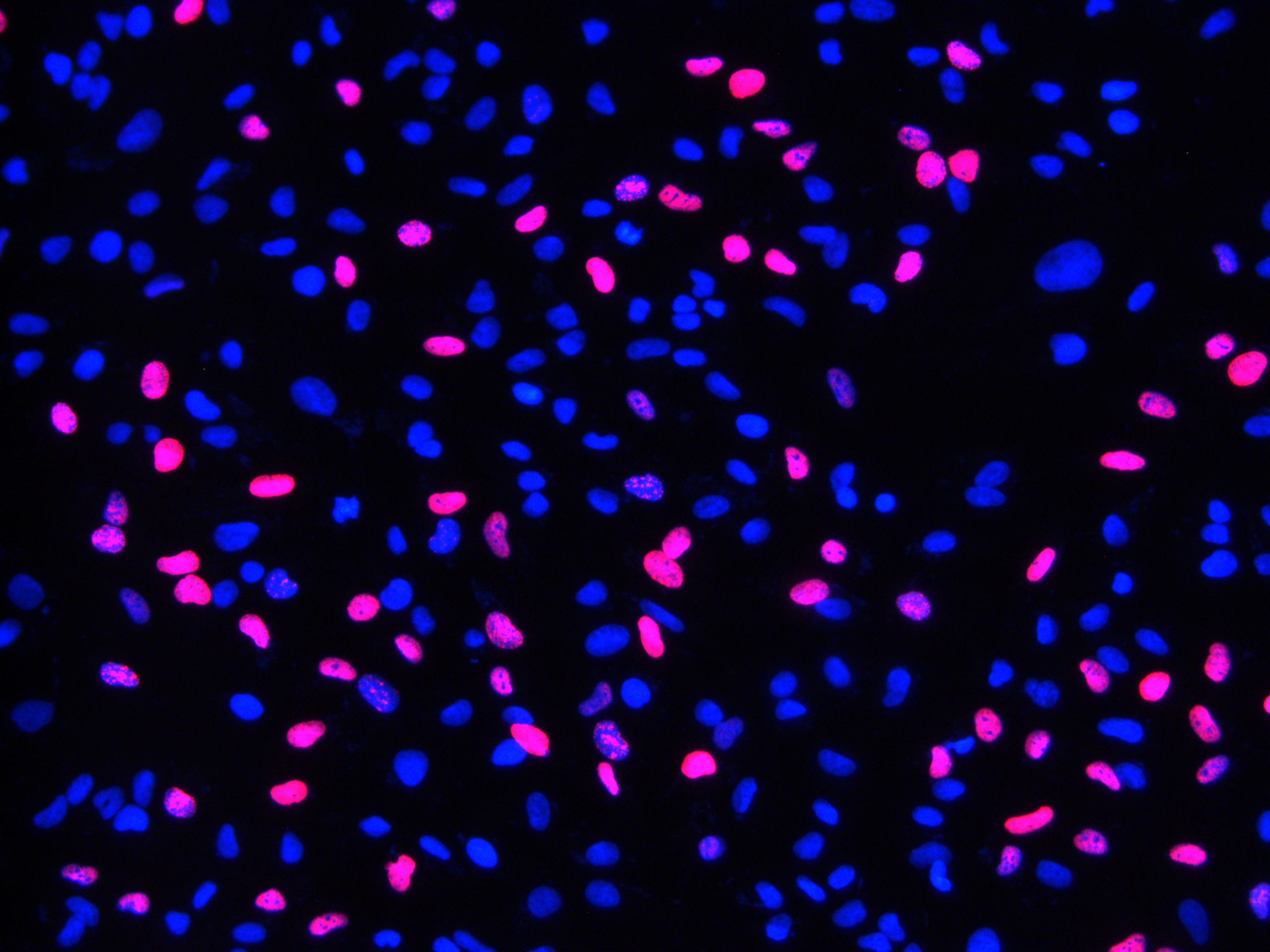

Supplement: Supplementary file 8 — Source Data Fig. 7 [file 44321_2024_25_MOESM8_ESM.zip › figure 7/7D/7D L-FTO WT EdU DAPI.tif]

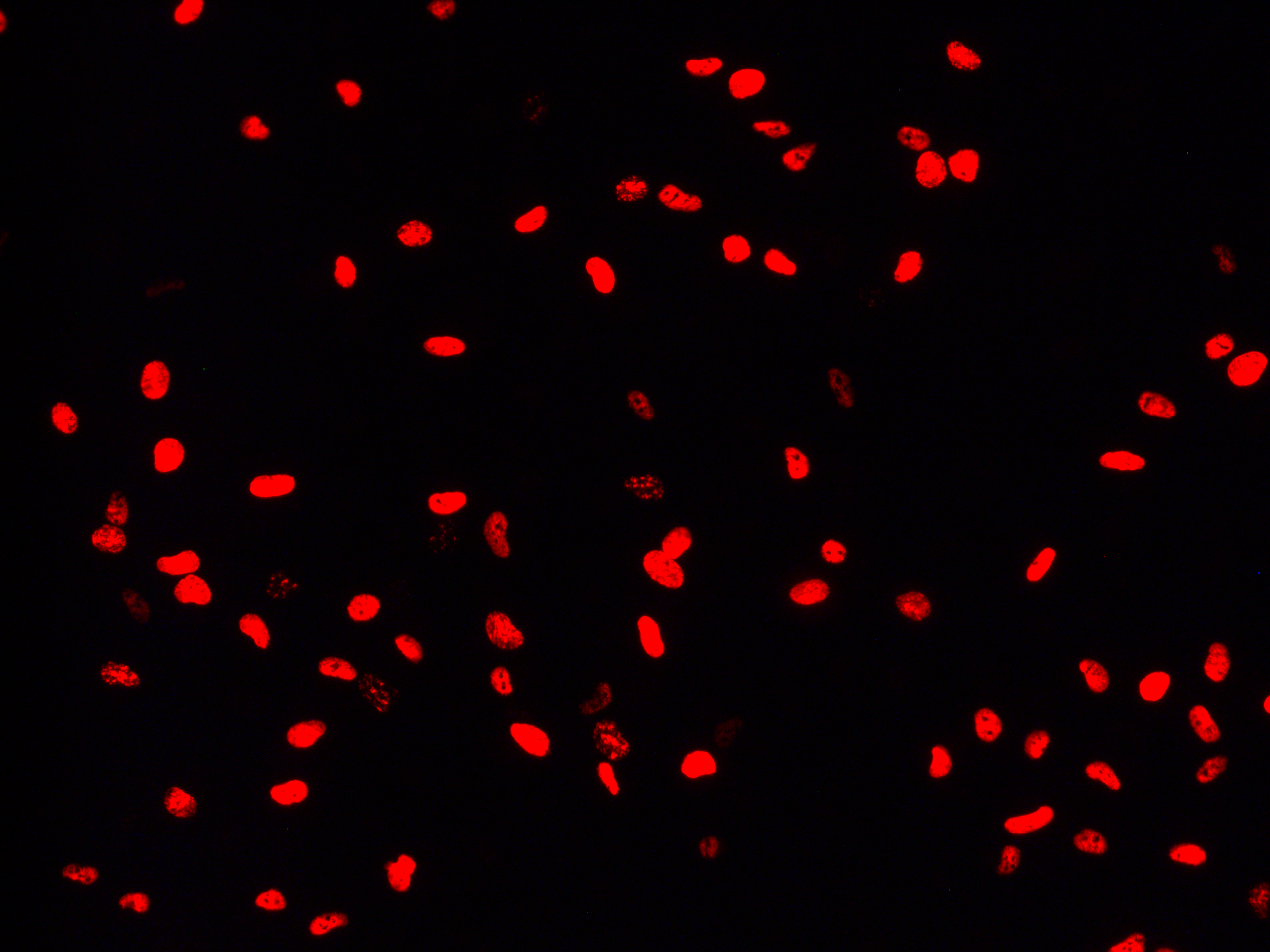

Supplement: Supplementary file 8 — Source Data Fig. 7 [file 44321_2024_25_MOESM8_ESM.zip › figure 7/7D/7D L-FTO WT EdU.tif]

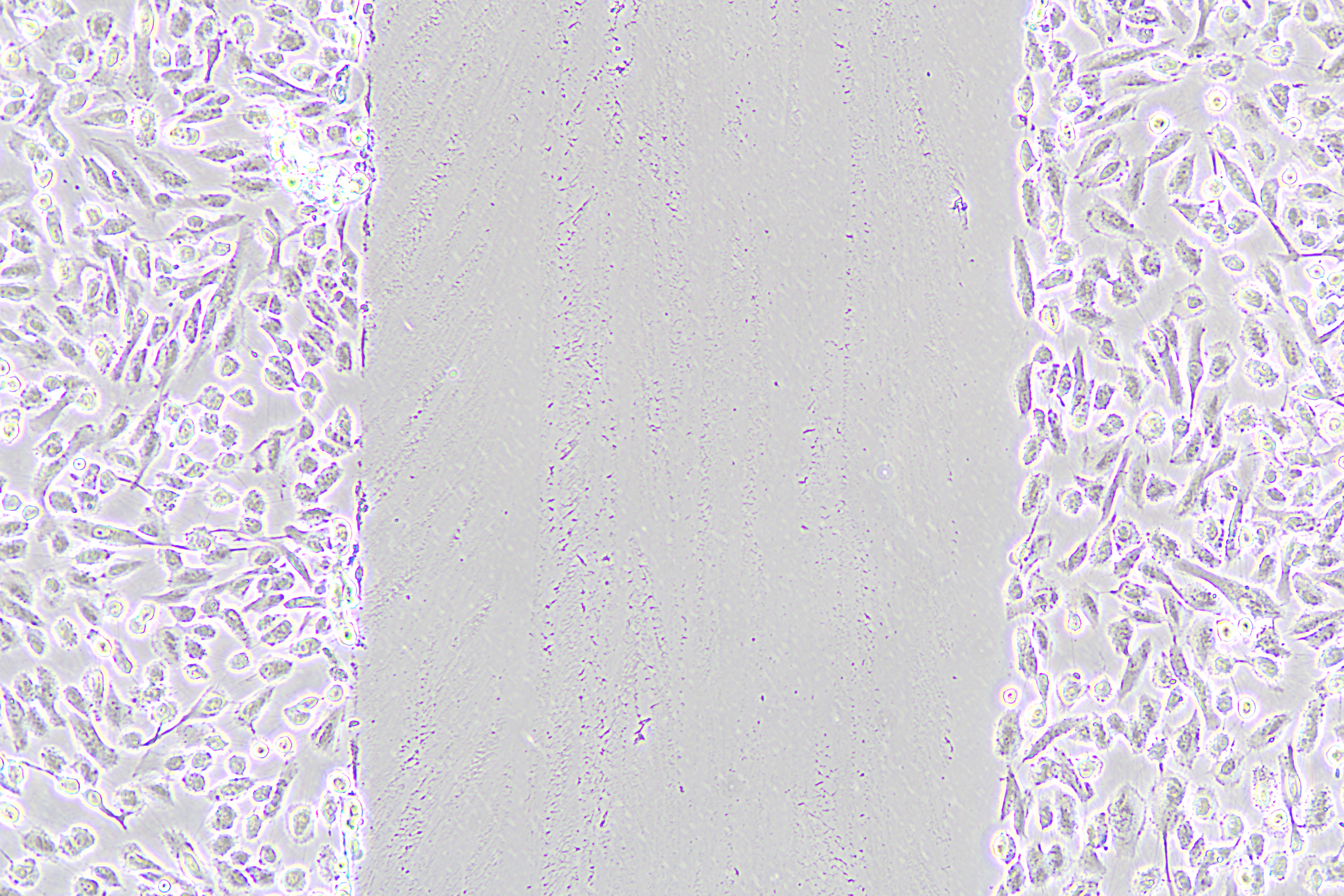

Supplement: Supplementary file 8 — Source Data Fig. 7 [file 44321_2024_25_MOESM8_ESM.zip › figure 7/7E/7E L-EV 0h.tif]

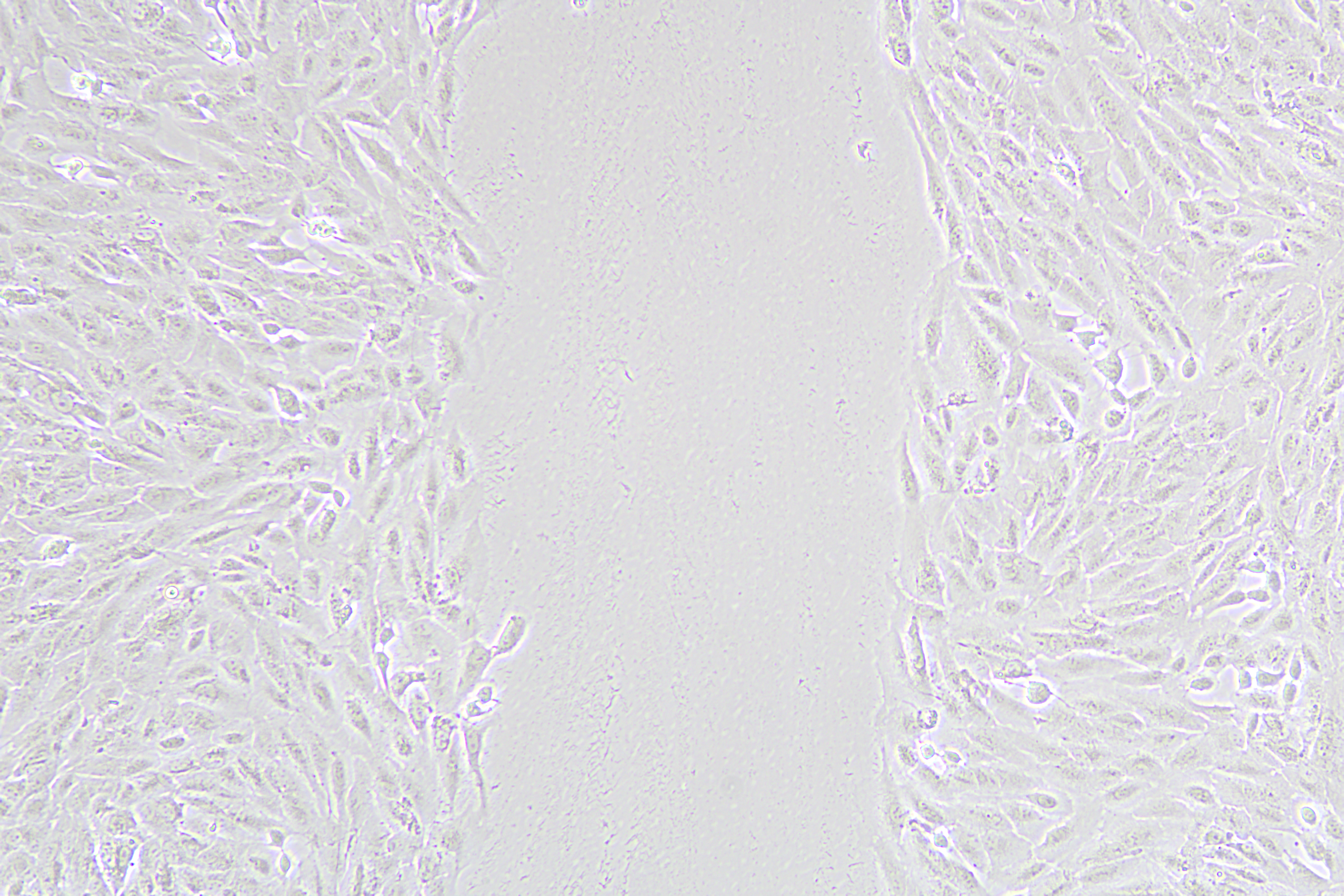

Supplement: Supplementary file 8 — Source Data Fig. 7 [file 44321_2024_25_MOESM8_ESM.zip › figure 7/7E/7E L-EV 24h.tif]

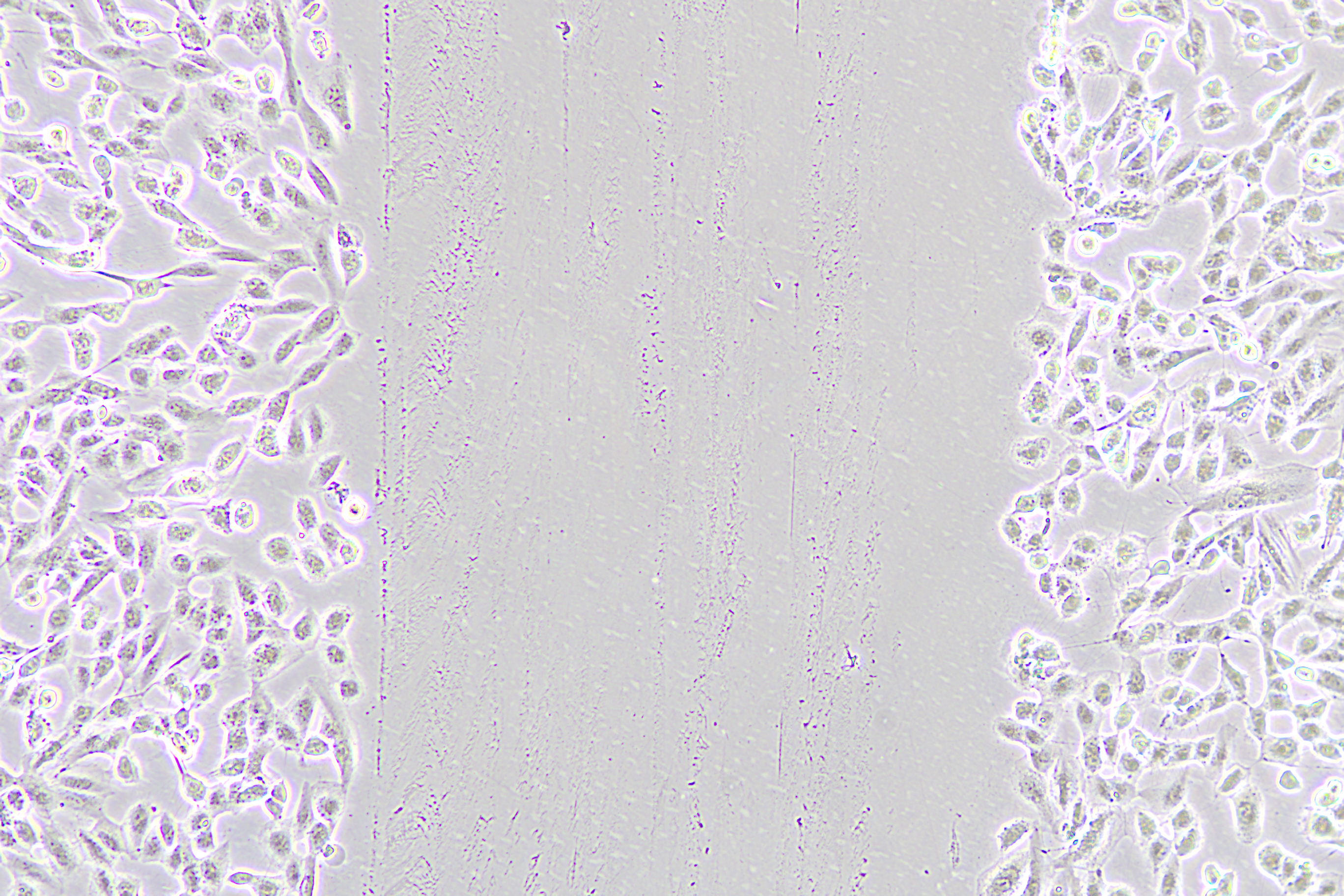

Supplement: Supplementary file 8 — Source Data Fig. 7 [file 44321_2024_25_MOESM8_ESM.zip › figure 7/7E/7E L-FTO MU 0h.tif]

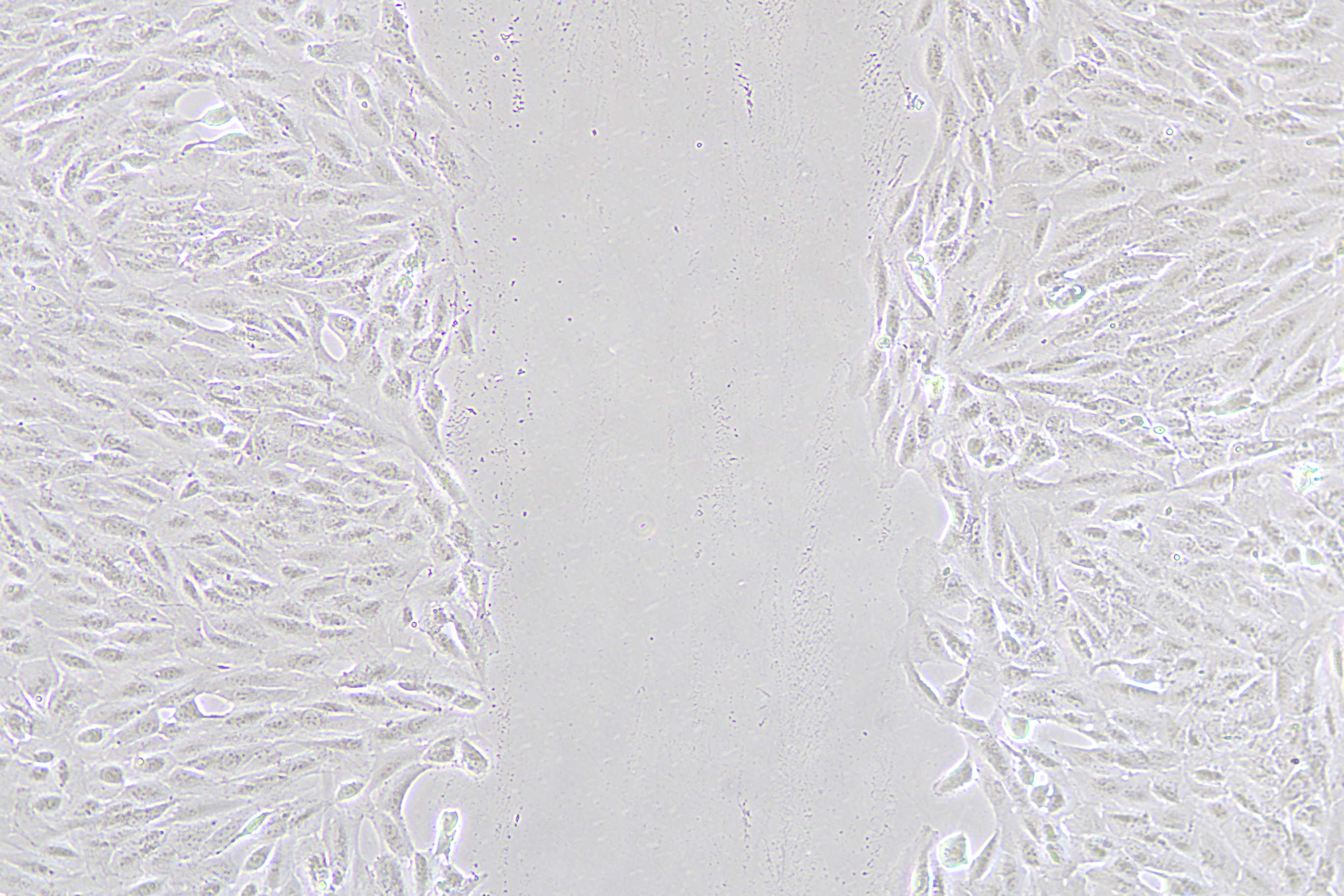

Supplement: Supplementary file 8 — Source Data Fig. 7 [file 44321_2024_25_MOESM8_ESM.zip › figure 7/7E/7E L-FTO MU 24h.tif]

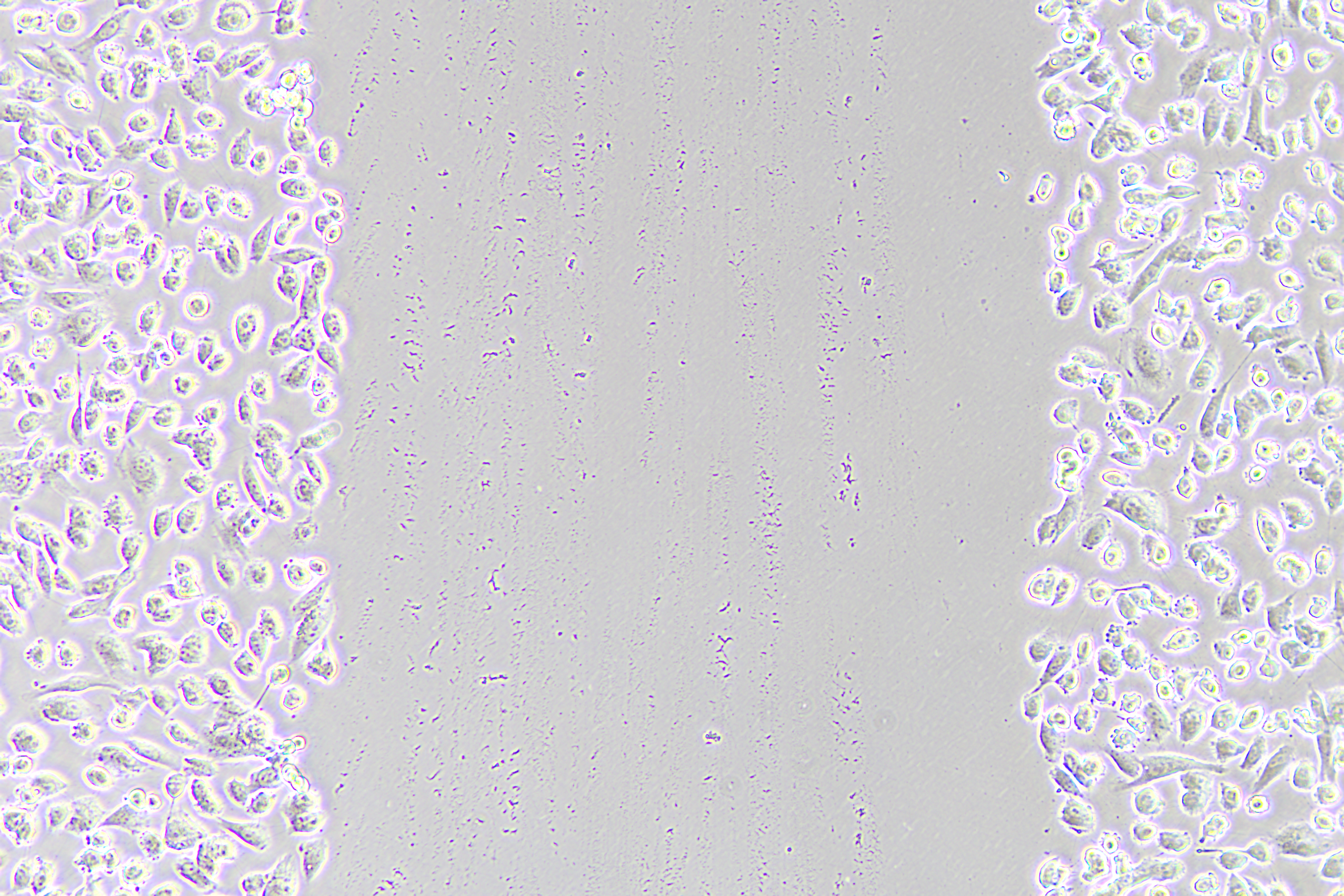

Supplement: Supplementary file 8 — Source Data Fig. 7 [file 44321_2024_25_MOESM8_ESM.zip › figure 7/7E/7E L-FTO WT 0h.tif]

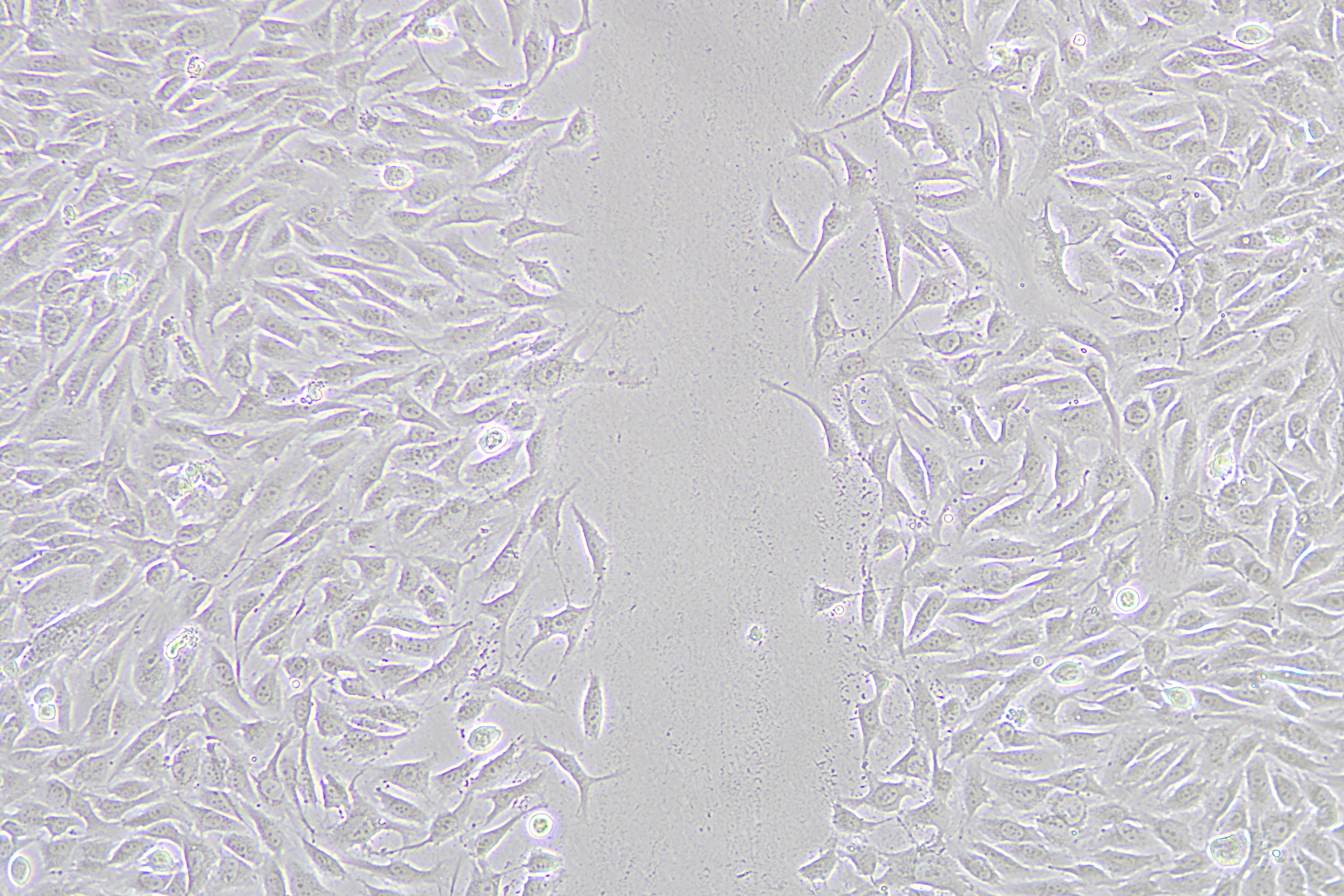

Supplement: Supplementary file 8 — Source Data Fig. 7 [file 44321_2024_25_MOESM8_ESM.zip › figure 7/7E/7E L-FTO WT 24h.tif]

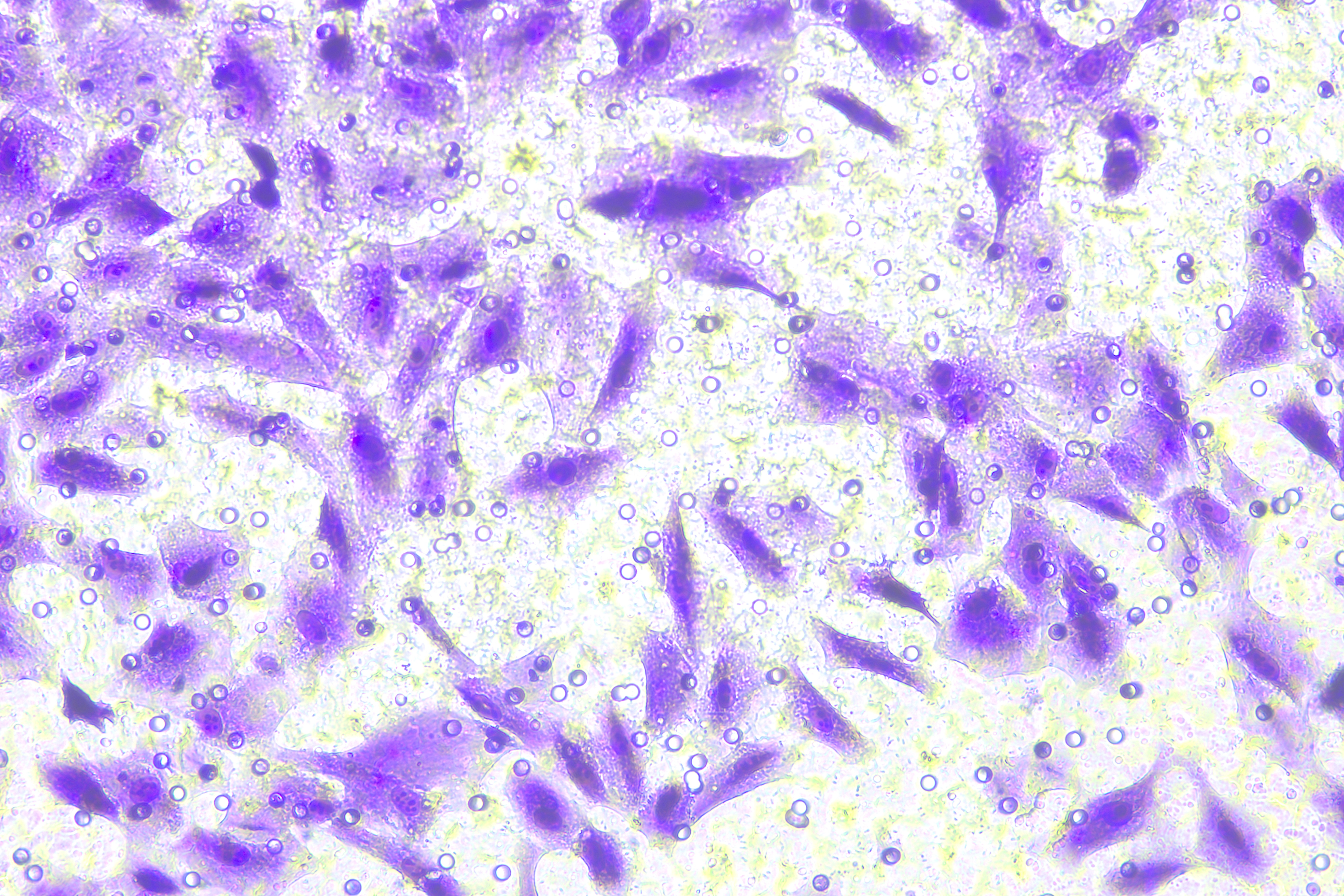

Supplement: Supplementary file 8 — Source Data Fig. 7 [file 44321_2024_25_MOESM8_ESM.zip › figure 7/7F/7F L-EV.tif]

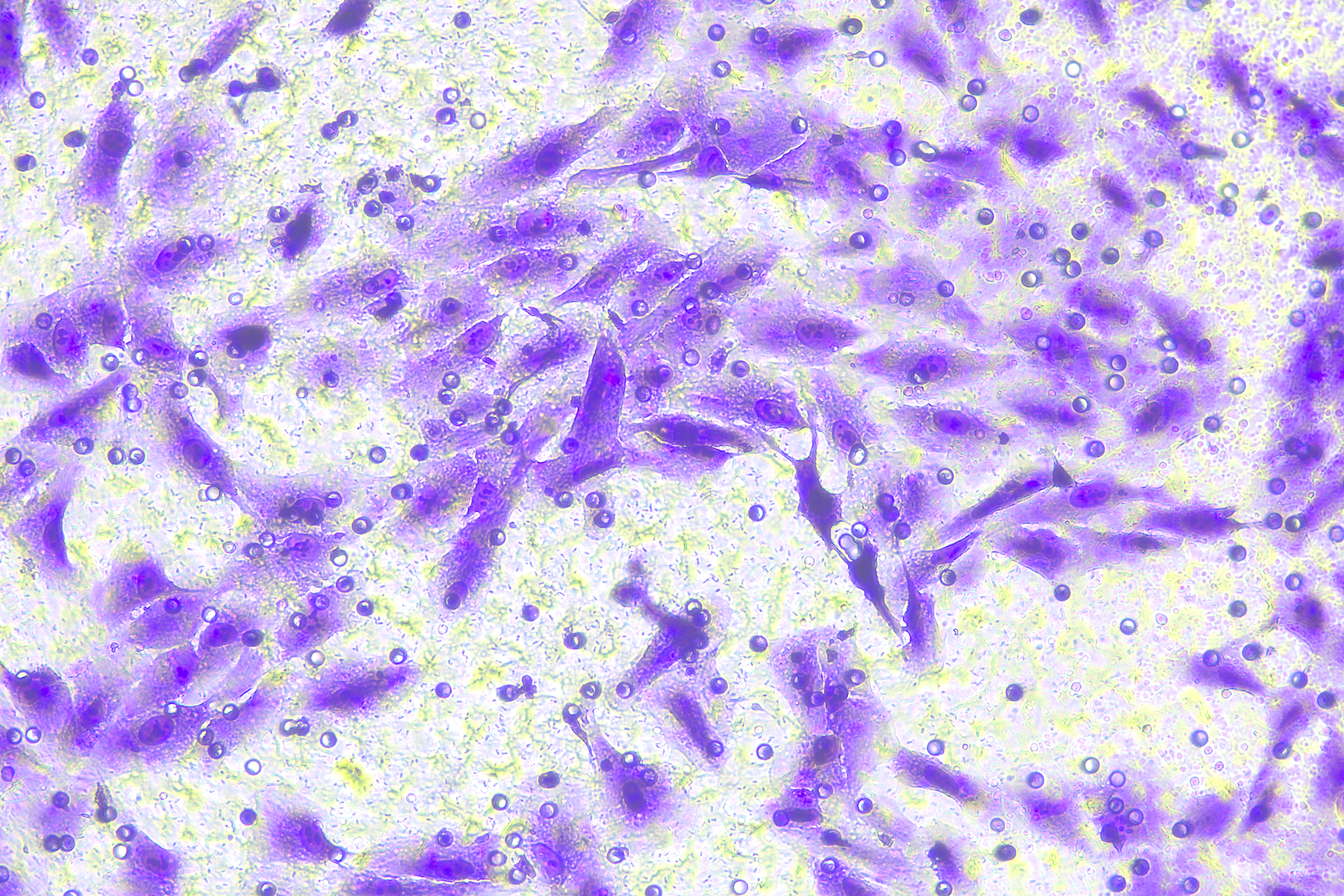

Supplement: Supplementary file 8 — Source Data Fig. 7 [file 44321_2024_25_MOESM8_ESM.zip › figure 7/7F/7F L-FTO MU.tif]

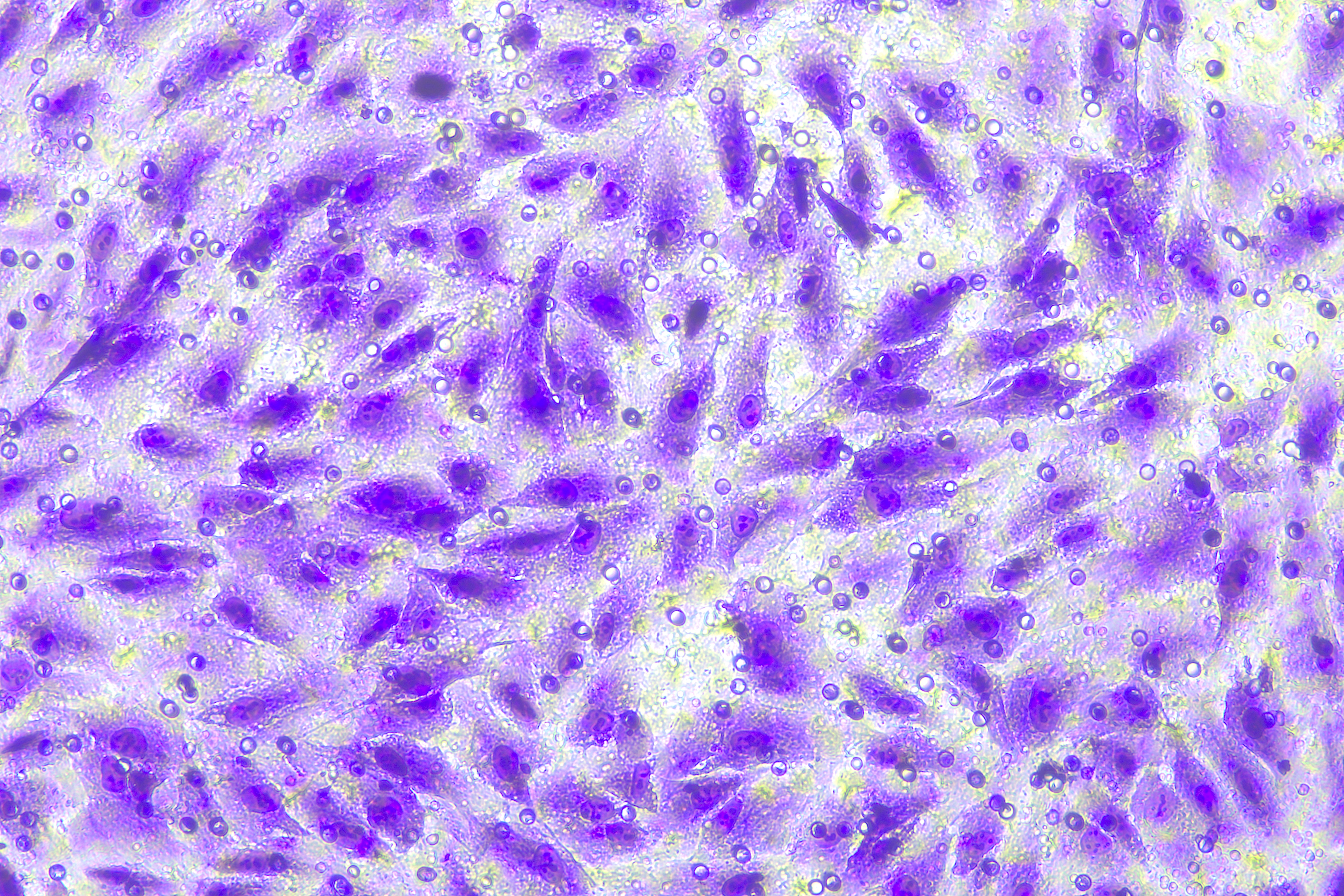

Supplement: Supplementary file 8 — Source Data Fig. 7 [file 44321_2024_25_MOESM8_ESM.zip › figure 7/7F/7F L-FTO WT.tif]

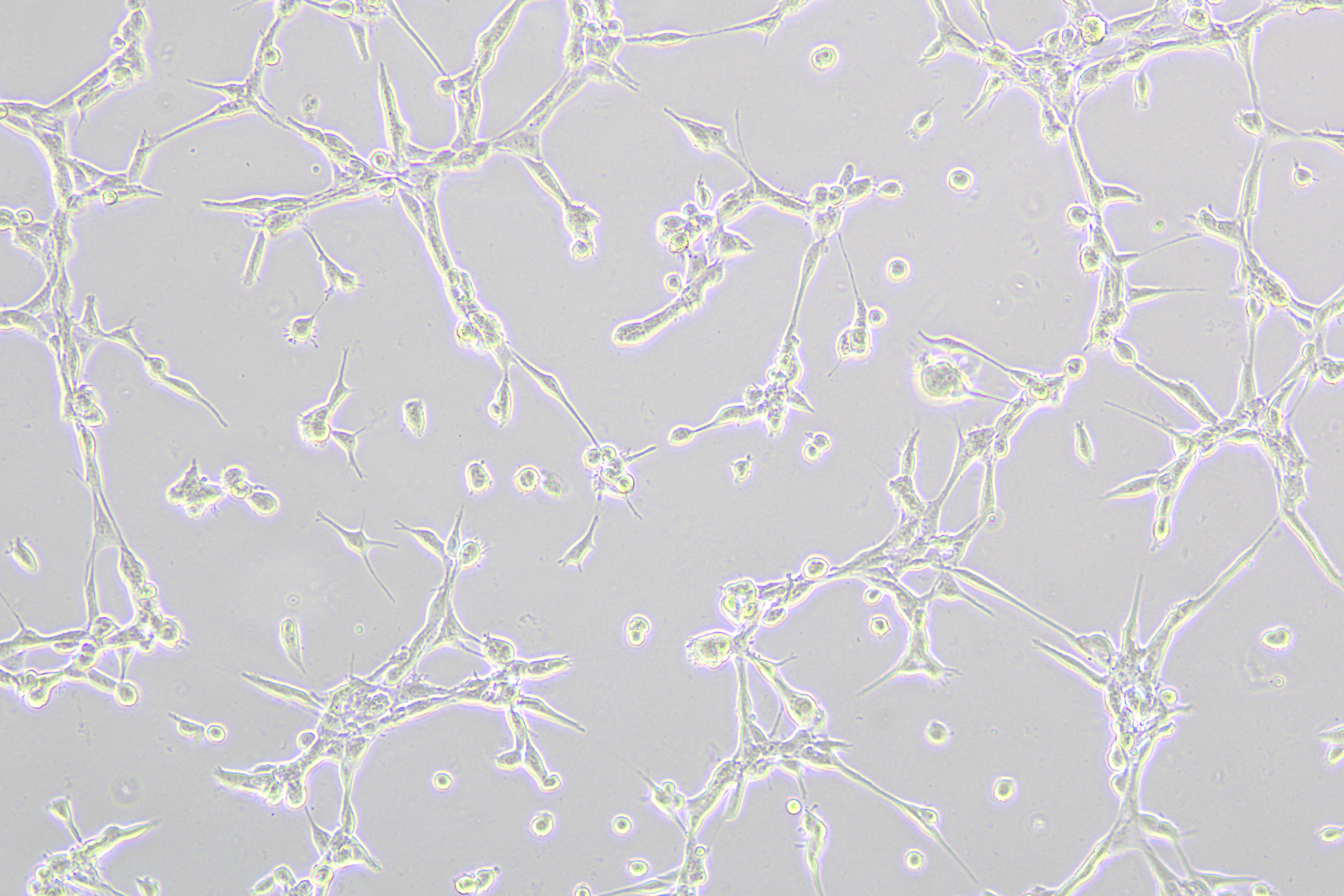

Supplement: Supplementary file 8 — Source Data Fig. 7 [file 44321_2024_25_MOESM8_ESM.zip › figure 7/7G/7G L-EV.tif]

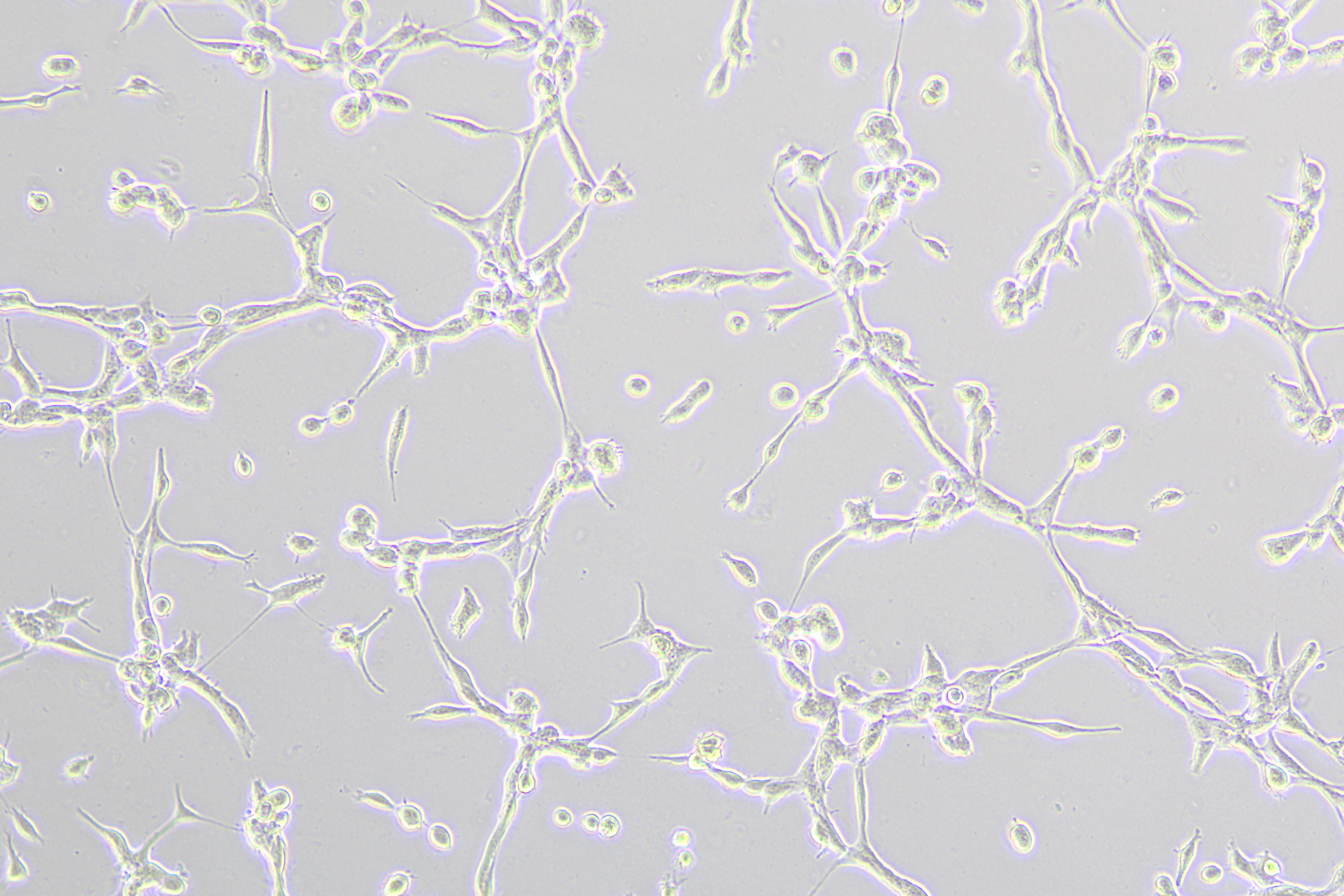

Supplement: Supplementary file 8 — Source Data Fig. 7 [file 44321_2024_25_MOESM8_ESM.zip › figure 7/7G/7G L-FTO MU.tif]

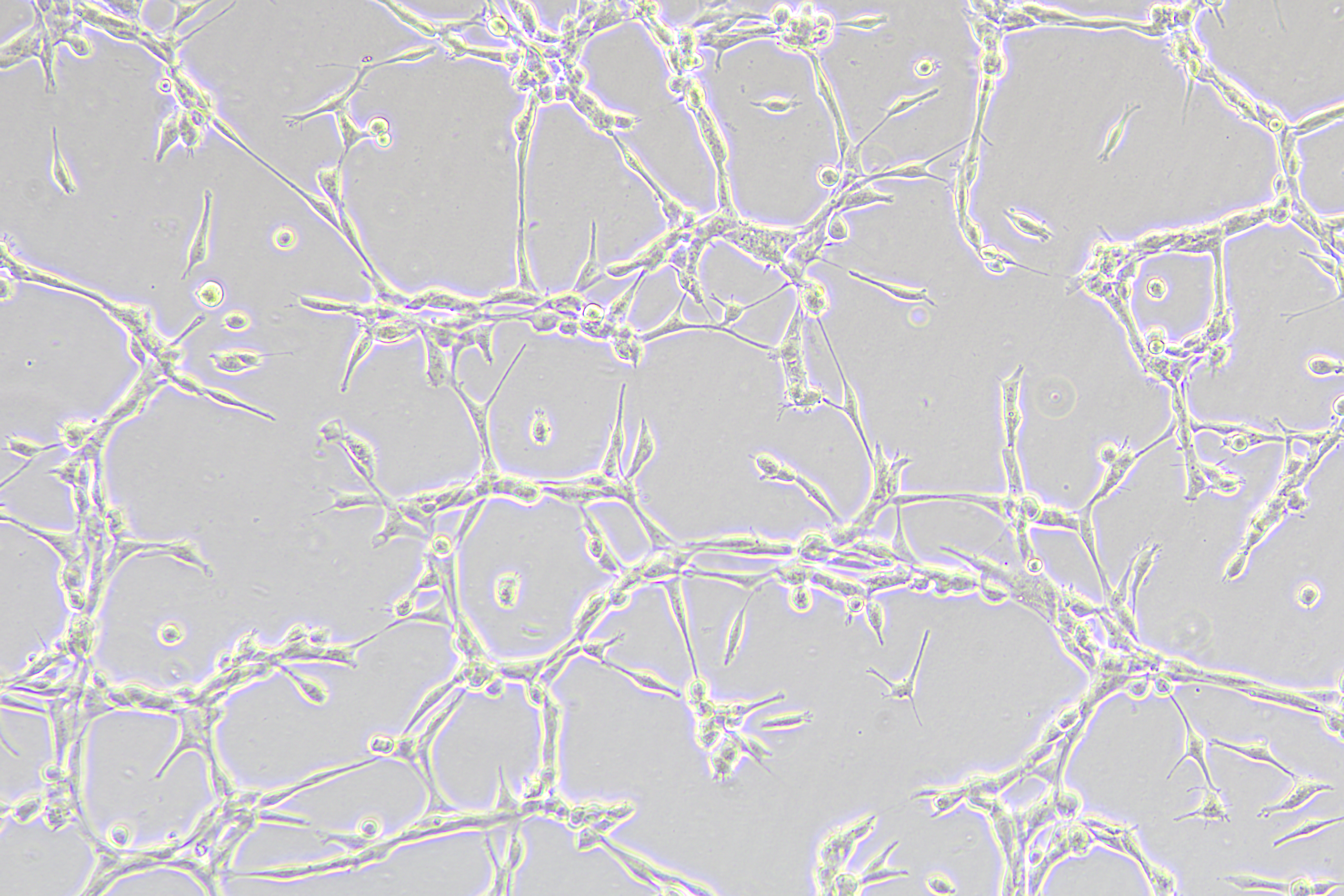

Supplement: Supplementary file 8 — Source Data Fig. 7 [file 44321_2024_25_MOESM8_ESM.zip › figure 7/7G/7G L-FTO WT.tif]

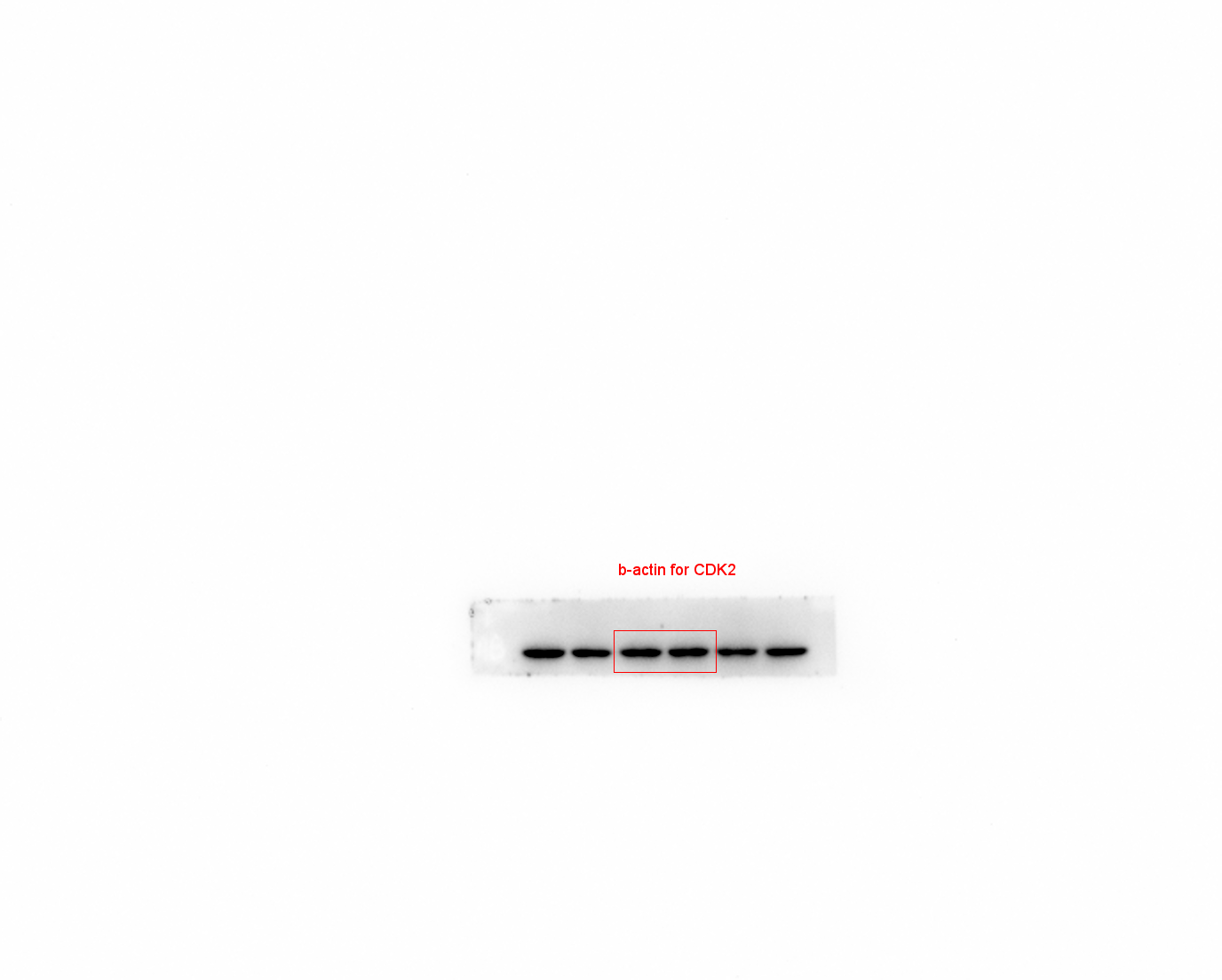

Supplement: Supplementary file 9 — Source Data Fig. 8 [file 44321_2024_25_MOESM9_ESM.zip › figure 8/8G/8G bactin mark.Tif]

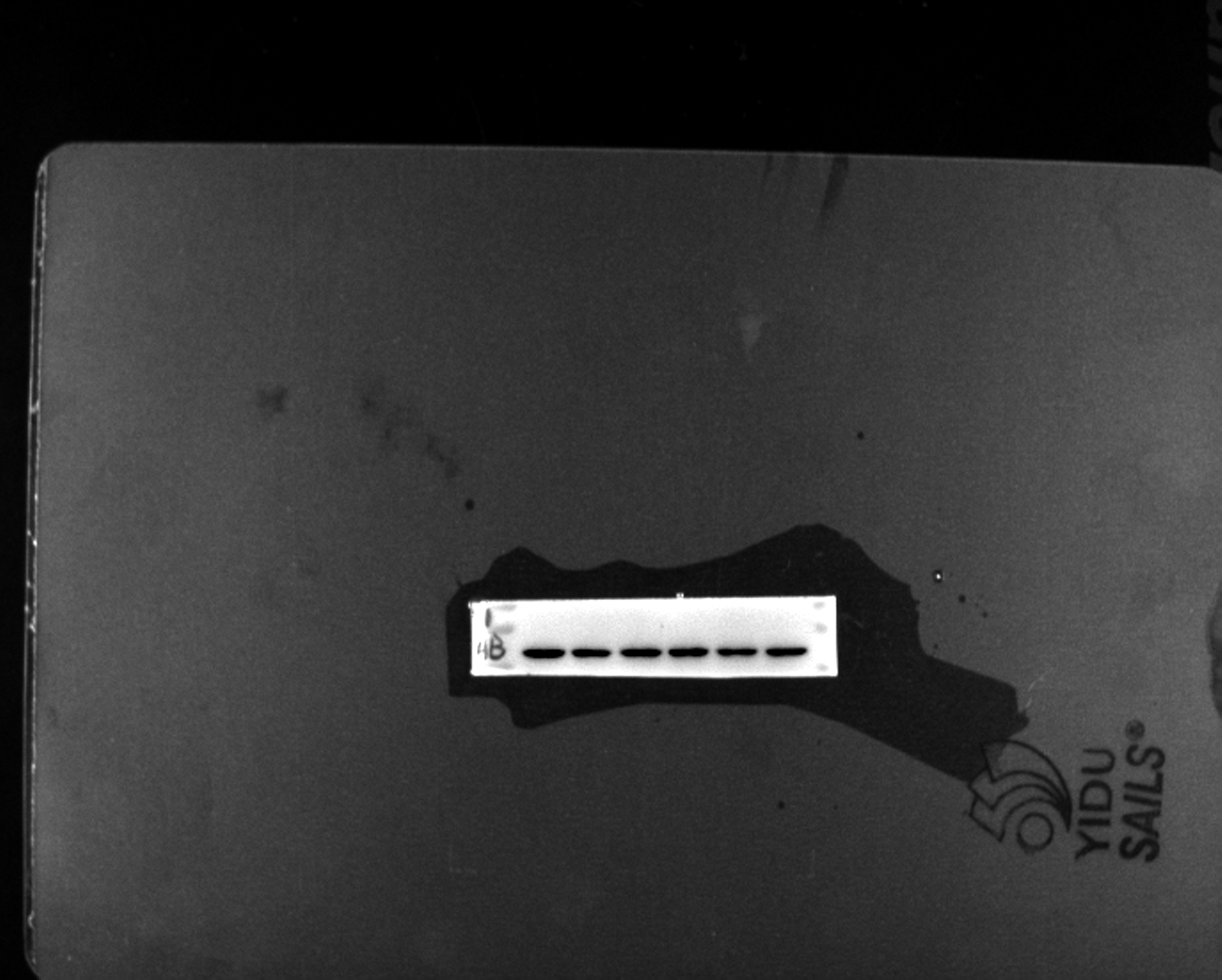

Supplement: Supplementary file 9 — Source Data Fig. 8 [file 44321_2024_25_MOESM9_ESM.zip › figure 8/8G/8G bactin.Tif]

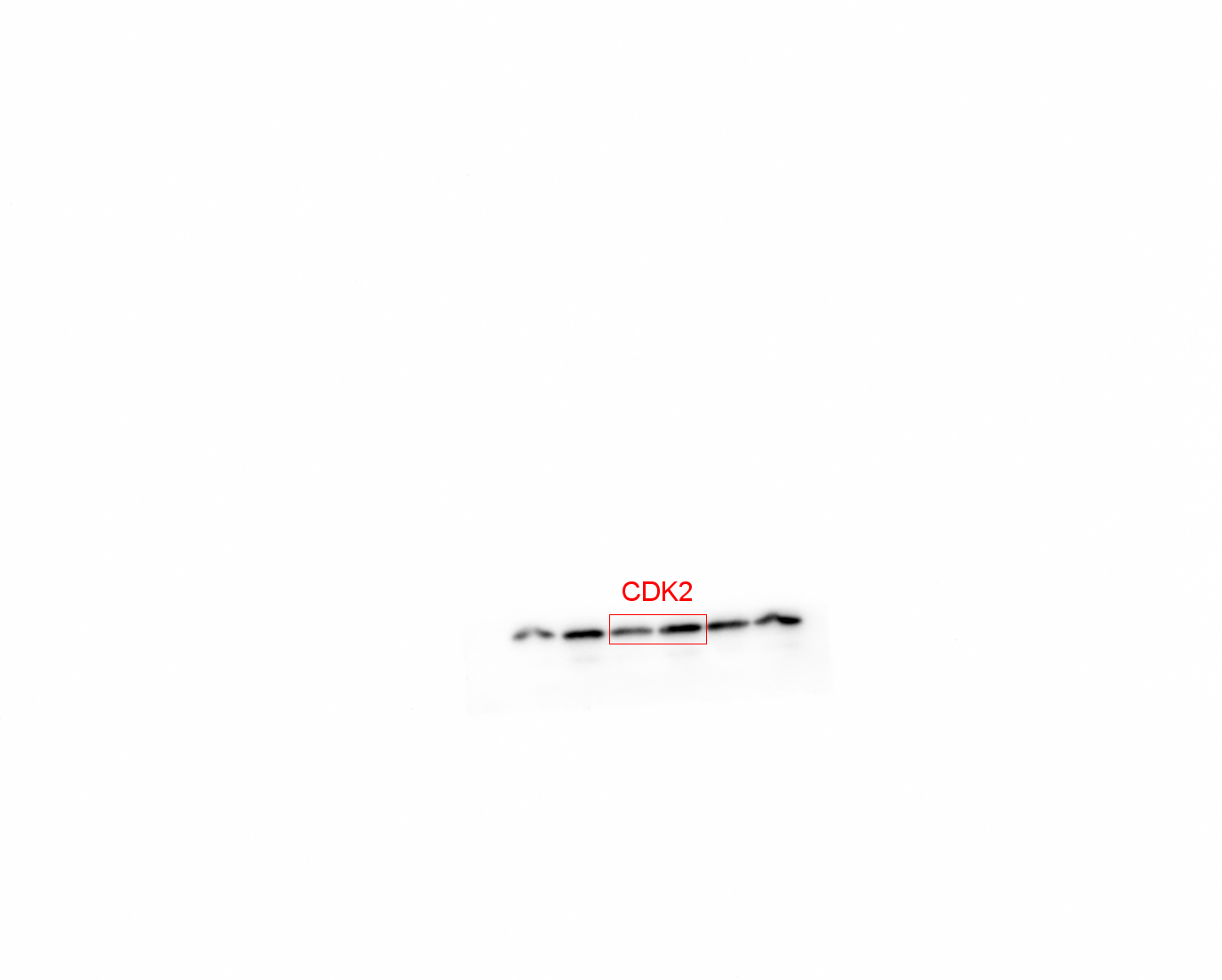

Supplement: Supplementary file 9 — Source Data Fig. 8 [file 44321_2024_25_MOESM9_ESM.zip › figure 8/8G/8G CDK2 mark.Tif]

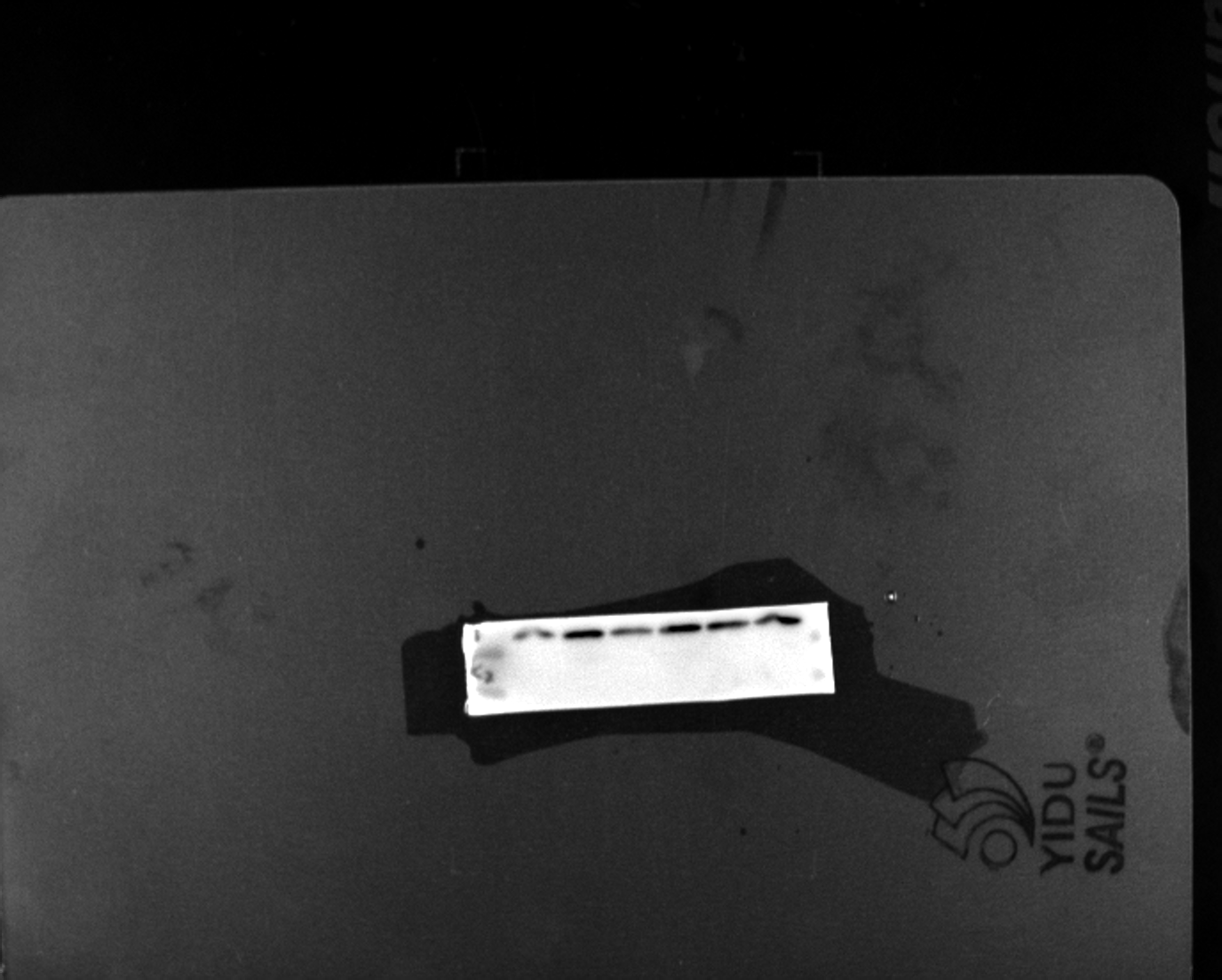

Supplement: Supplementary file 9 — Source Data Fig. 8 [file 44321_2024_25_MOESM9_ESM.zip › figure 8/8G/8G CDK2.Tif]

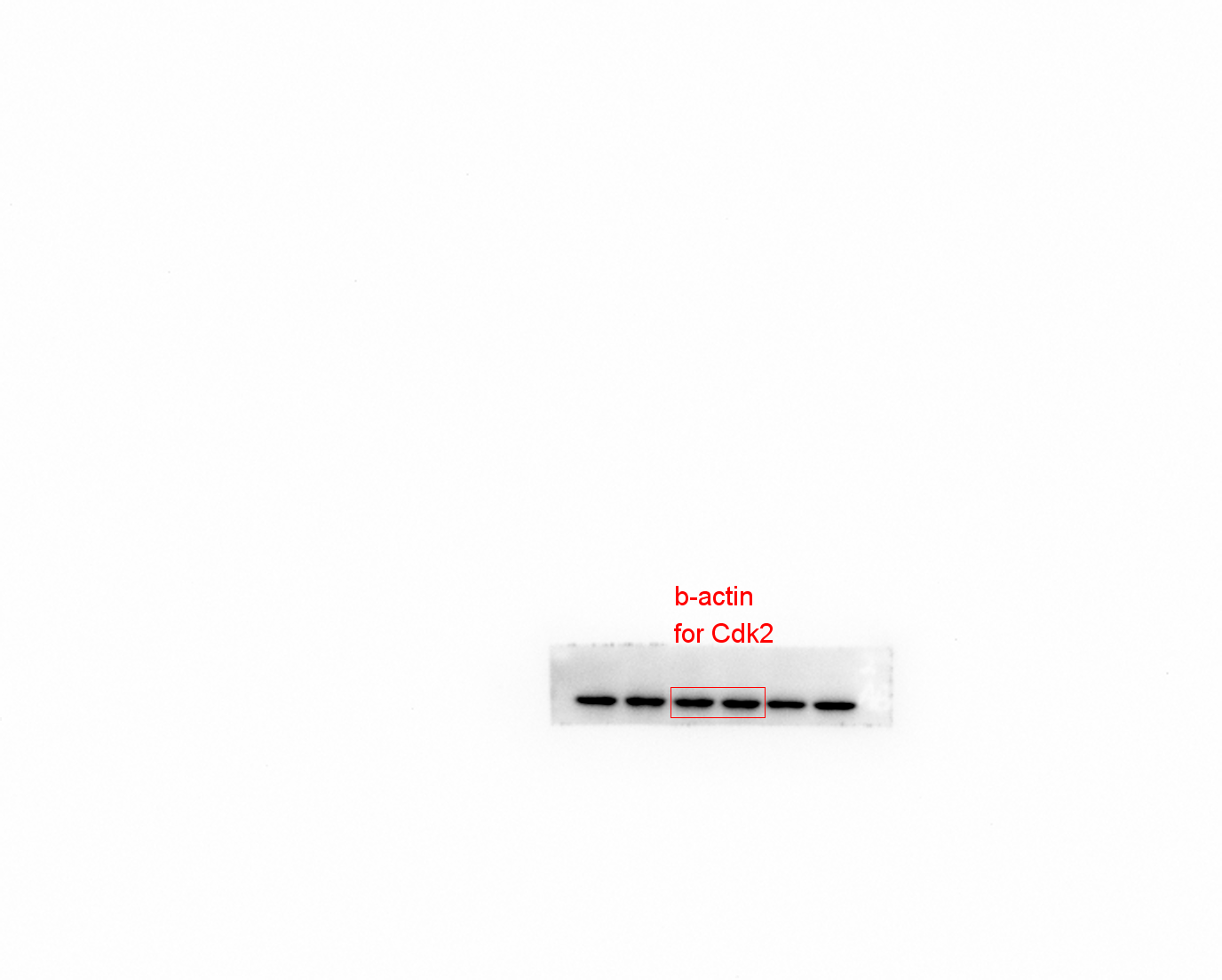

Supplement: Supplementary file 9 — Source Data Fig. 8 [file 44321_2024_25_MOESM9_ESM.zip › figure 8/8I/8I bactin mark.Tif]

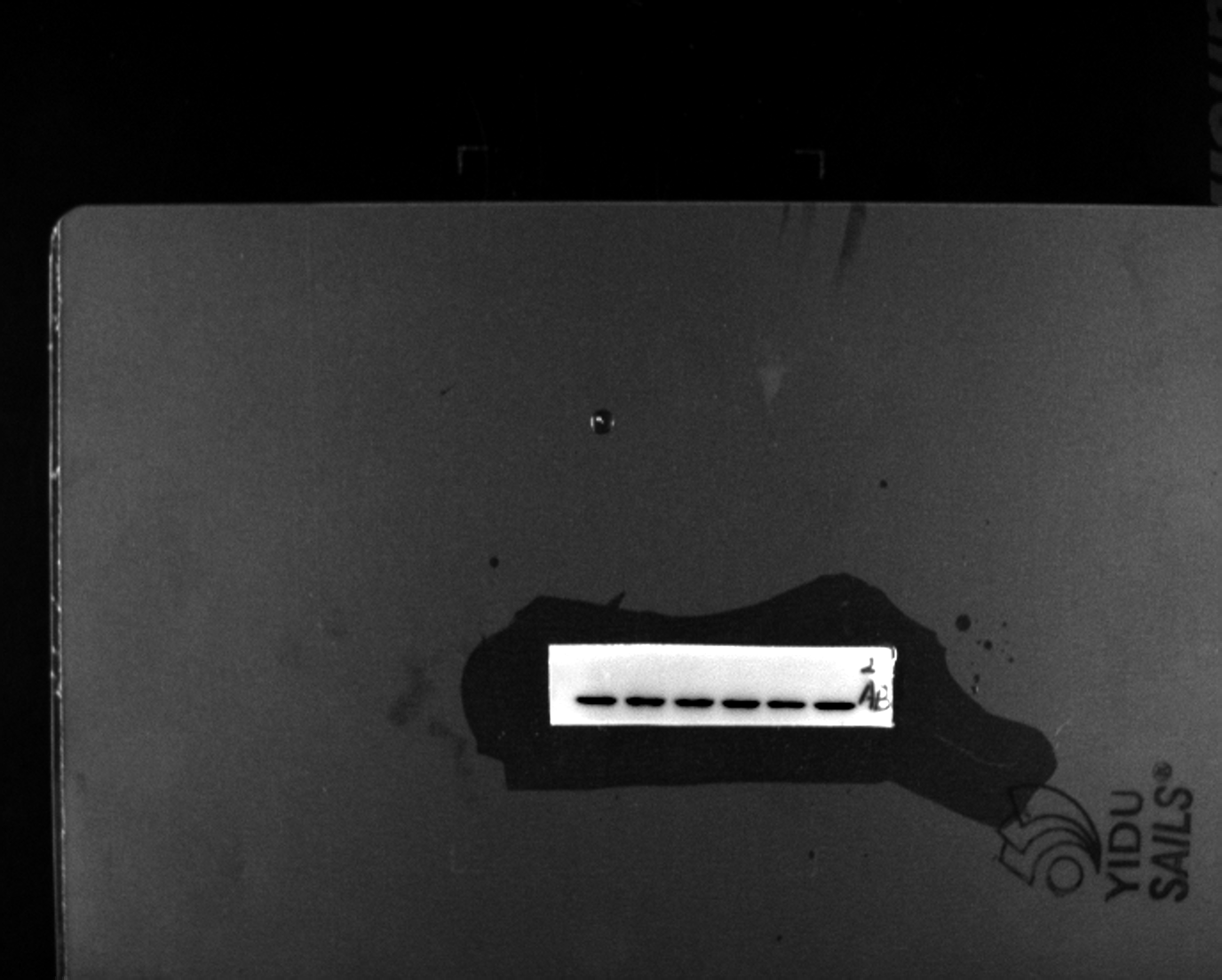

Supplement: Supplementary file 9 — Source Data Fig. 8 [file 44321_2024_25_MOESM9_ESM.zip › figure 8/8I/8I bactin.Tif]

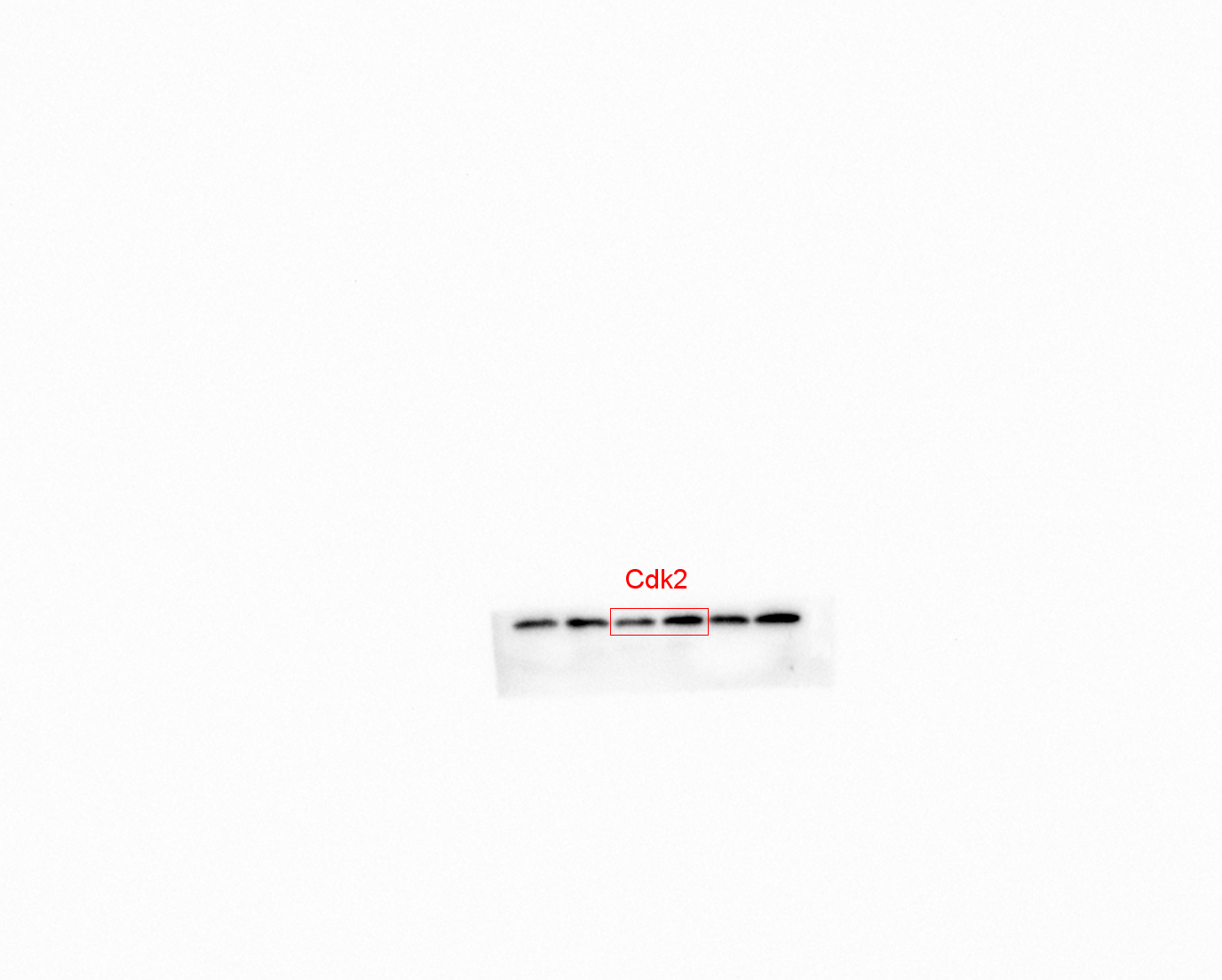

Supplement: Supplementary file 9 — Source Data Fig. 8 [file 44321_2024_25_MOESM9_ESM.zip › figure 8/8I/8I CDK2 mark.Tif]

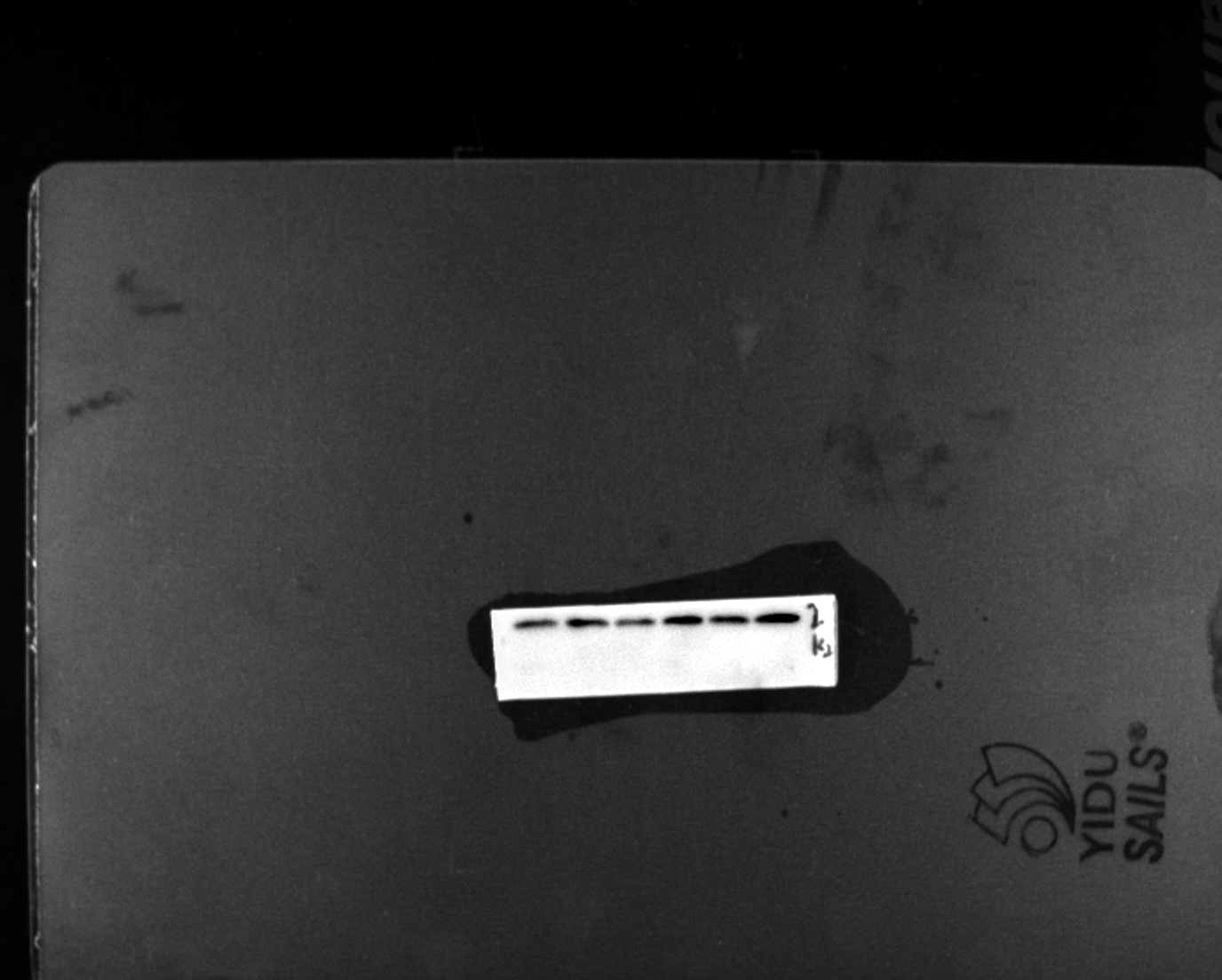

Supplement: Supplementary file 9 — Source Data Fig. 8 [file 44321_2024_25_MOESM9_ESM.zip › figure 8/8I/8I CDK2.Tif]

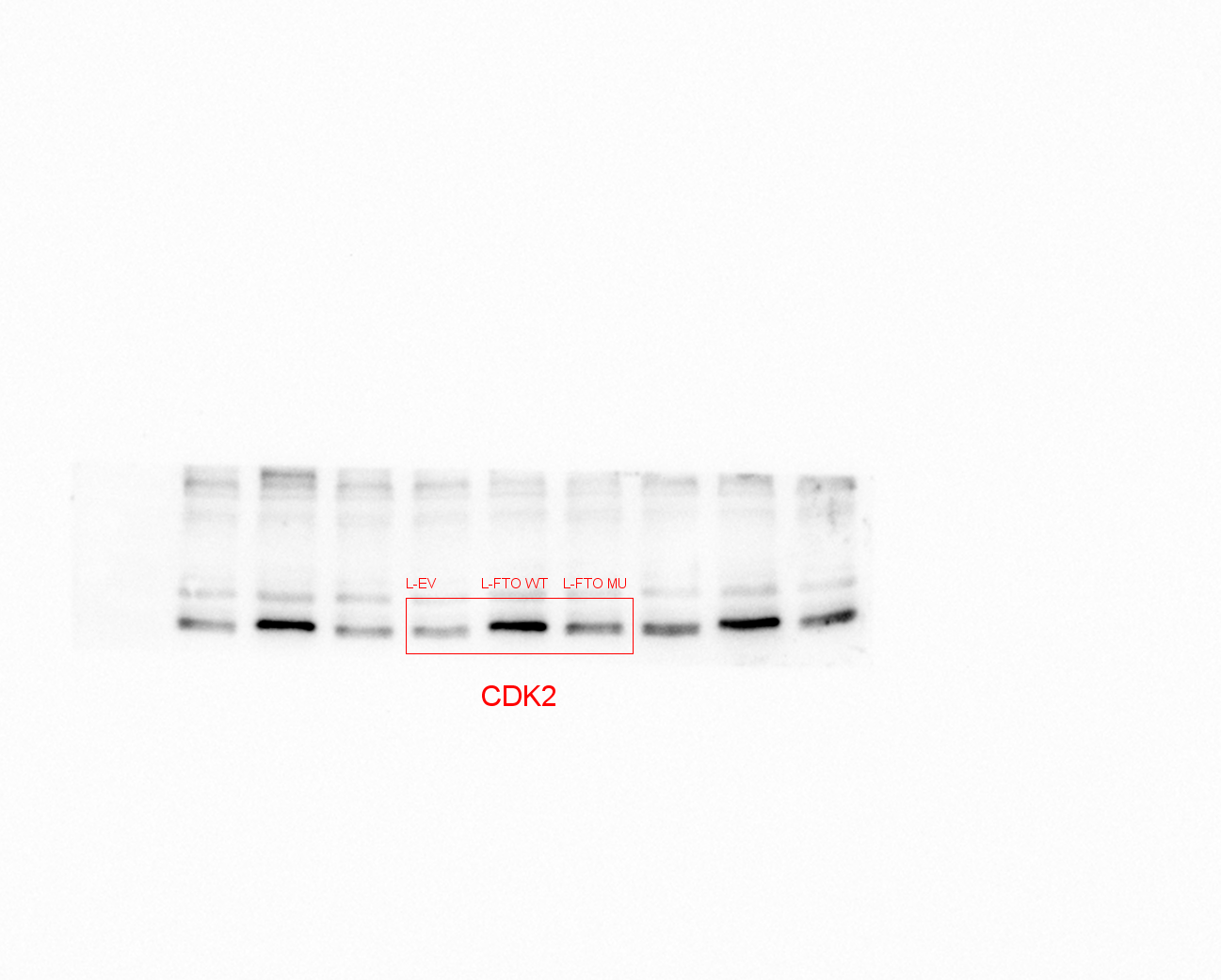

Supplement: Supplementary file 9 — Source Data Fig. 8 [file 44321_2024_25_MOESM9_ESM.zip › figure 8/8N/8N CDK2 mark.Tif]

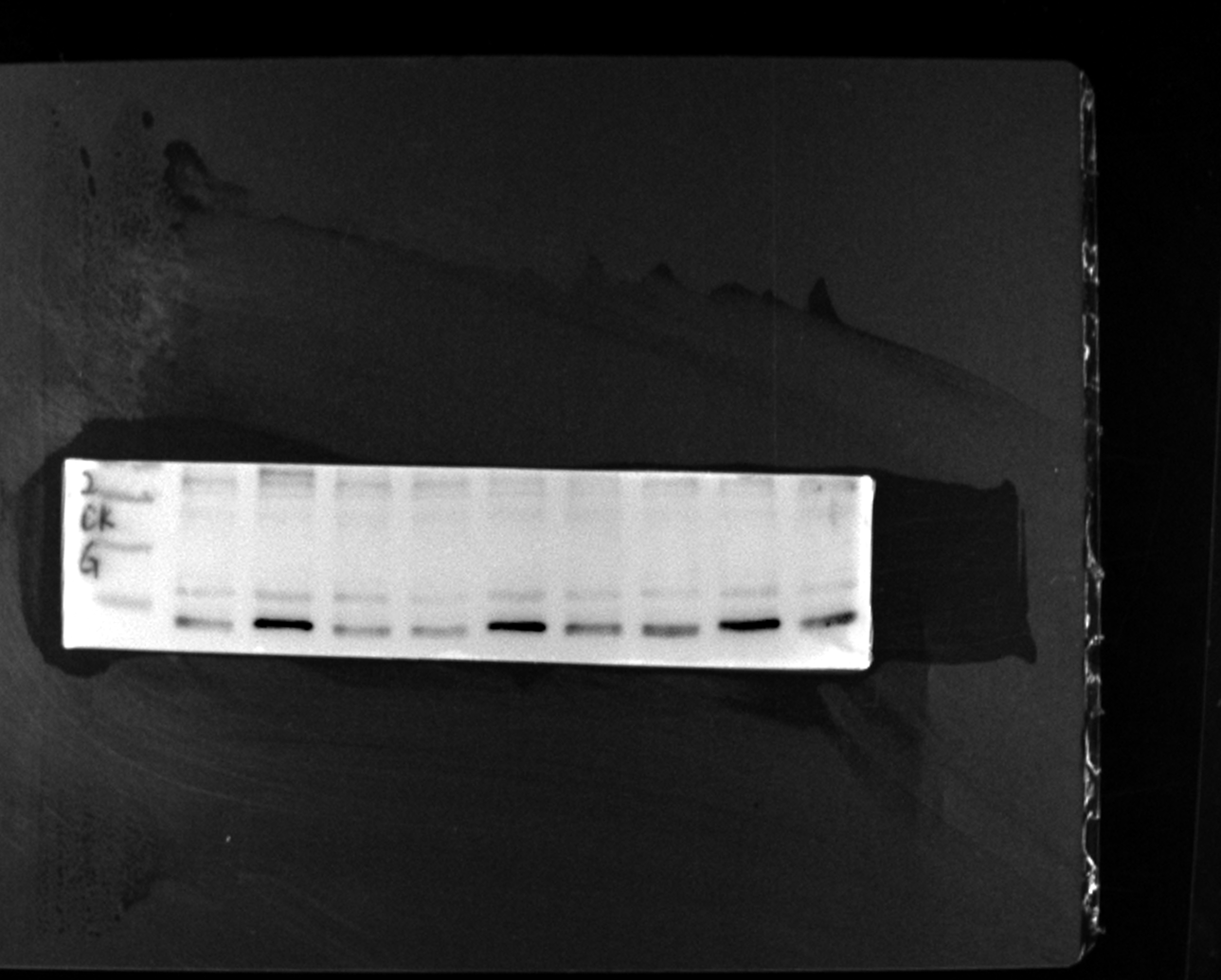

Supplement: Supplementary file 9 — Source Data Fig. 8 [file 44321_2024_25_MOESM9_ESM.zip › figure 8/8N/8N CDK2.Tif]

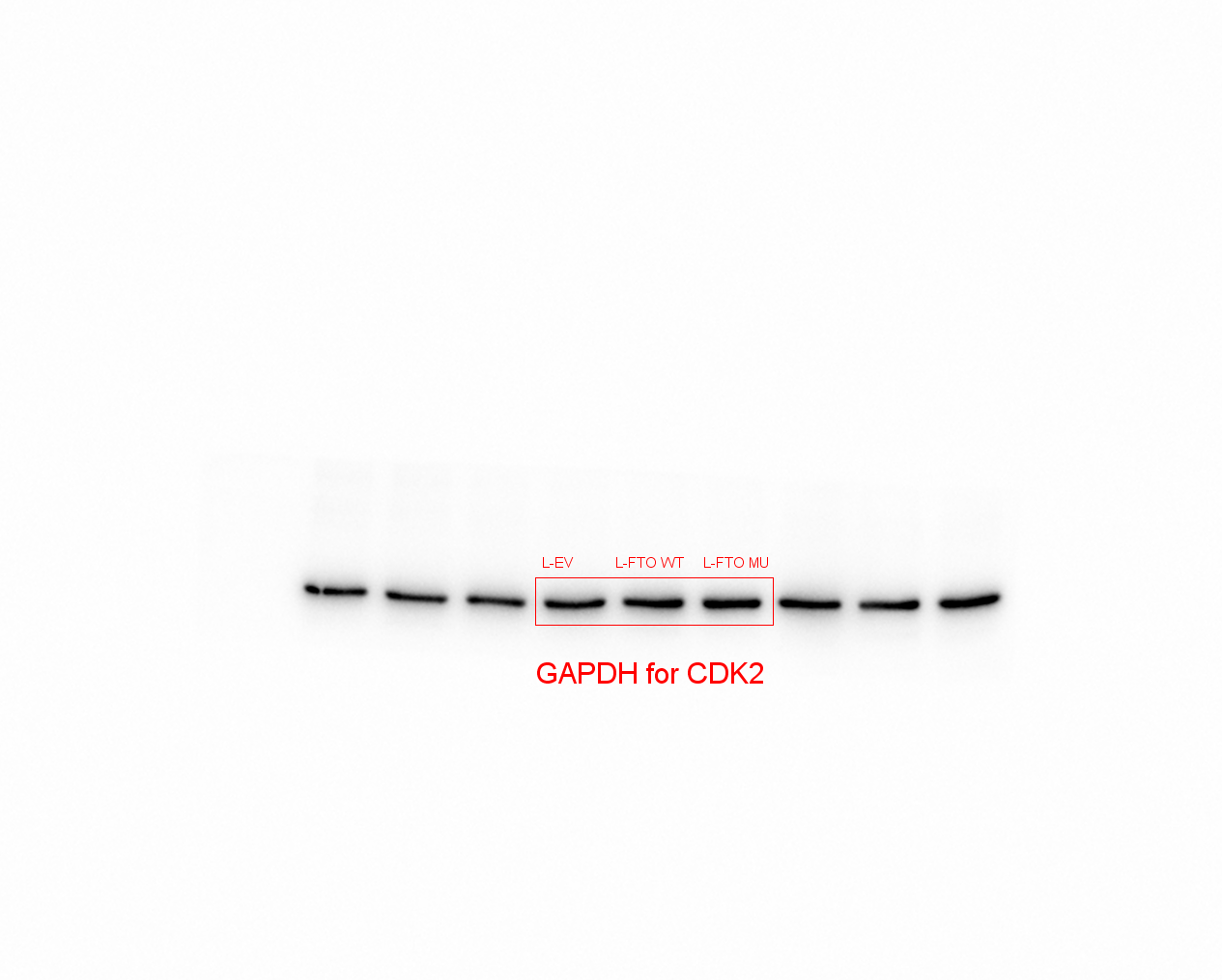

Supplement: Supplementary file 9 — Source Data Fig. 8 [file 44321_2024_25_MOESM9_ESM.zip › figure 8/8N/8N GAPDH for CDK2 mark.Tif]

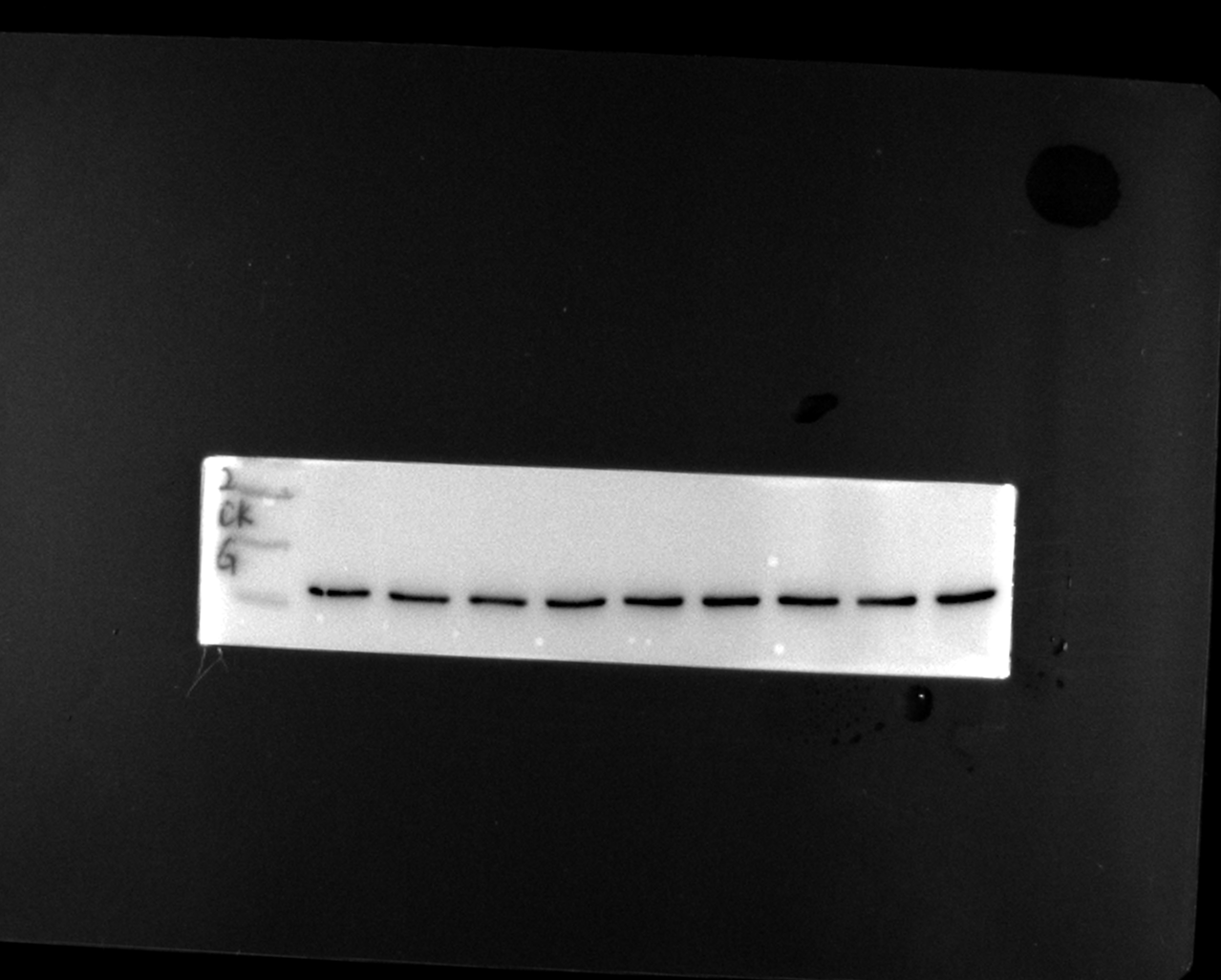

Supplement: Supplementary file 9 — Source Data Fig. 8 [file 44321_2024_25_MOESM9_ESM.zip › figure 8/8N/8N GAPDH for CDK2.Tif]

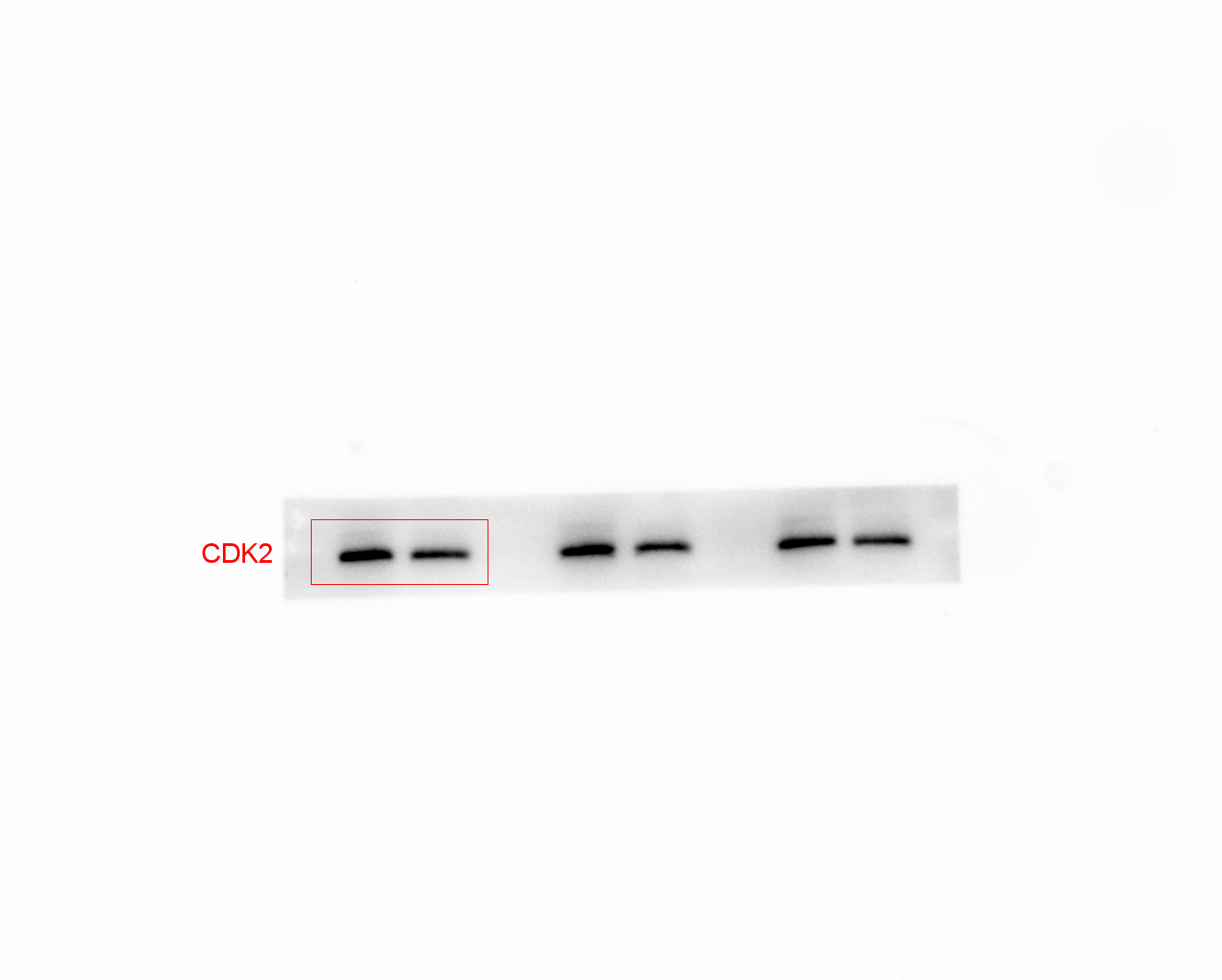

Supplement: Supplementary file 9 — Source Data Fig. 8 [file 44321_2024_25_MOESM9_ESM.zip › figure 8/8P/8P CDK2 mark.Tif]

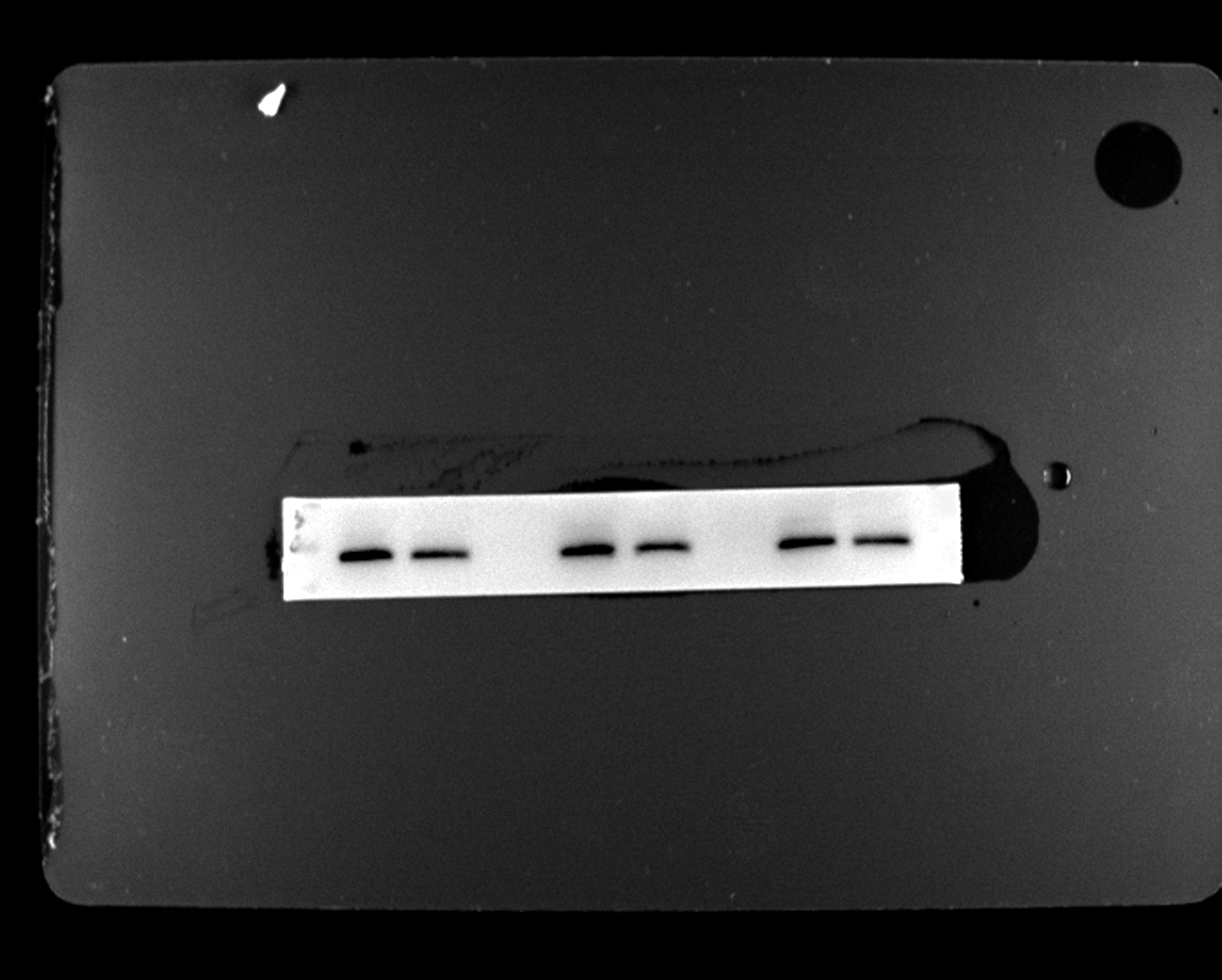

Supplement: Supplementary file 9 — Source Data Fig. 8 [file 44321_2024_25_MOESM9_ESM.zip › figure 8/8P/8P CDK2.Tif]

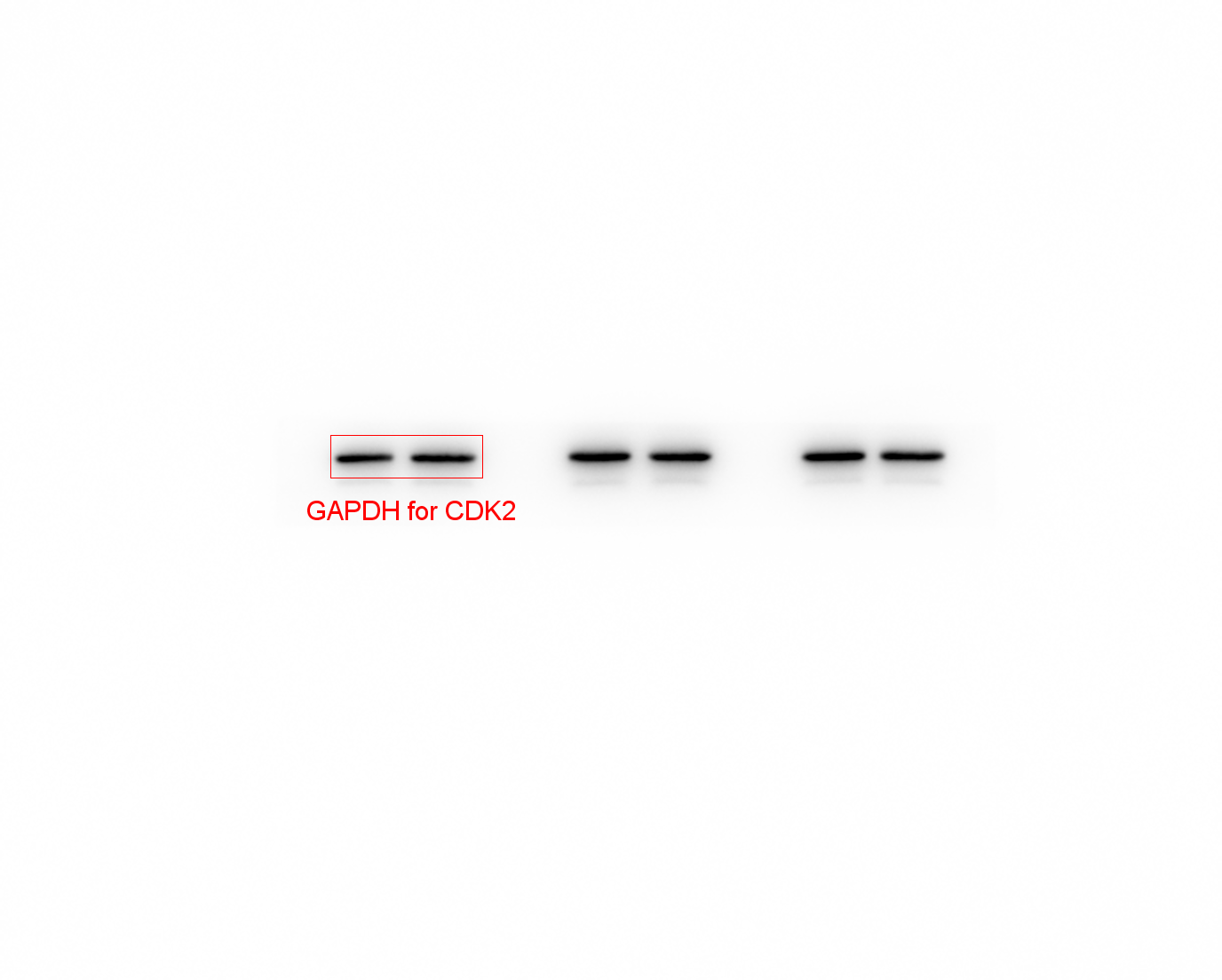

Supplement: Supplementary file 9 — Source Data Fig. 8 [file 44321_2024_25_MOESM9_ESM.zip › figure 8/8P/8P GAPDH mark.Tif]

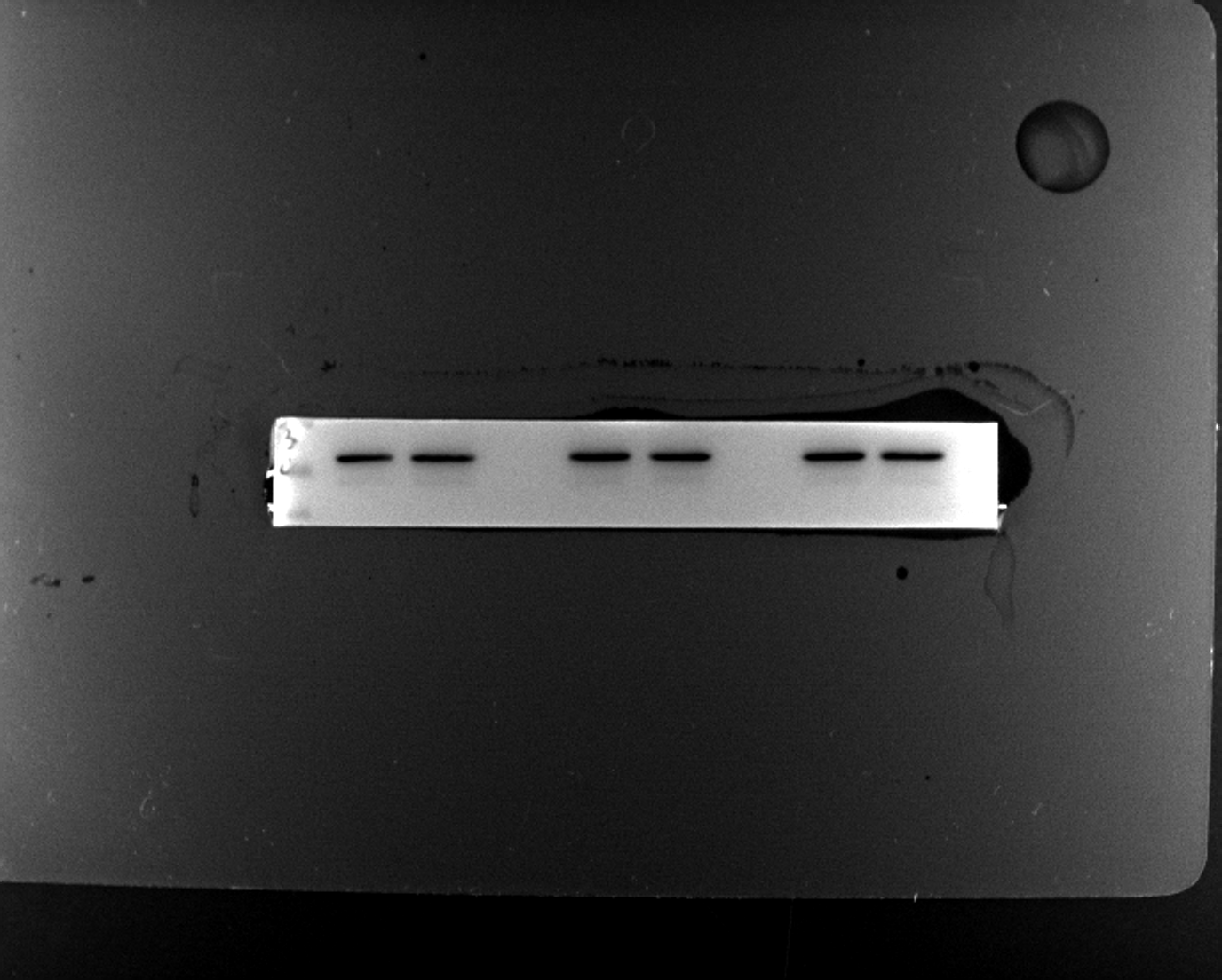

Supplement: Supplementary file 9 — Source Data Fig. 8 [file 44321_2024_25_MOESM9_ESM.zip › figure 8/8P/8P GAPDH.Tif]

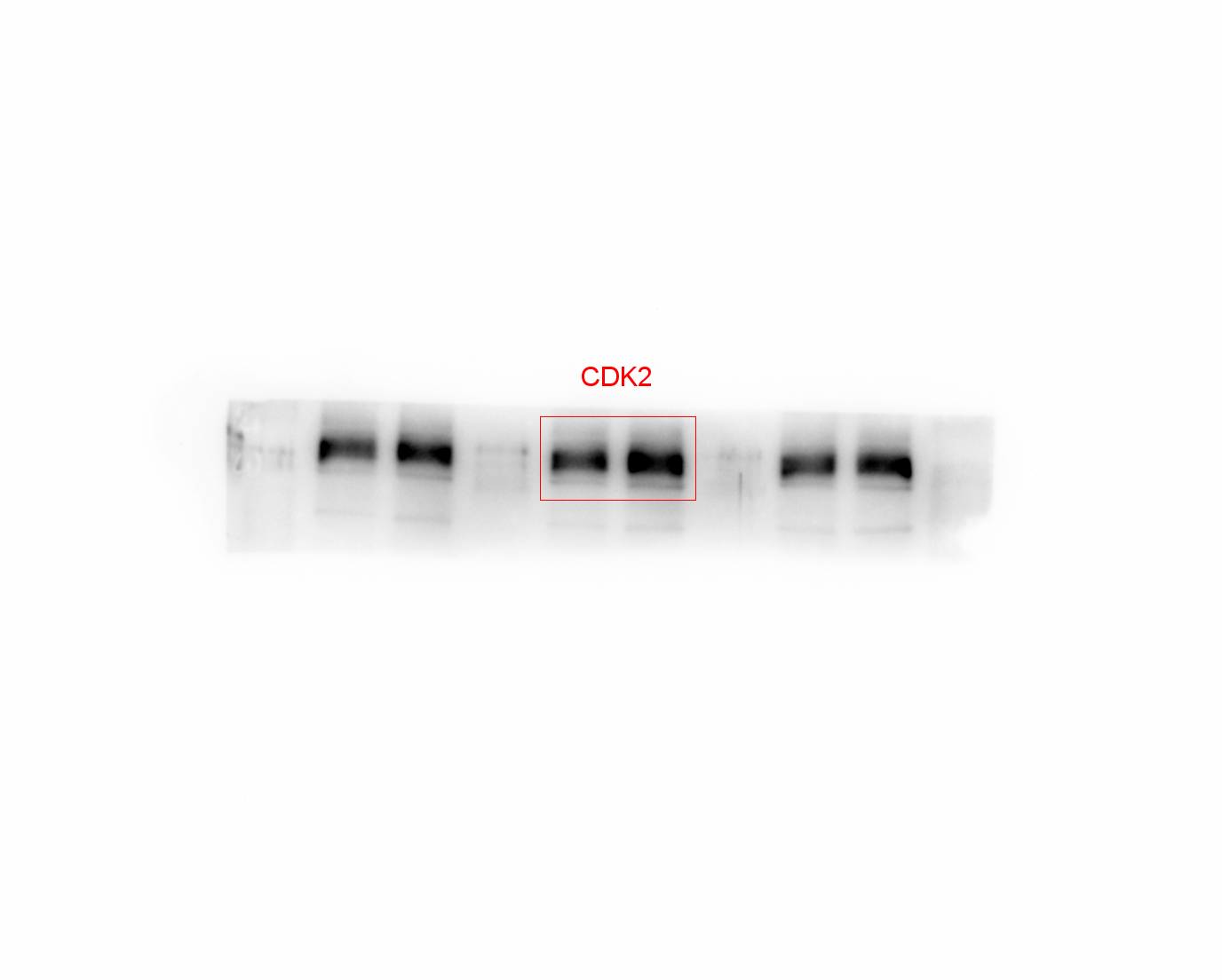

Supplement: Supplementary file 9 — Source Data Fig. 8 [file 44321_2024_25_MOESM9_ESM.zip › figure 8/8U/8U CDK2 mark.Tif]

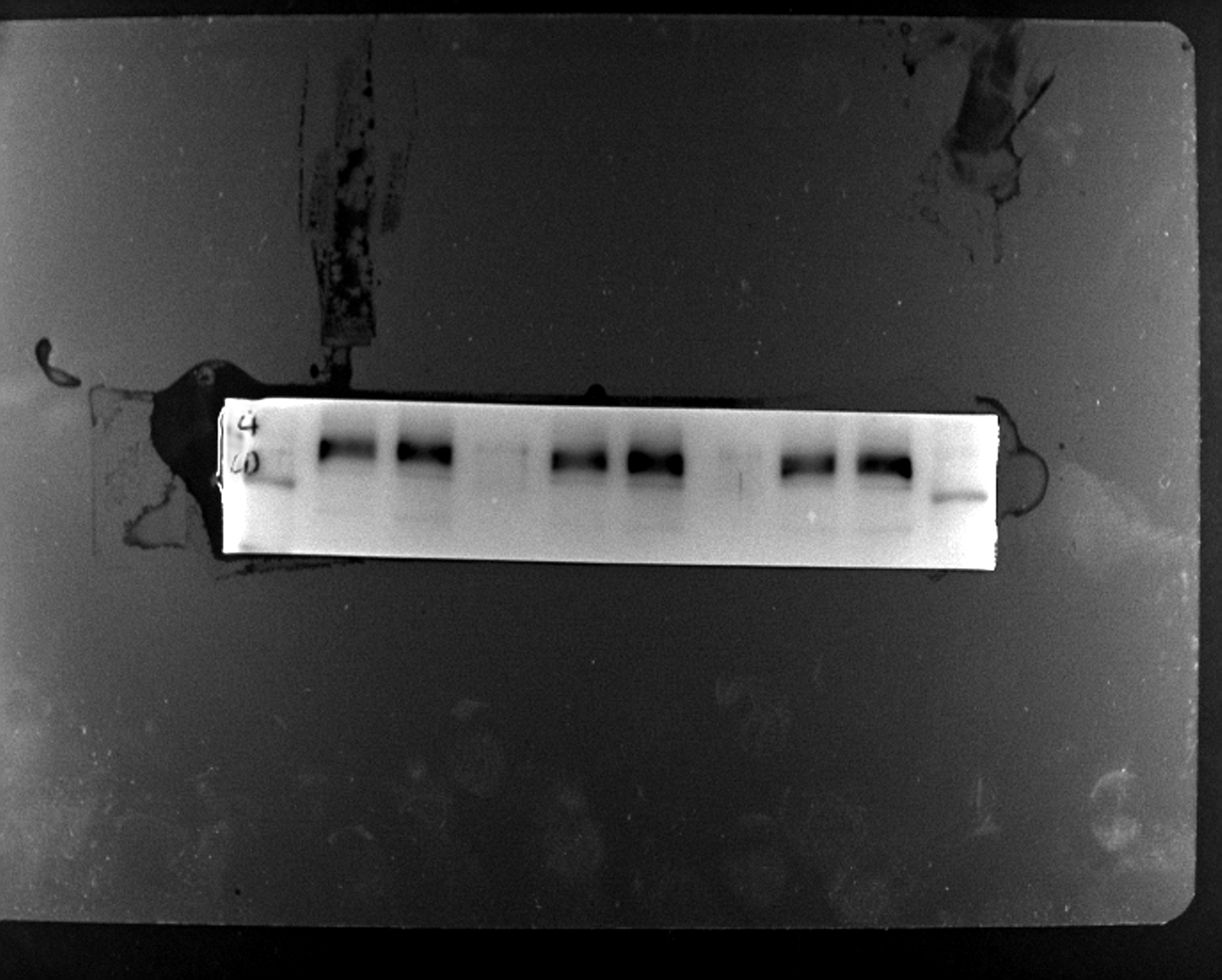

Supplement: Supplementary file 9 — Source Data Fig. 8 [file 44321_2024_25_MOESM9_ESM.zip › figure 8/8U/8U CDK2.Tif]

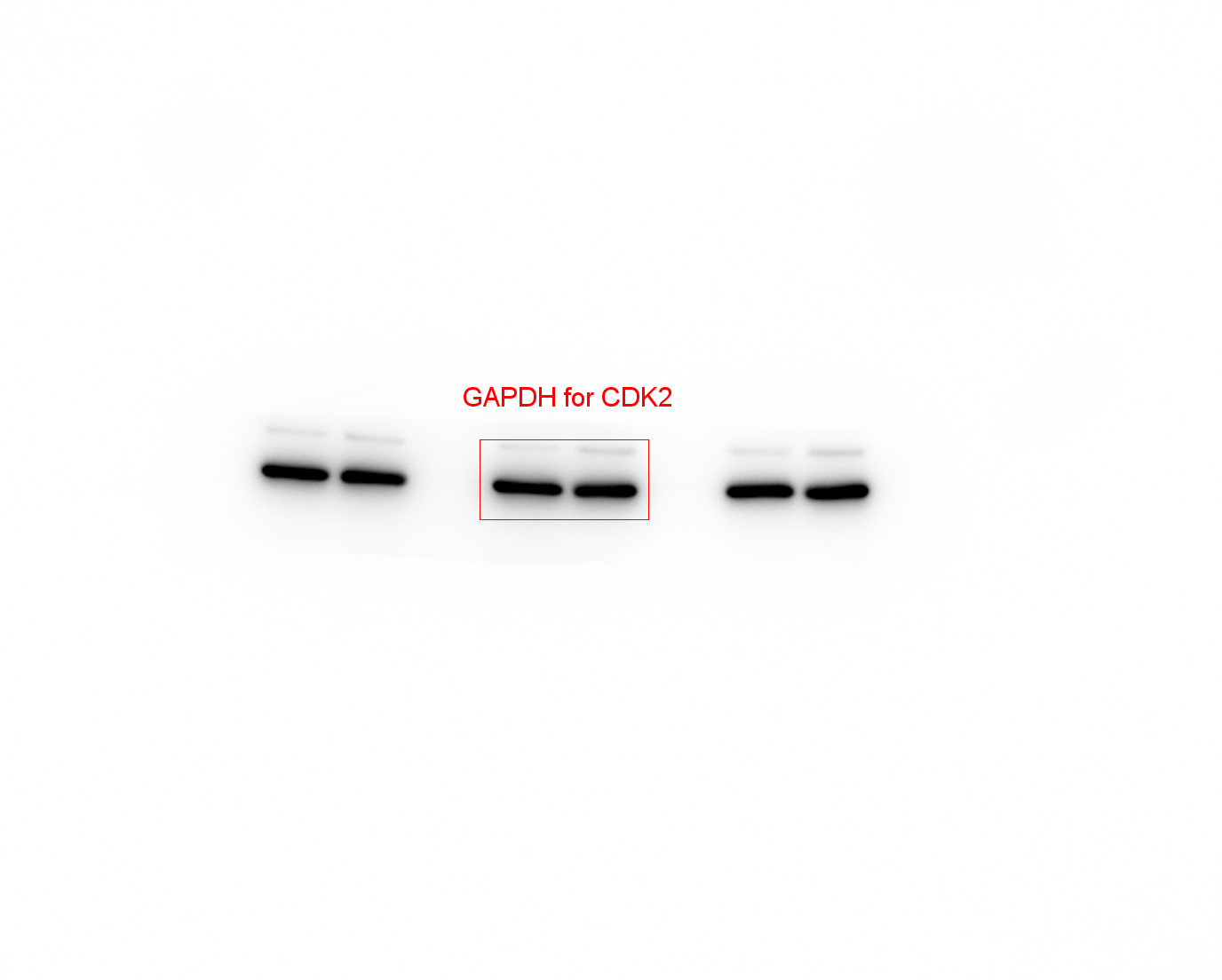

Supplement: Supplementary file 9 — Source Data Fig. 8 [file 44321_2024_25_MOESM9_ESM.zip › figure 8/8U/8U GAPDH mark.Tif]

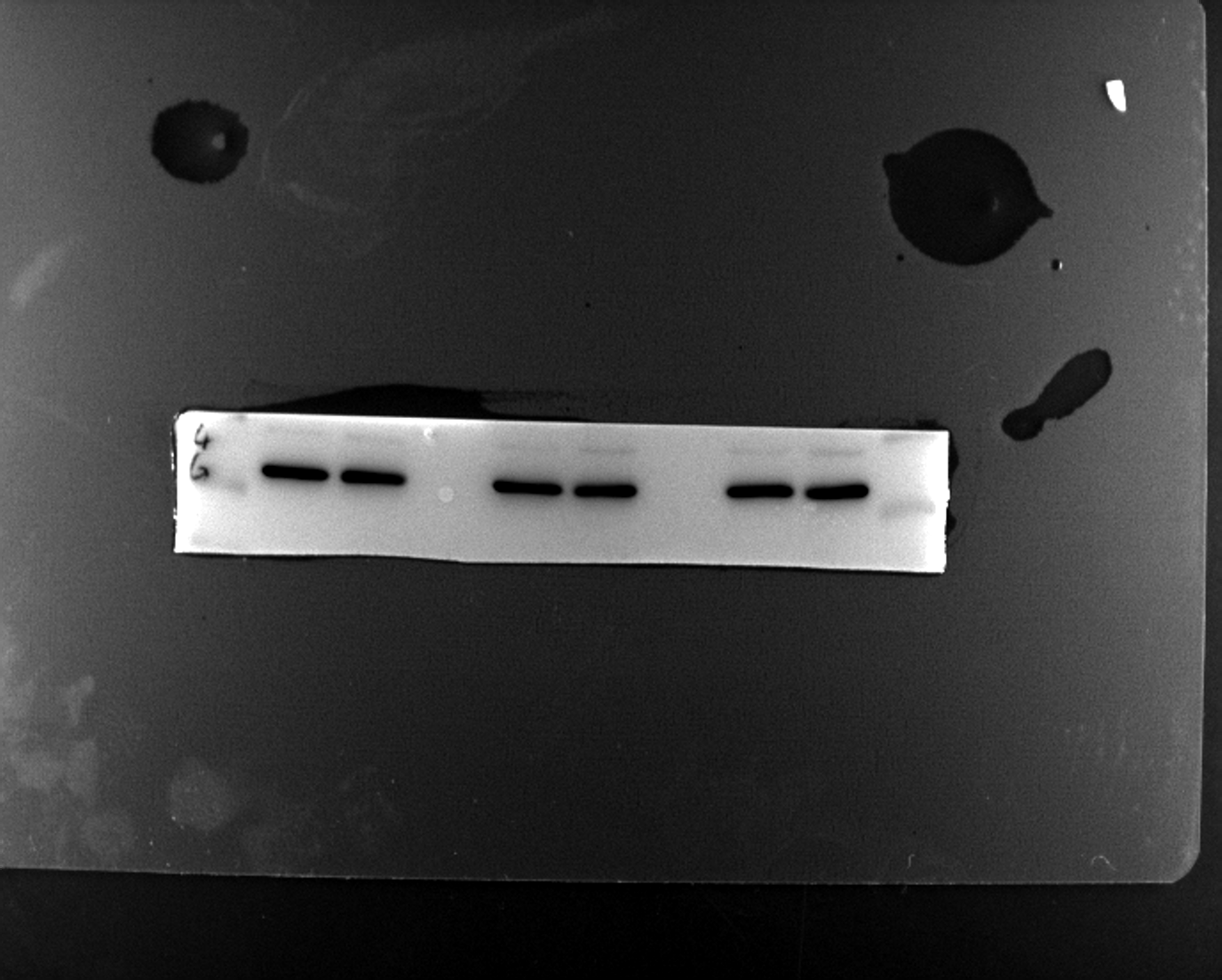

Supplement: Supplementary file 9 — Source Data Fig. 8 [file 44321_2024_25_MOESM9_ESM.zip › figure 8/8U/8U GAPDH.Tif]

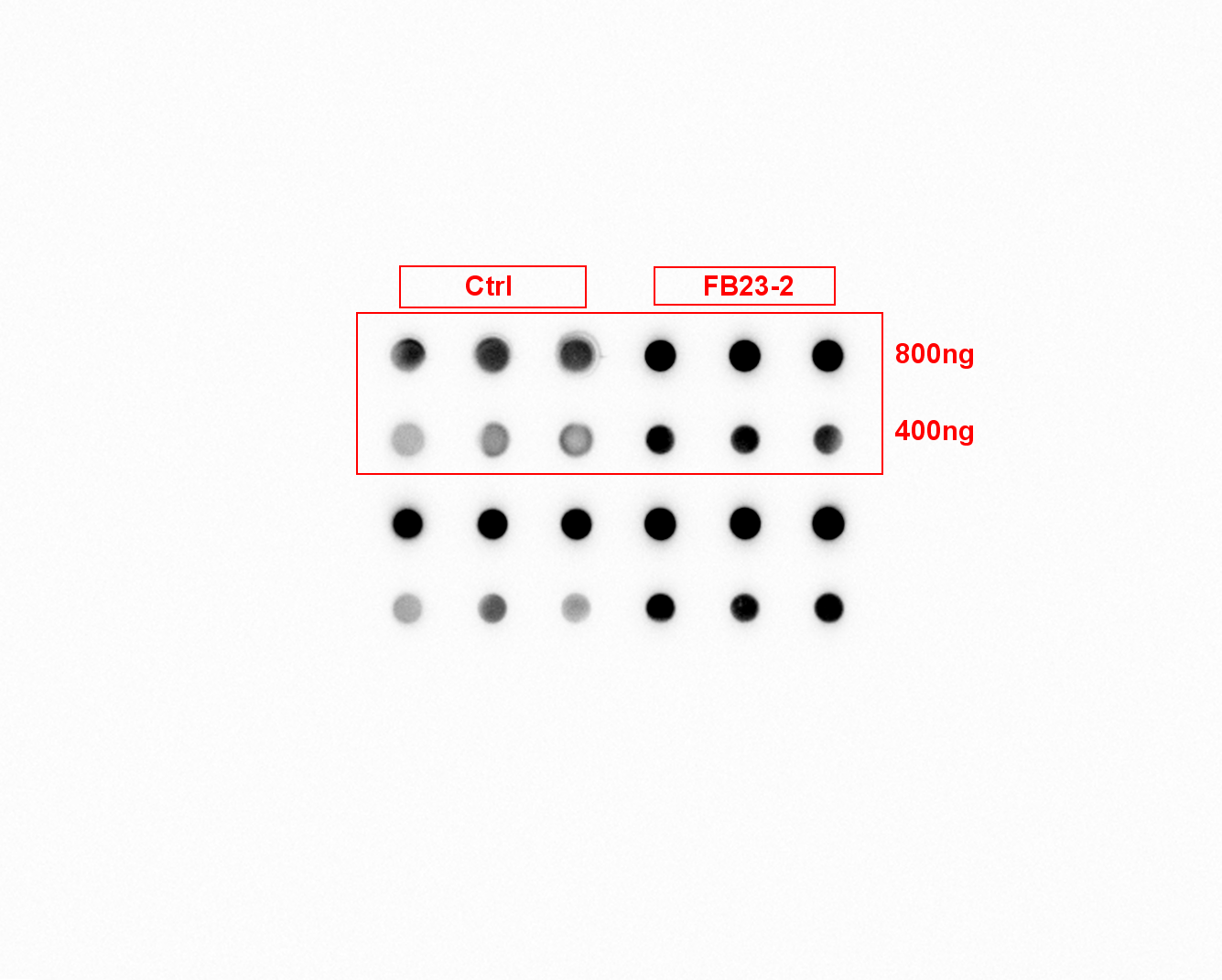

Supplement: Supplementary file 11 — Source Data Fig. 10 [file 44321_2024_25_MOESM11_ESM.zip › figure 10/10A/10A m6A mark.Tif]

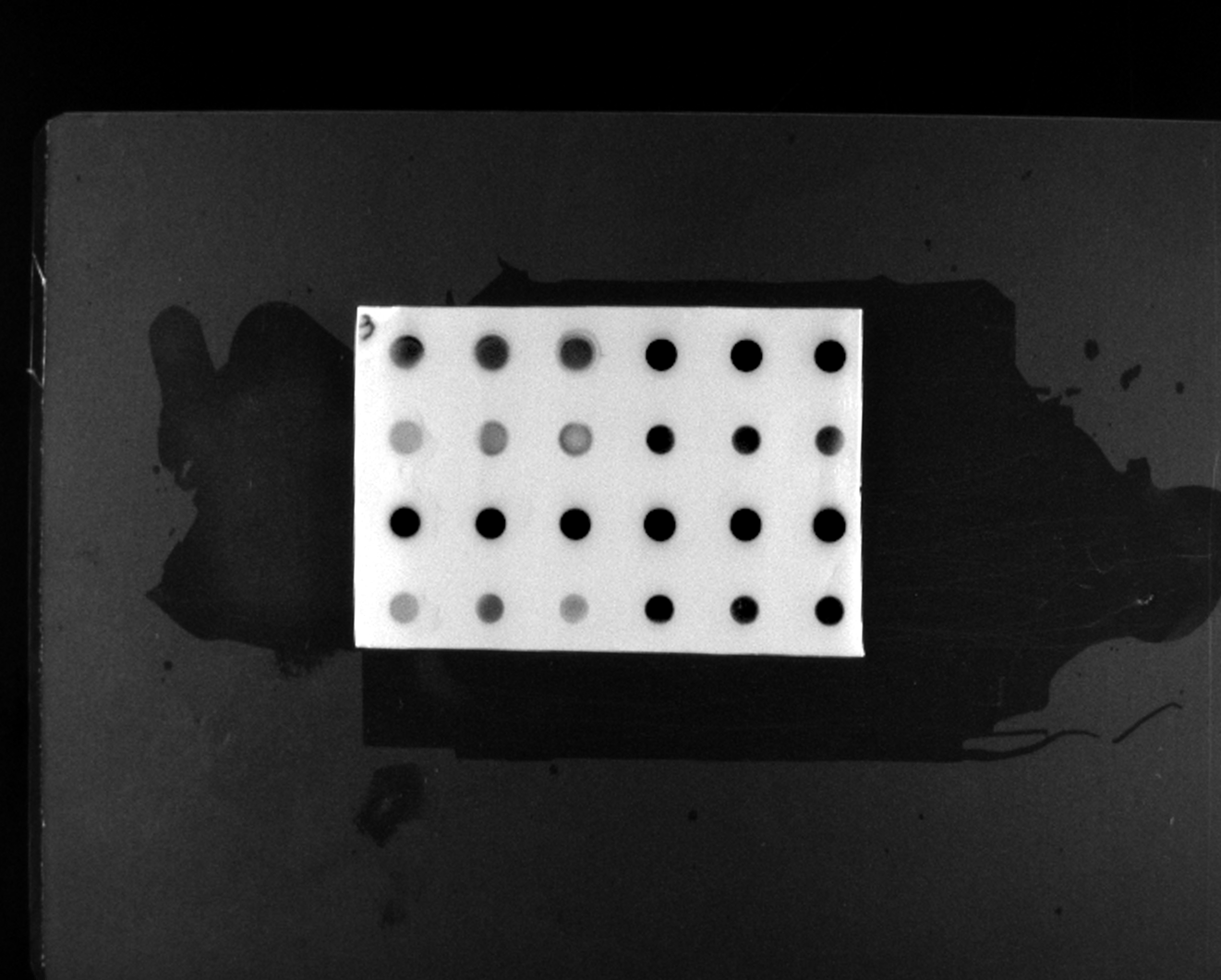

Supplement: Supplementary file 11 — Source Data Fig. 10 [file 44321_2024_25_MOESM11_ESM.zip › figure 10/10A/10A m6A.Tif]

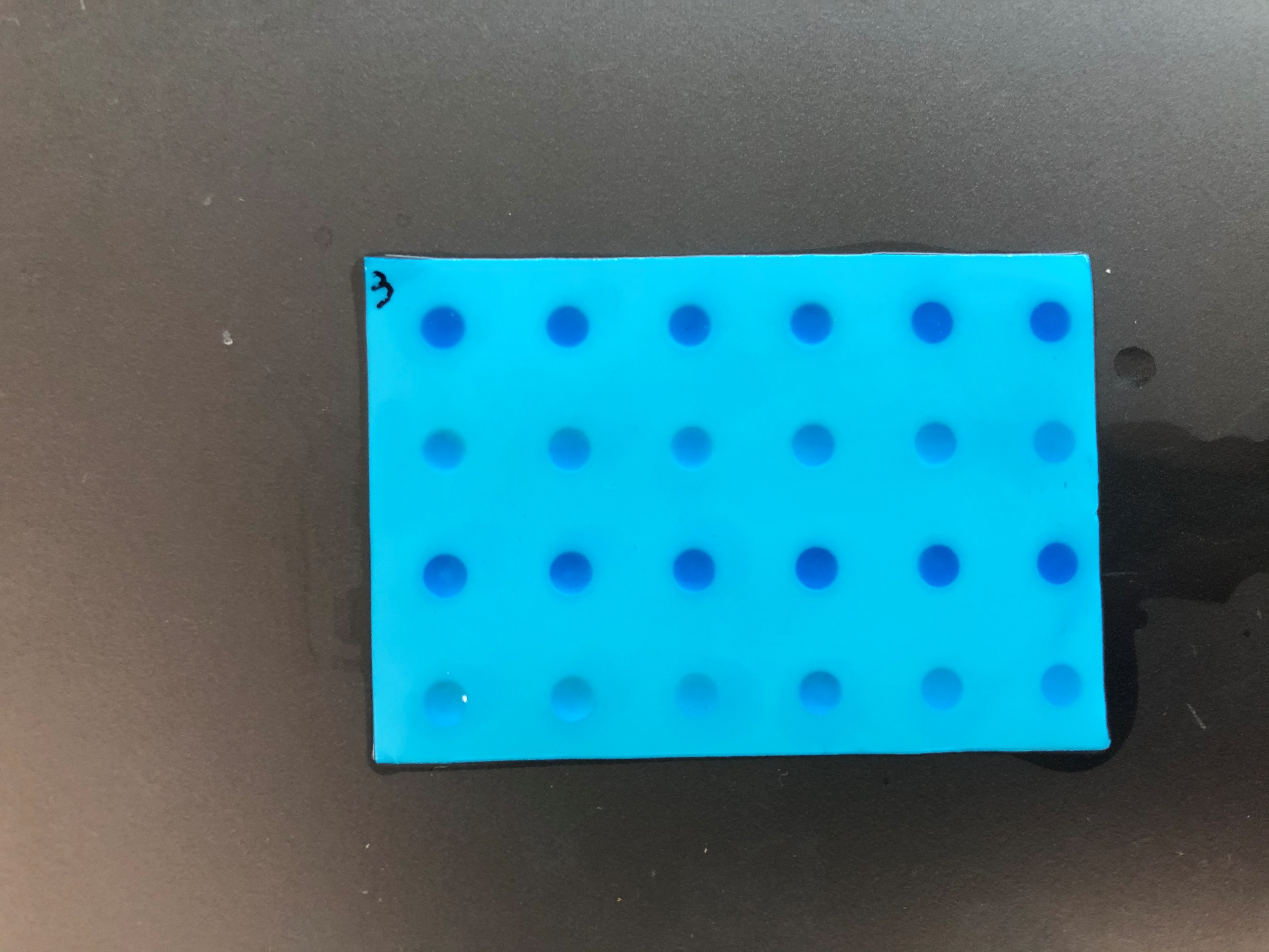

Supplement: Supplementary file 11 — Source Data Fig. 10 [file 44321_2024_25_MOESM11_ESM.zip › figure 10/10A/10A MB.tif]

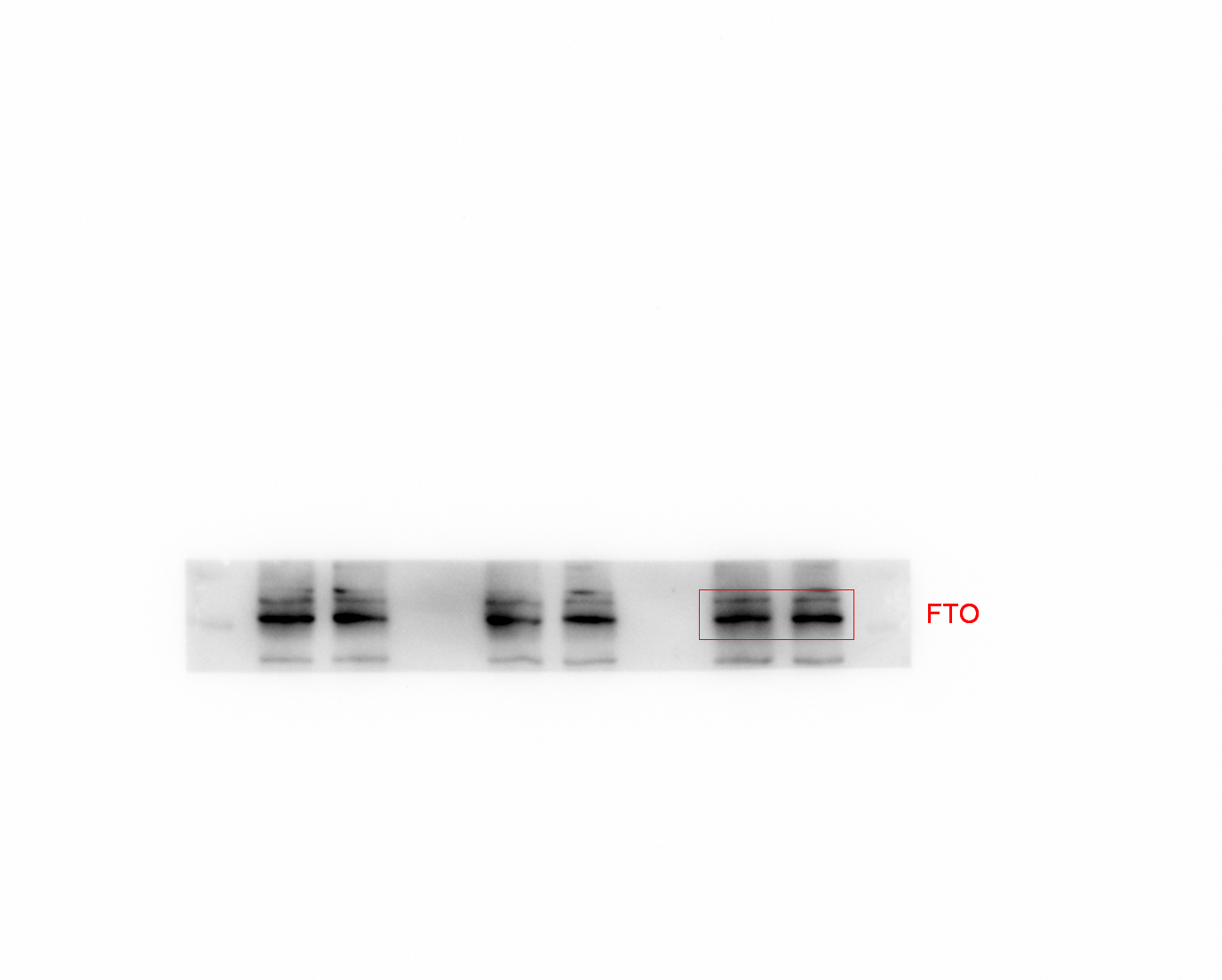

Supplement: Supplementary file 11 — Source Data Fig. 10 [file 44321_2024_25_MOESM11_ESM.zip › figure 10/10B/10B FTO mark.Tif]

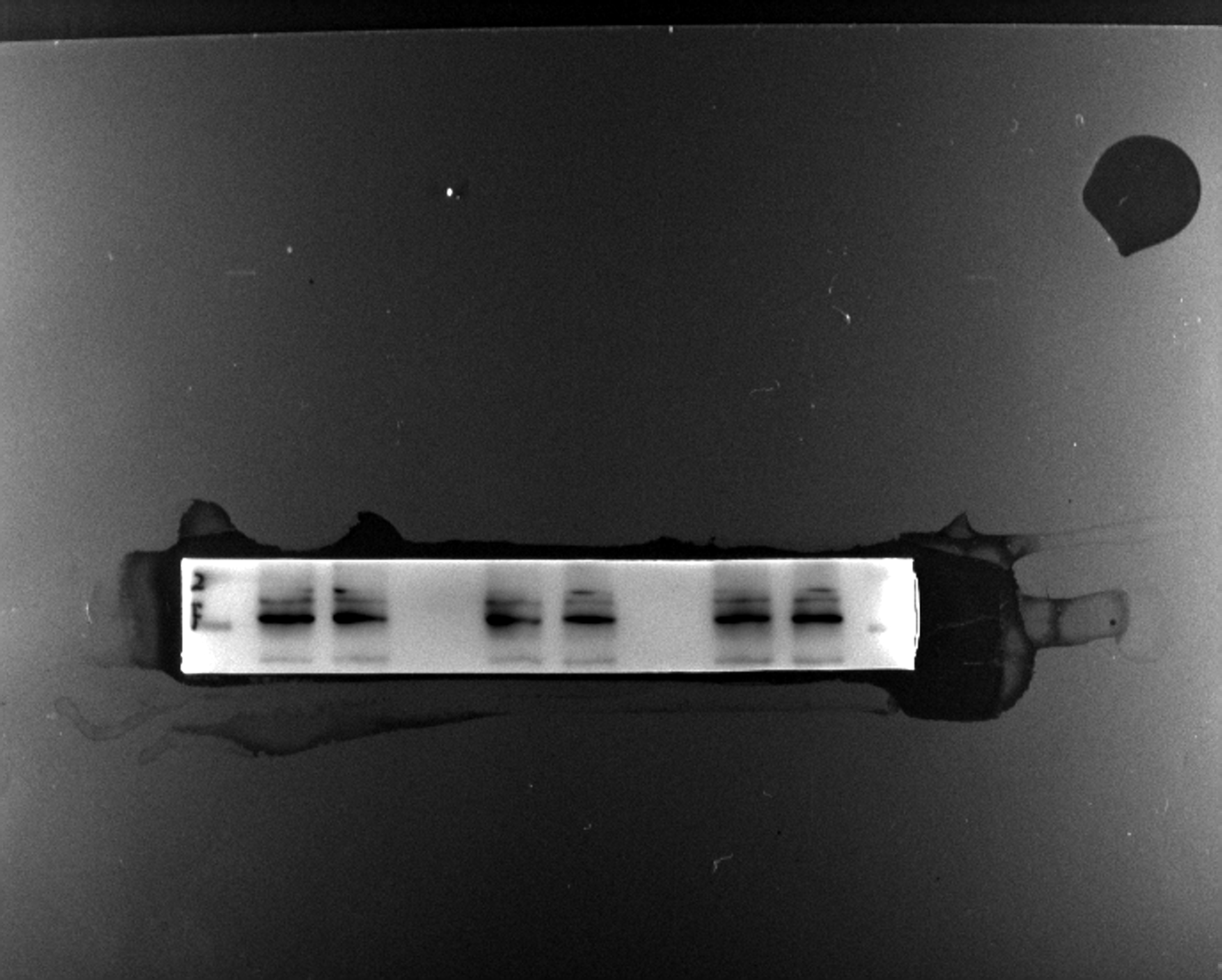

Supplement: Supplementary file 11 — Source Data Fig. 10 [file 44321_2024_25_MOESM11_ESM.zip › figure 10/10B/10B FTO.Tif]

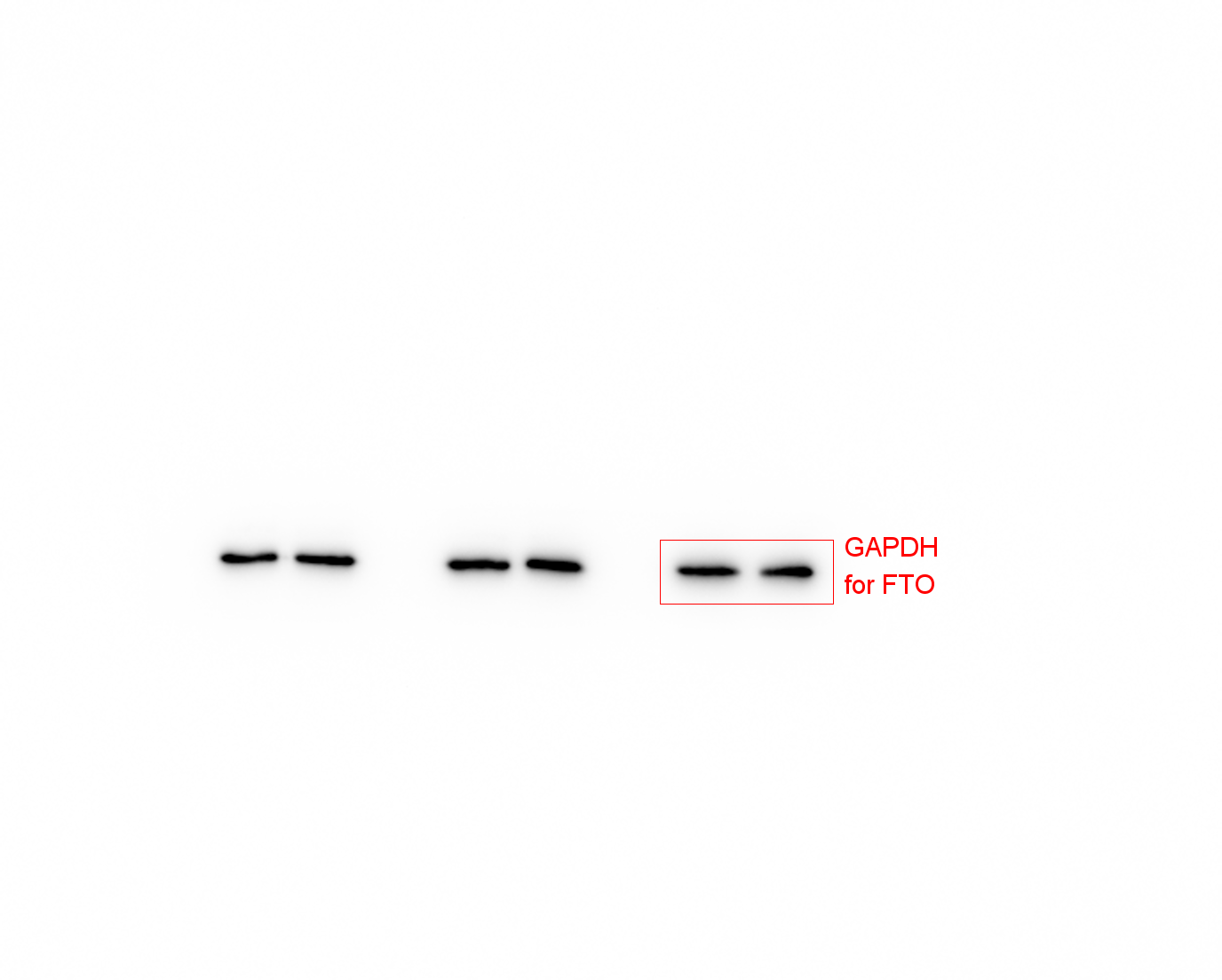

Supplement: Supplementary file 11 — Source Data Fig. 10 [file 44321_2024_25_MOESM11_ESM.zip › figure 10/10B/10B GAPDH for FTO mark.Tif]

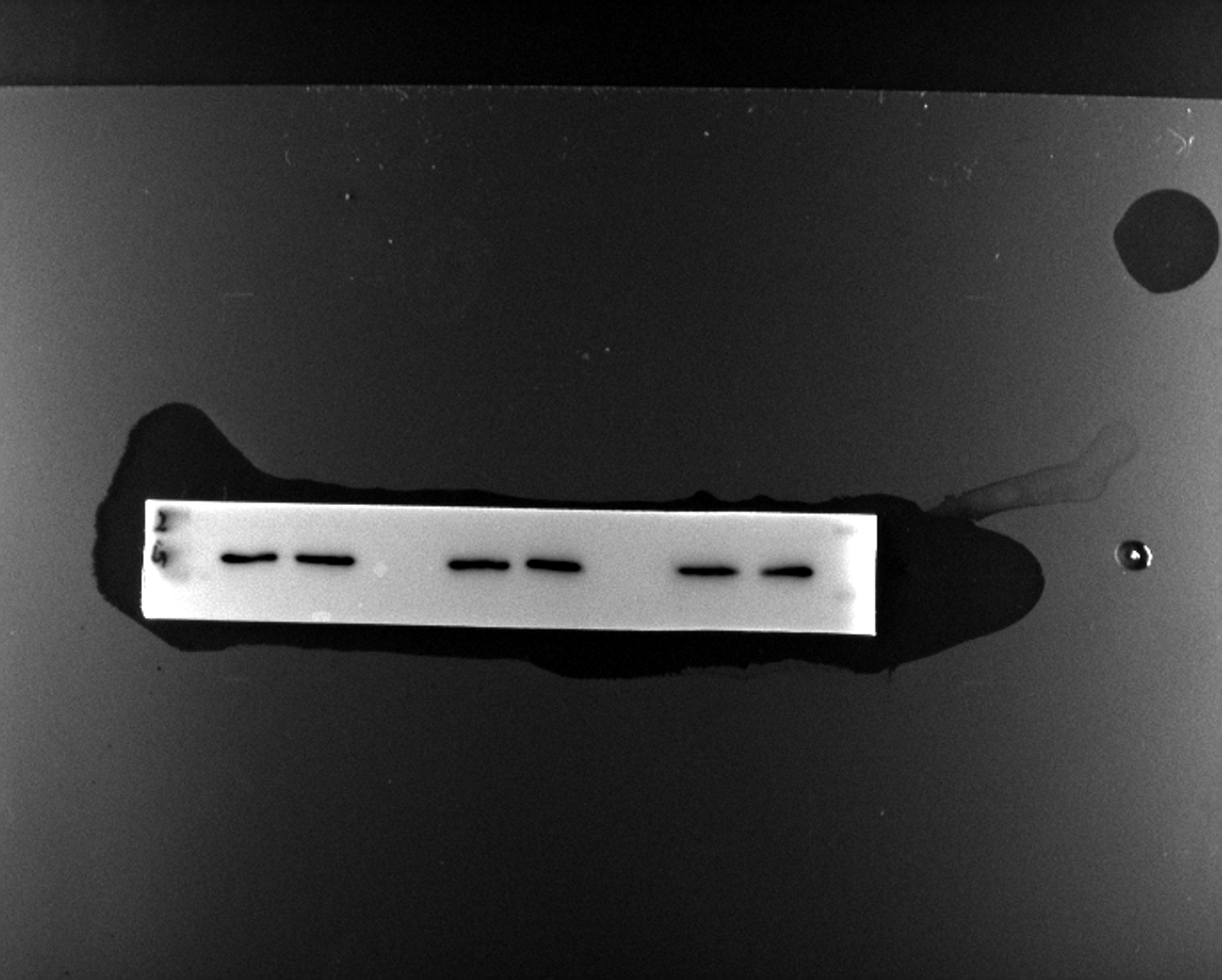

Supplement: Supplementary file 11 — Source Data Fig. 10 [file 44321_2024_25_MOESM11_ESM.zip › figure 10/10B/10B GAPDH for FTO.Tif]
