# Supplementary figures and images for: Lactylation-driven FTO targets CDK2 to aggravate microvascular anomalies in diabetic retinopathy (part 4 of 4)
Source: EMBO Mol Med. 2024 Jan 31;16(2):294–318. doi: 10.1038/s44321-024-00025-1 (PMC10897304; doi:10.1038/s44321-024-00025-1)

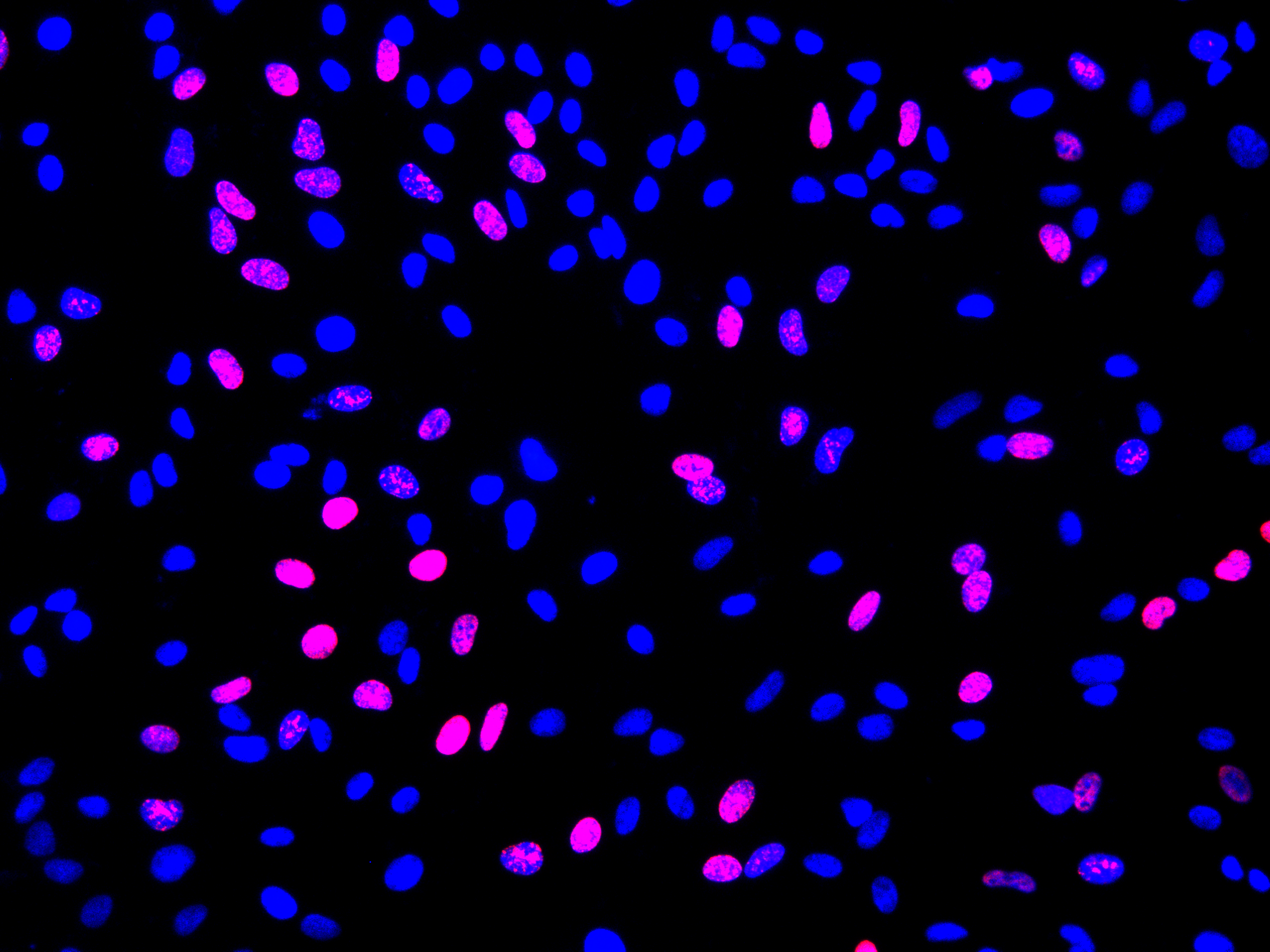

Supplement: Supplementary file 11 — Source Data Fig. 10 [file 44321_2024_25_MOESM11_ESM.zip › figure 10/10C/10C HG EdU DAPI.tif]

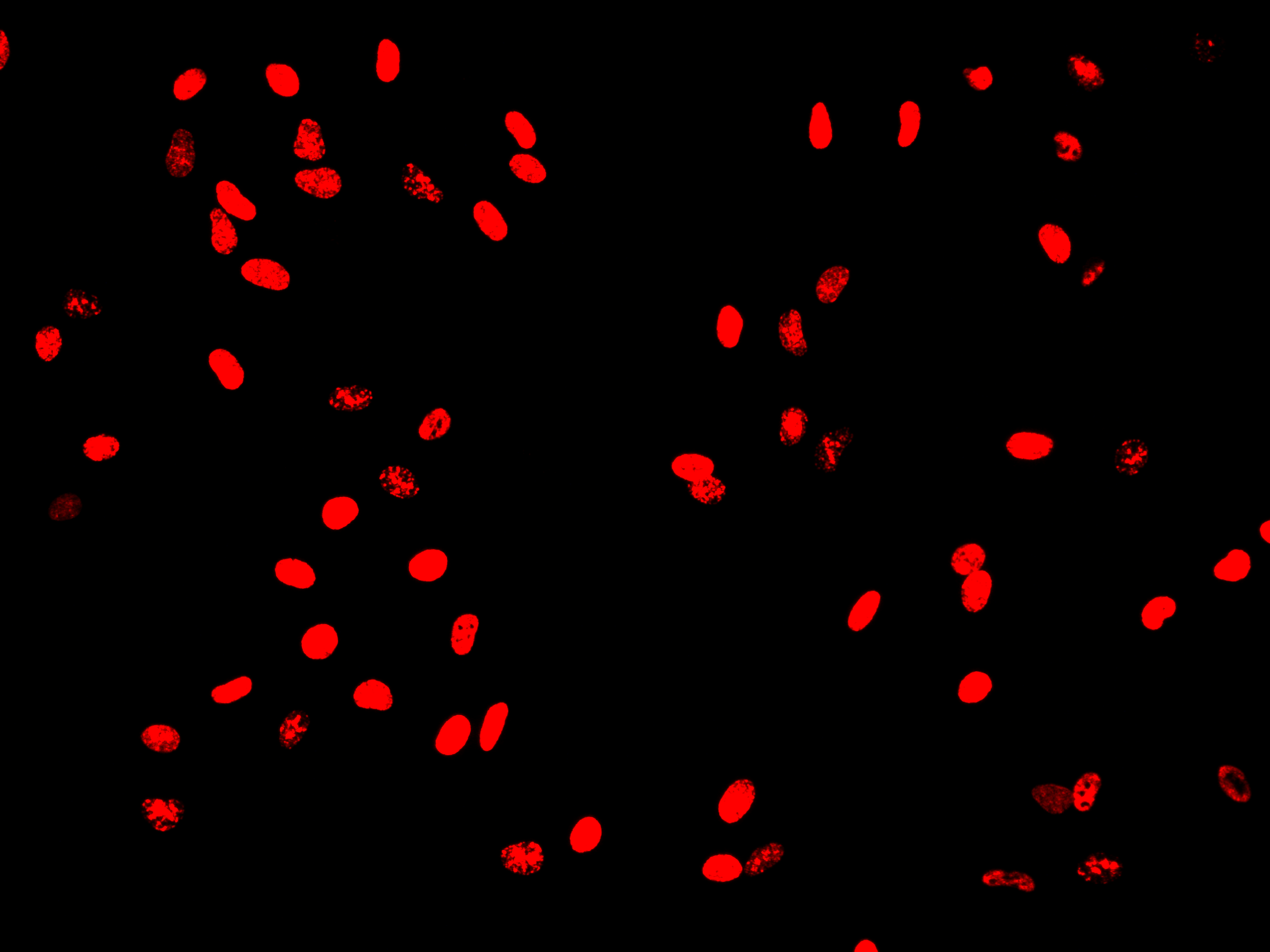

Supplement: Supplementary file 11 — Source Data Fig. 10 [file 44321_2024_25_MOESM11_ESM.zip › figure 10/10C/10C HG EdU.tif]

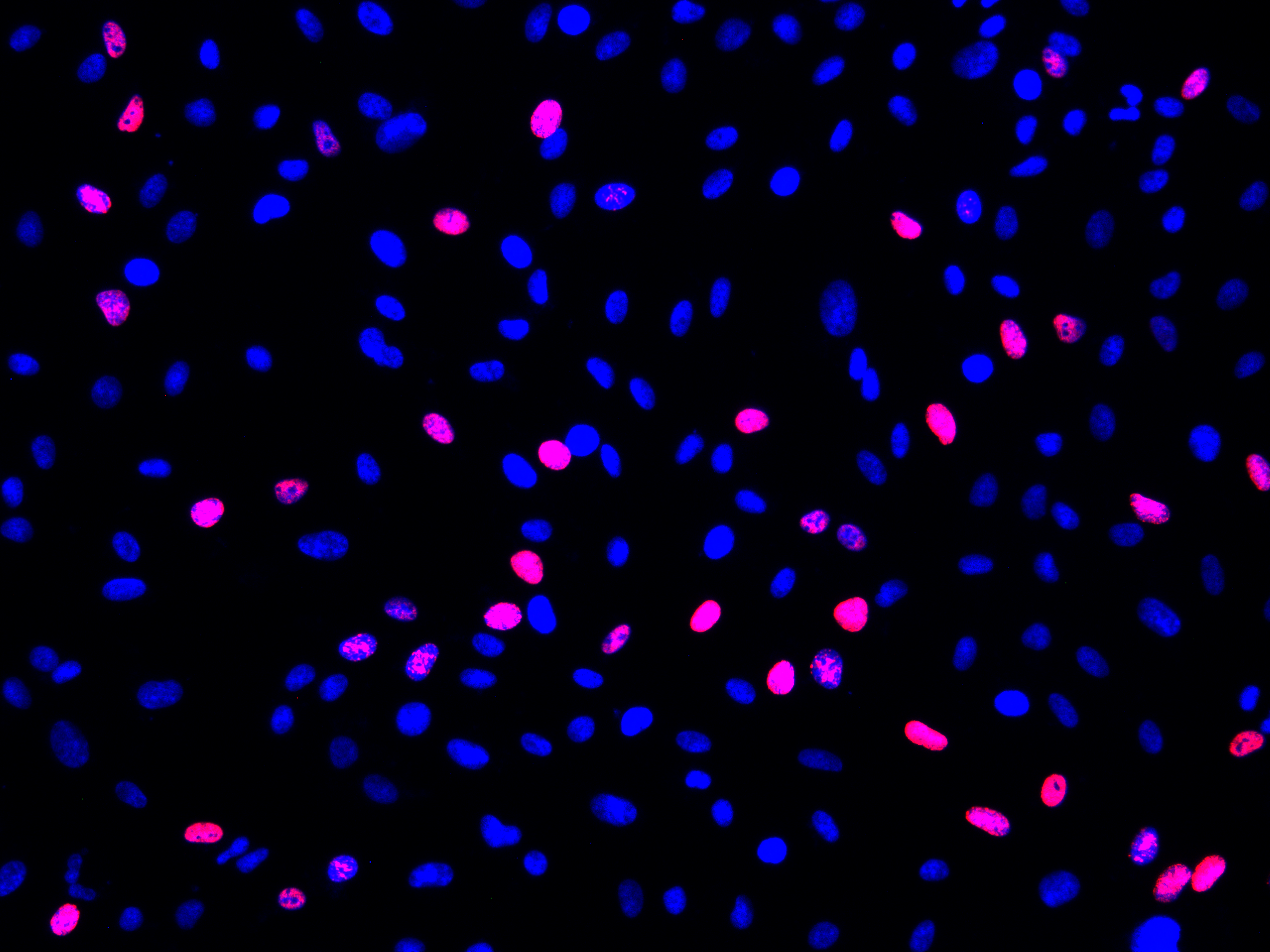

Supplement: Supplementary file 11 — Source Data Fig. 10 [file 44321_2024_25_MOESM11_ESM.zip › figure 10/10C/10C HG+FB23-2 EdU DAPI.tif]

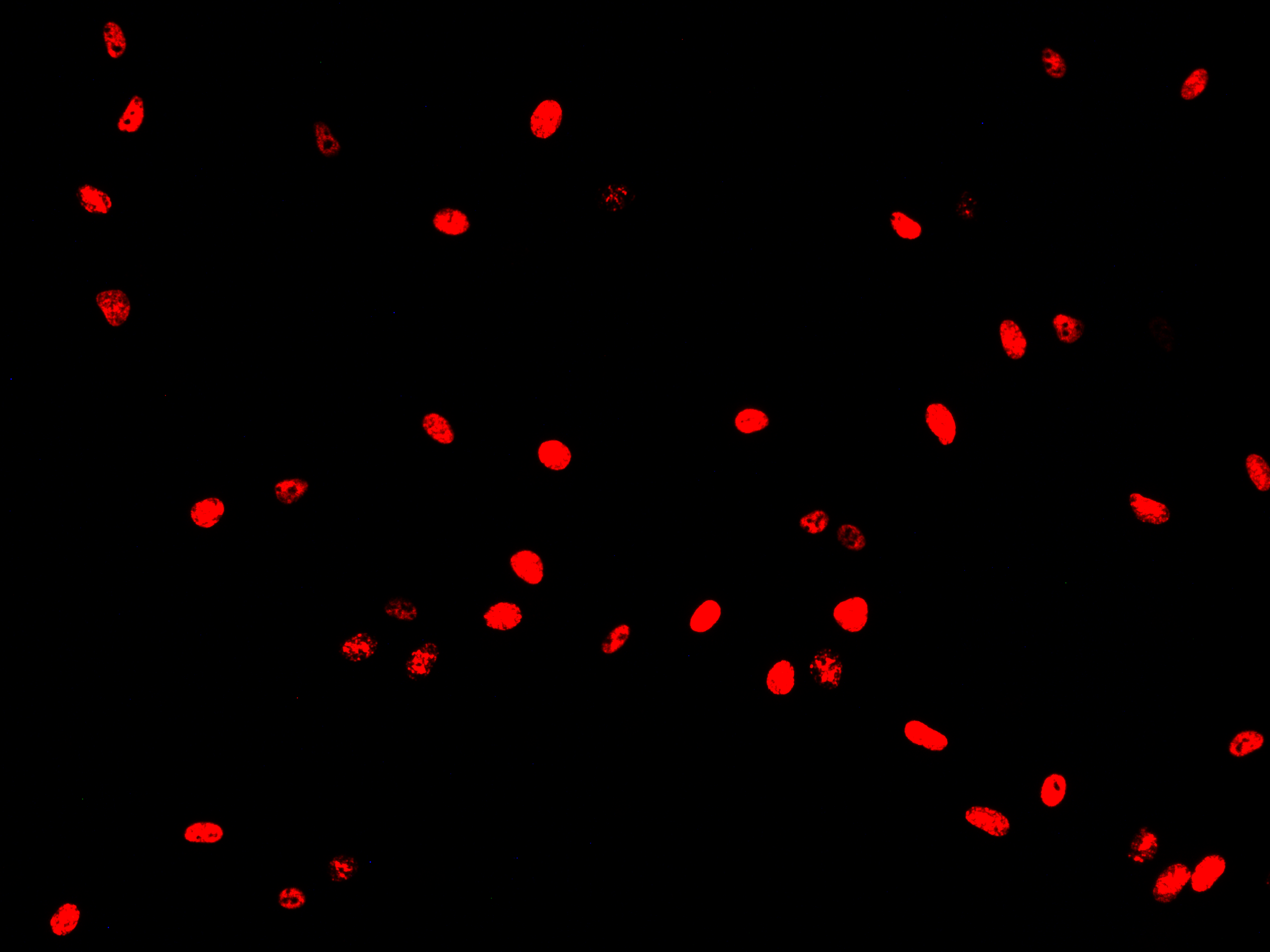

Supplement: Supplementary file 11 — Source Data Fig. 10 [file 44321_2024_25_MOESM11_ESM.zip › figure 10/10C/10C HG+FB23-2 EdU.tif]

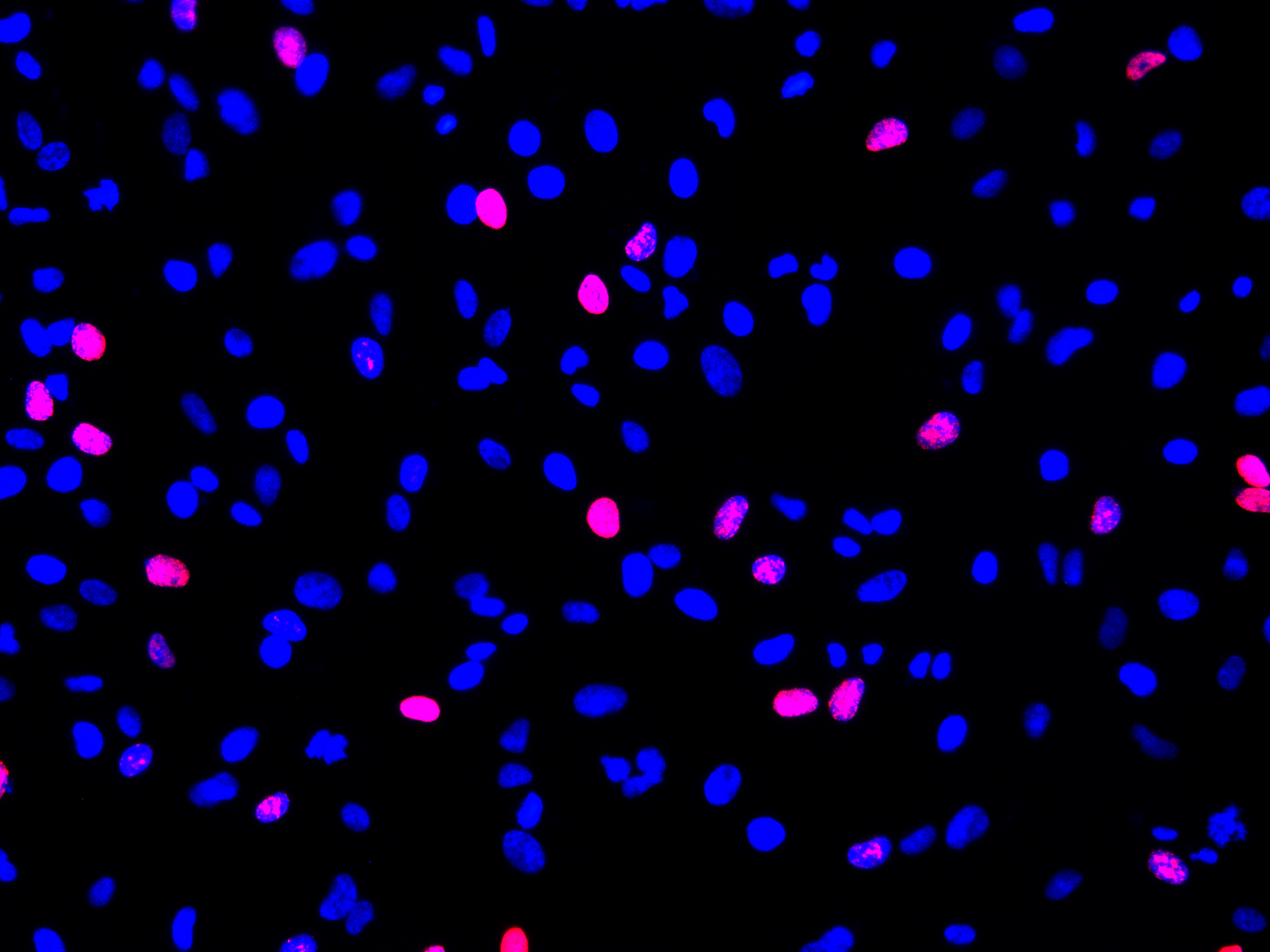

Supplement: Supplementary file 11 — Source Data Fig. 10 [file 44321_2024_25_MOESM11_ESM.zip › figure 10/10C/10C NG EdU DAPI.tif]

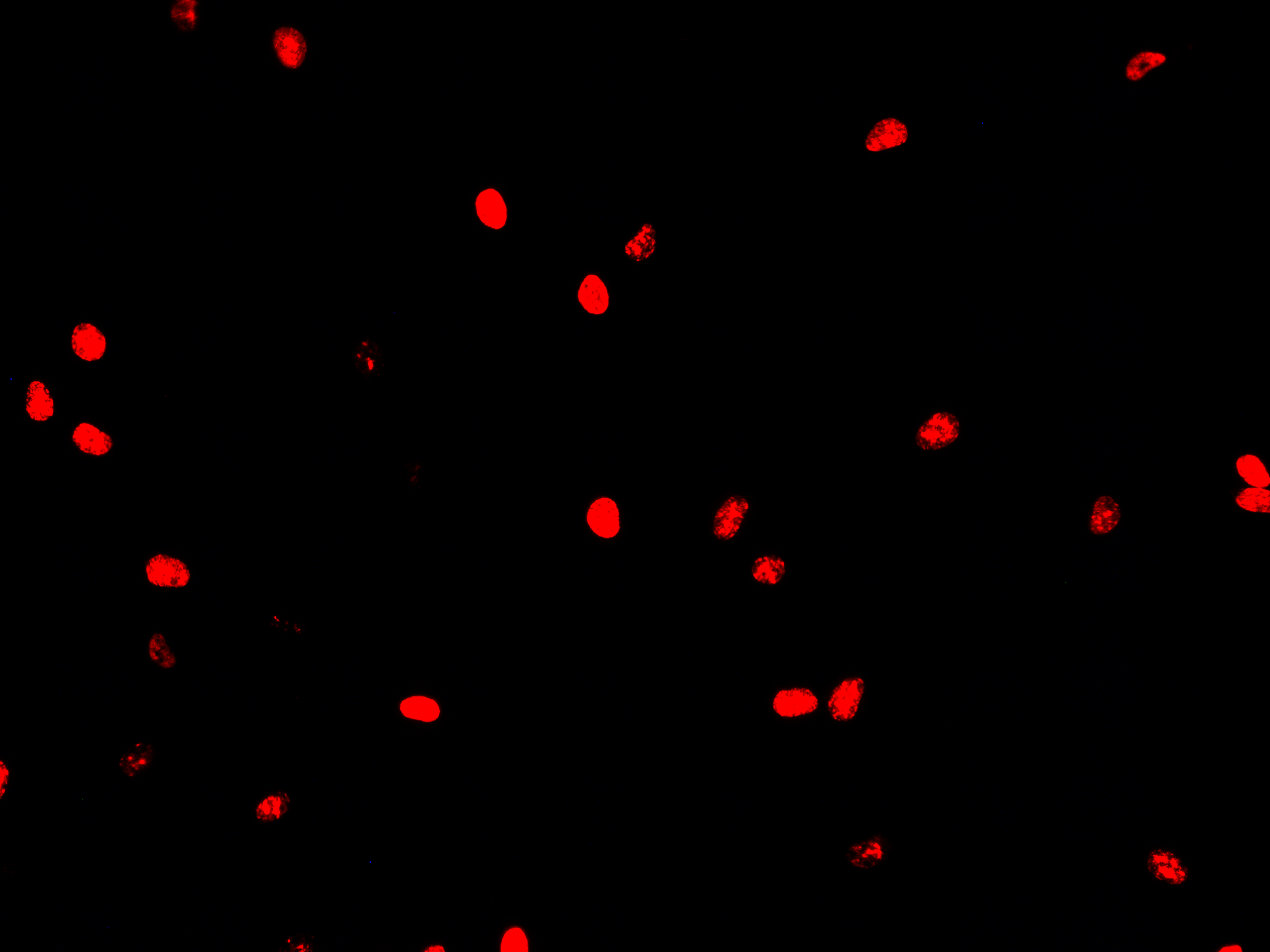

Supplement: Supplementary file 11 — Source Data Fig. 10 [file 44321_2024_25_MOESM11_ESM.zip › figure 10/10C/10C NG EdU.tif]

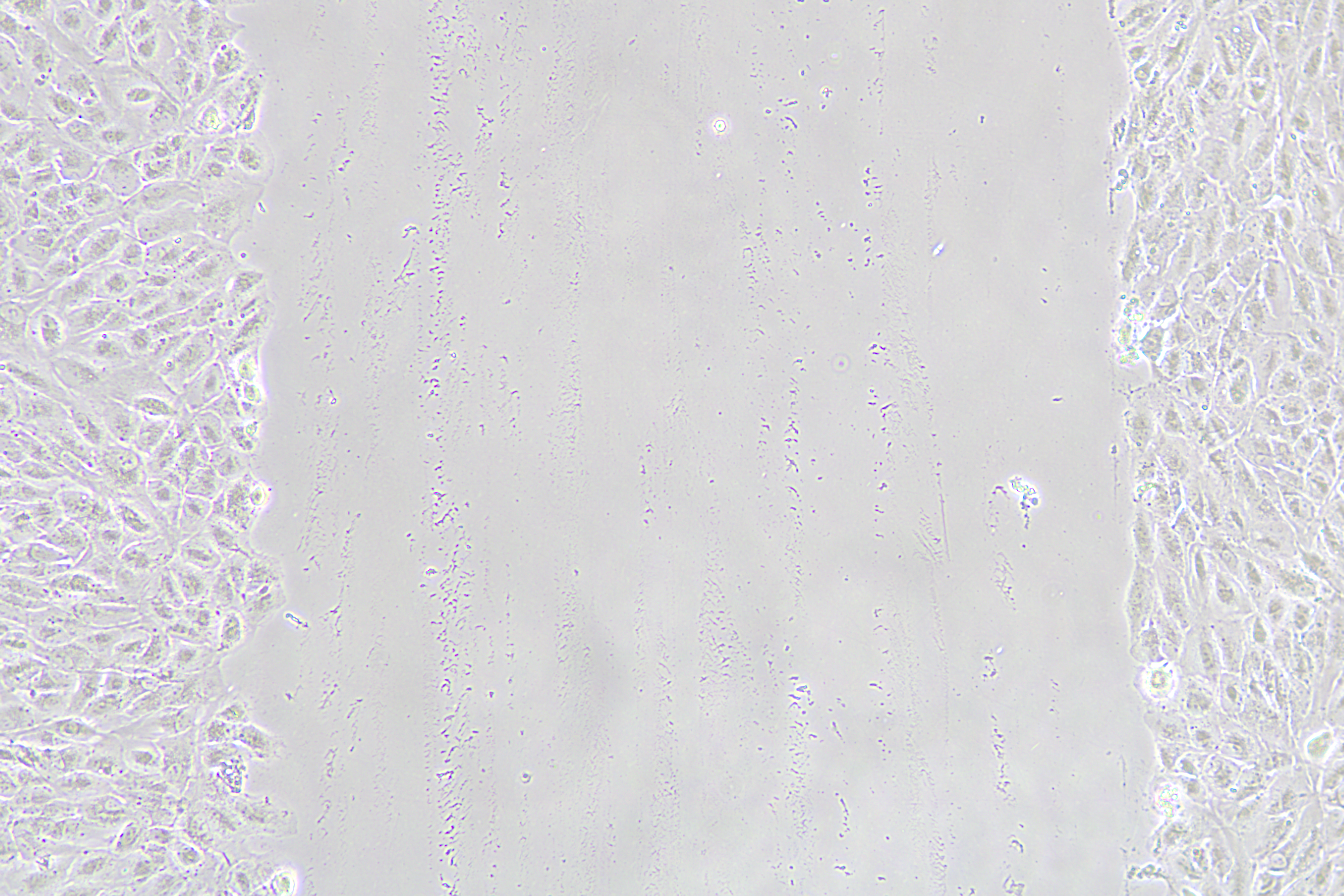

Supplement: Supplementary file 11 — Source Data Fig. 10 [file 44321_2024_25_MOESM11_ESM.zip › figure 10/10D/10D 0h HG.tif]

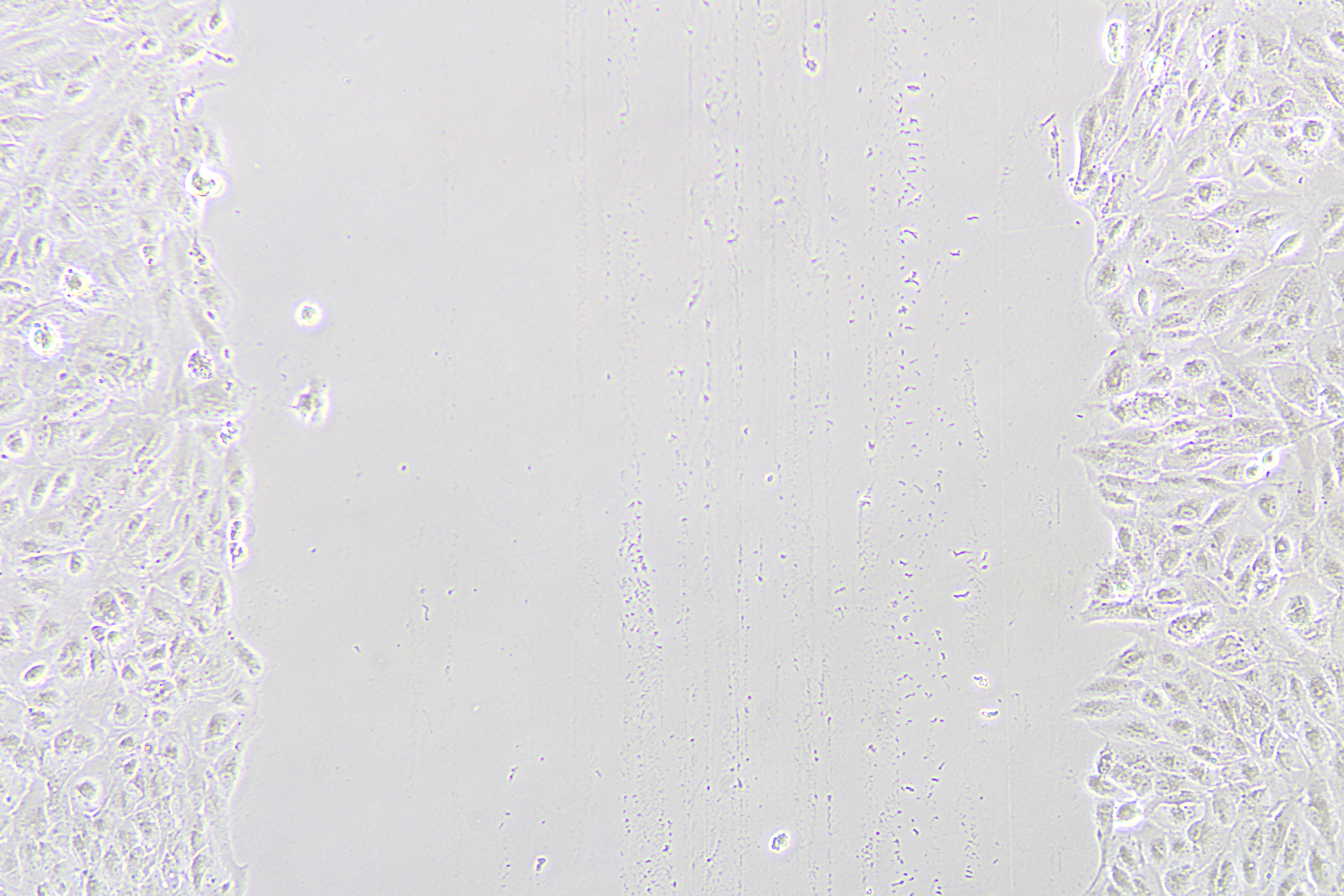

Supplement: Supplementary file 11 — Source Data Fig. 10 [file 44321_2024_25_MOESM11_ESM.zip › figure 10/10D/10D 0h HG+FB23-2.tif]

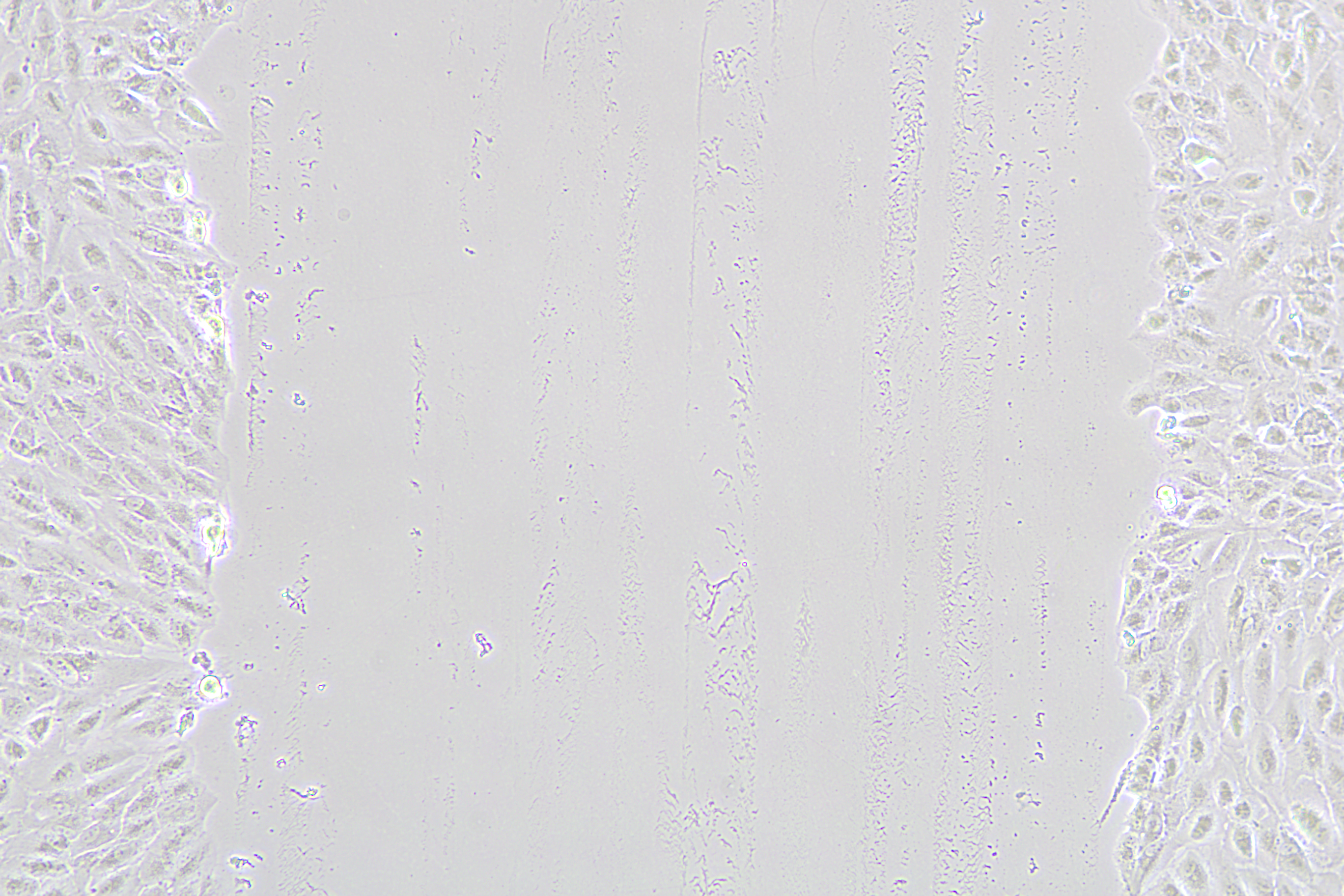

Supplement: Supplementary file 11 — Source Data Fig. 10 [file 44321_2024_25_MOESM11_ESM.zip › figure 10/10D/10D 0h NG.tif]

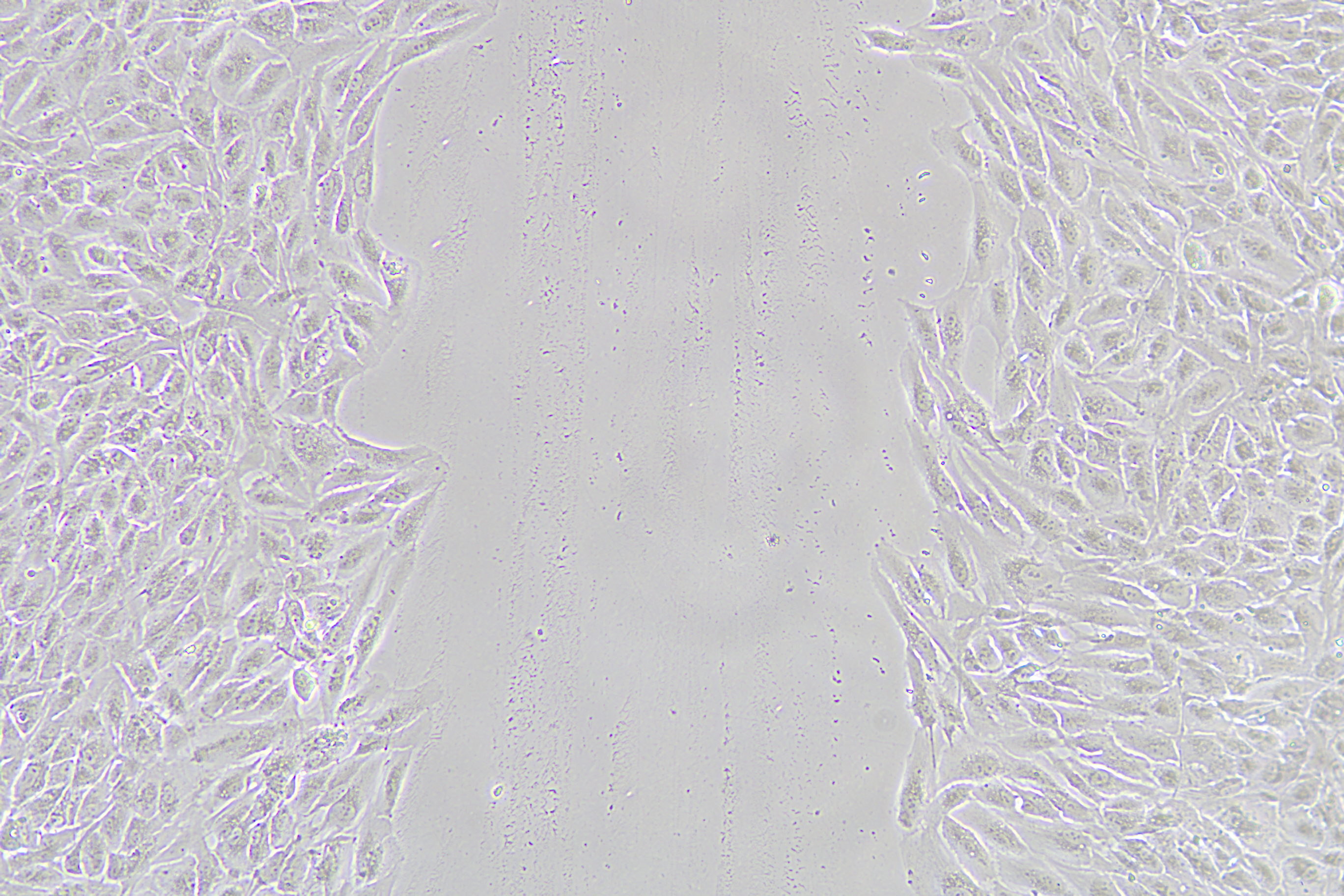

Supplement: Supplementary file 11 — Source Data Fig. 10 [file 44321_2024_25_MOESM11_ESM.zip › figure 10/10D/10D 24h HG.tif]

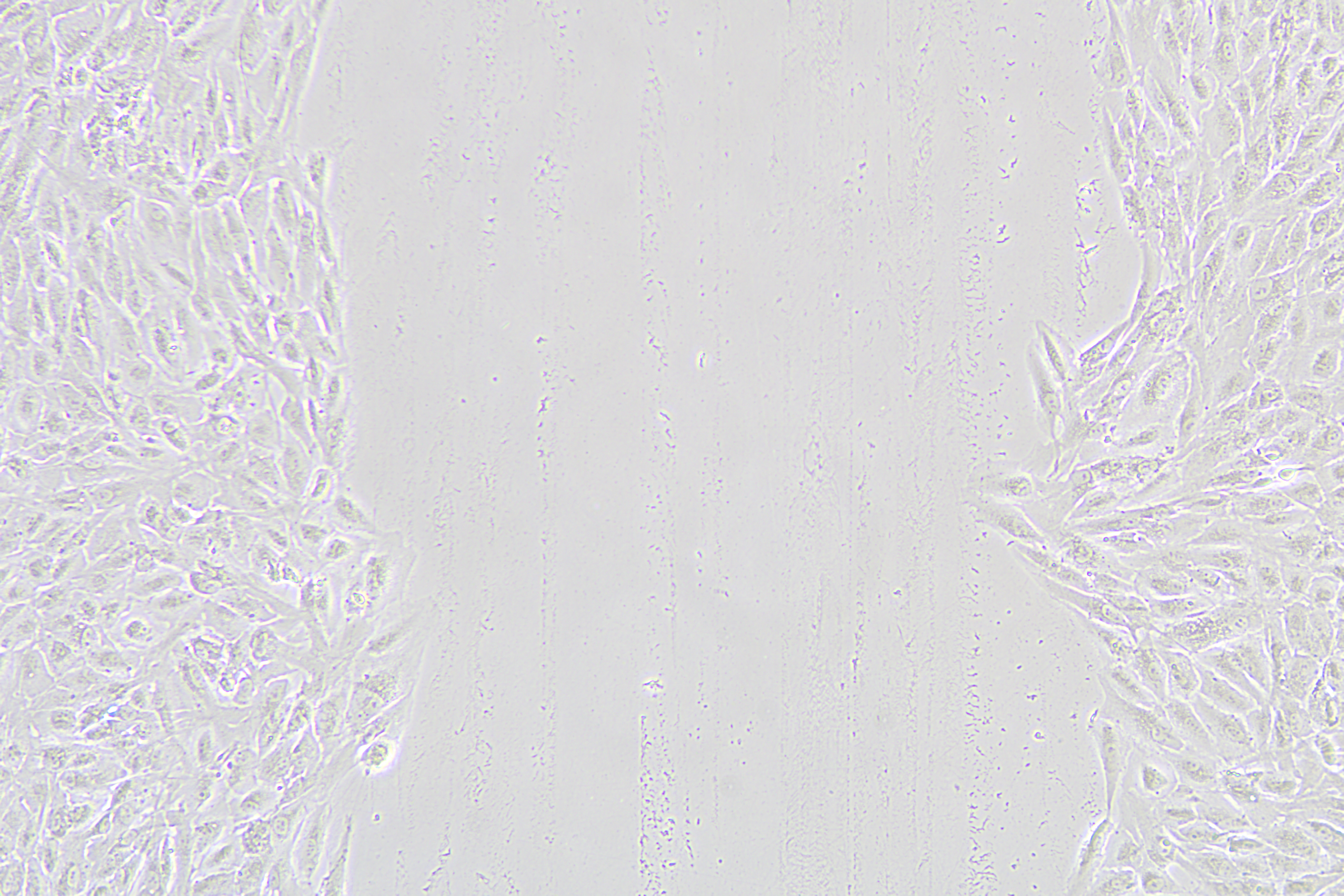

Supplement: Supplementary file 11 — Source Data Fig. 10 [file 44321_2024_25_MOESM11_ESM.zip › figure 10/10D/10D 24h HG+FB23-2.tif]

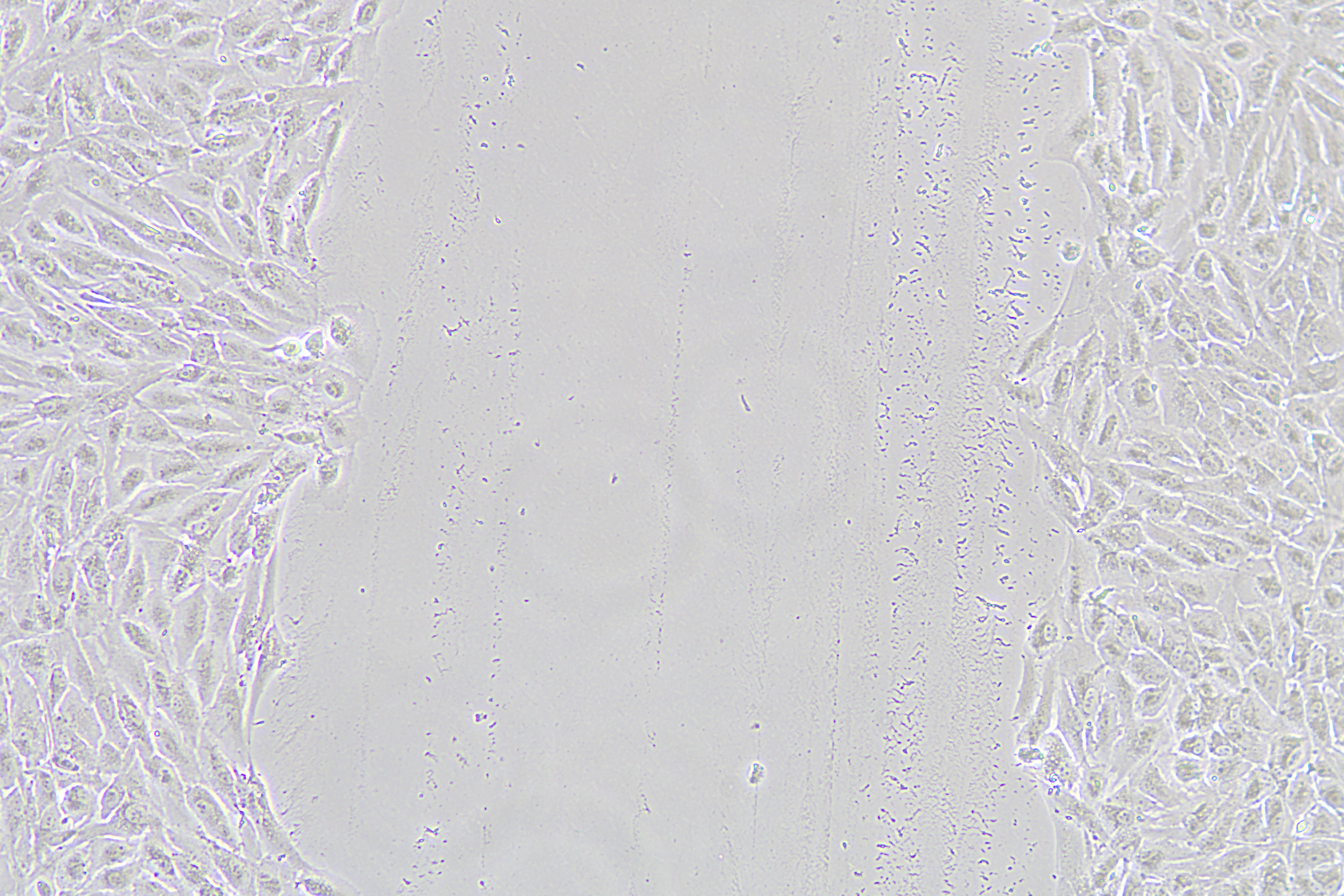

Supplement: Supplementary file 11 — Source Data Fig. 10 [file 44321_2024_25_MOESM11_ESM.zip › figure 10/10D/10D 24h NG.tif]

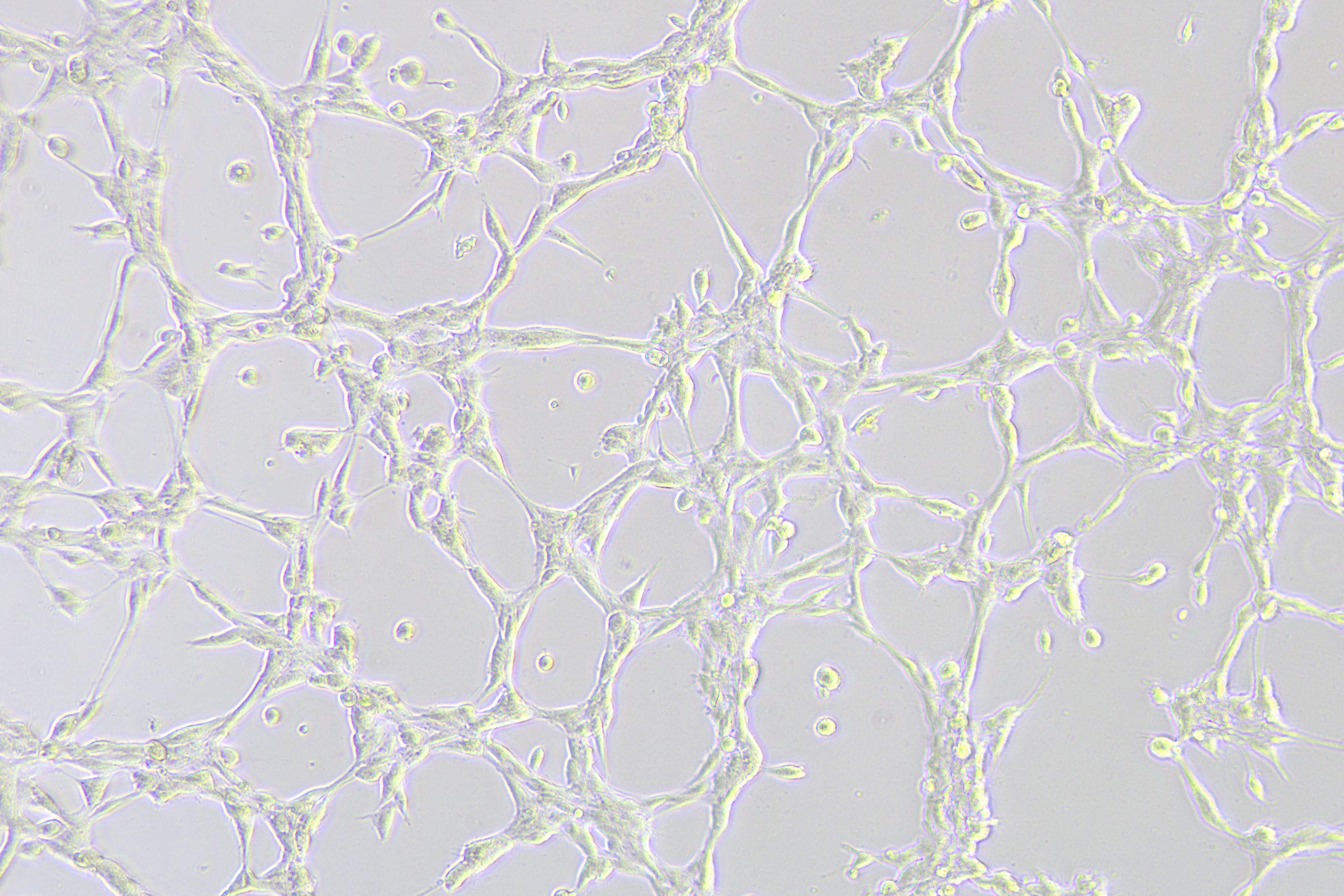

Supplement: Supplementary file 11 — Source Data Fig. 10 [file 44321_2024_25_MOESM11_ESM.zip › figure 10/10E/10E HG.tif]

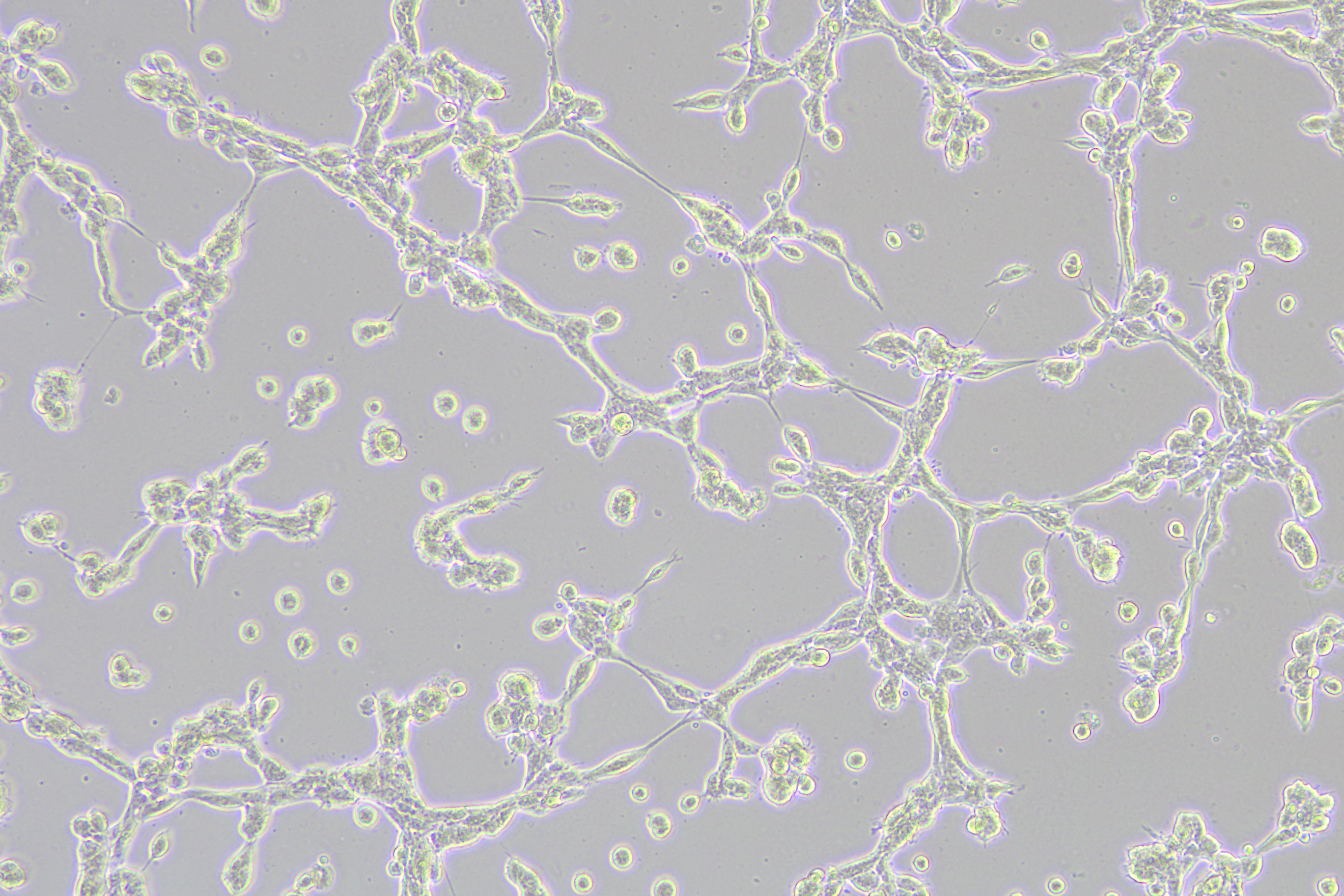

Supplement: Supplementary file 11 — Source Data Fig. 10 [file 44321_2024_25_MOESM11_ESM.zip › figure 10/10E/10E NG.tif]

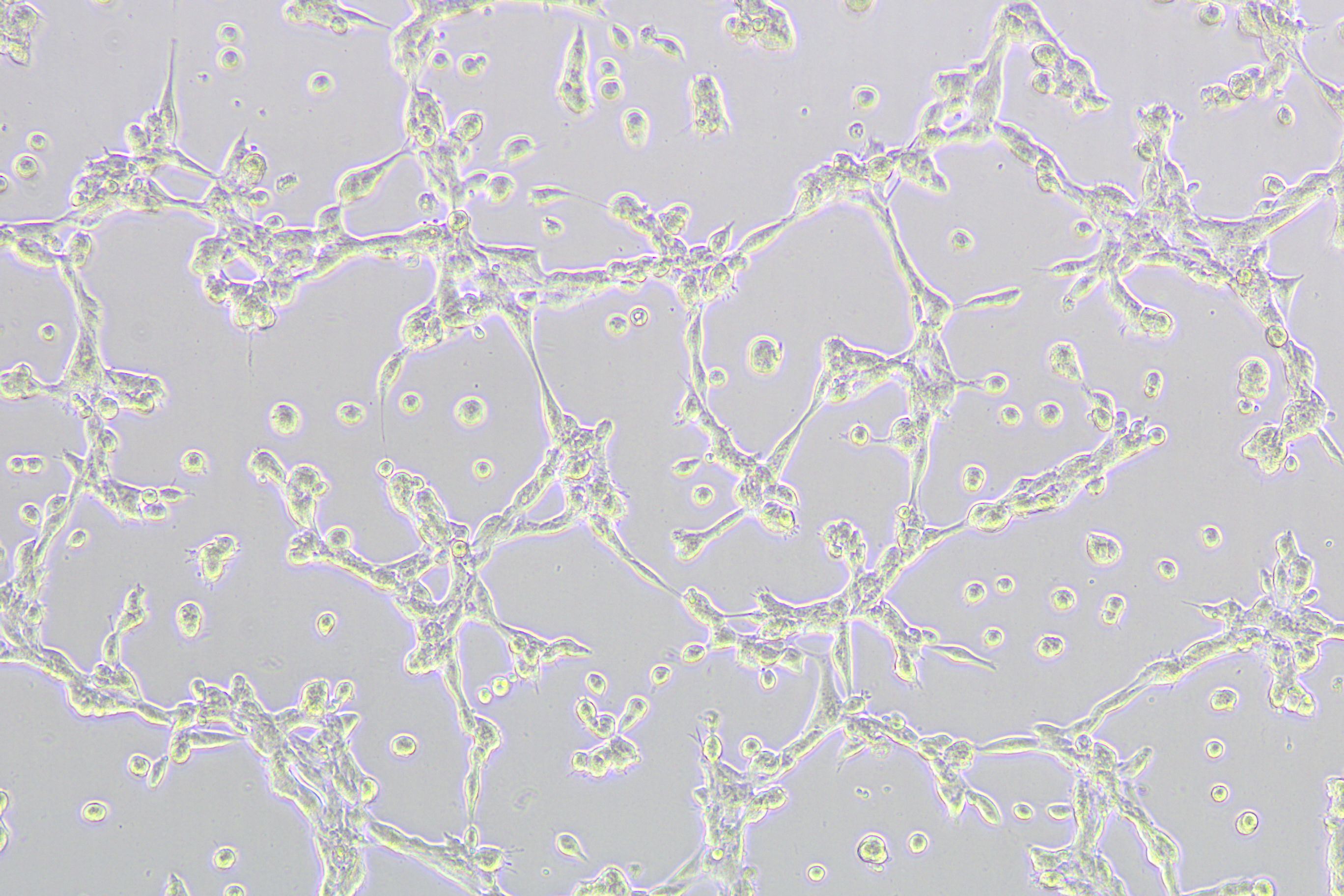

Supplement: Supplementary file 11 — Source Data Fig. 10 [file 44321_2024_25_MOESM11_ESM.zip › figure 10/10E/10EHG+FB23-2.tif]

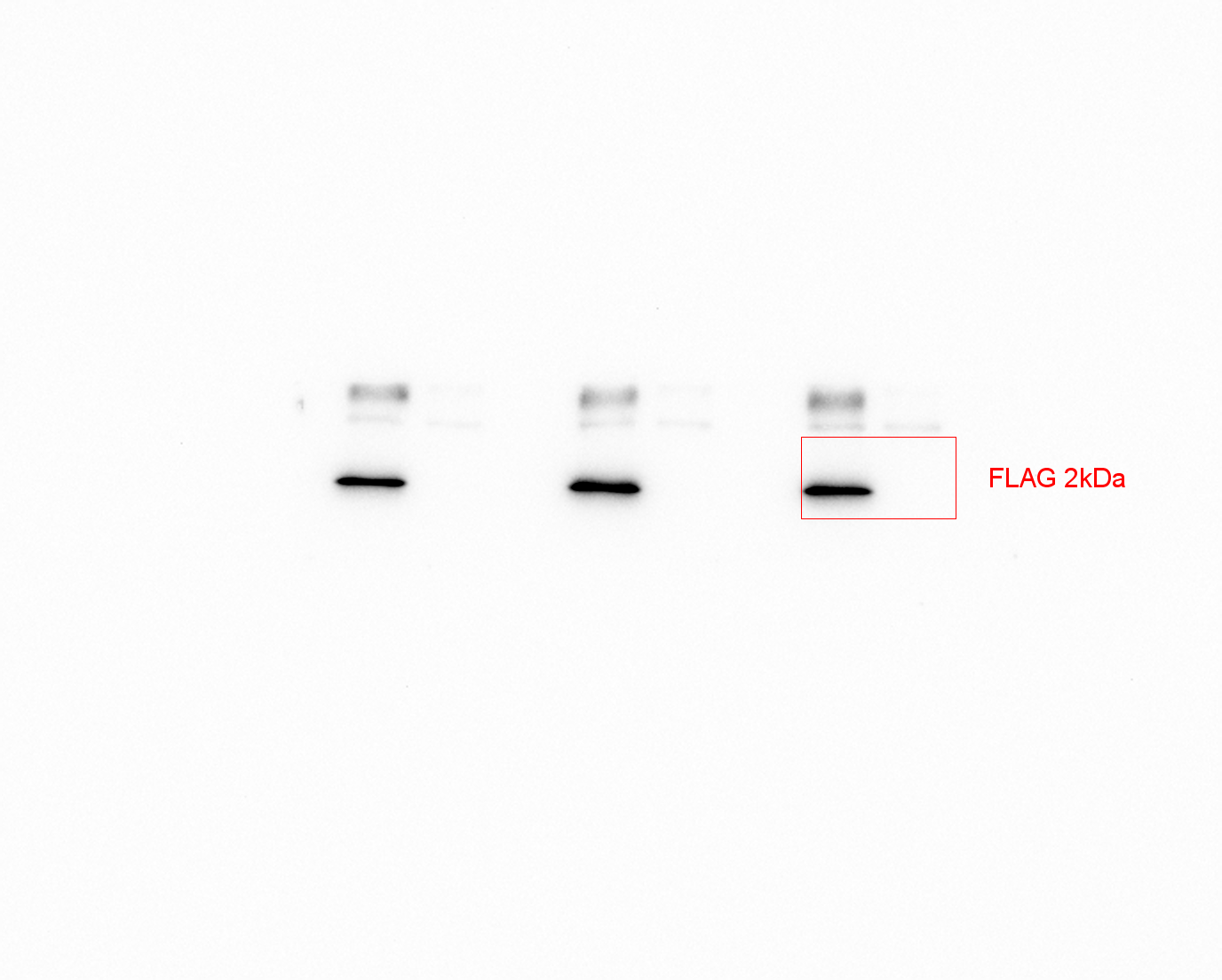

Supplement: Supplementary file 13 — Appendix Figures Source Data [file 44321_2024_25_MOESM13_ESM.zip › Appendix Figures/Appendix Figure S2/S2A/S2A FLAG 2kDa mark.Tif]

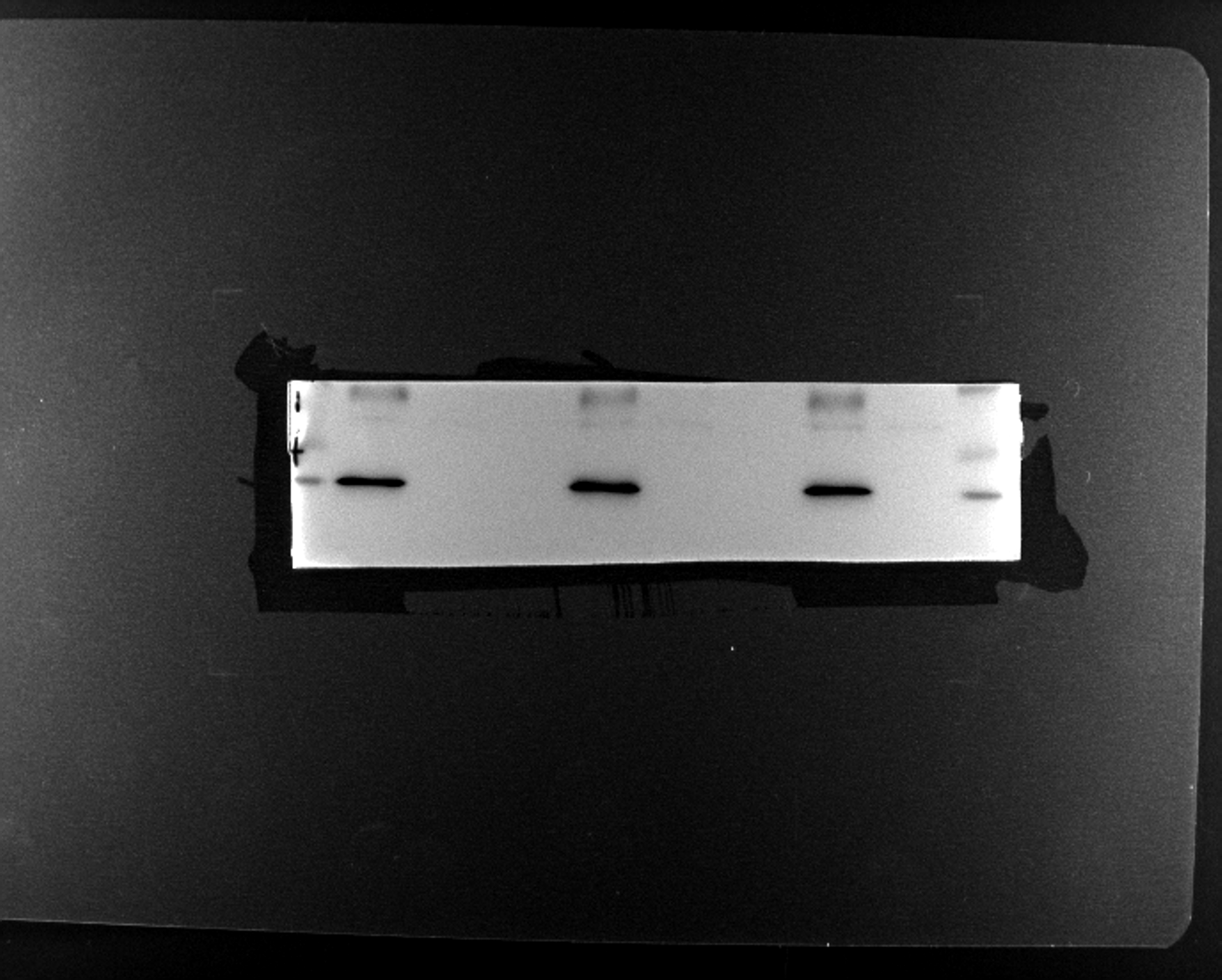

Supplement: Supplementary file 13 — Appendix Figures Source Data [file 44321_2024_25_MOESM13_ESM.zip › Appendix Figures/Appendix Figure S2/S2A/S2A FLAG 2kDa.Tif]

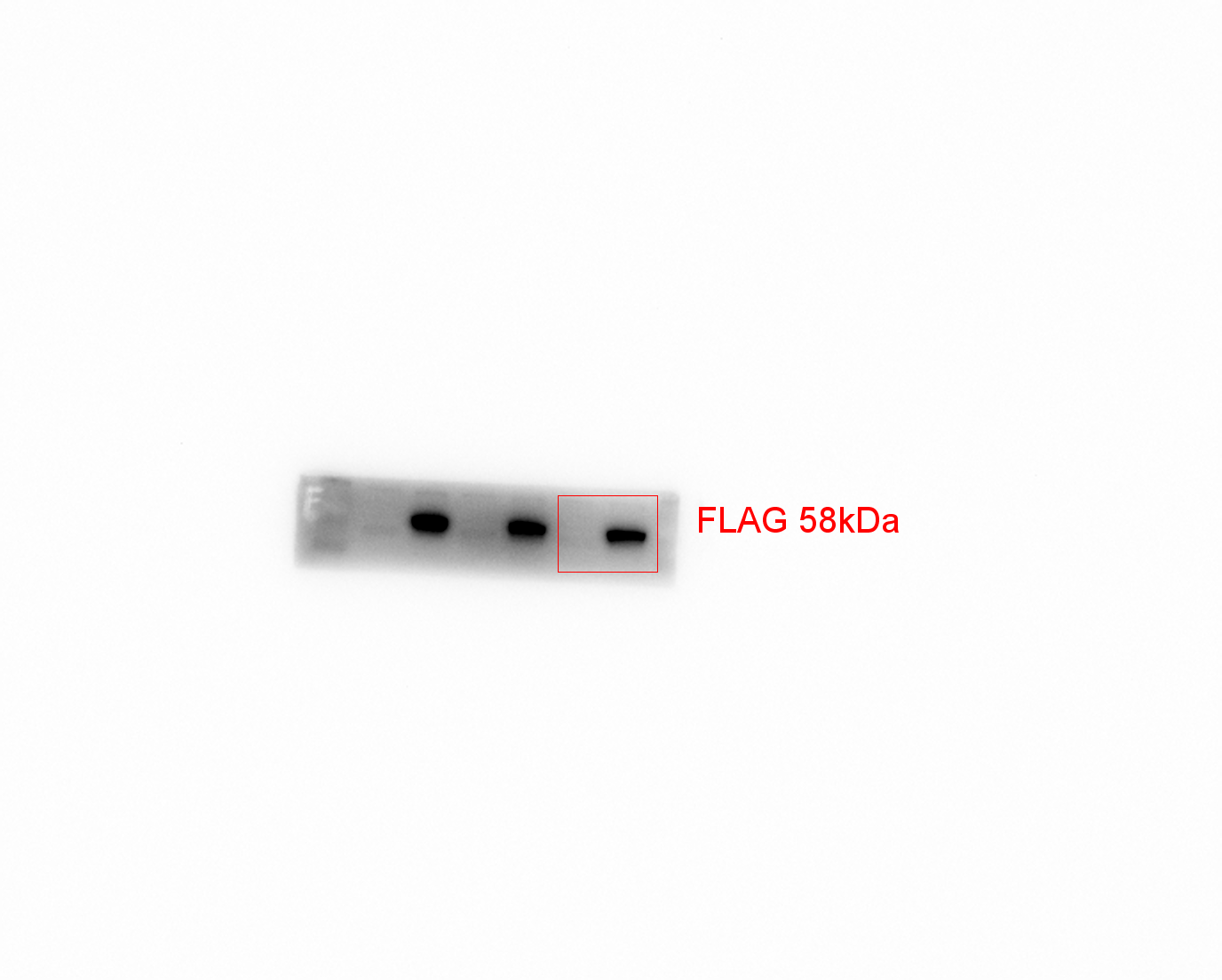

Supplement: Supplementary file 13 — Appendix Figures Source Data [file 44321_2024_25_MOESM13_ESM.zip › Appendix Figures/Appendix Figure S2/S2A/S2A FLAG 58kDa mark.Tif]

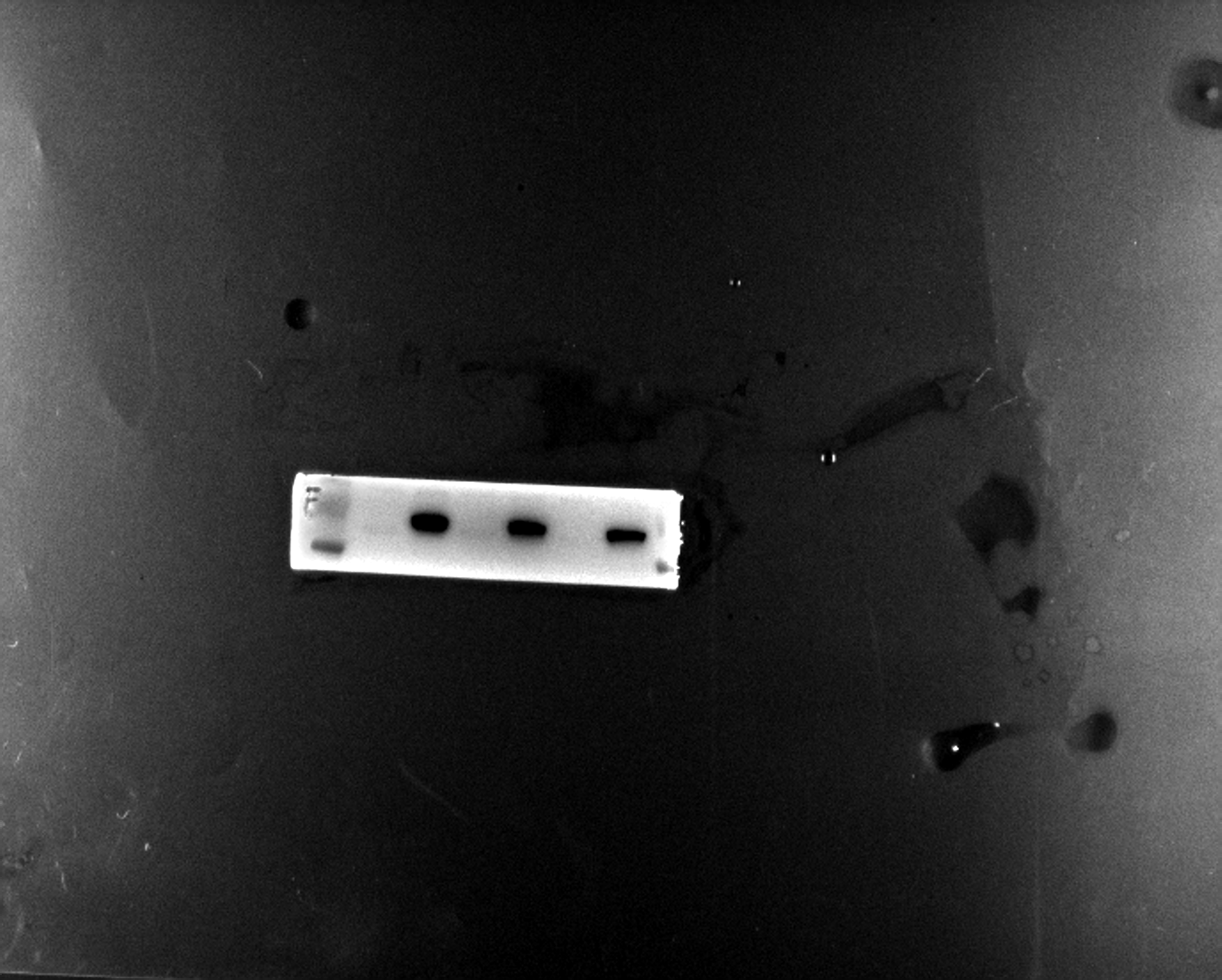

Supplement: Supplementary file 13 — Appendix Figures Source Data [file 44321_2024_25_MOESM13_ESM.zip › Appendix Figures/Appendix Figure S2/S2A/S2A FLAG 58kDa.Tif]

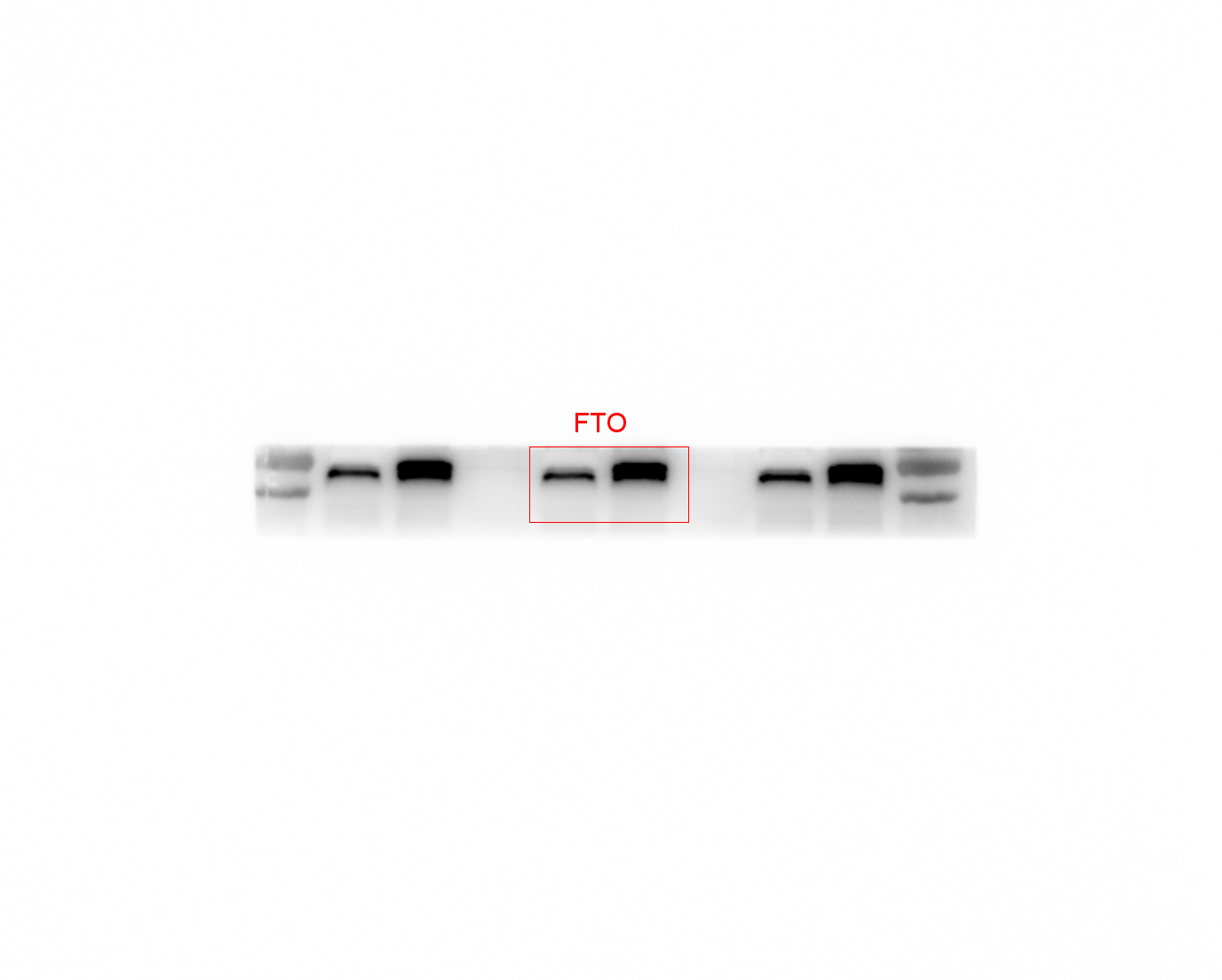

Supplement: Supplementary file 13 — Appendix Figures Source Data [file 44321_2024_25_MOESM13_ESM.zip › Appendix Figures/Appendix Figure S2/S2A/S2A FTO mark.Tif]

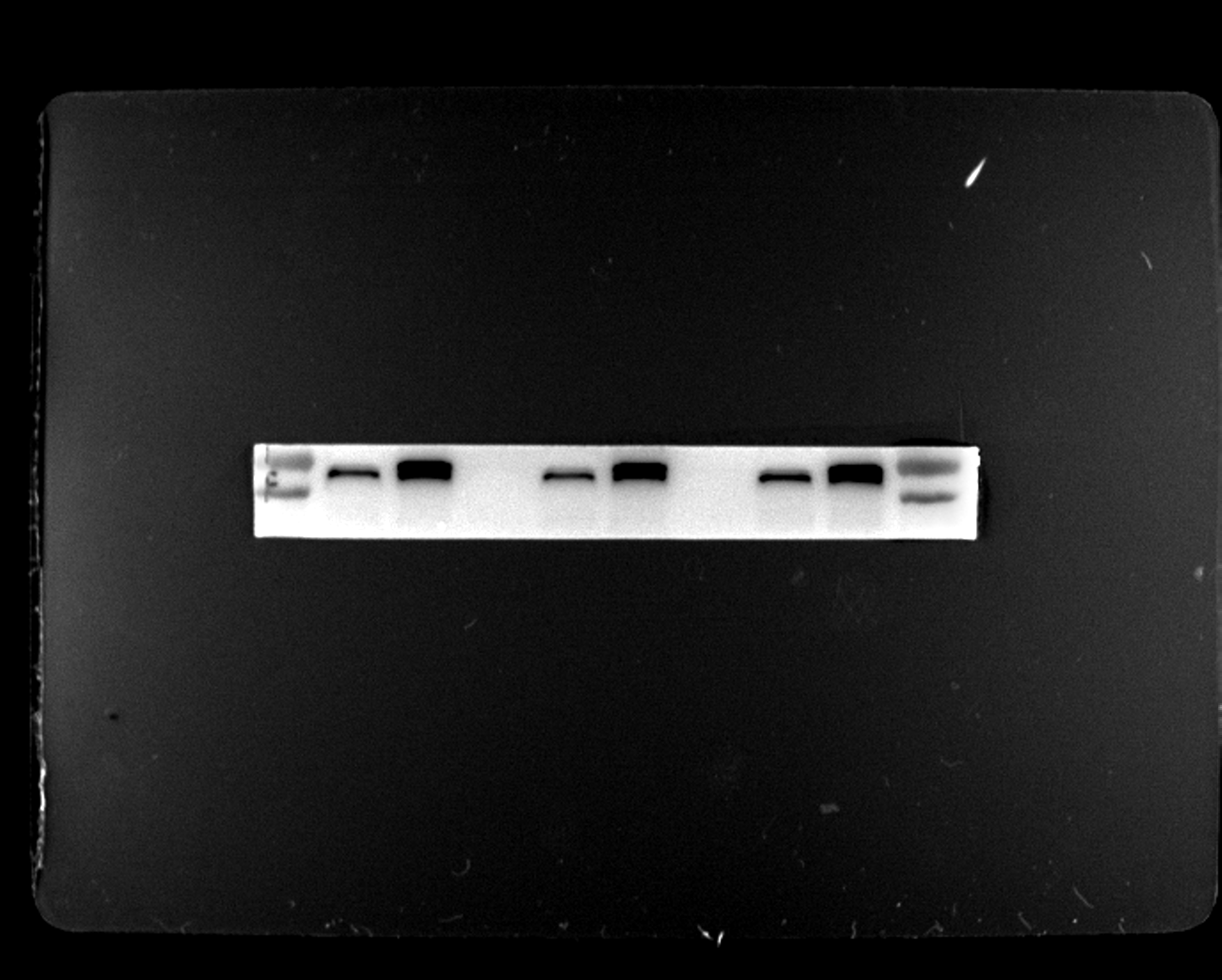

Supplement: Supplementary file 13 — Appendix Figures Source Data [file 44321_2024_25_MOESM13_ESM.zip › Appendix Figures/Appendix Figure S2/S2A/S2A FTO.Tif]

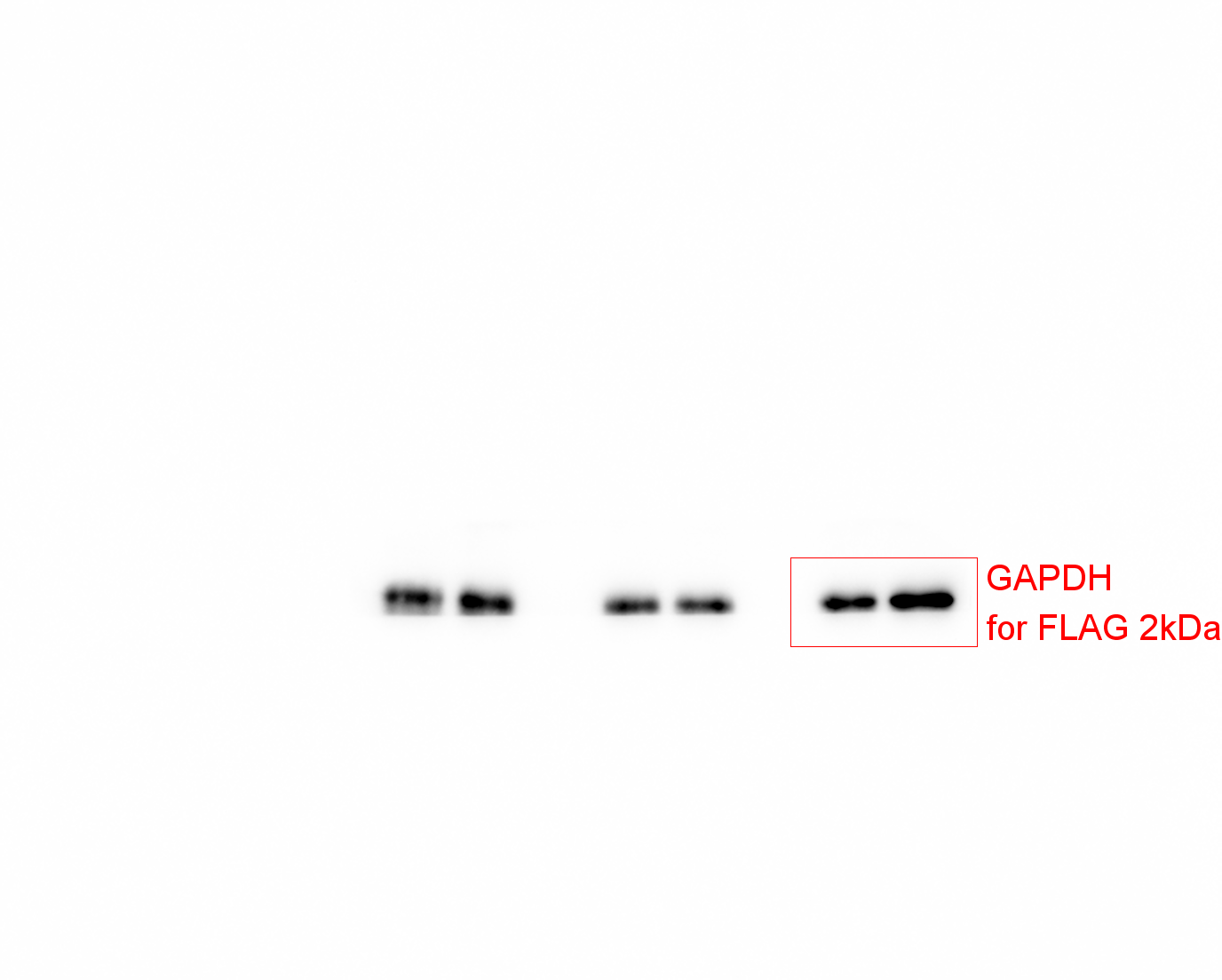

Supplement: Supplementary file 13 — Appendix Figures Source Data [file 44321_2024_25_MOESM13_ESM.zip › Appendix Figures/Appendix Figure S2/S2A/S2A GAPDH for FLAG 2kDa mark.Tif]

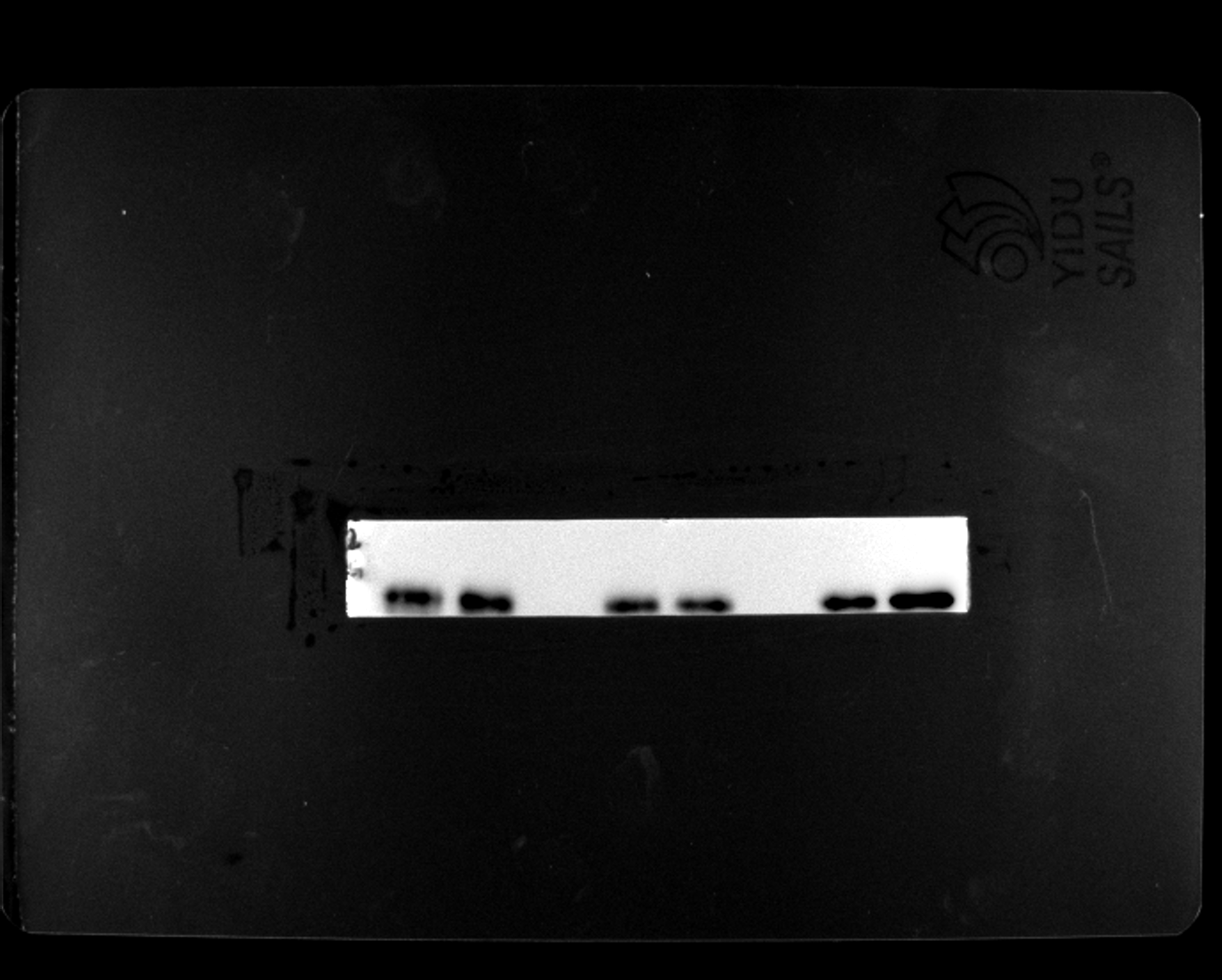

Supplement: Supplementary file 13 — Appendix Figures Source Data [file 44321_2024_25_MOESM13_ESM.zip › Appendix Figures/Appendix Figure S2/S2A/S2A GAPDH for FLAG 2kDa.Tif]

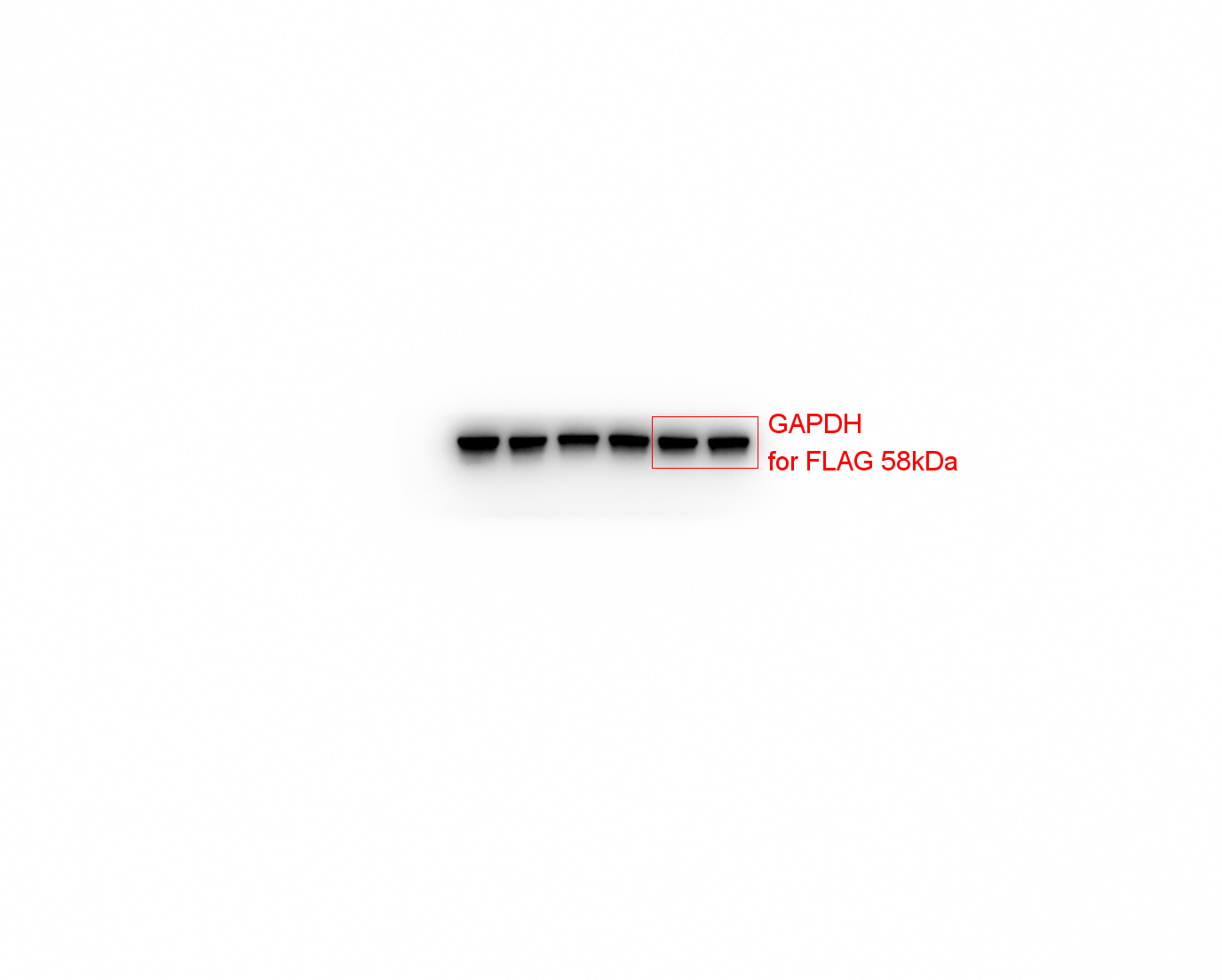

Supplement: Supplementary file 13 — Appendix Figures Source Data [file 44321_2024_25_MOESM13_ESM.zip › Appendix Figures/Appendix Figure S2/S2A/S2A GAPDH for FLAG 58kDa mark.Tif]

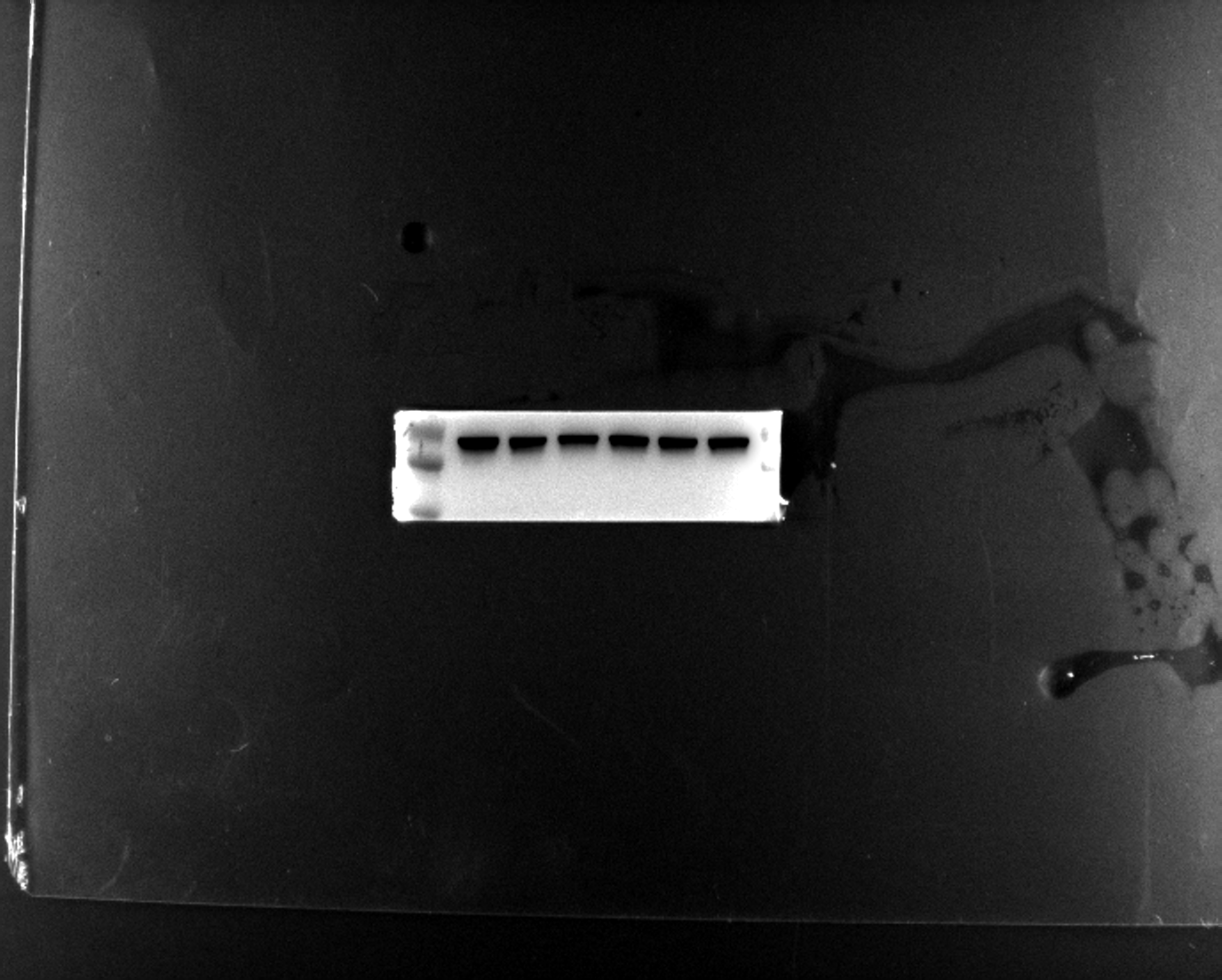

Supplement: Supplementary file 13 — Appendix Figures Source Data [file 44321_2024_25_MOESM13_ESM.zip › Appendix Figures/Appendix Figure S2/S2A/S2A GAPDH for FLAG 58kDa.Tif]

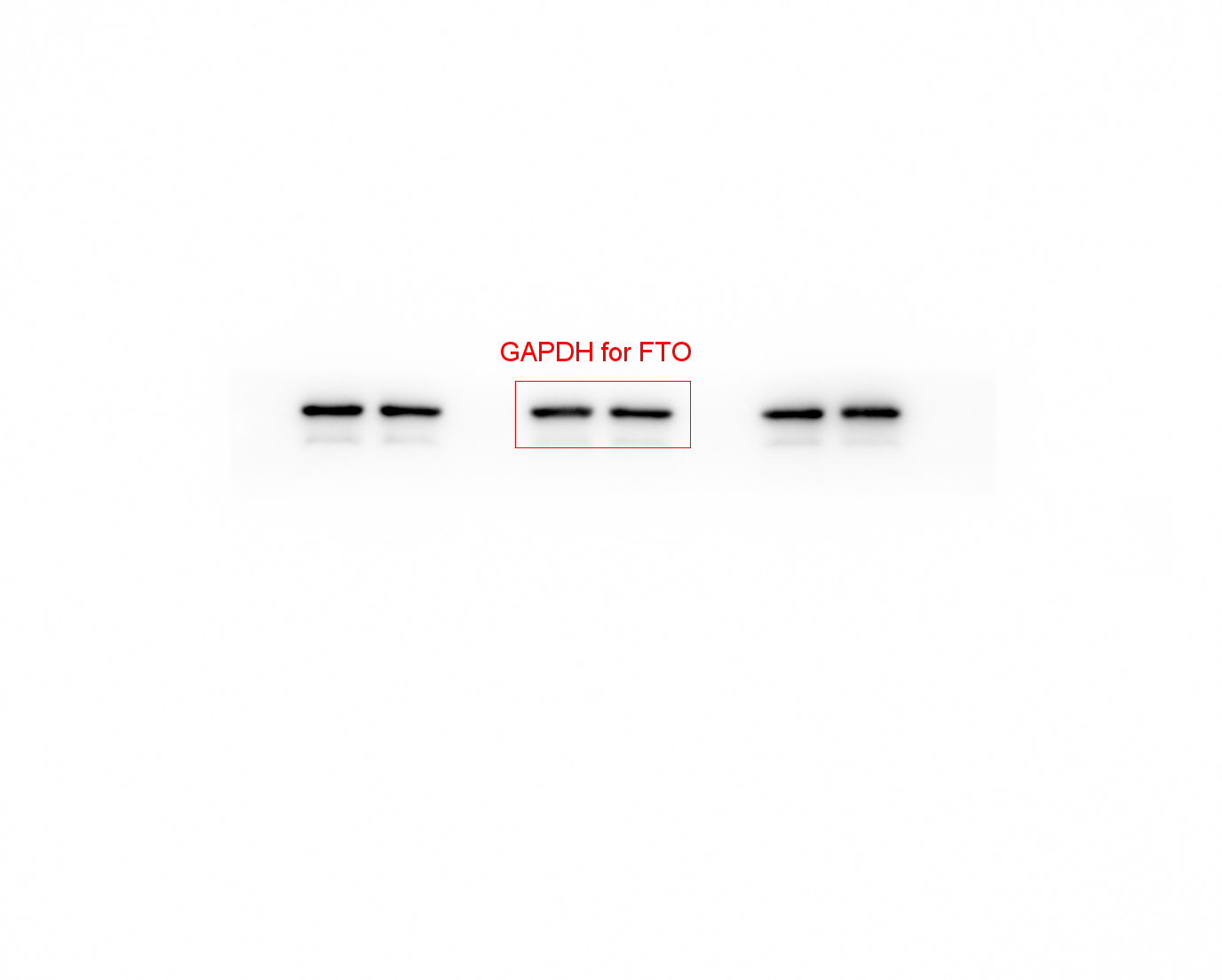

Supplement: Supplementary file 13 — Appendix Figures Source Data [file 44321_2024_25_MOESM13_ESM.zip › Appendix Figures/Appendix Figure S2/S2A/S2A GAPDH for FTO mark.Tif]

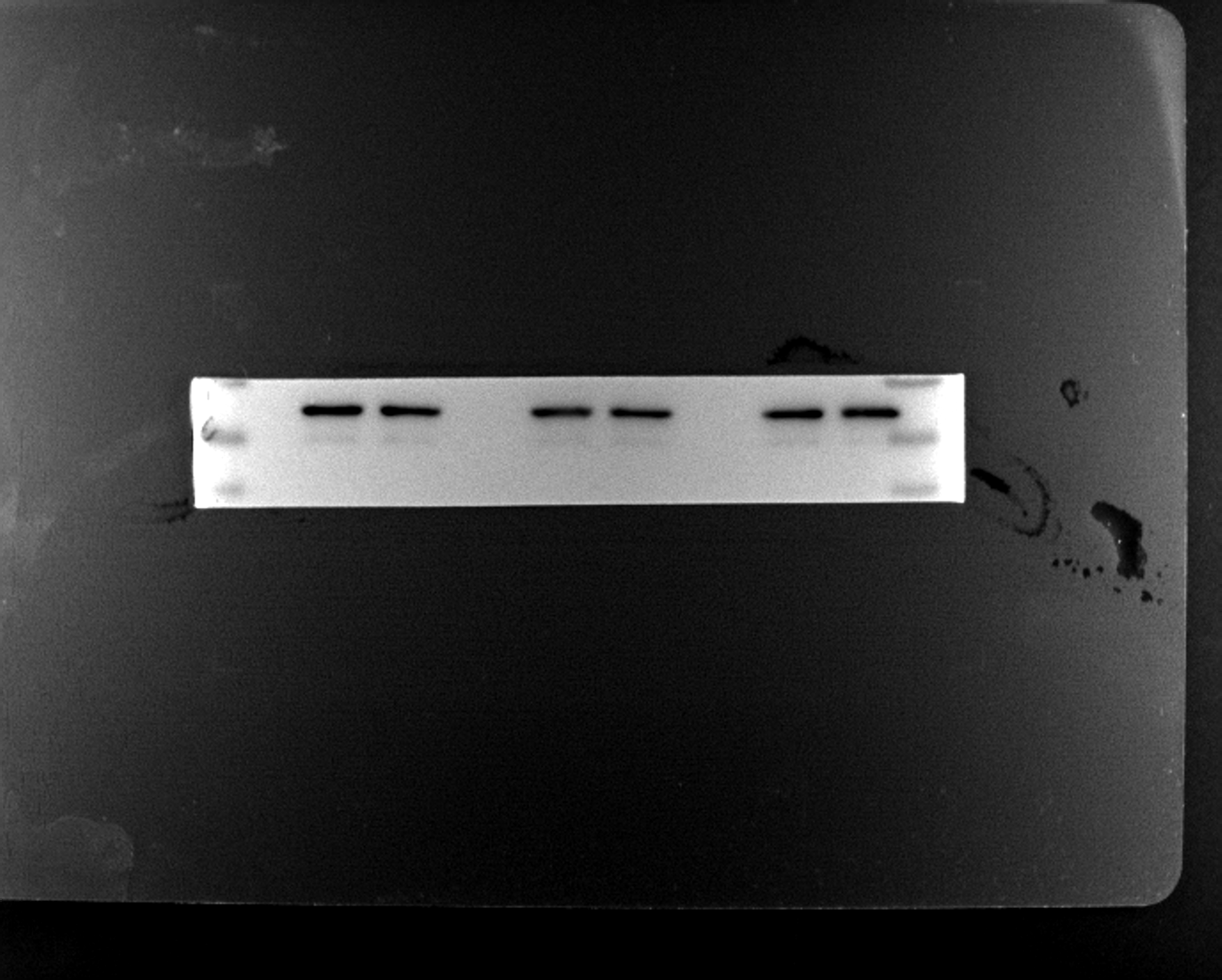

Supplement: Supplementary file 13 — Appendix Figures Source Data [file 44321_2024_25_MOESM13_ESM.zip › Appendix Figures/Appendix Figure S2/S2A/S2A GAPDH for FTO.Tif]

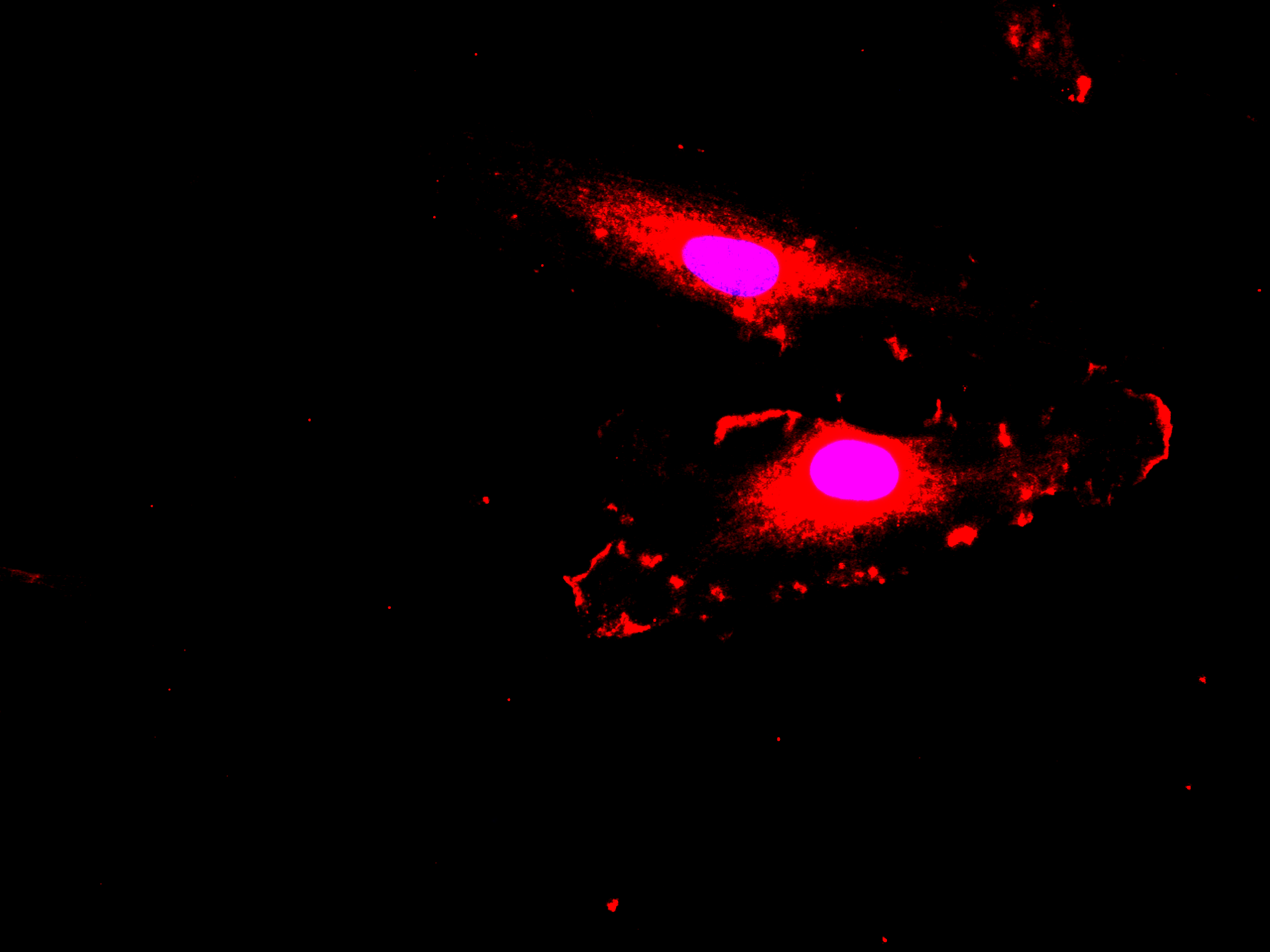

Supplement: Supplementary file 13 — Appendix Figures Source Data [file 44321_2024_25_MOESM13_ESM.zip › Appendix Figures/Appendix Figure S2/S2B/S2B L-EV FLAG DAPI.tif]

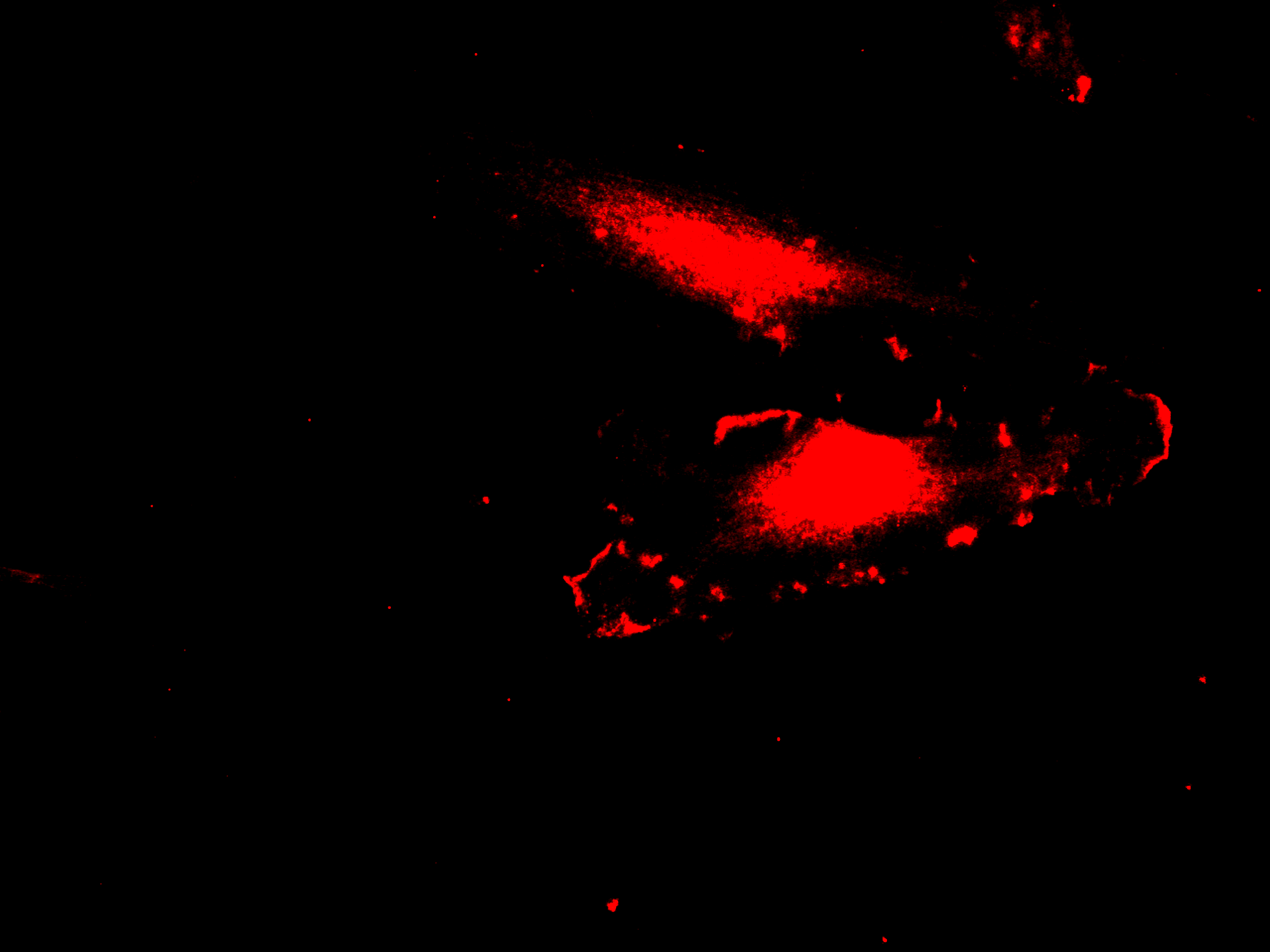

Supplement: Supplementary file 13 — Appendix Figures Source Data [file 44321_2024_25_MOESM13_ESM.zip › Appendix Figures/Appendix Figure S2/S2B/S2B L-EV FLAG.tif]

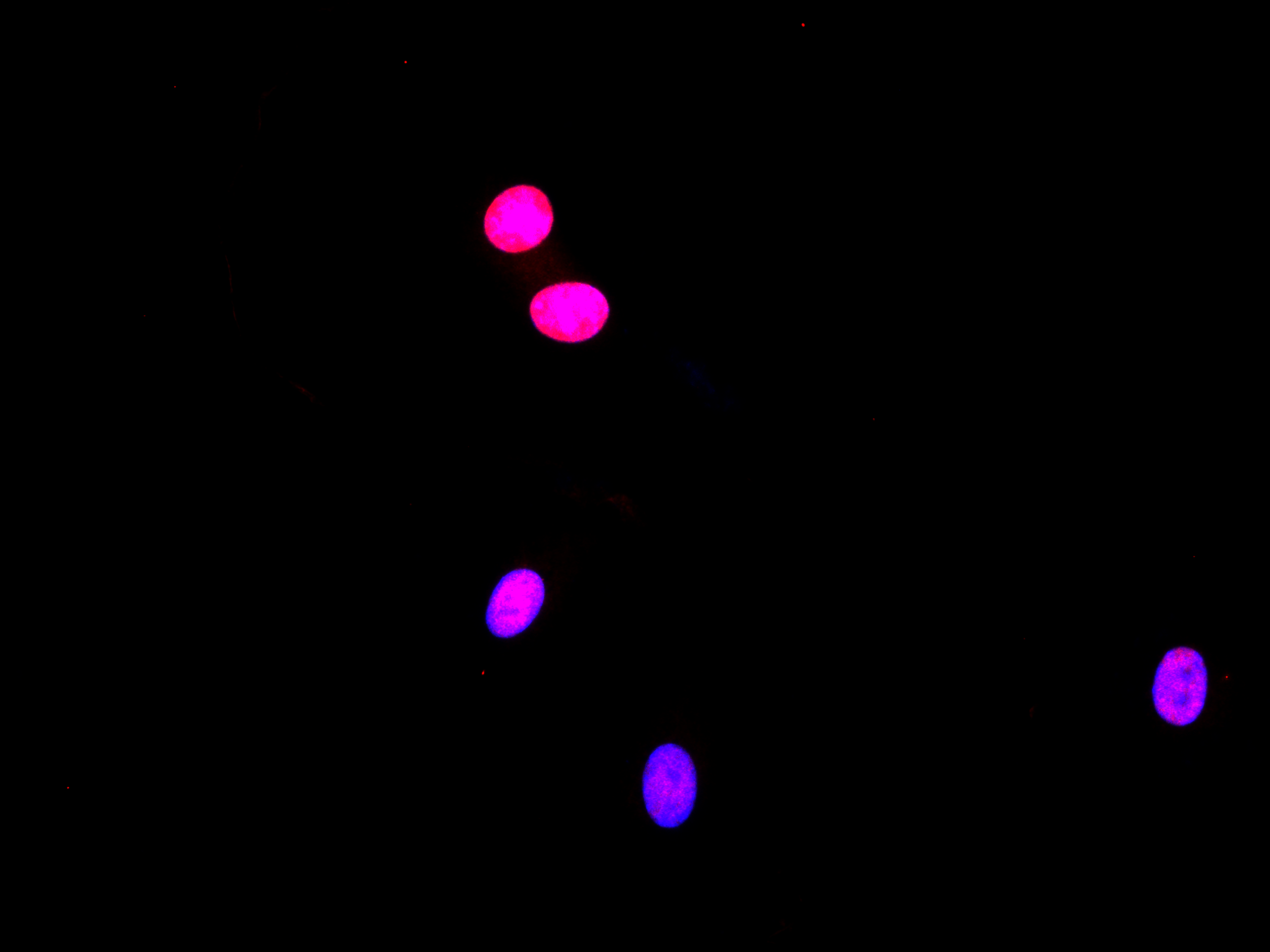

Supplement: Supplementary file 13 — Appendix Figures Source Data [file 44321_2024_25_MOESM13_ESM.zip › Appendix Figures/Appendix Figure S2/S2B/S2B L-FTO FLAG DAPI.tif]

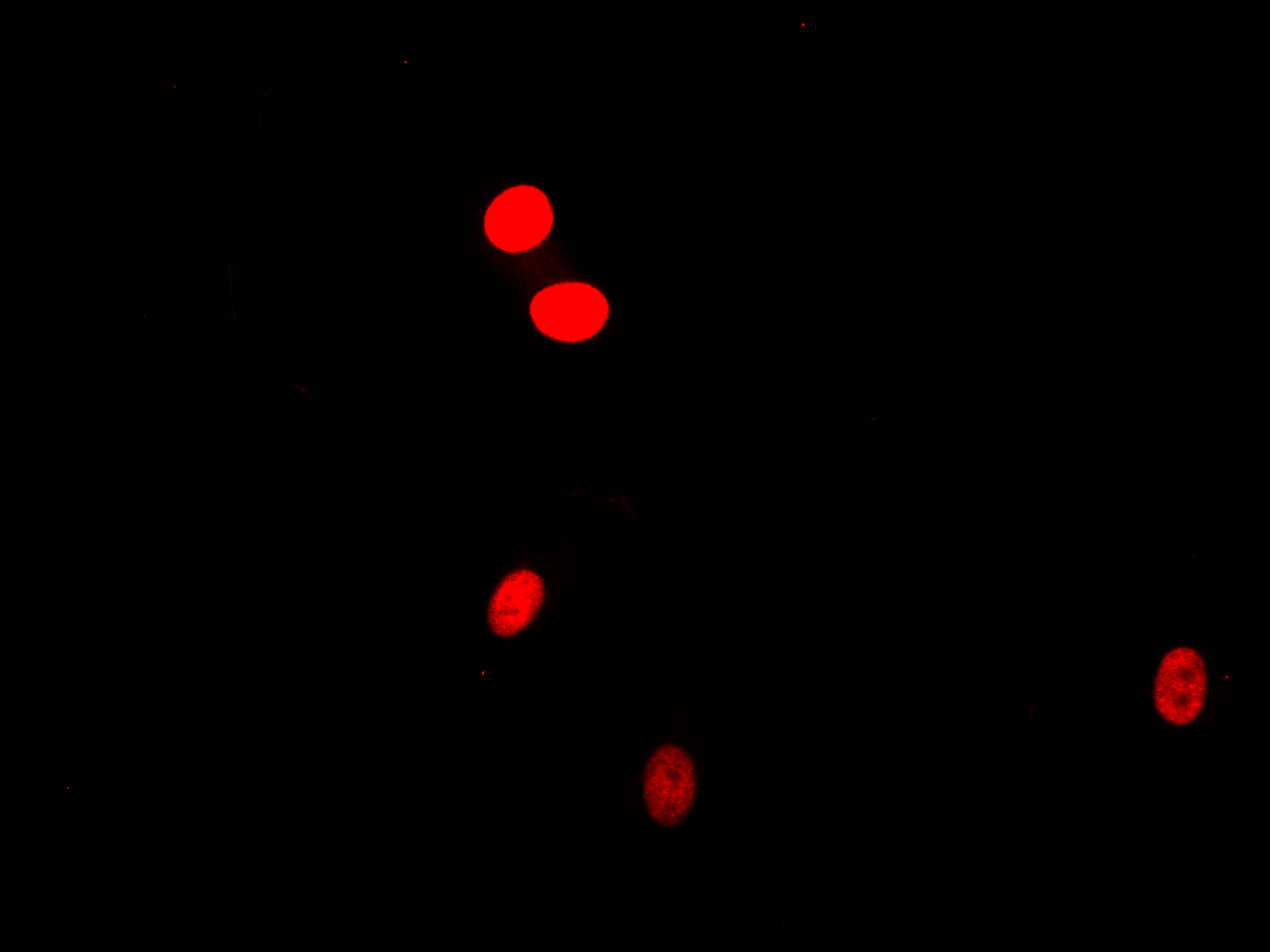

Supplement: Supplementary file 13 — Appendix Figures Source Data [file 44321_2024_25_MOESM13_ESM.zip › Appendix Figures/Appendix Figure S2/S2B/S2B L-FTO FLAG.tif]

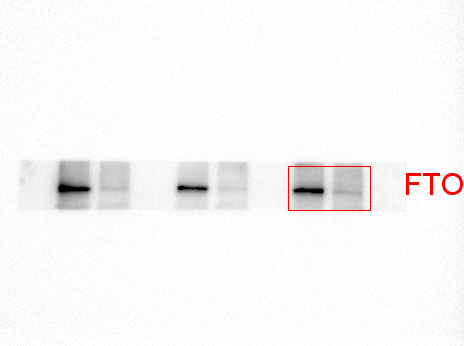

Supplement: Supplementary file 13 — Appendix Figures Source Data [file 44321_2024_25_MOESM13_ESM.zip › Appendix Figures/Appendix Figure S2/S2D/S2D FTO mark.tif]

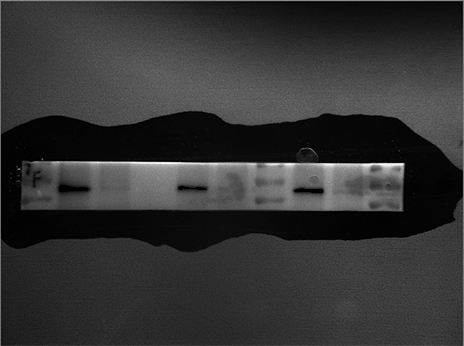

Supplement: Supplementary file 13 — Appendix Figures Source Data [file 44321_2024_25_MOESM13_ESM.zip › Appendix Figures/Appendix Figure S2/S2D/S2D FTO.tif]

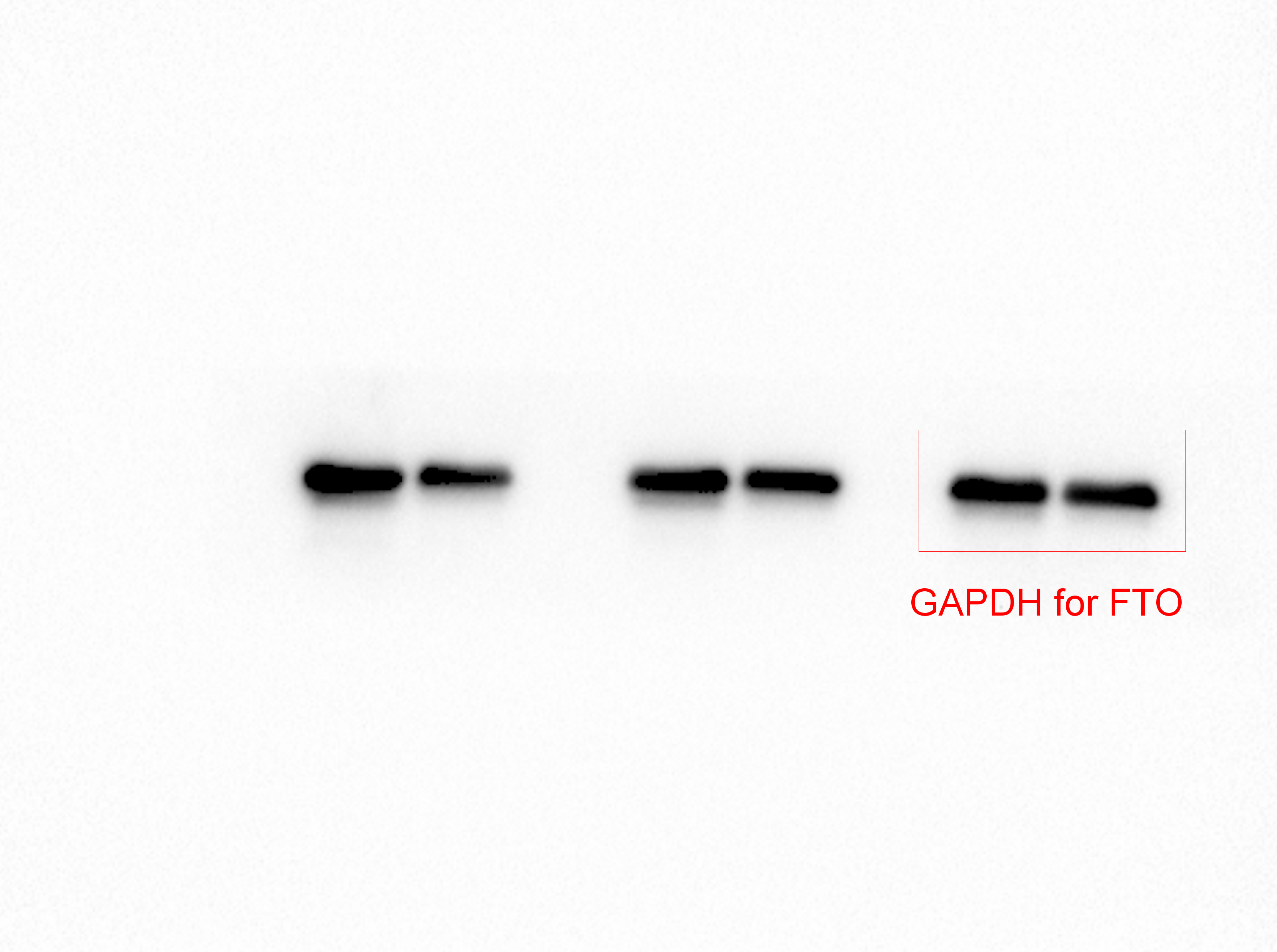

Supplement: Supplementary file 13 — Appendix Figures Source Data [file 44321_2024_25_MOESM13_ESM.zip › Appendix Figures/Appendix Figure S2/S2D/S2D GAPDH for FTO mark.tiff]

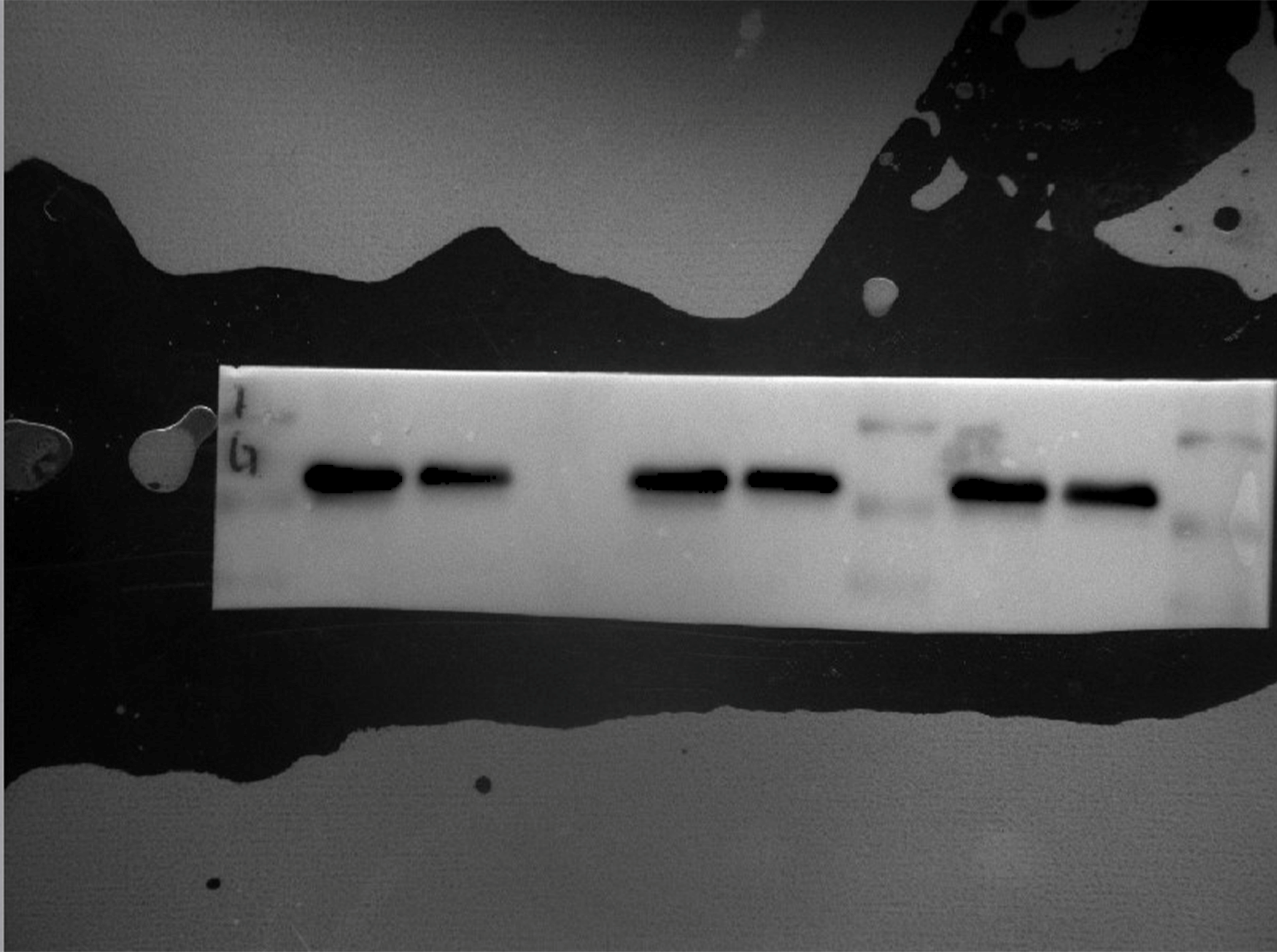

Supplement: Supplementary file 13 — Appendix Figures Source Data [file 44321_2024_25_MOESM13_ESM.zip › Appendix Figures/Appendix Figure S2/S2D/S2D GAPDH for FTO.tiff]

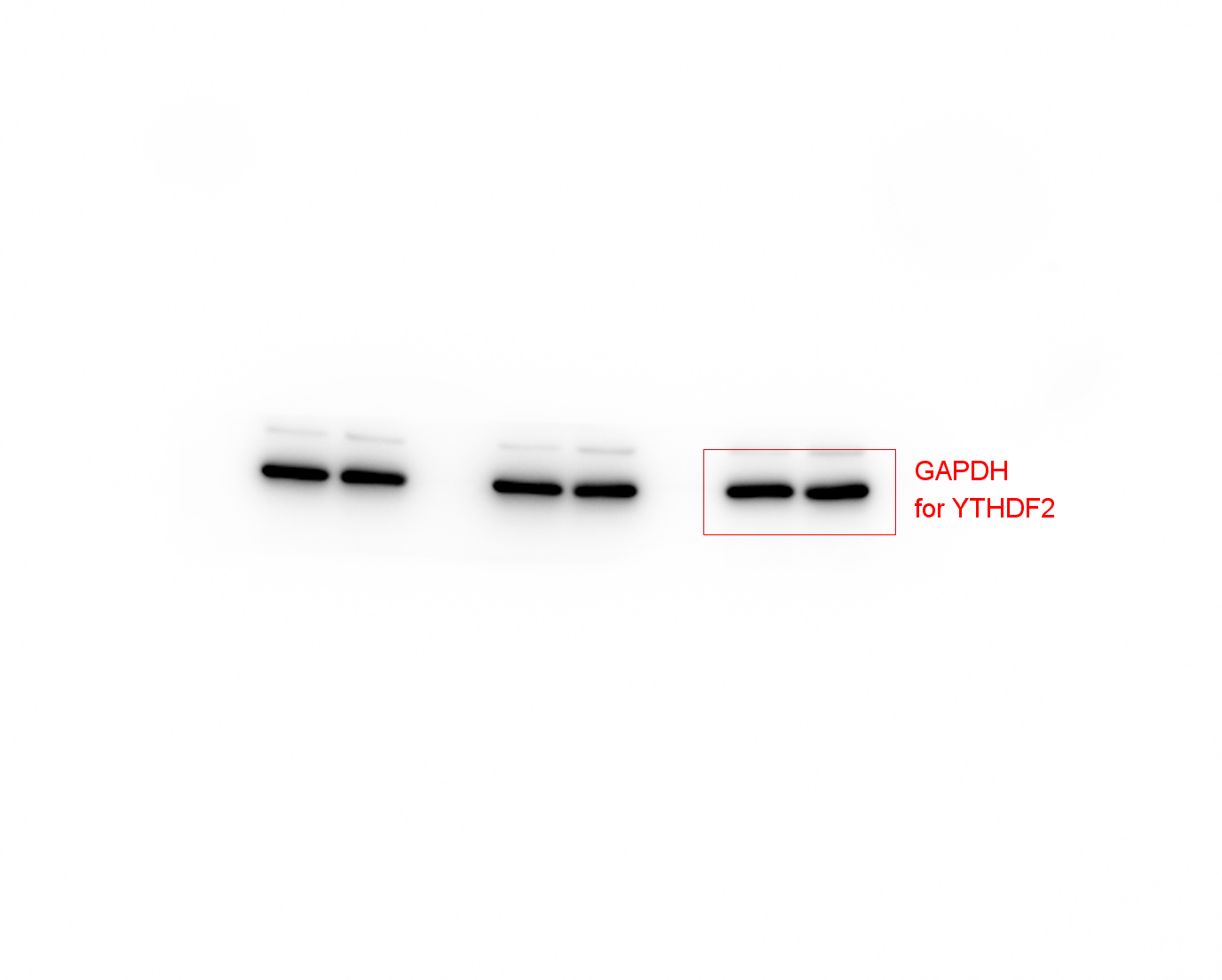

Supplement: Supplementary file 13 — Appendix Figures Source Data [file 44321_2024_25_MOESM13_ESM.zip › Appendix Figures/Appendix Figure S2/S2F/S2F GAPDH for YTHDF2 mark.Tif]

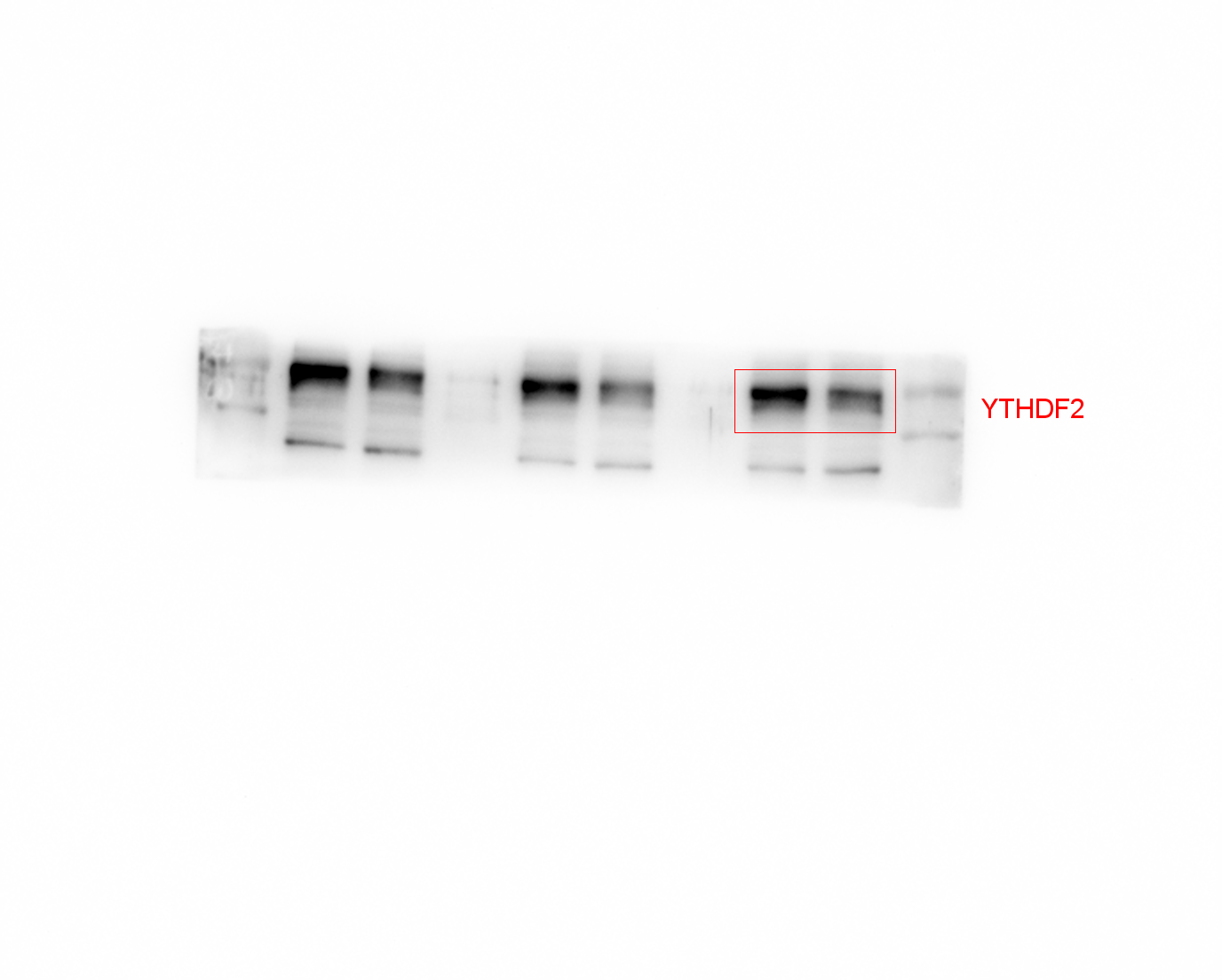

Supplement: Supplementary file 13 — Appendix Figures Source Data [file 44321_2024_25_MOESM13_ESM.zip › Appendix Figures/Appendix Figure S2/S2F/S2F YTHDF2 mark.Tif]

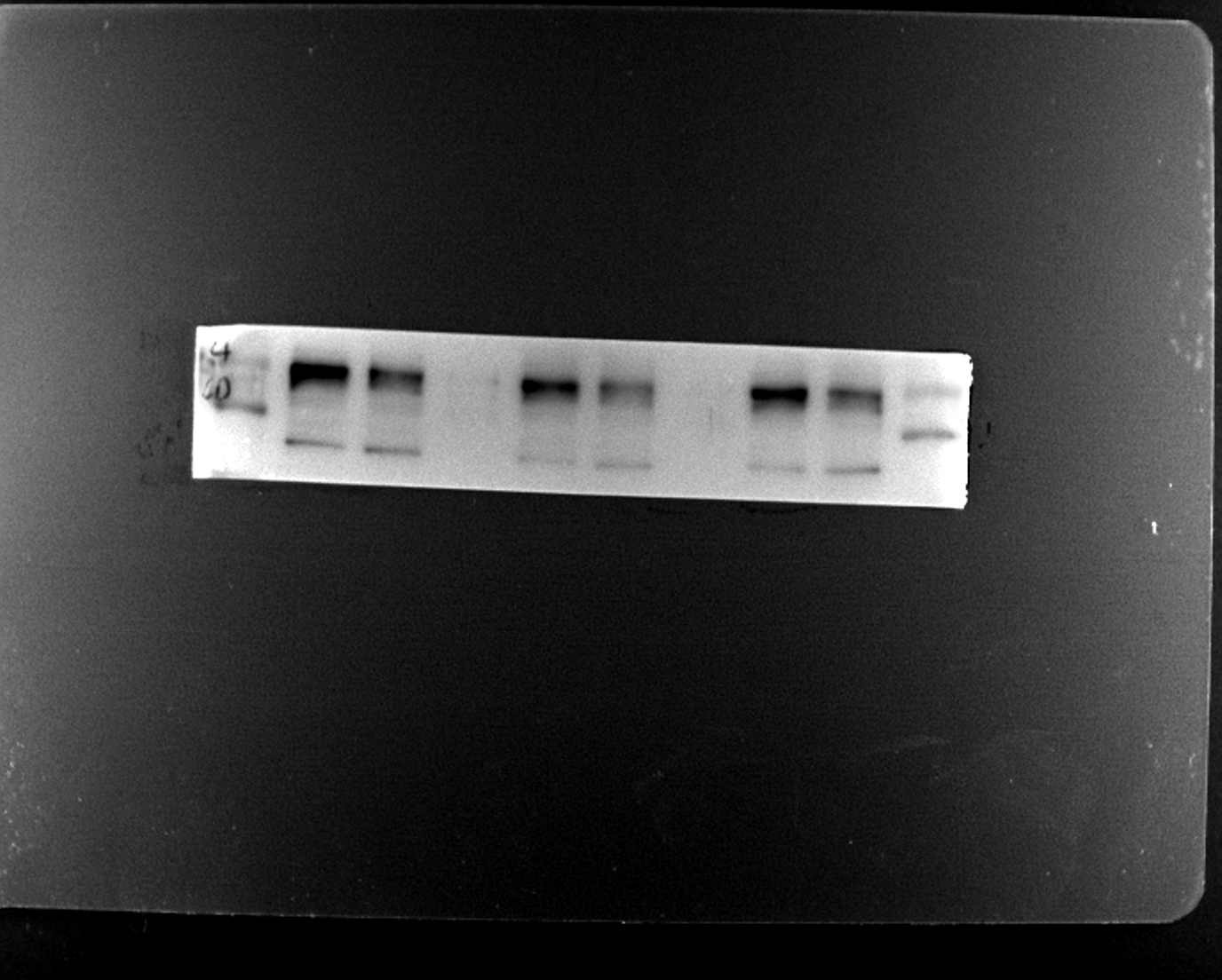

Supplement: Supplementary file 13 — Appendix Figures Source Data [file 44321_2024_25_MOESM13_ESM.zip › Appendix Figures/Appendix Figure S2/S2F/S2F YTHDF2.Tif]

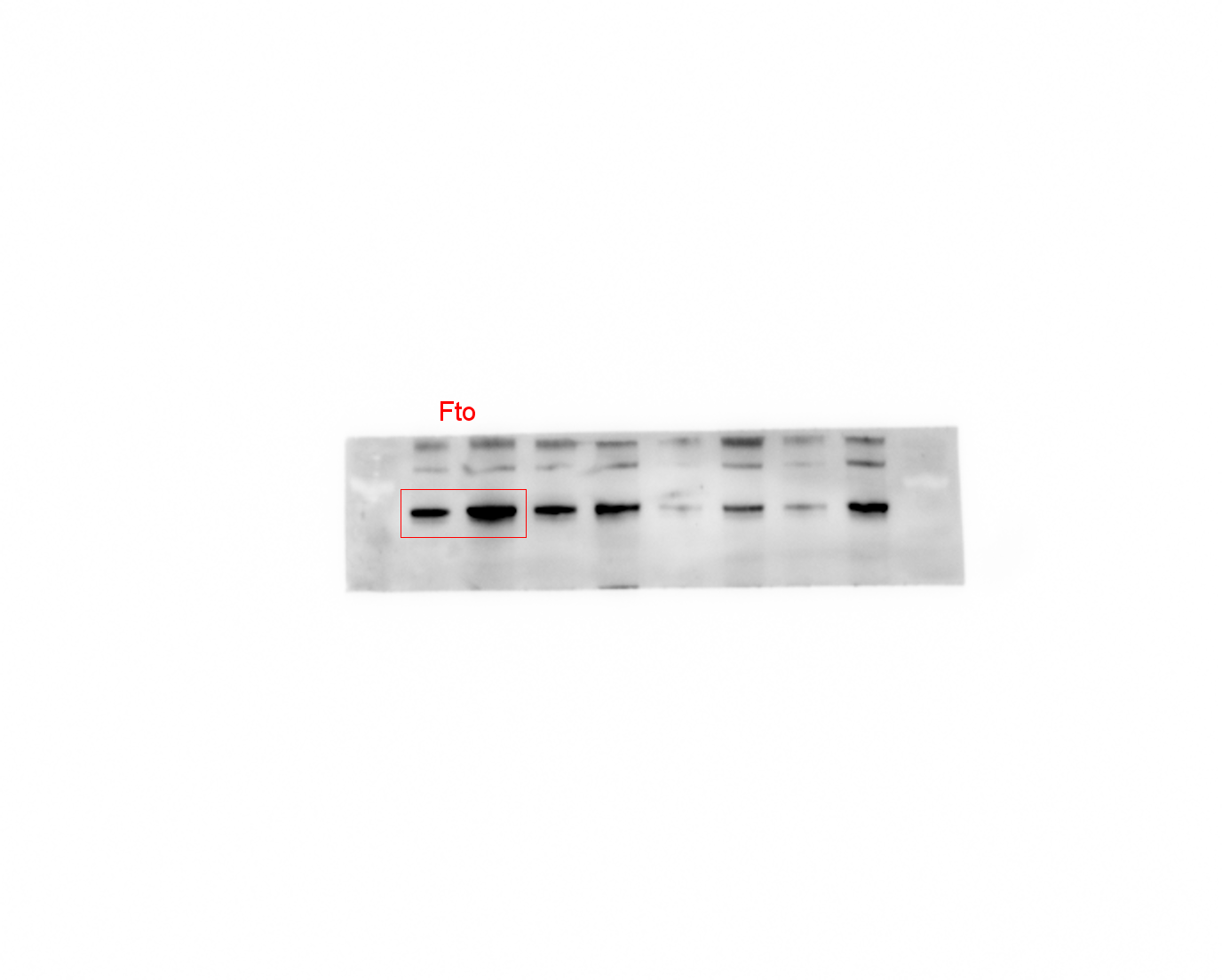

Supplement: Supplementary file 13 — Appendix Figures Source Data [file 44321_2024_25_MOESM13_ESM.zip › Appendix Figures/Appendix Figure S4/S4B/S4B Fto mark.Tif]

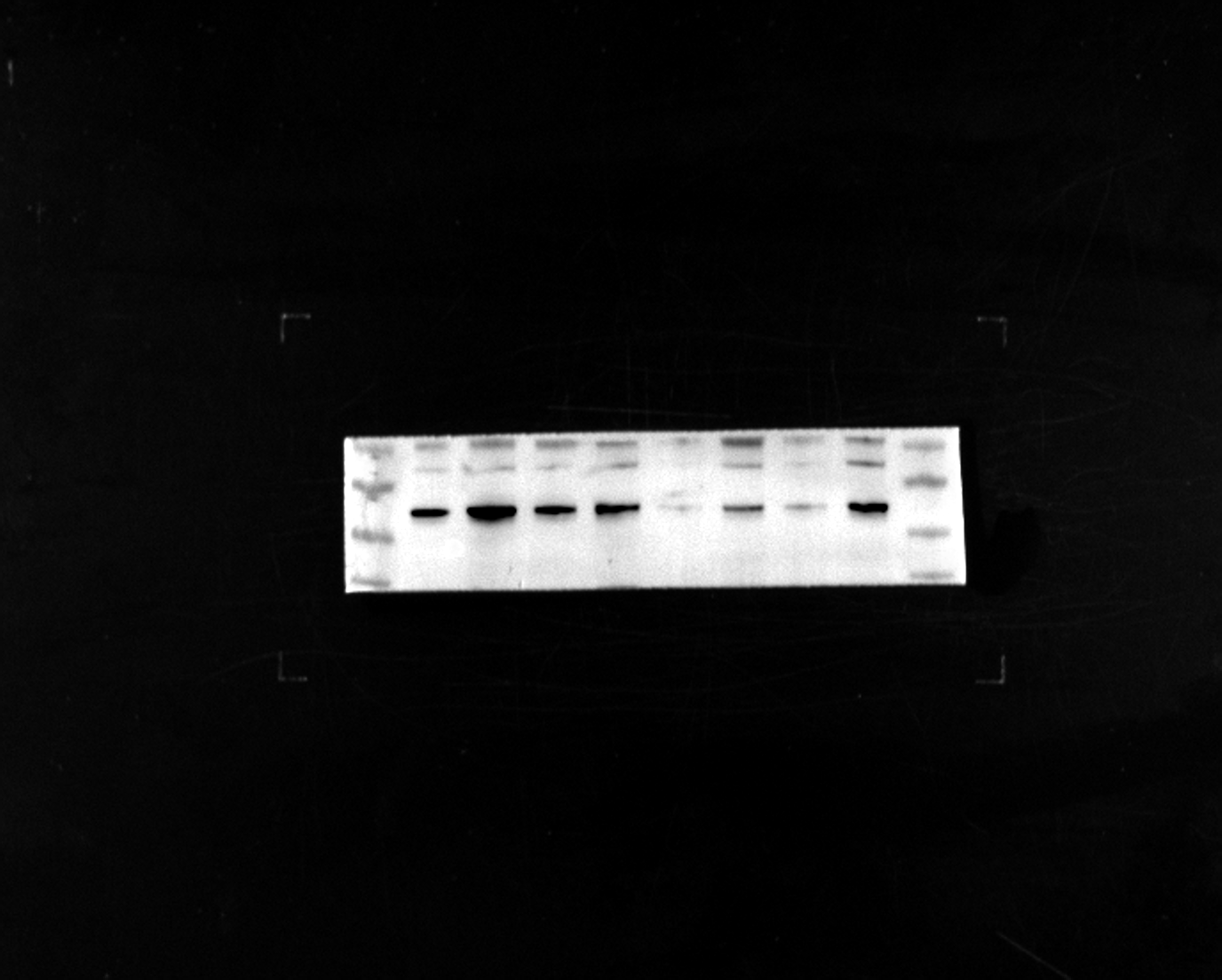

Supplement: Supplementary file 13 — Appendix Figures Source Data [file 44321_2024_25_MOESM13_ESM.zip › Appendix Figures/Appendix Figure S4/S4B/S4B Fto.Tif]

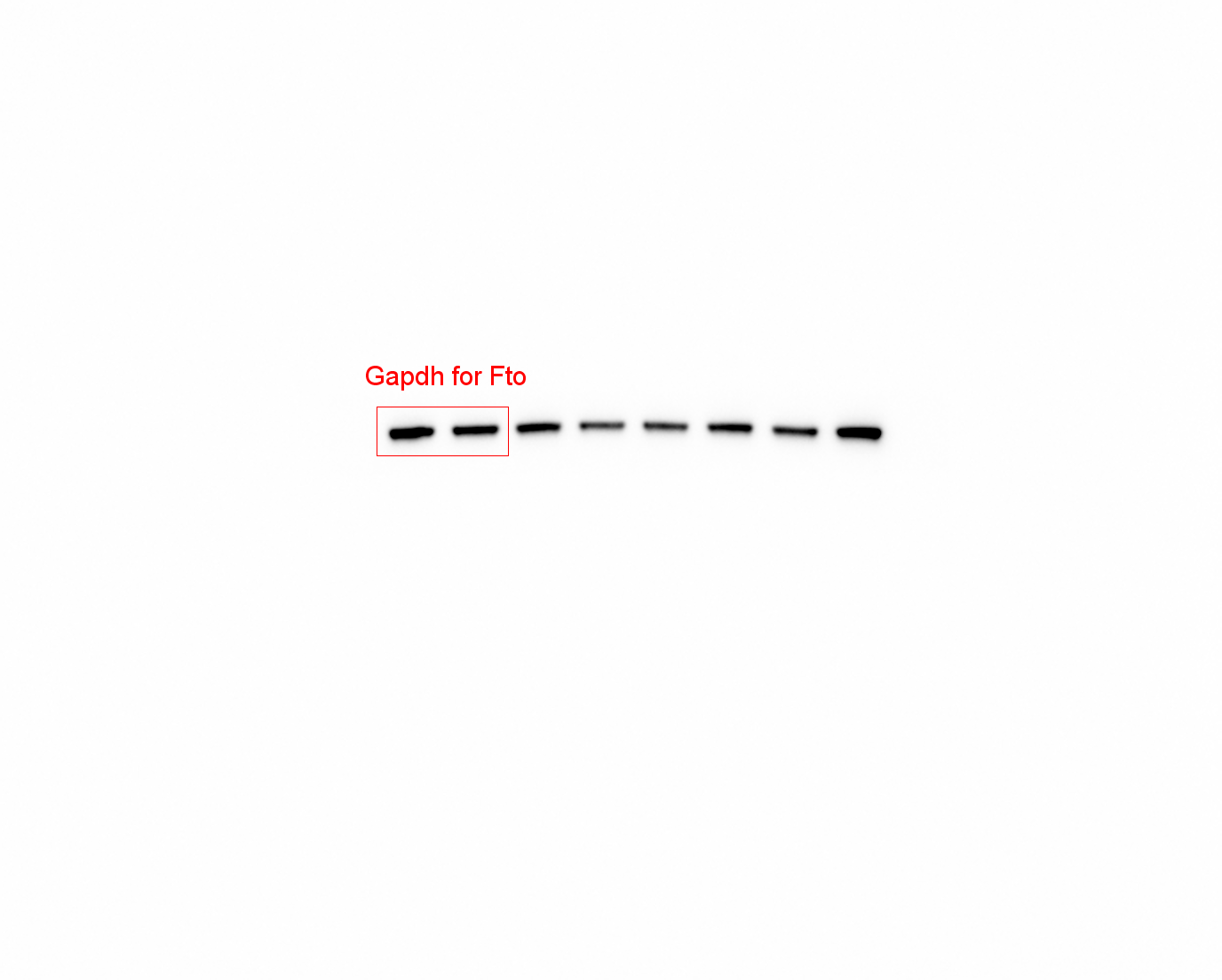

Supplement: Supplementary file 13 — Appendix Figures Source Data [file 44321_2024_25_MOESM13_ESM.zip › Appendix Figures/Appendix Figure S4/S4B/S4B Gapdh for Fto mark.Tif]

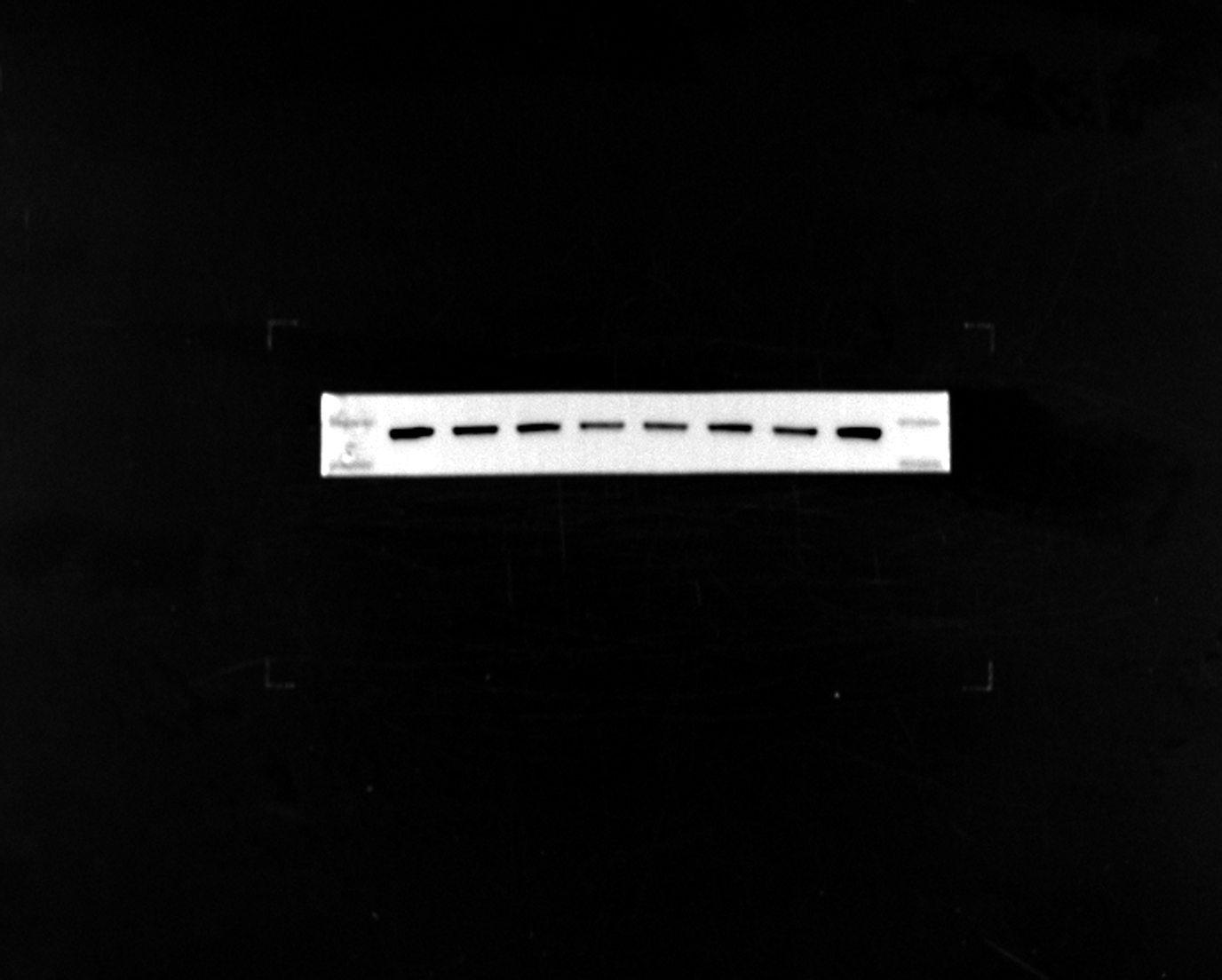

Supplement: Supplementary file 13 — Appendix Figures Source Data [file 44321_2024_25_MOESM13_ESM.zip › Appendix Figures/Appendix Figure S4/S4B/S4B Gapdh for Fto.Tif]

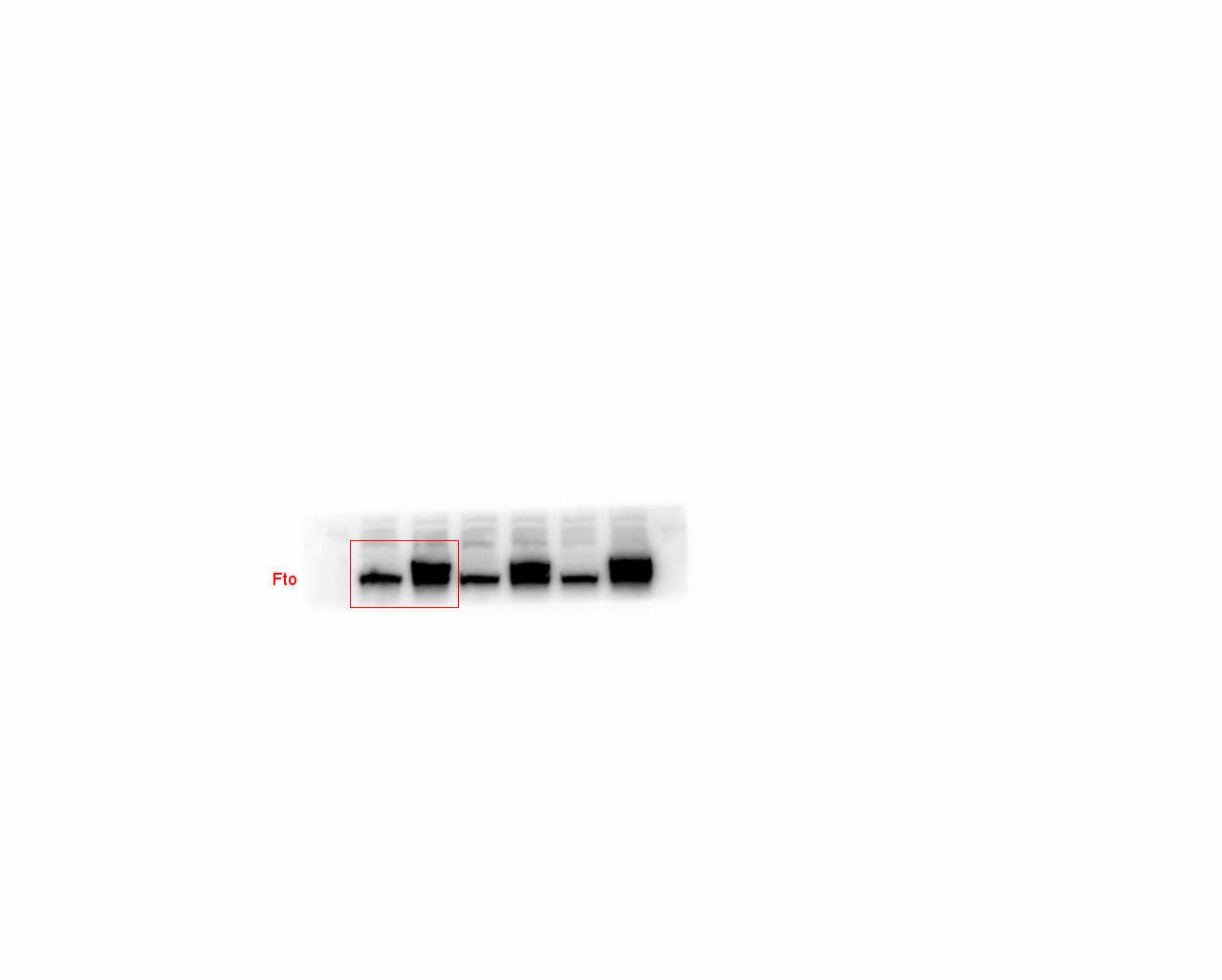

Supplement: Supplementary file 13 — Appendix Figures Source Data [file 44321_2024_25_MOESM13_ESM.zip › Appendix Figures/Appendix Figure S4/S4D/S4D Fto mark.Tif]

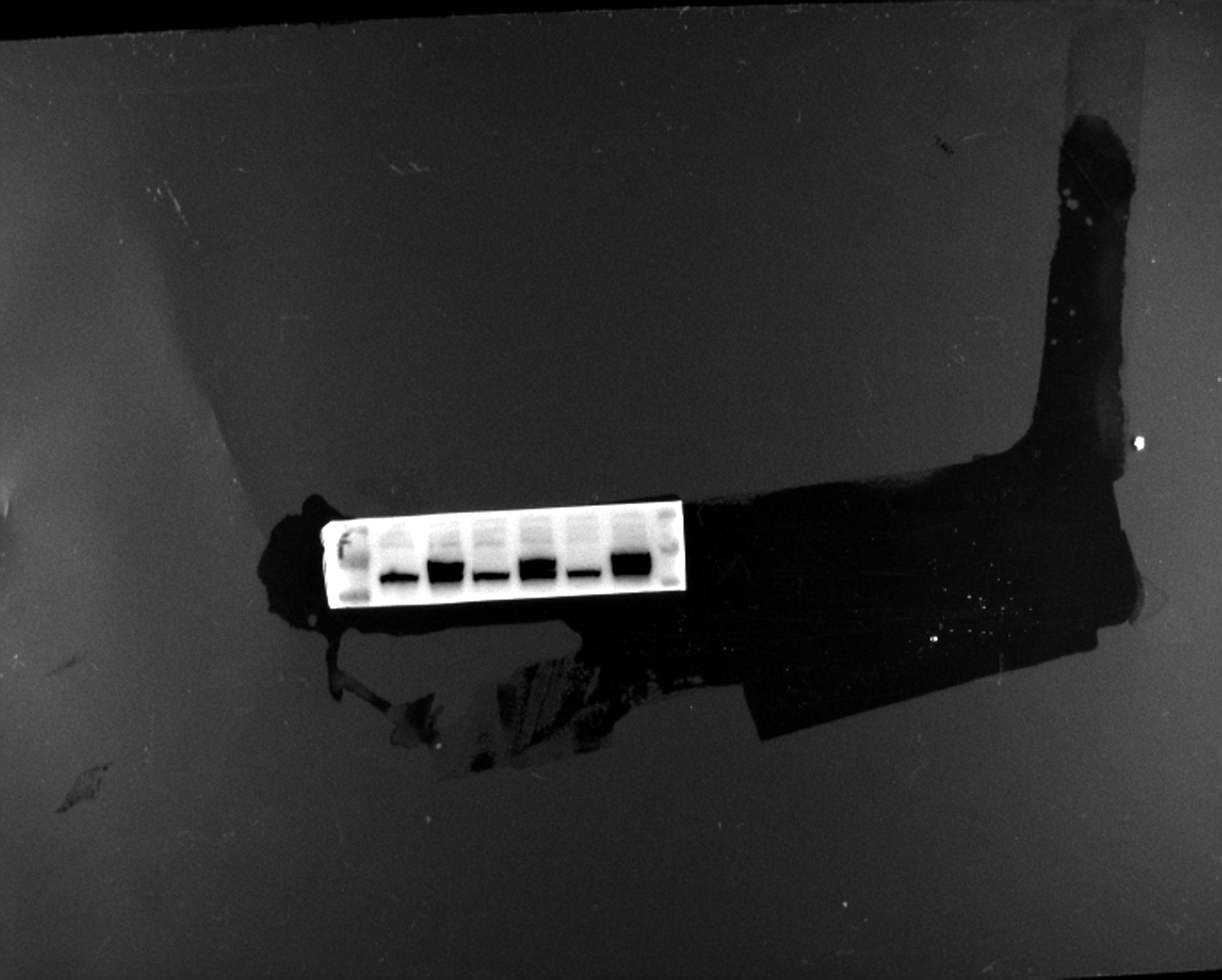

Supplement: Supplementary file 13 — Appendix Figures Source Data [file 44321_2024_25_MOESM13_ESM.zip › Appendix Figures/Appendix Figure S4/S4D/S4D Fto.Tif]

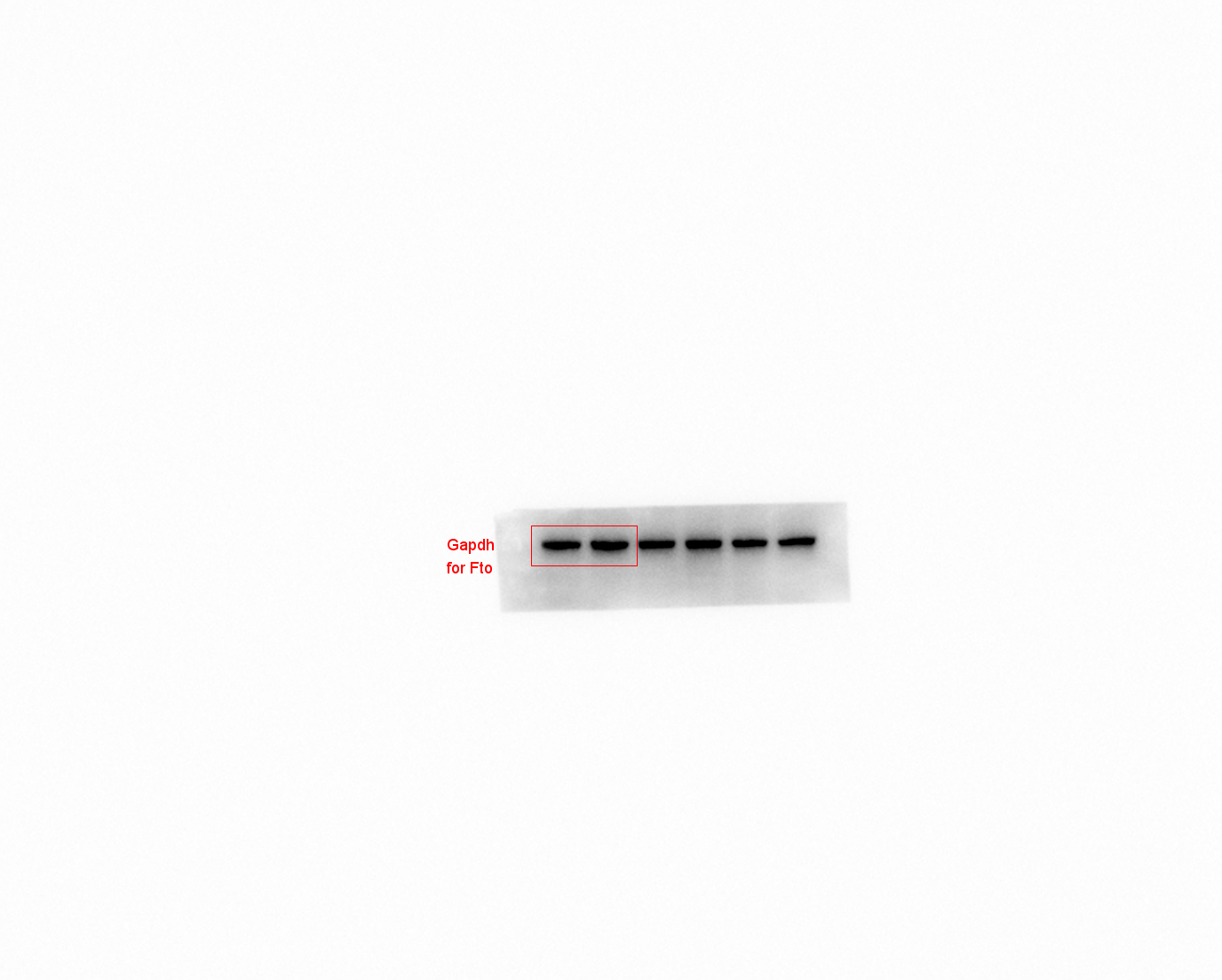

Supplement: Supplementary file 13 — Appendix Figures Source Data [file 44321_2024_25_MOESM13_ESM.zip › Appendix Figures/Appendix Figure S4/S4D/S4D Gapdh for Fto mark.Tif]

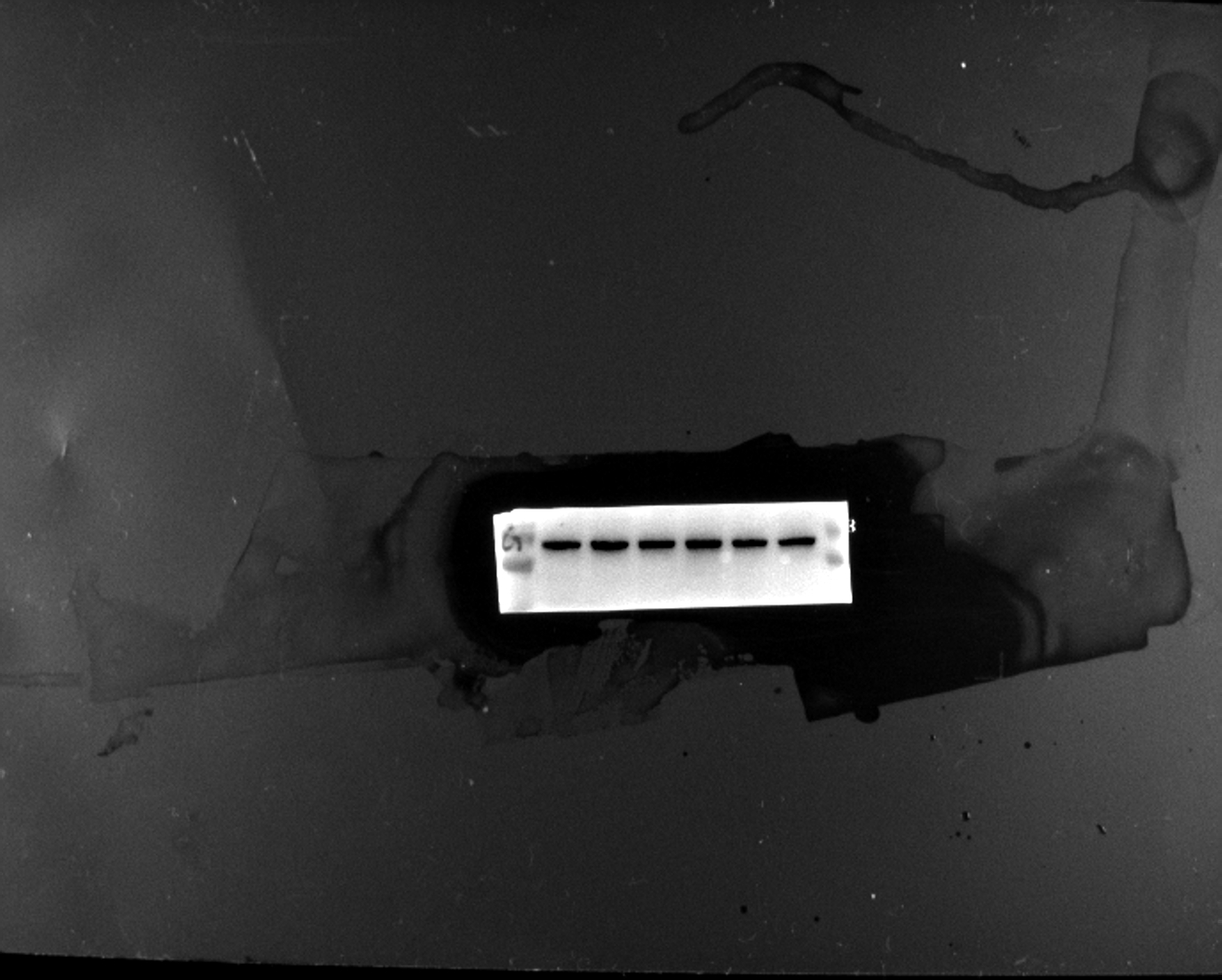

Supplement: Supplementary file 13 — Appendix Figures Source Data [file 44321_2024_25_MOESM13_ESM.zip › Appendix Figures/Appendix Figure S4/S4D/S4D Gapdh for Fto.Tif]

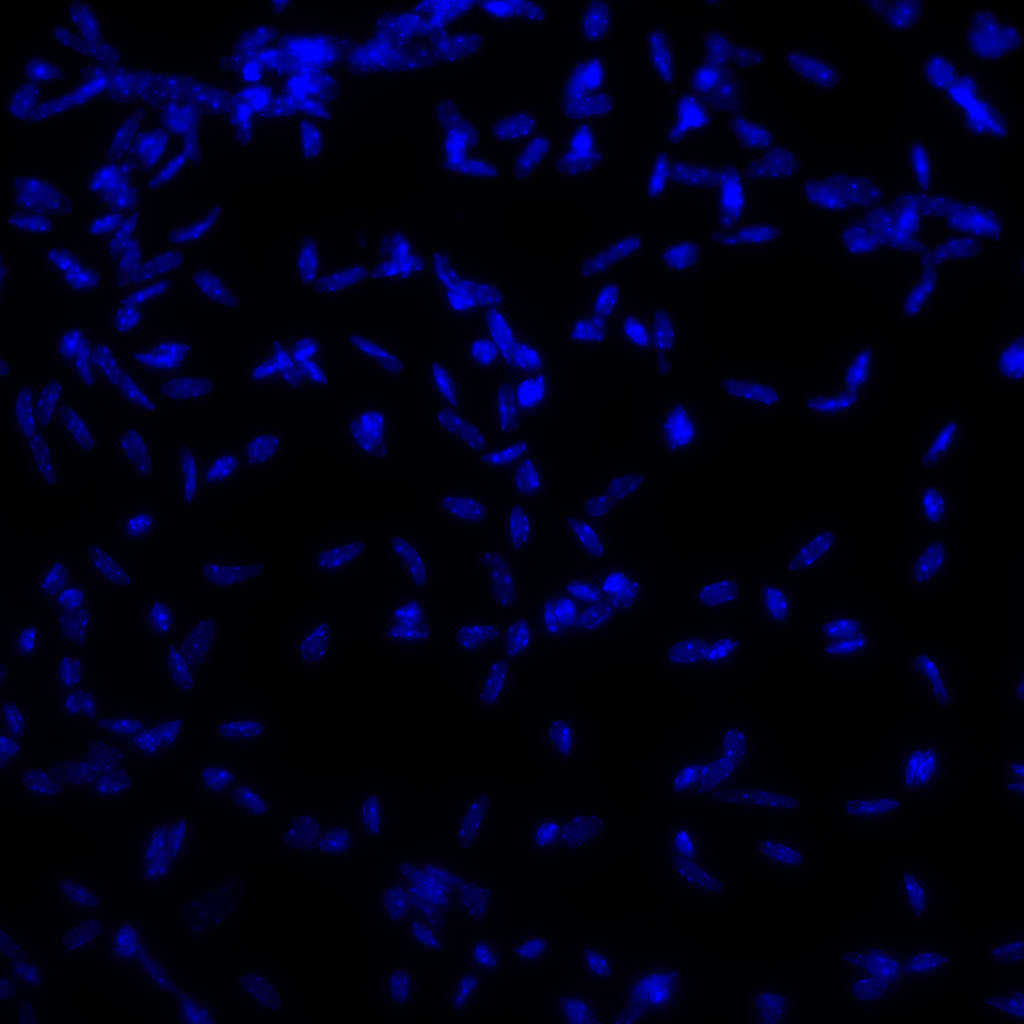

Supplement: Supplementary file 13 — Appendix Figures Source Data [file 44321_2024_25_MOESM13_ESM.zip › Appendix Figures/Appendix Figure S4/S4E/S4E AAV-blank DAPI.tif]

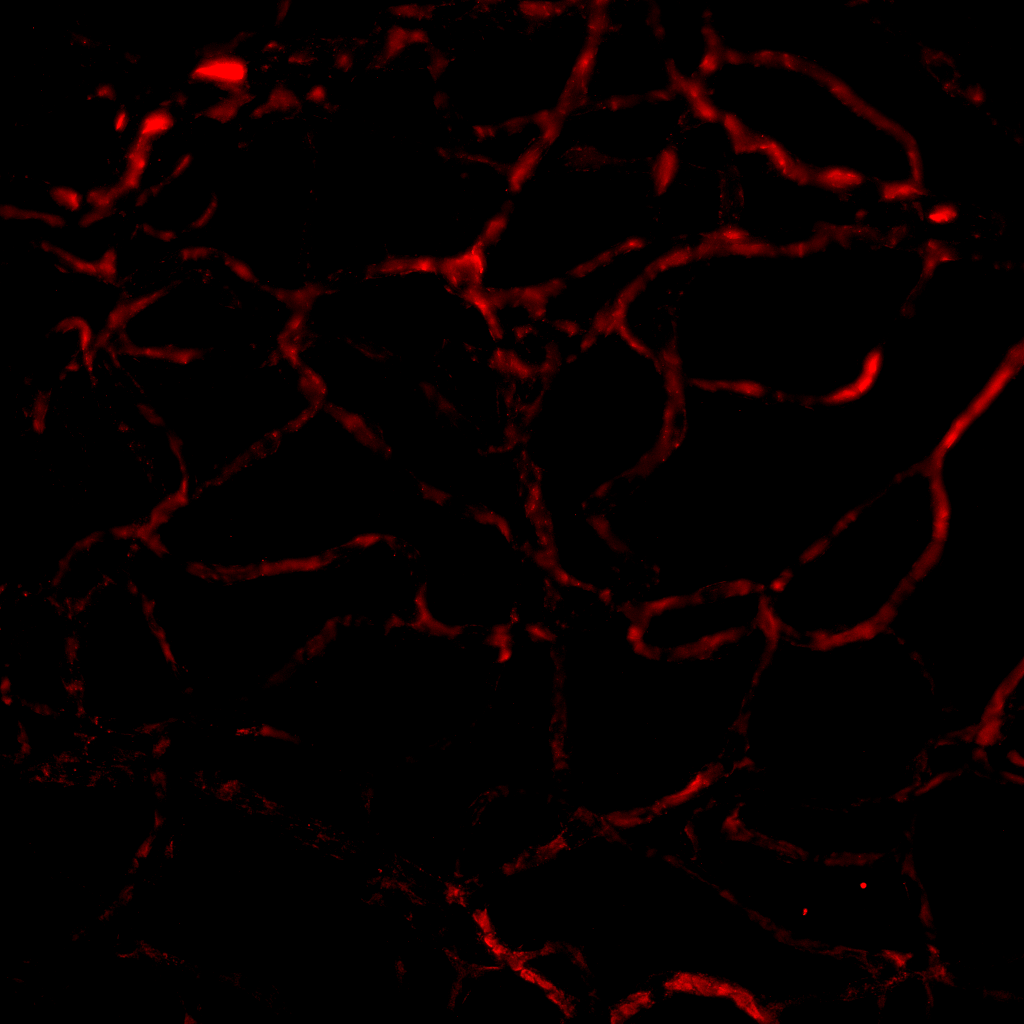

Supplement: Supplementary file 13 — Appendix Figures Source Data [file 44321_2024_25_MOESM13_ESM.zip › Appendix Figures/Appendix Figure S4/S4E/S4E AAV-blank FTO.tif]

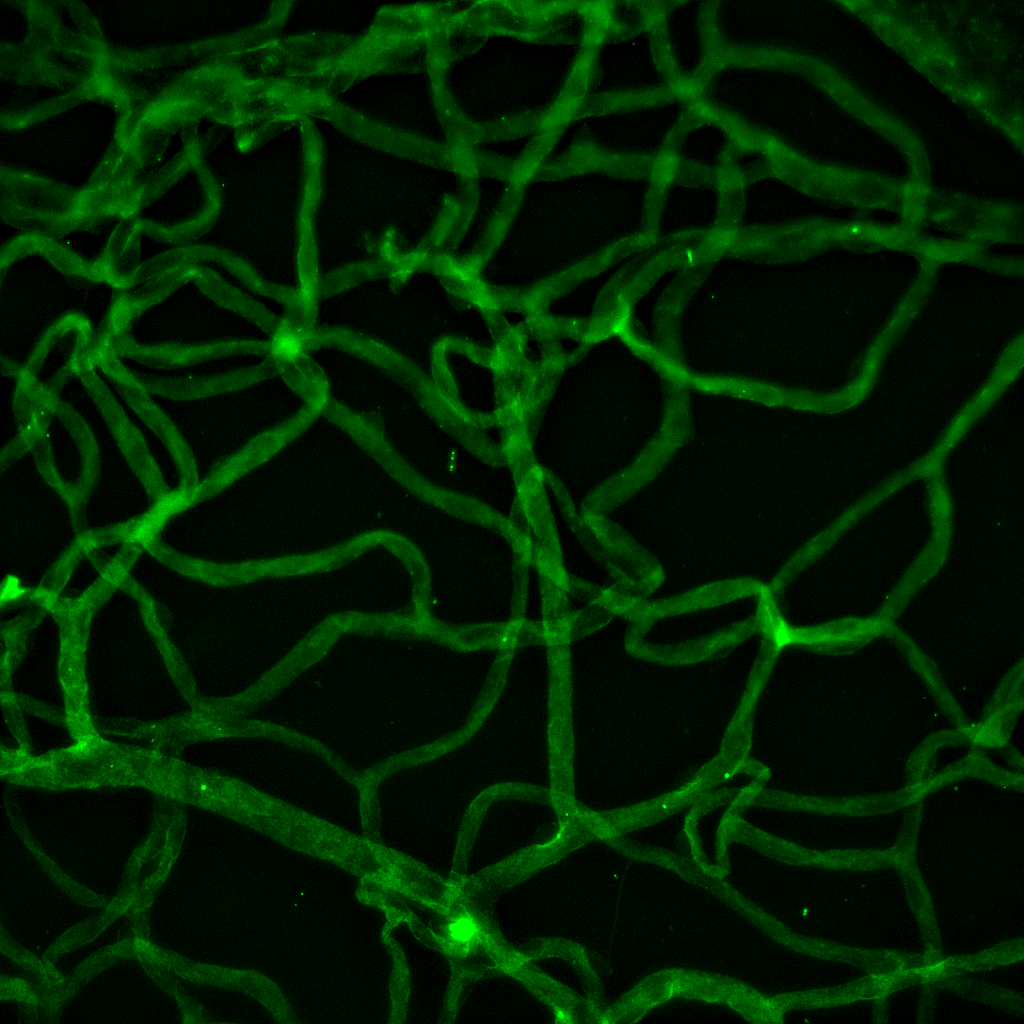

Supplement: Supplementary file 13 — Appendix Figures Source Data [file 44321_2024_25_MOESM13_ESM.zip › Appendix Figures/Appendix Figure S4/S4E/S4E AAV-blank IB4.tif]

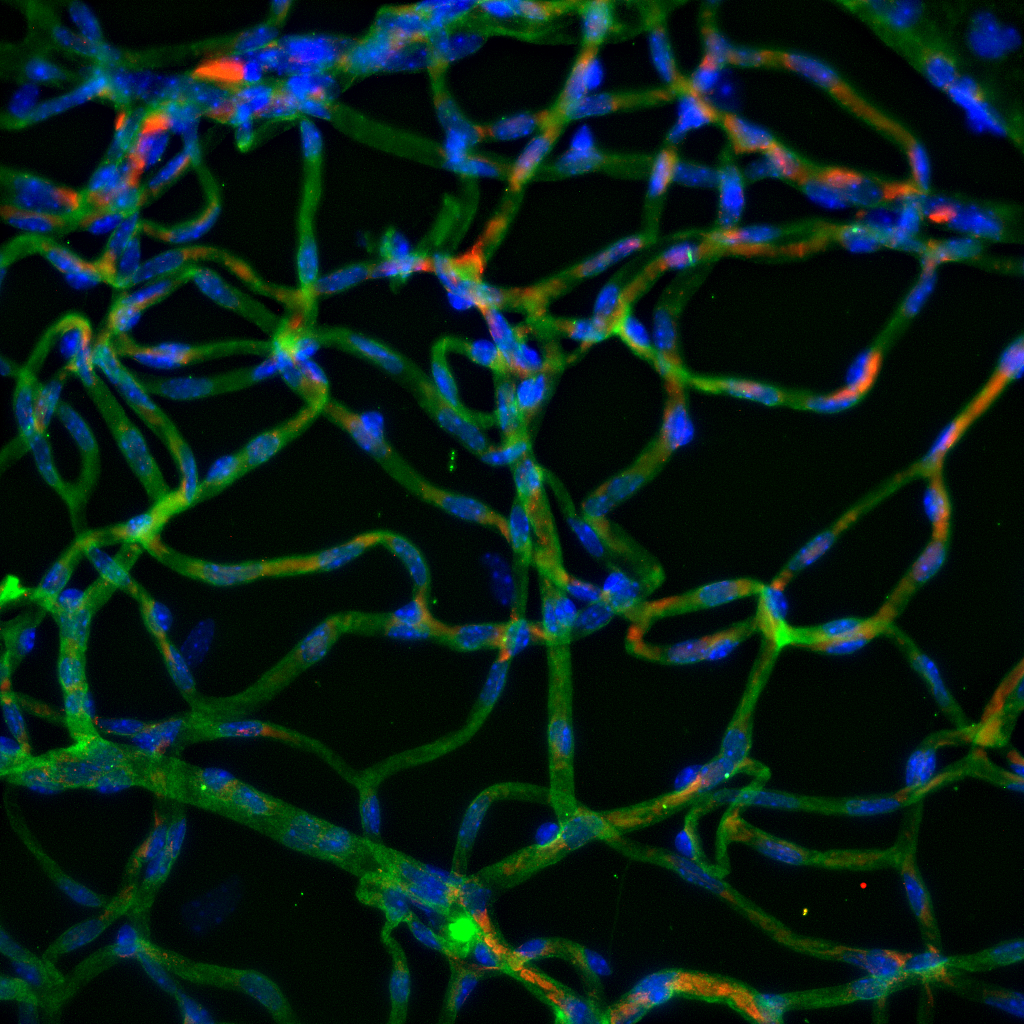

Supplement: Supplementary file 13 — Appendix Figures Source Data [file 44321_2024_25_MOESM13_ESM.zip › Appendix Figures/Appendix Figure S4/S4E/S4E AAV-blank Merge.tif]

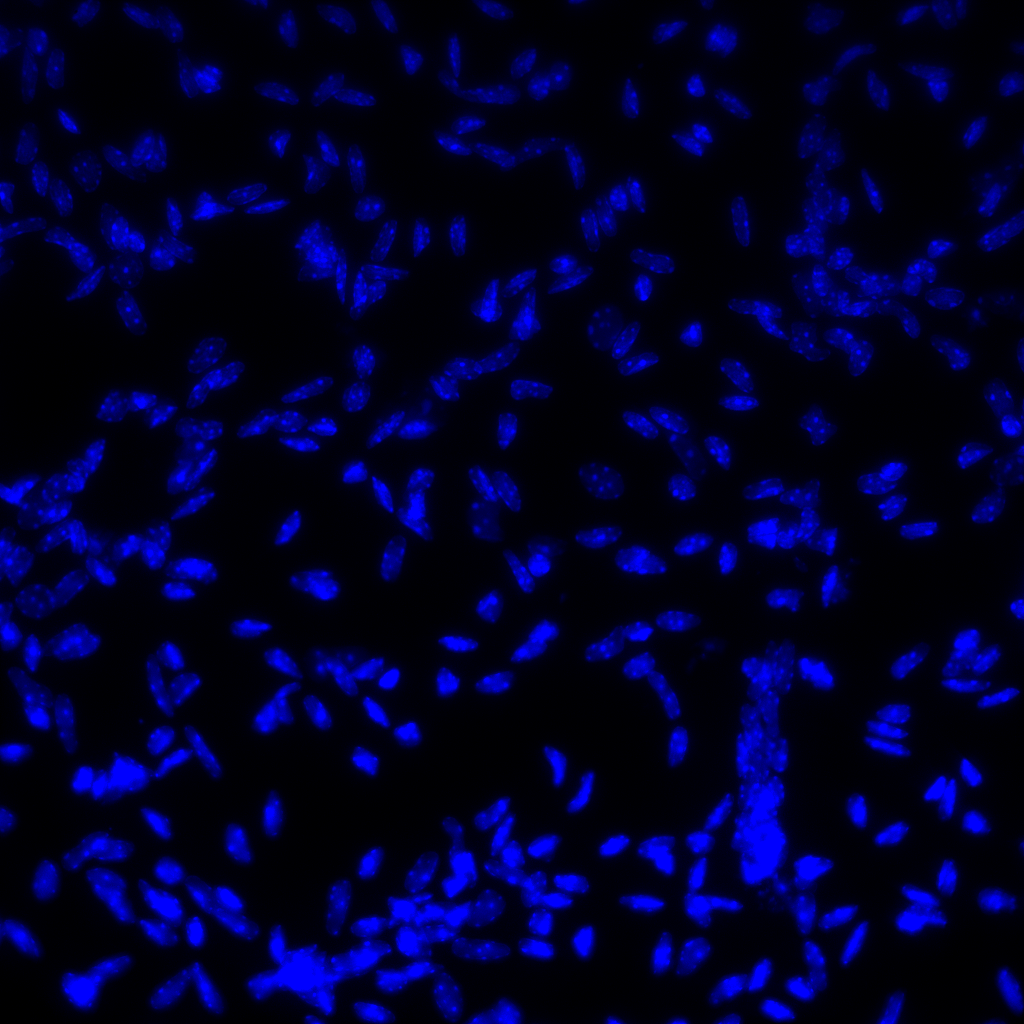

Supplement: Supplementary file 13 — Appendix Figures Source Data [file 44321_2024_25_MOESM13_ESM.zip › Appendix Figures/Appendix Figure S4/S4E/S4E AAV-Fto DAPI.tif]

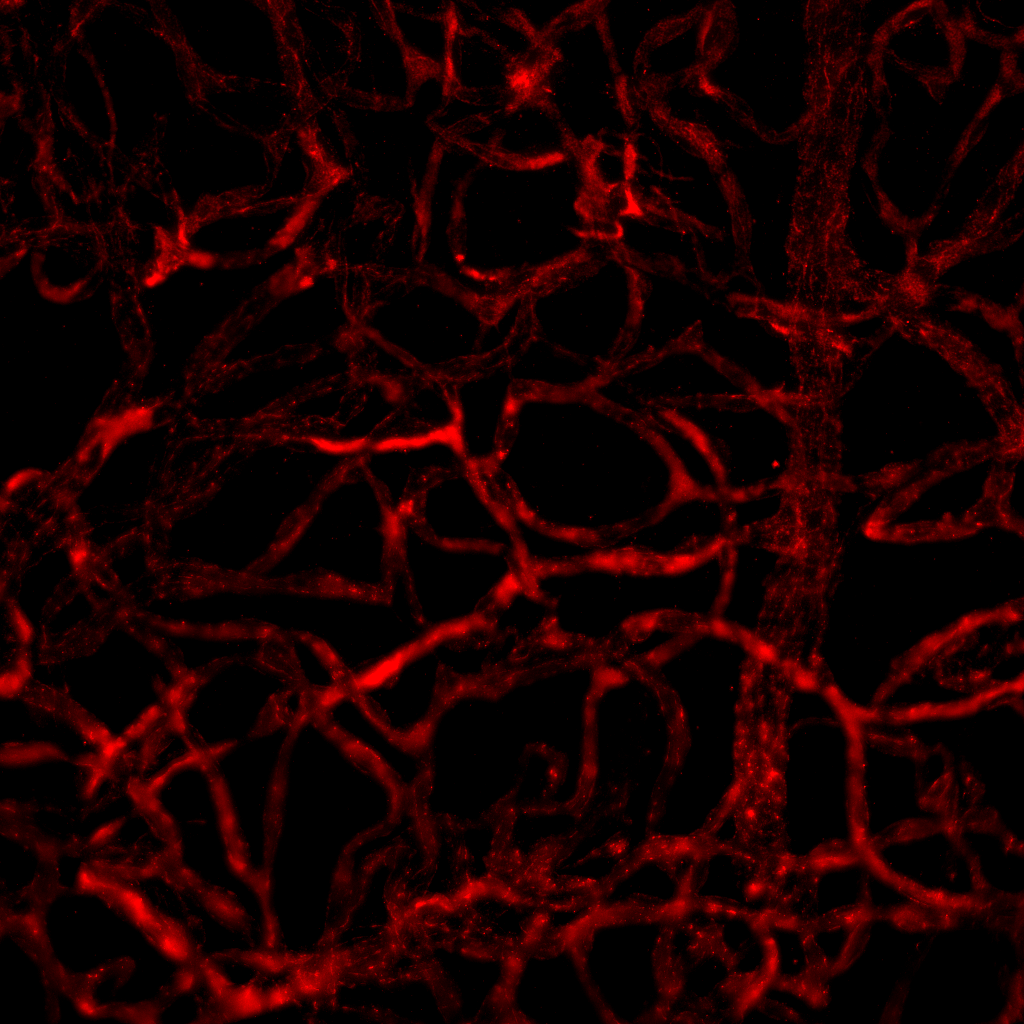

Supplement: Supplementary file 13 — Appendix Figures Source Data [file 44321_2024_25_MOESM13_ESM.zip › Appendix Figures/Appendix Figure S4/S4E/S4E AAV-Fto FTO.tif]

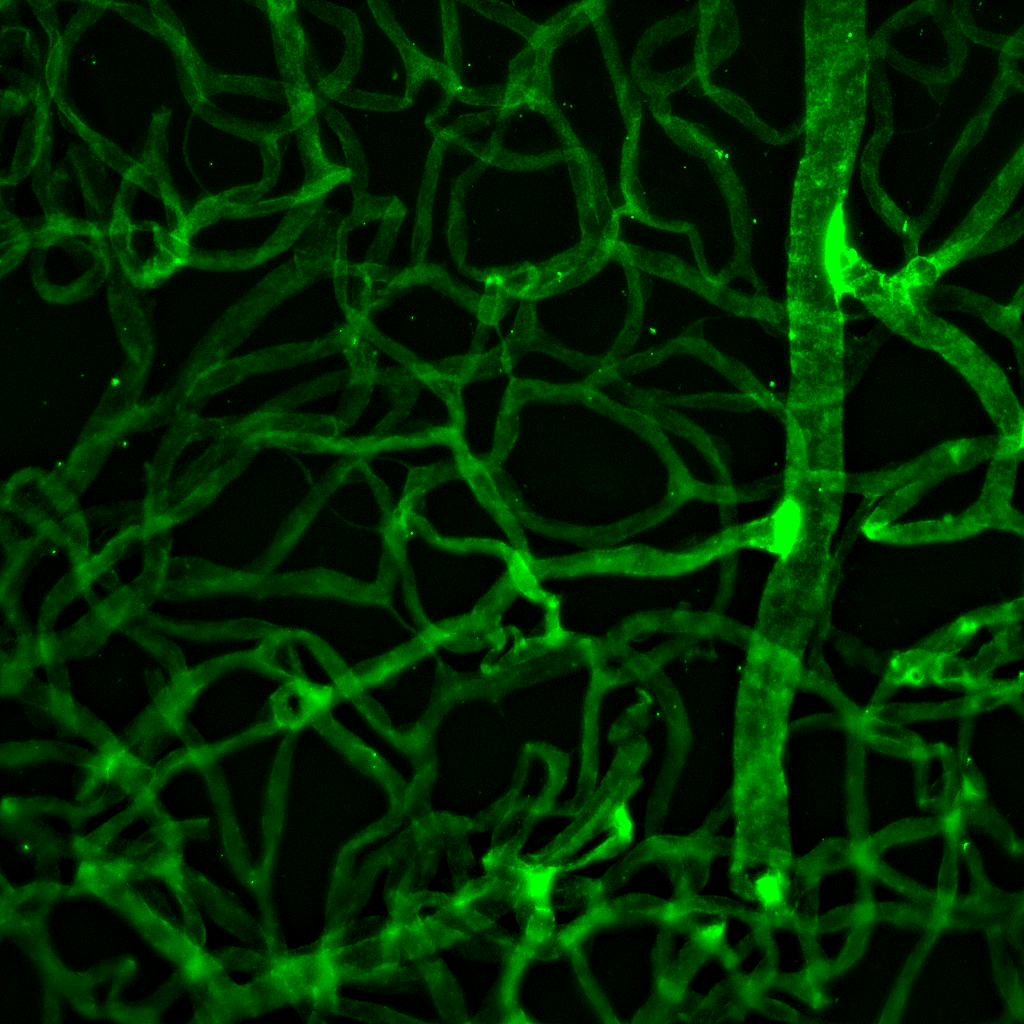

Supplement: Supplementary file 13 — Appendix Figures Source Data [file 44321_2024_25_MOESM13_ESM.zip › Appendix Figures/Appendix Figure S4/S4E/S4E AAV-Fto IB4.tif]

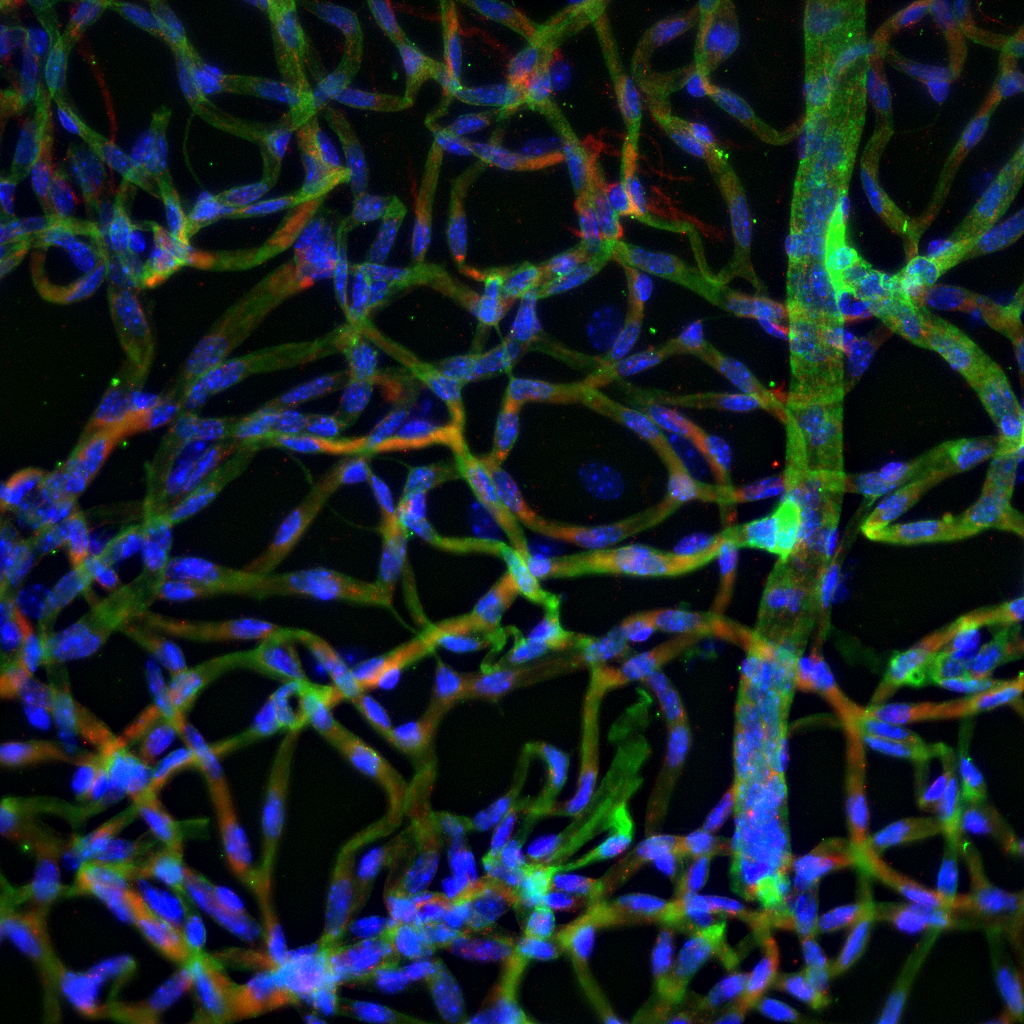

Supplement: Supplementary file 13 — Appendix Figures Source Data [file 44321_2024_25_MOESM13_ESM.zip › Appendix Figures/Appendix Figure S4/S4E/S4E AAV-Fto Merge.tif]

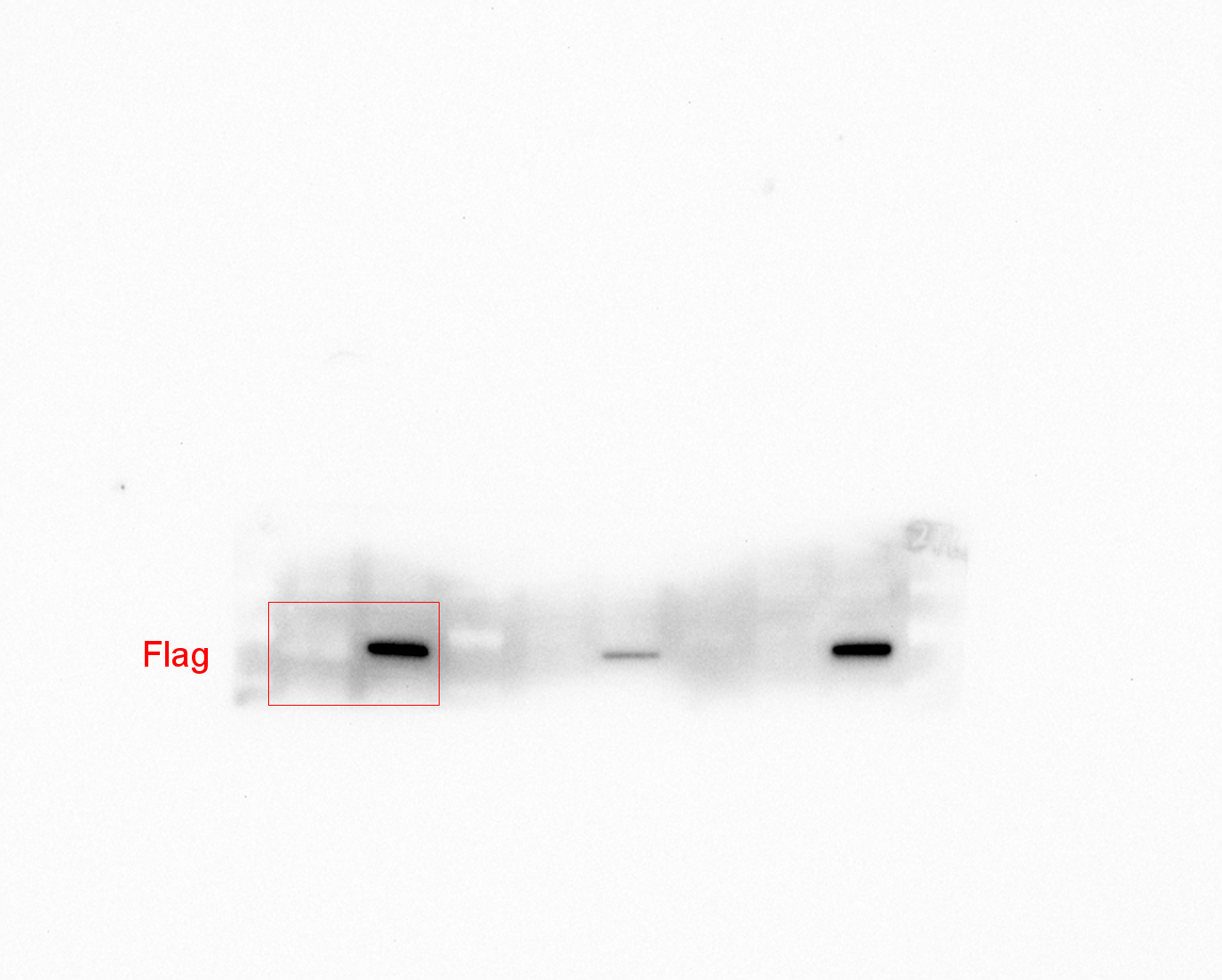

Supplement: Supplementary file 13 — Appendix Figures Source Data [file 44321_2024_25_MOESM13_ESM.zip › Appendix Figures/Appendix Figure S4/S4F/S4F Flag mark.tiff]

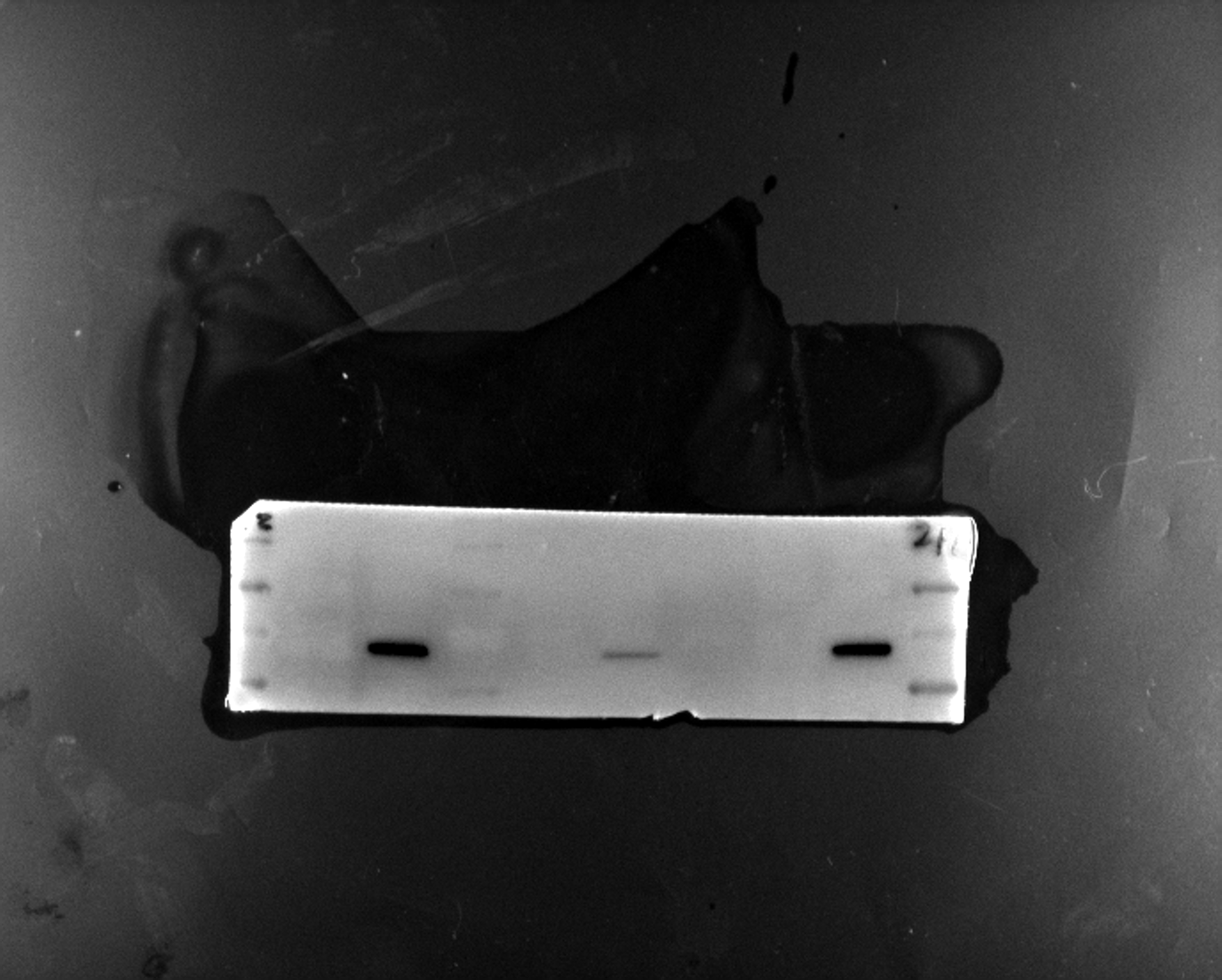

Supplement: Supplementary file 13 — Appendix Figures Source Data [file 44321_2024_25_MOESM13_ESM.zip › Appendix Figures/Appendix Figure S4/S4F/S4F Flag.tiff]

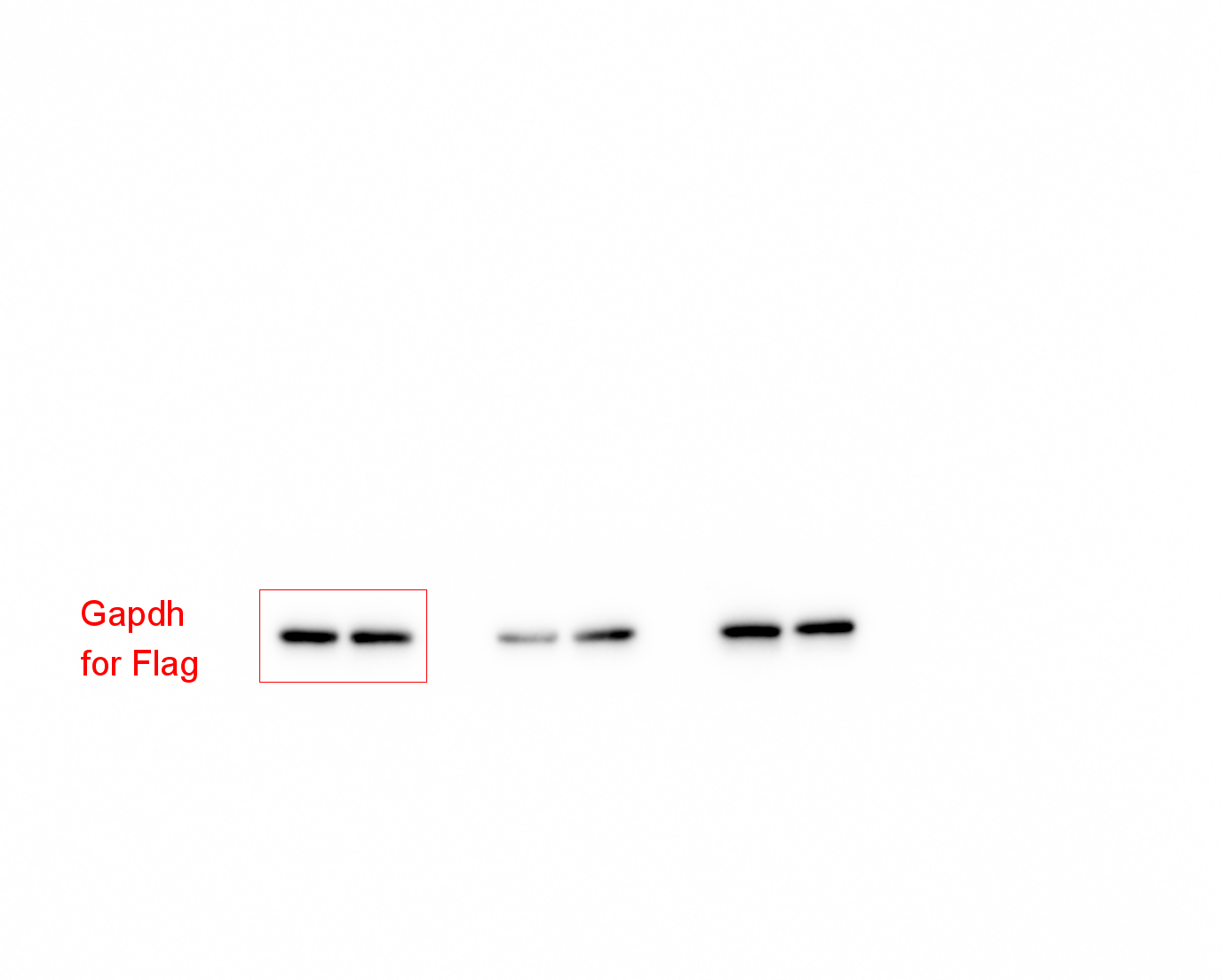

Supplement: Supplementary file 13 — Appendix Figures Source Data [file 44321_2024_25_MOESM13_ESM.zip › Appendix Figures/Appendix Figure S4/S4F/S4F Gapdh for Flag mark.tiff]

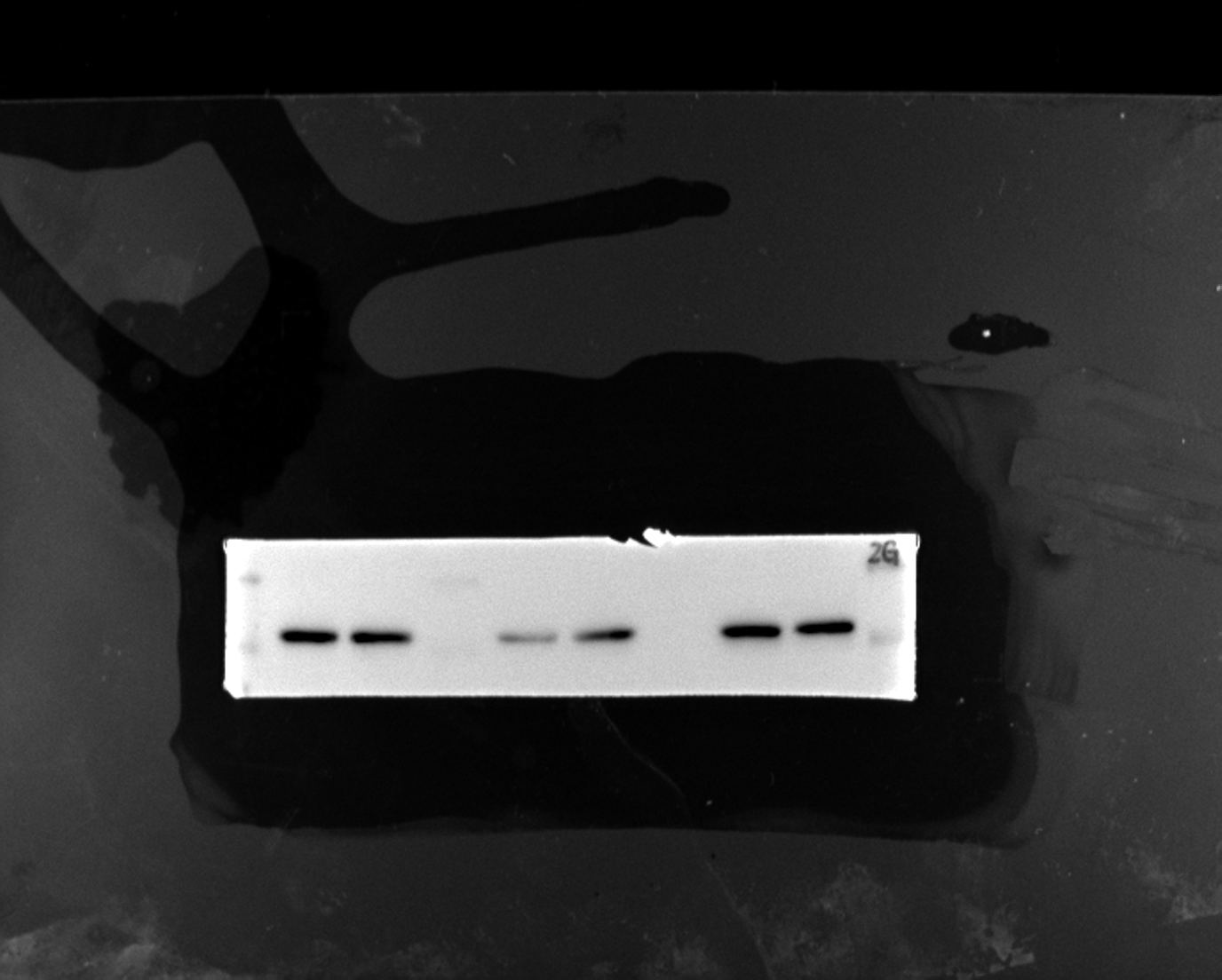

Supplement: Supplementary file 13 — Appendix Figures Source Data [file 44321_2024_25_MOESM13_ESM.zip › Appendix Figures/Appendix Figure S4/S4F/S4F Gapdh for Flag.tiff]

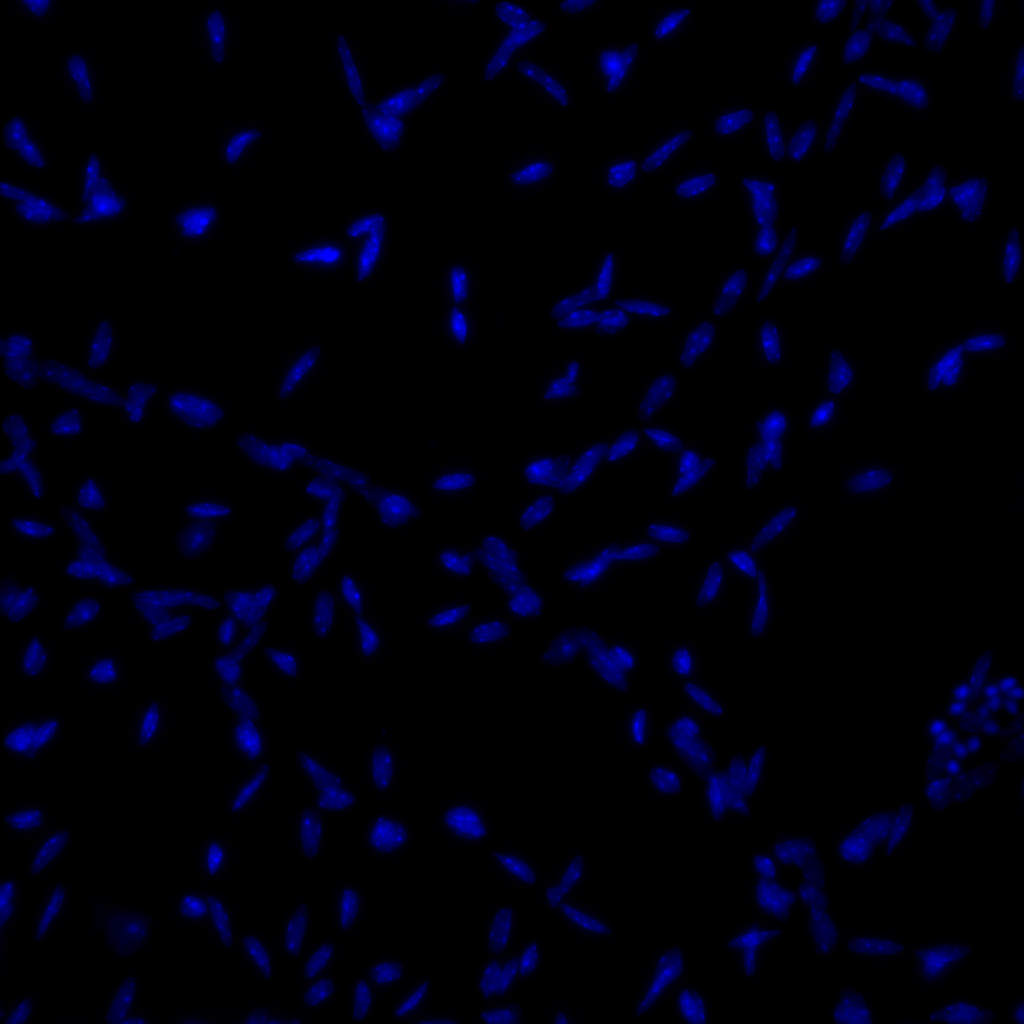

Supplement: Supplementary file 13 — Appendix Figures Source Data [file 44321_2024_25_MOESM13_ESM.zip › Appendix Figures/Appendix Figure S4/S4G/S4G AAV-blank DAPI.tif]

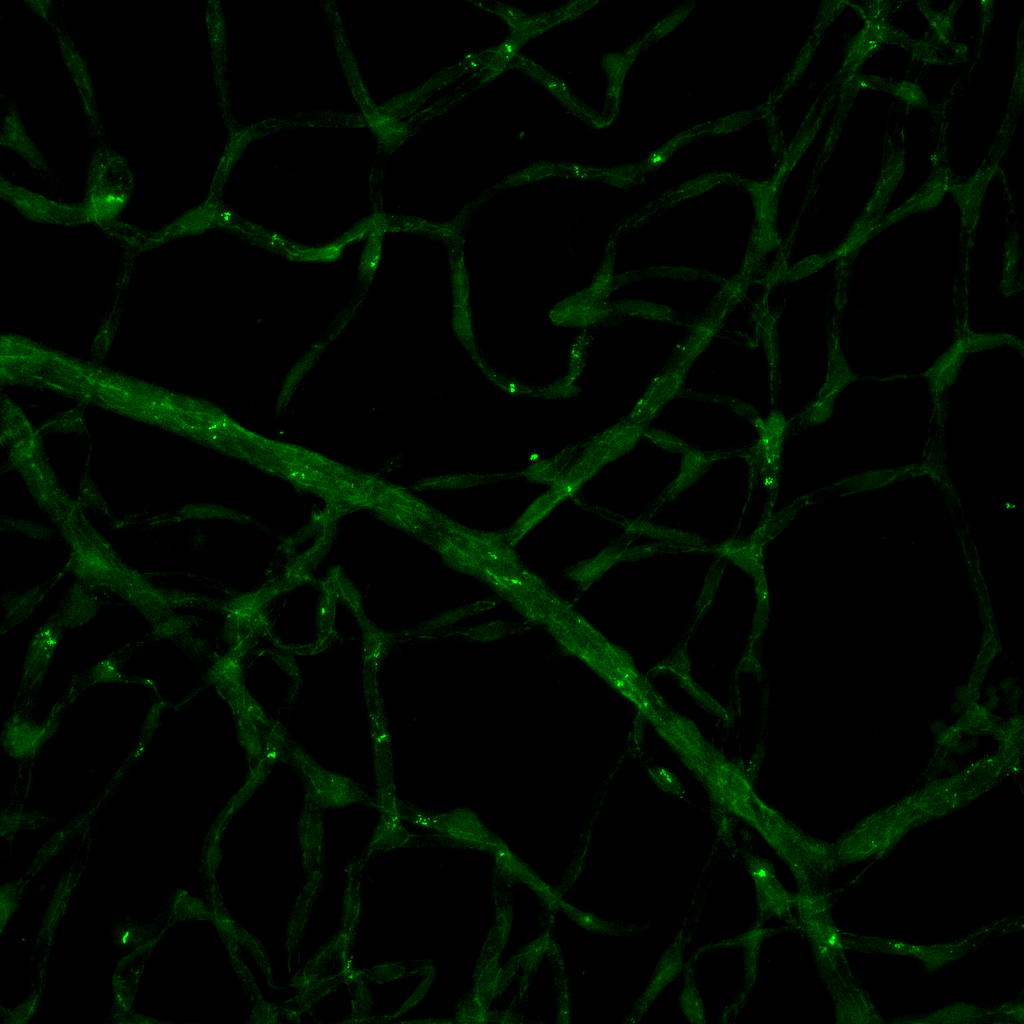

Supplement: Supplementary file 13 — Appendix Figures Source Data [file 44321_2024_25_MOESM13_ESM.zip › Appendix Figures/Appendix Figure S4/S4G/S4G AAV-blank FLAG.tif]

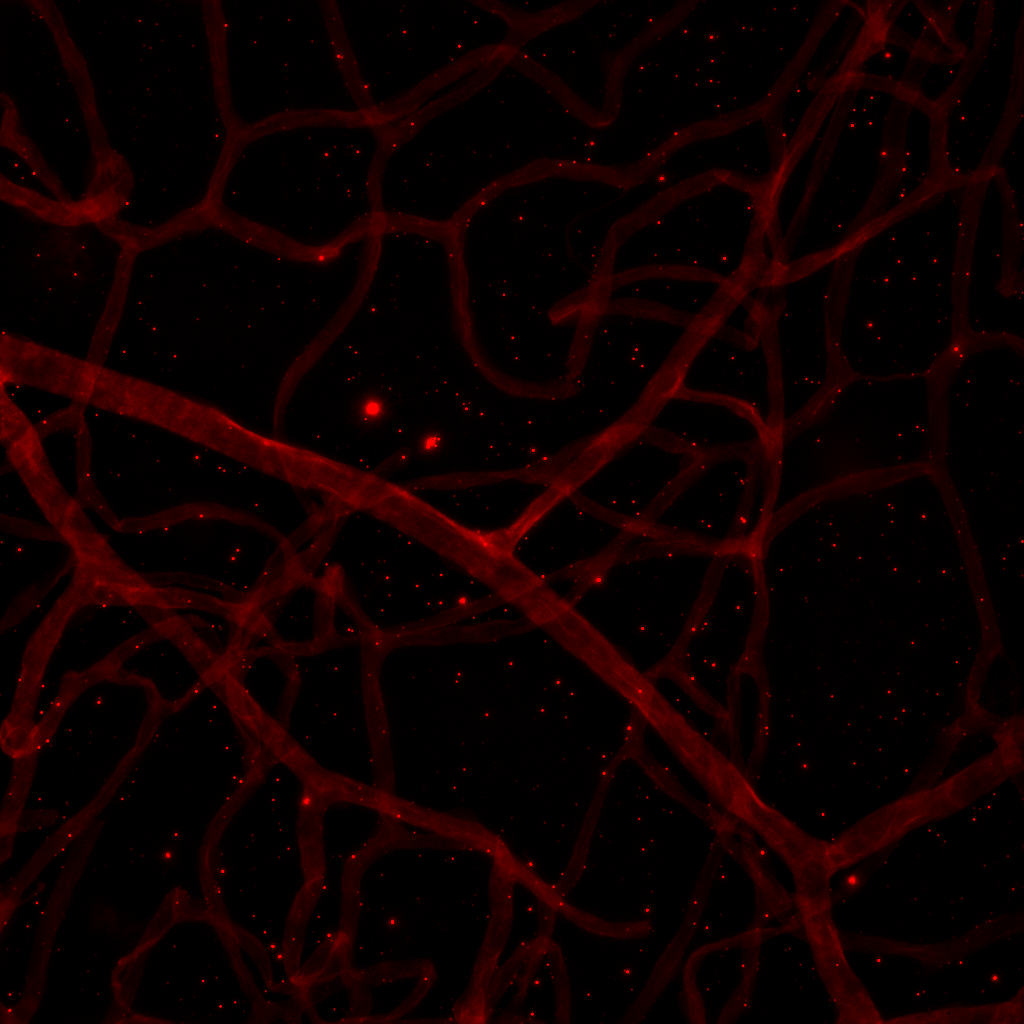

Supplement: Supplementary file 13 — Appendix Figures Source Data [file 44321_2024_25_MOESM13_ESM.zip › Appendix Figures/Appendix Figure S4/S4G/S4G AAV-blank IB4.tif]

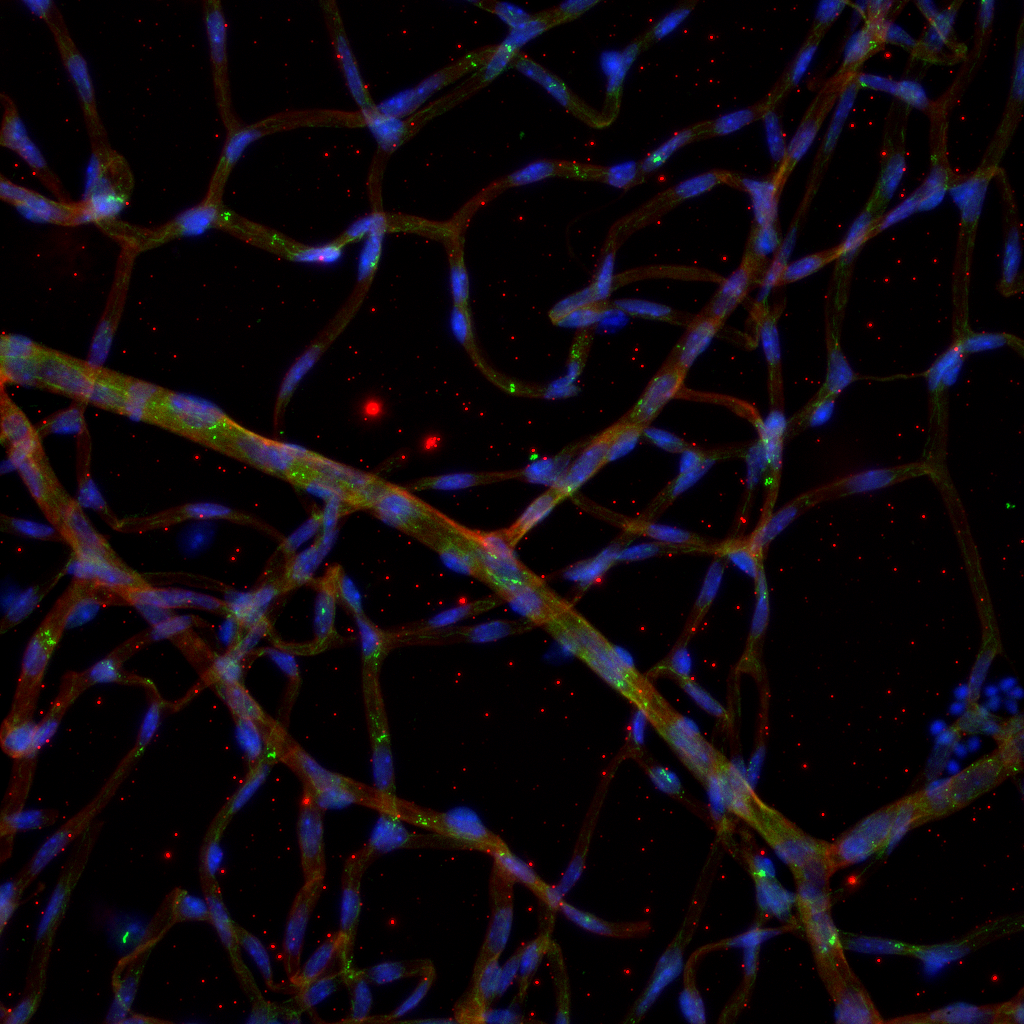

Supplement: Supplementary file 13 — Appendix Figures Source Data [file 44321_2024_25_MOESM13_ESM.zip › Appendix Figures/Appendix Figure S4/S4G/S4G AAV-blank Merge.tif]

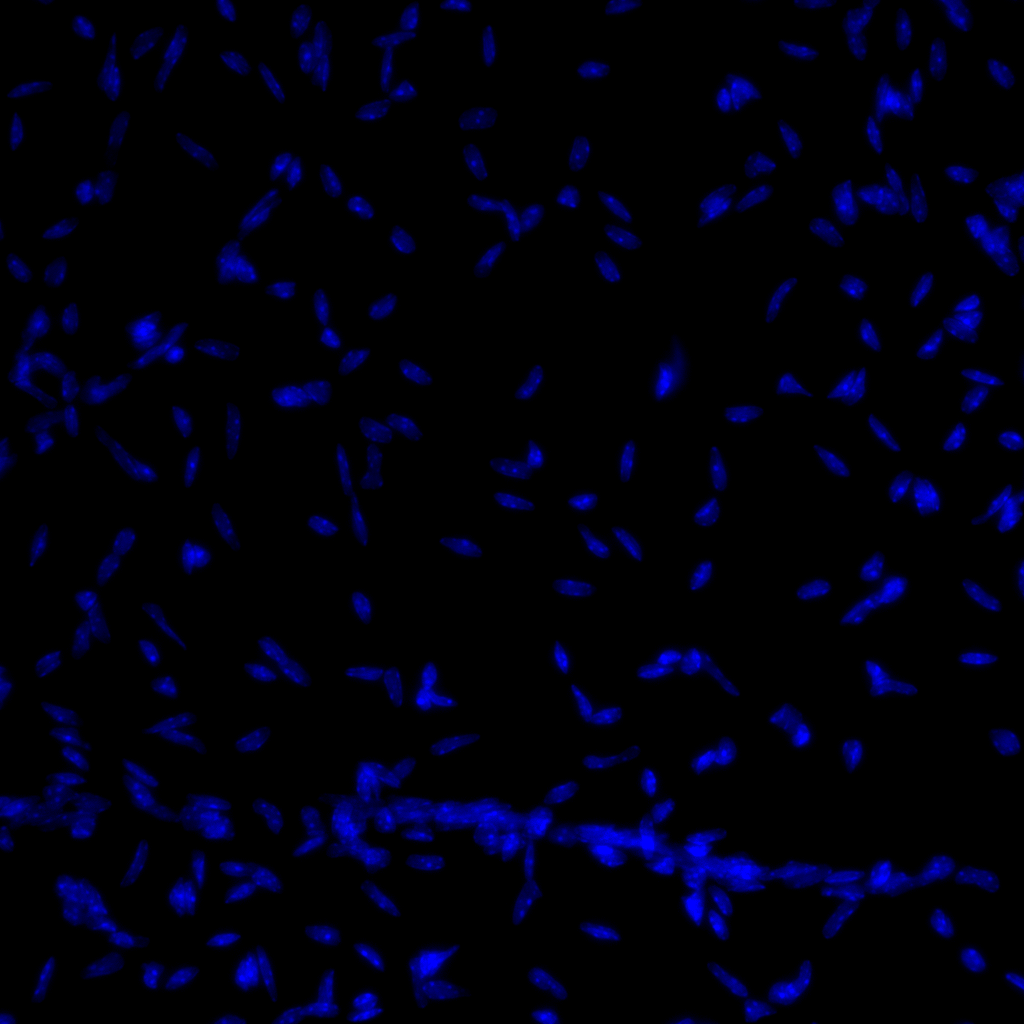

Supplement: Supplementary file 13 — Appendix Figures Source Data [file 44321_2024_25_MOESM13_ESM.zip › Appendix Figures/Appendix Figure S4/S4G/S4G AAV-Fto DAPI.tif]

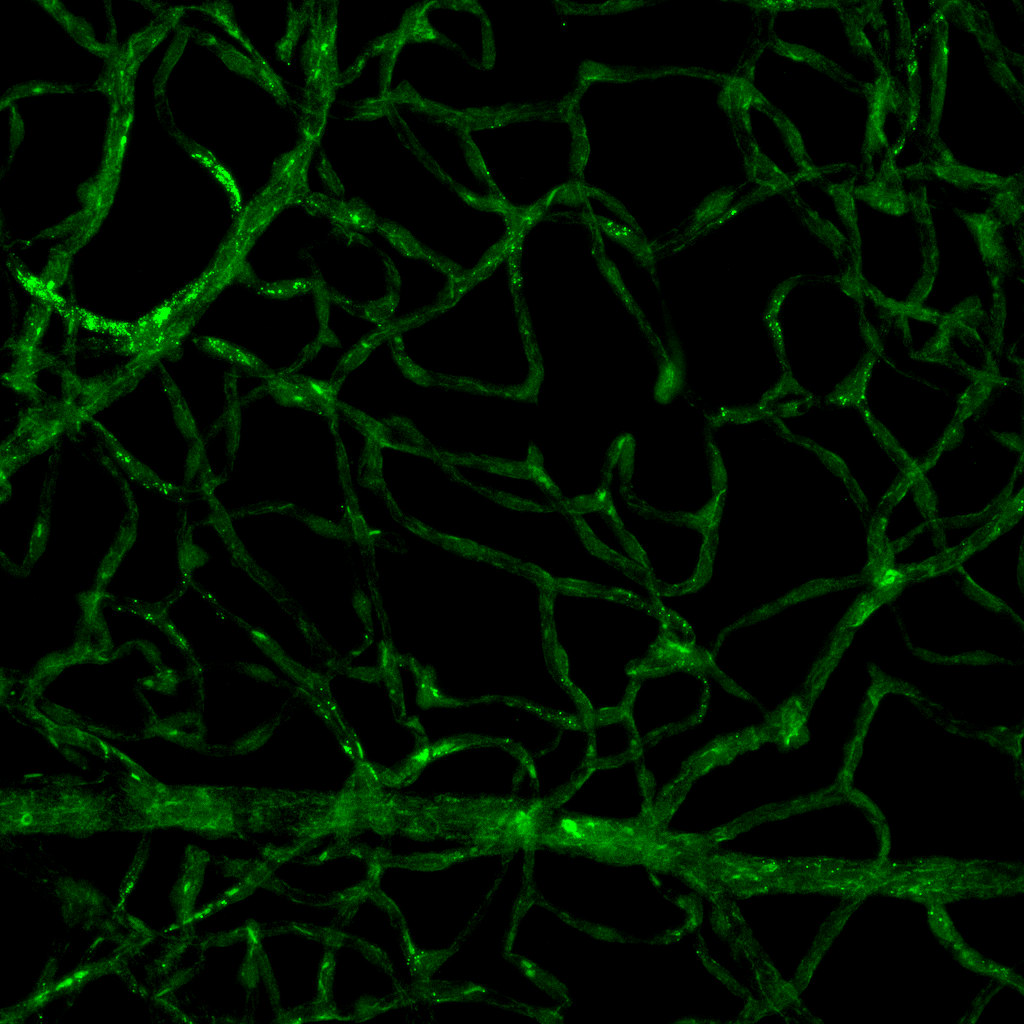

Supplement: Supplementary file 13 — Appendix Figures Source Data [file 44321_2024_25_MOESM13_ESM.zip › Appendix Figures/Appendix Figure S4/S4G/S4G AAV-Fto FLAG.tif]

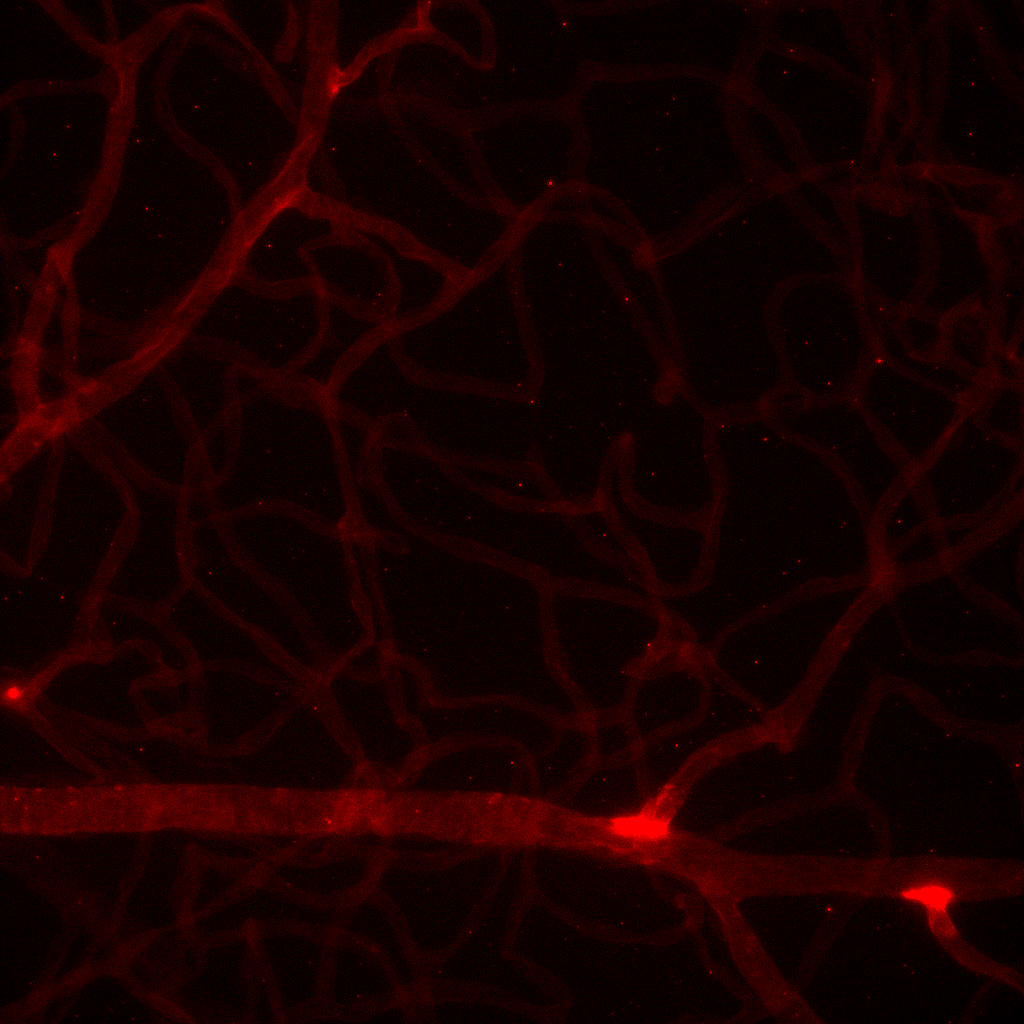

Supplement: Supplementary file 13 — Appendix Figures Source Data [file 44321_2024_25_MOESM13_ESM.zip › Appendix Figures/Appendix Figure S4/S4G/S4G AAV-Fto IB4.tif]

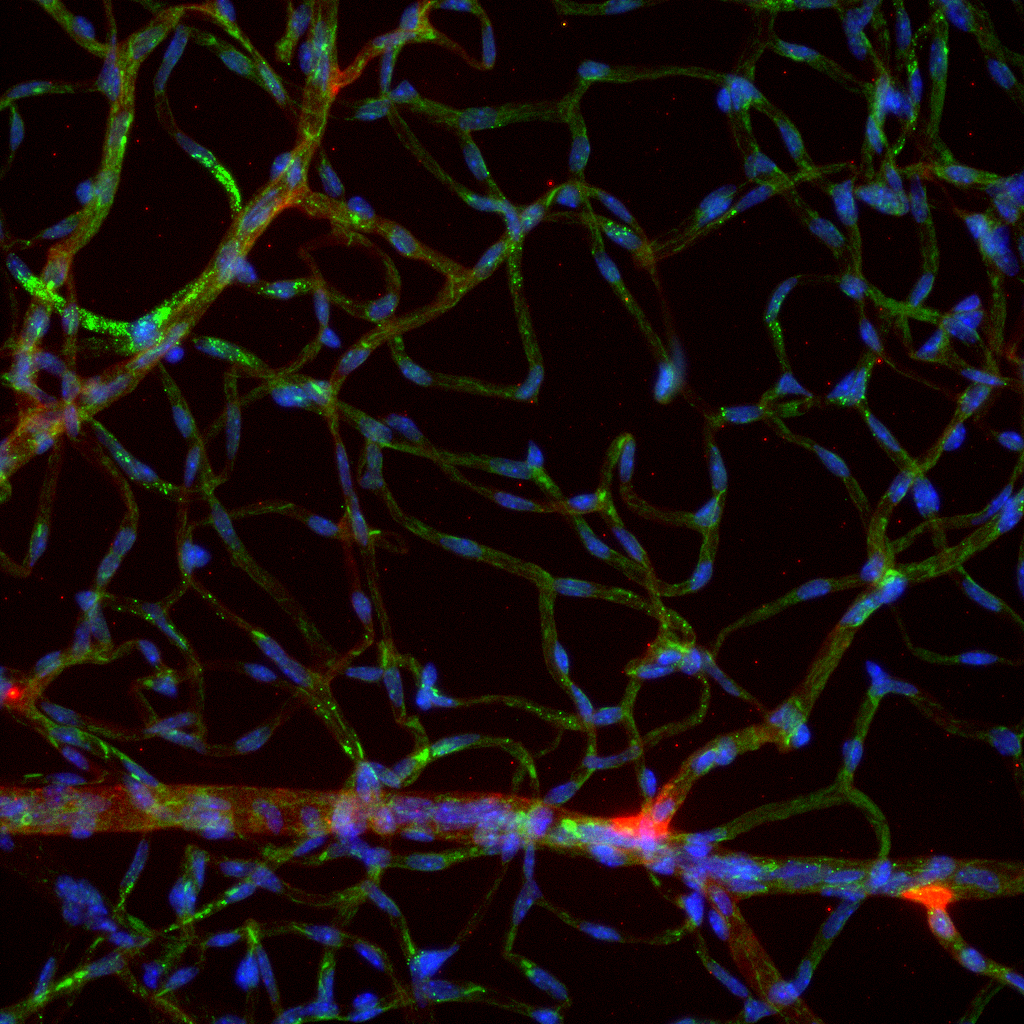

Supplement: Supplementary file 13 — Appendix Figures Source Data [file 44321_2024_25_MOESM13_ESM.zip › Appendix Figures/Appendix Figure S4/S4G/S4G AAV-Fto Merge.tif]

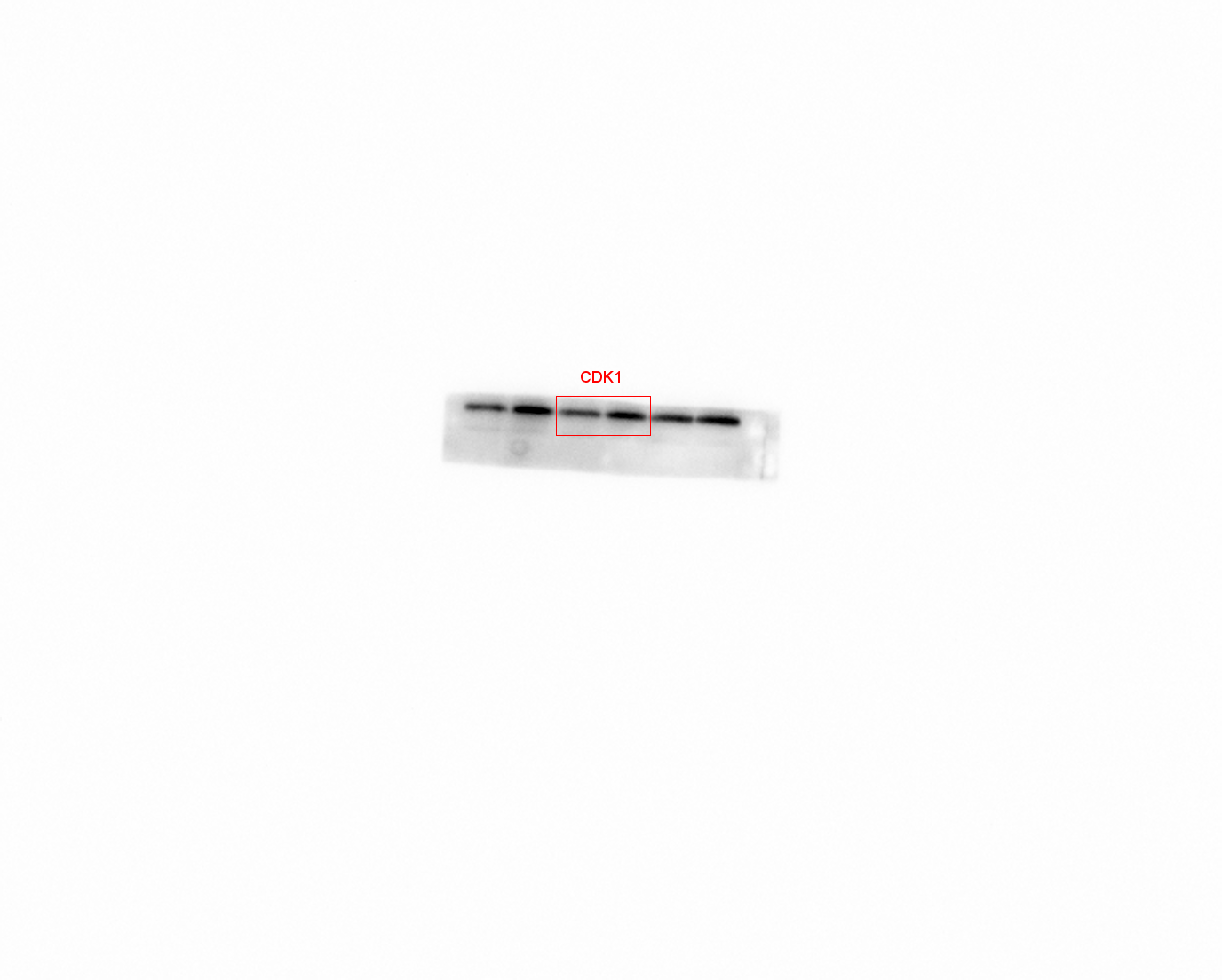

Supplement: Supplementary file 13 — Appendix Figures Source Data [file 44321_2024_25_MOESM13_ESM.zip › Appendix Figures/Appendix Figure X3/X1 A/X1A CDK1 mark.Tif]

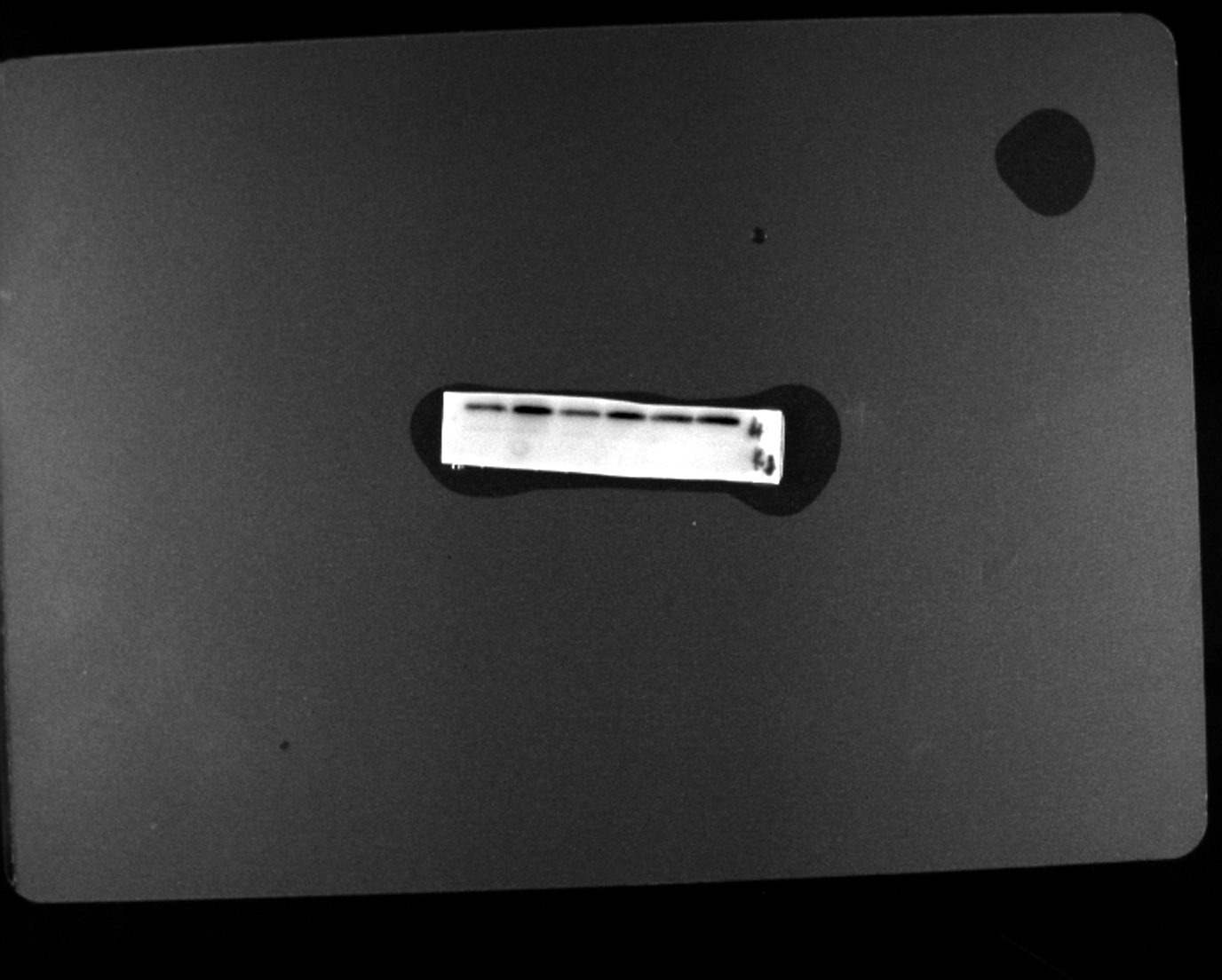

Supplement: Supplementary file 13 — Appendix Figures Source Data [file 44321_2024_25_MOESM13_ESM.zip › Appendix Figures/Appendix Figure X3/X1 A/X1A CDK1.Tif]

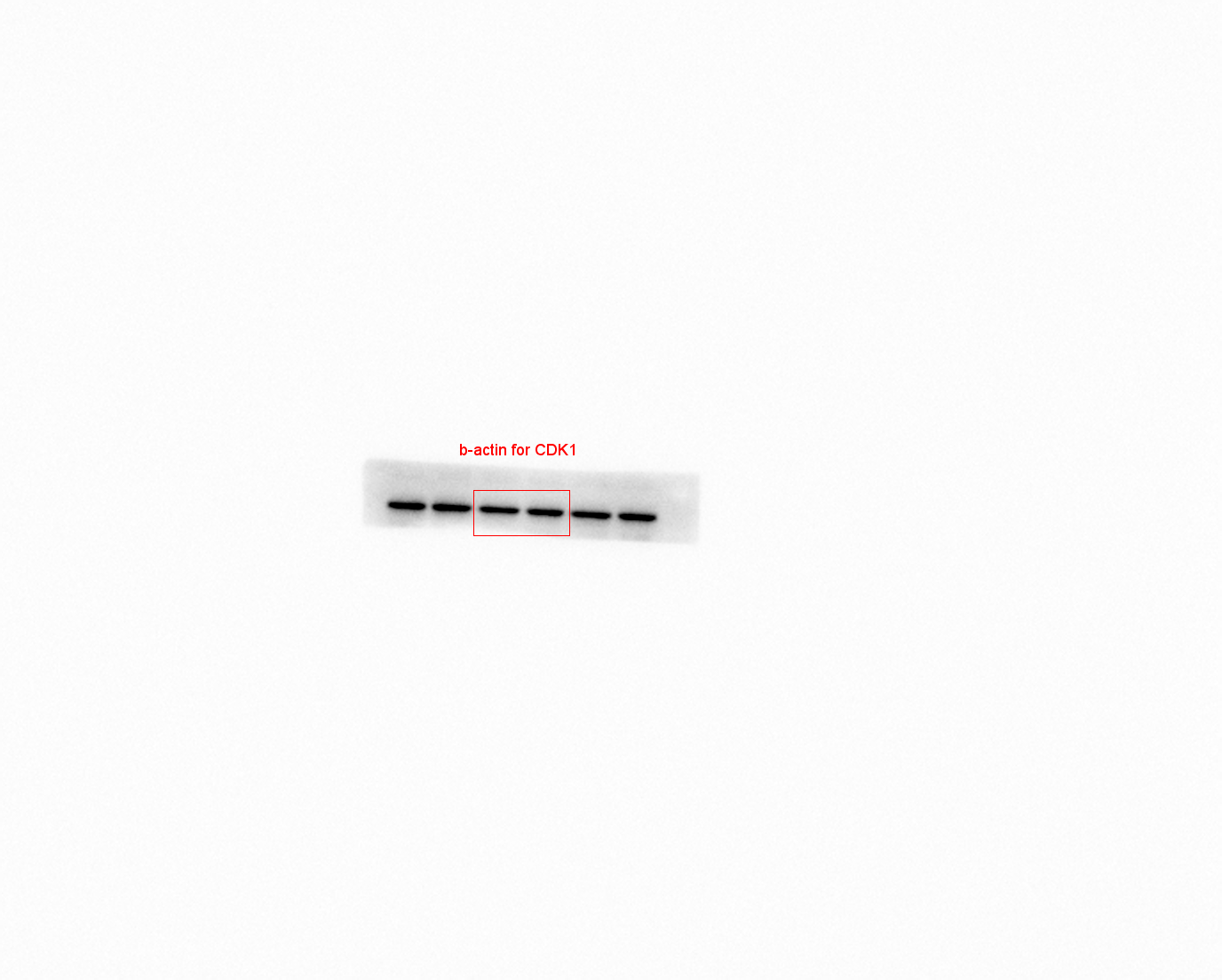

Supplement: Supplementary file 13 — Appendix Figures Source Data [file 44321_2024_25_MOESM13_ESM.zip › Appendix Figures/Appendix Figure X3/X1 A/X1A a┬-actin for CDK1 mark.Tif]

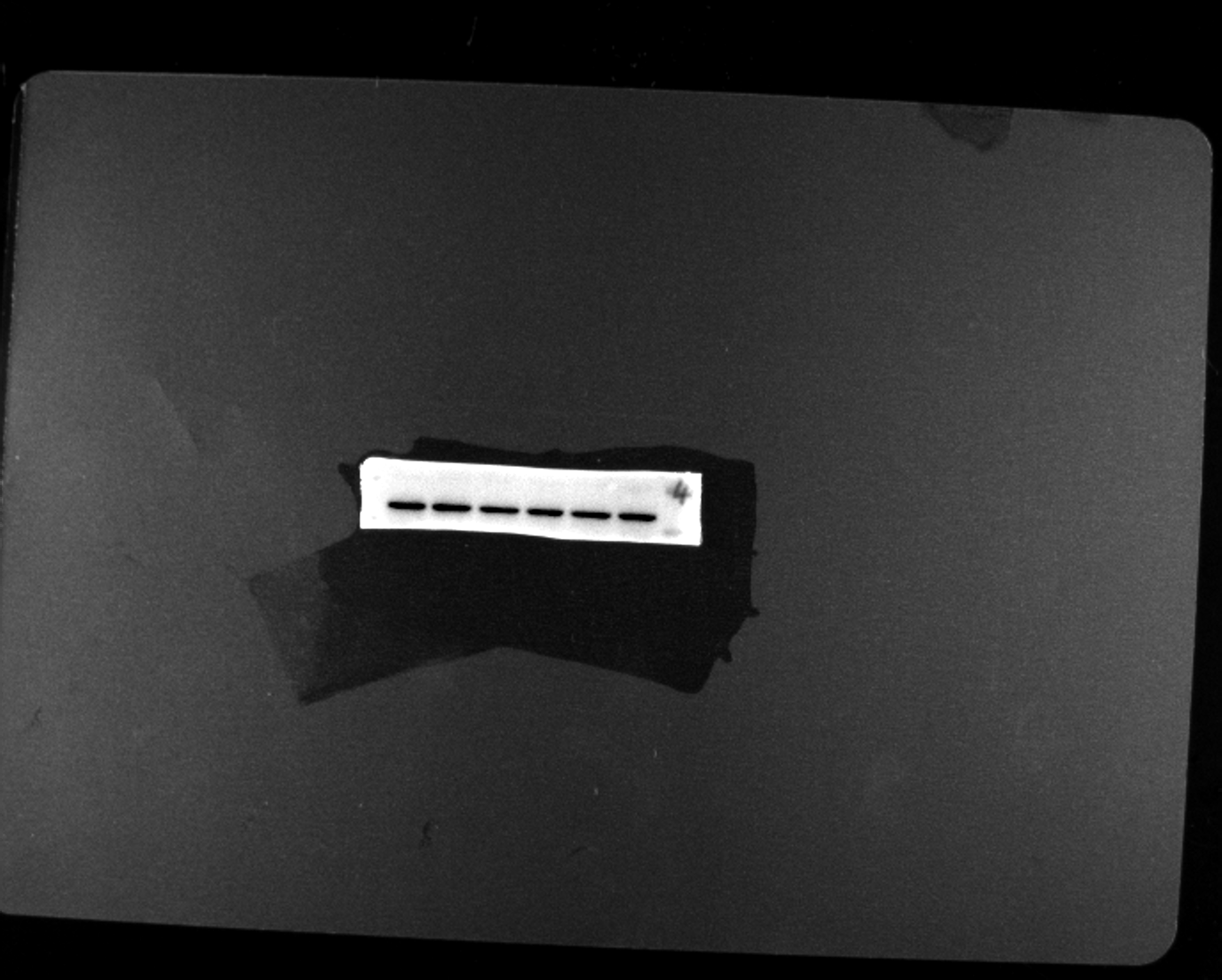

Supplement: Supplementary file 13 — Appendix Figures Source Data [file 44321_2024_25_MOESM13_ESM.zip › Appendix Figures/Appendix Figure X3/X1 A/X1A a┬-actin for CDK1.Tif]

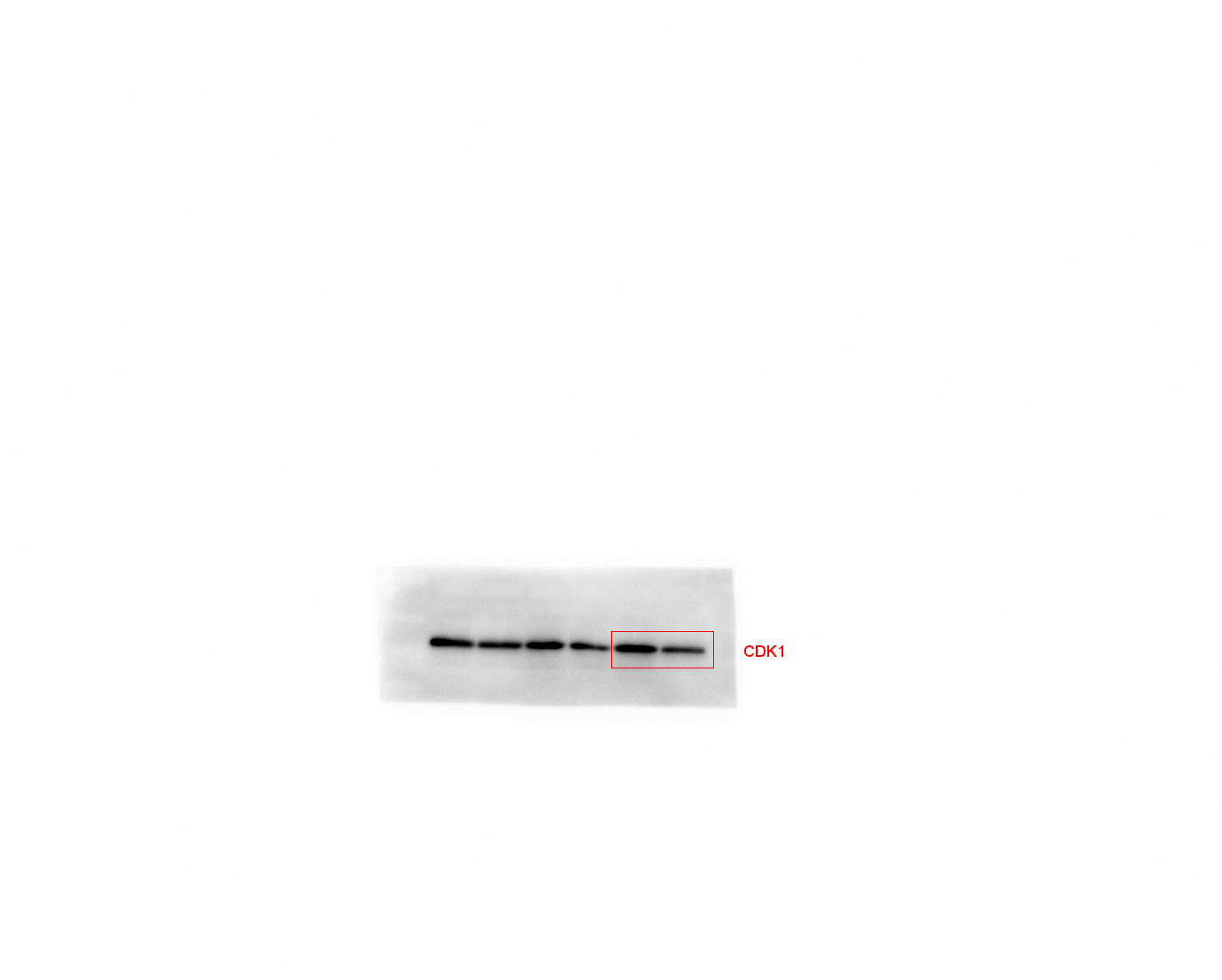

Supplement: Supplementary file 13 — Appendix Figures Source Data [file 44321_2024_25_MOESM13_ESM.zip › Appendix Figures/Appendix Figure X3/X1 B/X1B CDK1 mark.Tif]

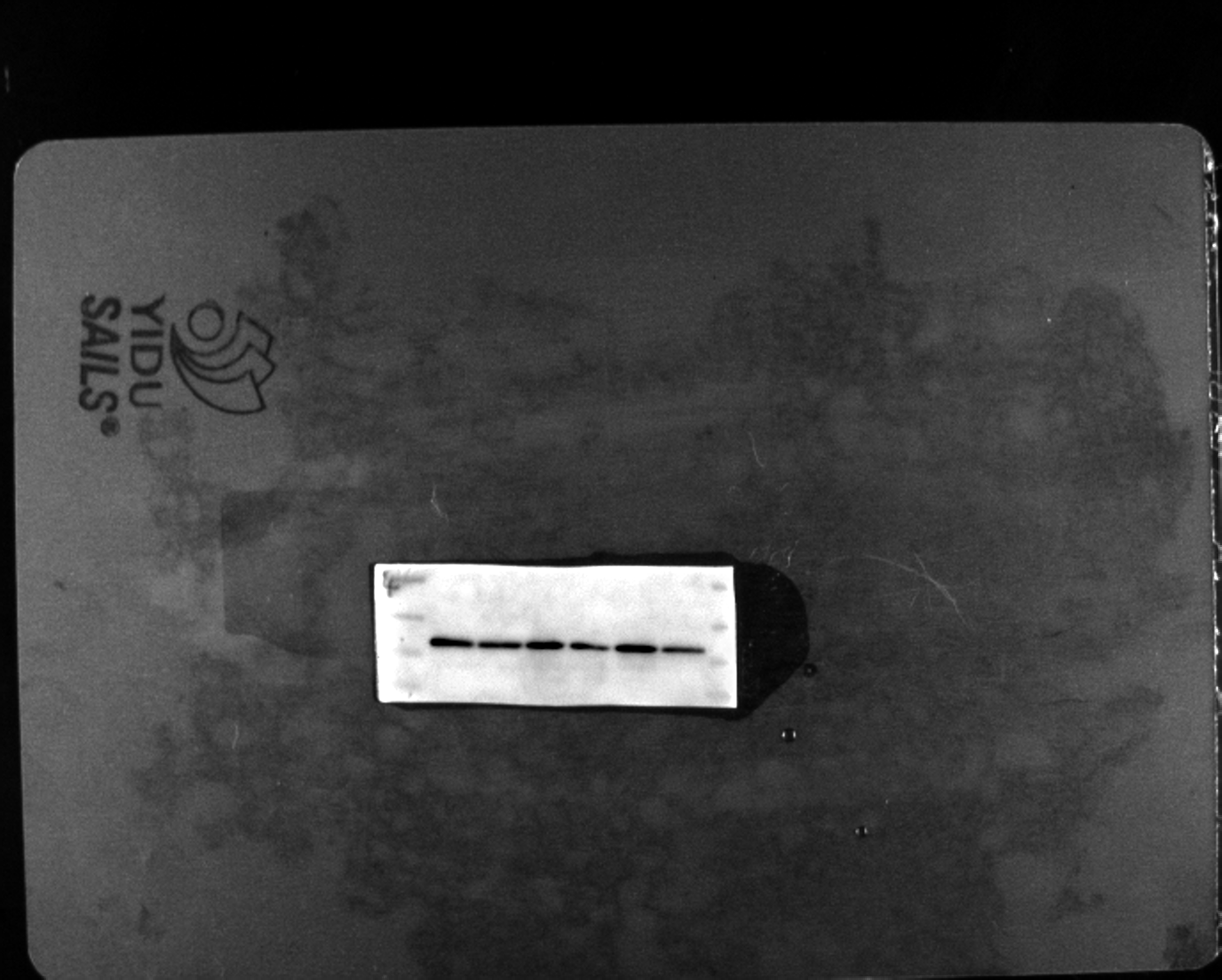

Supplement: Supplementary file 13 — Appendix Figures Source Data [file 44321_2024_25_MOESM13_ESM.zip › Appendix Figures/Appendix Figure X3/X1 B/X1B CDK1.Tif]

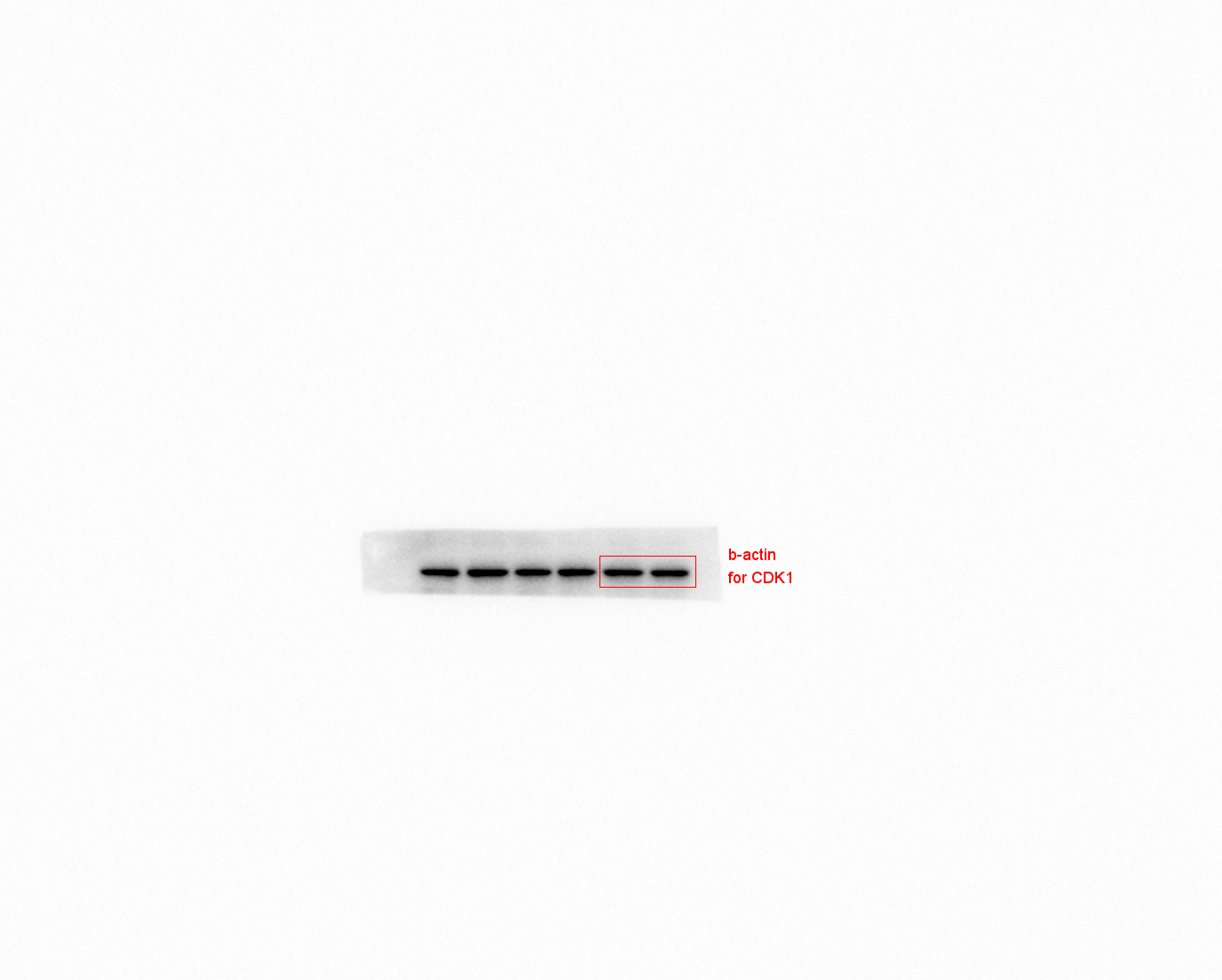

Supplement: Supplementary file 13 — Appendix Figures Source Data [file 44321_2024_25_MOESM13_ESM.zip › Appendix Figures/Appendix Figure X3/X1 B/X1B a┬-actin for CDK1 mark.Tif]

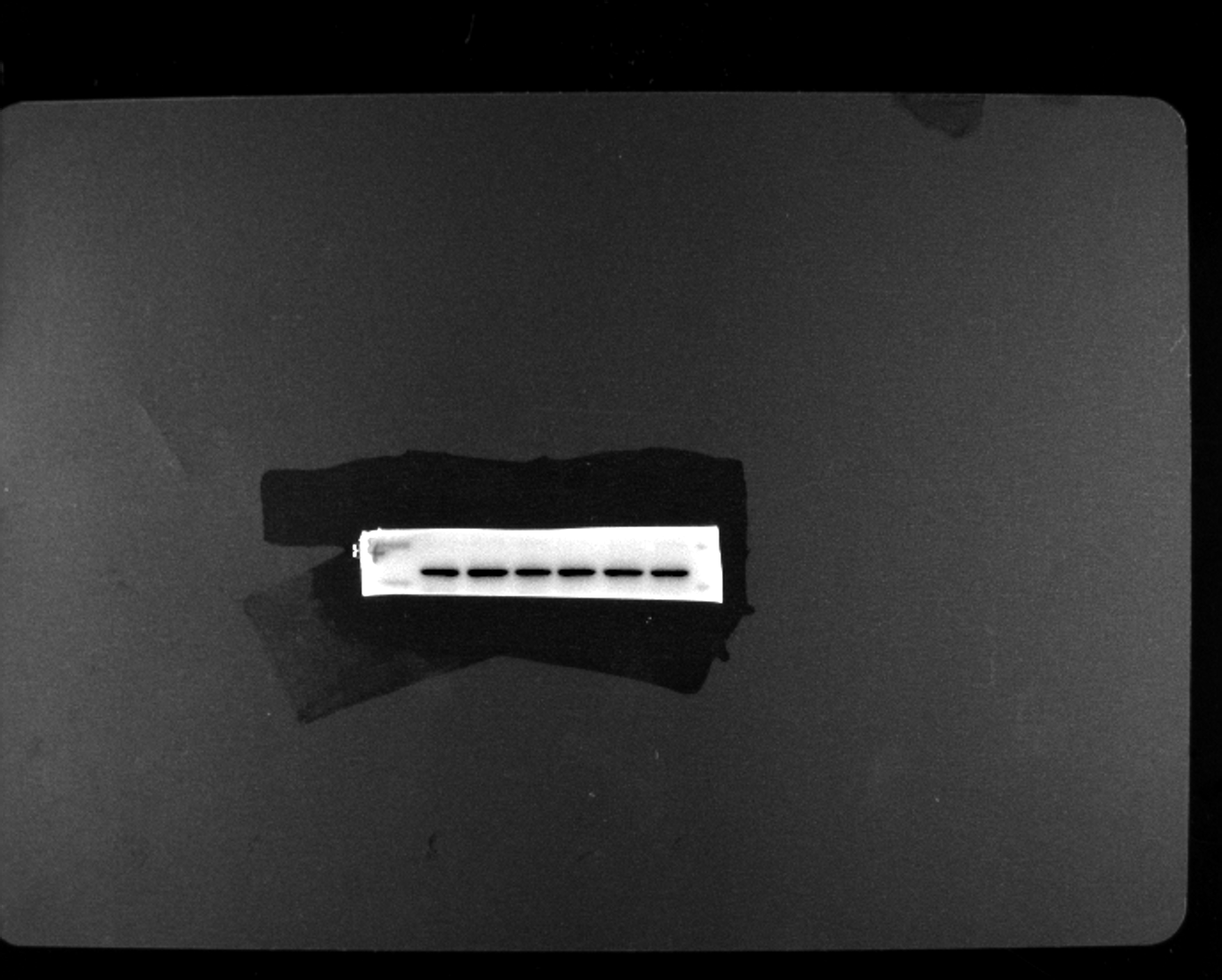

Supplement: Supplementary file 13 — Appendix Figures Source Data [file 44321_2024_25_MOESM13_ESM.zip › Appendix Figures/Appendix Figure X3/X1 B/X1B a┬-actin for CDK1.Tif]
