# Supplementary figures and images for: Author Correction: Lactylation-driven FTO targets CDK2 to aggravate microvascular anomalies in diabetic retinopathy
Source: EMBO Mol Med. 2025 Apr 25;17(6):1495–8. doi: 10.1038/s44321-025-00238-y (PMC12162844; doi:10.1038/s44321-025-00238-y)

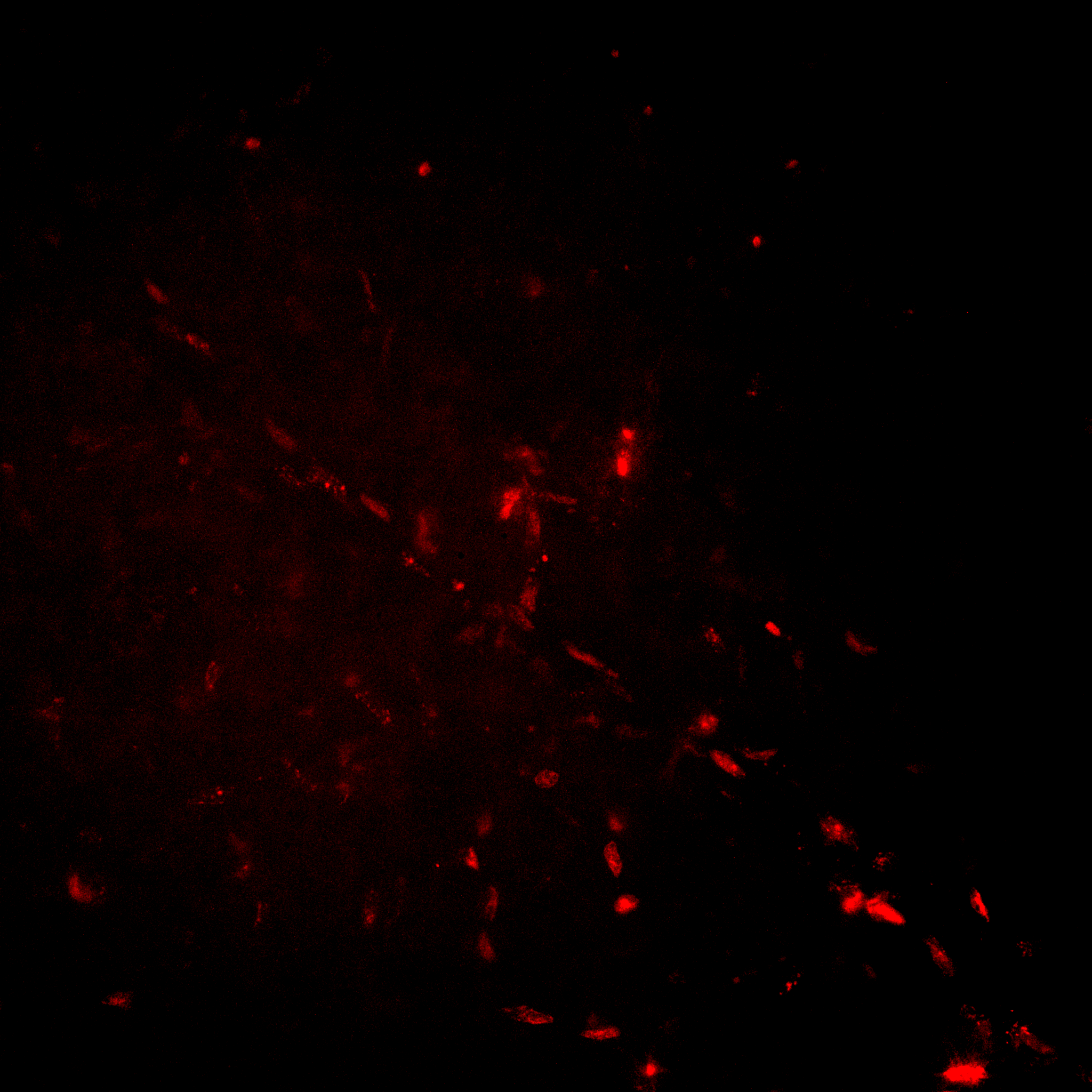

Supplement: Supplementary file 1 — source data for Figure 3C affected images [file 44321_2025_238_MOESM1_ESM.zip › source data for Figure 3C affected images/edu oir-2.tif]

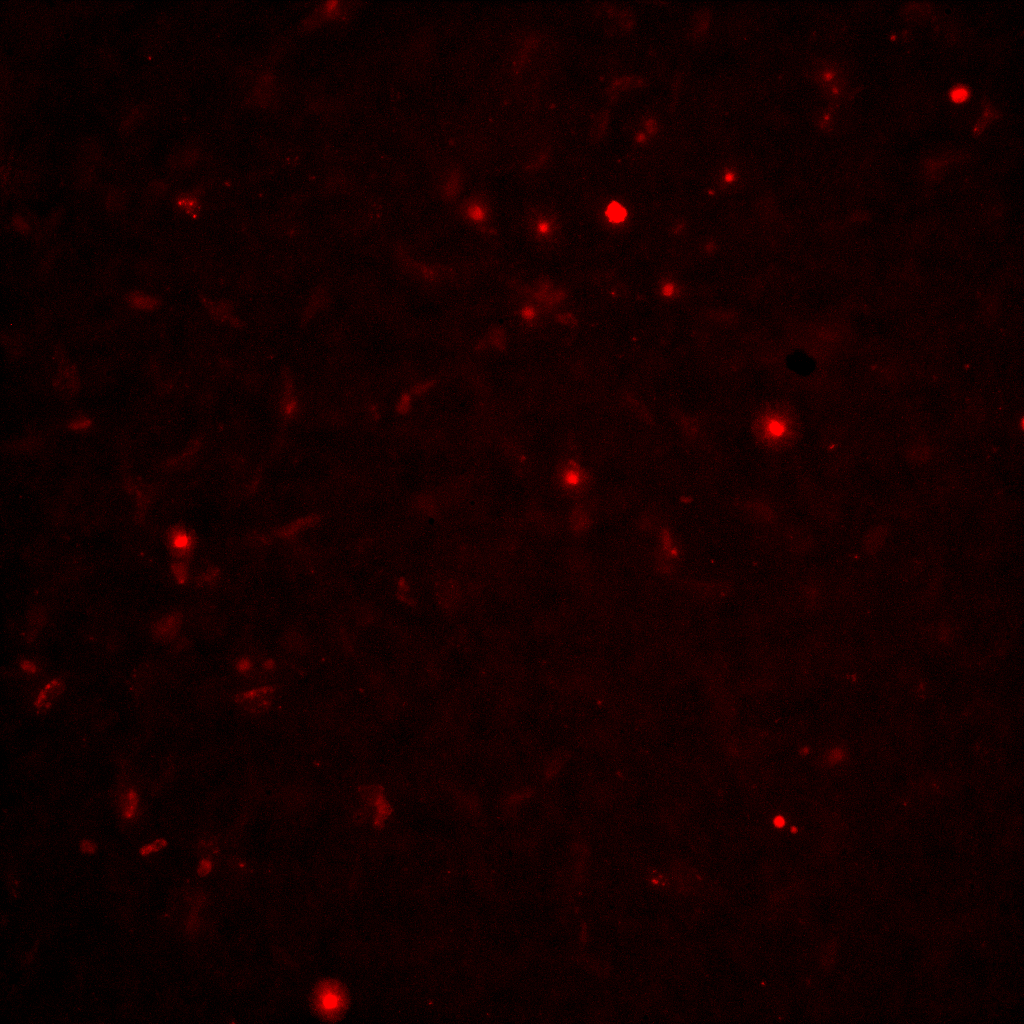

Supplement: Supplementary file 1 — source data for Figure 3C affected images [file 44321_2025_238_MOESM1_ESM.zip › source data for Figure 3C affected images/OIR blank-3-EdU.tif]

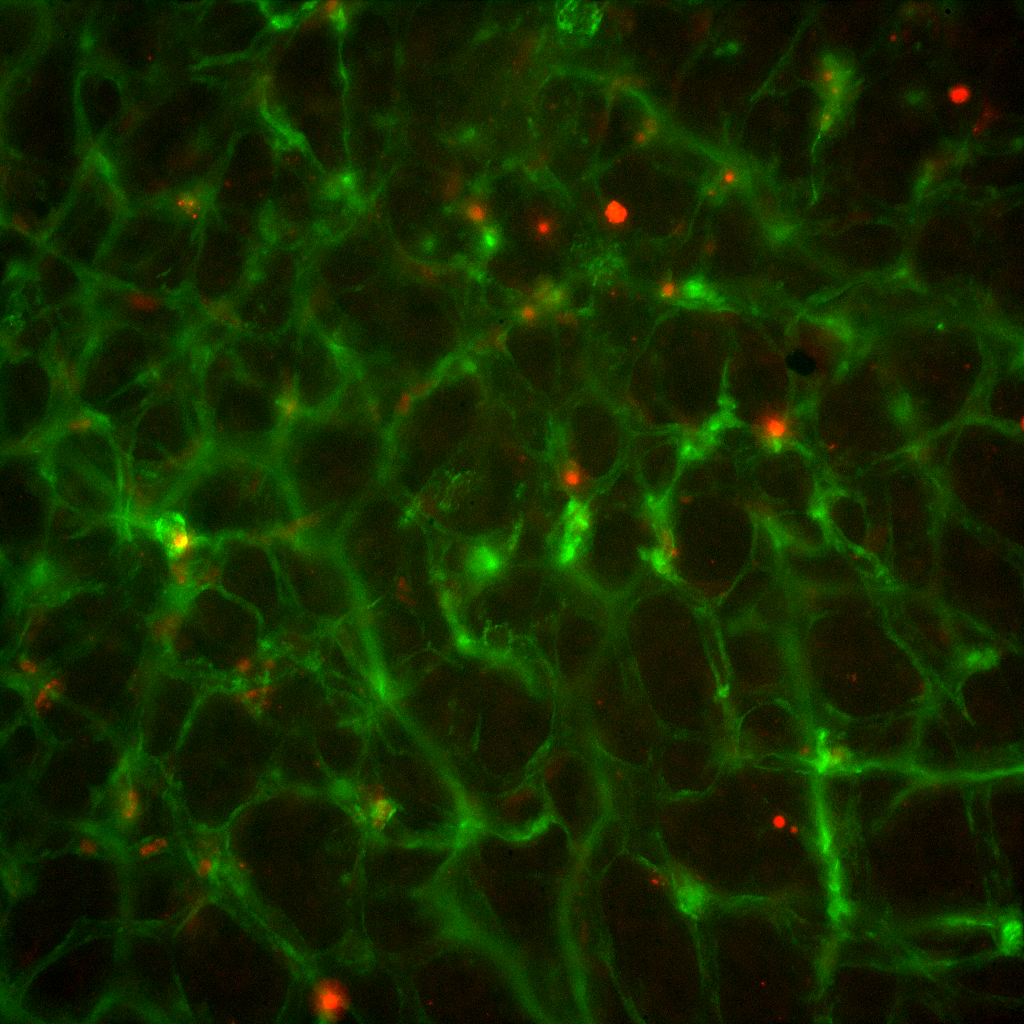

Supplement: Supplementary file 1 — source data for Figure 3C affected images [file 44321_2025_238_MOESM1_ESM.zip › source data for Figure 3C affected images/OIR blank-3-Merge.tif]

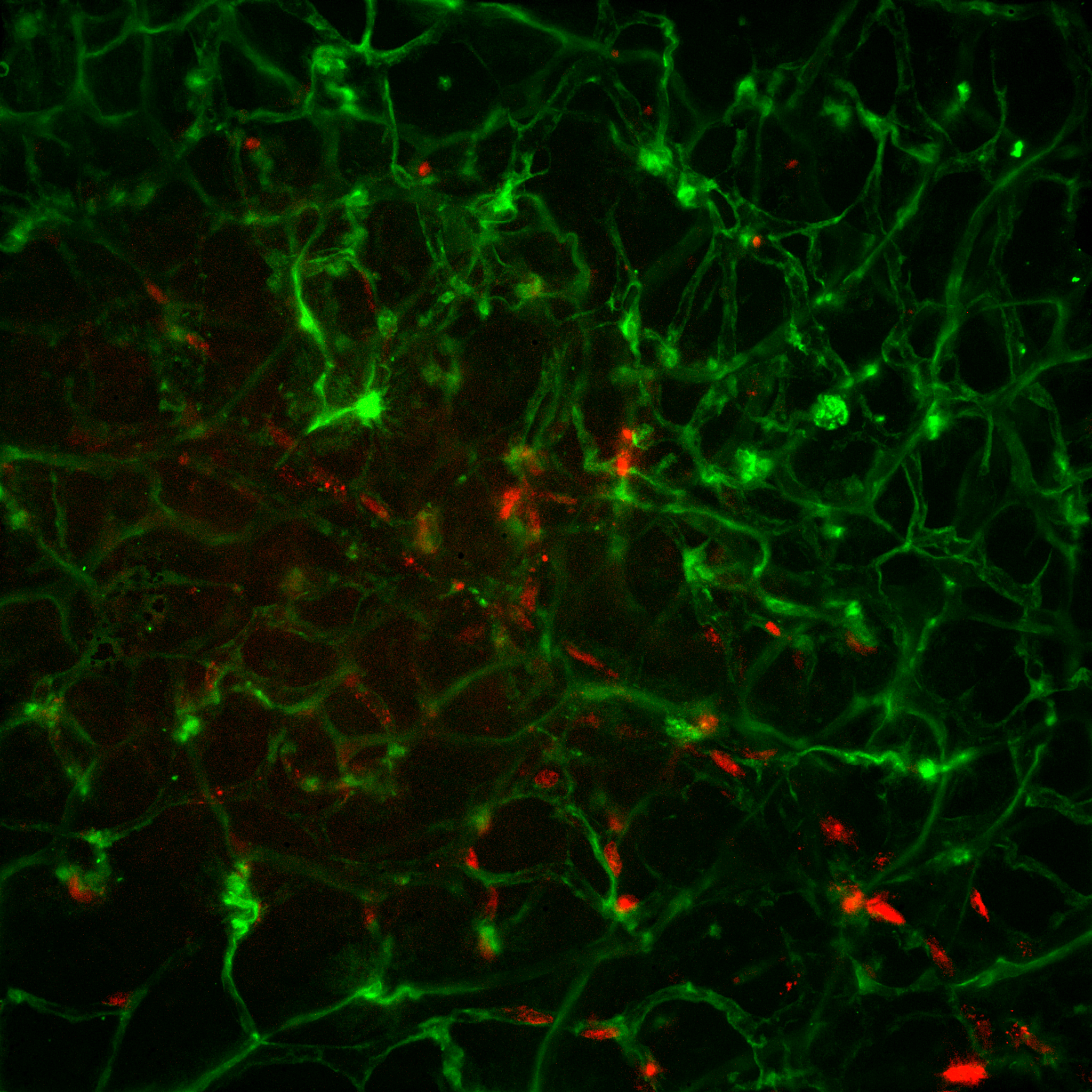

Supplement: Supplementary file 1 — source data for Figure 3C affected images [file 44321_2025_238_MOESM1_ESM.zip › source data for Figure 3C affected images/edu oir-2-merge.tif]

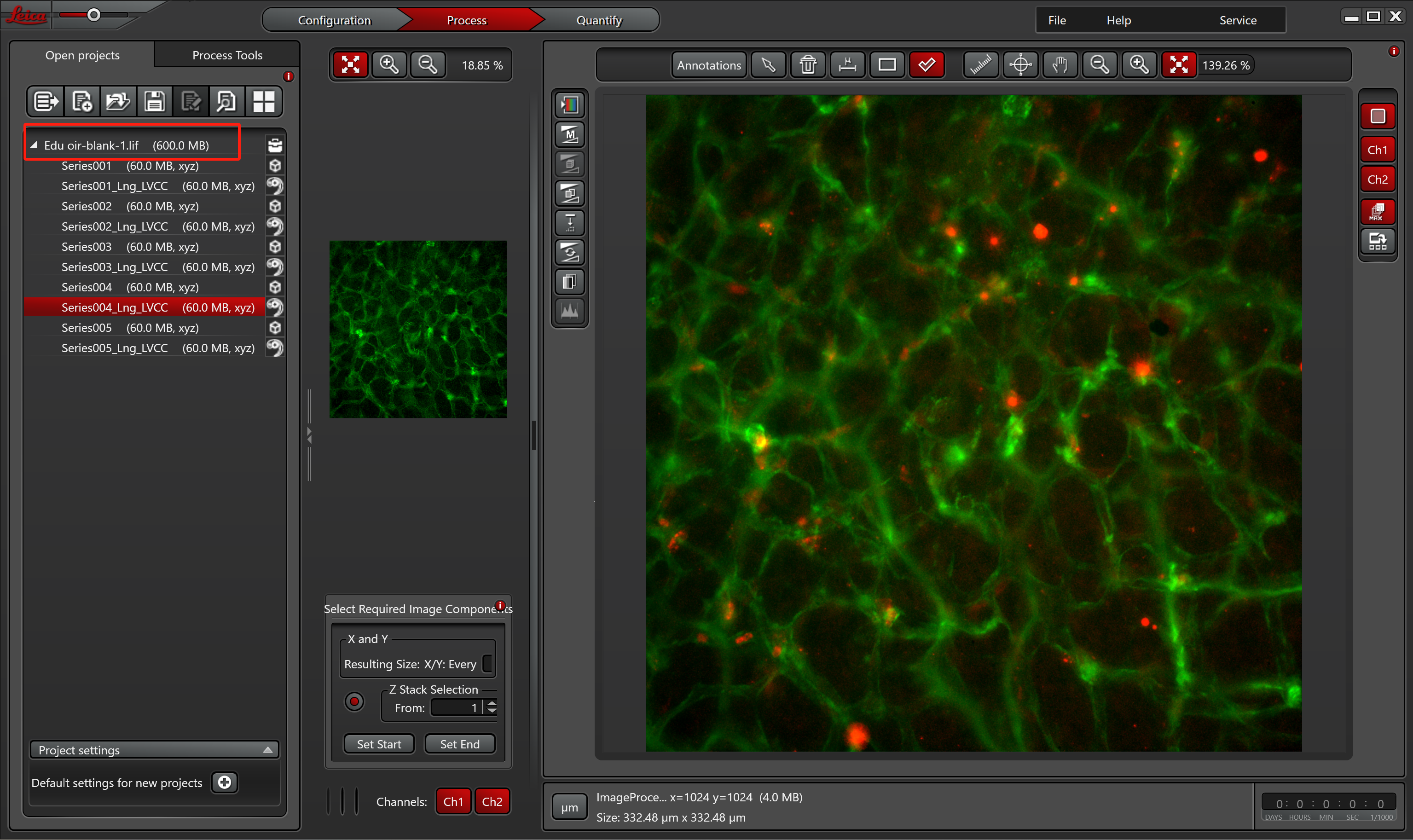

Supplement: Supplementary file 1 — source data for Figure 3C affected images [file 44321_2025_238_MOESM1_ESM.zip › source data for Figure 3C affected images/workbook images/7a16c501cfadb842bd23852cef37762.png]

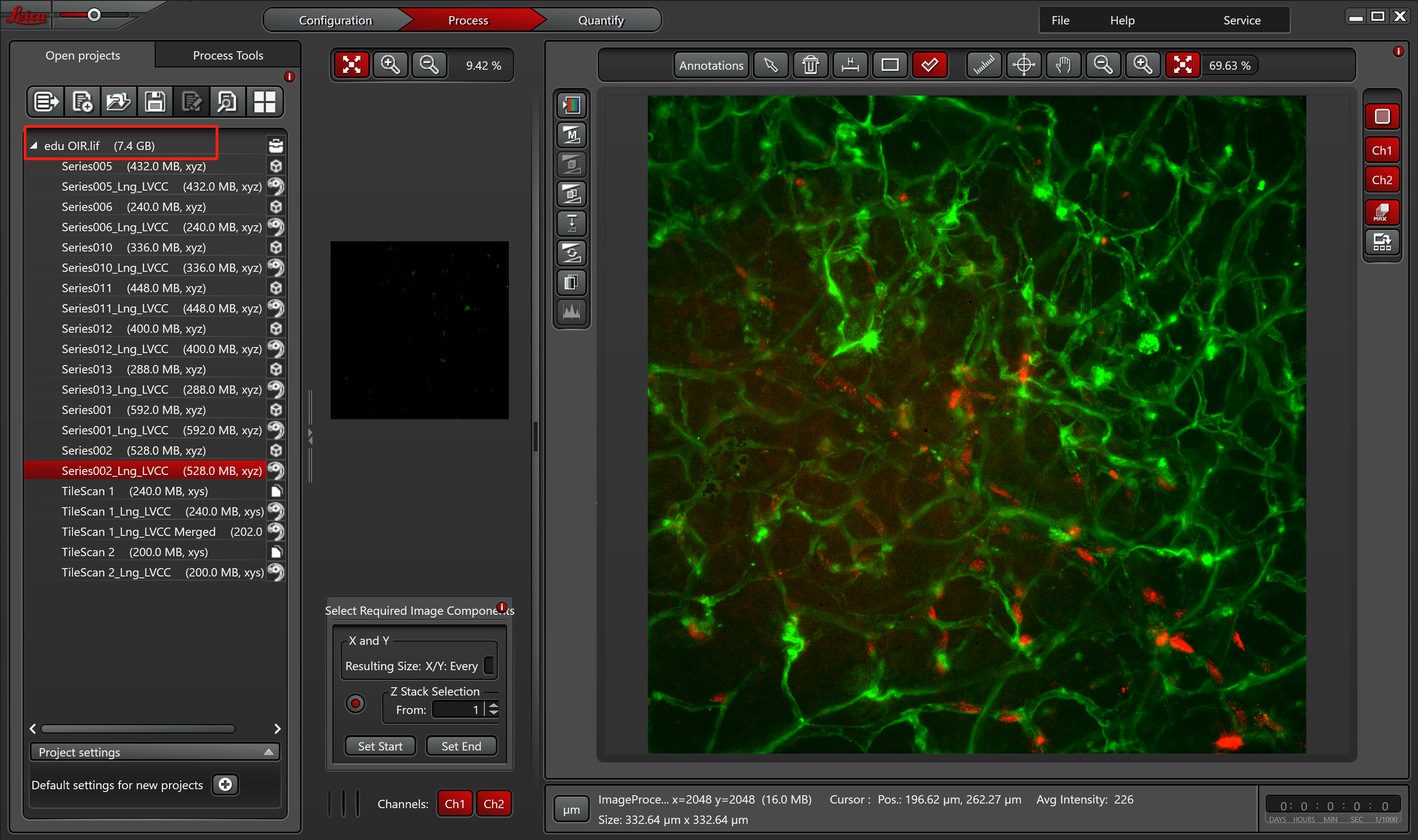

Supplement: Supplementary file 1 — source data for Figure 3C affected images [file 44321_2025_238_MOESM1_ESM.zip › source data for Figure 3C affected images/workbook images/39fe4f2af55d6edd063c0c1c548fbc5.png]

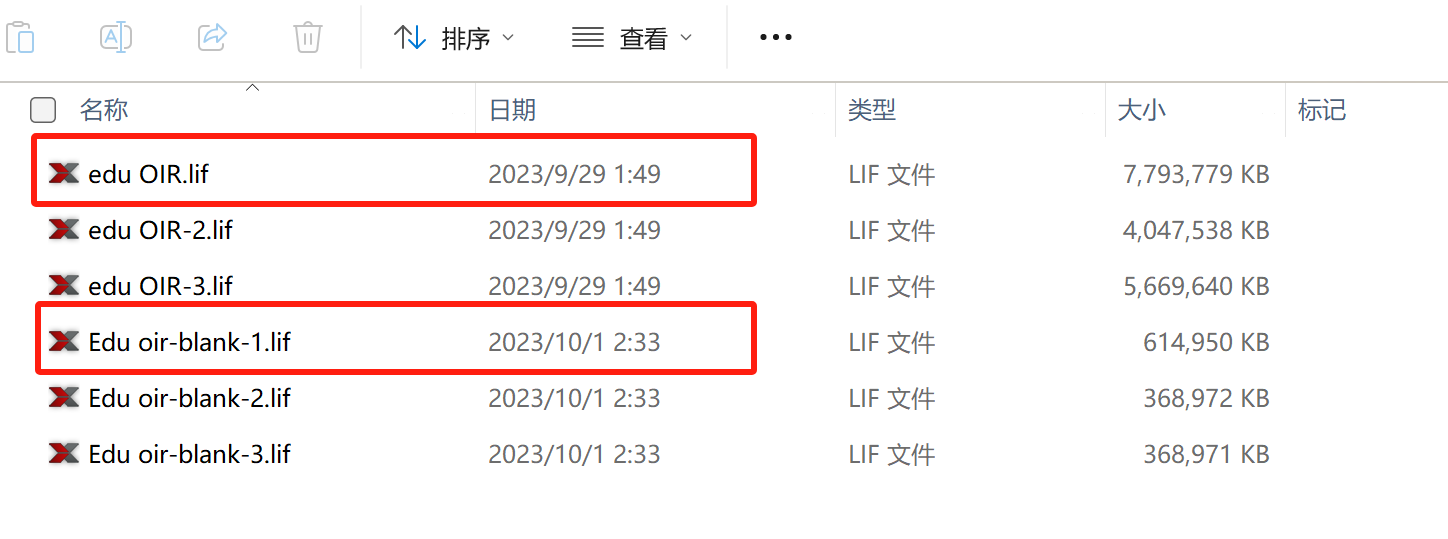

Supplement: Supplementary file 1 — source data for Figure 3C affected images [file 44321_2025_238_MOESM1_ESM.zip › source data for Figure 3C affected images/workbook images/68b518d06a54939fe86d62f63e16a3e.png]
